# Supplementary material for: Diagnostic accuracy of blood tests of inflammation in paediatric appendicitis: a systematic review and meta-analysis
Source: BMJ Open. 2022 Nov 2;12(11):e056854. doi: 10.1136/bmjopen-2021-056854 (PMC9639107; doi:10.1136/bmjopen-2021-056854)
Supplement: Supplementary data [file bmjopen-2021-056854supp009.pdf]

Supplementary Materials for "Diagnostic accuracy of blood tests in paediatric appendicitis – a systematic review and meta-analysis"

Overview of Supplementary materials

Supplementary Tables: 2

Supplementary Table 1 2

Supplementary Table 2: 8

Supplementary Table 3: 13

Supplementary Table 4: 18

Supplementary Table 5: 20

Supplementary Figures and legends: 21

Supplementary Figure 1: 21

Supplementary Figure 2: 22

Supplementary Figure 3 23

Supplementary Figure 4 24

Supplementary Figure 5 24

Supplementary Figure 6 24

Supplementary Figure 7 25

Supplementary Figure 8 25

Search Strategy: 26

QUADAS-2 Scoring criteria 28

Supplementary References 30

Fawkner-Corbett D, *et al. BMJ Open* 2022; 12:e056854. doi: 10.1136/bmjopen-2021-056854

Supplementary Tables:

Supplementary Table 1: Overview of studies excluded at full paper review stage and reason(s) for exclusion (* = Author was contacted and confirmed exclusion or did not respond. Unsuitable control group also includes those studies with no control group)

First Author	Year	Exclusion Reason 1	Exclusion Reason 2
1) Abu-Eshy	1995	Incompatible age demographics	Unsuitable control group
2)Barker	1998	Unsuitable control group	Incompatible age demographics
3)Bolandparvaz	2004	Incompatible age demographics	
4)Bower	1981	Unsuitable control group	
5)Clavien	1987	Incompatible age demographics	Unsuitable control group
6)Cohen*	2015	Data not available in 2x2	
7)Depinet*	2016	Data not available in 2x2	
8)Erdem	2015	Incompatible age demographics	
9)Ficek	1958	Unsuitable control group	
10) Kharbanda	2016	Study population already included	
11) Lofvenberg	2016	Unsuitable control group	Data not available in 2x2
12) Ng	2002	Incompatible age demographics	
13) Park	2010	Incompatible age demographics	
14) Vegar-Zubovic	2003	Unsuitable control group	
15) Wattanasirichaigoon	1994	Incompatible age demographics	
16) Anielski	2010	Incompatible age demographics	
17) Atema	2015	Incompatible age demographics	
18) Kessler	2004	Incompatible age demographics	
19) Nasiri	2012	Incompatible age demographics	
20) Thuijls	2011	Incompatible age demographics	
21) Yardimci	2016	Incompatible age demographics	
22) Yorke*	2016	Abstract	Incompatible age demographics

Fawkner-Corbett D, *et al. BMJ Open* 2022; 12:e056854. doi: 10.1136/bmjopen-2021-056854

23) Young	1986	Review	
24) Agrawal	2008	Incompatible age demographics	Unsuitable control group
25) Albu	1994	Incompatible age demographics	Data not available in 2x2
26) Allister*	2011	Incompatible age demographics	Data not available in 2x2
27) Amland	1989	Incompatible age demographics	
28) Atema	2015	Incompatible age demographics	Unsuitable control group
29) Bakal*	2016	Data not available in 2x2	
30) Bealer	2010	Incompatible age demographics	
31) Beardsley*	2014	Abstract	
32) Beardsley*	2012	Abstract	
33) Bhatt*	2009	Data not available in 2x2	
34) Birkhahn	2006	Incompatible age demographics	
35) Blab*	2004	Data not available in 2x2	
36) Cardall	2004	Incompatible age demographics	
37) Chakhunashvili	2005	Unsuitable control group	
38) Chandel	2011	Unsuitable control group	
39) Colvin*	2007	Data not available in 2x2	
40) D'Souza	2013	Incompatible age demographics	
41) Davies	1991	Incompatible age demographics	
42) Dueholm	1989	Incompatible age demographics	
43) Duzgun	2007	Incompatible age demographics	
44) Eriksson	1994	Incompatible age demographics	
45) Eriksson	1995	Incompatible age demographics	
46) Eriksson	1995	Incompatible age demographics	
47) Erkasap	2000	Incompatible age demographics	
48) Escriba*	2011	Data not available in 2x2	
49) Gendel	2011	Unsuitable control group	Data not available in 2x2

Fawkner-Corbett D, *et al. BMJ Open* 2022; 12:e056854. doi: 10.1136/bmjopen-2021-056854

50) Groselj-Grenc	2007	Study population already included	
51) Gurau	2016	Unsuitable control group	Data not available in 2x2
51) Hallan	1997	Incompatible age demographics	
53) Hotic	2012	Unsuitable control group	Data not available in 2x2
54) Hu*	2014	Data not available in 2x2	
55) Huang*	2012	Data not available in 2x2	
56) Jahn	1997	Incompatible age demographics	
57) Jamaluddin	2013	Incompatible age demographics	
58) Johansson	2007	Incompatible age demographics	
59) John	1993	Incompatible age demographics	
60) Khan	2004	Incompatible age demographics	
61) Khan	2009	Incompatible age demographics	Unsuitable control group
62) Kharbanda*	2011	Data not available in 2x2	
63) Kosloske*	2004	Data not available in 2x2	
64) Kostic*	2010	Data not available in 2x2	
65) Kyriakidis	2010	Incompatible age demographics	
66) Lam	2014	Incompatible age demographics	
67) Li	2005	Unsuitable control group	Data not available in 2x2
68) Li	2012	Unsuitable control group	
69) Lietzen	2016	Incompatible age demographics	
70) Lin*	2007	Data not available in 2x2	
71) Mollitt*	1988	Data not available in 2x2	
72) Oosterhuis	1993	Incompatible age demographics	
73) Patel	2014	Unsuitable control group	
74) Raftery	1976	Incompatible age demographics	Unsuitable control group
75) Ring-Mrozik*	1991	Unsuitable control group	
76) Sack*	2006	Unsuitable control group	

Fawkner-Corbett D, *et al. BMJ Open* 2022; 12:e056854. doi: 10.1136/bmjopen-2021-056854

77) Sarsu*	2017	Unsuitable control group	
78) Schellekens	2013	Incompatible age demographics	
79) Socea	2013	Incompatible age demographics	
80) Sondenaa	1992	Incompatible age demographics	
81) Suhaymi	2017	Unsuitable control group	Incompatible age demographics
82) Summa	2007	Incompatible age demographics	
83) Tamanna	2012	Incompatible age demographics	
84) Tanrikulu	2014	Incompatible age demographics	
85) Tepel	2004	Incompatible age demographics	
86) Thimsen	1989	Incompatible age demographics	
87) Thompson	1992	Incompatible age demographics	
88) Turkyilmaz	2000	Unsuitable control group	
89) Uyanik	2012	Unsuitable control group	
90) van den Broek	2002	Study population already included	
91) Wu	2010	Unsuitable control group	
92) Wu	2006	Incompatible age demographics	
93) Xharra	2012	Incompatible age demographics	
94) Zviedre*	2014	Abstract	
95) Brown*	2004	Abstract	
96) Hodgkinson*	2011	Abstract	
97) Hodgkinson*	2011	Abstract	
98) Akin	2018	Unsuitable control group	
99) Aydin	2017	Incompatible age demographics	
100) Favot	2017	Review	
101) Kharbanda*	2018	Study population already included	Data not available in 2x2
102) Mikaere	2018	Incompatible age demographics	
103) Nazik	2017	Unsuitable control group	

Fawkner-Corbett D, *et al. BMJ Open* 2022; 12:e056854. doi: 10.1136/bmjopen-2021-056854

104) Nia	2018	Unsuitable control group	
105) Salih	2017	Incompatible age demographics	Unsuitable control group
106) Zouari	2017	Unsuitable control group	
107) Daly	2018	Unsuitable control group	
108) Elmas	2017	Unsuitable control group	
109) Garcia Gamiz*	2016	Abstract	
110) Oztan	2018	Unsuitable control group	
111) Shommu	2018	Unsuitable control group	
112) Yu*	2018	Unsuitable control group	
113) Zouari	2018	Unsuitable control group	
114) Sola	2017	Unsuitable control group	
115) Zahoor*	2018	Abstract	
116) van Dieijen-Visser	1991	Incompatible age demographics	
117) Snyder	1999	Review	
118) Sengupta	2009	Incompatible age demographics	Data not available in 2x2
119) Scott	2015	Incompatible age demographics	
120) Randall Bond*	1990	Data not available in 2x2	
121) Msolli	2018	Incompatible age demographics	
122) Malik	1998	Incompatible age demographics	
123) Macco*	2016	Data not available in 2x2	
124) Lee	2008	Abstract	Incompatible age demographics
125) Kohn	2005	Incompatible age demographics	
126) Jang	2008	Incompatible age demographics	
127) Haghi	2018	Incompatible age demographics	
128) Blitman*	2015	Data not available in 2x2	
129) Akturk	2018	Incompatible age demographics	
130) Ahmed	2017	Unsuitable control group	

131) Agilinko	2019	Abstract	
132) Benito	2020	Data not available in 2x2	
133) Boettcher	2022	Data not available in 2x2	
134) Dal	2019	Incompatible age demographics	
135) Davis	2021	Data not available in 2x2	
136) Davis	2019	Abstract	
137) Dooki	2021	Unsuitable control group	
138) Duman	2022	Unsuitable control group	
139) Duman	2020	Unsuitable control group	
140) Güney	2019	Data not available in 2x2	
141) Lima	2019	Unsuitable control group	
142) Morandi	2020	Unsuitable control group	
143) Musbahi	2019	Unsuitable control group	Data not available in 2x2
144) Najd Sepas	2019	Unsuitable control group	Data not available in 2x2
145) Nawaz	2021	Abstract	
146) Ng	2019	Abstract	
147) Pehlivanli	2019	Incompatible age demographics	Unsuitable control group
148) Saleem	2021	Incompatible age demographics	Unsuitable control group
149) Altali Alhames	2021	Study population already included	

Fawkner-Corbett D, *et al. BMJ Open* 2022; 12:e056854. doi: 10.1136/bmjopen-2021-056854

Supplementary Table 2: Overview of demographics of included studies describing design, setting and population characteristics (* = data not provided or none described. AA= acute appendicitis. (P)ED = (Paediatric) Emergency Department. US = Ultrasound. CT = Computed Tomography. PICU= Paediatric Intensive Care Unit. In studies where authors provided paediatric data sub-population information is shown in square brackets.)

Study	Study Design; Setting; Country	No. of children in study; with AA (% prevalence)	Age range (years)	Gender (n (%) male)	Inclusion criteria	Exclusion criteria
Akgül, 2021 (1)	Prospective cohort study; PED; Turkey	320, 190 (59%)	2 to 18	201 (63%)	Acute abdominal pain and Paediatric Appendicitis Score (PAS) above 3 points	Previous appendicectomy. Abdominal trauma. Malignancies. Inflammatory intestinal disease. Familial Mediterranean Fever.
Altali, 2017 (2)	Prospective cohort study; Hospital ED; Spain	331, 116 (35%)	2 to 20	175 (53%)	Abdominal pain <72 hours suspicious of AA as reviewed by doctor	Radiology prior to presentation. Pregnancy. Previous appendicectomy. Cancer. Trauma. Surgery in last 7 days. Steroids, immunosuppressive treatment or chemotherapy.
Anandalwar, 2015 (3)	Retrospective cohort study; ED; USA	845, 393 (47%)	3 to 18	* (48%)	Consultation by surgeon for suspected AA. Underwent abdominal US.	History of abdominal surgery. Cognitive delay. Neurologic impairment. Immunodeficiency. Antibiotic use. Missing laboratory data. Abdominal imaging before presentation.
Andersson, 1999 (4)	Prospective cohort study; ED, 2 centres; Sweden	496,[data provided n=296 <18yrs] 79(27%)	10 to 86	133 (45%)	Patients admitted with suspected AA	*
Andersson, 2008 (5)	Prospective cohort study; Hospital, 2 centres; Sweden	545 [data provided n=436 <18yrs, 140 of which not included in Andersson 1999] , 191[25](18%)	All	58 (41%)	Admitted with suspected AA	Patients with missing blood tests.
Andersson, 2014 (6)	Prospective cohort study; Hospital ED, 2 centres; Sweden	428 [data provided n=259 children], 177[76](30%)	4 to 84	133 (49%)	Abdominal pain <5 days suggestive of AA	Pregnancy. Inability to consent. Multiple missing variables.
Andersson, 2017 (7)	Prospective interventional study with a nested randomized trial; ED, 21 centres; Sweden	3791[data provided n=1046 children], [338](33%)	5 to 95	2052 (54%)	Acute abdominal pain suggestive of AA	Pregnancy. Pain lasting >5 days.
Bachur 2016 (8)	Prospective cohort study; ED; USA	2133, 870 (41%)	3 to 18	* (52%)	Acute abdominal pain < 72 hours. Had a WCC count and WCC differential obtained.	Pregnancy. Prior abdominal surgery. Chronic gastrointestinal condition. Severe developmental delay.
Bal 2016 (9)	Prospective cohort study; PED; Turkey	96, 56 (58%)	4 to 18	58 (21%)	Acute abdominal pain < 96 hours. Surgical consultation for suspected AA. Documented follow up.	Pregnancy. Familial Mediterranean fever. Chronic abdominal illness. Sickle cell anaemia. Cystic fibrosis. Previous abdominal surgery. Immunosuppressive therapy.
Beltran, 2007 (10)	Prospective Cohort study; Hospital; ED; Chile	121, 71 (59%)	5 to 14	73 (60%)	Presenting with abdominal pain, nausea, vomiting and fever.	Re-presentation.
Benito, 2016 (11)	Prospective cohort study; PED; Spain	185, 89 (48%)	2 to 14	123 (66%)	Abdominal pain suggestive of AA as evaluated by attending physician.	Symptoms >5 days. Prior appendicectomy. Urinary tract infection. Cancer. Inflammatory disease. Corticoids
Calvo, 1998 (12)	Retrospective cohort study; Hospital; Spain	231, 141 (61%)	2 to 14	149 (65%)	Acute abdominal pain. Inpatient.	Less severe cases of abdominal pain.
Cayrol 2016 (13)	Prospective cohort study; PED; Spain	135, 52 (39%)	1 to 15	69 (51%)	Children with acute abdominal pain suggestive of AA.	Previous appendicectomy. Immunological, haemostatic or thrombotic disorder. Vasculitis. Cardiopathy. Cancer. Pregnancy.
Chiang, 2020 (14)	Retrospective cohort study; ED; Singapore	1391, 718 (52%)	2-16	814 (59%)	Admitted with suspected AA based on clinical history and physical examination.	Pregnancy. Previous appendicectomy. Inflammatory disease. Cancer.

Corkum, 2018 (15)	Retrospective cohort study; ED; USA	738, 156 (21%)	5 to 18	*	Child with suspected AA -defined by undergoing an abdominal US and had blood test.	*
Dadeh, 2021 (16)	Retrospective cohort study; ED; Thailand	1043, 97 (9%)	3 to 14	565 (54%)	Acute abdominal pain.	Intra-abdominal organ disease. Previous abdominal surgery. Cancer. Incomplete medical records.
Doraiswamy 1979 (17)	Case control study; Hospital; UK	375, 225 (60%)	5 to 15	*	Cases: children with AA. Controls: children with normal appendix on histology and children with acute abdominal pain.	Extra-abdominal complications.
Esparaz, 2019 (18)	Retrospective cohort study; ED; USA	327, 56 (17%)	2 to 17	*	Abdominal US for suspected AA	None
Fleischman, 2013 (19)	Prospective cohort study; PED; USA	178, 65 (37%)	3 to 18	84 (47%)	Patients with symptoms suggestive of AA, duration <7 days.	Negative appendectomy but other diagnosis indicated by imaging/surgery
Goldman, 2008(20)	Prospective cohort study; ED; Canada	849, 123 (36%)	1 to 17	*	Children with chief complaint of abdominal pain for <7 days.	Known diagnosis of AA (US or CT) on arrival to ED. Patients with prior appendicectomy.
Gonzalez del Castillo, 2016 (21)	Prospective cohort study; ED, 4 centres; Spain	321, 111 (35%)	2 to 20	167 (52%)	Abdominal pain <72 hours suggestive of AA	Ulcerative colitis. CT prior to consent. Pregnancy. Appendicectomy. Cancer. Trauma. Surgery within 7 days. Immunosuppressive treatment or chemotherapy.
Greer, 2019 (22)	Retrospective cohort study; ED; Australia	546, 86 (16%)	2 to 15	278 (51%)	Abdominal pain.	Trauma. Other confirmed diagnoses. Patients without blood test.
Groselj-Grenc 2007(23)	Nested case-control study; Hospital; Slovenia	82, 49 (60%)	3 to 14	53 (65%)	Cases: Children who underwent surgery for AA. Controls: Diagnosis of non-specific abdominal pain or sonographic mesenteric lymphadenitis.	Clinically or microbiologically established infections.
Hsiao 2005(24)	Case-control; Hospital; Taiwan	222, 111 (50%)	0 to 14	* (ratio 1.52 M:1 F)	Cases: Diagnosis of AA on histology. Controls: Suspected AA in same period.	None
Huckins 2013(25)	Prospective cohort study; ED; 12 centres; USA	503, 144 (29%)	2 to 20	243 (48%)	Right lower quadrant/ generalized abdominal pain with other signs/ symptoms consistent with AA of duration < 72 hours.	Previous appendicectomy. Cancer. Bleeding disorder. Trauma. Invasive abdominal procedures or prior diagnostic imaging during this illness. Participation in other research protocols within 2 weeks. Unable to obtain consent.
Huckins 2016(26)	Prospective cohort study; ED; 29 centres; USA	1887, 477 (25%)	2 to 20	848 (45%)	Right lower quadrant/generalized abdominal pain <72 hours with other signs/symptoms suggesting AA. Informed consent.	Previous appendicectomy. Cancer. Bleeding disorder. Autoimmune disorder. Trauma. Immunosuppressive medications. Invasive abdominal procedure within 2 weeks. Surgery without imaging. Prisoners. Participation in other research protocol within 30 days.
Kaiser, 2018(27)	Case control study; Hospital; Austria	64, 39(61%)	*(mean age 12 yr)	24 (38%)	Cases: children who underwent appendectomy. Controls: abdominal pain and improved under conservative treatment	*
Kashtan, 2020 (28)	Retrospective cohort study; ED; USA	2277, 974 (43%)	3 to 18	1098 (48%)	Abdominal US and surgical consultation for possible AA.	Missing data. Prior abdominal surgery. Immunodeficiency.
Khan 2012 (29)	Prospective cohort study; PED; USA	50, 22 (44%)	5 to 17	22 (44%)	Right lower quadrant abdominal pain without signs of gastroenteritis.	Chronic haematological, immunological or gastrointestinal disease. Pregnancy.
Khanafer, 2016(30)	Prospective cohort study; paediatric ED; Canada	180, 55(31%)	5 to 17	78 (43%)	Abdominal pain <5 days. AA in differential from ED with bloods done.	Previous appendicectomy. Positive imaging prior to presentation. Pregnancy. Immuno-suppressive disorders.
Kharbanda 2005(31)	Prospective cohort study; PED; USA	601, 211 (37%)	3 to 18	307 (51%)	Surgical consultation for possible AA	Pregnant. Previous abdominal surgery. Chronic medical condition. Radiological study of abdomen within 2 weeks.
Kharbanda, 2012 (32)	Prospective cross-sectional study; PED; USA	176, 58 (33%)	3 to 18	92 (52%)	Acute abdominal pain <96 hours duration evaluated for AA as defined by clinician.	Pregnancy. Prior abdominal surgery, chronic GI illness. Inaccurate history. Imaging / abdominal trauma in <7 days.
Kharbanda, 2012 (33)	Prospective cross-sectional study; PED; 10 centres; USA	2625, 1018 (39%)	3 to 18	1339 (51%)	Abdominal pain <96 hours duration in keeping with AA (as defined by clinician).	Pregnancy. Prior abdominal surgery. Chronic abdominal illness. Sickle cell. Cystic fibrosis. Radiological investigation diagnosing AA before arrival. Trauma within 7 days

Fawkner-Corbett D, *et al. BMJ Open* 2022; 12:e056854. doi: 10.1136/bmjopen-2021-056854

Klein, 2021 (34)	Prospective cohort study; PED; Israel	134, 53 (40%)	3 to 17	71 (53%)	Admitted with suspected AA	Previous appendectomy. Cancer. Haematological disorders. Active auto-inflammatory disorders. Trauma. Abdominal surgery. Previously known infectious disease such as urinary tract infection or pneumonia. Previously known urological/gynecological disorders such as pelvic inflammatory disease.
Ko, 1995 (35)	Prospective cohort study; Hospital; Taiwan	47, 27 (57%)	2 to 14	29 (62%)	Patients with clinically suspected AA	Atypical clinical signs. Didn't complete protocol.
Kouame, 2005 (36)	Prospective diagnostic accuracy study; Hospital; France	101, 68 (67%)	2 to 15	52 (51%)	Hospitalised children with abdominal pain that was suspected to require surgery.	Antibiotics prior to hospital presentation.
Kumar, 2021 (37)	Prospective observational study; tertiary PED; India	120, 55 (46%)	5 to 12	Unknown	Acute right lower abdomen pain.	Duration of symptoms <6 h or >72 h. Haematological disorders. Oncological disorders. Hepatic dysfunction. Infectious, or inflammatory conditions. Anticoagulants, anti-inflammatory medications or antibiotics.
Kwan, 2010(38)	Prospective observational study; PED; USA	209, 115 (55%)	1 to 18	123 (59%)	Abdominal pain suspicious of AA.	Pregnancy. Bleeding disorder. Severe anaemia. Chronic disease. Abdominal surgery in the previous year.
Lin, 2009(39)	Retrospective cohort study; ED; Taiwan	100, 53 (53%)	2 to 17	46(46%)	Children with RLQ pain presenting to ED	Patients who went home against medical advice.
Lycopoulou, 2005(40)	Prospective observational with nested case-control study; PED; Greece	60, 42 (70%)	5 to 14	30 (50%)	A nested control of children operated for AA; Controls: children presenting to PED during same period with symptoms and signs of AA	No consent. Retrocaecal abscess. Normal appendix histology.
Malia, 2019(41)	Prospective cohort study; Paediatric hospital ED; USA	762, 225 (30%)	0 to 18	356 (47%)	Children with suspected AA who underwent US	Patient absconded, previous appendicectomy, appendix not visualised on US, US at other facility.
Mandeville, 2011(42)	Prospective observational study; PED; USA	287, 155 (54%)	4 to 17	151 (53%)	Abdominal pain suggestive of AA	Pregnancy, previous abdominal surgery, chronic medical condition, non-verbal, radiological investigation of abdomen in preceding 2 weeks, no follow up.
Miguez, 2016(43)	Prospective cohort study; Paediatric hospital ED; Spain	136, 44 (32%)	3 to 16	76 (56%)	Admitted to ED with abdominal pain <96 hours AA suspected at initial evaluation.	Appendicectomy /recent surgery. Immune disease. Chronic respiratory or cardiovascular disease. Inflammatory bowel disease. Antibiotics or steroids in the last month.
Mohammed, 2004(44)	Prospective cohort study; Hospital paediatric surgical department; Libya	216, 130 (60%)	3 to 13	139(64%)	Child presenting with clinical suspicion of AA	*
Moreno, 2012(45)	Retrospective Cohort study; Hospital; Spain	184, 85 (46%)	* (Mean age 8.9 yr)	110 (60%)	Acute abdominal pain suggestive of AA.	*
Naqvi, 2019(46)	Observational cohort study; Tertiary Paediatric Hospital; Canada	185, 79 (43%)	4 to 17	86 (46%)	Children with suspected AA demonstrated by US or referral to paediatric surgical team. Also children with sepsis defined by SIRS criteria or PICU admission (AA sub-group reported)	Previous appendicectomy, resuscitation in ED, discharge to PICU, pregnancy, AP >5 days, immune-suppression, abdominal imaging prior to admission, life expectancy <24 hours.
Oikonomopoulou, 2019(47)	Prospective cohort study; PEDs, 6 centres; Spain	285, 100 (35%)	0 to 18	167 (59%)	Clinical suspicion of AA after physician assessment	Pain >72hr, lack of blood sample, history of surgery (within 3 months)/ immune pathology/ inflammatory bowel disease/ cardio-respiratory disease, recent steroid or antibiotic use

Fawkner-Corbett D, *et al. BMJ Open* 2022; 12:e056854. doi: 10.1136/bmjopen-2021-056854

Ovrebo, 1993(48)	Retrospective case series; Hospital surgical department; Norway	470, 161 (34%)	0 to 15	235 (50%)	All patients presenting with abdominal pain <1 week duration suggestive of AA.	Abdominal trauma. Incarcerated herniae.
Ozguner, 2014(49)	Prospective cohort study; Children's hospital; Turkey	49, 34(69%)	3 to 17	34 (69%)	Children hospitalised with right lower-quadrant pain suspicious of AA between 0400 and 1500.	If sample for CD64 measurement could not be obtained.
Peltola, 1986(50)	Prospective cohort study; Children's hospital; Finland	162, 94 (58%)	1 to 16	77 (48%)	Children presenting with abdominal pain or symptoms suggestive of AA	Symptoms suggestive or respiratory infection.
Prada-Arias, 2017(51)	Prospective cohort study; Tertiary hospital PED; Spain	275, 132 (48%)	5 to 15	153 (56%)	Clinical suspicion of AA with blood tests and US performed	Symptom duration <6hr or >72hr. Haematological / oncological / hepatic / infectious or inflammatory disease present. >12 hours between blood test and surgery
Rodríguez Sánchez de la Blanca, 2014(52)	Prospective cohort study, PED, Spain	105, 41 (39%)	<16	59 (56%)	Abdominal pain or clinical suspicion of AA	Antibiotic treatment in the preceding 10 days, known inflammatory pathology
Salo, 2016(53)	Case control study; PED; Sweden	44, 22 (50%)	3 to 14	27 (61%)	Referral to paediatric surgeon with suspicion of AA	Immuno-modulating therapy or clear-cut diagnosis of AA
Samuel, 2002(54)	Prospective cohort study; Hospital, 2 centres; UK	1170, 734 (63%)	4 to 15	752(64%)	Abdominal pain suggestive of AA	Appendicula mass with peri-appendiceal abscess
Sanchez, 1998(55)	Prospective cohort study; Paediatric hospital ED; Spain	195, 94 (48%)	2 to 14	107(55%)	Children with suspected AA	*
Shommu, 2018(56)	Prospective cohort study; PED; Canada	140, 52 (36%)	5 to 17	61 (44%)	Clinically suspected AA or US evaluation for AA with IV placed and did not require intensive care treatment from PED. Informed consent	Previous appendectomy. Pregnancy. Abdominal pain >5 days. Immuno-suppression. Previously enrolled in the study. Imaging study performed elsewhere
Thompson, 2015(57)	Retrospective cohort study; ED, 12 centres; Canada	619, 510 (83%)	3 to 17	352 (53%)	Children admitted with suspected AA	Intensive care unit admission.
Thompson, 2016 (58)	Retrospective cohort study; Children's hospital ED; Canada	1315, 561[data provided n=1138,471 children] (41%)	2 to17	565 (43%)	Children investigated for suspected AA with blood culture sample collected	*
Tuncer, 2019(59)	Retrospective cross-sectional study; Paediatric Surgical/Medical department; Turkey	301; 137 (46%)	*(Mean age 11yr)	139 (46%)	Children with acute abdominal pain, recorded diagnosis of acute appendicitis, mesenteric lymphadenitis or familial Mediterranean fever.	Heart failure, peripheral vascular / haematological /liver disease, anticoagulation, steroid use, concurrent acute or chronic infection
Turkyilmaz, 2006(60)	Prospective cohort study; Hospital; Turkey	105, 61 (58%)	3 to 16	68 (65%)	Admission with acute right-lower abdominal pain	*
Van Den Broek, 2004(61)	Prospective cohort study; ED, 2 centres; Netherlands	275, 169 (66%)	*(Mean age 9 yr)	148 (58%)	GP referral with suspicion of AA	*
Wang 2007(62)	Prospective and retrospective cohort study; PED; USA	722, 74 (18%)	1 to 19	334 (46%)	Prospective: ED presentation with suspected AA. Retrospective: AA on surgery, radiology, and laboratory records.	History of trauma
Wu, 2012(63)	Prospective cohort study; ED; Taiwan	594, 306 (52%)	4 to 18	358 (60%)	Children with clinically suspected AA, as determined by emergency physician.	Symptoms >3 day. Patients lost to follow up.
Yap, 2015(64)	Prospective cohort study; Paediatric hospital ED; Singapore	747, 229 (32%)	4 to 16	429(57%)	Admitted to ED with suspected AA.	Previous appendicectomy, pregnancy, chronic medical or malignant conditions. CT in the previous month.

Fawkner-Corbett D, *et al. BMJ Open* 2022; 12:e056854. doi: 10.1136/bmjopen-2021-056854

Yazici, 2010(65)	Retrospective case series; Paediatric surgical department; Turkey	240, 183 (76%)	3 to 16	*	Patients with acute abdominal pain	Urinary tract infection, Henoch-Schonlein pupura or gastroenteritis.
Zouari, 2016(66)	Prospective cohort study; Paediatric surgical department; Tunisia	292, 132 (45%)	3 to 14	170(58%)	Diagnosis of suspected AA by consultant paediatric surgeon on the basis of clinical history and physical exam	Patients who had not undergone US
Zuniga, 2012(67)	Prospective cohort study; Hospital ED; Spain	101, 28 (28%)	0 to 14	55 (54%)	Presenting with clinical suspicion of AA	Pain >7 days. Previous appendicectomy. No blood tests.

Fawkner-Corbett D, *et al. BMJ Open* 2022; 12:e056854. doi: 10.1136/bmjopen-2021-056854

Supplementary Table 3: Overview of included studies Index tests, cut off values and reference standards. (CRP = C-reactive protein. WCC = White Cell Count. PMN = Polymorphonuclear cells. * = non-AA not defined/not clearly defined, †=multiple cut offs or range in paper, ‡=author provided raw data)

Study	Reference Standard	Index Test(s) measured	Index test cut off value(s)
Akgül, 2021 (1)	Histopathology or 14 days follow up	• WCC<br>• Neutrophil count<br>• CRP<br>• Procalcitonin	10,000 µL<br>7500 µL<br>5 mg/L<br>0.5 ng/mL
Altali, 2017 (2)	Histopathology or discharge diagnosis	• WCC<br>• Neutrophil count<br>• CRP	>10,500/mm^3<br>>6,600/mm^3<br>>5mg/L
Anandalwar, 2015 (3)	Pathology proven appendicitis *	• WCC<br>• Neutrophil %	>9,000/mm^3<br>>65%
Andersson, 1999 (4)	Operation note and histopathology or failure to re-present	• WCC<br>• Neutrophil count<br>• Neutrophil %<br>• CRP<br>WCC/CRP as separate and combined	‡
Andersson, 2008 (5)	Histopathology or review of case files in the following 1 month.	• WCC<br>• Neutrophil count<br>• Neutrophil %<br>• CRP<br>WCC/CRP as separate and combined	‡
Andersson, 2014 (6)	Histopathology or 6 month follow up	• WCC<br>• Neutrophil count<br>• Neutrophil %<br>• CRP<br>WCC/CRP as separate and combined	‡
Andersson, 2017 (7)	Histopathology or 30 day follow up	• WCC<br>• Neutrophil count<br>• Neutrophil %<br>• CRP<br>WCC/ CRP as separate and combined	‡
Bachur 2016 (8)	Operative diagnosis, telephone follow-up (1 -2 weeks) or record review (90 days) after visit.	• WCC<br>• Neutrophil count	5-15,000/mm^3†<br>5-15,000/mm^3†
Bal 2016 (9)	Surgery and pathology report or follow-up by phone interview 7-14 days after visit	• WCC<br>• Neutrophil count<br>• CRP<br>• Procalcitonin	>13,400/mm^3<br>>9,400/mm^3<br>>0·9mg/L<br>0·37ng/mL
Beltran, 2007 (10)	Histopathological report or discharge diagnosis	• WCC<br>• CRP	>12,500/mm^3<br>>47mg/L

Fawkner-Corbett D, *et al. BMJ Open* 2022; 12:e056854. doi: 10.1136/bmjopen-2021-056854

Benito, 2016 (11)	Histopathological analysis of surgical specimen, telephone follow up 15 days after consultation, or check of electronic registry if not contactable.	• WCC<br>• Neutrophil count<br>• CRP<br>• Procalcitonin	<10,000/mm^3<br><7,500/mm^3<br><20 mg/L<br><0·1ng/ml
Calvo, 1998 (12)	Histopathological examination or discharge diagnosis	• WCC<br>• CRP<br>Tests reported as separate and as combination	>15,000/mm^3<br>>20mg/l
Cayrol 2016 (13)	Surgery and histopathology or clinical decision with telephone follow up 5 days later	• WCC<br>• CRP	>10,000/mm^3<br>>30mg/L
Chiang, 2020 (14)	Histopathology or 24 hours of clinical observation with or without radiologic imaging and with 3 days follow up after discharge.	• WCC<br>• Neutrophil %<br>• CRP	≥10,000/µL<br>≥75%<br>≥5 mg/L
Corkum, 2018 (15)	Histopathology and operative note or 30 day follow up	• Neutrophil count	>8,000/mm^3
Dadeh, 2021 (16)	Histopathology *	• WCC<br>• Neutrophil %	>10,000 or >14,000<br>>75%<br>(based on duration of pain less or more than 24 hours)
Doraiswamy 1979 (17)	Appendicitis confirmed or refuted at surgery or recovered without surgery	• WCC<br>• Neutrophil %	>15,000/mm^3 or >13,000/mm^3 † >50%, >65% or >75%†<br>(age dependent)
Esparaz, 2019 (18)	Histopathology *	• WCC	>10,000/mm^3
Fleischman, 2013 (19)	Surgical pathology, positive CT scan finding or diagnosis and follow up at 2 weeks	• WCC<br>• Neutrophil count<br>• Neutrophil %	>10,000/mm^3<br>>6750/mm^3<br>>75%
Goldman, 2008(20)	Appendicectomy pathology or follow-up 5 to 7 days after ED discharge	• WCC<br>• Neutrophil count	>10,000/mm^3<br>>7,500/mm^3
Gonzalez del Castillo, 2016 (21)	Histopathology reported by blinded pathologist or telephone follow up 2 weeks after discharge	• WCC<br>• CRP	>12,000/mm^3<br>>5mg/L
Greer, 2019 (22)	Histopathology or discharge diagnosis	• WCC<br>• Neutrophil count<br>• CRP	>13.5×10^9/L<br>>7.5×10^9/L<br>≥5 mg/L
Groselj-Grenc 2007(23)	Appendicitis diagnosed by surgery or ultrasound, or diagnosis of non-specific abdominal pain/sonographic mesenteric adenitis	• WCC<br>• CRP	>11,600/mm^3<br>>9.0mg/L
Hsiao 2005(24)	Surgery or pathology report, or appendicitis excluded in final diagnosis	• WCC<br>• Neutrophil %	>10,400/mm^3<br>>75%
Huckins 2013(25)	Surgical pathology report after appendectomy or discharge diagnosis	• WCC<br>• CRP<br>Tests only reported in combination	>10,000/mm^3<br>>8mg/L
Huckins 2016(26)	Surgical pathology reports after appendectomy or follow-up telephone calls at 14 days (±3 days)	• WCC<br>• CRP<br>Tests only reported in combination	>10,000/mm^3<br>>8mg/L
Kaiser, 2018(27)	Histopathology or discharge diagnosis	• WCC<br>• CRP	>10 -15,000/mm^3 †<br>5-50mg/L†

Fawkner-Corbett D, *et al. BMJ Open* 2022; 12:e056854. doi: 10.1136/bmjopen-2021-056854

		Tests as separate and combined	
Kashtan, 2020 (28)	Histopathology or 7 days follow up	• WCC	>9000 cells/uL
Khan 2012 (29)	Histopathological evidence or telephone follow up 24 hour and 2 weeks after discharge	• Procalcitonin	>0·39ng/ml
Khanafer, 2016(30)	Histopathology or follow up 2 weeks after discharge	• WCC	10-15,000m/^{3†}
Kharbanda 2005(31)	Pathology or follow-up telephone call 2 to 4 weeks after the ED visit	• WCC<br>• Neutrophil count	>8850/mm^3<br>6·75x10^3/ul
Kharbanda, 2012 (32)	Surgical histology or telephone follow up 14-21 days after admission/note review if uncontactable.	• WCC	>8850/mm^3
Kharbanda, 2012 (33)	Histology or telephone follow up 2 weeks after discharge or note review 90 days after discharge if unable to contact.	• Neutrophil count	>6750/mm^3
Klein, 2021 (34)	Histopathology or discharge diagnosis or clinical observation	• WCC<br>• Neutrophil %	> 12,000 or > 10,500<br>≥75%<br>(age dependent)
Ko, 1995 (35)	Surgical histology or diagnosis on discharge	• WCC<br>• Neutrophil %<br>• CRP	>10,000/mm^3<br>>75%<br>>9mg/L and >50mg/L
Kouame, 2005 (36)	Histology *	• Procalcitonin	>0.5µg/L
Kumar, 2021 (37)	Histopathology or 2 weeks follow up	• WCC<br>• Neutrophil count<br>• CRP	14,930/mm^3<br>10,485.5 cell/ul<br>10.5 mg/L
Kwan, 2010(38)	Operative reports and histology or telephone follow up or note review 2-6 weeks after discharge	• WCC<br>• CRP<br>Tests reported as separate and in combination	>12,000/mm^3<br>>30mg/L
Lin, 2009(39)	Appendectomy (histologic examination) or telephone follow-up within 1 week of consultation	• WCC<br>• CRP	>15,000/mm^3<br>>1 mg/dL
Lycopoulou, 2005(40)	Appendicectomy (histological examination) or 1 week follow up	• WCC<br>• CRP	>10,000/mm^{3†}<br>>10mg/L
Malia, 2019(41)	Histopathology report or failure to re-attend within 1 week	• WCC<br>• CRP	>10,000/mm^3<br>>5mg/L
Mandeville, 2011(42)	Histopathology or telephone follow up at 2 weeks	• WCC<br>• Neutrophil %	>10,000/mm^3<br>>75%
Miguez, 2016(43)	Histopathology of appendicectomy specimen or telephone follow up 5-7 days afterwards.	• WCC<br>• Neutrophil count<br>• CRP	>12,300/mm^3<br>>9,600/mm^3<br>>3 mg/L
Mohammed, 2004(44)	Appendicectomy (histopathological results) or clinical observation	• WCC<br>• Neutrophil %<br>• CRP<br>Tests reported as separate and in combination (all three)	>11,000/mm^3<br>>75%<br>>8mg/L
Moreno, 2012(45)	Surgery, histology, clinical observation or follow up	• WCC<br>• CRP	>12,800/mm^3<br>>18mg/L

Fawkner-Corbett D, *et al. BMJ Open* 2022; 12:e056854. doi: 10.1136/bmjopen-2021-056854

Naqvi, 2019(46)	Histopathology, insertion of percutaneous drain or failure to re-attend within 2 weeks	• CRP	>7·9mg/L
Oikonomopoulou, 2019(47)	Histopathology and surgeons notes, telephone follow up after 5-7 days or checked for readmission if no response	• WCC<br>• Neutrophil count<br>• CRP<br>Tests reported as separate and in paired combinations	>13,300/mm^3<br>>10,800/mm^3<br>>12·5mg/L
Ovrebo, 1993(48)	Operative note (all) and histology (some) or discharge diagnosis	• WCC<br>• CRP<br>Tests reported as separate and in combination	>11,000/mm^3<br>>10mg/L
Ozguner, 2014(49)	Operative findings, histopathological examination and discharge diagnosis	• WCC<br>• CRP	>12,000/mm^3<br>>16mg/L
Peltola, 1986(50)	Histology on those operated or diagnosis of non-specific abdominal pain	• WCC<br>• CRP<br>Tests reported as separate and in combination	>14,900/mm^3<br>>10mg/L
Prada-Arias, 2017(51)	Histology and surgical report or no specific disease identified, no findings on US and no antibiotic treatment	• WCC<br>• Neutrophil count<br>• CRP	>11,000/mm^3<br>>7,000/mm^3<br>>25·2mg/L
Rodríguez Sánchez de la Blanca, 2014(52)	Histopathology*	• WCC<br>• CRP<br>• Procalcitonin	>13,000/mm^3<br>>5mg/L<br>>0·5µg/L
Salo, 2016(53)	Surgical note and histopathology report or follow up	• WCC<br>• Neutrophil count<br>• Neutrophil %<br>• CRP<br>Combined WCC and neutrophil count reported	>13-16,000/mm^{3†}<br>>6·5-7,000/mm^{3†}<br>>71%<br>>15mg/L<br>(ranges age dependent)
Samuel, 2002(54)	Histology in surgical group*	• WCC<br>• Neutrophil count	>10,00/mm^3<br>>7,500/mm^3
Sanchez, 1998(55)	Histology and surgical report or discharge diagnosis	• WCC<br>• CRP	>14000/mm^3<br>>30mg/L
Shommu, 2018(56)	Histopathology*	• WCC<br>• Neutrophil count<br>• CRP	‡ (Provided 2x2 tables for multiple cut offs of tests but not IPD)
Thompson, 2015(57)	Histopathology or period of observation	• WCC<br>• Neutrophil count<br>• CRP	‡
Thompson, 2016 (58)	Histopathology or discharge diagnosis	• WCC<br>• Neutrophil count<br>• CRP	‡
Tuncer, 2019(59)	Histopathology or discharge diagnosis	• WCC	>13,050/mm^3
Turkyilmaz, 2006(60)	Histopathology and operative assessment or clinical observation	• WCC	>15000/mm^3 or 13,000/mm^{3†} (age dependent)
Van Den Broek, 2004(61)	Histology*	• WCC	>10,000/mm^3
Wang 2007(62)	Histology or review of medical records	• WCC	†(Age dependent cut-off)

Fawkner-Corbett D, *et al. BMJ Open* 2022; 12:e056854. doi: 10.1136/bmjopen-2021-056854

		• Neutrophil %<br>Tests reported as separate and in combination	>80%
Wu, 2012(63)	Surgery, histological examination or follow up interview two weeks after visit	• WCC<br>• CRP	>11000/mm^3<br>>25 and 89mg/L
Yap, 2015(64)	Histology, operative findings or observation on the ward until >24 hours pain free	• WCC<br>• Neutrophil %<br>• CRP<br>Tests reported as separate and in combination (all three)	> 10,000/mm^3<br>>75%<br>>5mg/L
Yazici, 2010(65)	Histopathology or observation for 24-48 hours	• WCC	>10,000/mm^3
Zouari, 2016(66)	Histology showing acute appendicitis or failure to represent for 2 weeks	• CRP	6mg/L
Zuniga, 2012(67)	Histopathology or failure to re-present for 7 days	• WCC<br>• Neutrophil count	>10,000/mm^3<br>>7,500/mm^3

Fawkner-Corbett D, *et al. BMJ Open* 2022; 12:e056854. doi: 10.1136/bmjopen-2021-056854

Supplementary Table 4: Individual assessment scoring of each included study assessed by means of the QUADAS-2 tool for risk of bias.

Study	RISK OF BIAS				APPLICABILITY CONCERNS		
	PATIENT SELECTION	INDEX TEST	REFERENCE STANDARD	FLOW AND TIMING	PATIENT SELECTION	INDEX TEST	REFERENCE STANDARD
Akgül, 2021	High	High	Low	High	Low	Low	Low
Altali, 2017	Low	Unclear	Low	Unclear	Low	High	Low
Anandalwar, 2015	High	High	Unclear	Unclear	Low	High	Unclear
Andersson, 1999	Unclear	High	High	High	Unclear	Unclear	High
Andersson, 2014	Low	Unclear	Unclear	High	Low	Unclear	Low
Andersson, 2017	Low	Unclear	Unclear	High	Low	Low	Low
Andersson,2008	Low	Unclear	Unclear	High	Low	Unclear	Low
Bachur, 2016	Low	Unclear	Unclear	High	Low	Unclear	Unclear
Bal, 2016	High	Unclear	Unclear	High	Low	Low	Low
Beltran, 2007	Low	Low	High	Unclear	Low	Low	Unclear
Benito, 2016	High	Low	High	High	Low	Low	Low
Calvo Rigual, 1998	Low	Unclear	High	High	Low	Low	Low
Cayrol, 2016	High	Low	High	High	Low	Low	High
Chiang, 2020	Low	Unclear	High	High	Low	Low	High
Corkum, 2018	High	Unclear	Unclear	High	Low	Unclear	Low
Dadeh, 2021	Unclear	Unclear	Unclear	Unclear	Low	Unclear	High
Diaz Moreno, 2012	Low	High	Unclear	Unclear	Low	Unclear	Unclear
Doraiswamy, 1979	High	Unclear	Unclear	High	Low	Low	Unclear
Esparaz, 2019	High	Unclear	High	High	Low	Low	Low
Fleischman, 2013	High	Unclear	Unclear	High	Low	Low	Low
Goldman, 2008	Low	Unclear	High	High	Low	Low	High
Gonzalez, 2016	Low	High	High	High	Low	High	Low
Greer, 2019	High	Unclear	High	High	Low	Low	High
Groselj-Grenc, 2007	Low	High	Unclear	High	Low	Low	High
Hsiao, 2005	High	Unclear	Unclear	High	Low	Low	Unclear
Huckins, 2013	High	Low	High	High	High	Low	High
Huckins, 2016	High	Low	Low	High	High	Low	Low
Kaiser, 2018	High	Unclear	High	High	Low	Unclear	High
Kashtan, 2020	Unclear	Unclear	High	High	Low	Low	Low
Khan, 2012	Low	High	Unclear	Unclear	Low	High	Low
Khanafer, 2016	High	Unclear	Unclear	High	Low	Low	Unclear
Kharbanda, 2005	High	Unclear	Unclear	High	Low	Low	Low
Kharbanda, 2012	Low	High	High	High	Low	Low	Low
Kharbanda, 2012	High	Unclear	Unclear	High	Low	Low	Low
Klein, 2021	High	Unclear	High	High	Low	Low	High
Ko, 1995	Low	Unclear	Unclear	High	Low	Low	Unclear
Kouame, 2005	Low	Unclear	Unclear	Unclear	Low	Low	Unclear
Kumar, 2021	High	Unclear	Low	High	Low	Low	Low
Kwan, 2010	Low	High	Unclear	High	Low	Low	Low
Lin, 2009	Low	Unclear	High	High	Low	High	High
Lycopoulou, 2005	High	Unclear	High	High	Low	Low	High

Malia, 2019	High	High	High	High	High	Unclear	Low
Mandeville, 2011	High	Unclear	Low	High	Low	Low	Low
Miguez, 2015	Low	High	High	High	Low	High	High
Mohammed, 2004	Low	Unclear	Unclear	High	Low	Low	Unclear
Naqvi, 2019	High	High	Unclear	High	Unclear	High	Low
Oikonomopoulou, 2019	Low	High	High	High	Low	High	Low
Ovrebo, 1993	Unclear	High	High	High	Low	Low	High
Ozguner, 2014	High	High	High	High	Low	High	High
Peltola, 1986	Low	Unclear	Unclear	High	Low	Low	Unclear
Prada-Arias, 2017	Low	Unclear	Unclear	High	Low	Low	Low
Rodriguez, 2014	High	Unclear	Low	High	Low	Low	Low
Salo, 2016	High	Unclear	Unclear	High	Low	Low	Unclear
Samuel, 2002	Low	Unclear	Unclear	High	Low	Low	Unclear
Sanchez, 1998	Low	High	Unclear	High	Unclear	Unclear	Unclear
Shommu, 2018	High	High	High	High	High	Unclear	High
Thompson, 2015	Low	Unclear	High	High	Low	Unclear	High
Thompson, 2017	Unclear	Unclear	High	High	Low	Unclear	High
Tuncer, 2019	High	High	High	High	Low	Unclear	Unclear
Turkyilmaz, 2006	Low	Unclear	Unclear	High	Low	Low	Unclear
Van Den Broek, 2004	Unclear	Unclear	High	High	Low	Low	High
Wang, 2007	Low	Unclear	Unclear	High	Low	Low	Unclear
Wu, 2012	Unclear	High	Unclear	High	Low	High	Low
Yap, 2015	Low	Unclear	Unclear	High	Low	Low	Unclear
Yazici, 2010	High	Unclear	High	High	Low	Unclear	High
Zouari, 2016	Low	Unclear	Unclear	High	Low	Unclear	Low
Zuniga, 2012	High	Unclear	High	High	Low	Low	High

Supplementary Table 5: Pooled estimates of diagnostic accuracy for blood tests used for diagnosis of appendicitis in children calculated using the bivariate model and reporting likelihood ratios(*Median prevalence of appendicitis in the included 54 cohort studies is 40%. Sensitivity analysis was performed for all tests at all cut offs, except procalcitonin as number of studies was too low, and identified significant (p<0.05) effects in: †WCC 10,000-15,000cells/ul for case-control study design (p=0·01, sensitivity 0·70[0·57-0·84] and specificity 0·49[0·31-0·66] n=3 case-control studies vs sensitivity 0·76[0·72-0·80] and specificity 0·72[0·68-0·76] n=27 non case-control studies))

Marker	Threshold	Studies	Number of patients	Pooled sensitivity (95% CI)	Pooled specificity (95% CI)	Pooled LR+ (95% CI)	Pooled LR- (95% CI)	Hypothetical cohort of 1000 children with appendicitis prevalence of 40%*	
								Missed appendicitis (FN)	Unnecessary treatment (FP)
CRP (mg/L)	≤5	14	3754	0·82 (0·72 – 0·89)	0·46 (0·28 – 0·66)	1·52 (1·14 – 2·03)	0·39 (0·30 – 0·51)	112	324
	5-10	16	3170	0·73 (0·67 – 0·80)	0·67 (0·58 – 0·75)	2·21 (1·75 – 2·79)	0·40 (0·33 – 0·50)	108	198
	10-50	19	4470	0·53 (0·45 – 0·62)	0·79 (0·74 – 0·83)	2·53 (1·99 – 3·21)	0·59 (0·49 – 0·71)	188	126
	≥50	10	2654	0·35 (0·25 – 0·46)	0·89 (0·87 – 0·91)	3·25 (2·63 – 4·01)	0·73 (0·63 – 0·84)	260	66
WCC (Cells/µl)	≤10,000	28	11791	0·87 (0·83 – 0·89)	0·58 (0·53 – 0·63)	2·08 (1·87 – 2·31)	0·23 (0·19 – 0·27)	52	252
	10,000 - 15,000†	30	9967	0·76 (0·72 – 0·79)	0·70 (0·65 – 0·75)	2·56 (2·19 – 3·00)	0·35 (0·30 – 0·40)	96	180
	≥15,000	14	6406	0·50 (0·44 – 0·55)	0·84 (0·80 – 0·87)	3·12 (2·74 – 3·55)	0·60 (0·55 – 0·66)	200	96
Neutrophils (% of lymphocytes)	65% - 80%	13	3316	0·81 (0·74 – 0·86)	0·64 (0·55 – 0·72)	2·23 (1·76 – 2·82)	0·30 (0·22 – 0·41)	76	216
	75%	9	1642	0·78 (0·73-0·82)	0·61 (0·53-0·69)	2·02 (1·61-2·53)	0·36 (0·28-0·46)	88	234
ANC (Cells/µl)	6600 - 10000	22	11966	0·85 (0·81 – 0·88)	0·63 (0·58 – 0·68)	2·29 (2·06 – 2·56)	0·23 (0·19 – 0·28)	60	222
	<7500	5	3834	0·90 (0·85 – 0·94)	0·54 (0·49 – 0·58)	1·95 (1·79 – 2·13)	0·18 (0·12 – 0·27)	40	276
	7500	11	4730	0·86 (0·82 – 0·90)	0·60 (0·54 – 0·65)	2·14 (1·90 – 2·42)	0·23 (0·17 – 0·30)	56	240
	>7500	5	3388	0·73 (0·70 – 0·77)	0·77 (0·70 – 0·82)	3·13 (2·35 – 4·16)	0·35 (0·29 – 0·41)	108	138
Procalcitonin (ng/ml)	0·1 – 0·5	5	540	0·31 (0·25 – 0·37)	0·82 (0·70 – 0·90)	1·72 (0·99 – 3·00)	0·84 (0·74 – 0·96)	276	108

Fawkner-Corbett D, *et al. BMJ Open* 2022; 12:e056854. doi: 10.1136/bmjopen-2021-056854

Supplementary Figures and legends:

Supplementary Figure 1: Funnel plots outputs for testing individual blood tests for publication bias(68). (Procalcitonin/ WCC and CRP/ WCC,ANC and CRP not shown as study numbers were low [n=4, p=0·2]. Testing was also performed for individual cut-offs of WCC and CRP with all results >0·1 [data not shown]. Y axis = inverse root of effective sample size. X axis = Odds ratio. 95% Confidence intervals for bias: CRP -6.10 – 15.03; WCC -5.26 – 7.15; Neutrophils % -13.72 – 17.74; ANC -4.18 – 11.87)

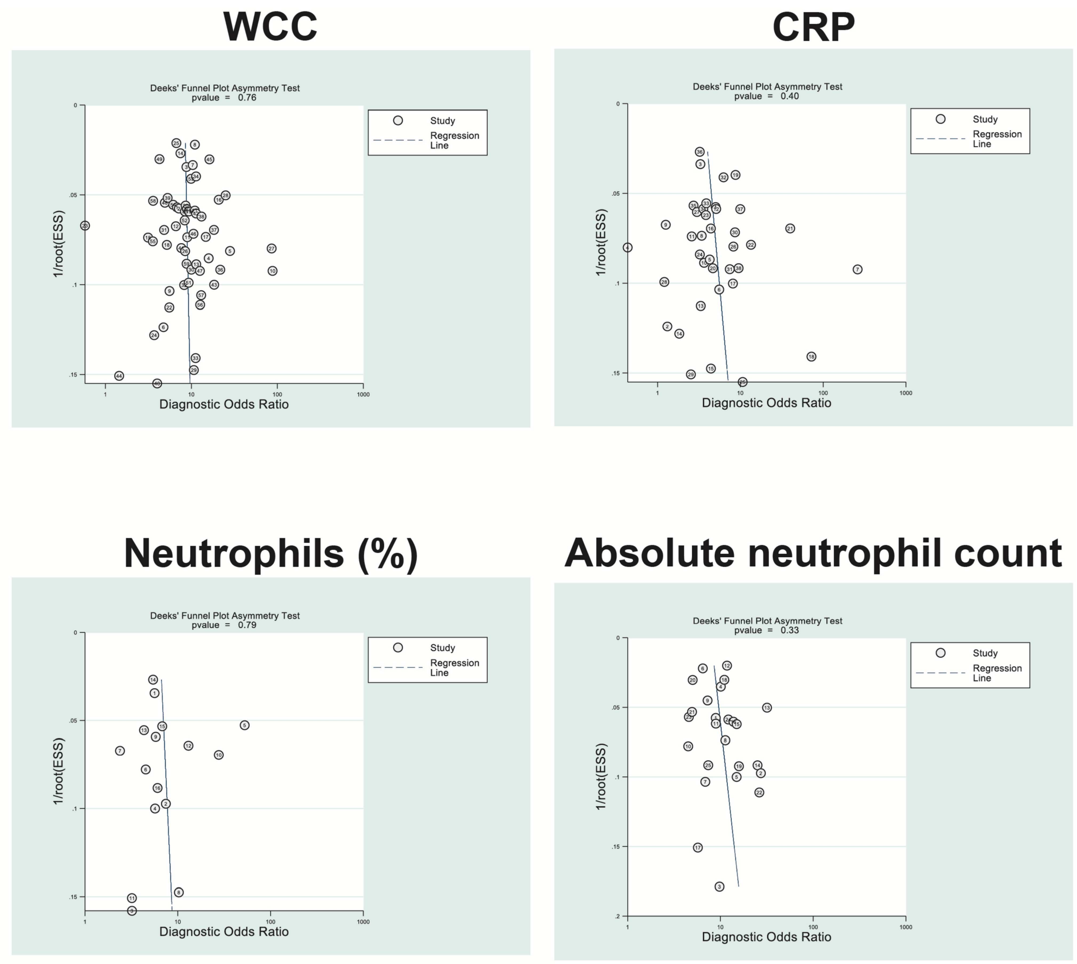

Fawkner-Corbett D, *et al. BMJ Open* 2022; 12:e056854. doi: 10.1136/bmjopen-2021-056854

Supplementary Figure 2: Forest plot showing all studies reporting diagnostic accuracy of WCC, split by reported cut off, in paediatric appendicitis. (Sensitivity and specificity for each study represented visually (blue square) with 95% confidence interval (line)).

WCC at or below 10,000 /mm^3

Study	TP	FP	FN	TN	Threshold	Sensitivity (95% CI)	Specificity (95% CI)
Kharbanda 2005	153	162	4	106	8.85	0.97 [0.94, 0.99]	0.40 [0.34, 0.46]
Kharbanda 2012	58	67	0	49	8.85	1.00 [0.94, 1.00]	0.42 [0.33, 0.52]
Anadalwar 2015	348	212	45	240	9.0	0.89 [0.85, 0.92]	0.53 [0.48, 0.58]
Kashtan 2020	857	681	117	622	9.0	0.88 [0.86, 0.90]	0.48 [0.45, 0.50]
Akgul 2021	173	78	17	52	10.0	0.91 [0.86, 0.95]	0.40 [0.32, 0.49]
Andersson 1999	42	37	6	84	10.0	0.88 [0.75, 0.95]	0.69 [0.60, 0.77]
Andersson 2008	17	35	4	39	10.0	0.81 [0.58, 0.95]	0.53 [0.41, 0.64]
Andersson 2014	52	61	2	66	10.0	0.96 [0.87, 1.00]	0.52 [0.43, 0.61]
Andersson 2017	303	339	30	348	10.0	0.91 [0.87, 0.94]	0.51 [0.47, 0.54]
Bachur 2016	786	581	84	682	10.0	0.90 [0.88, 0.92]	0.54 [0.51, 0.57]
Benito 2016	79	45	10	51	10.0	0.89 [0.80, 0.94]	0.53 [0.43, 0.63]
Cayrol 2016	46	33	6	49	10.0	0.88 [0.77, 0.96]	0.60 [0.48, 0.70]
Chiang 2020	625	319	93	354	10.0	0.87 [0.84, 0.89]	0.53 [0.49, 0.56]
Esparaz 2019	51	111	5	160	10.0	0.91 [0.80, 0.97]	0.59 [0.53, 0.65]
Fleischman 2013	53	52	12	61	10.0	0.82 [0.70, 0.90]	0.54 [0.44, 0.63]
Goldman 2008	98	84	14	112	10.0	0.88 [0.80, 0.93]	0.57 [0.50, 0.64]
Gonzalez Del Castillo 2016	93	18	79	131	10.0	0.54 [0.46, 0.62]	0.88 [0.82, 0.93]
Kaiser 2018	29	11	10	14	10.0	0.74 [0.58, 0.87]	0.56 [0.35, 0.76]
Khanafer 2016	46	51	8	75	10.0	0.85 [0.73, 0.93]	0.60 [0.50, 0.68]
Ko 1995	23	7	4	13	10.0	0.85 [0.66, 0.96]	0.65 [0.41, 0.85]
Lycopoulou 2005	32	4	10	14	10.0	0.76 [0.61, 0.88]	0.78 [0.52, 0.94]
Malia 2019	169	113	56	424	10.0	0.75 [0.69, 0.81]	0.79 [0.75, 0.82]
Mandeville 2011	129	49	26	83	10.0	0.83 [0.76, 0.89]	0.63 [0.54, 0.71]
Ozgunur 2014	32	12	2	3	10.0	0.94 [0.80, 0.99]	0.20 [0.04, 0.48]
Samuel 2002	624	113	110	323	10.0	0.85 [0.82, 0.88]	0.74 [0.70, 0.78]
Shommu 2018	41	32	5	49	10.0	0.89 [0.76, 0.96]	0.60 [0.49, 0.71]
Thompson 2015	425	56	71	46	10.0	0.86 [0.82, 0.89]	0.45 [0.35, 0.55]
Thompson 2017	334	243	137	424	10.0	0.71 [0.67, 0.75]	0.64 [0.60, 0.67]
van den Broek 2004	52	8	13	26	10.0	0.80 [0.68, 0.89]	0.76 [0.59, 0.89]
Yap 2015	102	126	17	129	10.0	0.86 [0.78, 0.91]	0.51 [0.44, 0.57]
Yazici 2010	162	39	21	18	10.0	0.89 [0.83, 0.93]	0.32 [0.20, 0.45]
Zuniga 2012	26	37	2	36	10.0	0.93 [0.76, 0.99]	0.49 [0.37, 0.61]

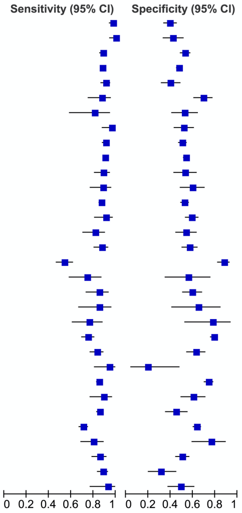

WCC above 10,000 and below 15,000 /mm^3

Study	TP	FP	FN	TN	Threshold	Sensitivity (95% CI)	Specificity (95% CI)
Altali 2017	92	75	24	140	10.5	0.79 [0.71, 0.86]	0.65 [0.58, 0.71]
Groselj-Grenc 2007	36	11	13	22	11.0	0.73 [0.59, 0.85]	0.67 [0.48, 0.82]
Hsiao 2005	86	95	25	16	11.0	0.77 [0.69, 0.85]	0.14 [0.08, 0.22]
Mohammed 2004	109	19	21	67	11.0	0.84 [0.76, 0.90]	0.78 [0.68, 0.86]
Ovrebo 1993	116	34	89	138	11.0	0.57 [0.50, 0.63]	0.80 [0.73, 0.86]
Prada Arias 2017	118	61	14	82	11.0	0.89 [0.83, 0.94]	0.57 [0.49, 0.66]
Wu 2012	245	83	61	205	11.0	0.80 [0.75, 0.84]	0.71 [0.66, 0.76]
Kaiser 2018	22	4	17	21	11.6	0.56 [0.40, 0.72]	0.84 [0.64, 0.95]
Andersson 1999	28	23	20	98	12.0	0.58 [0.43, 0.72]	0.81 [0.73, 0.88]
Andersson 2008	12	22	9	52	12.0	0.57 [0.34, 0.78]	0.70 [0.59, 0.80]
Andersson 2014	42	44	12	83	12.0	0.78 [0.64, 0.88]	0.65 [0.56, 0.74]
Andersson 2017	268	211	65	476	12.0	0.80 [0.76, 0.85]	0.69 [0.66, 0.73]
Bachur 2016	686	409	184	854	12.0	0.79 [0.76, 0.82]	0.68 [0.65, 0.70]
Gonzalez Del Castillo 2016	74	52	37	158	12.0	0.67 [0.57, 0.75]	0.75 [0.69, 0.81]
Khanafer 2016	35	34	19	92	12.0	0.65 [0.51, 0.77]	0.73 [0.64, 0.81]
Kwan 2010	82	32	33	62	12.0	0.71 [0.62, 0.79]	0.66 [0.55, 0.75]
Ozgunur 2014	29	8	5	7	12.0	0.85 [0.69, 0.95]	0.47 [0.21, 0.73]
Shommu 2018	37	21	9	60	12.0	0.80 [0.66, 0.91]	0.74 [0.63, 0.83]
Thompson 2015	380	42	116	60	12.0	0.77 [0.73, 0.80]	0.59 [0.49, 0.68]
Thompson 2017	281	161	190	506	12.0	0.60 [0.55, 0.64]	0.76 [0.72, 0.79]
Miguez 2016	35	14	9	78	12.3	0.80 [0.65, 0.90]	0.85 [0.76, 0.91]
Beltran 2007	67	8	4	42	12.5	0.94 [0.86, 0.98]	0.84 [0.71, 0.93]
Diaz Moreno 2012	59	42	26	58	12.8	0.69 [0.58, 0.79]	0.58 [0.48, 0.68]
Rodriguez Sanchez 2013	36	18	5	46	13.0	0.88 [0.74, 0.96]	0.72 [0.59, 0.82]
Tuncer 2019	104	43	33	121	13.05	0.76 [0.68, 0.83]	0.74 [0.66, 0.80]
Oikonomopoulou 2018	74	33	26	152	13.3	0.74 [0.64, 0.82]	0.82 [0.76, 0.87]
Bal 2016	38	11	18	29	13.4	0.68 [0.54, 0.80]	0.72 [0.56, 0.85]
Greer 2019	63	92	23	368	13.5	0.73 [0.63, 0.82]	0.80 [0.76, 0.84]
Kumar 2021	25	5	30	60	14.0	0.45 [0.32, 0.59]	0.92 [0.83, 0.97]
Sanchez-Echaniz 1997	73	25	21	76	14.0	0.78 [0.68, 0.86]	0.75 [0.66, 0.83]
Peltola 1986	56	11	38	57	14.9	0.60 [0.49, 0.70]	0.84 [0.73, 0.92]

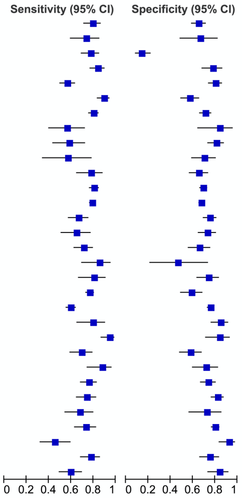

WCC at or above 15,000 /mm^3

Study	TP	FP	FN	TN	Threshold	Sensitivity (95% CI)	Specificity (95% CI)
Andersson 1999	16	5	32	116	15.0	0.33 [0.20, 0.48]	0.96 [0.91, 0.99]
Andersson 2008	9	10	12	64	15.0	0.43 [0.22, 0.66]	0.86 [0.77, 0.93]
Andersson 2014	20	17	34	110	15.0	0.37 [0.24, 0.51]	0.87 [0.79, 0.92]
Andersson 2017	159	103	174	584	15.0	0.48 [0.42, 0.53]	0.85 [0.82, 0.88]
Bachur 2016	485	240	385	1023	15.0	0.56 [0.52, 0.59]	0.81 [0.79, 0.83]
Calvo Rigual 1998	96	22	45	68	15.0	0.68 [0.60, 0.76]	0.76 [0.65, 0.84]
Gonzalez Del Castillo 2016	44	67	28	182	15.0	0.61 [0.49, 0.72]	0.73 [0.67, 0.78]
Kaiser 2018	17	3	22	22	15.0	0.44 [0.28, 0.60]	0.88 [0.69, 0.97]
Khanafer 2016	27	19	27	107	15.0	0.50 [0.36, 0.64]	0.85 [0.77, 0.91]
Lin 2009	23	4	30	43	15.0	0.43 [0.30, 0.58]	0.91 [0.80, 0.98]
Ozgunur 2014	19	8	15	7	15.0	0.56 [0.38, 0.73]	0.47 [0.21, 0.73]
Shommu 2018	30	11	16	70	15.0	0.65 [0.50, 0.79]	0.86 [0.77, 0.93]
Thompson 2015	252	24	244	78	15.0	0.51 [0.46, 0.55]	0.76 [0.67, 0.84]
Thompson 2017	195	82	276	585	15.0	0.41 [0.37, 0.46]	0.88 [0.85, 0.90]

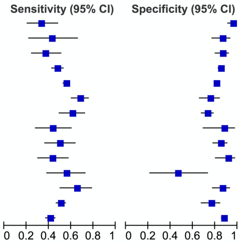

WCC threshold depending on age

Study	TP	FP	FN	TN	Threshold	Sensitivity (95% CI)	Specificity (95% CI)
Doraiswamy 1979	94	5	131	145		0.42 [0.35, 0.49]	0.97 [0.92, 0.99]
Klein 2021	40	21	13	60		0.75 [0.62, 0.86]	0.74 [0.63, 0.83]
Salo 2016	13	11	9	11		0.59 [0.36, 0.79]	0.50 [0.28, 0.72]
Turkyilmaz 2006	43	9	18	35		0.70 [0.57, 0.81]	0.80 [0.65, 0.90]
Wang 2007	50	67	24	269		0.68 [0.56, 0.78]	0.80 [0.75, 0.84]

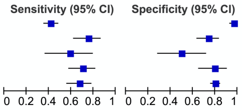

WCC threshold depending on duration pain

Study	TP	FP	FN	TN	Threshold	Sensitivity (95% CI)	Specificity (95% CI)
Dadeh 2021	73	434	24	512		0.75 [0.65, 0.83]	0.54 [0.51, 0.57]

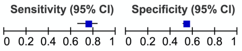

Supplementary Figure 3: Forest plot showing all studies reporting diagnostic accuracy of CPR, split by reported cut off, in paediatric appendicitis. (Sensitivity and specificity for each study represented visually (blue square) with 95% confidence interval (line)).

CRP at or below 5 mg/l

Study	TP	FP	FN	TN	Threshold	Sensitivity (95% CI)	Specificity (95% CI)
Bal 2016	50	24	6	16	0.9	0.89 [0.78, 0.96]	0.40 [0.25, 0.57]
Miguez 2016	30	29	14	63	3.0	0.68 [0.52, 0.81]	0.68 [0.58, 0.78]
Lycopoulou 2005	34	1	8	17	4.5	0.81 [0.66, 0.91]	0.94 [0.73, 1.00]
Akgul 2021	146	72	44	59	5.0	0.77 [0.70, 0.83]	0.45 [0.36, 0.54]
Altali 2017	92	93	24	122	5.0	0.79 [0.71, 0.86]	0.57 [0.50, 0.63]
Andersson 1999	29	34	17	85	5.0	0.63 [0.48, 0.77]	0.71 [0.62, 0.79]
Andersson 2008	17	55	4	17	5.0	0.81 [0.58, 0.95]	0.24 [0.14, 0.35]
Andersson 2014	55	127	0	0	5.0	1.00 [0.94, 1.00]	0.00 [0.00, 0.03]
Andersson 2017	285	481	38	211	5.0	0.88 [0.84, 0.92]	0.30 [0.27, 0.34]
Chiang 2020	589	394	129	279	5.0	0.82 [0.79, 0.85]	0.41 [0.38, 0.45]
Gonzalez Del Castillo 2016	88	90	23	120	5.0	0.79 [0.71, 0.86]	0.57 [0.50, 0.64]
Greer 2019	69	133	17	327	5.0	0.80 [0.70, 0.88]	0.71 [0.67, 0.75]
Kaiser 2018	23	11	16	14	5.0	0.59 [0.42, 0.74]	0.56 [0.35, 0.76]
Malia 2019	160	118	65	419	5.0	0.71 [0.65, 0.77]	0.78 [0.74, 0.81]
Ozgunur 2014	32	9	2	6	5.0	0.94 [0.80, 0.99]	0.40 [0.16, 0.68]
Rodriguez Sanchez 2013	21	29	21	35	5.0	0.50 [0.34, 0.66]	0.55 [0.42, 0.67]
Yap 2015	99	143	20	112	5.0	0.83 [0.75, 0.89]	0.44 [0.38, 0.50]

CRP between 5 and 11 mg/l

Study	TP	FP	FN	TN	Threshold	Sensitivity (95% CI)	Specificity (95% CI)
Zouari 2016	107	74	32	79	6.0	0.77 [0.69, 0.84]	0.52 [0.43, 0.60]
Naqvi 2019	64	20	15	63	7.9	0.81 [0.71, 0.89]	0.76 [0.65, 0.85]
Mohammed 2004	114	13	16	73	8.0	0.88 [0.81, 0.93]	0.85 [0.76, 0.92]
Groselj-Grenc 2007	36	15	13	18	9.0	0.73 [0.59, 0.85]	0.55 [0.36, 0.72]
Ko 1995	19	7	8	13	9.0	0.70 [0.50, 0.86]	0.65 [0.41, 0.85]
Andersson 1999	23	26	23	93	10.0	0.50 [0.35, 0.65]	0.78 [0.70, 0.85]
Andersson 2008	15	25	6	47	10.0	0.71 [0.48, 0.89]	0.65 [0.53, 0.76]
Andersson 2014	43	53	12	74	10.0	0.78 [0.65, 0.88]	0.58 [0.49, 0.67]
Andersson 2017	221	249	102	443	10.0	0.68 [0.63, 0.73]	0.64 [0.60, 0.68]
Gonzalez Del Castillo 2016	75	70	36	140	10.0	0.68 [0.58, 0.76]	0.67 [0.60, 0.73]
Kaiser 2018	20	10	19	15	10.0	0.51 [0.35, 0.68]	0.60 [0.39, 0.79]
Lin 2009	39	12	14	35	10.0	0.74 [0.60, 0.85]	0.74 [0.60, 0.86]
Lycopoulou 2005	26	1	16	17	10.0	0.62 [0.46, 0.76]	0.94 [0.73, 1.00]
Ovrebo 1993	48	80	5	27	10.0	0.91 [0.79, 0.97]	0.25 [0.17, 0.35]
Ozgunur 2014	25	7	9	8	10.0	0.74 [0.56, 0.87]	0.53 [0.27, 0.79]
Peltola 1986	60	12	34	56	10.0	0.64 [0.53, 0.73]	0.82 [0.71, 0.91]
Kumar 2021	49	30	6	35	10.5	0.89 [0.78, 0.96]	0.54 [0.41, 0.66]

CRP between 11 and 50 mg/l

Study	TP	FP	FN	TN	Threshold	Sensitivity (95% CI)	Specificity (95% CI)
Oikonomopoulou 2018	60	52	40	133	12.5	0.60 [0.50, 0.70]	0.72 [0.65, 0.78]
Salo 2016	14	9	8	13	15.0	0.64 [0.41, 0.83]	0.59 [0.36, 0.79]
Ozgunur 2014	24	5	10	10	16.0	0.71 [0.53, 0.85]	0.67 [0.38, 0.88]
Diaz Moreno 2012	46	31	39	68	18.0	0.54 [0.43, 0.65]	0.69 [0.59, 0.78]
Benito 2016	31	13	58	83	20.0	0.35 [0.25, 0.46]	0.86 [0.78, 0.93]
Calvo Rigual 1998	61	34	80	56	20.0	0.43 [0.35, 0.52]	0.62 [0.51, 0.72]
Shommu 2018	24	9	24	67	20.0	0.50 [0.35, 0.65]	0.88 [0.79, 0.94]
Wu 2012	214	78	92	210	25.0	0.70 [0.64, 0.75]	0.73 [0.67, 0.78]
Prada Arias 2017	57	29	75	114	25.2	0.43 [0.35, 0.52]	0.80 [0.72, 0.86]
Andersson 1999	14	14	32	105	30.0	0.30 [0.18, 0.46]	0.88 [0.81, 0.93]
Andersson 2008	8	11	13	61	30.0	0.38 [0.18, 0.62]	0.85 [0.74, 0.92]
Andersson 2014	31	35	24	92	30.0	0.56 [0.42, 0.70]	0.72 [0.64, 0.80]
Andersson 2017	140	128	183	564	30.0	0.43 [0.38, 0.49]	0.82 [0.78, 0.84]
Cayrol 2016	20	12	32	70	30.0	0.38 [0.25, 0.53]	0.85 [0.76, 0.92]
Gonzalez Del Castillo 2016	59	40	52	170	30.0	0.53 [0.43, 0.63]	0.81 [0.75, 0.86]
Kaiser 2018	13	8	26	17	30.0	0.33 [0.19, 0.50]	0.68 [0.46, 0.85]
Kwan 2010	81	33	34	61	30.0	0.70 [0.61, 0.79]	0.65 [0.54, 0.74]
Sanchez-Echaniz 1997	40	8	54	93	30.0	0.43 [0.32, 0.53]	0.92 [0.85, 0.97]
Beltran 2007	68	4	3	46	47.0	0.96 [0.88, 0.99]	0.92 [0.81, 0.98]

CRP at or above 50 mg/l

Study	TP	FP	FN	TN	Threshold	Sensitivity (95% CI)	Specificity (95% CI)
Andersson 1999	7	10	39	109	50.0	0.15 [0.06, 0.29]	0.92 [0.85, 0.96]
Andersson 2008	5	7	16	65	50.0	0.24 [0.08, 0.47]	0.90 [0.81, 0.96]
Andersson 2014	20	21	35	106	50.0	0.36 [0.24, 0.50]	0.83 [0.76, 0.89]
Andersson 2017	93	62	230	630	50.0	0.29 [0.24, 0.34]	0.91 [0.89, 0.93]
Gonzalez Del Castillo 2016	37	28	74	182	50.0	0.33 [0.25, 0.43]	0.87 [0.81, 0.91]
Kaiser 2018	9	2	30	23	50.0	0.23 [0.11, 0.39]	0.92 [0.74, 0.99]
Ko 1995	14	1	13	19	50.0	0.52 [0.32, 0.71]	0.95 [0.75, 1.00]
Ozgunur 2014	15	3	19	12	50.0	0.44 [0.27, 0.62]	0.80 [0.52, 0.96]
Shommu 2018	12	1	36	75	50.0	0.25 [0.14, 0.40]	0.99 [0.93, 1.00]
Wu 2012	214	49	92	239	89.0	0.70 [0.64, 0.75]	0.83 [0.78, 0.87]

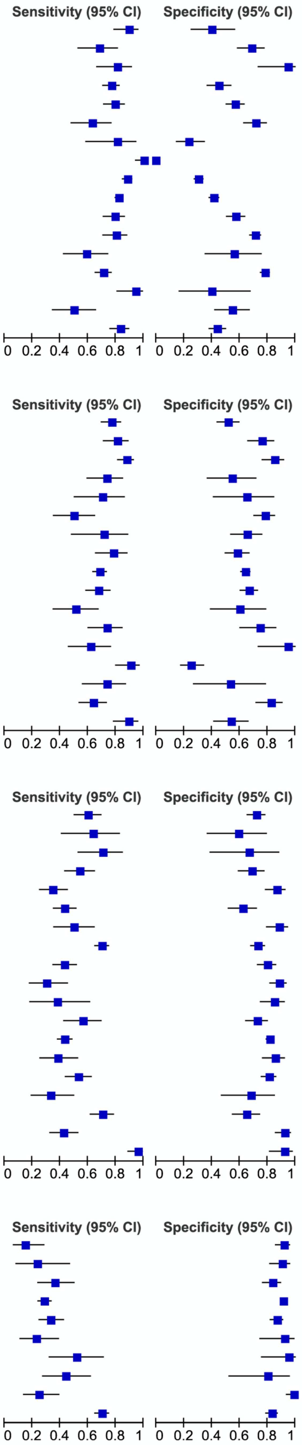

Supplementary Figure 4: Forest plot showing all studies reporting diagnostic accuracy of ANC in paediatric appendicitis. (Sensitivity and specificity for each study represented visually (blue square) with 95% confidence interval (line)).

Study	TP	FP	FN	TN	Threshold	Sensitivity (95% CI)	Specificity (95% CI)
Salo 2016	16	7	6	15	age dependent	0.73 [0.50, 0.89]	0.68 [0.45, 0.86]
Altali 2017	99	85	17	130	6.6	0.85 [0.78, 0.91]	0.60 [0.54, 0.67]
Kharbanda 2012	942	824	76	783	6.73	0.93 [0.91, 0.94]	0.49 [0.46, 0.51]
Fleischman 2013	53	56	12	57	6.75	0.82 [0.70, 0.90]	0.50 [0.41, 0.60]
Kharbanda 2005	152	131	5	137	6.75	0.97 [0.93, 0.99]	0.51 [0.45, 0.57]
Prada Arias 2017	119	57	13	86	7.0	0.90 [0.84, 0.95]	0.60 [0.52, 0.68]
Akgul 2021	163	74	27	56	7.5	0.86 [0.80, 0.90]	0.43 [0.34, 0.52]
Andersson 1999	34	38	2	61	7.5	0.94 [0.81, 0.99]	0.62 [0.51, 0.71]
Andersson 2008	8	27	1	33	7.5	0.89 [0.52, 1.00]	0.55 [0.42, 0.68]
Andersson 2014	35	32	3	41	7.5	0.92 [0.79, 0.98]	0.56 [0.44, 0.68]
Andersson 2017	274	282	31	322	7.5	0.90 [0.86, 0.93]	0.53 [0.49, 0.57]
Benito 2016	78	37	11	59	7.5	0.88 [0.79, 0.94]	0.61 [0.51, 0.71]
Goldman 2008	84	67	17	121	7.5	0.83 [0.74, 0.90]	0.64 [0.57, 0.71]
Greer 2019	69	115	17	345	7.5	0.80 [0.70, 0.88]	0.75 [0.71, 0.79]
Samuel 2002	595	122	139	314	7.5	0.81 [0.78, 0.84]	0.72 [0.68, 0.76]
Shommu 2018	40	24	6	57	7.5	0.87 [0.74, 0.95]	0.70 [0.59, 0.80]
Thompson 2015	351	68	47	45	7.5	0.88 [0.85, 0.91]	0.40 [0.31, 0.49]
Thompson 2017	319	206	141	457	7.5	0.69 [0.65, 0.74]	0.69 [0.65, 0.72]
Zuniga 2012	27	37	1	36	7.5	0.96 [0.82, 1.00]	0.49 [0.37, 0.61]
Corkum 2018	116	166	40	416	8.0	0.74 [0.67, 0.81]	0.71 [0.68, 0.75]
Bal 2016	43	13	13	27	9.4	0.77 [0.64, 0.87]	0.68 [0.51, 0.81]
Miguez 2016	37	16	7	76	9.6	0.84 [0.70, 0.93]	0.83 [0.73, 0.90]
Bachur 2016	630	365	240	898	10.0	0.72 [0.69, 0.75]	0.71 [0.69, 0.74]
Kumar 2021	28	8	27	57	10.48	0.51 [0.37, 0.65]	0.88 [0.77, 0.95]
Oikonomopoulou 2018	71	26	29	159	10.8	0.71 [0.61, 0.80]	0.86 [0.80, 0.91]

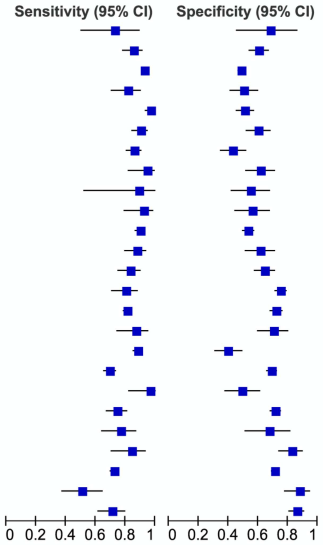

Supplementary Figure 5: Forest plot showing all studies reporting diagnostic accuracy of Neutrophil count as a percentage in paediatric appendicitis. (Sensitivity and specificity for each study represented visually (blue square) with 95% confidence interval (line)).

Study	TP	FP	FN	TN	Threshold	Sensitivity (95% CI)	Specificity (95% CI)
Anadalwar 2015	340	241	53	211	65%	0.87 [0.83, 0.90]	0.47 [0.42, 0.51]
Salo 2016	16	10	6	12	71%	0.73 [0.50, 0.89]	0.55 [0.32, 0.76]
Andersson 1999	31	45	5	54	75%	0.86 [0.71, 0.95]	0.55 [0.44, 0.65]
Andersson 2008	9	29	3	31	75%	0.75 [0.43, 0.95]	0.52 [0.38, 0.65]
Andersson 2014	30	29	8	44	75%	0.79 [0.63, 0.90]	0.60 [0.48, 0.72]
Chiang 2020	524	224	194	449	75%	0.73 [0.70, 0.76]	0.67 [0.63, 0.70]
Dadeh 2021	74	304	23	642	75%	0.76 [0.67, 0.84]	0.68 [0.65, 0.71]
Fleischman 2013	50	48	15	65	75%	0.77 [0.65, 0.86]	0.58 [0.48, 0.67]
Hsiao 2005	87	67	24	44	75%	0.78 [0.70, 0.86]	0.40 [0.30, 0.49]
Klein 2021	36	21	17	60	75%	0.68 [0.54, 0.80]	0.74 [0.63, 0.83]
Ko 1995	22	6	5	14	75%	0.81 [0.62, 0.94]	0.70 [0.46, 0.88]
Mandeville 2011	119	48	36	84	75%	0.77 [0.69, 0.83]	0.64 [0.55, 0.72]
Mohammed 2004	111	15	19	71	75%	0.85 [0.78, 0.91]	0.83 [0.73, 0.90]
Yap 2015	79	80	40	175	75%	0.66 [0.57, 0.75]	0.69 [0.63, 0.74]
Wang 2007	44	34	30	302	80%	0.59 [0.47, 0.71]	0.90 [0.86, 0.93]
Doraiswamy 1979	216	47	9	103	age dependent	0.96 [0.93, 0.98]	0.69 [0.61, 0.76]

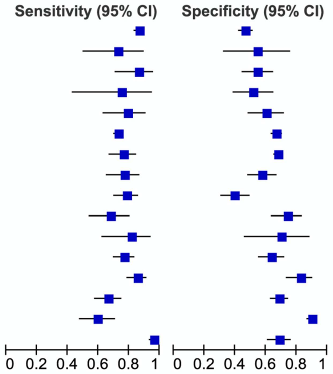

Supplementary Figure 6: Forest plot showing all studies reporting diagnostic accuracy of procalcitonin in paediatric appendicitis. (Sensitivity and specificity for each study represented visually (blue square) with 95% confidence interval (line)).

Study	TP	FP	FN	TN	Threshold	Sensitivity (95% CI)	Specificity (95% CI)
Benito 2016	34	34	55	62	0.1	0.38 [0.28, 0.49]	0.65 [0.54, 0.74]
Bal 2016	18	4	38	36	0.37	0.32 [0.20, 0.46]	0.90 [0.76, 0.97]
Khan 2012	6	2	16	26	0.39	0.27 [0.11, 0.50]	0.93 [0.76, 0.99]
Rodriguez Sanchez 2013	10	18	34	46	0.5	0.23 [0.11, 0.38]	0.72 [0.59, 0.82]
Kouame 2005	19	4	49	29	0.5	0.28 [0.18, 0.40]	0.88 [0.72, 0.97]
Akgul 2021	55	19	135	111	0.5	0.29 [0.23, 0.36]	0.85 [0.78, 0.91]

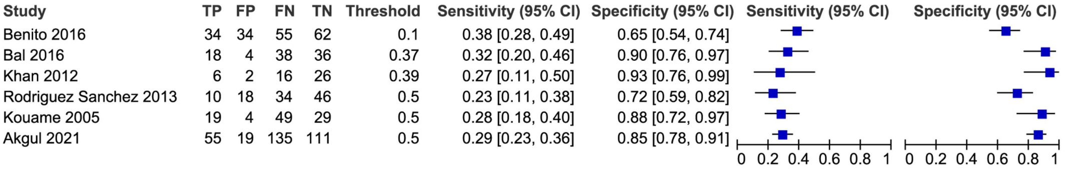

Fawkner-Corbett D, *et al. BMJ Open* 2022; 12:e056854. doi: 10.1136/bmjopen-2021-056854

Supplementary Figure 7: Forest plot showing all studies reporting diagnostic accuracy of combination of WCC and/or CRP in paediatric appendicitis. (Sensitivity and specificity for each study represented visually (blue square) with 95% confidence interval (line)).

CRP and WCC

Study	TP	FP	FN	TN	Threshold WCC - CRP	Sensitivity (95% CI)	Specificity (95% CI)
Andersson 1999	20	12	26	107	10.0 - 10	0.43 [0.29, 0.59]	0.90 [0.83, 0.95]
Andersson 2008	12	16	9	56	10.0 - 10	0.57 [0.34, 0.78]	0.78 [0.66, 0.87]
Andersson 2014	39	31	14	96	10.0 - 10	0.74 [0.60, 0.85]	0.76 [0.67, 0.83]
Andersson 2017	197	150	122	521	10.0 - 10	0.62 [0.56, 0.67]	0.78 [0.74, 0.81]
Calvo Rigual 1998	113	45	28	45	15.0 - 20	0.80 [0.73, 0.86]	0.50 [0.39, 0.61]
Kaiser 2018	16	7	23	18	10.0 - 10	0.41 [0.26, 0.58]	0.72 [0.51, 0.88]
Kwan 2010	48	8	67	86	12.0 - 30	0.42 [0.33, 0.51]	0.91 [0.84, 0.96]
Ovrebo 1993	38	30	14	77	11.0- 10	0.73 [0.59, 0.84]	0.72 [0.62, 0.80]
Ozgunur 2014	24	5	10	10	10.0 - 10	0.71 [0.53, 0.85]	0.67 [0.38, 0.88]
Peltola 1986	42	20	52	48	14.9 - 100	0.45 [0.34, 0.55]	0.71 [0.58, 0.81]
Sanchez-Echaniz 1997	32	5	62	96	14.0 - 30	0.34 [0.25, 0.45]	0.95 [0.89, 0.98]

CRP or WCC

Study	TP	FP	FN	TN	Threshold WCC - CRP	Sensitivity (95% CI)	Specificity (95% CI)
Andersson 1999	44	50	2	69	10.0 - 10	0.96 [0.85, 0.99]	0.58 [0.49, 0.67]
Andersson 2008	20	43	1	29	10.0 - 10	0.95 [0.76, 1.00]	0.40 [0.29, 0.53]
Andersson 2014	53	83	0	44	10.0 - 10	1.00 [0.93, 1.00]	0.35 [0.26, 0.44]
Andersson 2017	313	425	6	246	10.0 - 10	0.98 [0.96, 0.99]	0.37 [0.33, 0.40]
Chiang 2020	716	514	2	159	10.0 - 5	1.00 [0.99, 1.00]	0.24 [0.20, 0.27]
Huckins 2013	140	238	4	121	10.0 - 8	0.97 [0.93, 0.99]	0.34 [0.29, 0.39]
Huckins 2016	457	775	19	635	10.0 - 8	0.96 [0.94, 0.98]	0.45 [0.42, 0.48]
Kaiser 2018	33	14	6	11	10.0 - 10	0.85 [0.69, 0.94]	0.44 [0.24, 0.65]
Ovrebo 1993	62	86	0	21	11.0- 10	1.00 [0.94, 1.00]	0.20 [0.13, 0.28]
Ozgunur 2014	33	14	1	1	10.0 - 10	0.97 [0.85, 1.00]	0.07 [0.00, 0.32]

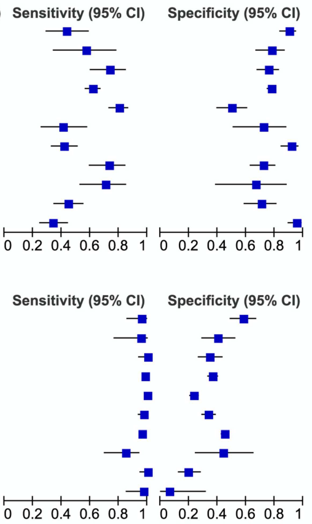

Supplementary Figure 8: Forest plot showing all studies reporting diagnostic accuracy of combination of WCC, CRP and/or NC in paediatric appendicitis. (Sensitivity and specificity for each study represented visually (blue square) with 95% confidence interval (line)).

WCC, CRP, and Neutrophils

Study	TP	FP	FN	TN	Threshold WCC - CRP - NC%	Sensitivity (95% CI)	Specificity (95% CI)
Andersson 1999	15	11	20	88	10.0 - 10 - 75%	0.43 [0.26, 0.61]	0.89 [0.81, 0.94]
Andersson 2008	6	11	6	48	10.0 - 10 - 75%	0.50 [0.21, 0.79]	0.81 [0.69, 0.90]
Andersson 2014	21	12	17	61	10.0 - 10 - 75%	0.55 [0.38, 0.71]	0.84 [0.73, 0.91]
Andersson 2017	139	89	152	498	10.0 - 10 - 75%	0.48 [0.42, 0.54]	0.85 [0.82, 0.88]
Mohammed 2004	112	8	18	78	11.0 - 80 - 75%	0.86 [0.79, 0.92]	0.91 [0.82, 0.96]

WCC, CRP, or Neutrophils

Study	TP	FP	FN	TN	Threshold WCC - CRP - NC%	Sensitivity (95% CI)	Specificity (95% CI)
Yap 2015	119	192	0	63	10.0 - 5 - 75%	1.00 [0.97, 1.00]	0.25 [0.20, 0.30]

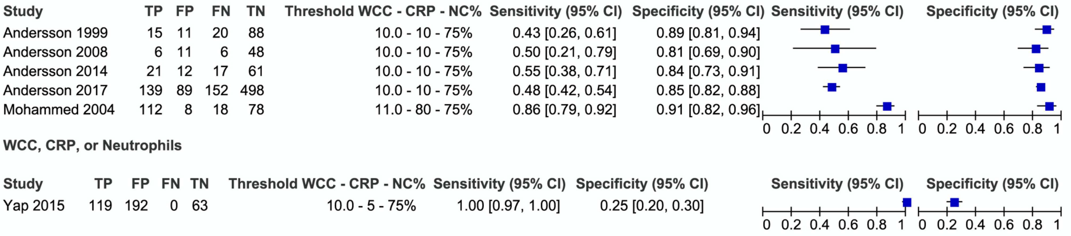

Search Strategy:
Searches were performed on the 15th March 2022 with search terms that had been informed by scoping searches. Databases searched included Cochrane Database of Systematic Reviews, Cochrane Central Register of controlled trials, Database of abstracts of Reviews of Effects, Embase, Ovid(Medline) Epub Ahead of print in process and other Non-Indexed Citations, Ovid Medline(R) Daily, Ovid Medline(R) and Science Citation Index and Conference Proceedings Citation Index (SCIENCE). These databases were searched through the interfaces Cochrane Library(Wiley), OvidSP and Web of Science Core Collection. Specific terms used for these interfaces are detailed below, following searching all results were merged and checked for duplicates before review (**Figure 1**).

Medline:

#	Searches	Results
1	adolescent/ or exp child/ or exp infant/	3410061
2	(child* or schoolchild* or preschool* or pre-school* or boys or girls or infant? or pediatric* or paediatric* or adolescen* or teen* or youth?).ti,ab.	1876871
3	1 or 2	3876495
4	exp Leukocyte Count/ or leukocytes/ or neutrophils/	225318
5	(leukocyte? or white cell or white blood cell or neutrophil*).ti,ab.	272998
6	(wbcc or wcc).ti,ab.	655
7	C-Reactive Protein/	42976
8	(c-reactive protein? or crp).ti,ab.	75114
9	Calcitonin/	15622
10	(procalcitonin or calcitonin or pct).ti,ab.	32717
11	4 or 5 or 6 or 7 or 8 or 9 or 10	490743
12	Appendicitis/	18470
13	Appendectomy/	10586
14	(appendix or appendicitis or appendectom* or appendicectom*).ti,ab.	33426
15	12 or 13 or 14	38889
16	3 and 11 and 15	787

Embase:

#	Searches	Results
1	juvenile/ or exp adolescent/ or exp child/	3172209
2	(child* or schoolchild* or preschool* or pre-school* or boys or girls or infant? or pediatric* or paediatric* or adolescen* or teen* or youth?).ti,ab.	2298797
3	1 or 2	3780916
4	exp leukocyte count/ or leukocyte/ or neutrophil/	394999
5	(leukocyte? or white cell or white blood cell or neutrophil*).ti,ab.	364068
6	(wbcc or wcc).ti,ab.	1603
7	C reactive protein/	158251
8	(c-reactive protein? or crp).ti,ab.	129235
9	Procalcitonin/	10738
10	(procalcitonin or calcitonin or pct).ti,ab.	43836

11	4 or 5 or 6 or 7 or 8 or 9 or 10	749518
12	exp appendicitis/	22927
13	appendectomy/	18892
14	(appendix or appendicitis or appendectom* or appendicectom*).ti,ab.	37059
15	12 or 13 or 14	46062
16	3 and 11 and 15	1369

Cochrane:

#	Searches
1	MeSH descriptor: [Adolescent] explode all trees
2	MeSH descriptor: [Child] explode all trees
3	MeSH descriptor: [Infant] explode all trees
4	exp leukocyte count/ or leukocyte/ or neutrophil/
5	child* or schoolchild* or preschool* or pre-school* or boys or girls or infant? or pediatric* or paediatric* or adolescen* or teen* or youth?:ti,ab,kw (Word variations have been searched)
6	#1 or #2 or #3 or #4
7	MeSH descriptor: [Leukocyte Count] explode all trees
8	MeSH descriptor: [Leukocytes] this term only
9	MeSH descriptor: [Neutrophils] explode all trees
10	wbcc or wcc:ti,ab,kw (Word variations have been searched)
11	MeSH descriptor: [C-Reactive Protein] explode all trees
12	c-reactive protein* or crp:ti,ab,kw (Word variations have been searched)
13	MeSH descriptor: [Calcitonin] explode all trees
14	procalcitonin or calcitonin or pct:ti,ab,kw
15	#6 or #7 or #8 or #9 or #10 or #11 or #12 or #13 or #14
16	MeSH descriptor: [Appendicitis] explode all trees
17	MeSH descriptor: [Appendectomy] explode all trees
18	appendix or appendicitis or appendectom* or appendicectom*:ti,ab,kw (Word variations have been searched)
19	#16 or #17 or #18
20	#5 and #15 and #19

Web of Science:

#	Searches	Results
1	TS=(child* or schoolchild* or preschool* or pre-school* or boys or girls or infant? or pediatric* or paediatric* or adolescen* or teen* or youth?)	1709598
2	TS=(leukocyte* or "white cell" or "white blood cell" or neutrophil*) OR TS=("c-reactive protein" OR "c reactive protein" OR crp) OR TS=(procalcitonin OR calcitonin OR pct)	433876
3	TS=(appendix or appendicitis or appendectom* or appendicectom*)	33245
4	#3 AND #2 AND #1	397

QUADAS-2 Scoring criteria

Below is an overview, with examples of how the QUADAS-2 scoring criteria was used to measure risk of bias and applicability in selected studies(69). In those patients not undergoing surgery the optimal reference standard was follow up after 2 weeks to confirm absence of disease. Studies including only patients who had undergone a radiological investigation were included but this caveat was noted when assessing these studies for risk of bias. Study quality was not an exclusion criteria.

Domain 1: Patient Selection

- Was a consecutive or random sample of patients enrolled? (Yes/Unclear/No)
    - *Scored yes if the words in article state "consecutive", "random", or "all patients were included*
- Was a case-control design avoided? (Yes/Unclear/No)
    - *Scored yes if article stated the study design as not being case-control*
- Did the study avoid inappropriate exclusions? (Yes/Unclear/No)
    - *Scored yes if studies excluded no children with abdominal pain or suspected appendicitis.*
- (Risk) Could the selection of patients have introduced bias? (Low / Unclear/ High)
    - *Scored low if all answers to signalling questions were yes. Score high if any were answered no. Score unclear if any were answered as unclear with remainder scoring low.*
- (Concern) Is there concern that included patients do not match the review question? (Low / Unclear /High)
    - *Scored low if all patients were <18 years, had symptoms in keeping with appendicitis and no previous diagnosis of appendicitis*

Domain 2: Index Test

- Were the index tests interpreted without knowledge of the results of the reference standard? (Yes / Unclear/ No)
    - *Scored yes if text stated blood test results were concealed or blinded to clinician or pathologist.*
- If a threshold was used, was it pre-specified? (Yes / Unclear/ No)
    - *Scored yes if a cut-off for index test was stated before commencing study or analysing results*
- (Risk) Could the conduct or interpretation of the index test have introduced bias? (Low / Unclear/ High)
    - *Scored low if all answers to signalling questions were yes. Score high if any were answered no. Score unclear if any were answered as unclear with remainder scoring low.*
- (Concern) Is there concern that the index test, its conduct, or interpretation differ from the review question? (Low / Unclear / High)
    - *Scored low if the study provides a clear description of the index test and a clear description or definition of a positive test result*

Domain 3: Reference Standard

- Is the reference standard likely to correctly classify the target condition? (Yes / Unclear/ No)
    - *Score yes if the reference standard was histology after surgery or clinical follow up of patient after 2 weeks. Score no if the reference standard was surgery alone without histology.*
- Were the reference standard results interpreted without knowledge of the results of the index test? (Yes / Unclear/ No)
    - *Score yes if study states the assessment of reference standard was done without knowledge or with blinding of the results of the index test*
- (Risk) Could the reference standard, its conduct, or its interpretation have introduced bias? (Low / Unclear/ High)
    - *Scored low if all answers to signalling questions were yes. Score high if any were answered no. Score unclear if any were answered as unclear with remainder scoring low.*
- (Concern) Is there concern that the target condition as defined by the reference standard does not match the review question? (Low / Unclear/ High)
    - *Score Low if all patients were in keeping with the target condition of appendicitis as defined by histology after surgery or refuted by clinical follow-up of greater than or equal to 2 weeks*

Domain 4: Flow and Timing

- Was there an appropriate interval between index test(s) and reference standard? (Yes / Unclear/ No)
    - *Score yes if the time period was 48 hours or less and was stated in text; Score no if the time period was more than 48 hours. Score unclear if insufficient information to determine the interval between index test and reference standard.*

- Did all patients receive a reference standard? (Yes / Unclear/ No)
  - *Score yes if all patients received either appendicectomy with histology or clinical follow up of greater than or equal to 2 weeks to refute appendicitis*
- Did patients receive the same reference standard? (Yes / Unclear/ No)
  - *Score yes if all patients received the same reference standard.*
- Were all patients included in the analysis? (Yes / Unclear/ No)
  - *Score yes if all patients included at patient selection underwent index test and reference standard.*
- (Risk) Could the patient flow have introduced bias? (Low / Unclear/ High)
  - *Scored low if all answers to signalling questions were yes. Score high if any were answered no. Score unclear if any were answered as unclear with remainder scoring low.*

Supplementary References

1. Akgül F, Er A, Ulusoy E, Çağlar A, Çitlenbik H, Keskinoğlu P, et al. Integration of Physical Examination, Old and New Biomarkers, and Ultrasonography by Using Neural Networks for Pediatric Appendicitis. Pediatr Emerg Care 2021 Dec 1;37(12):e1075-e1081.
2. Altali K, Ruiz-Artacho P, Trenchs V, Martinez Ortiz de Zarate M, Navarro C, Fernandez C, et al. [Hospital emergency room diagnosis of acute appendicitis in patients aged 2 to 20 years: the INFURG-SEMES score from the emergency infections study of the Spanish Society of Emergency Medicine]. Emergencias : revista de la Sociedad Espanola de Medicina de Emergencias. 2017;29(4):231-6.
3. Anandalwar SP, Callahan MJ, Bachur RG, Feng C, Sidhwa F, Karki M, et al. Use of White Blood Cell Count and Polymorphonuclear Leukocyte Differential to Improve the Predictive Value of Ultrasound for Suspected Appendicitis in Children. Journal of the American College of Surgeons. 2015;220(6):1010-7.
4. Andersson RE, Hugander AP, Ghazi SH, Ravn H, Offenbartl SK, Nystrom PO, et al. Diagnostic value of disease history, clinical presentation, and inflammatory parameters of appendicitis. World J Surg. 1999;23(2):133-40.
5. Andersson M, Andersson RE. The appendicitis inflammatory response score: a tool for the diagnosis of acute appendicitis that outperforms the Alvarado score. World J Surg. 2008;32(8):1843-9.
6. Andersson M, Rubér M, Ekerfelt C, Hallgren HB, Olaison G, Andersson RE. Can New Inflammatory Markers Improve the Diagnosis of Acute Appendicitis? World Journal of Surgery. 2014;38(11):2777-83.
7. Andersson M, Kolodziej B, Andersson RE. Randomized clinical trial of Appendicitis Inflammatory Response score-based management of patients with suspected appendicitis. The British journal of surgery. 2017;104(11):1451-61.
8. Bachur RG, Dayan PS, Dudley NC, Bajaj L, Stevenson MD, Macias CG, et al. The Influence of Age on the Diagnostic Performance of White Blood Cell Count and Absolute Neutrophil Count in Suspected Pediatric Appendicitis. Academic emergency medicine : official journal of the Society for Academic Emergency Medicine. 2016;23(11):1235-42.
9. Bal A, Anil M, Narturk M, Ozdemir T, Arikan A, Koyluoglu G, et al. Importance of Clinical Decision Making by Experienced Pediatric Surgeons When Children Are Suspected of Having Acute Appendicitis: The Reality in a High-Volume Pediatric Emergency Department. Pediatr Emerg Care. 2017;33(9):e38-e42.
10. Beltrán A M, Almonacid F J, Gutiérrez C J, Cruces B K. Puntuación diagnóstica de apendicitis aguda en niños realizada por pediatras de las Unidades de Emergencia. Revista chilena de pediatría. 2007;78:584-91.
11. Benito J, Acedo Y, Medrano L, Barcena E, Garay RP, Arri EA. Usefulness of new and traditional serum biomarkers in children with suspected appendicitis. The American journal of emergency medicine. 2016;34(5):871-6.
12. Calvo Rigual F, Sendra Esteve S, Mialaret Lahiguera A, Montagud Beltrán E, Llanes Domingo S, Medrano González J. [The value of C-reactive protein in the diagnosis of acute appendicitis in children]. An Esp Pediatr. 1998;48(4):376-80.
13. Cayrol J, Miguez MC, Guerrero G, Tomatis C, Simal I, Maranon R. Diagnostic accuracy and prognostic utility of D Dimer in acute appendicitis in children. European journal of pediatrics. 2016;175(3):313-20.
14. Chiang JJY, Angus MI, Nah SA, Jacobsen AS, Low Y, Choo CSC, et al. Time course response of inflammatory markers in pediatric appendicitis. Pediatr Surg Int 2020 Apr;36(4):493-500.
15. Corkum KS, Oyetunji TA, Grabowski JE, Rigsby CK, Lautz TB. Absolute neutrophil count as a diagnostic guide for the use of MRI in the workup of suspected appendicitis in children. Journal of pediatric surgery. 2019;54(7):1359-64.
16. Dadeh AA, Puitong K. Predictive Factors to Diagnose Appendicitis in Children in the Emergency Department. Open Access Emerg Med 2021 Jul 29;13:363-372.
17. Doraiswamy NV. Leucocyte counts in the diagnosis and prognosis of acute appendicitis in children. The British journal of surgery. 1979;66(11):782-4.
18. Esparaz JR, McGovern GC, Mowrer AR, Nierstedt RT, Biesboer EA, Elger BM, et al. A simple algorithm to improve quality while reducing resource utilization in evaluation of suspected appendicitis in children. American journal of surgery. 2019;217(3):469-72.
19. Fleischman RJ, Devine MK, Yagapen MA, Steichen AJ, Hansen ML, Zigman AF, et al. Evaluation of a novel pediatric appendicitis pathway using high- and low-risk scoring systems. Pediatr Emerg Care. 2013;29(10):1060-5.
20. Goldman RD, Carter S, Stephens D, Antoon R, Mounstephen W, Langer JC. Prospective validation of the pediatric appendicitis score. The Journal of pediatrics. 2008;153(2):278-82.
21. Gonzalez Del Castillo J, Ayuso FJ, Trenchs V, Martinez Ortiz de Zarate M, Navarro C, Altali K, et al. Diagnostic accuracy of the APPY1 Test in patients aged 2-20 years with suspected acute appendicitis presenting to emergency departments. Emergency medicine journal : EMJ. 2016;33(12):853-9.

22. Greer D, Bennett P, Wagstaff B, Croaker D. Lymphopaenia in the diagnosis of paediatric appendicitis: a false sense of security? ANZ J Surg 2019 Sep;89(9):1122-1125.
23. Groselj-Grenc M, Repse S, Dolenc-Strazar Z, Hojker S, Derganc M. Interleukin-6 and lipopolysaccharide-binding protein in acute appendicitis in children. Scandinavian journal of clinical and laboratory investigation. 2007;67(2):197-206.
24. Hsiao KH, Lin LH, Chen DF. Application of the MANTRELS scoring system in the diagnosis of acute appendicitis in children. Acta paediatrica Taiwanica = Taiwan er ke yi xue hui za zhi. 2005;46(3):128-31.
25. Huckins DS, Simon HK, Copeland K, Spiro DM, Gogain J, Wandell M. A novel biomarker panel to rule out acute appendicitis in pediatric patients with abdominal pain. The American journal of emergency medicine. 2013;31(9):1368-75.
26. Huckins DS, Simon HK, Copeland K, Milling TJ, Jr., Spandorfer PR, Hennes H, et al. Prospective validation of a biomarker panel to identify pediatric ED patients with abdominal pain who are at low risk for acute appendicitis. The American journal of emergency medicine. 2016;34(8):1373-82.
27. Kaiser M, Schroeckenfuchs M, Castellani C, Warncke G, Till H, Singer G. The diagnostic value of hepcidin to predict the presence and severity of appendicitis in children. The Journal of surgical research. 2018;222:102-7.
28. Kashtan M, Graham D, Anandalwar S, Hills-Dunlap J, Rangel S. Influence of symptom duration and WBC profile on the negative predictive value of a nondiagnostic ultrasound in children with suspected appendicitis. J Pediatr Surg 2020 Jun;55(6):1032-1036.
29. Khan AN, Sawan A, Likourezos A, Schnellinger M, Garcia E. The usefulness of procalcitonin in the diagnosis of appendicitis in children: a pilot study. Emergency medicine international. 2012;2012:317504.
30. Khanafer I, Martin DA, Mitra TP, Eccles R, Brindle ME, Nettel-Aguirre A, et al. Test characteristics of common appendicitis scores with and without laboratory investigations: a prospective observational study. BMC Pediatr. 2016;16(1):147.
31. Kharbanda AB, Taylor GA, Fishman SJ, Bachur RG. A clinical decision rule to identify children at low risk for appendicitis. Pediatrics. 2005;116(3):709-16.
32. Kharbanda AB, Rai AJ, Cosme Y, Liu K, Dayan PS. Novel serum and urine markers for pediatric appendicitis. Academic emergency medicine : official journal of the Society for Academic Emergency Medicine. 2012;19(1):56-62.
33. Kharbanda AB, Dudley NC, Bajaj L, Stevenson MD, Macias CG, Mittal MK, et al. Validation and refinement of a prediction rule to identify children at low risk for acute appendicitis. Archives of pediatrics & adolescent medicine. 2012;166(8):738-44.
34. Klein TT, Kohn E, Klin B, Ziv-Baran T, Kozer E, Berkovitch M, et al. sTREM-1 as a diagnostic biomarker for acute appendicitis in children. Asian J Surg 2021 Sep;44(9):1172-1178.
35. Ko YS, Lin LH, Chen DF. Laboratory aid and ultrasonography in the diagnosis of appendicitis in children. Zhonghua Minguo xiao er ke yi xue hui za zhi [Journal] Zhonghua Minguo xiao er ke yi xue hui. 1995;36(6):415-9.
36. Kouame DB, Garrigue MA, Lardy H, Machet MC, Giraudeau B, Robert M. [Is procalcitonin able to help in pediatric appendicitis diagnosis?]. Annales de chirurgie. 2005;130(3):169-74.
37. Kumar MSV, Tiwari MK, Singh J, Malik A. Plasma Fibrinogen: An Independent Predictor of Pediatric Appendicitis. J Indian Assoc Pediatr Surg 2021 Jul-Aug;26(4):240-245.
38. Kwan KY, Nager AL. Diagnosing pediatric appendicitis: usefulness of laboratory markers. The American journal of emergency medicine. 2010;28(9):1009-15.
39. Lin CH, Chen JH, Li TC, Ho YJ, Lin WC. Children presenting at the emergency department with right lower quadrant pain. The Kaohsiung journal of medical sciences. 2009;25(1):1-9.
40. Lycopoulou L, Mamoulakis C, Hantzi E, Demetriadis D, Antypas S, Giannaki M, et al. Serum amyloid A protein levels as a possible aid in the diagnosis of acute appendicitis in children. Clinical chemistry and laboratory medicine. 2005;43(1):49-53.
41. Malia L, Sturm JJ, Smith SR, Brown RT, Campbell B, Chicaiza H. Predictors for Acute Appendicitis in Children. Pediatr Emerg Care. 2019.
42. Mandeville K, Pottker T, Bulloch B, Liu J. Using appendicitis scores in the pediatric ED. The American journal of emergency medicine. 2011;29(9):972-7.
43. Miguez C, Tomatis Souverbielle C, Haro A, Guerrero G, Perez-Egido L, Garcia-Gamiz M, et al. Evaluation of proadrenomedullin as a diagnostic or prognostic biomarker of acute appendicitis in children. The American journal of emergency medicine. 2016;34(12):2298-305.
44. Mohammed AA, Daghman NA, Aboud SM, Oshibi HO. The diagnostic value of C-reactive protein, white blood cell count and neutrophil percentage in childhood appendicitis. Saudi medical journal. 2004;25(9):1212-5.

45. Diaz Moreno E, Garcia Gomez M, Castejon Casado J, Liceras Liceras E, Martin Cano F, Munoz Miguelsanz MA. [Analysis of the medical decision in abdominal pain suggestive of acute appendicitis]. Cirugia pediatrica : organo oficial de la Sociedad Espanola de Cirugia Pediatrica. 2012;25(1):40-5.
46. Naqvi SA, Thompson GC, Joffe AR, Blackwood J, Martin DA, Brindle M, et al. Cytokines and Chemokines in Pediatric Appendicitis: A Multiplex Analysis of Inflammatory Protein Mediators. Mediators of inflammation. 2019;2019:2359681.
47. Oikonomopoulou N, Miguez-Navarro C, Rivas-Garcia A, Garcia Gamiz M, Lopez-Lopez R, Oliver-Saez P, et al. Assessment of proadrenomedullin as diagnostic or prognostic biomarker of acute appendicitis in children with acute abdominal pain. The American journal of emergency medicine. 2019;37(7):1289-94.
48. Ovrebo KK, Eckerbom RM, Haram S, Rokke O. [Acute abdomen among children and adolescents. A retrospective study of 470 children and adolescents with acute abdominal pain]. Tidsskrift for den Norske laegeforening : tidsskrift for praktisk medicin, ny raekke. 1993;113(26):3244-7.
49. Ozguner I, Kizilgun M, Karaman A, Cavusoglu YH, Erdogan D, Karaman I, et al. Are neutrophil CD64 expression and interleukin-6 early useful markers for diagnosis of acute appendicitis? European journal of pediatric surgery : official journal of Austrian Association of Pediatric Surgery [et al] = Zeitschrift fur Kinderchirurgie. 2014;24(2):179-83.
50. Peltola H, Ahlqvist J, Rapola J, Rasanen J, Louhimo I, Saarinen M, et al. C-reactive protein compared with white blood cell count and erythrocyte sedimentation rate in the diagnosis of acute appendicitis in children. Acta chirurgica Scandinavica. 1986;152:55-8.
51. Prada-Arias M, Vazquez JL, Salgado-Barreira A, Gomez-Veiras J, Montero-Sanchez M, Fernandez-Lorenzo JR. Diagnostic accuracy of fibrinogen to differentiate appendicitis from nonspecific abdominal pain in children. The American journal of emergency medicine. 2017;35(1):66-70.
52. A. Rodríguez Sánchez de la Blanca RMP, J. Lorente Romero, A. Rivas García, P. Vázquez López, C. Míguez Navarro. [Usefulness of procalcitonin in early diagnosis of acute appendicitis]. Acta Pediatr Esp. 2014;72(7):4.
53. Salo M, Roth B, Stenstrom P, Arnbjornsson E, Ohlsson B. Urinary biomarkers in pediatric appendicitis. Pediatric surgery international. 2016;32(8):795-804.
54. Samuel M. Pediatric appendicitis score. Journal of pediatric surgery. 2002;37(6):877-81.
55. Sanchez Echaniz J, Luis Garcia M, Vazquez Ronco MA, Mintegui Raso S, Benito Fernandez J, Lopez Alvarez-Buhilla P. [Diagnostic value of reactive C protein in suspected acute appendicitis in children]. An Esp Pediatr. 1998;48(5):470-4.
56. Shommu NS, Jenne CN, Blackwood J, Martin DA, Joffe AR, Eccles R, et al. The Use of Metabolomics and Inflammatory Mediator Profiling Provides a Novel Approach to Identifying Pediatric Appendicitis in the Emergency Department. Scientific reports. 2018;8(1):4083.
57. Thompson GC, Schuh S, Gravel J, Reid S, Fitzpatrick E, Turner T, et al. Variation in the Diagnosis and Management of Appendicitis at Canadian Pediatric Hospitals. Academic emergency medicine : official journal of the Society for Academic Emergency Medicine. 2015;22(7):811-22.
58. Thompson GC, Morrison E, Ross M, Liu H, Vanderkooi OG, Eccles R. The Use of Routine Blood Cultures in Pediatric Appendicitis. Pediatr Emerg Care. 2017;33(12):e160-e3.
59. Tuncer AA, Cavus S, Balcioglu A, Silay S, Demiralp I, Calkan E, et al. Can mean platelet volume, Neutrophil-to-Lymphocyte, Lymphocyte-to-Monocyte, Platelet-to-Lymphocyte ratios be favourable predictors for the differential diagnosis of appendicitis? JPMA The Journal of the Pakistan Medical Association. 2019;69(5):647-54.
60. Turkyilmaz Z, Sonmez K, Karabulut R, Elbeg S, Moralioglu S, Demirtola A, et al. Sequential cytokine levels in the diagnosis of appendicitis. Scandinavian journal of clinical and laboratory investigation. 2006;66(8):723-31.
61. van den Broek WT, van der Ende ED, Bijnen AB, Breslau PJ, Gouma DJ. Which children could benefit from additional diagnostic tools in case of suspected appendicitis? Journal of pediatric surgery. 2004;39(4):570-4.
62. Wang LT, Prentiss KA, Simon JZ, Doody DP, Ryan DP. The use of white blood cell count and left shift in the diagnosis of appendicitis in children. Pediatr Emerg Care. 2007;23(2):69-76.
63. Wu HP, Chen CY, Kuo IT, Wu YK, Fu YC. Diagnostic values of a single serum biomarker at different time points compared with Alvarado score and imaging examinations in pediatric appendicitis. The Journal of surgical research. 2012;174(2):272-7.
64. Yap TL, Chen Y, Low WW, Ong CC, Nah SA, Jacobsen AS, et al. A new 2-step risk-stratification clinical score for suspected appendicitis in children. Journal of pediatric surgery. 2015;50(12):2051-5.
65. Yazici M, Ozkisacik S, Oztan MO, Gursoy H. Neutrophil/lymphocyte ratio in the diagnosis of childhood appendicitis. The Turkish journal of pediatrics. 2010;52(4):400-3.

66. Zouari M, Jallouli M, Louati H, Kchaou R, Chtourou R, Kotti A, et al. Predictive value of C-reactive protein, ultrasound and Alvarado score in acute appendicitis: a prospective pediatric cohort. The American journal of emergency medicine. 2016;34(2):189-92.
67. Zuniga RV, Arribas JL, Montes SP, Fernandez MN, Abad CG, Martin LG, et al. Application of Pediatric Appendicitis Score on the emergency department of a secondary level hospital. Pediatr Emerg Care. 2012;28(6):489-92.
68. Deeks JJ, Macaskill P, Irwig L. The performance of tests of publication bias and other sample size effects in systematic reviews of diagnostic test accuracy was assessed. Journal of Clinical Epidemiology. 2005;58(9):882-93.
69. Whiting PF, Rutjes AWS, Westwood ME, Mallett S, Deeks JJ, Reitsma JB, et al. QUADAS-2: A Revised Tool for the Quality Assessment of Diagnostic Accuracy Studies. Annals of Internal Medicine. 2011;155(8):529-36.
