# Supplementary figures and images for: Diagnostic accuracy of blood tests of inflammation in paediatric appendicitis: a systematic review and meta-analysis
Source: BMJ Open. 2022 Nov 2;12(11):e056854. doi: 10.1136/bmjopen-2021-056854 (PMC9639107; doi:10.1136/bmjopen-2021-056854)

WCC

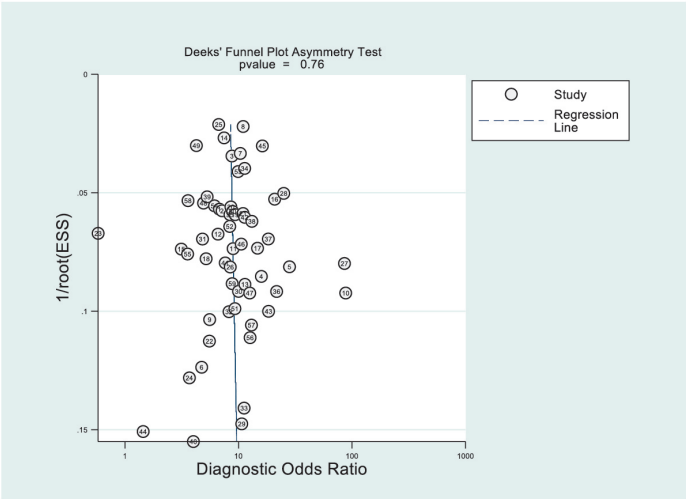

CRP

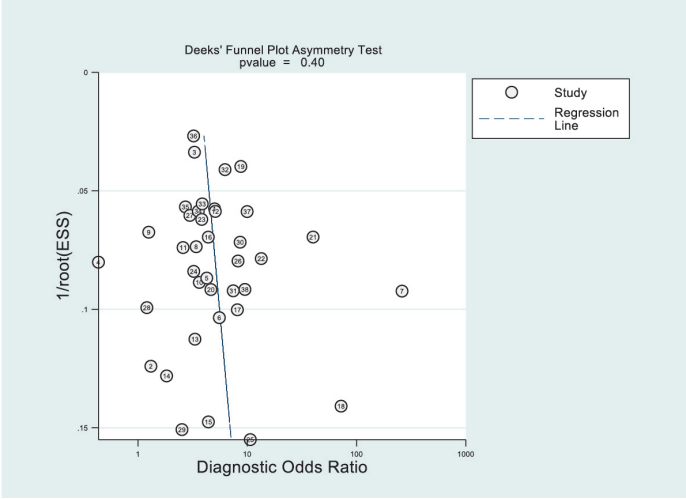

Neutrophils (%)

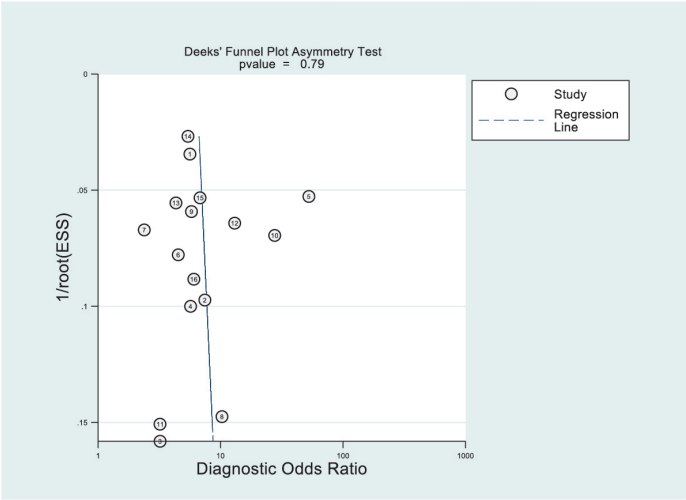

Absolute neutrophil count

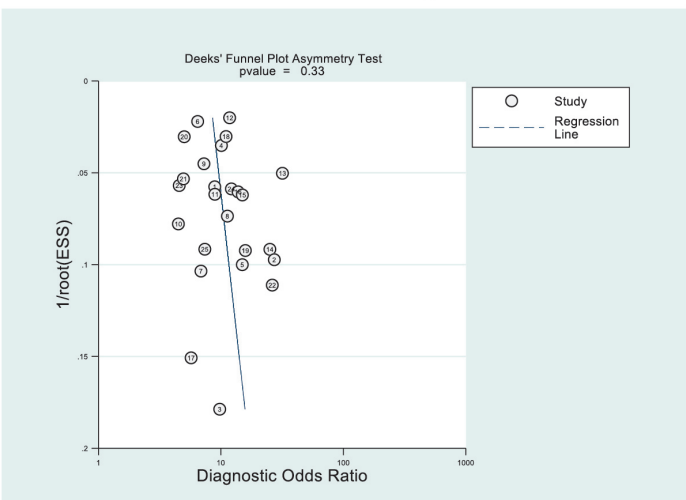

Supplement: Supplementary data [file bmjopen-2021-056854supp001.pdf]

CRP at or below 5 mg/l

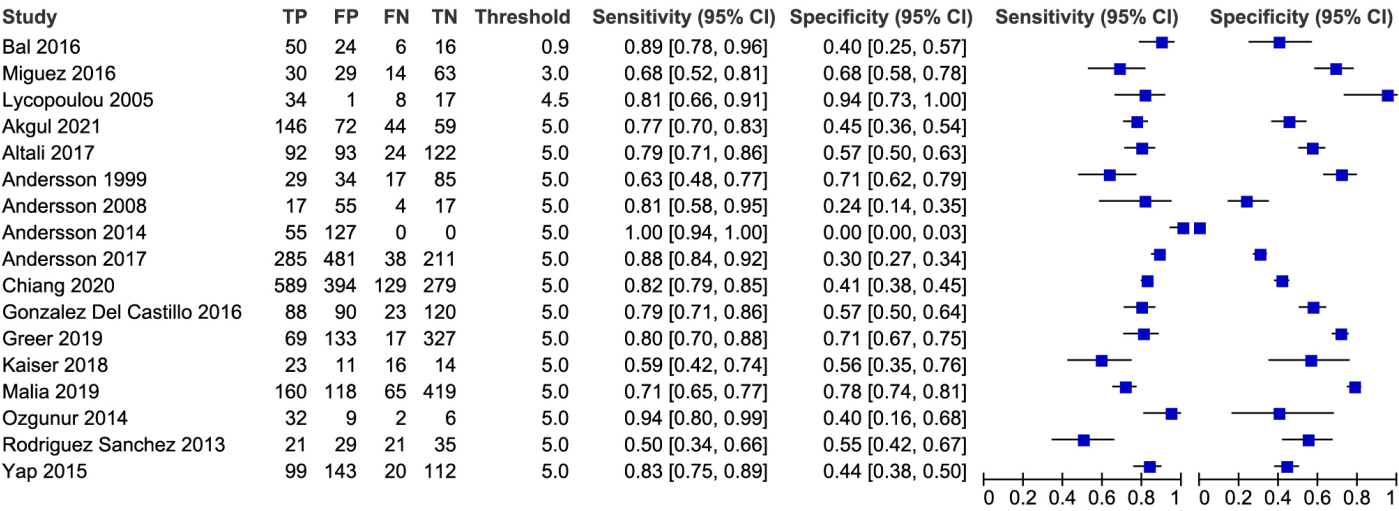

CRP between 5 and 11 mg/l

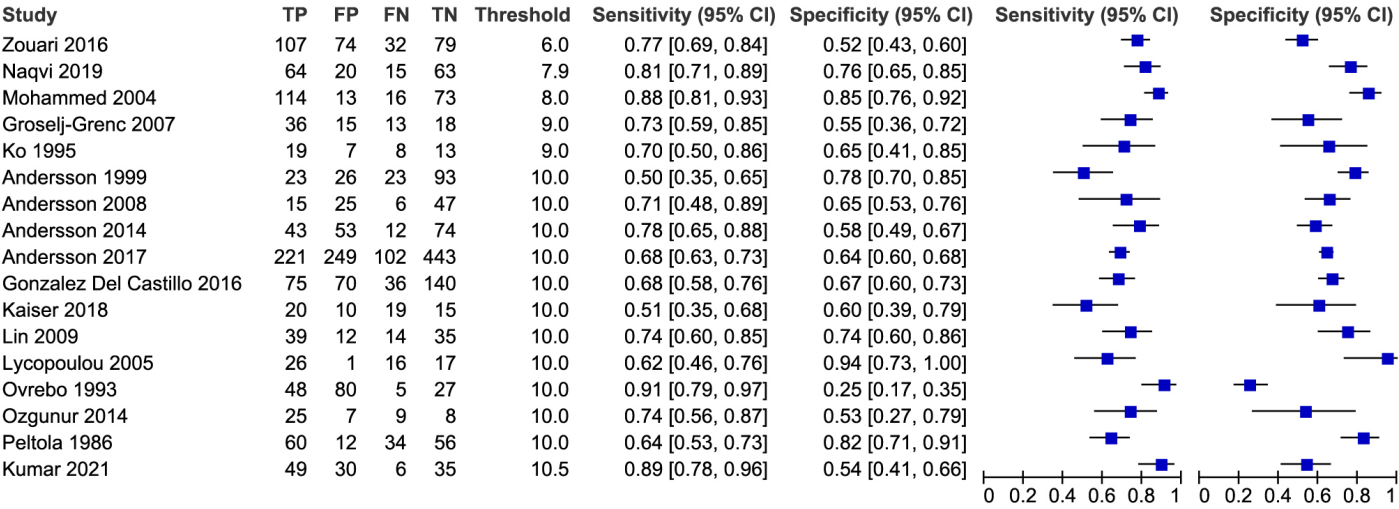

CRP between 11 and 50 mg/l

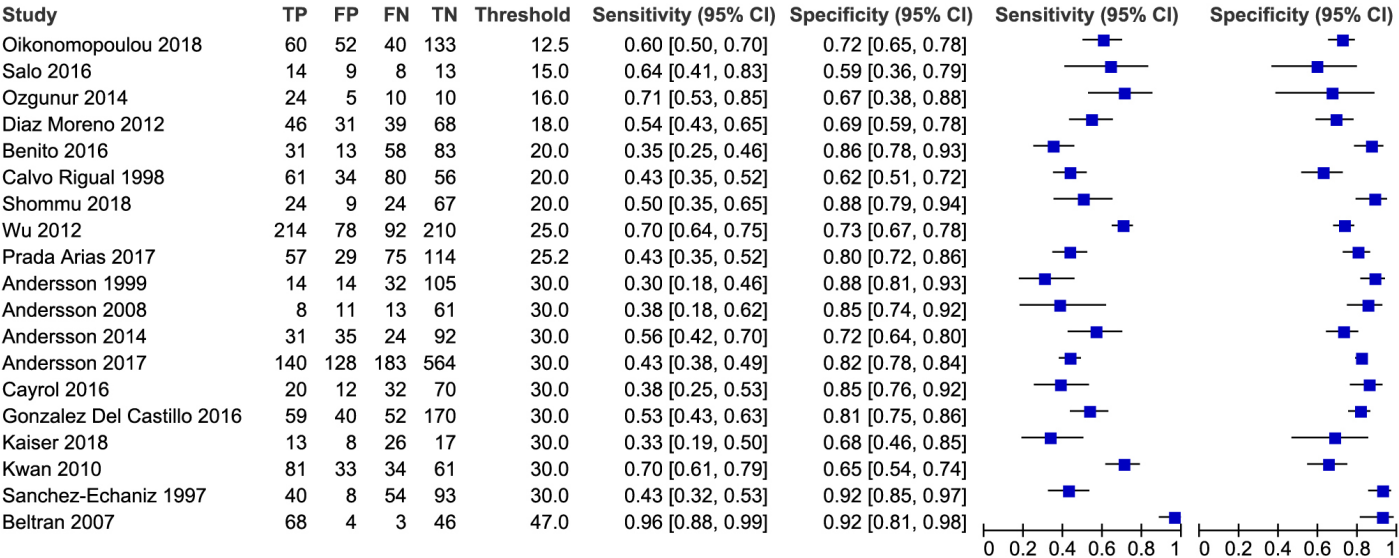

CRP at or above 50 mg/l

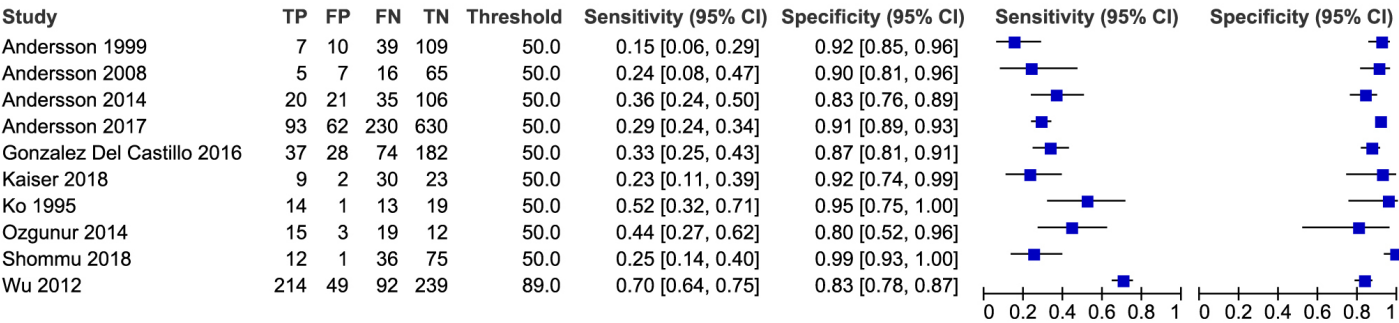

Supplement: Supplementary data [file bmjopen-2021-056854supp003.pdf]

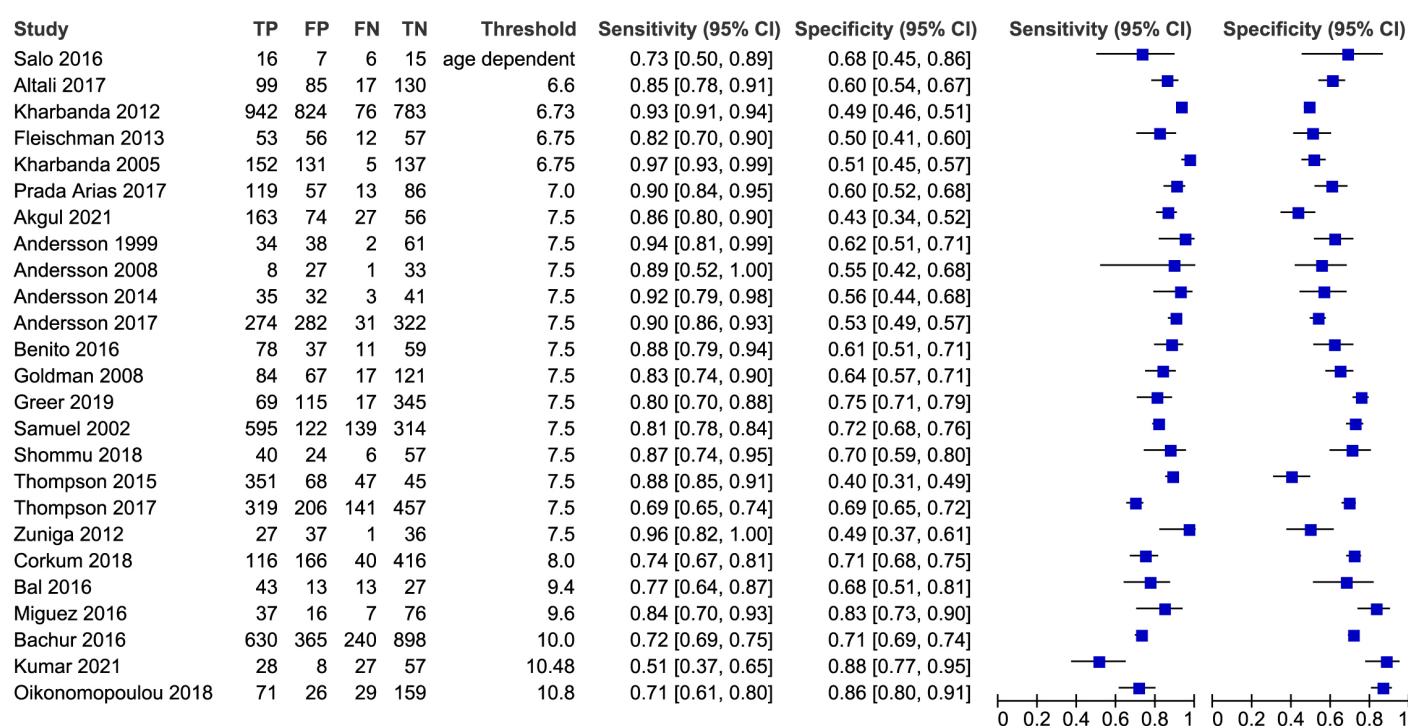

Supplement: Supplementary data [file bmjopen-2021-056854supp004.pdf]

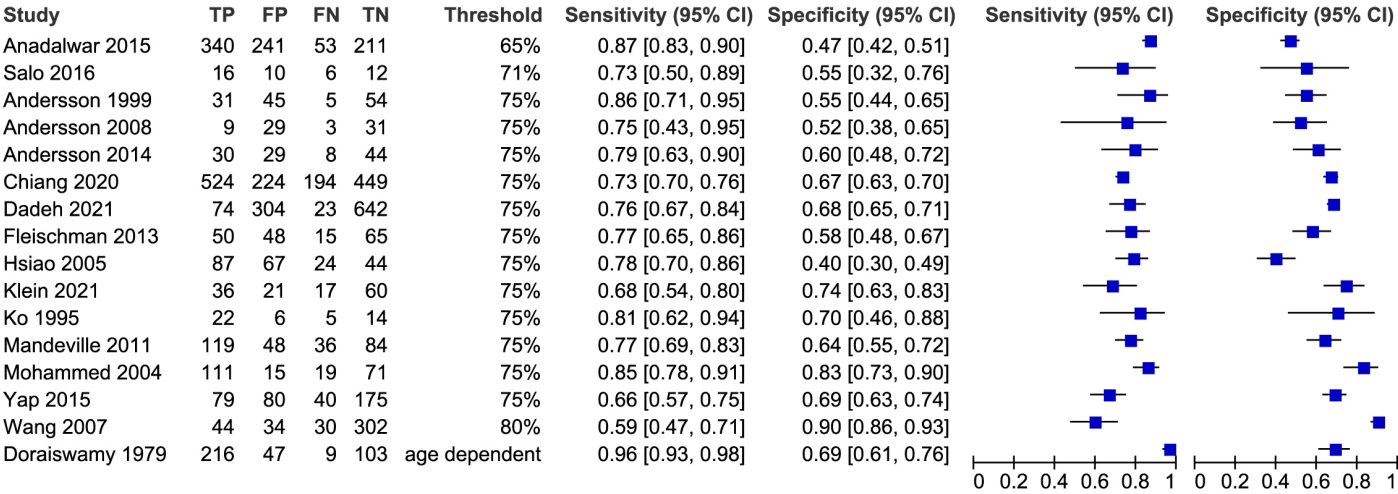

Supplement: Supplementary data [file bmjopen-2021-056854supp005.pdf]

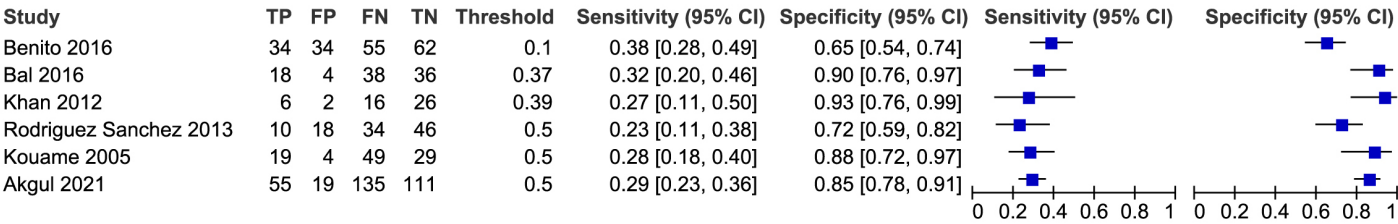

Supplement: Supplementary data [file bmjopen-2021-056854supp006.pdf]

CRP and WCC

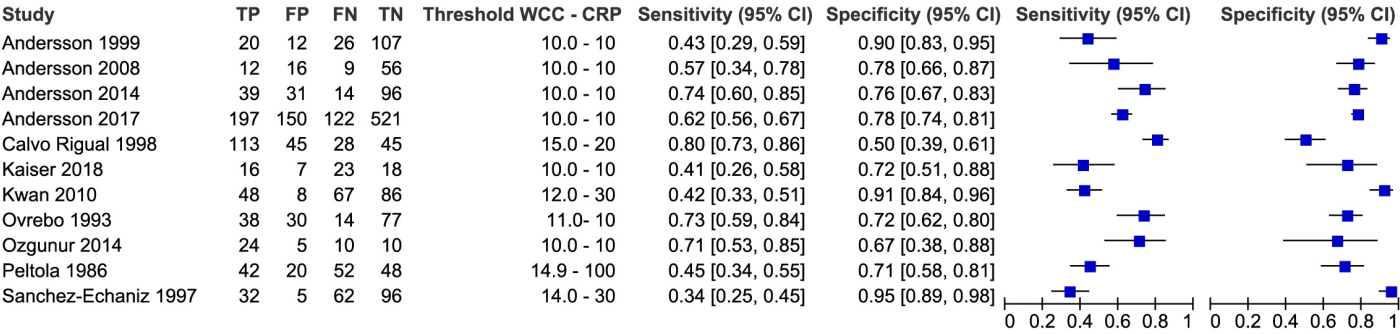

CRP or WCC

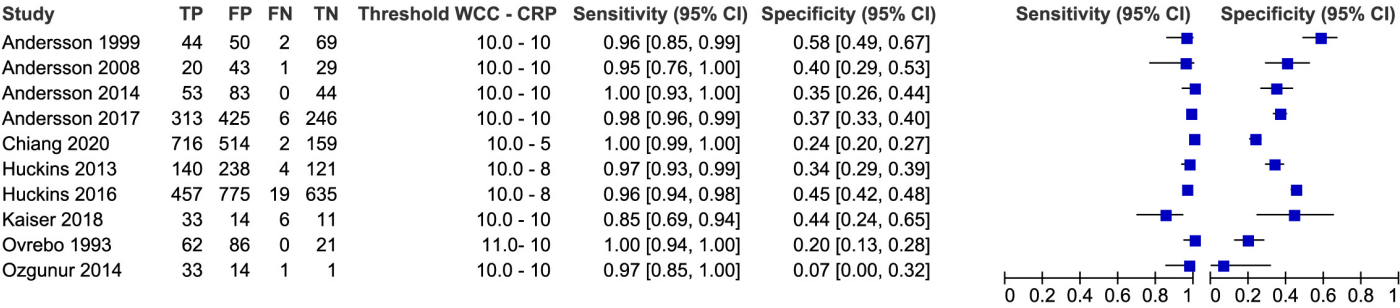

Supplement: Supplementary data [file bmjopen-2021-056854supp007.pdf]
