# Supplementary material for: Diagnostic accuracy of blood tests of inflammation in paediatric appendicitis: a systematic review and meta-analysis
Source: BMJ Open. 2022 Nov 2;12(11):e056854. doi: 10.1136/bmjopen-2021-056854 (PMC9639107; doi:10.1136/bmjopen-2021-056854)

WCC at or below 10,000 /mm^3

Study	TP	FP	FN	TN	Threshold	Sensitivity (95% CI)	Specificity (95% CI)
Kharbanda 2005	153	162	4	106	8.85	0.97 [0.94, 0.99]	0.40 [0.34, 0.46]
Kharbanda 2012	58	67	0	49	8.85	1.00 [0.94, 1.00]	0.42 [0.33, 0.52]
Anadalwar 2015	348	212	45	240	9.0	0.89 [0.85, 0.92]	0.53 [0.48, 0.58]
Kashtan 2020	857	681	117	622	9.0	0.88 [0.86, 0.90]	0.48 [0.45, 0.50]
Akgul 2021	173	78	17	52	10.0	0.91 [0.86, 0.95]	0.40 [0.32, 0.49]
Andersson 1999	42	37	6	84	10.0	0.88 [0.75, 0.95]	0.69 [0.60, 0.77]
Andersson 2008	17	35	4	39	10.0	0.81 [0.58, 0.95]	0.53 [0.41, 0.64]
Andersson 2014	52	61	2	66	10.0	0.96 [0.87, 1.00]	0.52 [0.43, 0.61]
Andersson 2017	303	339	30	348	10.0	0.91 [0.87, 0.94]	0.51 [0.47, 0.54]
Bachur 2016	786	581	84	682	10.0	0.90 [0.88, 0.92]	0.54 [0.51, 0.57]
Benito 2016	79	45	10	51	10.0	0.89 [0.80, 0.94]	0.53 [0.43, 0.63]
Cayrol 2016	46	33	6	49	10.0	0.88 [0.77, 0.96]	0.60 [0.48, 0.70]
Chiang 2020	625	319	93	354	10.0	0.87 [0.84, 0.89]	0.53 [0.49, 0.56]
Esparaz 2019	51	111	5	160	10.0	0.91 [0.80, 0.97]	0.59 [0.53, 0.65]
Fleischman 2013	53	52	12	61	10.0	0.82 [0.70, 0.90]	0.54 [0.44, 0.63]
Goldman 2008	98	84	14	112	10.0	0.88 [0.80, 0.93]	0.57 [0.50, 0.64]
Gonzalez Del Castillo 2016	93	18	79	131	10.0	0.54 [0.46, 0.62]	0.88 [0.82, 0.93]
Kaiser 2018	29	11	10	14	10.0	0.74 [0.58, 0.87]	0.56 [0.35, 0.76]
Khanafer 2016	46	51	8	75	10.0	0.85 [0.73, 0.93]	0.60 [0.50, 0.68]
Ko 1995	23	7	4	13	10.0	0.85 [0.66, 0.96]	0.65 [0.41, 0.85]
Lycopoulou 2005	32	4	10	14	10.0	0.76 [0.61, 0.88]	0.78 [0.52, 0.94]
Malia 2019	169	113	56	424	10.0	0.75 [0.69, 0.81]	0.79 [0.75, 0.82]
Mandeville 2011	129	49	26	83	10.0	0.83 [0.76, 0.89]	0.63 [0.54, 0.71]
Ozgunur 2014	32	12	2	3	10.0	0.94 [0.80, 0.99]	0.20 [0.04, 0.48]
Samuel 2002	624	113	110	323	10.0	0.85 [0.82, 0.88]	0.74 [0.70, 0.78]
Shommu 2018	41	32	5	49	10.0	0.89 [0.76, 0.96]	0.60 [0.49, 0.71]
Thompson 2015	425	56	71	46	10.0	0.86 [0.82, 0.89]	0.45 [0.35, 0.55]
Thompson 2017	334	243	137	424	10.0	0.71 [0.67, 0.75]	0.64 [0.60, 0.67]
van den Broek 2004	52	8	13	26	10.0	0.80 [0.68, 0.89]	0.76 [0.59, 0.89]
Yap 2015	102	126	17	129	10.0	0.86 [0.78, 0.91]	0.51 [0.44, 0.57]
Yazici 2010	162	39	21	18	10.0	0.89 [0.83, 0.93]	0.32 [0.20, 0.45]
Zuniga 2012	26	37	2	36	10.0	0.93 [0.76, 0.99]	0.49 [0.37, 0.61]

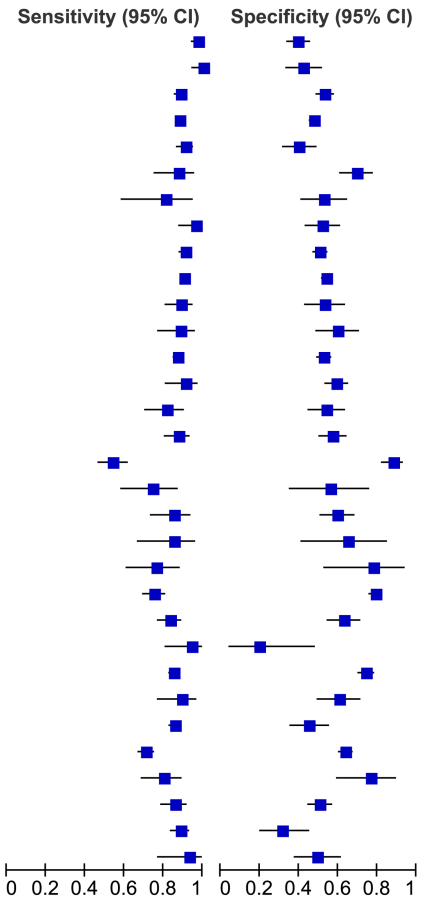

WCC above 10,000 and below 15,000 /mm^3

Study	TP	FP	FN	TN	Threshold	Sensitivity (95% CI)	Specificity (95% CI)
Altali 2017	92	75	24	140	10.5	0.79 [0.71, 0.86]	0.65 [0.58, 0.71]
Groselj-Grenc 2007	36	11	13	22	11.0	0.73 [0.59, 0.85]	0.67 [0.48, 0.82]
Hsiao 2005	86	95	25	16	11.0	0.77 [0.69, 0.85]	0.14 [0.08, 0.22]
Mohammed 2004	109	19	21	67	11.0	0.84 [0.76, 0.90]	0.78 [0.68, 0.86]
Ovrebo 1993	116	34	89	138	11.0	0.57 [0.50, 0.63]	0.80 [0.73, 0.86]
Prada Arias 2017	118	61	14	82	11.0	0.89 [0.83, 0.94]	0.57 [0.49, 0.66]
Wu 2012	245	83	61	205	11.0	0.80 [0.75, 0.84]	0.71 [0.66, 0.76]
Kaiser 2018	22	4	17	21	11.6	0.56 [0.40, 0.72]	0.84 [0.64, 0.95]
Andersson 1999	28	23	20	98	12.0	0.58 [0.43, 0.72]	0.81 [0.73, 0.88]
Andersson 2008	12	22	9	52	12.0	0.57 [0.34, 0.78]	0.70 [0.59, 0.80]
Andersson 2014	42	44	12	83	12.0	0.78 [0.64, 0.88]	0.65 [0.56, 0.74]
Andersson 2017	268	211	65	476	12.0	0.80 [0.76, 0.85]	0.69 [0.66, 0.73]
Bachur 2016	686	409	184	854	12.0	0.79 [0.76, 0.82]	0.68 [0.65, 0.70]
Gonzalez Del Castillo 2016	74	52	37	158	12.0	0.67 [0.57, 0.75]	0.75 [0.69, 0.81]
Khanafer 2016	35	34	19	92	12.0	0.65 [0.51, 0.77]	0.73 [0.64, 0.81]
Kwan 2010	82	32	33	62	12.0	0.71 [0.62, 0.79]	0.66 [0.55, 0.75]
Ozgunur 2014	29	8	5	7	12.0	0.85 [0.69, 0.95]	0.47 [0.21, 0.73]
Shommu 2018	37	21	9	60	12.0	0.80 [0.66, 0.91]	0.74 [0.63, 0.83]
Thompson 2015	380	42	116	60	12.0	0.77 [0.73, 0.80]	0.59 [0.49, 0.68]
Thompson 2017	281	161	190	506	12.0	0.60 [0.55, 0.64]	0.76 [0.72, 0.79]
Miguez 2016	35	14	9	78	12.3	0.80 [0.65, 0.90]	0.85 [0.76, 0.91]
Beltran 2007	67	8	4	42	12.5	0.94 [0.86, 0.98]	0.84 [0.71, 0.93]
Diaz Moreno 2012	59	42	26	58	12.8	0.69 [0.58, 0.79]	0.58 [0.48, 0.68]
Rodriguez Sanchez 2013	36	18	5	46	13.0	0.88 [0.74, 0.96]	0.72 [0.59, 0.82]
Tuncer 2019	104	43	33	121	13.05	0.76 [0.68, 0.83]	0.74 [0.66, 0.80]
Oikonomopoulou 2018	74	33	26	152	13.3	0.74 [0.64, 0.82]	0.82 [0.76, 0.87]
Bal 2016	38	11	18	29	13.4	0.68 [0.54, 0.80]	0.72 [0.56, 0.85]
Greer 2019	63	92	23	368	13.5	0.73 [0.63, 0.82]	0.80 [0.76, 0.84]
Kumar 2021	25	5	30	60	14.0	0.45 [0.32, 0.59]	0.92 [0.83, 0.97]
Sanchez-Echaniz 1997	73	25	21	76	14.0	0.78 [0.68, 0.86]	0.75 [0.66, 0.83]
Peltola 1986	56	11	38	57	14.9	0.60 [0.49, 0.70]	0.84 [0.73, 0.92]

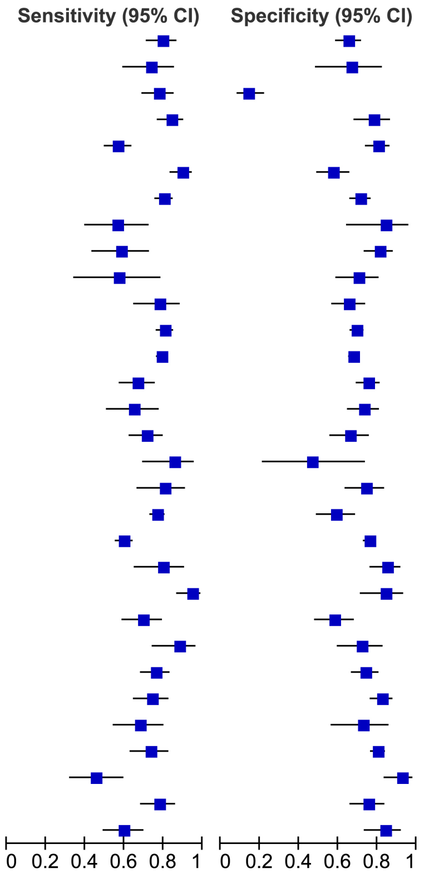

WCC at or above 15,000 /mm^3

Study	TP	FP	FN	TN	Threshold	Sensitivity (95% CI)	Specificity (95% CI)
Andersson 1999	16	5	32	116	15.0	0.33 [0.20, 0.48]	0.96 [0.91, 0.99]
Andersson 2008	9	10	12	64	15.0	0.43 [0.22, 0.66]	0.86 [0.77, 0.93]
Andersson 2014	20	17	34	110	15.0	0.37 [0.24, 0.51]	0.87 [0.79, 0.92]
Andersson 2017	159	103	174	584	15.0	0.48 [0.42, 0.53]	0.85 [0.82, 0.88]
Bachur 2016	485	240	385	1023	15.0	0.56 [0.52, 0.59]	0.81 [0.79, 0.83]
Calvo Rigual 1998	96	22	45	68	15.0	0.68 [0.60, 0.76]	0.76 [0.65, 0.84]
Gonzalez Del Castillo 2016	44	67	28	182	15.0	0.61 [0.49, 0.72]	0.73 [0.67, 0.78]
Kaiser 2018	17	3	22	22	15.0	0.44 [0.28, 0.60]	0.88 [0.69, 0.97]
Khanafer 2016	27	19	27	107	15.0	0.50 [0.36, 0.64]	0.85 [0.77, 0.91]
Lin 2009	23	4	30	43	15.0	0.43 [0.30, 0.58]	0.91 [0.80, 0.98]
Ozgunur 2014	19	8	15	7	15.0	0.56 [0.38, 0.73]	0.47 [0.21, 0.73]
Shommu 2018	30	11	16	70	15.0	0.65 [0.50, 0.79]	0.86 [0.77, 0.93]
Thompson 2015	252	24	244	78	15.0	0.51 [0.46, 0.55]	0.76 [0.67, 0.84]
Thompson 2017	195	82	276	585	15.0	0.41 [0.37, 0.46]	0.88 [0.85, 0.90]

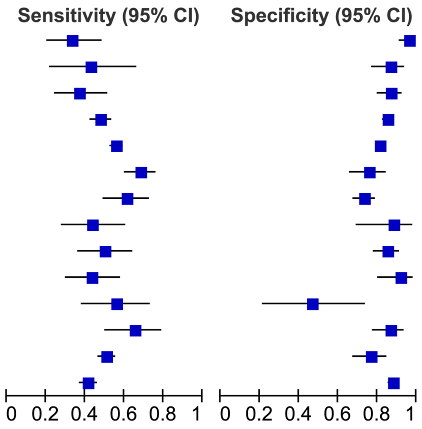

WCC threshold depending on age

Study	TP	FP	FN	TN	Threshold	Sensitivity (95% CI)	Specificity (95% CI)
Doraiswamy 1979	94	5	131	145		0.42 [0.35, 0.49]	0.97 [0.92, 0.99]
Klein 2021	40	21	13	60		0.75 [0.62, 0.86]	0.74 [0.63, 0.83]
Salo 2016	13	11	9	11		0.59 [0.36, 0.79]	0.50 [0.28, 0.72]
Turkyilmaz 2006	43	9	18	35		0.70 [0.57, 0.81]	0.80 [0.65, 0.90]
Wang 2007	50	67	24	269		0.68 [0.56, 0.78]	0.80 [0.75, 0.84]

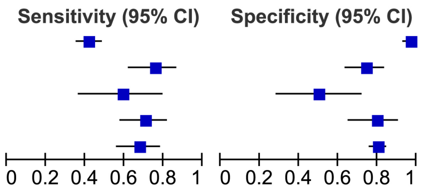

WCC threshold depending on duration pain

Study	TP	FP	FN	TN	Threshold	Sensitivity (95% CI)	Specificity (95% CI)
Dadeh 2021	73	434	24	512		0.75 [0.65, 0.83]	0.54 [0.51, 0.57]

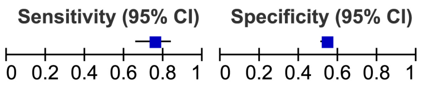

Supplement: Supplementary data [file bmjopen-2021-056854supp002.pdf]
