# Supplementary material for: Diagnostic accuracy of blood tests of inflammation in paediatric appendicitis: a systematic review and meta-analysis
Source: BMJ Open. 2022 Nov 2;12(11):e056854. doi: 10.1136/bmjopen-2021-056854 (PMC9639107; doi:10.1136/bmjopen-2021-056854)

WCC, CRP, and Neutrophils

Study	TP	FP	FN	TN	Threshold WCC - CRP - NC%	Sensitivity (95% CI)	Specificity (95% CI)
Andersson 1999	15	11	20	88	10.0 - 10 - 75%	0.43 [0.26, 0.61]	0.89 [0.81, 0.94]
Andersson 2008	6	11	6	48	10.0 - 10 - 75%	0.50 [0.21, 0.79]	0.81 [0.69, 0.90]
Andersson 2014	21	12	17	61	10.0 - 10 - 75%	0.55 [0.38, 0.71]	0.84 [0.73, 0.91]
Andersson 2017	139	89	152	498	10.0 - 10 - 75%	0.48 [0.42, 0.54]	0.85 [0.82, 0.88]
Mohammed 2004	112	8	18	78	11.0 - 80 - 75%	0.86 [0.79, 0.92]	0.91 [0.82, 0.96]

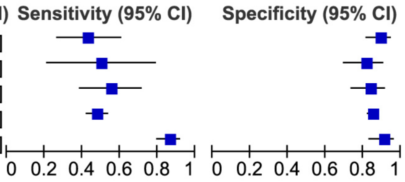

WCC, CRP, or Neutrophils

Study	TP	FP	FN	TN	Threshold WCC - CRP - NC%	Sensitivity (95% CI)	Specificity (95% CI)
Yap 2015	119	192	0	63	10.0 - 5 - 75%	1.00 [0.97, 1.00]	0.25 [0.20, 0.30]

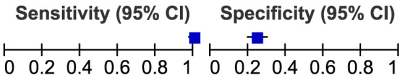

Supplement: Supplementary data [file bmjopen-2021-056854supp008.pdf]
