# Supplementary material for: Diagnostic accuracy of blood tests of inflammation in paediatric appendicitis: a systematic review and meta-analysis
Source: BMJ Open. 2022 Nov 2;12(11):e056854. doi: 10.1136/bmjopen-2021-056854 (PMC9639107; doi:10.1136/bmjopen-2021-056854)
Supplement: Author's manuscript [file bmjopen-2021-056854.draft_revisions.pdf]

BMJ Open is committed to open peer review. As part of this commitment we make the peer review history of every article we publish publicly available.

When an article is published we post the peer reviewers' comments and the authors' responses online. We also post the versions of the paper that were used during peer review. These are the versions that the peer review comments apply to.

The versions of the paper that follow are the versions that were submitted during the peer review process. They are not the versions of record or the final published versions. They should not be cited or distributed as the published version of this manuscript.

BMJ Open is an open access journal and the full, final, typeset and author-corrected version of record of the manuscript is available on our site with no access controls, subscription charges or pay-per-view fees (http://bmjopen.bmj.com).

If you have any questions on BMJ Open's open peer review process please email
info.bmjopen@bmj.com

BMJ Open

# Diagnostic accuracy of blood tests of inflammation in paediatric appendicitis – a systematic review and meta-analysis

| | |
|---|---|
| Journal: | *BMJ Open* |
| Manuscript ID | bmjopen-2021-056854 |
| Article Type: | Original research |
| Date Submitted by the Author: | 29-Aug-2021 |
| Complete List of Authors: | Fawkner-Corbett, David; Weatherall Institute of Molecular Medicine, ; University of Oxford Nuffield Department of Surgical Sciences,<br>Hayward, Gail; University of Oxford, Nuffield Department of Primary Care Health Sciences<br>Van Den Bruel, Ann; KU Leuven<br>Ordóñez-Mena, JM; University of Oxford, Nuffield Department of Primary Care Health Sciences<br>Holtman, G.; University Medical Centre Groningen, Department of General Practice and Elderly Care Medicine |
| Keywords: | PAEDIATRIC SURGERY, PAEDIATRICS, ACCIDENT & EMERGENCY MEDICINE |
| | |

SCHOLARONE™
Manuscripts

*I, the Submitting Author has the right to grant and does grant on behalf of all authors of the Work (as defined in the below author licence), an exclusive licence and/or a non-exclusive licence for contributions from authors who are: i) UK Crown employees; ii) where BMJ has agreed a CC-BY licence shall apply, and/or iii) in accordance with the terms applicable for US Federal Government officers or employees acting as part of their official duties; on a worldwide, perpetual, irrevocable, royalty-free basis to BMJ Publishing Group Ltd ("BMJ") its licensees and where the relevant Journal is co-owned by BMJ to the co-owners of the Journal, to publish the Work in this journal and any other BMJ products and to exploit all rights, as set out in our licence.*

*The Submitting Author accepts and understands that any supply made under these terms is made by BMJ to the Submitting Author unless you are acting as an employee on behalf of your employer or a postgraduate student of an affiliated institution which is paying any applicable article publishing charge ("APC") for Open Access articles. Where the Submitting Author wishes to make the Work available on an Open Access basis (and intends to pay the relevant APC), the terms of reuse of such Open Access shall be governed by a Creative Commons licence – details of these licences and which Creative Commons licence will apply to this Work are set out in our licence referred to above.*

*Other than as permitted in any relevant BMJ Author's Self Archiving Policies, I confirm this Work has not been accepted for publication elsewhere, is not being considered for publication elsewhere and does not duplicate material already published. I confirm all authors consent to publication of this Work and authorise the granting of this licence.*

For peer review only

**Title:**

Diagnostic accuracy of blood tests of inflammation in paediatric appendicitis – a systematic review and meta-analysis

**Authors names:**

David Fawkner-Corbett (0000-0002-5273-3682), Gail N Hayward (0000-0003-0852-627X), Ann Van den Bruel (000-0001-9012-2009), Jose M Ordóñez-Mena (0000-0002-8965-103X), Gea A Holtman (0000-0001-6579-767X)

**Address and positions for each author:**

NIHR Community Healthcare MedTech and IVD Co-operative, Nuffield Department of Primary Care Health Sciences, University of Oxford, Oxford OX2 6GG, United Kingdom; Academic Paediatric Surgery Unit, Nuffield Department of Surgical Sciences, University of Oxford, OX3 9DU, Oxford, United Kingdom; MRC Weatherall Institute of Molecular Medicine, Radcliffe Department of Medicine, University of Oxford, OX3 9DS, Oxford United Kingdom; David Fawkner-Corbett, DPhil doctoral training fellow

NIHR Community Healthcare MedTech and IVD Co-operative, Nuffield Department of Primary Care Health Sciences, University of Oxford, Oxford OX2 6GG, United Kingdom; Gail N Hayward, Associate professor

NIHR Community Healthcare MedTech and IVD Co-operative, Nuffield Department of Primary Care Health Sciences, University of Oxford, Oxford OX2 6GG, United Kingdom; NIHR Oxford Biomedical Research Centre, Oxford University Hospitals NHS Foundation Trust, Oxford, OX3 9DU, Oxford, United Kingdom, Jose M Ordóñez-Mena, medical statistician

EPI-Centre, Academic Centre for Primary Care, Department of Public Health and Primary Care, KU Leuven, Bus 7001, 3000 Leuven, Belgium; Ann Van den Bruel, Associate professor

NIHR Community Healthcare MedTech and IVD Co-operative, Nuffield Department of Primary Care Health Sciences, University of Oxford, Oxford OX2 6GG, United Kingdom; Department of General Practice and Elderly Care Medicine, University of Groningen, University Medical Center Groningen, PO Box 196, 9700 AD, Groningen, Netherlands, Gea A Holtman, postdoc

Correspondence to: David Fawkner-Corbett (David.fawkner-corbett@imm.ox.ac.uk)

NIHR Community Healthcare MedTech and IVD Co-operative, Nuffield Department of Primary Care Health Sciences, University of Oxford, Oxford OX2 6GG, United Kingdom

**Word count:** 4444

**Figures:** Five main figures, six supplementary

**Tables:** Two main tables, five supplementary



For peer review only - http://bmjopen.bmj.com/site/about/guidelines.xhtml

## Abstract

**Objective:** Possible appendicitis in children is a common emergency presentation. The exact value of blood tests is debated. This study sought to determine the diagnostic accuracy of four blood tests (White cell count [WCC], neutrophil [count or percentage], C-reactive protein [CRP] and/or procalcitonin) for suspected childhood appendicitis.

**Design:** Systematic review and diagnostic meta-analysis.

**Data sources:** MEDLINE, EMBASE, Central, Web of Science were searched from inception to June 2019 with reference searching and authors were contacted for missing/unclear data.

**Eligibility criteria for study selection:** Studies reported on the diagnostic accuracy of the four blood tests compared to the reference standard histology or follow-up. Risk of bias was assessed (QUADAS-2), pooled sensitivity and specificity were generated for each test and for commonly presented cut-offs. To provide insight into clinical impact, we present several strategies using a hypothetical cohort of children suspected of appendicitis.

**Results:** 60 studies were included (28,001 children, 11,252 with appendicitis), all were in the hospital setting (70% in emergency department). The most sensitive tests were WCC (≥10,000 cells/µl, 48 studies sensitivity 0·87[95% CI 0·83-0·89]) and absolute neutrophil count [ANC] (≥7,500 cells/µl, five studies sensitivity 0·90[95% CI 0·85-0·94]). Applying results to a hypothetical cohort of 1000 children presenting with symptoms of appendicitis, of whom 400 have appendicitis, 64 and 40 children would be wrongly discharged based solely on WCC and ANC respectively.
The most specific tests were CRP alone (≥50mg/l, 34 studies, specificity 0·87[95% CI 0·81-0·91]) or combined with WCC (≥10,000 cells/µl, six studies 0·93[95% CI 0·91-0·95].

**Conclusions:** The best performing blood tests for ruling-out paediatric appendicitis are WCC or ANC. With technology developing at pace these tests could be used at the point of care in combination with clinical prediction rules at the emergency department. Optimal cut-offs are provided to improve interpretation of these tests.

**Systematic review registration:** PROSPERO-CRD42017080036



For peer review only - http://bmjopen.bmj.com/site/about/guidelines.xhtml

## Strengths and limitations

- This systematic review and meta-analysis looked at the accuracy of the four most commonly used blood tests for diagnosing appendicitis against a reference standard of histology/surgical diagnosis or follow-up.
- All studies reporting children presenting with symptoms of appendicitis reporting metrics for white cell count, neutrophil (count or percentage), C-Reactive protein and/or procalcitonin were included.
- Authors were contacted when data was unclear, generating a pool of individual patient data to allow use of new methods and information about the optimal cut-offs offs for all tests.
- The literature is limited for application of results in primary care or as point of care tests, as no studies reported diagnostic accuracy in these settings.
- Results were applied to a hypothetical cohort to aid clinical interpretation.

For peer review only


For peer review only - http://bmjopen.bmj.com/site/about/guidelines.xhtml

## Introduction

Abdominal pain is a common reason for a child to attend the emergency department (ED)[1]. Differentiation between self-limiting and surgical conditions, such as acute appendicitis, can be especially difficult in children where less than 50% of patients present with classically described symptoms[2–4]. Acute appendicitis is the most common surgical condition with over 12,000 children undergoing emergency surgery for the condition each year in England[5]. It is vital to prevent an unnecessary operation in children, which is reported in 5% to 20% of cases[6–9], whilst also undertaking prompt treatment and avoiding inappropriate discharge to mitigate the risks of more severe advanced disease including perforation[10,11]. Radiological investigations including computed tomography (CT) imaging, magnetic resonance imaging (MRI) or ultrasound are useful adjunct to decide who needs surgery[7,12]. However, these modalities will often require admission and routine use in all suspected cases would increase the burden on the health-care system and children[13].

Blood tests are frequently used to investigate children with suspected appendicitis to help inform diagnostic decisions; with White cell count (WCC), neutrophils (as count or percentage) and C-reactive protein (CRP) being most widely reported[14,15]. Procalcitonin is also increasingly used to distinguish paediatric sepsis[16]. Much of the evidence for their accuracy in this setting is applied from adult studies[17,18]. All these blood tests can be available within hours and are also increasingly available rapidly as point-of-care (POC) tests thus extending their future application to expediate the clinical decision making process and broaden their use into community care[19].

Clinical scoring tools, which can incorporate clinical signs and blood results, can be used to triage patients at hospital to determine the need for imaging or surgery[20]. Although the clinical prediction scores are recommended their reported accuracy and uptake varies widely, and use is more limited in children[21]. Additionally a core scoring criteria of each is blood tests with the type of test and cut-off varying, reflecting the fact the evidence of optimum diagnostic cut off if scarce[13,22].

Taken together, blood tests are widely performed but exact accuracy for informing the diagnosis of paediatric appendicitis is debated[23]. More insight in the diagnostic accuracy of several cut offs of blood tests would help to choose which POC tests should be prioritised


For peer review only - http://bmjopen.bmj.com/site/about/guidelines.xhtml

for implementation in the emergency department, which should best inform admission or discharge decisions, and which cut-offs are optimum to integrate with future optimisation of clinical scoring tools. To date a large, systematic appraisal of the diagnostic accuracy of these tests in paediatric appendicitis is lacking and so this study aims to undertake a systematic review and meta-analysis of all studies reporting the diagnostic accuracy of common blood tests in the diagnosis of paediatric appendicitis.

For peer review only


For peer review only - http://bmjopen.bmj.com/site/about/guidelines.xhtml

## Methods

This systematic review and meta-analysis was undertaken in line with the Preferred Reporting Items for Systematic Reviews and Meta-Analyses (PRISMA) guidelines[24] and was prospectively registered (PROSPERO: CRD42017080036).

### Public and patient involvement:

The concept of this review involved discussion with patients and families before it was undertaken and discussed experience in paediatric blood tests, identifying it as important to know the exact value (Precision antimicrobial prescribing patient and public involvement group, University of Oxford). The results of this manuscript were also presented in this forum which helped formulate the write up and key discussion points within the final manuscript.

For peer review only

### Search Strategy:

Searching of databases (Cochrane Database of Systematic Reviews, Cochrane Central Register of controlled trials, Database of abstracts of reviews of effects(DARE), Embase, Ovid(Medline) Epub Ahead of print in process and other Non-Indexed Citations, Ovid Medline(R) Daily, Ovid Medline(R) and Science Citation Index and Conference Proceedings Citation Index (SCIENCE)) included key word combinations to identify children with symptoms suggestive of appendicitis having one of the four blood tests (WCC, neutrophil, CRP, procalcitonin) for diagnosis. Searching was performed on 4$^{th}$ June 2019 to include all relevant studies from database inception with no language or date limitations. Full search strategy is provided in **Supplementary Materials**. Hand searching of included studies references was undertaken. Assessment of foreign language studies was completed by a native language speaker.

### Study Selection and Data Extraction:

Included studies reported children (<18 years) presenting with symptoms suggestive of appendicitis that would have testing of WCC, neutrophil, CRP and/or procalcitonin, by laboratory or POC, to distinguish appendicitis from non-surgical cause.


For peer review only - http://bmjopen.bmj.com/site/about/guidelines.xhtml

The optimal reference standard for appendicitis was histology after surgery (ideal standard) or; surgery alone, imaging confirming appendicitis in non-operatively managed patients or surgical diagnosis in studies without histological reporting. In patients without surgery (non-appendicitis) the reference standard was follow-up after initial assessment to rule out appendicitis with an ideal standard of ≥14 days with active follow up.

Studies were included if they matched these pre-defined criteria and reported sufficient data to construct or derive a two-by-two contingency table for any/multiple blood tests. Studies were excluded if they only reported surgical cases, had <20 participants, reported atypical appendiceal pathology (e.g. carcinoid, appendiceal mass) or if the proportion of participants outside the age range was >15%. Where multiple studies reported data on the same population, only outcomes of the earliest study were used, unless the authors confirmed individual patient results.

Screening of titles and abstracts was performed independently by two reviewers (DFC and GAH). Full text articles of all short-listed studies were then assessed for eligibility.

Three reviewers (DFC, GAH, JMOM) independently extracted data from each included study on a pre-designed data extraction sheet including information on demographics, prevalence, reference standards, measurement of blood tests including cut-offs, and 2x2 tables. Disagreements were resolved by consensus or from input of an independent reviewer (GNH).

In studies which matched inclusion criteria but a. data was not available in 2x2 format; b. a paediatric sub-group was not adequately detailed; c. exclusion criteria were unclear; d. the paper was only available in an abstract form, an attempt would be made to contact an author on up to three occasions before exclusion. If contacted authors provided individual patient data, this was included.

**Assessment of study methodological quality:**

All included studies were assessed by three reviewers (DFC, GAH, JMOM) independently for risk of bias and applicability using the QUADAS-2 tool[25]. Discrepancies were resolved by discussion or review from a third independent reviewer (GNH) to achieve consensus. Full details of QUADAS-2 scoring criteria are detailed in **Supplementary Materials: QUADAS-2**. Overview graph of QUADAS-2 outcomes was created using GraphPad Prism (V 8.0, GraphPad Software Inc).


For peer review only - http://bmjopen.bmj.com/site/about/guidelines.xhtml

**Data synthesis and statistical analysis:**

We extracted binary data for all appropriate measurements of diagnostic accuracy for the pre-defined blood tests. Cut-offs were defined by authors in each individual publication. In cases where individual data was provided by authors a 2x2 table was calculated using the most commonly reported cut-offs. Data for age-dependent cut-offs would be included in study overviews and forest plots but not in cut-off specific meta-analysis. Measurement of neutrophils by means of absolute count (ANC) or percentage of white cells was analysed separately.

Test accuracy overviews for each marker were reported as forest plots including 2x2 tables, sensitivity and specificity with 95% confidence intervals using RevMan (Cochrane collaboration, Copenhagen, Denmark). Meta-analysis was performed using a bivariate random effects model and pooled estimates of sensitivity, specificity and likelihood ratios were generated when >4 studies per marker was included using the *metandi* module in STATA version 16[26–28]. When a study provided multiple cut-offs for each test, we calculated the summary receiver operating characteristics (SROC) curve with 95% CI using R *diagmeta* package developed by Steinhauser et al[29] assuming a fixed intercept (diagnostic accuracy) and slope (threshold effect) across studies. In addition, we calculated the pooled sensitivity and specificity for commonly presented cut-offs and we compared the results with the bivariate random effects model of cut-off groups of these tests.

We assessed the heterogeneity by visual inspection of the forest plot and SROC plot. Potential differences in studies with: high risk of bias in patient selection; case-control design; from primary care; with low prevalence (<10%); with high prevalence (≥60%), were evaluated by adding these covariates to the bivariate model and performing sensitivity and sub-group analysis to measure if these factors could explain heterogeneity. To assess the robustness of the diagnostic accuracy, we evaluated the effect of removing outliers. To assess for publication bias we used the Deeks' test[30].

**Potential clinical impact:**

To provide more insight into the clinical consequences of using the results of each test, for each measurement with pooled sensitivity and specificity hypothetical 2×2 tables were constructed in 1000 children presenting with symptoms suggestive of acute appendicitis.


For peer review only - http://bmjopen.bmj.com/site/about/guidelines.xhtml
For peer review only

The appendicitis prevalence was modelled on the median prevalence in the cohort studies included in the final meta-analysis. Standardisation of prevalence allowed comparison of the results of each test.

To illustrate the clinical application of tests we interpreted these as follows: children with appendicitis missed are those with appendicitis and a negative index test result (false negatives); the numbers of unnecessary surgeries are the children without appendicitis with a positive index test result (false positives); and the reduction of patients requiring surgery is the total number of patients with a negative index test result. For calculating the latter, we assumed that in the alternative strategy, all 1,000 hypothetical children would undergo surgery for appendicitis.

For peer review only



For peer review only - http://bmjopen.bmj.com/site/about/guidelines.xhtml

## Results

### Search Strategy and study selection

The search strategy, reference screening and paediatric data provided by contacted authors identified 2,652 studies. 60 studies matched inclusion criteria and were included in final analysis, of which 11 studies had patient data provided after contacting authors to clarify criteria or provide paediatric-only data[31–41]. Remaining papers were excluded with reason (**Figure 1, Supplementary Table 1**).

### Study Characteristics

All included studies were in the hospital or emergency department setting. The most common study designs were cohort study, with either a prospective (63%) or retrospective (12%) design, or case control (10%) (**Table 1, Supplementary Table 2**). No study had a low prevalence (<10%) of appendicitis. Seven cohort studies had a high prevalence (>60%) due to a more select setting such as GP referrals[42], hospitalised [39,42–44] or ITU based patients[41]. In one study the reason for high prevalence was unclear[45]. The median prevalence for all cohort studies was 40%. No study used POC blood test devices. The most frequent reference standard in appendicitis patients was histopathology with the remainder using operative reports (**Table 1, Supplementary Table 3**). Almost all studies described inclusion of a population of children with clinical symptoms of appendicitis (n=54) and seven of these studies specified a blood test and/or ultrasound for inclusion; the remaining studies (n=6) were of case-control designs and most commonly identified cases through surgery and controls as those with symptoms of appendicitis which had the condition ruled out (**Supplementary Table 2**).

### Risk of Bias

Applying the QUADAS-2 framework[25] patient selection in almost all studies (88%, 53 studies) were low risk of concern for applicability of results as they clearly included patients with suspected acute appendicitis. Those not scoring low included higher ages (n=4)[36,46–48] or only those undergoing ultrasound scans (8·3%, n=5)[49–53]. However not as many scored low for risk of bias (48%, n=29) when considering patient selection; most commonly because


For peer review only - http://bmjopen.bmj.com/site/about/guidelines.xhtml

studies did not clearly state whether they recruited a random or fully consecutive population (**Figure 2, Supplementary table 4)**.

Risk of bias and applicability of the index test and reference standard was often low risk or unclear - owing to a lack of detail regarding blinding (i.e. clinician or pathologist) or follow-up method. No study scored low for "flow and timing" as a component as this required both groups to receive the same reference standard. It is understandably not ethical to perform surgery in all children, and therefore in this dimension the seven studies that score "unclear" due to lack of detailed outcomes for all participants are likely of greater concern.

**Blood Tests**

The 60 included studies consisted of 28,001 children (11,252 with appendicitis and 16,749 without) which evaluated the following tests: WCC, CRP, ANC, neutrophils as percentage of leukocytes, procalcitonin, combined WCC and CRP, and combined WCC/CRP/neutrophil percentage. Neutrophil to lymphocyte ratio (NLR) was reported in one study (sensitivity = 0.86, specificity =0.60) and so not included in pooled analysis[54]. Similarly, the combination of WCC and ANC (>13,300/μl and >10,800/μl respectively) was reported in one study (sensitivity = 0.76, specificity =0.82)[55]. We found no evidence of publication bias for each test where this was appropriate to assess (**Supplementary Figure 1**).

An overall pooled estimate for each test, at different cut-offs, is shown in **Table 2**. For WCC and CRP the pooled estimates obtained for each cut-off with the Steinhauser method were similar for the cut-off groups using the bivariate model (**Supplemental table 5**). In subgroup analyses, the test characteristics did not differ between studies with a high appendicitis prevalence (≥60%) and lower prevalence. In some tests when using bivariate analysis the study design (for WCC 10-15,000 cells/μl) or patient selection (for WCC and CRP) had an effect on the sensitivity or specificity which we quantified (**Supplementary table 5**).

*WCC*

WCC was the most frequently reported blood test (52 studies, including 16,948 patients; 7,600 with appendicitis). At a cut-off of 10,000 cell/μl WCC exhibited one of the highest pooled sensitivities of any individual test (0.84, 95% CI 0.80-89, 28 studies) but specificity was low (0.59, 95% CI 0.52-0.66] **Table 2, Figure 3** and **4A**).


For peer review only - http://bmjopen.bmj.com/site/about/guidelines.xhtml

*CRP*

CRP was the second most frequently reported test (34 studies, 7556 patients [3152 appendicitis]. The sensitivity and specificity for lower cut-off values varied considerably between studies on visual inspection (**Figure 5**). Specificity of a CRP value of ≥50 mg/L was more homogenous. The highest pooled sensitivity of CRP was at a cut-off of ≤5mg/L (0.77 (95% CI 0.68 – 0.84)), although specificity was low (0.55, 95% CI 0.45 – 0.64**, Table 2**). Use of a cut-off ≥50mg/L had the best specificity of any single test at 0.87 (95% CI 0.81-0.91, **Table 2** and **Figure 4B**) and all studies reported a specificity of >0.8 for this (**Figure 5**).

*Neutrophil measurement*

Neutrophil measurement was reported as an absolute count (ANC) in 22 studies, including 11,996 patients (4980 appendicitis). ANC was more frequently reported and demonstrated the highest sensitivity of any test at a low cut-off (≥7,500 cells/μl, 0.90, 95% CI 0.85-0.94) **Supplementary Figure 2**, **Table 2**). However, none of the studies evaluating ANC reported multiple cut-offs and therefore the SROC curve could not be calculated using the Steinhauser method. Relatively more studies (n=11) reported a cut-off of 7,500 cells/μl which reported a similar sensitivity (0.86 (95% CI 0.82-0.90), **Table 2**) to WCC.

Fewer studies (n=13, 3316 patients; 1407 appendicitis) reported neutrophil count as a percentage (**Supplementary Figure 3, Supplementary Table 5**), the most common cut-off reported was 75% and pooled (n=9) analysis at this level showed the sensitivity to be lower than ANC and WCC (**Table 2**).

*Procalcitonin*

Procalcitonin accuracy was quantified in 5 studies (540 patients; 279 appendicitis) which led to pooled analysis being performed on varied cut-offs. Sensitivity was homogenous, while specificity varied between the studies (**Supplementary Figure 4**). Pooled procalcitonin had a relatively high pooled specificity (0.82, 95% CI 0.70-0.90) (**Table 2**).

*Combinations of tests*


For peer review only - http://bmjopen.bmj.com/site/about/guidelines.xhtml

A combination of CRP and WCC (13 studies, 4,887 patients, 1,629 appendicitis) demonstrated moderate specificity (0.78, 95% CI 0.65-0.88, **Table 2**) although cut-offs did vary between studies (**Supplementary Figure 5**).

A combination of CRP, WCC and neutrophils (six studies, 1,784 patients, 625 appendicitis) showed a reasonably good sensitivity and specificity (0.80, 95% CI 0.35-0.97 and 0.79, 95% CI 0.60-0.90 respectively, **Table 2**). Yap et al (2015) reported a very high sensitivity (1.00 95% CI 0.97-1.00) and low specificity (0.25, 95% CI 0.20-0.30) which were markedly different from the results of other studies most likely due to a lower cut off for CRP (5mg/dL, **Supplementary Figure 6**)[56]; excluding this as an outlier decreased pooled sensitivity (0.59, 95% CI 0.41 – 0.74) and increased specificity (0.86, 95% CI 0.83 – 0.89).

To account for pooling of differing cut-offs in combinations of tests we performed a sub-group analysis of patients from six studies which provided individual patient data (IPD) on request for clarification[31–35,41]. This included the raw results for 1541 patients and showed that a combination of WCC >10,000 cell/μl and CRP>10mg/L has reasonable specificity (0.79, 95% CI 0.74-0.84) which was increased when the threshold for CRP was raised to >50mg/L with WCC (0.93, 95% CI 0.91-0.95), with a resulting low sensitivity (0.27, 95% CI 0.19-0.37, **Supplementary Table 5**).

*Clinical impact*

Modelling the effect of each test upon a hypothetical cohort of 1,000 children with a 40% prevalence of appendicitis, which was the median prevalence of all included studies, showed that the tests performing best for ruling out appendicitis were ANC <7,500 cells/μl and WCC ≥10,000cells/μl, as these would exclude acute appendicitis in 364 and 418 children and miss 40 and 64 of the 400 children with appendicitis respectively (**Table 2, Supplementary Table 5**). Therefore, in clinical application these tests would be the best for supporting a decision to discharge, or to not refer a patient for surgical review.

The best single test for ruling in appendicitis was a CRP value ≥50mg/L and so could be the most useful in deciding to rule in the diagnosis of appendicitis. Although in the hypothetical cohort 78 would still undergo unnecessary treatment (False positive) of the 202 that would meet this threshold. Also, 276 children with appendicitis would be missed (**Table 2**). Using IPD identified a combination of WCC >10,000 cells/μl and CRP >50mg/L as more specific,


For peer review only - http://bmjopen.bmj.com/site/about/guidelines.xhtml

application of this to inform surgical decision would result in the lowest number of unnecessary operations (n=46) but only 150 patients would meet this threshold in the hypothetical cohort, 104 of which would have appendicitis (**Supplementary table 5**). Although no included studies were in the community setting, the analysis was repeated with a lower prevalence (20%) to reflect metrics for use of blood tests in a non-emergency department setting, or if it was used for a screening test to decide other diagnostic modalities such as ultrasound investigation (**Supplementary table 6**).

For peer review only


For peer review only - http://bmjopen.bmj.com/site/about/guidelines.xhtml

## Discussion

In a pooled analysis of 28,001 children attending hospital with symptoms of appendicitis the most sensitive blood tests for discriminating appendicitis from non-surgical conditions were WCC or ANC, and the most specific were CRP with or without WCC. However, if these were used as a sole decision-making tool in a hypothetical cohort of 1000 children with 40% appendicitis prevalence, even the most sensitive test if used to support a clinicians decision to discharge a patient would result in 64 cases of missed appendicitis (WCC). Conversely, although a blood test alone would unlikely be used as the sole tool for surgical decision making, if a blood test was used to rule in the condition the most specific test would result in 46 unnecessary treatments (in the case of WCC and CRP). Therefore, all common blood tests have limitations in the diagnosis of paediatric appendicitis which we accurately quantify. We highlight which tests should be valued most highly in more complex diagnostic pathways.

*Strengths*

To our knowledge, this is the largest systematic review of the diagnostic accuracy of commonly utilised blood tests in children with suspected appendicitis. Through use of a broad search strategy we combined information from 60 studies and report pooled measurements for four common blood tests at multiple cut-offs and in combination. Our analysis used multiple models for quantification and also undertook analysis for potential sub-group and publication biases. Previous diagnostic accuracy meta-analysis methods have used diagnostic data for a single cut-off, and often the selected cut-off is the one maximizing the diagnostic accuracy parameters. Thus, by using our approach, we ensure the pooled diagnostic accuracy parameters are not overestimated[29]. The study gained important additional data through contacting authors and utilised individual patient data where possible, adding to the applicability of results into clinical practice. The included studies had representation from a variety of continents and 65% were undertaken in the last nine years adding to the applicability of results. In addition, 93% of the studies confirmed the diagnosis of acute appendicitis with histopathology results adding to the reliability of findings.


For peer review only - http://bmjopen.bmj.com/site/about/guidelines.xhtml

*Potential limitations*

An important limitation is no studies were identified from the primary care settings, or reported results of POC devices. Our findings could be readily applicable to the emergency setting for use in triage, similar to other conditions[57]. We included no studies with very low (<10%) prevalence, which could feasibly be seen in primary care. Although the sensitivity analysis showed that prevalence did not affect the diagnostic accuracy, these results should be interpreted with care in community which represents a gap in current knowledge. We repeated the cohort analysis in a lower prevalence setting (20%) to illustrate if blood tests were used in the community or as a screening test for imaging investigation, although metrics did reflect information from higher prevalence studies.

Additionally, pooled metrics were applied to a hypothetical cohort which aids in the application but would not wholly represent the clinical setting, where full decision making would be more complex and could utilise other diagnostic modalities such as imaging. Also, although these results give one of the largest pooled analysis of blood tests the strategy is not able to identify the added value of these to specific signs and symptoms. However, our data will give insight into the best tests and optimal cut offs to measure this combination which could be used for future work.

*Comparison with literature*

Smaller systematic reviews of adults and children have reported different metrics for diagnostic accuracy, such as WCC sensitivity being 0.62-0.87[18,58,59], which is likely a result of pooling multiple cut-offs from studies. To mitigate this effect we have pooled by similar cut-off generating SROC curves for each or from IPD where possible. This should increase the reliability and applicability of results, and highlighted that at a specific cut-off WCC sensitivity was higher than this (0.84 at 10,000cells/μl).

Other studies report combined clinical scoring tools for diagnosing appendicitis, with blood tests as one component. A recent review reported the best performing of these at a low cut-off to miss 18 out of 539 cases of paediatric appendicitis if used[20]. The specificity of this was lower than any of our measured blood tests however (44.3% 95% CI 41.4-47.2%) and the included scoring tools attempted to generate a scoring tool using blood results as continuous values alongside clinical findings. These tools have the advantage of


For peer review only - http://bmjopen.bmj.com/site/about/guidelines.xhtml

incorporating clinical signs and symptoms, and our results complement these by quantifying the exact benefit of tests at different cut-offs.

Blood tests in paediatric appendicitis have also been reviewed as part of a screen of other diagnostic modalities, with WCC identified as sensitive at 10,000 cells/μl (0.88% 95% CI 87-90%) and CRP as specific (no pooled analysis)[60]. This did not include retrospective or non-ED studies, being limited to single studies at some cut offs. Our study seems to agree with this but included more studies and IPD where available. This allowed reporting of different cut-offs in pooled analyses and highlighted the benefit of combined tests (e.g. WCC and CRP).

*Clinical implications*

Applying the meta-analysis results to a hypothetical clinical cohort demonstrated that ANC, WCC and CRP are the tests with greatest clinical value. Although in practice clinicians may not rely on blood tests alone, it is still important to know ANC or WCC at a low cut off would be the best tests to inform a decision to discharge. Using this strategy for triage would result in a relatively low number of missed appendicitis cases (64 and 40 out of the 400 cases in the modelled cohort respectively). These values could be used to develop triage pathways, or equate risk to parents. These results could provide the basis for application of selected blood tests in a prospective trial in the triage setting, or an evaluation of POC tests outside the emergency department[61].

CRP at a high cut off or combined with WCC was the most specific test but a significant proportion of children would still reach this threshold, and a large number of true cases would be missed (276 and 292 respectively). A recent international cohort study of over 1800 children with right iliac fossa pain, identified a negative appendicectomy rate of 15.9%, increasing to 22.4% in girls aged 11-15 years[20]. Our results suggest using those blood tests alone to inform surgery would perform worse than this, highlighting blood tests alone should not be used to rule in appendicitis.

Application of these results, especially in the situation of combined blood tests in addition to signs and symptoms, would leave a proportion of children between the two clinical situations of a positive low WCC/ANC, and high CRP. In these children where diagnostic uncertainty remains, further assessment by specialist or use of an adjunct such as ultrasound could be utilised. Using specific tests in combination with prediction rules to


For peer review only - http://bmjopen.bmj.com/site/about/guidelines.xhtml

determine the need for investigations such as ultrasound could also lead to an increased diagnostic accuracy of imaging, being higher when the pre-test probability increases[62]. Our results would not support using procalcitonin or neutrophil percentage as a preferred test for clinical decision making[63].

*Conclusion*

In conclusion, we report the largest study of commonly available blood tests and identify WCC value below 10,000 cells/µl and ANC value below 7,500 cells/µl are the best test in ruling-out suspected childhood appendicitis. These tests could be used in addition to signs and symptoms to discharge patients. A CRP value above 50mg/l in addition to signs and symptoms could be used to select patients for further imaging. However, more information about the exact added value of these blood tests incorporated with other diagnostic modalities is needed. Although we did not include primary care studies or studies evaluating POC tests, these three tests could be prioritised for future evaluation.

For peer review only


For peer review only - http://bmjopen.bmj.com/site/about/guidelines.xhtml

**Figure Legends:**

**Figure 1:** PRISMA flow chart for identification of included studies (*some studies excluded for 2 reasons, see full overview of excluded studies in **Supplementary table 1**)[24]

**Figure 2:** Overview of QUADAS-2 assessment of all included studies (n=60) showing review authors' assessment of risk of bias and study applicability in each domain reported as a percentage of all included studies (proportion coloured by assessment as high [red], unclear [yellow] or low [blue], full outline of all individual components of QUADAS-2 is detailed in **Supplementary table 4**).

**Figure 3:** Forest plot showing all studies reporting diagnostic accuracy of WCC in paediatric appendicitis, grouped by reference range reported or obtained from study. (Sensitivity and specificity for each study represented visually (blue square) with 95% confidence interval (line)).

**Figure 4:** Summary receiver operating characteristic plot of WCC **(A)** and CRP **(B)** for diagnosis of paediatric acute appendicitis (Colour of dot representing study identified in legend, hashed grey box = 95% confidence interval specificity, solid grey box = 95% confidence interval sensitivity. Examples of commonly reported cut-offs shown in **(A)** as blue [10,000/µl], green [12,000/ µl] and red [15,000/ µl] crosses on curve; in **(B)** as yellow [5ml/L], pink [10mg/L], blue [20mg/L], green [30mg/L] and red [50mg/L] crosses on curve)

**Figure 5:** Forest plot showing all studies reporting diagnostic accuracy of CRP in paediatric appendicitis, grouped by reference range reported or obtained from study. (Sensitivity and specificity for each study represented visually (blue square) with 95% confidence interval (line)).

For peer review only


For peer review only - http://bmjopen.bmj.com/site/about/guidelines.xhtml

**Contribution statement:** DFC, GNH, AvdB and GAH conceived and designed the study. DFC and GAH assessed initial search results and selected studies for inclusion with discrepancies resolved with GNH and AvdB. DFC, GNH and JMOM performed data extraction. DFC and GAH performed analysis. All authors were involved in formation and revision of final manuscript. The corresponding author attests that all listed authors meet authorship criteria and that no others meeting the criteria have been omitted.

**Competing interest**

No authors declare any competing interests.

**Funding statement:**

DFC received funding from the National Institute for Health Research as an Academic Clinical Fellow and the Wellcome Trust Doctoral Training Fellowship at the University of Oxford. GAH received funding by Ter Meulen Grant of the Royal Netherlands Academy of Arts and Sciences, and NWO talent programme Veni

**Acknowledgements**: We thank Nia Roberts and the Bodleian healthcare libraries for assistance in conducting the search strategy. We acknowledge help in the review of foreign language studies provided by Patrick Garfjeld Roberts, University of Oxford. We appreciate those authors that provided patient data upon request to allow inclusion of their original studies

**Ethics approval statement:** Study design and methodology was prospectively registered (PROSPERO-CRD42017080036). Study design used analysis of published data which did not involve participants and so did not require institutional or research committee ethical approvals.



For peer review only - http://bmjopen.bmj.com/site/about/guidelines.xhtml

## References


1. Armon, K. *et al.* Determining the common medical presenting problems to an accident and emergency department. *Arch. Dis. Child.* **84**, 390–392 (2001).
2. Baxter, K. J., Nguyen, H. T. M. H., Wulkan, M. L. & Raval, M. V. Association of Health Care Utilization With Rates of Perforated Appendicitis in Children 18 Years or Younger. *JAMA Surg.* **153**, 544–550 (2018).
3. Rothrock, S. G., Skeoch, G., Rush, J. J. & Johnson, N. E. Clinical features of misdiagnosed appendicitis in children. *Ann. Emerg. Med.* **20**, 45–50 (1991).
4. Rentea, R. M. & St. Peter, S. D. Pediatric Appendicitis. *Surg. Clin. North Am.* **97**, 93–112 (2017).
5. Statistics, H. E. Hospital Episode Statistics (HES), Admitted Patient Care, England 2012-2013. *Hospital Episode Statistics (HES), Admitted Patient Care, England 2012-2013 [Report].* content.digital.nhs.uk/catalogue/PUB12566/hosp-epis-stat-admi-proc-2012-13-%0Dtab.xlsx (2012).
6. Wagner, J. M., McKinney, W. P. & Carpenter, J. L. Does This Patient Have Appendicitis? *JAMA* **276**, 1589–1594 (1996).
7. Tseng, J., Cohen, T., Melo, N. & Alban, R. F. Imaging utilization affects negative appendectomy rates in appendicitis: An ACS-NSQIP study. *Am. J. Surg.* **217**, 1094–1098 (2019).
8. Macco, S., Vrouenraets, B. C. & de Castro, S. M. M. Evaluation of scoring systems in predicting acute appendicitis in children. *Surgery* **160**, 1599–1604 (2016).
9. van Rossem, C. C. *et al.* Diagnosing acute appendicitis: surgery or imaging? *Color. Dis.* **18**, 1129–1132 (2016).
10. Lewis, S. R. R., Mahony, P. J. & Simpson, J. Appendicitis. *BMJ* **343**, (2011).
11. Meltzer, J. A. *et al.* Association of Delay in Appendectomy With Perforation in Children With Appendicitis. *Pediatr. Emerg. Care* **35**, 45–49 (2019).
12. Sola, R. *et al.* Standardized reporting of appendicitis-related findings improves reliability of ultrasound in diagnosing appendicitis in children. *J. Pediatr. Surg.* **53**, 984–987 (2018).
13. Di Saverio, S. *et al.* WSES Jerusalem guidelines for diagnosis and treatment of acute appendicitis. *World J. Emerg. Surg.* **11**, 1–25 (2016).
14. Andersson, R. E. B. Meta-analysis of the clinical and laboratory diagnosis of appendicitis. *Br. J. Surg.* **91**, 28–37 (2003).
15. Kwan, K. Y. & Nager, A. L. Diagnosing pediatric appendicitis: usefulness of laboratory markers. *Am. J. Emerg. Med.* **28**, 1009–1015 (2010).
16. Waterfield, T., Maney, J. A., Hanna, M., Fairley, D. & Shields, M. D. Point-of-care testing for procalcitonin in identifying bacterial infections in young infants: A diagnostic accuracy study. *BMC Pediatr.* **18**, 1–6 (2018).
17. Snyder, B. K. & Hayden, S. R. Accuracy of Leukocyte Count in the Diagnosis of Acute Appendicitis. *Ann. Emerg. Med.* **33**, 565–574 (1999).
18. Yu, C. W. *et al.* Systematic review and meta-analysis of the diagnostic accuracy of procalcitonin, C-reactive protein and white blood cell count for suspected acute appendicitis. *Br. J. Surg.* **100**, 322–329 (2013).
19. Ordóñez-Mena, J. M. *et al.* C-reactive protein and neutrophil count laboratory test requests from primary care: What is the demand and would substitution by point-of-care technology be viable? *J. Clin. Pathol.* **72**, 474–481 (2019).


For peer review only



For peer review only - http://bmjopen.bmj.com/site/about/guidelines.xhtml

20. Nepogodiev, D. *et al.* Appendicitis risk prediction models in children presenting with right iliac fossa pain (RIFT study): a prospective, multicentre validation study. *Lancet Child Adolesc. Heal.* **4**, 271–280 (2020).
21. Pogorelić, Z., Rak, S., Mrklić, I. & Jurić, I. Prospective validation of Alvarado score and pediatric appendicitis score for the diagnosis of acute appendicitis in children. *Pediatr. Emerg. Care* **31**, 164–168 (2015).
22. Gorter, R. R. *et al.* Diagnosis and management of acute appendicitis. EAES consensus development conference 2015. *Surg. Endosc. 2016 3011* **30**, 4668–4690 (2016).
23. Humes, D. J. & Simpson, J. Acute appendicitis. *Br. Med. J.* **333**, 530–531 (2006).
24. Moher, D., Liberati, A., Tetzlaff, J. & Altman, D. G. Preferred reporting items for systematic reviews and meta-analyses: the PRISMA statement. *BMJ* **339**, 332–336 (2009).
25. Whiting, P. F. *et al.* Quadas-2: A revised tool for the quality assessment of diagnostic accuracy studies. *Ann. Intern. Med.* **155**, 529–536 (2011).
26. Reitsma, J. B. *et al.* Bivariate analysis of sensitivity and specificity produces informative summary measures in diagnostic reviews. *J. Clin. Epidemiol.* **58**, 982–990 (2005).
27. Diaz, M. Performance measures of the bivariate random effects model for meta-analyses of diagnostic accuracy. *Comput. Stat. Data Anal.* **83**, 82–90 (2015).
28. RM Harbord, P Whiting, J. S. metandi: Stata software for statistically rigorous meta-analysis of diagnostic accuracy studies — University of Bristol. https://research-information.bris.ac.uk/en/publications/metandi-stata-software-for-statistically-rigorous-meta-analysis-o.
29. Steinhauser, S., Schumacher, M. & Rücker, G. Modelling multiple thresholds in meta-analysis of diagnostic test accuracy studies. *BMC Med. Res. Methodol.* **16**, 1–15 (2016).
30. Deeks, J. J., Macaskill, P. & Irwig, L. The performance of tests of publication bias and other sample size effects in systematic reviews of diagnostic test accuracy was assessed. *J. Clin. Epidemiol.* **58**, 882–893 (2005).
31. Andersson, R. E. *et al.* Diagnostic Value of Disease History , Clinical Presentation , and Inflammatory Parameters of Appendicitis. *World J. Surg.* **23**, 133–140 (1999).
32. Andersson, M. & Andersson, R. E. The appendicitis inflammatory response score: A tool for the diagnosis of acute appendicitis that outperforms the Alvarado score. *World J. Surg.* **32**, 1843–1849 (2008).
33. Thompson, G. C. *et al.* The use of routine blood cultures in pediatric appendicitis. *Pediatr. Emerg. Care* **33**, e160–e163 (2017).
34. Andersson, M. *et al.* Can new inflammatory markers improve the diagnosis of acute appendicitis? *World J. Surg.* **38**, 2777–2783 (2014).
35. Andersson, M. *et al.* Randomized clinical trial of Appendicitis Inflammatory Response score-based management of patients with suspected appendicitis. *Br. J. Surg.* **104**, 1451–1461 (2017).
36. González Del Castillo, J. *et al.* Diagnostic accuracy of the APPY1 Test in patients aged 2-20 years with suspected acute appendicitis presenting to emergency departments. *Emerg. Med. J.* **33**, 853–859 (2016).
37. Kaiser, M. *et al.* The diagnostic value of hepcidin to predict the presence and severity of appendicitis in children. *J. Surg. Res.* **222**, 102–107 (2018).
38. Khanafer, I. *et al.* Test characteristics of common appendicitis scores with and

For peer review only


For peer review only - http://bmjopen.bmj.com/site/about/guidelines.xhtml

without laboratory investigations: A prospective observational study. *BMC Pediatr.* **16**, 1–8 (2016).
39. Ozguner, I. F. *et al.* Are neutrophil CD64 expression and interleukin-6 early useful markers for diagnosis of acute appendicitis? *Eur. J. Pediatr. Surg.* **24**, 179–183 (2014).
40. Shommu, N. S. *et al.* The Use of Metabolomics and Inflammatory Mediator Profiling Provides a Novel Approach to Identifying Pediatric Appendicitis in the Emergency Department. *Sci. Rep.* **8**, 1–11 (2018).
41. Thompson, G. C. *et al.* Variation in the Diagnosis and Management of Appendicitis at Canadian Pediatric Hospitals. *Acad. Emerg. Med.* **22**, 811–822 (2015).
42. Mohammed, A. A., Daghman, N. A., Aboud, S. M. & Oshibi, H. O. The diagnostic value of C-reactive protein, white blood cell count and neutrophil percentage in childhood appendicitis. *Saudi Med. J.* **25**, 1212–1215 (2004).
43. Calvo Rigual, F. *et al.* [The value of C-reactive protein in the diagnosis of acute appendicitis in children]. *An. Esp. Pediatr.* **48**, 376–380 (1998).
44. Kouame, D. B. *et al.* La procalcitonine peut-elle aider au diagnostic des appendicites de l'enfant ? *Ann. Chir.* **130**, 169–174 (2005).
45. Samuel, M. Pediatric appendicitis score. *J. Pediatr. Surg.* **37**, 877–881 (2002).
46. Altali, K. *et al.* Escala INFURG-SEMES para el diagnóstico de apendicitis aguda en los pacientes de 2 a 20 años atendidos en los servicios de urgencias hospitalarios. *Emergencias (St. Vicenç dels Horts)* **84**, 231–236 (2017).
47. Huckins, D. S. *et al.* Prospective validation of a biomarker panel to identify pediatric ED patients with abdominal pain who are at low risk for acute appendicitis. *Am. J. Emerg. Med.* **34**, 1373–1382 (2016).
48. Huckins, D. S. *et al.* A novel biomarker panel to rule out acute appendicitis in pediatric patients with abdominal pain. *Am. J. Emerg. Med.* **31**, 1368–1375 (2013).
49. Anandalwar, S. P. *et al.* Use of white blood cell count and polymorphonuclear leukocyte differential to improve the predictive value of ultrasound for suspected appendicitis in children. *J. Am. Coll. Surg.* **220**, 1010–1017 (2015).
50. Corkum, K. S., Oyetunji, T. A., Grabowski, J. E., Rigsby, C. K. & Lautz, T. B. Absolute neutrophil count as a diagnostic guide for the use of MRI in the workup of suspected appendicitis in children. *J. Pediatr. Surg.* **54**, 1359–1364 (2019).
51. Esparaz, J. R. *et al.* A simple algorithm to improve quality while reducing resource utilization in evaluation of suspected appendicitis in children. *Am. J. Surg.* **217**, 469–472 (2019).
52. Malia, L. *et al.* Predictors for Acute Appendicitis in Children. *Pediatr. Emerg. Care* (2019).
53. Prada-Arias, M. *et al.* Diagnostic accuracy of fibrinogen to differentiate appendicitis from nonspecific abdominal pain in children. *Am. J. Emerg. Med.* **35**, 66–70 (2017).
54. Tuncer, A. A. *et al.* Can mean platelet volume, Neutrophil-to-Lymphocyte, Lymphocyte-to-Monocyte, Platelet-to-Lymphocyte ratios be favourable predictors for the differential diagnosis of appendicitis? *J. Pak. Med. Assoc.* **69**, 647–654 (2019).
55. Oikonomopoulou, N. *et al.* Assessment of proadrenomedullin as diagnostic or prognostic biomarker of acute appendicitis in children with acute abdominal pain. *Am. J. Emerg. Med.* **37**, 1289–1294 (2019).
56. Yap, T. L. *et al.* A new 2-step risk-stratification clinical score for suspected appendicitis in children. *J. Pediatr. Surg.* **50**, 2051–2055 (2015).
57. Verbakel, J. Y. *et al.* Point-of-care C reactive protein to identify serious infection in

For peer review only


For peer review only - http://bmjopen.bmj.com/site/about/guidelines.xhtml

acutely ill children presenting to hospital: Prospective cohort study. *Arch. Dis. Child.* **103**, 420–426 (2018).
58. Acharya, A., Markar, S. R., Ni, M. & Hanna, G. B. Biomarkers of acute appendicitis: systematic review and cost–benefit trade-off analysis. *Surg. Endosc.* **31**, 1022–1031 (2017).
59. Shogilev, D. J., Duus, N., Odom, S. R. & Shapiro, N. I. Diagnosing appendicitis: Evidence-based review of the diagnostic approach in 2014. *West. J. Emerg. Med.* **15**, 859–871 (2014).
60. Benabbas, R., Hanna, M., Shah, J. & Sinert, R. Diagnostic Accuracy of History, Physical Examination, Laboratory Tests, and Point-of-care Ultrasound for Pediatric Acute Appendicitis in the Emergency Department: A Systematic Review and Meta-analysis. *Acad. Emerg. Med.* **24**, 523–551 (2017).
61. Horvath, A. R. *et al.* From biomarkers to medical tests: The changing landscape of test evaluation. *Clin. Chim. Acta* **427**, 49–57 (2014).
62. Lahaye, M. J. *et al.* Mandatory imaging cuts costs and reduces the rate of unnecessary surgeries in the diagnostic work-up of patients suspected of having appendicitis. *Eur. Radiol.* **25**, 1464–1470 (2015).
63. Gongidi, P. & Bellah, R. D. Ultrasound of the pediatric appendix. *Pediatr. Radiol.* **47**, 1091–1100 (2017).

For peer review only


For peer review only - http://bmjopen.bmj.com/site/about/guidelines.xhtml

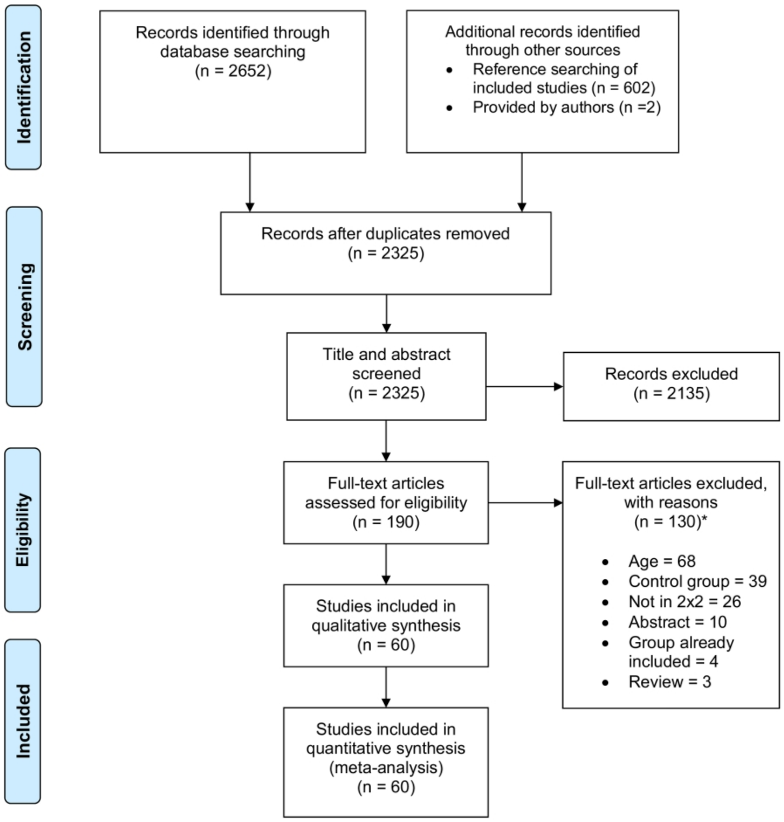


Figure 1: PRISMA flow chart for identification of included studies (*some studies excluded for 2 reasons, see full overview of excluded studies in Supplementary table 1)

215x279mm (300 x 300 DPI)

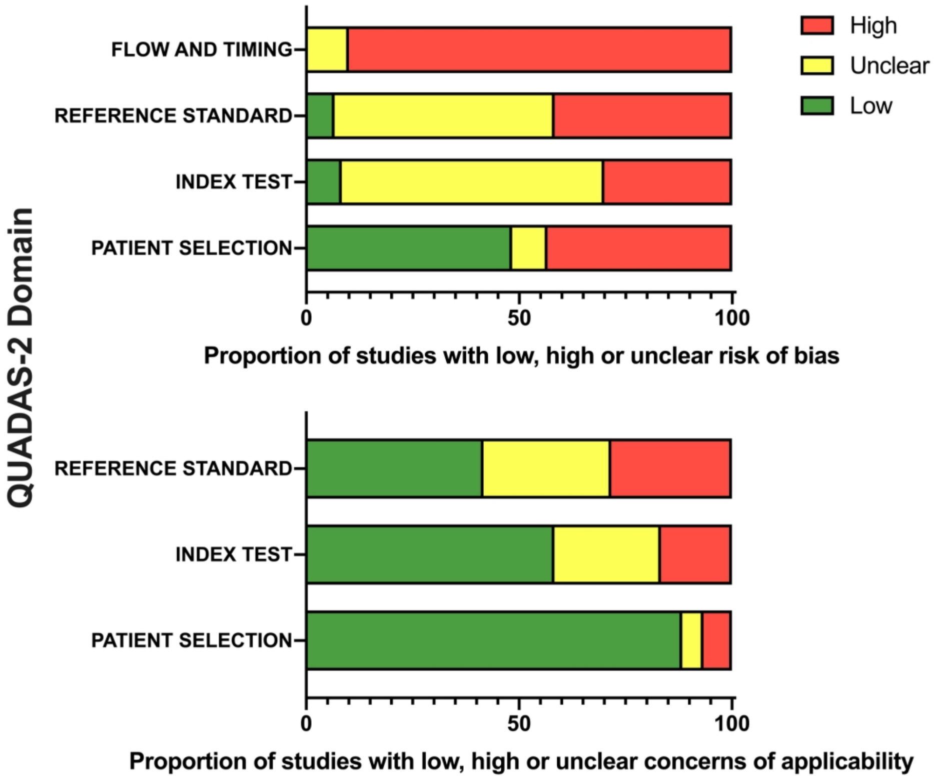


Figure 2: Overview of QUADAS-2 assessment of all included studies (n=60) showing review authors' assessment of risk of bias and study applicability in each domain reported as a percentage of all included studies (proportion coloured by assessment as high [red], unclear [yellow] or low [blue], full outline of all individual components of QUADAS-2 is detailed in Supplementary table 4).

189x159mm (300 x 300 DPI)

**WCC at or below 10,000 /mm^3**

| Study | TP | FP | FN | TN | Threshold | Sensitivity (95% CI) | Specificity (95% CI) |
|---|---|---|---|---|---|---|---|
| Kharbanda 2005 | 153 | 162 | 4 | 106 | 8.85 | 0.97 [0.94, 0.99] | 0.40 [0.34, 0.46] |
| Kharbanda 2012 | 58 | 67 | 0 | 49 | 8.85 | 1.00 [0.94, 1.00] | 0.42 [0.33, 0.52] |
| Anadalwar 2015 | 348 | 212 | 45 | 240 | 9.0 | 0.89 [0.85, 0.92] | 0.53 [0.48, 0.58] |
| Andersson 1999 | 42 | 37 | 6 | 84 | 10.0 | 0.88 [0.75, 0.95] | 0.69 [0.60, 0.77] |
| Andersson 2008 | 17 | 35 | 4 | 39 | 10.0 | 0.81 [0.58, 0.95] | 0.53 [0.41, 0.64] |
| Andersson 2014 | 52 | 61 | 2 | 66 | 10.0 | 0.96 [0.87, 1.00] | 0.52 [0.43, 0.61] |
| Andersson 2017 | 303 | 339 | 30 | 348 | 10.0 | 0.91 [0.87, 0.94] | 0.51 [0.47, 0.54] |
| Bachur 2016 | 786 | 581 | 84 | 682 | 10.0 | 0.90 [0.88, 0.92] | 0.54 [0.51, 0.57] |
| Benito 2016 | 79 | 45 | 10 | 51 | 10.0 | 0.89 [0.80, 0.94] | 0.53 [0.43, 0.63] |
| Cayrol 2016 | 46 | 33 | 6 | 49 | 10.0 | 0.88 [0.77, 0.96] | 0.60 [0.48, 0.70] |
| Esparaz 2019 | 51 | 111 | 5 | 160 | 10.0 | 0.91 [0.80, 0.97] | 0.59 [0.53, 0.65] |
| Fleischman 2013 | 53 | 52 | 12 | 61 | 10.0 | 0.82 [0.70, 0.90] | 0.54 [0.44, 0.63] |
| Goldman 2008 | 98 | 84 | 14 | 112 | 10.0 | 0.88 [0.80, 0.93] | 0.57 [0.50, 0.64] |
| Gonzalez Del Castillo 2016 | 93 | 18 | 79 | 131 | 10.0 | 0.54 [0.46, 0.62] | 0.88 [0.82, 0.93] |
| Kaiser 2018 | 29 | 11 | 10 | 14 | 10.0 | 0.74 [0.58, 0.87] | 0.56 [0.35, 0.76] |
| Khanafer 2016 | 46 | 51 | 8 | 75 | 10.0 | 0.85 [0.73, 0.93] | 0.60 [0.50, 0.68] |
| Ko 1995 | 23 | 7 | 4 | 13 | 10.0 | 0.85 [0.66, 0.96] | 0.65 [0.41, 0.85] |
| Lycopoulou 2005 | 32 | 4 | 10 | 14 | 10.0 | 0.76 [0.61, 0.88] | 0.78 [0.52, 0.94] |
| Malia 2019 | 169 | 113 | 56 | 424 | 10.0 | 0.75 [0.69, 0.81] | 0.79 [0.75, 0.82] |
| Mandeville 2011 | 129 | 49 | 26 | 83 | 10.0 | 0.83 [0.76, 0.89] | 0.63 [0.54, 0.71] |
| Ozgunur 2014 | 32 | 12 | 2 | 3 | 10.0 | 0.94 [0.80, 0.99] | 0.20 [0.04, 0.48] |
| Samuel 2002 | 624 | 113 | 110 | 323 | 10.0 | 0.85 [0.82, 0.88] | 0.74 [0.70, 0.78] |
| Shommu 2018 | 41 | 32 | 5 | 49 | 10.0 | 0.89 [0.76, 0.96] | 0.60 [0.49, 0.71] |
| Thompson 2015 | 425 | 56 | 71 | 46 | 10.0 | 0.86 [0.82, 0.89] | 0.45 [0.35, 0.55] |
| Thompson 2017 | 334 | 243 | 137 | 424 | 10.0 | 0.71 [0.67, 0.75] | 0.64 [0.60, 0.67] |
| van den Broek 2004 | 52 | 8 | 13 | 26 | 10.0 | 0.80 [0.68, 0.89] | 0.76 [0.59, 0.89] |
| Yap 2015 | 102 | 126 | 17 | 129 | 10.0 | 0.86 [0.78, 0.91] | 0.51 [0.44, 0.57] |
| Yazici 2010 | 162 | 39 | 21 | 18 | 10.0 | 0.89 [0.83, 0.93] | 0.32 [0.20, 0.45] |
| Zuniga 2012 | 26 | 37 | 2 | 36 | 10.0 | 0.93 [0.76, 0.99] | 0.49 [0.37, 0.61] |

**WCC above 10,000 and below 15,000 /mm^3**

| Study | TP | FP | FN | TN | Threshold | Sensitivity (95% CI) | Specificity (95% CI) |
|---|---|---|---|---|---|---|---|
| Altali 2017 | 92 | 75 | 24 | 140 | 10.5 | 0.79 [0.71, 0.86] | 0.65 [0.58, 0.71] |
| Groselj-Grenc 2007 | 36 | 11 | 13 | 22 | 11.0 | 0.73 [0.59, 0.85] | 0.67 [0.48, 0.82] |
| Hsiao 2005 | 86 | 95 | 25 | 16 | 11.0 | 0.77 [0.69, 0.85] | 0.14 [0.08, 0.22] |
| Mohammed 2004 | 109 | 19 | 21 | 67 | 11.0 | 0.84 [0.76, 0.90] | 0.78 [0.68, 0.86] |
| Ovrebo 1993 | 116 | 34 | 89 | 138 | 11.0 | 0.57 [0.50, 0.63] | 0.80 [0.73, 0.86] |
| Prada Arias 2017 | 118 | 61 | 14 | 82 | 11.0 | 0.89 [0.83, 0.94] | 0.57 [0.49, 0.66] |
| Wu 2012 | 245 | 83 | 61 | 205 | 11.0 | 0.80 [0.75, 0.84] | 0.71 [0.66, 0.76] |
| Kaiser 2018 | 22 | 4 | 17 | 21 | 11.6 | 0.56 [0.40, 0.72] | 0.84 [0.64, 0.95] |
| Andersson 1999 | 28 | 23 | 20 | 98 | 12.0 | 0.58 [0.43, 0.72] | 0.81 [0.73, 0.88] |
| Andersson 2008 | 12 | 22 | 9 | 52 | 12.0 | 0.57 [0.34, 0.78] | 0.70 [0.59, 0.80] |
| Andersson 2014 | 42 | 44 | 12 | 83 | 12.0 | 0.78 [0.64, 0.88] | 0.65 [0.56, 0.74] |
| Andersson 2017 | 268 | 211 | 65 | 476 | 12.0 | 0.80 [0.76, 0.85] | 0.69 [0.66, 0.73] |
| Bachur 2016 | 686 | 409 | 184 | 854 | 12.0 | 0.79 [0.76, 0.82] | 0.68 [0.65, 0.70] |
| Gonzalez Del Castillo 2016 | 74 | 52 | 37 | 158 | 12.0 | 0.67 [0.57, 0.75] | 0.75 [0.69, 0.81] |
| Khanafer 2016 | 35 | 34 | 19 | 92 | 12.0 | 0.65 [0.51, 0.77] | 0.73 [0.64, 0.81] |
| Kwan 2010 | 82 | 32 | 33 | 62 | 12.0 | 0.71 [0.62, 0.79] | 0.66 [0.55, 0.75] |
| Ozgunur 2014 | 29 | 8 | 5 | 7 | 12.0 | 0.85 [0.69, 0.95] | 0.47 [0.21, 0.73] |
| Shommu 2018 | 37 | 21 | 9 | 60 | 12.0 | 0.80 [0.66, 0.91] | 0.74 [0.63, 0.83] |
| Thompson 2015 | 380 | 42 | 116 | 60 | 12.0 | 0.77 [0.73, 0.80] | 0.59 [0.49, 0.68] |
| Thompson 2017 | 281 | 161 | 190 | 506 | 12.0 | 0.60 [0.55, 0.64] | 0.76 [0.72, 0.79] |
| Miguez 2016 | 35 | 14 | 9 | 78 | 12.3 | 0.80 [0.65, 0.90] | 0.85 [0.76, 0.91] |
| Beltran 2007 | 67 | 8 | 4 | 42 | 12.5 | 0.94 [0.86, 0.98] | 0.84 [0.71, 0.93] |
| Diaz Moreno 2012 | 59 | 42 | 26 | 58 | 12.8 | 0.69 [0.58, 0.79] | 0.58 [0.48, 0.68] |
| Rodriguez Sanchez 2013 | 36 | 18 | 5 | 46 | 13.0 | 0.88 [0.74, 0.96] | 0.72 [0.59, 0.82] |
| Tuncer 2019 | 104 | 43 | 33 | 121 | 13.05 | 0.76 [0.68, 0.83] | 0.74 [0.66, 0.80] |
| Oikonomopoulou 2018 | 74 | 33 | 26 | 152 | 13.3 | 0.74 [0.64, 0.82] | 0.82 [0.76, 0.87] |
| Bal 2016 | 38 | 11 | 18 | 29 | 13.4 | 0.68 [0.54, 0.80] | 0.72 [0.56, 0.85] |
| Sanchez-Echaniz 1997 | 73 | 25 | 21 | 76 | 14.0 | 0.78 [0.68, 0.86] | 0.75 [0.66, 0.83] |
| Peltola 1986 | 56 | 11 | 38 | 57 | 14.9 | 0.60 [0.49, 0.70] | 0.84 [0.73, 0.92] |

**WCC at or above 15,000 /mm^3**

| Study | TP | FP | FN | TN | Threshold | Sensitivity (95% CI) | Specificity (95% CI) |
|---|---|---|---|---|---|---|---|
| Andersson 1999 | 16 | 5 | 32 | 116 | 15.0 | 0.33 [0.20, 0.48] | 0.96 [0.91, 0.99] |
| Andersson 2008 | 9 | 10 | 12 | 64 | 15.0 | 0.43 [0.22, 0.66] | 0.86 [0.77, 0.93] |
| Andersson 2014 | 20 | 17 | 34 | 110 | 15.0 | 0.37 [0.24, 0.51] | 0.87 [0.79, 0.92] |
| Andersson 2017 | 159 | 103 | 174 | 584 | 15.0 | 0.48 [0.42, 0.53] | 0.85 [0.82, 0.88] |
| Bachur 2016 | 485 | 240 | 385 | 1023 | 15.0 | 0.56 [0.52, 0.59] | 0.81 [0.79, 0.83] |
| Calvo Rigual 1998 | 96 | 22 | 45 | 68 | 15.0 | 0.68 [0.60, 0.76] | 0.76 [0.65, 0.84] |
| Gonzalez Del Castillo 2016 | 44 | 67 | 28 | 182 | 15.0 | 0.61 [0.49, 0.72] | 0.73 [0.67, 0.78] |
| Kaiser 2018 | 17 | 3 | 22 | 22 | 15.0 | 0.44 [0.28, 0.60] | 0.88 [0.69, 0.97] |
| Khanafer 2016 | 27 | 19 | 27 | 107 | 15.0 | 0.50 [0.36, 0.64] | 0.85 [0.77, 0.91] |
| Lin 2009 | 23 | 4 | 30 | 43 | 15.0 | 0.43 [0.30, 0.58] | 0.91 [0.80, 0.98] |
| Ozgunur 2014 | 19 | 8 | 15 | 7 | 15.0 | 0.56 [0.38, 0.73] | 0.47 [0.21, 0.73] |
| Shommu 2018 | 30 | 11 | 16 | 70 | 15.0 | 0.65 [0.50, 0.79] | 0.86 [0.77, 0.93] |
| Thompson 2015 | 252 | 24 | 244 | 78 | 15.0 | 0.51 [0.46, 0.55] | 0.76 [0.67, 0.84] |
| Thompson 2017 | 195 | 82 | 276 | 585 | 15.0 | 0.41 [0.37, 0.46] | 0.88 [0.85, 0.90] |

**WCC threshold depending on age**

| Study | TP | FP | FN | TN | Threshold | Sensitivity (95% CI) | Specificity (95% CI) |
|---|---|---|---|---|---|---|---|
| Doraiswamy 1979 | 94 | 5 | 131 | 145 | | 0.42 [0.35, 0.49] | 0.97 [0.92, 0.99] |
| Salo 2016 | 13 | 11 | 9 | 11 | | 0.59 [0.36, 0.79] | 0.50 [0.28, 0.72] |
| Turkyilmaz 2006 | 43 | 9 | 18 | 35 | | 0.70 [0.57, 0.81] | 0.80 [0.65, 0.90] |
| Wang 2007 | 50 | 67 | 24 | 269 | | 0.68 [0.56, 0.78] | 0.80 [0.75, 0.84] |

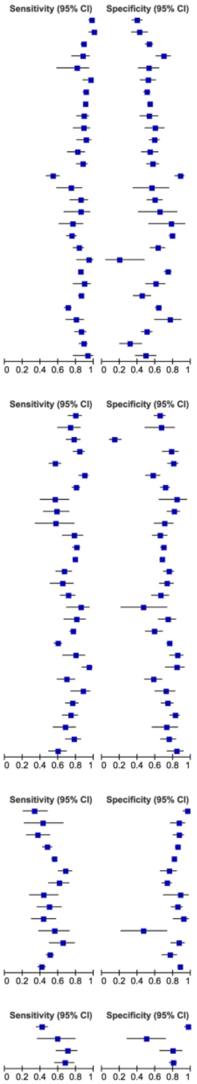


Figure 3: Forest plot showing all studies reporting diagnostic accuracy of WCC in paediatric appendicitis, grouped by reference range reported or obtained from study. (Sensitivity and specificity for each study represented visually (blue square) with 95% confidence interval (line)).

209x279mm (300 x 300 DPI)

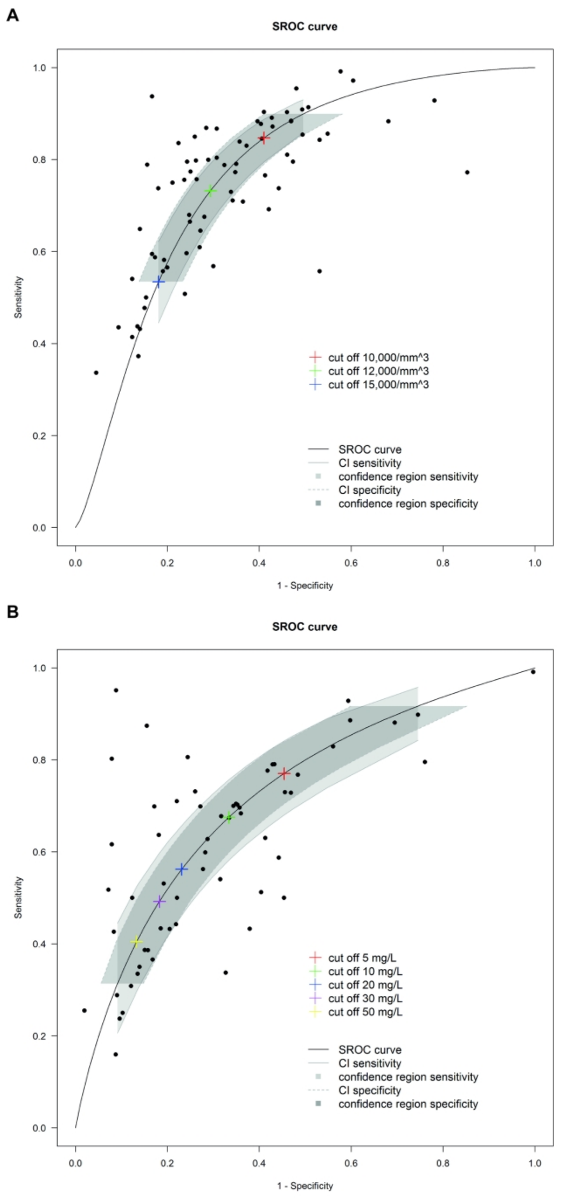


Figure 4: Summary receiver operating characteristic plot of WCC (A) and CRP (B) for diagnosis of paediatric acute appendicitis (Colour of dot representing study identified in legend, hashed grey box = 95% confidence interval specificity, solid grey box = 95% confidence interval sensitivity. Examples of commonly reported cut-offs shown in (A) as blue [10,000/μl], green [12,000/ μl] and red [15,000/ μl] crosses on curve; in (B) as yellow [5ml/L], pink [10mg/L], blue [20mg/L], green [30mg/L] and red [50mg/L] crosses on curve)

145x289mm (300 x 300 DPI)

CRP at or below 5 mg/l

| Study | TP | FP | FN | TN | Threshold | Sensitivity (95% CI) | Specificity (95% CI) |
|---|---|---|---|---|---|---|---|
| Bal 2016 | 50 | 24 | 6 | 16 | 0.9 | 0.89 [0.78, 0.96] | 0.40 [0.25, 0.57] |
| Miguez 2016 | 30 | 29 | 14 | 63 | 3.0 | 0.68 [0.52, 0.81] | 0.68 [0.58, 0.78] |
| Lycopoulou 2005 | 34 | 1 | 8 | 17 | 4.5 | 0.81 [0.66, 0.91] | 0.94 [0.73, 1.00] |
| Altali 2017 | 92 | 93 | 24 | 122 | 5.0 | 0.79 [0.71, 0.86] | 0.57 [0.50, 0.63] |
| Andersson 1999 | 29 | 34 | 17 | 85 | 5.0 | 0.63 [0.48, 0.77] | 0.71 [0.62, 0.79] |
| Andersson 2008 | 17 | 55 | 4 | 17 | 5.0 | 0.81 [0.58, 0.95] | 0.24 [0.14, 0.35] |
| Andersson 2014 | 55 | 127 | 0 | 0 | 5.0 | 1.00 [0.94, 1.00] | 0.00 [0.00, 0.03] |
| Andersson 2017 | 285 | 481 | 38 | 211 | 5.0 | 0.88 [0.84, 0.92] | 0.30 [0.27, 0.34] |
| Gonzalez Del Castillo 2016 | 88 | 90 | 23 | 120 | 5.0 | 0.79 [0.71, 0.86] | 0.57 [0.50, 0.64] |
| Kaiser 2018 | 23 | 11 | 16 | 14 | 5.0 | 0.59 [0.42, 0.74] | 0.56 [0.35, 0.76] |
| Malia 2019 | 160 | 118 | 65 | 419 | 5.0 | 0.71 [0.65, 0.77] | 0.78 [0.74, 0.81] |
| Ozgunur 2014 | 32 | 9 | 2 | 6 | 5.0 | 0.94 [0.80, 0.99] | 0.40 [0.16, 0.68] |
| Rodriguez Sanchez 2013 | 21 | 29 | 21 | 35 | 5.0 | 0.50 [0.34, 0.66] | 0.55 [0.42, 0.67] |
| Yap 2015 | 99 | 143 | 20 | 112 | 5.0 | 0.83 [0.75, 0.89] | 0.44 [0.38, 0.50] |

CRP between 5 and 11 mg/l

| Study | TP | FP | FN | TN | Threshold | Sensitivity (95% CI) | Specificity (95% CI) |
|---|---|---|---|---|---|---|---|
| Zouari 2016 | 107 | 74 | 32 | 79 | 6.0 | 0.77 [0.69, 0.84] | 0.52 [0.43, 0.60] |
| Naqvi 2019 | 64 | 20 | 15 | 63 | 7.9 | 0.81 [0.71, 0.89] | 0.76 [0.65, 0.85] |
| Mohammed 2004 | 114 | 13 | 16 | 73 | 8.0 | 0.88 [0.81, 0.93] | 0.85 [0.76, 0.92] |
| Groselj-Grenc 2007 | 36 | 15 | 13 | 18 | 9.0 | 0.73 [0.59, 0.85] | 0.55 [0.36, 0.72] |
| Ko 1995 | 19 | 7 | 8 | 13 | 9.0 | 0.70 [0.50, 0.86] | 0.65 [0.41, 0.85] |
| Andersson 1999 | 23 | 26 | 23 | 93 | 10.0 | 0.50 [0.35, 0.65] | 0.78 [0.70, 0.85] |
| Andersson 2008 | 15 | 25 | 6 | 47 | 10.0 | 0.71 [0.48, 0.89] | 0.65 [0.53, 0.76] |
| Andersson 2014 | 43 | 53 | 12 | 74 | 10.0 | 0.78 [0.65, 0.88] | 0.58 [0.49, 0.67] |
| Andersson 2017 | 221 | 249 | 102 | 443 | 10.0 | 0.68 [0.63, 0.73] | 0.64 [0.60, 0.68] |
| Gonzalez Del Castillo 2016 | 75 | 70 | 36 | 140 | 10.0 | 0.68 [0.58, 0.76] | 0.67 [0.60, 0.73] |
| Kaiser 2018 | 20 | 10 | 19 | 15 | 10.0 | 0.51 [0.35, 0.68] | 0.60 [0.39, 0.79] |
| Lin 2009 | 39 | 12 | 14 | 35 | 10.0 | 0.74 [0.60, 0.85] | 0.74 [0.60, 0.86] |
| Lycopoulou 2005 | 26 | 1 | 16 | 17 | 10.0 | 0.62 [0.46, 0.76] | 0.94 [0.73, 1.00] |
| Ovrebo 1993 | 48 | 80 | 5 | 27 | 10.0 | 0.91 [0.79, 0.97] | 0.25 [0.17, 0.35] |
| Ozgunur 2014 | 25 | 7 | 9 | 8 | 10.0 | 0.74 [0.56, 0.87] | 0.53 [0.27, 0.79] |
| Peltola 1986 | 60 | 12 | 34 | 56 | 10.0 | 0.64 [0.53, 0.73] | 0.82 [0.71, 0.91] |

CRP between 11 and 50 mg/l

| Study | TP | FP | FN | TN | Threshold | Sensitivity (95% CI) | Specificity (95% CI) |
|---|---|---|---|---|---|---|---|
| Oikonomopoulou 2018 | 60 | 52 | 40 | 133 | 12.5 | 0.60 [0.50, 0.70] | 0.72 [0.65, 0.78] |
| Salo 2016 | 14 | 9 | 8 | 13 | 15.0 | 0.64 [0.41, 0.83] | 0.59 [0.36, 0.79] |
| Ozgunur 2014 | 24 | 5 | 10 | 10 | 16.0 | 0.71 [0.53, 0.85] | 0.67 [0.38, 0.88] |
| Diaz Moreno 2012 | 46 | 31 | 39 | 68 | 18.0 | 0.54 [0.43, 0.65] | 0.69 [0.59, 0.78] |
| Benito 2016 | 31 | 13 | 58 | 83 | 20.0 | 0.35 [0.25, 0.46] | 0.86 [0.78, 0.93] |
| Calvo Rigual 1998 | 61 | 34 | 80 | 56 | 20.0 | 0.43 [0.35, 0.52] | 0.62 [0.51, 0.72] |
| Shommu 2018 | 24 | 9 | 24 | 67 | 20.0 | 0.50 [0.35, 0.65] | 0.88 [0.79, 0.94] |
| Wu 2012 | 214 | 78 | 92 | 210 | 25.0 | 0.70 [0.64, 0.75] | 0.73 [0.67, 0.78] |
| Prada Arias 2017 | 57 | 29 | 75 | 114 | 25.2 | 0.43 [0.35, 0.52] | 0.80 [0.72, 0.86] |
| Andersson 1999 | 14 | 14 | 32 | 105 | 30.0 | 0.30 [0.18, 0.46] | 0.88 [0.81, 0.93] |
| Andersson 2008 | 8 | 11 | 13 | 61 | 30.0 | 0.38 [0.18, 0.62] | 0.85 [0.74, 0.92] |
| Andersson 2014 | 31 | 35 | 24 | 92 | 30.0 | 0.56 [0.42, 0.70] | 0.72 [0.64, 0.80] |
| Andersson 2017 | 140 | 128 | 183 | 564 | 30.0 | 0.43 [0.38, 0.49] | 0.82 [0.78, 0.84] |
| Cayrol 2016 | 20 | 12 | 32 | 70 | 30.0 | 0.38 [0.25, 0.53] | 0.85 [0.76, 0.92] |
| Gonzalez Del Castillo 2016 | 59 | 40 | 52 | 170 | 30.0 | 0.53 [0.43, 0.63] | 0.81 [0.75, 0.86] |
| Kaiser 2018 | 13 | 8 | 26 | 17 | 30.0 | 0.33 [0.19, 0.50] | 0.68 [0.46, 0.85] |
| Kwan 2010 | 81 | 33 | 34 | 61 | 30.0 | 0.70 [0.61, 0.79] | 0.65 [0.54, 0.74] |
| Sanchez-Echaniz 1997 | 40 | 8 | 54 | 93 | 30.0 | 0.43 [0.32, 0.53] | 0.92 [0.85, 0.97] |
| Beltran 2007 | 68 | 4 | 3 | 46 | 47.0 | 0.96 [0.88, 0.99] | 0.92 [0.81, 0.98] |

CRP at or above 50 mg/l

| Study | TP | FP | FN | TN | Threshold | Sensitivity (95% CI) | Specificity (95% CI) |
|---|---|---|---|---|---|---|---|
| Andersson 1999 | 7 | 10 | 39 | 109 | 50.0 | 0.15 [0.06, 0.29] | 0.92 [0.85, 0.96] |
| Andersson 2008 | 5 | 7 | 16 | 65 | 50.0 | 0.24 [0.08, 0.47] | 0.90 [0.81, 0.96] |
| Andersson 2014 | 20 | 21 | 35 | 106 | 50.0 | 0.36 [0.24, 0.50] | 0.83 [0.76, 0.89] |
| Andersson 2017 | 93 | 62 | 230 | 630 | 50.0 | 0.29 [0.24, 0.34] | 0.91 [0.89, 0.93] |
| Gonzalez Del Castillo 2016 | 37 | 28 | 74 | 182 | 50.0 | 0.33 [0.25, 0.43] | 0.87 [0.81, 0.91] |
| Kaiser 2018 | 9 | 2 | 30 | 23 | 50.0 | 0.23 [0.11, 0.39] | 0.92 [0.74, 0.99] |
| Ko 1995 | 14 | 1 | 13 | 19 | 50.0 | 0.52 [0.32, 0.71] | 0.95 [0.75, 1.00] |
| Ozgunur 2014 | 15 | 3 | 19 | 12 | 50.0 | 0.44 [0.27, 0.62] | 0.80 [0.52, 0.96] |
| Shommu 2018 | 12 | 1 | 36 | 75 | 50.0 | 0.25 [0.14, 0.40] | 0.99 [0.93, 1.00] |
| Wu 2012 | 214 | 49 | 92 | 239 | 89.0 | 0.70 [0.64, 0.75] | 0.83 [0.78, 0.87] |

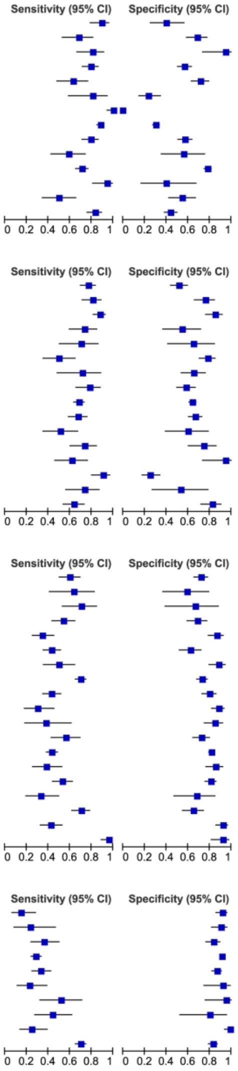


Figure 5: Forest plot showing all studies reporting diagnostic accuracy of CRP in paediatric appendicitis, grouped by reference range reported or obtained from study. (Sensitivity and specificity for each study represented visually (blue square) with 95% confidence interval (line)).

209x279mm (300 x 300 DPI)

| | | Studies (n=60) |
|---|---|---|
| **Study design** | Prospective cohort study | 38 (63%) |
| | Retrospective cohort study | 7 (12%) |
| | Case control study | 6 (10%) |
| | Prospective observational study | 2 (3%) |
| | Retrospective case series | 2 (3%) |
| | Other* | 5 (8%) |
| **Setting** | **Institution**<br>Hospital | 60 (100%) |
| | **Department**†<br>Emergency department<br>Surgical department | 42 (70%)<br>17 (28%) |
| | **Centre**<br>Single centre<br>Multiple centre | 47 (78%)<br>13 (22%) |
| **Study participants** | Total (n) | 29,008 ‡ |
| | Median patients included (range) | 258 (44-3791) |
| | <50 participants | 3 (5%) |
| | 51-250 participants | 27 (45%) |
| | 251-500 participants | 13 (22%) |
| | >500 participants | 17 (28%) |
| **Year Published** | Before 1989 | 2 (3%) |
| | 1990-1999 | 5 (8%) |
| | 2000-2009 | 14 (23%) |
| | 2010-2019 | 39 (65%) |
| **AA Prevalence** | <20% | 3 (5%) |
| | 21-35% | 16 (27%) |
| | 36-50% | 20 (33%) |
| | 51-65% | 15 (25%) |
| | >65% | 6 (10%) |
| **Gender demographics(% Male)** | <35% | 1 (2%) |
| | 36-45% | 8 (13%) |
| | 46-55% | 25 (42%) |
| | 56-65% | 19 (32%) |
| | >65% | 2 (3%) |
| | Unclear | 5 (8%) |
| **Reference standard (AA)** | Histology/ Pathology | 56 (93%) |
| | Operative findings | 4 (7%) |
| **Reference Standard (non-AA)** | Active follow up | 16 (27%) |
| | Discharge diagnosis | 15 (25%) |
| | Unspecified follow up | 8 (13%) |
| | Observation | 7 (12%) |
| | Unclear | 7 (12%) |
| | Failure to reattend | 5 (8%) |
| | Case note review | 2 (3%) |
| **Index test(s) measured** | White cell count (WCC) | 52 (87%) |
| | C-reactive protein (CRP) | 34 (57%) |
| | Absolute neutrophil count | 22 (37%) |
| | Neutrophil percentage | 13 (22%) |
| | WCC and CRP | 13 (22%) |
| | WCC, CRP and neutrophil | 6 (10%) |
| | Procalcitonin | 5 (8%) |
| | Neutrophil : lymphocyte ratio | 1 (2%) |
| | WCC and Neutrophil count | 1 (2%) |

For peer review only

**Table 1**: **Pooled overview of design and standards of included studies**

(*In 5 studies authors reported other study designs than those listed. † In one study (2%) department setting was unclear. ‡ value includes all participants in included studies, in 5 studies a paediatric sub-group was provided meaning total paediatric patients in analysis n=28,001. Full details of all study characteristics including design, setting, population characteristics and demographics, exclusion and inclusion criteria is fully detailed in **Supplementary Table 2.** Full details of study reference standard, index tests and cut offs in **Supplementary Table 3**)

For peer review only

| Marker | Studies | Threshold | Pooled sensitivity* (95% CI) | Pooled specificity* (95% CI) | Hypothetical cohort of 1000 children with appendicitis prevalence of 40%† | | |
|---|---|---|---|---|---|---|---|
| | | | | | Reduction in surgery | Missed appendicitis (FN) | Unnecessary surgery(FP) |
| WCC‡ (Cells/µl) | 48 | 10,000 | 0·84 (0·80 – 0·89) | 0·59 (0·52 – 0·66) | 418 | 64 | 246 |
| | | 12,000 | 0·73 (0·66 – 0·79) | 0·71 (0·65 – 0·76) | 534 | 108 | 174 |
| | | 15,000 | 0·53 (0·45 – 0·62) | 0·82 (0·77 – 0·86) | 680 | 188 | 108 |
| CRP§ (mg/L) | 34 | 5 | 0·77 (0·68 – 0·84) | 0·55 (0·45 – 0·64) | 422 | 92 | 270 |
| | | 10 | 0·68 (0·58 – 0·76) | 0·67 (0·58 – 0·74) | 530 | 128 | 198 |
| | | 30 | 0·49 (0·39 – 0·60) | 0·82 (0·75 – 0·87) | 696 | 204 | 108 |
| | | 50 | 0·41 (0·30 – 0·52) | 0·87 (0·81 – 0·91) | 798 | 276 | 78 |
| Neutrophils¶ (% of lymphocytes) | 9 | 75% | 0·78 (0·73-0·82) | 0·61 (0·53-0·69) | 454 | 88 | 234 |
| ANC(Cells/µl) | 5 | <7,500 | 0·90 (0·85 – 0·94 | 0·54 (0·49 – 0·58) | 364 | 40 | 276 |
| | 11 | 7,500 | 0·86 (0·82 – 0·90) | 0·60 (0·54 – 0·65) | 416 | 56 | 240 |
| | 5 | >7,500 | 0·73 (0·70 – 0·77) | 0·77 (0·70 – 0·82) | 570 | 108 | 138 |
| Procalcitonin (ng/ml) | 5 | 0·1 – 0·5 | 0·31 (0·25 – 0·37) | 0·82 (0·70 – 0·90) | 768 | 276 | 108 |
| CRP and WCC± | 13 | | 0·68 (0·52 – 0·81) | 0·78 (0·65 – 0·88) | 468 | 128 | 132 |
| CRP, WCC, and Neutrophils± | 6 | | 0·80 (0·35 – 0·97) | 0·79 (0·60 – 0·90) | 554 | 80 | 126 |

For peer review only

**Table 2.** Pooled estimates of diagnostic accuracy of makers for diagnosis of appendicitis in children ([*]All pooled sensitivity and specificity were obtained from the bivariate model, except for WCC and CRP for which we calculated sensitivity and specificity for selected cut offs using the Steinhauser method, comparison with bivariate mode including Likelihood ratios for all tests is demonstrated in **Supplementary Table 5**. [†]Median prevalence of appendicitis in the included 54 cohort studies is 40% [‡]WCC was evaluated in 48 studies whereof 12 provided multiple cut offs, 4/52 studies reporting WCC provided age-dependent cut offs and could not be used in pooled analysis. [§]CRP was evaluated in 34 studies whereof 11 provided multiple cut offs. [¶]Neutrophils evaluated in 13 studies of which 9 provided cut off at 75% [±]Combinations of tests results using pooled cut-offs, calculations using provided Individual patient data is demonstrated in **Supplementary Table 5**)

For peer review only

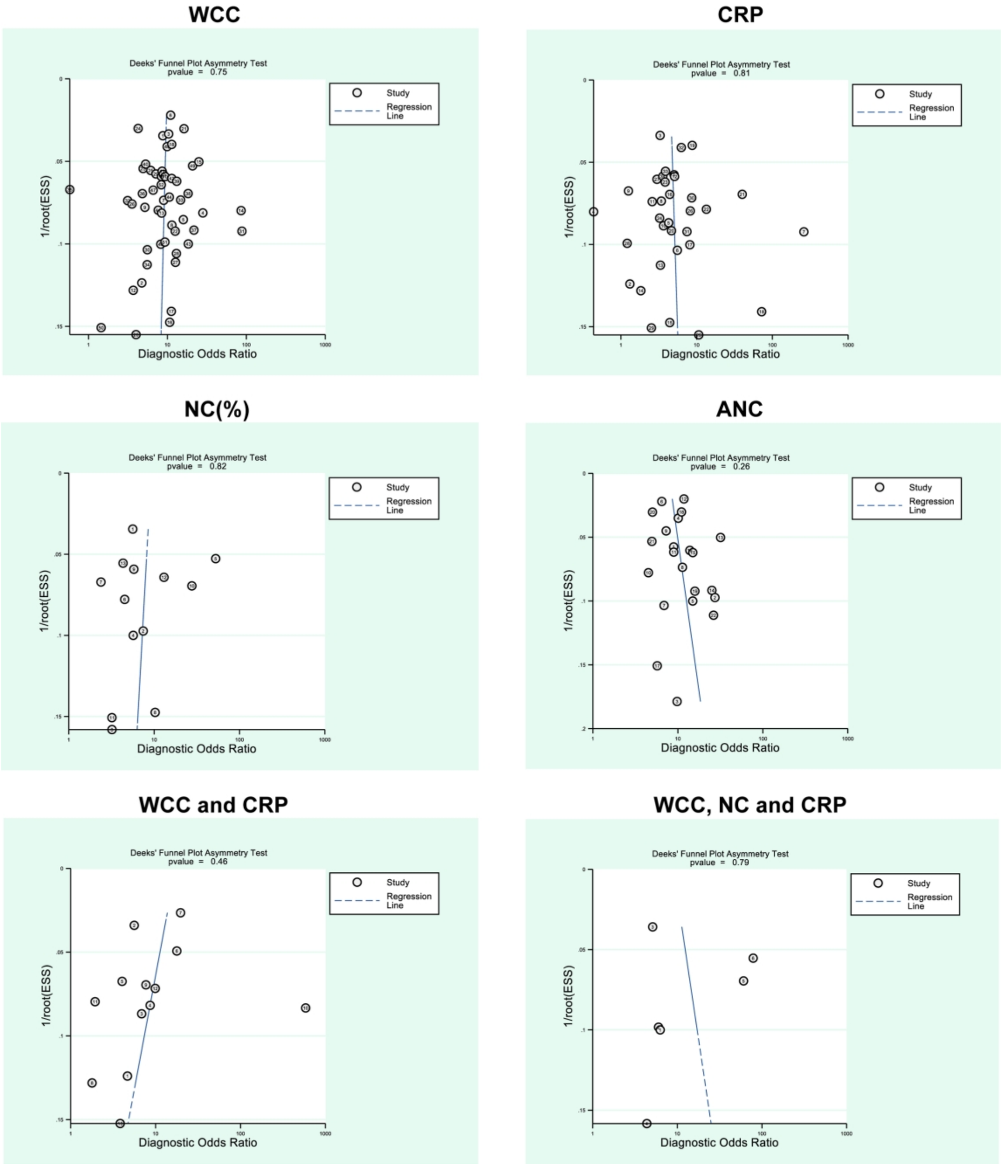


193x224mm (300 x 300 DPI)

# Supplementary Materials for "Diagnostic accuracy of blood tests in paediatric appendicitis – a systematic review and meta-analysis"

Overview of Supplementary materials

**Supplementary Tables:** ........................................................ **2**

*Supplementary Table 1* ........................................................ *6*

*Supplementary Table 2 (related to Table 1):* ........................................................ *10*

*Supplementary Table 3 (Related to Table 1):* ........................................................ *14*

*Supplementary Table 4 (Related to Figure 2):* ........................................................ *16*

*Supplementary Table 5 (Related to Table 2):* ........................................................ *17*

*Supplementary Table 6 (Related to table 2):* ........................................................ *19*

**Supplementary Figure Legends:** ........................................................ **20**

**Search Strategy:** ........................................................ **21**

**QUADAS-2 Scoring criteria** ........................................................ **23**

**Supplementary References** ........................................................ **25**

For peer review only

## Supplementary Tables:

| First Author | Year | Exclusion Reason 1 | Exclusion Reason 2 |
|---|---|---|---|
| 1) Abu-Eshy | 1995 | Incompatible age demographics | Unsuitable control group |
| 2)Barker | 1998 | Unsuitable control group | Incompatible age demographics |
| 3)Bolandparvaz | 2004 | Incompatible age demographics | |
| 4)Bower | 1981 | Unsuitable control group | |
| 5)Clavien | 1987 | Incompatible age demographics | Unsuitable control group |
| 6)Cohen* | 2015 | Data not available in 2x2 | |
| 7)Depinet* | 2016 | Data not available in 2x2 | |
| 8)Erdem | 2015 | Incompatible age demographics | |
| 9)Ficek | 1958 | Unsuitable control group | |
| 10) Kharbanda | 2016 | Study population already included | |
| 11) Lofvenberg | 2016 | Unsuitable control group | Data not available in 2x2 |
| 12) Ng | 2002 | Incompatible age demographics | |
| 13) Park | 2010 | Incompatible age demographics | |
| 14) Vegar-Zubovic | 2003 | Unsuitable control group | |
| 15) Wattanasirichaigoon | 1994 | Incompatible age demographics | |
| 16) Anielski | 2010 | Incompatible age demographics | |
| 17) Atema | 2015 | Incompatible age demographics | |
| 18) Kessler | 2004 | Incompatible age demographics | |
| 19) Nasiri | 2012 | Incompatible age demographics | |
| 20) Thuijls | 2011 | Incompatible age demographics | |
| 21) Yardimci | 2016 | Incompatible age demographics | |
| 22) Yorke* | 2016 | Abstract | Incompatible age demographics |
| 23) Young | 1986 | Review | |
| 24) Agrawal | 2008 | Incompatible age demographics | Unsuitable control group |



For peer review only - http://bmjopen.bmj.com/site/about/guidelines.xhtml

| | | | |
|---|---|---|---|
| 25) Albu | 1994 | Incompatible age demographics | Data not available in 2x2 |
| 26) Allister* | 2011 | Incompatible age demographics | Data not available in 2x2 |
| 27) Amland | 1989 | Incompatible age demographics | |
| 28) Atema | 2015 | Incompatible age demographics | Unsuitable control group |
| 29) Bakal* | 2016 | Data not available in 2x2 | |
| 30) Bealer | 2010 | Incompatible age demographics | |
| 31) Beardsley* | 2014 | Abstract | |
| 32) Beardsley* | 2012 | Abstract | |
| 33) Bhatt* | 2009 | Data not available in 2x2 | |
| 34) Birkhahn | 2006 | Incompatible age demographics | |
| 35) Blab* | 2004 | Data not available in 2x2 | |
| 36) Cardall | 2004 | Incompatible age demographics | |
| 37) Chakhunashvili | 2005 | Unsuitable control group | |
| 38) Chandel | 2011 | Unsuitable control group | |
| 39) Colvin* | 2007 | Data not available in 2x2 | |
| 40) D'Souza | 2013 | Incompatible age demographics | |
| 41) Davies | 1991 | Incompatible age demographics | |
| 42) Dueholm | 1989 | Incompatible age demographics | |
| 43) Duzgun | 2007 | Incompatible age demographics | |
| 44) Eriksson | 1994 | Incompatible age demographics | |
| 45) Eriksson | 1995 | Incompatible age demographics | |
| 46) Eriksson | 1995 | Incompatible age demographics | |
| 47) Erkasap | 2000 | Incompatible age demographics | |
| 48) Escriba* | 2011 | Data not available in 2x2 | |
| 49) Gendel | 2011 | Unsuitable control group | Data not available in 2x2 |
| 50) Groselj-Grenc | 2007 | Study population already included | |
| 51) Gurau | 2016 | Unsuitable control group | Data not available in 2x2 |



For peer review only - http://bmjopen.bmj.com/site/about/guidelines.xhtml

For peer review only

| | | | |
|---|---|---|---|
| 51) Hallan | 1997 | Incompatible age demographics | |
| 53) Hotic | 2012 | Unsuitable control group | Data not available in 2x2 |
| 54) Hu* | 2014 | Data not available in 2x2 | |
| 55) Huang* | 2012 | Data not available in 2x2 | |
| 56) Jahn | 1997 | Incompatible age demographics | |
| 57) Jamaluddin | 2013 | Incompatible age demographics | |
| 58) Johansson | 2007 | Incompatible age demographics | |
| 59) John | 1993 | Incompatible age demographics | |
| 60) Khan | 2004 | Incompatible age demographics | |
| 61) Khan | 2009 | Incompatible age demographics | Unsuitable control group |
| 62) Kharbanda* | 2011 | Data not available in 2x2 | |
| 63) Kosloske* | 2004 | Data not available in 2x2 | |
| 64) Kostic* | 2010 | Data not available in 2x2 | |
| 65) Kyriakidis | 2010 | Incompatible age demographics | |
| 66) Lam | 2014 | Incompatible age demographics | |
| 67) Li | 2005 | Unsuitable control group | Data not available in 2x2 |
| 68) Li | 2012 | Unsuitable control group | |
| 69) Lietzen | 2016 | Incompatible age demographics | |
| 70) Lin* | 2007 | Data not available in 2x2 | |
| 71) Mollitt* | 1988 | Data not available in 2x2 | |
| 72) Oosterhuis | 1993 | Incompatible age demographics | |
| 73) Patel | 2014 | Unsuitable control group | |
| 74) Raftery | 1976 | Incompatible age demographics | Unsuitable control group |
| 75) Ring-Mrozik* | 1991 | Unsuitable control group | |
| 76) Sack* | 2006 | Unsuitable control group | |
| 77) Sarsu* | 2017 | Unsuitable control group | |
| 78) Schellekens | 2013 | Incompatible age demographics | |

For peer review only


For peer review only - http://bmjopen.bmj.com/site/about/guidelines.xhtml

| | | | |
|---|---|---|---|
| 79) Socea | 2013 | Incompatible age demographics | |
| 80) Sondenaa | 1992 | Incompatible age demographics | |
| 81) Suhaymi | 2017 | Unsuitable control group | Incompatible age demographics |
| 82) Summa | 2007 | Incompatible age demographics | |
| 83) Tamanna | 2012 | Incompatible age demographics | |
| 84) Tanrikulu | 2014 | Incompatible age demographics | |
| 85) Tepel | 2004 | Incompatible age demographics | |
| 86) Thimsen | 1989 | Incompatible age demographics | |
| 87) Thompson | 1992 | Incompatible age demographics | |
| 88) Turkyilmaz | 2000 | Unsuitable control group | |
| 89) Uyanik | 2012 | Unsuitable control group | |
| 90) van den Broek | 2002 | Study population already included | |
| 91) Wu | 2010 | Unsuitable control group | |
| 92) Wu | 2006 | Incompatible age demographics | |
| 93) Xharra | 2012 | Incompatible age demographics | |
| 94) Zviedre* | 2014 | Abstract | |
| 95) Brown* | 2004 | Abstract | |
| 96) Hodgkinson* | 2011 | Abstract | |
| 97) Hodgkinson* | 2011 | Abstract | |
| 98) Akin | 2018 | Unsuitable control group | |
| 99) Aydin | 2017 | Incompatible age demographics | |
| 100) Favot | 2017 | Review | |
| 101) Kharbanda* | 2018 | Study population already included | Data not available in 2x2 |
| 102) Mikaere | 2018 | Incompatible age demographics | |
| 103) Nazik | 2017 | Unsuitable control group | |
| 104) Nia | 2018 | Unsuitable control group | |
| 105) Salih | 2017 | Incompatible age demographics | Unsuitable control group |



For peer review only - http://bmjopen.bmj.com/site/about/guidelines.xhtml

| | | | |
|---|---|---|---|
| 106) Zouari | 2017 | Unsuitable control group | |
| 107) Daly | 2018 | Unsuitable control group | |
| 108) Elmas | 2017 | Unsuitable control group | |
| 109) Garcia Gamiz* | 2016 | Abstract | |
| 110) Oztan | 2018 | Unsuitable control group | |
| 111) Shommu | 2018 | Unsuitable control group | |
| 112) Yu* | 2018 | Unsuitable control group | |
| 113) Zouari | 2018 | Unsuitable control group | |
| 114) Sola | 2017 | Unsuitable control group | |
| 115) Zahoor* | 2018 | Abstract | |
| 116) van Dieijen-Visser | 1991 | Incompatible age demographics | |
| 117) Snyder | 1999 | Review | |
| 118) Sengupta | 2009 | Incompatible age demographics | Data not available in 2x2 |
| 119) Scott | 2015 | Incompatible age demographics | |
| 120) Randall Bond* | 1990 | Data not available in 2x2 | |
| 121) Msolli | 2018 | Incompatible age demographics | |
| 122) Malik | 1998 | Incompatible age demographics | |
| 123) Macco* | 2016 | Data not available in 2x2 | |
| 124) Lee | 2008 | Abstract | Incompatible age demographics |
| 125) Kohn | 2005 | Incompatible age demographics | |
| 126) Jang | 2008 | Incompatible age demographics | |
| 127) Haghi | 2018 | Incompatible age demographics | |
| 128) Blitman* | 2015 | Data not available in 2x2 | |
| 129) Akturk | 2018 | Incompatible age demographics | |
| 130) Ahmed | 2017 | Unsuitable control group | |

**Supplementary Table 1:** Overview of studies excluded at full paper review stage and reason(s) for exclusion (* = Author was contacted and confirmed exclusion or did not respond. Unsuitable control group also includes those studies with no control group)



For peer review only - http://bmjopen.bmj.com/site/about/guidelines.xhtml

For peer review only

| Study | Study Design; Setting; Country | No. of children in study; with AA (% prevalence) | Age range (years) | Gender (n (%) male) | Inclusion criteria | Exclusion criteria |
|---|---|---|---|---|---|---|
| Altali, 2017 (1) | Prospective cohort study; Hospital ED; Spain | 331, 116 (35%) | 2 to 20 | 175 (53%) | Abdominal pain <72 hours suspicious of AA as reviewed by doctor | Radiology prior to presentation. Pregnancy. Previous appendicectomy. Cancer. Trauma. Surgery in last 7 days. Steroids, immunosuppressive treatment or chemotherapy. |
| Anandalwar 2015 (2) | Retrospective cohort study; ED; USA | 845, 393 (47%) | 3 to 18 | * (48%) | Consultation by surgeon for suspected AA. Underwent abdominal US. | History of abdominal surgery. Cognitive delay. Neurologic impairment. Immunodeficiency. Antibiotic use. Missing laboratory data. Abdominal imaging before presentation. |
| Andersson, 1999 (3) | Prospective cohort study; ED, 2 centres; Sweden | 496,[data provided n=296 <18yrs] 79(27%) | 10 to 86 | 133 (45%) | Patients admitted with suspected AA | * |
| Andersson, 2008 (4) | Prospective cohort study; Hospital, 2 centres; Sweden | 545 [data provided n=436 <18yrs, 249 of which not included in Andersson 1999] , 191(18%) | All | 58 (41%) | Admitted with suspected AA | Patients with missing blood tests. |
| Andersson, 2014 (5) | Prospective cohort study; Hospital ED, 2 centres; Sweden | 428 [data provided n=259 children], 177[76](30%) | 4 to 84 | 133 (49%) | Abdominal pain <5 days suggestive of AA | Pregnancy. Inability to consent. Multiple missing variables. |
| Andersson, 2017 (6) | Prospective interventional study with a nested randomized trial; ED, 21 centres; Sweden | 3791[data provided n=1046 children], [338](33%) | 5 to 95 | 2052 (54%) | Acute abdominal pain suggestive of AA | Pregnancy. Pain lasting >5 days. |
| Bachur 2016 (7) | Prospective cohort study; ED; USA | 2133, 870 (41%) | 3 to 18 | * (52%) | Acute abdominal pain < 72 hours. Had a WCC count and WCC differential obtained. | Pregnancy. Prior abdominal surgery. Chronic gastrointestinal condition. Severe developmental delay. |
| Bal 2016 (8) | Prospective cohort study; PED; Turkey | 96, 56 (58%) | 4 to 18 | 58 (21%) | Acute abdominal pain < 96 hours. Surgical consultation for suspected AA. Documented follow up. | Pregnancy. Familial Mediterranean fever. Chronic abdominal illness. Sickle cell anaemia. Cystic fibrosis. Previous abdominal surgery. Immunosuppressive therapy. |
| Beltran, 2007 (9) | Prospective Cohort study; Hospital; ED; Chile | 121, 71 (59%) | 5 to 14 | 73 (60%) | Presenting with abdominal pain, nausea, vomiting and fever. | Re-presentation. |
| Benito, 2016 (10) | Prospective cohort study; PED; Spain | 185, 89 (48%) | 2 to 14 | 123 (66%) | Abdominal pain suggestive of AA as evaluated by attending physician. | Symptoms >5 days. Prior appendicectomy. Urinary tract infection. Cancer. Inflammatory disease. Corticoids |
| Calvo, 1998 (11) | Retrospective cohort study; Hospital; Spain | 231, 141 (61%) | 2 to 14 | 149 (65%) | Acute abdominal pain. Inpatient. | Less severe cases of abdominal pain. |
| Cayrol 2016 (12) | Prospective cohort study; PED; Spain | 135, 52 (39%) | 1 to 15 | 69 (51%) | Children with acute abdominal pain suggestive of AA. | Previous appendicectomy. Immunological, haemostatic or thrombotic disorder. Vasculitis. Cardiopathy. Cancer. Pregnancy. |
| Corkum, 2018 (13) | Retrospective cohort study; ED; USA | 738, 156 (21%) | 5 to 18 | * | Child with suspected AA -defined by undergoing an abdominal US and had blood test. | * |
| Doraiswamy 1979 (14) | Case control study; Hospital; UK | 375, 225 (60%) | 5 to 15 | * | Cases: children with AA. Controls: children with normal appendix on histology and children with acute abdominal pain. | Extra-abdominal complications. |
| Esparaz, 2019 (15) | Retrospective cohort study; ED; USA | 327, 56 (17%) | 2 to 17 | * | Abdominal US for suspected AA | None |
| Fleischman, 2013 (16) | Prospective cohort study; PED; USA | 178, 65 (37%) | 3 to 18 | 84 (47%) | Patients with symptoms suggestive of AA, duration <7 days. | Negative appendectomy but other diagnosis indicated by imaging/surgery |



For peer review only - http://bmjopen.bmj.com/site/about/guidelines.xhtml

| | | | | | | |
|---|---|---|---|---|---|---|
| Goldman, 2008(17) | Prospective cohort study; ED; Canada | 849, 123 (36%) | 1 to 17 | * | Children with chief complaint of abdominal pain for <7 days. | Known diagnosis of AA (US or CT) on arrival to ED. Patients with prior appendicectomy. |
| Gonzalez del Castillo, 2016(18) | Prospective cohort study; ED, 4 centres; Spain | 321, 111 (35%) | 2 to 20 | 167 (52%) | Abdominal pain <72 hours suggestive of AA | Ulcerative colitis. CT prior to consent. Pregnancy. Appendicectomy. Cancer. Trauma. Surgery within 7 days. Immunosuppressive treatment or chemotherapy. |
| Groselj-Grenc 2007(19) | Nested case-control study; Hospital; Slovenia | 82, 49 (60%) | 3 to 14 | 53 (65%) | Cases: Children who underwent surgery for AA. Controls: Diagnosis of non-specific abdominal pain or sonographic mesenteric lymphadenitis. | Clinically or microbiologically established infections. |
| Hsiao 2005(20) | Case-control; Hospital; Taiwan | 222, 111 (50%) | 0 to 14 | * (ratio 1.52 M:1 F) | Cases: Diagnosis of AA on histology. Controls: Suspected AA in same period. | None |
| Huckins 2013(21) | Prospective cohort study; ED; 12 centres; USA | 503, 144 (29%) | 2 to 20 | 243 (48%) | Right lower quadrant/ generalized abdominal pain with other signs/ symptoms consistent with AA of duration < 72 hours. | Previous appendicectomy. Cancer. Bleeding disorder. Trauma. Invasive abdominal procedures or prior diagnostic imaging during this illness. Participation in other research protocols within 2 weeks. Unable to obtain consent. |
| Huckins 2016(22) | Prospective cohort study; ED; 29 centres; USA | 1887, 477 (25%) | 2 to 20 | 848 (45%) | Right lower quadrant/generalized abdominal pain <72 hours with other signs/symptoms suggesting AA. Informed consent. | Previous appendicectomy. Cancer. Bleeding disorder. Autoimmune disorder. Trauma. Immunosuppressive medications. Invasive abdominal procedure within 2 weeks. Surgery without imaging. Prisoners. Participation in other research protocol within 30 days. |
| Kaiser, 2018(23) | Case control study; Hospital; Austria | 64, 39(61%) | *(mean age 12 yr) | 24 (38%) | Cases: children who underwent appendectomy. Controls: abdominal pain and improved under conservative treatment | * |
| Khan 2012 (24) | Prospective cohort study; PED; USA | 50, 22 (44%) | 5 to 17 | 22 (44%) | Right lower quadrant abdominal pain without signs of gastroenteritis. | Chronic haematological, immunological or gastrointestinal disease. Pregnancy. |
| Khanafer, 2016(25) | Prospective cohort study; paediatric ED; Canada | 180, 55(31%) | 5 to 17 | 78 (43%) | Abdominal pain <5 days. AA in differential from ED with bloods done. | Previous appendicectomy. Positive imaging prior to presentation. Pregnancy. Immuno-suppressive disorders. |
| Kharbanda 2005(26) | Prospective cohort study; PED; USA | 601, 211 (37%) | 3 to 18 | 307 (51%) | Surgical consultation for possible AA | Pregnant. Previous abdominal surgery. Chronic medical condition. Radiological study of abdomen within 2 weeks. |
| Kharbanda, 2012 (27) | Prospective cross-sectional study; PED; USA | 176, 58 (33%) | 3 to 18 | 92 (52%) | Acute abdominal pain <96 hours duration evaluated for AA as defined by clinician. | Pregnancy. Prior abdominal surgery, chronic GI illness. Inaccurate history. Imaging / abdominal trauma in <7 days. |
| Kharbanda, 2012 (28) | Prospective cross-sectional study; PED; 10 centres; USA | 2625, 1018 (39%) | 3 to 18 | 1339 (51%) | Abdominal pain <96 hours duration in keeping with AA (as defined by clinician). | Pregnancy. Prior abdominal surgery. Chronic abdominal illness. Sickle cell. Cystic fibrosis. Radiological investigation diagnosing AA before arrival. Trauma within 7 days |
| Ko, 1995(29) | Prospective cohort study; Hospital; Taiwan | 47, 27 (57%) | 2 to 14 | 29 (62%) | Patients with clinically suspected AA | Atypical clinical signs. Didn't complete protocol. |
| Kouame, 2005(30) | Prospective diagnostic accuracy study; Hospital; France | 101, 68 (67%) | 2 to 15 | 52 (51%) | Hospitalised children with abdominal pain that was suspected to require surgery. | Antibiotics prior to hospital presentation. |
| Kwan, 2010(31) | Prospective observational study; PED; USA | 209, 115 (55%) | 1 to 18 | 123 (59%) | Abdominal pain suspicious of AA. | Pregnancy. Bleeding disorder. Severe anaemia. Chronic disease. Abdominal surgery in the previous year. |
| Lin, 2009(32) | Retrospective cohort study; ED; Taiwan | 100, 53 (53%) | 2 to 17 | 46(46%) | Children with RLQ pain presenting to ED | Patients who went home against medical advice. |
| Lycopoulou, 2005(33) | Prospective observational with nested case-control study; PED; Greece | 60, 42 (70%) | 5 to 14 | 30 (50%) | A nested control of children operated for AA; Controls: children presenting to PED during same period with symptoms and signs of AA | No consent. Retrocaecal abscess. Normal appendix histology. |



For peer review only - http://bmjopen.bmj.com/site/about/guidelines.xhtml

| | | | | | | |
|---|---|---|---|---|---|---|
| Malia, 2019(34) | Prospective cohort study; Paediatric hospital ED; USA | 762, 225 (30%) | 0 to 18 | 356 (47%) | Children with suspected AA who underwent US | Patient absconded, previous appendicectomy, appendix not visualised on US, US at other facility. |
| Mandeville, 2011(35) | Prospective observational study; PED; USA | 287, 155 (54%) | 4 to 17 | 151 (53%) | Abdominal pain suggestive of AA | Pregnancy, previous abdominal surgery, chronic medical condition, non-verbal, radiological investigation of abdomen in preceding 2 weeks, no follow up. |
| Miguez, 2016(36) | Prospective cohort study; Paediatric hospital ED; Spain | 136, 44 (32%) | 3 to 16 | 76 (56%) | Admitted to ED with abdominal pain <96 hours AA suspected at initial evaluation. | Appendicectomy /recent surgery. Immune disease. Chronic respiratory or cardiovascular disease. Inflammatory bowel disease. Antibiotics or steroids in the last month. |
| Mohammed, 2004(37) | Prospective cohort study; Hospital paediatric surgical department; Libya | 216, 130 (60%) | 3 to 13 | 139(64%) | Child presenting with clinical suspicion of AA | * |
| Moreno, 2012(38) | Retrospective Cohort study; Hospital; Spain | 184, 85 (46%) | * (Mean age 8.9 yr) | 110 (60%) | Acute abdominal pain suggestive of AA. | * |
| Naqvi, 2019(39) | Observational cohort study; Tertiary Paediatric Hospital; Canada | 185, 79 (43%) | 4 to 17 | 86 (46%) | Children with suspected AA demonstrated by US or referral to paediatric surgical team. Also children with sepsis defined by SIRS criteria or PICU admission (AA sub-group reported) | Previous appendicectomy, resuscitation in ED, discharge to PICU, pregnancy, AP >5 days, immune-suppression, abdominal imaging prior to admission, life expectancy <24 hours. |
| Oikonomopoulou, 2019(40) | Prospective cohort study; PEDs, 6 centres; Spain | 285, 100 (35%) | 0 to 18 | 167 (59%) | Clinical suspicion of AA after physician assessment | Pain >72hr, lack of blood sample, history of surgery (within 3 months)/ immune pathology/ inflammatory bowel disease/ cardio-respiratory disease, recent steroid or antibiotic use |
| Ovrebo, 1993(41) | Retrospective case series; Hospital surgical department; Norway | 470, 161 (34%) | 0 to 15 | 235 (50%) | All patients presenting with abdominal pain <1 week duration suggestive of AA. | Abdominal trauma. Incarcerated herniae. |
| Ozguner, 2014(42) | Prospective cohort study; Children's hospital; Turkey | 49, 34(69%) | 3 to 17 | 34 (69%) | Children hospitalised with right lower-quadrant pain suspicious of AA between 0400 and 1500. | If sample for CD64 measurement could not be obtained. |
| Peltola, 1986(43) | Prospective cohort study; Children's hospital; Finland | 162, 94 (58%) | 1 to 16 | 77 (48%) | Children presenting with abdominal pain or symptoms suggestive of AA | Symptoms suggestive or respiratory infection. |
| Prada-Arias, 2017(44) | Prospective cohort study; Tertiary hospital PED; Spain | 275, 132 (48%) | 5 to 15 | 153 (56%) | Clinical suspicion of AA with blood tests and US performed | Symptom duration <6hr or >72hr. Haematological / oncological / hepatic / infectious or inflammatory disease present. >12 hours between blood test and surgery |
| Rodríguez Sánchez de la Blanca, 2014(45) | Prospective cohort study, PED, Spain | 105, 41 (39%) | <16 | 59 (56%) | Abdominal pain or clinical suspicion of AA | Antibiotic treatment in the preceding 10 days, known inflammatory pathology |
| Salo, 2016(46) | Case control study; PED; Sweden | 44, 22 (50%) | 3 to 14 | 27 (61%) | Referral to paediatric surgeon with suspicion of AA | Immuno-modulating therapy or clear-cut diagnosis of AA |
| Samuel, 2002(47) | Prospective cohort study; Hospital, 2 centres; UK | 1170, 734 (63%) | 4 to 15 | 752(64%) | Abdominal pain suggestive of AA | Appendicula mass with peri-appendiceal abscess |
| Sanchez, 1998(48) | Prospective cohort study; Paediatric hospital ED; Spain | 195, 94 (48%) | 2 to 14 | 107(55%) | Children with suspected AA | * |
| Shommu, 2018(49) | Prospective cohort study; PED; Canada | 140, 52 (36%) | 5 to 17 | 61 (44%) | Clinically suspected AA or US evaluation for AA with IV placed and did not require intensive care treatment from PED. Informed consent | Previous appendectomy. Pregnancy. Abdominal pain >5 days. Immuno-suppression. Previously enrolled in the study. Imaging study performed elsewhere |


For peer review only - http://bmjopen.bmj.com/site/about/guidelines.xhtml

| | | | | | | |
|---|---|---|---|---|---|---|
| Thompson, 2015(50) | Retrospective cohort study; ED, 12 centres; Canada | 619, 510 (83%) | 3 to 17 | 352 (53%) | Children admitted with suspected AA | Intensive care unit admission. |
| Thompson, 2016 (51) | Retrospective cohort study; Children's hospital ED; Canada | 1315, 561[data provided n=471] (41%) | 2 to17 | 565 (43%) | Children investigated for suspected AA with blood culture sample collected | * |
| Tuncer, 2019(52) | Retrospective cross-sectional study; Paediatric Surgical/Medical department; Turkey | 301; 137 (46%) | *(Mean age 11yr) | 139 (46%) | Children with acute abdominal pain, recorded diagnosis of acute appendicitis, mesenteric lymphadenitis or familial Mediterranean fever. | Heart failure, peripheral vascular / haematological /liver disease, anticoagulation, steroid use, concurrent acute or chronic infection |
| Turkyilmaz, 2006(53) | Prospective cohort study; Hospital; Turkey | 105, 61 (58%) | 3 to 16 | 68 (65%) | Admission with acute right-lower abdominal pain | * |
| Van Den Broek, 2004(54) | Prospective cohort study; ED, 2 centres; Netherlands | 275, 169 (66%) | *(Mean age 9 yr) | 148 (58%) | GP referral with suspicion of AA | * |
| Wang 2007(55) | Prospective and retrospective cohort study; PED; USA | 722, 74 (18%) | 1 to 19 | 334 (46%) | Prospective: ED presentation with suspected AA. Retrospective: AA on surgery, radiology, and laboratory records. | History of trauma |
| Wu, 2012(56) | Prospective cohort study; ED; Taiwan | 594, 306 (52%) | 4 to 18 | 358 (60%) | Children with clinically suspected AA, as determined by emergency physician. | Symptoms >3 day. Patients lost to follow up. |
| Yap, 2015(57) | Prospective cohort study; Paediatric hospital ED; Singapore | 747, 229 (32%) | 4 to 16 | 429(57%) | Admitted to ED with suspected AA. | Previous appendicectomy, pregnancy, chronic medical or malignant conditions. CT in the previous month. |
| Yazici, 2010(58) | Retrospective case series; Paediatric surgical department; Turkey | 240, 183 (76%) | 3 to 16 | * | Patients with acute abdominal pain | Urinary tract infection, Henoch-Schonlein pupura or gastroenteritis. |
| Zouari, 2016(59) | Prospective cohort study; Paediatric surgical department; Tunisia | 292, 132 (45%) | 3 to 14 | 170(58%) | Diagnosis of suspected AA by consultant paediatric surgeon on the basis of clinical history and physical exam | Patients who had not undergone US |
| Zuniga, 2012(60) | Prospective cohort study; Hospital ED; Spain | 101, 28 (28%) | 0 to 14 | 55 (54%) | Presenting with clinical suspicion of AA | Pain >7 days. Previous appendicectomy. No blood tests. |

**Supplementary Table 2 (related to Table 1):** Overview of demographics of included studies describing design, setting and population characteristics (* = data not provided or none described. AA= acute appendicitis. (P)ED = (Paediatric) Emergency Department. US = Ultrasound. CT = Computed Tomography. PICU= Paediatric Intensive Care Unit. In studies where authors provided paediatric data sub-population information is shown in square brackets.)

For peer review only

| Study | Reference Standard | Index Test(s) measured | Index test cut off value(s) |
| --- | --- | --- | --- |
| Altali, 2017 (1) | Histopathology or discharge diagnosis | • WCC<br>• Neutrophil count<br>• CRP | >10,500/mm$^3$<br>>6,600/mm$^3$<br>>5mg/L |
| Anandalwar 2015 (2) | Pathology proven appendicitis * | • WCC<br>• Neutrophil % | >9,000/mm$^3$<br>>65% |
| Andersson, 1999‡(3) | Operation note and histopathology or failure to re-present | • WCC<br>• Neutrophil count<br>• Neutrophil %<br>• CRP<br>WCC/CRP as separate and combined | ‡ |
| Andersson, 2008‡(4) | Histopathology or review of case files in the following 1 month. | • WCC<br>• Neutrophil count<br>• Neutrophil %<br>• CRP<br>WCC/CRP as separate and combined | ‡ |
| Andersson, 2014‡ (5) | Histopathology or 6 month follow up | • WCC<br>• Neutrophil count<br>• Neutrophil %<br>• CRP<br>WCC/CRP as separate and combined | ‡ |
| Andersson, 2017 ‡ (6) | Histopathology or 30 day follow up | • WCC<br>• Neutrophil count<br>• Neutrophil %<br>• CRP<br>WCC/ CRP as separate and combined | ‡ |
| Bachur 2016 (7) | Operative diagnosis, telephone follow-up (1 -2 weeks) or record review (90 days) after visit. | • WCC<br>• Neutrophil count | 5-15,000/mm$^3$†<br>5-15,000/mm$^3$† |
| Bal 2016 (8) | Surgery and pathology report or follow-up by phone interview 7-14 days after visit | • WCC<br>• Neutrophil count<br>• CRP<br>• Procalcitonin | >13,400/mm$^3$<br>>9,400/mm$^3$<br>>0·9mg/L<br>0·37ng/mL |
| Beltran, 2007 (9) | Histopathological report or discharge diagnosis | • WCC<br>• CRP | >12,500/mm$^3$<br>>47mg/L |
| Benito, 2016 (10) | Histopathological analysis of surgical specimen, telephone follow up 15 days after consultation, or check of electronic registry if not contactable. | • WCC<br>• Neutrophil count<br>• CRP<br>• Procalcitonin | <10,000/mm$^3$<br><7,500/mm$^3$<br><20 mg/L<br><0·1ng/ml |
| Calvo, 1998 (11) | Histopathological examination or discharge diagnosis | • WCC<br>• CRP<br>Tests reported as separate and as combination | >15,000/mm$^3$<br>>20mg/l |


For peer review only - http://bmjopen.bmj.com/site/about/guidelines.xhtml

| | | | |
|---|---|---|---|
| Cayrol 2016 (12) | Surgery and histopathology or clinical decision with telephone follow up 5 days later | • WCC<br>• CRP | >10,000/mm$^3$<br>>30mg/L |
| Corkum, 2018 (13) | Histopathology and operative note or 30 day follow up | • Neutrophil count | >8,000/mm$^3$ |
| Doraiswamy 1979 (14) | Appendicitis confirmed or refuted at surgery or recovered without surgery | • WCC<br>• Neutrophil % | >15,000/mm$^3$ or >13,000/mm$^3$[†]<br>>50%, >65% or >75%[†]<br>(age dependent) |
| Esparaz, 2019 (15) | Histopathology * | • WCC | >10,000/mm$^3$ |
| Fleischman, 2013 (16) | Surgical pathology, positive CT scan finding or diagnosis and follow up at 2 weeks | • WCC<br>• Neutrophil count<br>• Neutrophil % | >10,000/mm$^3$<br>>6750/mm$^3$<br>>75% |
| Goldman, 2008(17) | Appendicectomy pathology or follow-up 5 to 7 days after ED discharge | • WCC<br>• Neutrophil count | >10,000/mm$^3$<br>>7,500/mm$^3$ |
| Gonzalez del Castillo, 2016[‡](18) | Histopathology reported by blinded pathologist or telephone follow up 2 weeks after discharge | • WCC<br>• CRP | >12,000/mm$^3$<br>>5mg/L |
| Groselj-Grenc 2007(19) | Appendicitis diagnosed by surgery or ultrasound, or diagnosis of non-specific abdominal pain/sonographic mesenteric adenitis | • WCC<br>• CRP | >11,600/mm$^3$<br>>9.0mg/L |
| Hsiao 2005 (20) | Surgery or pathology report, or appendicitis excluded in final diagnosis | • WCC<br>• Neutrophil % | >10,400/mm$^3$<br>>75% |
| Huckins 2013(21) | Surgical pathology report after appendectomy  or discharge diagnosis | • WCC<br>• CRP<br>Tests only reported in combination | >10,000/mm$^3$<br>>8mg/L |
| Huckins 2016(22) | Surgical pathology reports after appendectomy or follow-up telephone calls at 14 days (±3 days) | • WCC<br>• CRP<br>Tests only reported in combination | >10,000/mm$^3$<br>>8mg/L |
| Kaiser, 2018[‡](23) | Histopathology or discharge diagnosis | • WCC<br>• CRP<br>Tests as separate and combined | >10 -15,000/mm$^3$[†]<br>5-50mg/L[†] |
| Khan 2012 (24) | Histopathological evidence or telephone follow up 24 hour and 2 weeks after discharge | • Procalcitonin | >0·39ng/ml |
| Khanafer, 2016[‡](25) | Histopathology  or follow up 2 weeks after discharge | • WCC | 10-15,000m/$^3$[†] |
| Kharbanda 2005(26) | Pathology or follow-up telephone call 2 to 4 weeks after the ED visit | • WCC<br>• Neutrophil count | >8850/mm$^3$<br>6·75x10$^3$/ul |
| Kharbanda, 2012(27) | Surgical histology or telephone follow up 14-21 days after admission/note review if uncontactable. | • WCC | >8850/mm$^3$ |
| Kharbanda, 2012 (28) | Histology or telephone follow up 2 weeks after discharge or note review 90 days after discharge if unable to contact. | • Neutrophil count | >6750/mm$^3$ |
| Ko, 1995(29) | Surgical histology or diagnosis on discharge | • WCC<br>• Neutrophil %<br>• CRP | >10,000/mm$^3$<br>>75%<br>>9mg/L and >50mg/L |
| Kouame, 2005(30) | Histology * | • Procalcitonin | >0.5µg/L |
| Kwan, 2010(31) | Operative reports and histology or telephone follow up or note review 2-6 weeks after discharge | • WCC<br>• CRP<br>Tests reported as separate and in combination | >12,000/mm$^3$<br>>30mg/L |

For peer review only

| | | | |
|---|---|---|---|
| Lin, 2009(32) | Appendectomy (histologic examination) or telephone follow-up within 1 week of consultation | • WCC<br>• CRP | >15,000/$mm^3$<br>>1 mg/dL |
| Lycopoulou, 2005(33) | Appendicectomy (histological examination) or 1 week follow up | • WCC<br>• CRP | >10,000/$mm^{3}$†<br>>10mg/L |
| Malia, 2019(34) | Histopathology report or failure to re-attend within 1 week | • WCC<br>• CRP | >10,000/$mm^3$<br>>5mg/L |
| Mandeville, 2011(35) | Histopathology or telephone follow up at 2 weeks | • WCC<br>• Neutrophil % | >10,000/$mm^3$<br>>75% |
| Miguez, 2016(36) | Histopathology of appendicectomy specimen or telephone follow up 5-7 days afterwards. | • WCC<br>• Neutrophil count<br>• CRP | >12,300/$mm^3$<br>>9,600/$mm^3$<br>>3 mg/L |
| Mohammed, 2004(37) | Appendicectomy (histopathological results) or clinical observation | • WCC<br>• Neutrophil %<br>• CRP<br>Tests reported as separate and in combination (all three) | >11,000/$mm^3$<br>>75%<br>>8mg/L |
| Moreno, 2012(38) | Surgery, histology, clinical observation or follow up | • WCC<br>• CRP | >12,800/$mm^3$<br>>18mg/L |
| Naqvi, 2019(39) | Histopathology, insertion of percutaneous drain or failure to re-attend within 2 weeks | • CRP | >7·9mg/L |
| Oikonomopoulou, 2019(40) | Histopathology and surgeons notes, telephone follow up after 5-7 days or checked for readmission if no response | • WCC<br>• Neutrophil count<br>• CRP<br>Tests reported as separate and in paired combinations | >13,300/$mm^3$<br>>10,800/$mm^3$<br>>12·5mg/L |
| Ovrebo, 1993(41) | Operative note (all) and histology (some) or discharge diagnosis | • WCC<br>• CRP<br>Tests reported as separate and in combination | >11,000/$mm^3$<br>>10mg/L |
| Ozguner, 2014‡(42) | Operative findings, histopathological examination and discharge diagnosis | • WCC<br>• CRP | >12,000/$mm^3$<br>>16mg/L |
| Peltola, 1986(43) | Histology on those operated or diagnosis of non-specific abdominal pain | • WCC<br>• CRP<br>Tests reported as separate and in combination | >14,900/$mm^3$<br>>10mg/L |
| Prada-Arias, 2017(44) | Histology and surgical report or no specific disease identified, no findings on US and no antibiotic treatment | • WCC<br>• Neutrophil count<br>• CRP | >11,000/$mm^3$<br>>7,000/$mm^3$<br>>25·2mg/L |
| Rodríguez Sánchez de la Blanca, 2014(45) | Histopathology* | • WCC<br>• CRP<br>• Procalcitonin | >13,000/$mm^3$<br>>5mg/L<br>>0·5µg/L |
| Salo, 2016(46) | Surgical note and histopathology report or follow up | • WCC<br>• Neutrophil count<br>• Neutrophil %<br>• CRP<br>Combined WCC and neutrophil count reported | >13-16,000/$mm^{3}$†<br>>6·5-7,000/$mm^{3}$†<br>>71%<br>>15mg/L<br>(ranges age dependent) |

For peer review only

| | | | |
|---|---|---|---|
| Samuel, 2002(47) | Histology in surgical group* | • WCC<br>• Neutrophil count | >10,00/mm$^3$<br>>7,500/mm$^3$ |
| Sanchez, 1998(48) | Histology and surgical report or discharge diagnosis | • WCC<br>• CRP | >14000/mm$^3$<br>>30mg/L |
| Shommu, 2018‡(49) | Histopathology* | • WCC<br>• Neutrophil count<br>• CRP | ‡ (Provided 2x2 tables for multiple cut offs of tests but not IPD) |
| Thompson, 2015‡(50) | Histopathology or period of observation | • WCC<br>• Neutrophil count<br>• CRP | ‡ |
| Thompson, 2017‡(51) | Histopathology or discharge diagnosis | • WCC<br>• Neutrophil count<br>• CRP | ‡ |
| Tuncer, 2019(52) | Histopathology or discharge diagnosis | • WCC | >13,050/mm$^3$ |
| Turkyilmaz, 2006(53) | Histopathology and operative assessment or clinical observation | • WCC | >15000/mm$^3$ or 13,000/mm$^3$† (age dependent) |
| Van Den Broek, 2004(54) | Histology* | • WCC | >10,000/mm$^3$ |
| Wang 2007(55) | Histology or review of medical records | • WCC<br>• Neutrophil %<br>Tests reported as separate and in combination | †(Age dependent cut-off)<br>>80% |
| Wu, 2012(56) | Surgery, histological examination or follow up interview two weeks after visit | • WCC<br>• CRP | >11000/mm$^3$<br>>25 and 89mg/L |
| Yap, 2015(57) | Histology, operative findings or observation on the ward until >24 hours pain free | • WCC<br>• Neutrophil %<br>• CRP<br>Tests reported as separate and in combination (all three) | > 10,000/mm$^3$<br>>75%<br>>5mg/L |
| Yazici, 2010(58) | Histopathology or observation for 24-48 hours | • WCC | >10,000/mm$^3$ |
| Zouari, 2016(59) | Histology showing acute appendicitis or failure to represent for 2 weeks | • CRP | 6mg/L |
| Zuniga, 2012(60) | Histopathology or failure to re-present for 7 days | • WCC<br>• Neutrophil count | >10,000/mm$^3$<br>>7,500/mm$^3$ |

**Supplementary Table 3 (Related to Table 1):** Overview of included studies Index tests, cut off values and reference standards. (CRP = C-reactive protein. WCC = White Cell Count. PMN = Polymorphonuclear cells. * = non-AA not defined/not clearly defined, †=multiple cut offs or range in paper, ‡=author provided raw data)


For peer review only - http://bmjopen.bmj.com/site/about/guidelines.xhtml

| Study | RISK OF BIAS | | | | APPLICABILITY CONCERNS | | |
|---|---|---|---|---|---|---|---|
| | PATIENT SELECTION | INDEX TEST | REFERENCE STANDARD | FLOW AND TIMING | PATIENT SELECTION | INDEX TEST | REFERENCE STANDARD |
| Altali, 2017 | Low | Unclear | Low | Unclear | Low | High | Low |
| Anandalwar, 2015 | High | High | Unclear | Unclear | Low | High | Unclear |
| Andersson, 1999 | Unclear | High | High | High | Unclear | Unclear | High |
| Andersson, 2014 | Low | Unclear | Unclear | High | Low | Unclear | Low |
| Andersson, 2017 | Low | Unclear | Unclear | High | Low | Low | Low |
| Andersson,2008 | Low | Unclear | Unclear | High | Low | Unclear | Low |
| Bachur, 2016 | Low | Unclear | Unclear | High | Low | Unclear | Unclear |
| Bal, 2016 | High | Unclear | Unclear | High | Low | Low | Low |
| Beltran, 2007 | Low | Low | High | Unclear | Low | Low | Unclear |
| Benito, 2016 | High | Low | High | High | Low | Low | Low |
| Calvo Rigual, 1998 | Low | Unclear | High | High | Low | Low | Low |
| Cayrol, 2016 | High | Low | High | High | Low | Low | High |
| Corkum, 2018 | High | Unclear | Unclear | High | Low | Unclear | Low |
| Diaz Moreno, 2012 | Low | High | Unclear | Unclear | Low | Unclear | Unclear |
| Doraiswamy, 1979 | High | Unclear | Unclear | High | Low | Low | Unclear |
| Esparaz, 2019 | High | Unclear | High | High | Low | Low | Low |
| Fleischman, 2013 | High | Unclear | Unclear | High | Low | Low | Low |
| Goldman, 2008 | Low | Unclear | High | High | Low | Low | High |
| Gonzalez, 2016 | Low | High | High | High | Low | High | Low |
| Groselj-Grenc, 2007 | Low | High | Unclear | High | Low | Low | High |
| Hsiao, 2005 | High | Unclear | Unclear | High | Low | Low | Unclear |
| Huckins, 2013 | High | Low | High | High | High | Low | High |
| Huckins, 2016 | High | Low | Low | High | High | Low | Low |
| Kaiser, 2018 | High | Unclear | High | High | Low | Unclear | High |
| Khan, 2012 | Low | High | Unclear | Unclear | Low | High | Low |
| Khanafer, 2016 | High | Unclear | Unclear | High | Low | Low | Unclear |
| Kharbanda, 2005 | High | Unclear | Unclear | High | Low | Low | Low |
| Kharbanda, 2012 | Low | High | High | High | Low | Low | Low |
| Kharbanda, 2012 | High | Unclear | Unclear | High | Low | Low | Low |
| Ko, 1995 | Low | Unclear | Unclear | High | Low | Low | Unclear |
| Kouame, 2005 | Low | Unclear | Unclear | Unclear | Low | Low | Unclear |
| Kwan, 2010 | Low | High | Unclear | High | Low | Low | Low |
| Lin, 2009 | Low | Unclear | High | High | Low | High | High |
| Lycopoulou, 2005 | High | Unclear | High | High | Low | Low | High |
| Malia, 2019 | High | High | High | High | High | Unclear | Low |
| Mandeville, 2011 | High | Unclear | Low | High | Low | Low | Low |
| Miguez, 2015 | Low | High | High | High | Low | High | High |
| Mohammed, 2004 | Low | Unclear | Unclear | High | Low | Low | Unclear |
| Naqvi, 2019 | High | High | Unclear | High | Unclear | High | Low |
| Oikonomopoulou, 2019 | Low | High | High | High | Low | High | Low |
| Ovrebo, 1993 | Unclear | High | High | High | Low | Low | High |
| Ozguner, 2014 | High | High | High | High | Low | High | High |
| Peltola, 1986 | Low | Unclear | Unclear | High | Low | Low | Unclear |
| Prada-Arias, 2017 | Low | Unclear | Unclear | High | Low | Low | Low |



For peer review only - http://bmjopen.bmj.com/site/about/guidelines.xhtml

| | | | | | | | |
|---|---|---|---|---|---|---|---|
| Rodriguez, 2014 | High | Unclear | Low | High | Low | Low | Low |
| Salo, 2016 | High | Unclear | Unclear | High | Low | Low | Unclear |
| Samuel, 2002 | Low | Unclear | Unclear | High | Low | Low | Unclear |
| Sanchez, 1998 | Low | High | Unclear | High | Unclear | Unclear | Unclear |
| Shommu, 2018 | High | High | High | High | High | Unclear | High |
| Thompson, 2015 | Low | Unclear | High | High | Low | Unclear | High |
| Thompson, 2017 | Unclear | Unclear | High | High | Low | Unclear | High |
| Tuncer, 2019 | High | High | High | High | Low | Unclear | Unclear |
| Turkyilmaz, 2006 | Low | Unclear | Unclear | High | Low | Low | Unclear |
| Van Den Broek, 2004 | Unclear | Unclear | High | High | Low | Low | High |
| Wang, 2007 | Low | Unclear | Unclear | High | Low | Low | Unclear |
| Wu, 2012 | Unclear | High | Unclear | High | Low | High | Low |
| Yap, 2015 | Low | Unclear | Unclear | High | Low | Low | Unclear |
| Yazici, 2010 | High | Unclear | High | High | Low | Unclear | High |
| Zouari, 2016 | Low | Unclear | Unclear | High | Low | Unclear | Low |
| Zuniga, 2012 | High | Unclear | High | High | Low | Low | High |

**Supplementary Table 4 (Related to Figure 2):** Individual assessment scoring of each included study assessed by means of the QUADAS-2 tool for risk of bias.

For peer review only


For peer review only - http://bmjopen.bmj.com/site/about/guidelines.xhtml

| Marker | Threshold | Studies | Number of patients | Pooled sensitivity (95% CI) | Pooled specificity (95% CI) | Pooled LR+ (95% CI) | Pooled LR- (95% CI) | Hypothetical cohort of 1000 children with appendicitis prevalence of 40%[*] | | |
|---|---|---|---|---|---|---|---|---|---|---|
| | | | | | | | | Reduction in treatment | Missed appendicitis (FN) | Unnecessary treatment (FP) |
| CRP (mg/L) | ≤5 | 14 | 3754 | 0·82 (0·72 – 0·89) | 0·46 (0·28 – 0·66) | 1·52 (1·14 – 2·03) | 0·39 (0·30 – 0·51) | 388 | 112 | 324 |
| | 5-10 | 16 | 3170 | 0·73 (0·67 – 0·80) | 0·67 (0·58 – 0·75) | 2·21 (1·75 – 2·79) | 0·40 (0·33 – 0·50) | 510 | 108 | 198 |
| | 10-50 | 19 | 4470 | 0·53 (0·45 – 0·62) | 0·79 (0·74 – 0·83) | 2·53 (1·99 – 3·21) | 0·59 (0·49 – 0·71) | 662 | 188 | 126 |
| | ≥50 | 10 | 2654 | 0·35 (0·25 – 0·46) | 0·89 (0·87 – 0·91) | 3·25 (2·63 – 4·01) | 0·73 (0·63 – 0·84) | 794 | 260 | 66 |
| WCC (Cells/µl) | ≤10,000 | 28 | 11791 | 0·87 (0·83 – 0·89) | 0·58 (0·53 – 0·63) | 2·08 (1·87 – 2·31) | 0·23 (0·19 – 0·27) | 400 | 52 | 252 |
| | 10,000 - 15,000[†] | 30 | 9967 | 0·76 (0·72 – 0·79) | 0·70 (0·65 – 0·75) | 2·56 (2·19 – 3·00) | 0·35 (0·30 – 0·40) | 516 | 96 | 180 |
| | ≥15,000 | 14 | 6406 | 0·50 (0·44 – 0·55) | 0·84 (0·80 – 0·87) | 3·12 (2·74 – 3·55) | 0·60 (0·55 – 0·66) | 704 | 200 | 96 |
| Neutrophils (% of lymphocytes) | 65% - 80% | 13 | 3316 | 0·81 (0·74 – 0·86) | 0·64 (0·55 – 0·72) | 2·23 (1·76 – 2·82) | 0·30 (0·22 – 0·41) | 460 | 76 | 216 |
| | 75% | 9 | 1642 | 0·78 (0·73-0·82) | 0·61 (0·53-0·69) | 2·02 (1·61-2·53) | 0·36 (0·28-0·46) | 454 | 88 | 234 |
| ANC (Cells/µl) | 6600 - 10000 | 22 | 11966 | 0·85 (0·81 – 0·88) | 0·63 (0·58 – 0·68) | 2·29 (2·06 – 2·56) | 0·23 (0·19 – 0·28) | 438 | 60 | 222 |
| | <7500 | 5 | 3834 | 0·90 (0·85 – 0·94) | 0·54 (0·49 – 0·58) | 1·95 (1·79 – 2·13) | 0·18 (0·12 – 0·27) | 364 | 40 | 276 |
| | 7500 | 11 | 4730 | 0·86 (0·82 – 0·90) | 0·60 (0·54 – 0·65) | 2·14 (1·90 – 2·42) | 0·23 (0·17 – 0·30) | 416 | 56 | 240 |
| | >7500 | 5 | 3388 | 0·73 (0·70 – 0·77) | 0·77 (0·70 – 0·82) | 3·13 (2·35 – 4·16) | 0·35 (0·29 – 0·41) | 570 | 108 | 138 |
| Procalcitonin (ng/ml) | 0·1 – 0·5 | 5 | 540 | 0·31 (0·25 – 0·37) | 0·82 (0·70 – 0·90) | 1·72 (0·99 – 3·00) | 0·84 (0·74 – 0·96) | 768 | 276 | 108 |
| WCC and CRP[‡] | | 13 | 4887 | 0·68 (0·52 – 0·81) | 0·78 (0·65 – 0·88) | 3·17 (2·04 – 4·93) | 0·41 (0·27 – 0·60) | 468 | 128 | 132 |
| WCC, CRP and Neutrophils | | 6 | 1784 | 0·80 (0·35 – 0·97) | 0·79 (0·60 – 0·90) | 3·78 (2·44 – 5·85) | 0·26 (0·06 – 1·09) | 554 | 80 | 126 |
| WCC and CRP | 10,000 and 10 | 6 IPD studies | 1541 | 0·59 (0·49-0·68) | 0·79 (0·74-0·84) | 2·85 (2·43-3·35) | 0·52 (0·43-0·63) | 638 | 164 | 126 |
| WCC and CRP | 10,000 and 50 | 6 IPD studies | 1541 | 0·27 (0·19-0·37) | 0·93 (0·91-0·95) | 3·91 (2·94-5·19) | 0·78 (0·70-0·87) | 850 | 292 | 46 |

**Supplementary Table 5 (Related to Table 2):** Pooled estimates of diagnostic accuracy for blood tests used for diagnosis of appendicitis in children calculated using the bivariate model and reporting likelihood ratios([*]Median prevalence of appendicitis in the included 54 cohort studies is 40%. Sensitivity analysis was performed for all tests at all cut offs, except procalcitonin as number of studies was too low, and identified significant ($p<0.05$) effects in: [†]WCC 10,000-15,000cells/ul for case-control study design



For peer review only - http://bmjopen.bmj.com/site/about/guidelines.xhtml

(p=0·01, sensitivity 0·70[0·57-0·84] and specificity 0·49[0·31-0·66] n=3 case-control studies vs sensitivity 0·76[0·72-0·80] and specificity 0·72[0·68-0·76] n=27 non case-control studies); ‡WCC and CRP for high risk of bias in patient selection (p=0·04, sensitivity 0·86[0·73-99] and specificity 0·54[0·29-0·80] in n=4 high risk studies vs sensitivity 0·57[0·41-0·74] and specificity 0·85[0·77-0·94] in n=9 low risk studies)

For peer review only

| Marker | Studies | Threshold | Pooled sensitivity* (95% CI) | Pooled specificity* (95% CI) | Hypothetical cohort of 1000 children with appendicitis prevalence of 20%[†] | | |
|---|---|---|---|---|---|---|---|
| | | | | | Reduction in treatment | Missed appendicitis (FN) | Unnecessary treatment (FP) |
| WCC[‡] (Cells/µl) | 48 | 10,000 | 0·84 (0·80 – 0·89) | 0·59 (0·52 – 0·66) | 504 | 32 | 328 |
| | | 12,000 | 0·73 (0·66 – 0·79) | 0·71 (0·65 – 0·76) | 622 | 54 | 232 |
| | | 15,000 | 0·53 (0·45 – 0·62) | 0·82 (0·77 – 0·86) | 750 | 94 | 144 |
| CRP[§] (mg/L) | 34 | 5 | 0·77 (0·68 – 0·84) | 0·55 (0·45 – 0·64) | 486 | 46 | 360 |
| | | 10 | 0·68 (0·58 – 0·76) | 0·67 (0·58 – 0·74) | 600 | 64 | 264 |
| | | 30 | 0·49 (0·39 – 0·60) | 0·82 (0·75 – 0·87) | 758 | 102 | 144 |
| | | 50 | 0·41 (0·30 – 0·52) | 0·87 (0·81 – 0·91) | 834 | 138 | 104 |
| Neutrophils[¶] (% of lymphocytes) | 9 | 75% | 0·78 (0·73-0·82) | 0·61 (0·53-0·69) | 532 | 44 | 312 |
| ANC(Cells/µl) | 5 | <7,500 | 0·90 (0·85 – 0·94) | 0·54 (0·49 – 0·58) | 432 | 20 | 368 |
| | 11 | 7,500 | 0·86 (0·82 – 0·90) | 0·60 (0·54 – 0·65) | 508 | 28 | 320 |
| | 5 | >7,500 | 0·73 (0·70 – 0·77) | 0·77 (0·70 – 0·82) | 670 | 54 | 184 |
| Procalcitonin (ng/ml) | 5 | 0·1 – 0·5 | 0·31 (0·25 – 0·37) | 0·82 (0·70 – 0·90) | 794 | 138 | 138 |
| CRP and WCC[±] | 13 | | 0·68 (0·52 – 0·81) | 0·78 (0·65 – 0·88) | 688 | 64 | 176 |
| CRP, WCC, and Neutrophils[±] | 6 | | 0·80 (0·35 – 0·97) | 0·79 (0·60 – 0·90) | 672 | 40 | 168 |

**Supplementary Table 6 (Related to table 2):** Pooled estimates of diagnostic accuracy of makers for diagnosis of appendicitis in children (*All pooled sensitivity and specificity were obtained from the bivariate model, except for WCC and CRP for which we calculated sensitivity and specificity for selected cut offs using the Steinhauser method, comparison with bivariate mode including Likelihood ratios for all tests is demonstrated in **Supplementary Table 5**. †Median prevalence of appendicitis in the included 54 cohort studies is 40% ‡WCC was evaluated in 48 studies whereof 12 provided multiple cut offs, 4/52 studies reporting WCC provided age-dependent cut offs and could not be used in pooled analysis. §CRP was evaluated in 34 studies whereof 11 provided multiple cut offs. ¶Neutrophils evaluated in 13 studies of which 9 provided cut off at 75% ±Combinations of tests results using pooled cut-offs, calculations using provided Individual patient data is demonstrated in **Supplementary Table 5**)

For peer review only



For peer review only - http://bmjopen.bmj.com/site/about/guidelines.xhtml

**Supplementary Figure Legends:**

**Supplementary Figure 1:** Funnel plots outputs for testing individual blood tests for publication bias.(61) (Procalcitonin not shown as study numbers were low [n=4, p=0·2]. Testing was also performed for individual cut-offs of WCC and CRP with all results >0·1 [data not shown]. Y axis = inverse root of effective sample size. X axis = Odds ratio).

**Supplementary Figure 2:** Forest plot showing all studies reporting diagnostic accuracy of ANC in paediatric appendicitis. (Sensitivity and specificity for each study represented visually (blue square) with 95% confidence interval (line)).

**Supplementary Figure 3:** Forest plot showing all studies reporting diagnostic accuracy of NC (percentage) in paediatric appendicitis. (Sensitivity and specificity for each study represented visually (blue square) with 95% confidence interval (line)).

**Supplementary Figure 4:** Forest plot showing all studies reporting diagnostic accuracy of procalcitonin in paediatric appendicitis. (Sensitivity and specificity for each study represented visually (blue square) with 95% confidence interval (line)).

**Supplementary Figure 5:** Forest plot showing all studies reporting diagnostic accuracy of WCC and CRP combination in paediatric appendicitis. (Sensitivity and specificity for each study represented visually (blue square) with 95% confidence interval (line)).

**Supplementary Figure 6:** Forest plot showing all studies reporting diagnostic accuracy of WCC, CRP and NC combination in paediatric appendicitis. (Sensitivity and specificity for each study represented visually (blue square) with 95% confidence interval (line)).

For peer review only


For peer review only - http://bmjopen.bmj.com/site/about/guidelines.xhtml

**Search Strategy:**
Searches were performed on the 4th June 2019 with search terms that had been informed by scoping searches. Databases searched included Cochrane Database of Systematic Reviews, Cochrane Central Register of controlled trials, Database of abstracts of Reviews of Effects, Embase, Ovid(Medline) Epub Ahead of print in process and other Non-Indexed Citations, Ovid Medline(R) Daily, Ovid Medline(R) and Science Citation Index and Conference Proceedings Citation Index (SCIENCE). These databases were searched through the interfaces Cochrane Library(Wiley), OvidSP and Web of Science Core Collection. Specific terms used for these interfaces are detailed below, following searching all results were merged and checked for duplicates before review (**Figure 1**).

For peer review only

**Medline**:

| # | Searches | Results |
|---|---|---|
| 1 | adolescent/ or exp child/ or exp infant/ | 3410061 |
| 2 | (child* or schoolchild* or preschool* or pre-school* or boys or girls or infant? or pediatric* or paediatric* or adolescen* or teen* or youth?).ti,ab. | 1876871 |
| 3 | 1 or 2 | 3876495 |
| 4 | exp Leukocyte Count/ or leukocytes/ or neutrophils/ | 225318 |
| 5 | (leukocyte? or white cell or white blood cell or neutrophil*).ti,ab. | 272998 |
| 6 | (wbcc or wcc).ti,ab. | 655 |
| 7 | C-Reactive Protein/ | 42976 |
| 8 | (c-reactive protein? or crp).ti,ab. | 75114 |
| 9 | Calcitonin/ | 15622 |
| 10 | (procalcitonin or calcitonin or pct).ti,ab. | 32717 |
| 11 | 4 or 5 or 6 or 7 or 8 or 9 or 10 | 490743 |
| 12 | Appendicitis/ | 18470 |
| 13 | Appendectomy/ | 10586 |
| 14 | (appendix or appendicitis or appendectom* or appendicectom*).ti,ab. | 33426 |
| 15 | 12 or 13 or 14 | 38889 |
| 16 | 3 and 11 and 15 | 787 |

**Embase:**

| # | Searches | Results |
|---|---|---|
| 1 | juvenile/ or exp adolescent/ or exp child/ | 3172209 |
| 2 | (child* or schoolchild* or preschool* or pre-school* or boys or girls or infant? or pediatric* or paediatric* or adolescen* or teen* or youth?).ti,ab. | 2298797 |
| 3 | 1 or 2 | 3780916 |
| 4 | exp leukocyte count/ or leukocyte/ or neutrophil/ | 394999 |
| 5 | (leukocyte? or white cell or white blood cell or neutrophil*).ti,ab. | 364068 |
| 6 | (wbcc or wcc).ti,ab. | 1603 |
| 7 | C reactive protein/ | 158251 |
| 8 | (c-reactive protein? or crp).ti,ab. | 129235 |
| 9 | Procalcitonin/ | 10738 |
| 10 | (procalcitonin or calcitonin or pct).ti,ab. | 43836 |


For peer review only - http://bmjopen.bmj.com/site/about/guidelines.xhtml

| | | |
|---|---|---|
| 11 | 4 or 5 or 6 or 7 or 8 or 9 or 10 | 749518 |
| 12 | exp appendicitis/ | 22927 |
| 13 | appendectomy/ | 18892 |
| 14 | (appendix or appendicitis or appendectom* or appendicectom*).ti,ab. | 37059 |
| 15 | 12 or 13 or 14 | 46062 |
| 16 | 3 and 11 and 15 | 1369 |

**Cochrane:**

| # | Searches |
|---|---|
| 1 | MeSH descriptor: [Adolescent] explode all trees |
| 2 | MeSH descriptor: [Child] explode all trees |
| 3 | MeSH descriptor: [Infant] explode all trees |
| 4 | exp leukocyte count/ or leukocyte/ or neutrophil/ |
| 5 | child* or schoolchild* or preschool* or pre-school* or boys or girls or infant? or pediatric* or paediatric* or adolescen* or teen* or youth?:ti,ab,kw (Word variations have been searched) |
| 6 | #1 or #2 or #3 or #4 |
| 7 | MeSH descriptor: [Leukocyte Count] explode all trees |
| 8 | MeSH descriptor: [Leukocytes] this term only |
| 9 | MeSH descriptor: [Neutrophils] explode all trees |
| 10 | wbcc or wcc:ti,ab,kw (Word variations have been searched) |
| 11 | MeSH descriptor: [C-Reactive Protein] explode all trees |
| 12 | c-reactive protein* or crp:ti,ab,kw (Word variations have been searched) |
| 13 | MeSH descriptor: [Calcitonin] explode all trees |
| 14 | procalcitonin or calcitonin or pct:ti,ab,kw |
| 15 | #6 or #7 or #8 or #9 or #10 or #11 or #12 or #13 or #14 |
| 16 | MeSH descriptor: [Appendicitis] explode all trees |
| 17 | MeSH descriptor: [Appendectomy] explode all trees |
| 18 | appendix or appendicitis or appendectom* or appendicectom*:ti,ab,kw (Word variations have been searched) |
| 19 | #16 or #17 or #18 |
| 20 | #5 and #15 and #19 |

**Web of Science:**

| # | Searches | Results |
|---|---|---|
| 1 | TS=(child* or schoolchild* or preschool* or pre-school* or boys or girls or infant? or pediatric* or paediatric* or adolescen* or teen* or youth?) | 1709598 |
| 2 | TS=(leukocyte* or "white cell" or "white blood cell" or neutrophil*) OR TS=("c-reactive protein" OR "c reactive protein" OR crp) OR TS=(procalcitonin OR calcitonin OR pct) | 433876 |
| 3 | TS=(appendix or appendicitis or appendectom* or appendicectom*) | 33245 |
| 4 | #3 AND #2 AND #1 | 397 |


For peer review only - http://bmjopen.bmj.com/site/about/guidelines.xhtml

**QUADAS-2 Scoring criteria**

Below is an overview, with examples of how the QUADAS-2 scoring criteria was used to measure risk of bias and applicability in selected studies(62). In those patients not undergoing surgery the optimal reference standard was follow up after 2 weeks to confirm absence of disease. Studies including only patients who had undergone a radiological investigation were included but this caveat was noted when assessing these studies for risk of bias. Study quality was not an exclusion criteria.

Domain 1: Patient Selection

- Was a consecutive or random sample of patients enrolled? (Yes/Unclear/No)
  - *Scored yes if the words in article state "consecutive", "random", or "all patients were included*
- Was a case-control design avoided? (Yes/Unclear/No)
  - *Scored yes if article stated the study design as not being case-control*
- Did the study avoid inappropriate exclusions? (Yes/Unclear/No)
  - *Scored yes if studies excluded no children with abdominal pain or suspected appendicitis.*
- (Risk) Could the selection of patients have introduced bias? (Low / Unclear/ High)
  - *Scored low if all answers to signalling questions were yes. Score high if any were answered no. Score unclear if any were answered as unclear with remainder scoring low.*
- (Concern) Is there concern that included patients do not match the review question? (Low / Unclear /High)
  - *Scored low if all patients were <18 years, had symptoms in keeping with appendicitis and no previous diagnosis of appendicitis*

Domain 2: Index Test

- Were the index tests interpreted without knowledge of the results of the reference standard? (Yes / Unclear/ No)
  - *Scored yes if text stated blood test results were concealed or blinded to clinician or pathologist.*
- If a threshold was used, was it pre-specified? (Yes / Unclear/ No)
  - *Scored yes if a cut-off for index test was stated before commencing study or analysing results*
- (Risk) Could the conduct or interpretation of the index test have introduced bias? (Low / Unclear/ High)
  - *Scored low if all answers to signalling questions were yes. Score high if any were answered no. Score unclear if any were answered as unclear with remainder scoring low.*
- (Concern) Is there concern that the index test, its conduct, or interpretation differ from the review question? (Low / Unclear / High)
  - *Scored low if the study provides a clear description of the index test and a clear description or definition of a positive test result*

Domain 3: Reference Standard

- Is the reference standard likely to correctly classify the target condition? (Yes / Unclear/ No)
  - *Score yes if the reference standard was histology after surgery or clinical follow up of patient after 2 weeks. Score no if the reference standard was surgery alone without histology.*
- Were the reference standard results interpreted without knowledge of the results of the index test? (Yes / Unclear/ No)
  - *Score yes if study states the assessment of reference standard was done without knowledge or with blinding of the results of the index test*
- (Risk) Could the reference standard, its conduct, or its interpretation have introduced bias? (Low / Unclear/ High)
  - *Scored low if all answers to signalling questions were yes. Score high if any were answered no. Score unclear if any were answered as unclear with remainder scoring low.*
- (Concern) Is there concern that the target condition as defined by the reference standard does not match the review question? (Low / Unclear/ High)
  - *Score Low if all patients were in keeping with the target condition of appendicitis as defined by histology after surgery or refuted by clinical follow-up of greater than or equal to 2 weeks*

Domain 4: Flow and Timing

- Was there an appropriate interval between index test(s) and reference standard? (Yes / Unclear/ No)
  - *Score yes if the time period was 48 hours or less and was stated in text; Score no if the time period was more than 48 hours. Score unclear if insufficient information to determine the interval between index test and reference standard.*


For peer review only - http://bmjopen.bmj.com/site/about/guidelines.xhtml
For peer review only

- Did all patients receive a reference standard? (Yes / Unclear/ No)
    - *Score yes if all patients received either appendicectomy with histology or clinical follow up of greater than or equal to 2 weeks to refute appendicitis*
- Did patients receive the same reference standard? (Yes / Unclear/ No)
    - *Score yes if all patients received the same reference standard.*
- Were all patients included in the analysis? (Yes / Unclear/ No)
    - *Score yes if all patients included at patient selection underwent index test and reference standard.*
- (Risk) Could the patient flow have introduced bias? (Low / Unclear/ High)
    - *Scored low if all answers to signalling questions were yes. Score high if any were answered no. Score unclear if any were answered as unclear with remainder scoring low.*

For peer review only

## Supplementary References

1. Altali K, Ruiz-Artacho P, Trenchs V, Martinez Ortiz de Zarate M, Navarro C, Fernandez C, et al. [Hospital emergency room diagnosis of acute appendicitis in patients aged 2 to 20 years: the INFURG-SEMES score from the emergency infections study of the Spanish Society of Emergency Medicine]. Emergencias : revista de la Sociedad Espanola de Medicina de Emergencias. 2017;29(4):231-6.
2. Anandalwar SP, Callahan MJ, Bachur RG, Feng C, Sidhwa F, Karki M, et al. Use of White Blood Cell Count and Polymorphonuclear Leukocyte Differential to Improve the Predictive Value of Ultrasound for Suspected Appendicitis in Children. Journal of the American College of Surgeons. 2015;220(6):1010-7.
3. Andersson RE, Hugander AP, Ghazi SH, Ravn H, Offenbartl SK, Nystrom PO, et al. Diagnostic value of disease history, clinical presentation, and inflammatory parameters of appendicitis. World J Surg. 1999;23(2):133-40.
4. Andersson M, Andersson RE. The appendicitis inflammatory response score: a tool for the diagnosis of acute appendicitis that outperforms the Alvarado score. World J Surg. 2008;32(8):1843-9.
5. Andersson M, Rubér M, Ekerfelt C, Hallgren HB, Olaison G, Andersson RE. Can New Inflammatory Markers Improve the Diagnosis of Acute Appendicitis? World Journal of Surgery. 2014;38(11):2777-83.
6. Andersson M, Kolodziej B, Andersson RE. Randomized clinical trial of Appendicitis Inflammatory Response score-based management of patients with suspected appendicitis. The British journal of surgery. 2017;104(11):1451-61.
7. Bachur RG, Dayan PS, Dudley NC, Bajaj L, Stevenson MD, Macias CG, et al. The Influence of Age on the Diagnostic Performance of White Blood Cell Count and Absolute Neutrophil Count in Suspected Pediatric Appendicitis. Academic emergency medicine : official journal of the Society for Academic Emergency Medicine. 2016;23(11):1235-42.
8. Bal A, Anil M, Narturk M, Ozdemir T, Arikan A, Koyluoglu G, et al. Importance of Clinical Decision Making by Experienced Pediatric Surgeons When Children Are Suspected of Having Acute Appendicitis: The Reality in a High-Volume Pediatric Emergency Department. Pediatr Emerg Care. 2017;33(9):e38-e42.
9. BELTRÁN A M, ALMONACID F J, GUTIÉRREZ C J, CRUCES B K. Puntuación diagnóstica de apendicitis aguda en niños realizada por pediatras de las Unidades de Emergencia. Revista chilena de pediatría. 2007;78:584-91.
10. Benito J, Acedo Y, Medrano L, Barcena E, Garay RP, Arri EA. Usefulness of new and traditional serum biomarkers in children with suspected appendicitis. The American journal of emergency medicine. 2016;34(5):871-6.
11. Calvo Rigual F, Sendra Esteve S, Mialaret Lahiguera A, Montagud Beltrán E, Llanes Domingo S, Medrano González J. [The value of C-reactive protein in the diagnosis of acute appendicitis in children]. An Esp Pediatr. 1998;48(4):376-80.
12. Cayrol J, Miguez MC, Guerrero G, Tomatis C, Simal I, Maranon R. Diagnostic accuracy and prognostic utility of D Dimer in acute appendicitis in children. European journal of pediatrics. 2016;175(3):313-20.
13. Corkum KS, Oyetunji TA, Grabowski JE, Rigsby CK, Lautz TB. Absolute neutrophil count as a diagnostic guide for the use of MRI in the workup of suspected appendicitis in children. Journal of pediatric surgery. 2019;54(7):1359-64.
14. Doraiswamy NV. Leucocyte counts in the diagnosis and prognosis of acute appendicitis in children. The British journal of surgery. 1979;66(11):782-4.
15. Esparaz JR, McGovern GC, Mowrer AR, Nierstedt RT, Biesboer EA, Elger BM, et al. A simple algorithm to improve quality while reducing resource utilization in evaluation of suspected appendicitis in children. American journal of surgery. 2019;217(3):469-72.
16. Fleischman RJ, Devine MK, Yagapen MA, Steichen AJ, Hansen ML, Zigman AF, et al. Evaluation of a novel pediatric appendicitis pathway using high- and low-risk scoring systems. Pediatr Emerg Care. 2013;29(10):1060-5.
17. Goldman RD, Carter S, Stephens D, Antoon R, Mounstephen W, Langer JC. Prospective validation of the pediatric appendicitis score. The Journal of pediatrics. 2008;153(2):278-82.
18. Gonzalez Del Castillo J, Ayuso FJ, Trenchs V, Martinez Ortiz de Zarate M, Navarro C, Altali K, et al. Diagnostic accuracy of the APPY1 Test in patients aged 2-20 years with suspected acute appendicitis presenting to emergency departments. Emergency medicine journal : EMJ. 2016;33(12):853-9.
19. Groselj-Grenc M, Repse S, Dolenc-Strazar Z, Hojker S, Derganc M. Interleukin-6 and lipopolysaccharide-binding protein in acute appendicitis in children. Scandinavian journal of clinical and laboratory investigation. 2007;67(2):197-206.
20. Hsiao KH, Lin LH, Chen DF. Application of the MANTRELS scoring system in the diagnosis of acute appendicitis in children. Acta paediatrica Taiwanica = Taiwan er ke yi xue hui za zhi. 2005;46(3):128-31.


For peer review only - http://bmjopen.bmj.com/site/about/guidelines.xhtml

21. Huckins DS, Simon HK, Copeland K, Spiro DM, Gogain J, Wandell M. A novel biomarker panel to rule out acute appendicitis in pediatric patients with abdominal pain. The American journal of emergency medicine. 2013;31(9):1368-75.
22. Huckins DS, Simon HK, Copeland K, Milling TJ, Jr., Spandorfer PR, Hennes H, et al. Prospective validation of a biomarker panel to identify pediatric ED patients with abdominal pain who are at low risk for acute appendicitis. The American journal of emergency medicine. 2016;34(8):1373-82.
23. Kaiser M, Schroeckenfuchs M, Castellani C, Warncke G, Till H, Singer G. The diagnostic value of hepcidin to predict the presence and severity of appendicitis in children. The Journal of surgical research. 2018;222:102-7.
24. Khan AN, Sawan A, Likourezos A, Schnellinger M, Garcia E. The usefulness of procalcitonin in the diagnosis of appendicitis in children: a pilot study. Emergency medicine international. 2012;2012:317504.
25. Khanafer I, Martin DA, Mitra TP, Eccles R, Brindle ME, Nettel-Aguirre A, et al. Test characteristics of common appendicitis scores with and without laboratory investigations: a prospective observational study. BMC Pediatr. 2016;16(1):147.
26. Kharbanda AB, Taylor GA, Fishman SJ, Bachur RG. A clinical decision rule to identify children at low risk for appendicitis. Pediatrics. 2005;116(3):709-16.
27. Kharbanda AB, Rai AJ, Cosme Y, Liu K, Dayan PS. Novel serum and urine markers for pediatric appendicitis. Academic emergency medicine : official journal of the Society for Academic Emergency Medicine. 2012;19(1):56-62.
28. Kharbanda AB, Dudley NC, Bajaj L, Stevenson MD, Macias CG, Mittal MK, et al. Validation and refinement of a prediction rule to identify children at low risk for acute appendicitis. Archives of pediatrics & adolescent medicine. 2012;166(8):738-44.
29. Ko YS, Lin LH, Chen DF. Laboratory aid and ultrasonography in the diagnosis of appendicitis in children. Zhonghua Minguo xiao er ke yi xue hui za zhi [Journal] Zhonghua Minguo xiao er ke yi xue hui. 1995;36(6):415-9.
30. Kouame DB, Garrigue MA, Lardy H, Machet MC, Giraudeau B, Robert M. [Is procalcitonin able to help in pediatric appendicitis diagnosis?]. Annales de chirurgie. 2005;130(3):169-74.
31. Kwan KY, Nager AL. Diagnosing pediatric appendicitis: usefulness of laboratory markers. The American journal of emergency medicine. 2010;28(9):1009-15.
32. Lin CH, Chen JH, Li TC, Ho YJ, Lin WC. Children presenting at the emergency department with right lower quadrant pain. The Kaohsiung journal of medical sciences. 2009;25(1):1-9.
33. Lycopoulou L, Mamoulakis C, Hantzi E, Demetriadis D, Antypas S, Giannaki M, et al. Serum amyloid A protein levels as a possible aid in the diagnosis of acute appendicitis in children. Clinical chemistry and laboratory medicine. 2005;43(1):49-53.
34. Malia L, Sturm JJ, Smith SR, Brown RT, Campbell B, Chicaiza H. Predictors for Acute Appendicitis in Children. Pediatr Emerg Care. 2019.
35. Mandeville K, Pottker T, Bulloch B, Liu J. Using appendicitis scores in the pediatric ED. The American journal of emergency medicine. 2011;29(9):972-7.
36. Miguez C, Tomatis Souverbielle C, Haro A, Guerrero G, Perez-Egido L, Garcia-Gamiz M, et al. Evaluation of proadrenomedullin as a diagnostic or prognostic biomarker of acute appendicitis in children. The American journal of emergency medicine. 2016;34(12):2298-305.
37. Mohammed AA, Daghman NA, Aboud SM, Oshibi HO. The diagnostic value of C-reactive protein, white blood cell count and neutrophil percentage in childhood appendicitis. Saudi medical journal. 2004;25(9):1212-5.
38. Diaz Moreno E, Garcia Gomez M, Castejon Casado J, Liceras Liceras E, Martin Cano F, Munoz Miguelsanz MA. [Analysis of the medical decision in abdominal pain suggestive of acute appendicitis]. Cirugia pediatrica : organo oficial de la Sociedad Espanola de Cirugia Pediatrica. 2012;25(1):40-5.
39. Naqvi SA, Thompson GC, Joffe AR, Blackwood J, Martin DA, Brindle M, et al. Cytokines and Chemokines in Pediatric Appendicitis: A Multiplex Analysis of Inflammatory Protein Mediators. Mediators of inflammation. 2019;2019:2359681.
40. Oikonomopoulou N, Miguez-Navarro C, Rivas-Garcia A, Garcia Gamiz M, Lopez-Lopez R, Oliver-Saez P, et al. Assessment of proadrenomedullin as diagnostic or prognostic biomarker of acute appendicitis in children with acute abdominal pain. The American journal of emergency medicine. 2019;37(7):1289-94.
41. Ovrebo KK, Eckerbom RM, Haram S, Rokke O. [Acute abdomen among children and adolescents. A retrospective study of 470 children and adolescents with acute abdominal pain]. Tidsskrift for den Norske laegeforening : tidsskrift for praktisk medicin, ny raekke. 1993;113(26):3244-7.
42. Ozguner I, Kizilgun M, Karaman A, Cavusoglu YH, Erdogan D, Karaman I, et al. Are neutrophil CD64 expression and interleukin-6 early useful markers for diagnosis of acute appendicitis? European journal of pediatric surgery : official journal of Austrian Association of Pediatric Surgery [et al] = Zeitschrift fur Kinderchirurgie. 2014;24(2):179-83.

For peer review only


For peer review only - http://bmjopen.bmj.com/site/about/guidelines.xhtml

43. Peltola H, Ahlqvist J, Rapola J, Rasanen J, Louhimo I, Saarinen M, et al. C-reactive protein compared with white blood cell count and erythrocyte sedimentation rate in the diagnosis of acute appendicitis in children. Acta chirurgica Scandinavica. 1986;152:55-8.
44. Prada-Arias M, Vazquez JL, Salgado-Barreira A, Gomez-Veiras J, Montero-Sanchez M, Fernandez-Lorenzo JR. Diagnostic accuracy of fibrinogen to differentiate appendicitis from nonspecific abdominal pain in children. The American journal of emergency medicine. 2017;35(1):66-70.
45. A. Rodríguez Sánchez de la Blanca RMP, J. Lorente Romero, A. Rivas García, P. Vázquez López, C. Míguez Navarro. [Usefulness of procalcitonin in early diagnosis of acute appendicitis]. Acta Pediatr Esp. 2014;72(7):4.
46. Salo M, Roth B, Stenstrom P, Arnbjornsson E, Ohlsson B. Urinary biomarkers in pediatric appendicitis. Pediatric surgery international. 2016;32(8):795-804.
47. Samuel M. Pediatric appendicitis score. Journal of pediatric surgery. 2002;37(6):877-81.
48. Sanchez Echaniz J, Luis Garcia M, Vazquez Ronco MA, Mintegui Raso S, Benito Fernandez J, Lopez Alvarez-Buhilla P. [Diagnostic value of reactive C protein in suspected acute appendicitis in children]. An Esp Pediatr. 1998;48(5):470-4.
49. Shommu NS, Jenne CN, Blackwood J, Martin DA, Joffe AR, Eccles R, et al. The Use of Metabolomics and Inflammatory Mediator Profiling Provides a Novel Approach to Identifying Pediatric Appendicitis in the Emergency Department. Scientific reports. 2018;8(1):4083.
50. Thompson GC, Schuh S, Gravel J, Reid S, Fitzpatrick E, Turner T, et al. Variation in the Diagnosis and Management of Appendicitis at Canadian Pediatric Hospitals. Academic emergency medicine : official journal of the Society for Academic Emergency Medicine. 2015;22(7):811-22.
51. Thompson GC, Morrison E, Ross M, Liu H, Vanderkooi OG, Eccles R. The Use of Routine Blood Cultures in Pediatric Appendicitis. Pediatr Emerg Care. 2017;33(12):e160-e3.
52. Tuncer AA, Cavus S, Balcioglu A, Silay S, Demiralp I, Calkan E, et al. Can mean platelet volume, Neutrophil-to-Lymphocyte, Lymphocyte-to-Monocyte, Platelet-to-Lymphocyte ratios be favourable predictors for the differential diagnosis of appendicitis? JPMA The Journal of the Pakistan Medical Association. 2019;69(5):647-54.
53. Turkyilmaz Z, Sonmez K, Karabulut R, Elbeg S, Moralioglu S, Demirtola A, et al. Sequential cytokine levels in the diagnosis of appendicitis. Scandinavian journal of clinical and laboratory investigation. 2006;66(8):723-31.
54. van den Broek WT, van der Ende ED, Bijnen AB, Breslau PJ, Gouma DJ. Which children could benefit from additional diagnostic tools in case of suspected appendicitis? Journal of pediatric surgery. 2004;39(4):570-4.
55. Wang LT, Prentiss KA, Simon JZ, Doody DP, Ryan DP. The use of white blood cell count and left shift in the diagnosis of appendicitis in children. Pediatr Emerg Care. 2007;23(2):69-76.
56. Wu HP, Chen CY, Kuo IT, Wu YK, Fu YC. Diagnostic values of a single serum biomarker at different time points compared with Alvarado score and imaging examinations in pediatric appendicitis. The Journal of surgical research. 2012;174(2):272-7.
57. Yap TL, Chen Y, Low WW, Ong CC, Nah SA, Jacobsen AS, et al. A new 2-step risk-stratification clinical score for suspected appendicitis in children. Journal of pediatric surgery. 2015;50(12):2051-5.
58. Yazici M, Ozkisacik S, Oztan MO, Gursoy H. Neutrophil/lymphocyte ratio in the diagnosis of childhood appendicitis. The Turkish journal of pediatrics. 2010;52(4):400-3.
59. Zouari M, Jallouli M, Louati H, Kchaou R, Chtourou R, Kotti A, et al. Predictive value of C-reactive protein, ultrasound and Alvarado score in acute appendicitis: a prospective pediatric cohort. The American journal of emergency medicine. 2016;34(2):189-92.
60. Zuniga RV, Arribas JL, Montes SP, Fernandez MN, Abad CG, Martin LG, et al. Application of Pediatric Appendicitis Score on the emergency department of a secondary level hospital. Pediatr Emerg Care. 2012;28(6):489-92.
61. Deeks JJ, Macaskill P, Irwig L. The performance of tests of publication bias and other sample size effects in systematic reviews of diagnostic test accuracy was assessed. Journal of Clinical Epidemiology. 2005;58(9):882-93.
62. Whiting PF, Rutjes AWS, Westwood ME, Mallett S, Deeks JJ, Reitsma JB, et al. QUADAS-2: A Revised Tool for the Quality Assessment of Diagnostic Accuracy Studies. Annals of Internal Medicine. 2011;155(8):529-36.


For peer review only - http://bmjopen.bmj.com/site/about/guidelines.xhtml

| Study | TP | FP | FN | TN | Threshold | Sensitivity (95% CI) | Specificity (95% CI) |
|---|---|---|---|---|---|---|---|
| Salo 2016 | 16 | 7 | 6 | 15 | age dependent | 0.73 [0.50, 0.89] | 0.68 [0.45, 0.86] |
| Altali 2017 | 99 | 85 | 17 | 130 | 6.6 | 0.85 [0.78, 0.91] | 0.60 [0.54, 0.67] |
| Kharbanda 2012 | 942 | 824 | 76 | 783 | 6.73 | 0.93 [0.91, 0.94] | 0.49 [0.46, 0.51] |
| Fleischman 2013 | 53 | 56 | 12 | 57 | 6.75 | 0.82 [0.70, 0.90] | 0.50 [0.41, 0.60] |
| Kharbanda 2005 | 152 | 131 | 5 | 137 | 6.75 | 0.97 [0.93, 0.99] | 0.51 [0.45, 0.57] |
| Prada Arias 2017 | 119 | 57 | 13 | 86 | 7.0 | 0.90 [0.84, 0.95] | 0.60 [0.52, 0.68] |
| Andersson 1999 | 34 | 38 | 2 | 61 | 7.5 | 0.94 [0.81, 0.99] | 0.62 [0.51, 0.71] |
| Andersson 2008 | 8 | 27 | 1 | 33 | 7.5 | 0.89 [0.52, 1.00] | 0.55 [0.42, 0.68] |
| Andersson 2014 | 35 | 32 | 3 | 41 | 7.5 | 0.92 [0.79, 0.98] | 0.56 [0.44, 0.68] |
| Andersson 2017 | 274 | 282 | 31 | 322 | 7.5 | 0.90 [0.86, 0.93] | 0.53 [0.49, 0.57] |
| Benito 2016 | 78 | 37 | 11 | 59 | 7.5 | 0.88 [0.79, 0.94] | 0.61 [0.51, 0.71] |
| Goldman 2008 | 84 | 67 | 17 | 121 | 7.5 | 0.83 [0.74, 0.90] | 0.64 [0.57, 0.71] |
| Samuel 2002 | 595 | 122 | 139 | 314 | 7.5 | 0.81 [0.78, 0.84] | 0.72 [0.68, 0.76] |
| Shommu 2018 | 40 | 24 | 6 | 57 | 7.5 | 0.87 [0.74, 0.95] | 0.70 [0.59, 0.80] |
| Thompson 2015 | 351 | 68 | 47 | 45 | 7.5 | 0.88 [0.85, 0.91] | 0.40 [0.31, 0.49] |
| Thompson 2017 | 319 | 206 | 141 | 457 | 7.5 | 0.69 [0.65, 0.74] | 0.69 [0.65, 0.72] |
| Zuniga 2012 | 27 | 37 | 1 | 36 | 7.5 | 0.96 [0.82, 1.00] | 0.49 [0.37, 0.61] |
| Corkum 2018 | 116 | 166 | 40 | 416 | 8.0 | 0.74 [0.67, 0.81] | 0.71 [0.68, 0.75] |
| Bal 2016 | 43 | 13 | 13 | 27 | 9.4 | 0.77 [0.64, 0.87] | 0.68 [0.51, 0.81] |
| Miguez 2016 | 37 | 16 | 7 | 76 | 9.6 | 0.84 [0.70, 0.93] | 0.83 [0.73, 0.90] |
| Bachur 2016 | 630 | 365 | 240 | 898 | 10.0 | 0.72 [0.69, 0.75] | 0.71 [0.69, 0.74] |
| Oikonomopoulou 2018 | 71 | 26 | 29 | 159 | 10.8 | 0.71 [0.61, 0.80] | 0.86 [0.80, 0.91] |

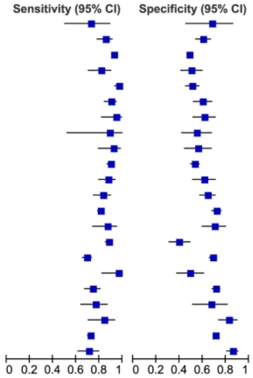


209x279mm (300 x 300 DPI)

| Study | TP | FP | FN | TN | Threshold | Sensitivity (95% CI) | Specificity (95% CI) |
|---|---|---|---|---|---|---|---|
| Anadalwar 2015 | 340 | 241 | 53 | 211 | 65% | 0.87 [0.83, 0.90] | 0.47 [0.42, 0.51] |
| Salo 2016 | 16 | 10 | 6 | 12 | 71% | 0.73 [0.50, 0.89] | 0.55 [0.32, 0.76] |
| Andersson 1999 | 31 | 45 | 5 | 54 | 75% | 0.86 [0.71, 0.95] | 0.55 [0.44, 0.65] |
| Andersson 2008 | 9 | 29 | 3 | 31 | 75% | 0.75 [0.43, 0.95] | 0.52 [0.38, 0.65] |
| Andersson 2014 | 30 | 29 | 8 | 44 | 75% | 0.79 [0.63, 0.90] | 0.60 [0.48, 0.72] |
| Fleischman 2013 | 50 | 48 | 15 | 65 | 75% | 0.77 [0.65, 0.86] | 0.58 [0.48, 0.67] |
| Hsiao 2005 | 87 | 67 | 24 | 44 | 75% | 0.78 [0.70, 0.86] | 0.40 [0.30, 0.49] |
| Ko 1995 | 22 | 6 | 5 | 14 | 75% | 0.81 [0.62, 0.94] | 0.70 [0.46, 0.88] |
| Mandeville 2011 | 119 | 48 | 36 | 84 | 75% | 0.77 [0.69, 0.83] | 0.64 [0.55, 0.72] |
| Mohammed 2004 | 111 | 15 | 19 | 71 | 75% | 0.85 [0.78, 0.91] | 0.83 [0.73, 0.90] |
| Yap 2015 | 79 | 80 | 40 | 175 | 75% | 0.66 [0.57, 0.75] | 0.69 [0.63, 0.74] |
| Wang 2007 | 44 | 34 | 30 | 302 | 80% | 0.59 [0.47, 0.71] | 0.90 [0.86, 0.93] |
| Doraiswamy 1979 | 216 | 47 | 9 | 103 | age dependent | 0.96 [0.93, 0.98] | 0.69 [0.61, 0.76] |

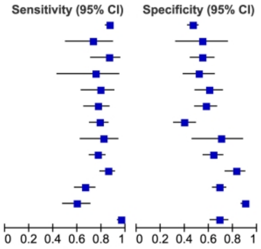


209x279mm (300 x 300 DPI)

| Study | TP | FP | FN | TN | Threshold | Sensitivity (95% CI) | Specificity (95% CI) |
|---|---|---|---|---|---|---|---|
| Benito 2016 | 34 | 34 | 55 | 62 | 0.1 | 0.38 [0.28, 0.49] | 0.65 [0.54, 0.74] |
| Bal 2016 | 18 | 4 | 38 | 36 | 0.37 | 0.32 [0.20, 0.46] | 0.90 [0.76, 0.97] |
| Khan 2012 | 6 | 2 | 16 | 26 | 0.39 | 0.27 [0.11, 0.50] | 0.93 [0.76, 0.99] |
| Rodriguez Sanchez 2013 | 10 | 18 | 34 | 46 | 0.5 | 0.23 [0.11, 0.38] | 0.72 [0.59, 0.82] |
| Kouame 2005 | 19 | 4 | 49 | 29 | 0.5 | 0.28 [0.18, 0.40] | 0.88 [0.72, 0.97] |

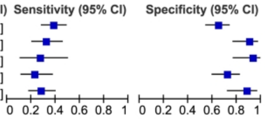


209x279mm (300 x 300 DPI)

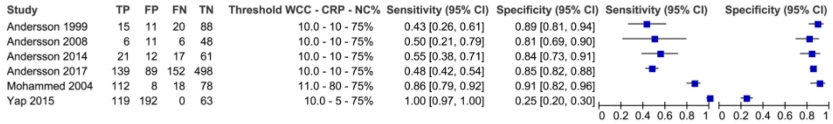


209x279mm (300 x 300 DPI)

BMJ Open

# Diagnostic accuracy of blood tests of inflammation in paediatric appendicitis – a systematic review and meta-analysis

| | |
|---|---|
| Journal: | *BMJ Open* |
| Manuscript ID | bmjopen-2021-056854.R1 |
| Article Type: | Original research |
| Date Submitted by the Author: | 01-Aug-2022 |
| Complete List of Authors: | Fawkner-Corbett, David; Weatherall Institute of Molecular Medicine, ; University of Oxford Nuffield Department of Surgical Sciences, Hayward, Gail; University of Oxford, Nuffield Department of Primary Care Health Sciences Alkhmees, Mohammed; University of Groningen, Department of General Practice and Elderly Care Medicine Van Den Bruel, Ann; KU Leuven Ordóñez-Mena, JM; University of Oxford, Nuffield Department of Primary Care Health Sciences Holtman, G.; University Medical Centre Groningen, Department of General Practice and Elderly Care Medicine |
| <b>Primary Subject Heading</b>: | Surgery |
| Secondary Subject Heading: | Surgery, Paediatrics, Evidence based practice |
| Keywords: | PAEDIATRIC SURGERY, PAEDIATRICS, ACCIDENT & EMERGENCY MEDICINE |
| | |

SCHOLARONE™
Manuscripts

*I, the Submitting Author has the right to grant and does grant on behalf of all authors of the Work (as defined in the below author licence), an exclusive licence and/or a non-exclusive licence for contributions from authors who are: i) UK Crown employees; ii) where BMJ has agreed a CC-BY licence shall apply, and/or iii) in accordance with the terms applicable for US Federal Government officers or employees acting as part of their official duties; on a worldwide, perpetual, irrevocable, royalty-free basis to BMJ Publishing Group Ltd ("BMJ") its licensees and where the relevant Journal is co-owned by BMJ to the co-owners of the Journal, to publish the Work in this journal and any other BMJ products and to exploit all rights, as set out in our licence.*

*The Submitting Author accepts and understands that any supply made under these terms is made by BMJ to the Submitting Author unless you are acting as an employee on behalf of your employer or a postgraduate student of an affiliated institution which is paying any applicable article publishing charge ("APC") for Open Access articles. Where the Submitting Author wishes to make the Work available on an Open Access basis (and intends to pay the relevant APC), the terms of reuse of such Open Access shall be governed by a Creative Commons licence – details of these licences and which Creative Commons licence will apply to this Work are set out in our licence referred to above.*

*Other than as permitted in any relevant BMJ Author's Self Archiving Policies, I confirm this Work has not been accepted for publication elsewhere, is not being considered for publication elsewhere and does not duplicate material already published. I confirm all authors consent to publication of this Work and authorise the granting of this licence.*

For peer review only

**Title:**

Diagnostic accuracy of blood tests of inflammation in paediatric appendicitis – a systematic review and meta-analysis

**Authors names:**

David Fawkner-Corbett (0000-0002-5273-3682), Gail N Hayward (0000-0003-0852-627X), Mohammed Alkhmees (0000-0002-8608-6938), Ann Van den Bruel (000-0001-9012-2009), Jose M Ordóñez-Mena (0000-0002-8965-103X), Gea A Holtman (0000-0001-6579-767X)

**Address and positions for each author:**

NIHR Community Healthcare MedTech and IVD Co-operative, Nuffield Department of Primary Care Health Sciences, University of Oxford, Oxford OX2 6GG, United Kingdom; Academic Paediatric Surgery Unit, Nuffield Department of Surgical Sciences, University of Oxford, OX3 9DU, Oxford, United Kingdom; MRC Weatherall Institute of Molecular Medicine, Radcliffe Department of Medicine, University of Oxford, OX3 9DS, Oxford United Kingdom; David Fawkner-Corbett, Paediatric surgical trainee

NIHR Community Healthcare MedTech and IVD Co-operative, Nuffield Department of Primary Care Health Sciences, University of Oxford, Oxford OX2 6GG, United Kingdom; Gail N Hayward, Associate professor

Department of General Practice and Elderly Care Medicine, University of Groningen, University Medical Center Groningen, PO Box 196, 9700 AD, Groningen, Netherlands, Mohammed Alkhmees, medical student

NIHR Community Healthcare MedTech and IVD Co-operative, Nuffield Department of Primary Care Health Sciences, University of Oxford, Oxford OX2 6GG, United Kingdom; NIHR Oxford Biomedical Research Centre, Oxford University Hospitals NHS Foundation Trust, Oxford, OX3 9DU, Oxford, United Kingdom, Jose M Ordóñez-Mena, medical statistician

EPI-Centre, Academic Centre for Primary Care, Department of Public Health and Primary Care, KU Leuven, Bus 7001, 3000 Leuven, Belgium; Ann Van den Bruel, Associate professor

NIHR Community Healthcare MedTech and IVD Co-operative, Nuffield Department of Primary Care Health Sciences, University of Oxford, Oxford OX2 6GG, United Kingdom; Department of General Practice and Elderly Care Medicine, University of Groningen, University Medical Center Groningen, PO Box 196, 9700 AD, Groningen, Netherlands, Gea A Holtman, assistant professor

Correspondence to: David Fawkner-Corbett (David.fawkner-corbett@imm.ox.ac.uk)

NIHR Community Healthcare MedTech and IVD Co-operative, Nuffield Department of Primary Care Health Sciences, University of Oxford, Oxford OX2 6GG, United Kingdom

**Word count:** 4539

**Figures:** Three main figures, eight supplementary

**Tables:** Two main tables, five supplementary



For peer review only - http://bmjopen.bmj.com/site/about/guidelines.xhtml

## Abstract

**Objective:** Possible appendicitis in children is a common emergency presentation. The exact value of blood tests is debated. This study sought to determine the diagnostic accuracy of four blood tests (White cell count [WCC], neutrophil [count or percentage], C-reactive protein [CRP] and/or procalcitonin) for suspected childhood appendicitis.

**Design:** Systematic review and diagnostic meta-analysis.

**Data sources:** MEDLINE, EMBASE, Central, Web of Science were searched from inception to March 2022 with reference searching and authors were contacted for missing/unclear data.

**Eligibility criteria for study selection:** Studies reported on the diagnostic accuracy of the four blood tests compared to the reference standard histology or follow-up. Risk of bias was assessed (QUADAS-2), pooled sensitivity and specificity were generated for each test and for commonly presented cut-offs. To provide insight into clinical impact, we present several strategies using a hypothetical cohort of children suspected of appendicitis.

**Results:** 67 studies were included (34,839 children, 13,342 with appendicitis), all were in the hospital setting (73% in emergency department). The most sensitive tests were WCC (≥10,000 cells/µl, 53 studies sensitivity 0·85[95% CI 0·80-0·89]) and absolute neutrophil count [ANC] (≥7,500 cells/µl, five studies sensitivity 0·90[95% CI 0·85-0·94]). Applying results to a hypothetical cohort of 1000 children presenting with symptoms of appendicitis, of whom 400 have appendicitis, 60 and 40 children would be wrongly discharged based solely on WCC and ANC respectively.
The most specific tests were CRP alone (≥50mg/l, 38 studies, specificity 0·87[95% CI 0·80-0·91]) or combined with WCC (≥10,000 cells/µl and CRP≥50mg/l, individual patient data of six studies, 0·93[95% CI 0·91-0·95]).

**Conclusions:** The best performing blood tests for ruling-out paediatric appendicitis are WCC or ANC. With technology developing at pace these tests could be used at the point of care in combination with clinical prediction rules at the emergency department. We provide insight into the cut offs which perform best for clinical application.

**Systematic review registration:** PROSPERO-CRD42017080036


For peer review only - http://bmjopen.bmj.com/site/about/guidelines.xhtml

## Strengths and limitations

- This systematic review and meta-analysis looked at the accuracy of the four most commonly used blood tests for diagnosing appendicitis against a reference standard of histology/surgical diagnosis or follow-up.
- An extensive literature search for all studies reporting children presenting with symptoms of appendicitis reporting metrics for white cell count, neutrophil (count or percentage), C-Reactive protein and/or procalcitonin were included.
- Authors were contacted when data was unclear, generating a pool of individual patient data to allow use of new methods and information about the cut offs that perform best for clinical application for all tests.
- The literature is limited for application of results in primary care or as point of care tests, as no studies reported diagnostic accuracy in these settings.
- Results were applied to a hypothetical cohort to aid clinical interpretation.

For peer review only


For peer review only - http://bmjopen.bmj.com/site/about/guidelines.xhtml

## Introduction

Abdominal pain is a common reason for a child to attend the emergency department (ED)[1]. Differentiation between self-limiting and surgical conditions, such as acute appendicitis, can be especially difficult in children where less than 50% of patients present with classically described symptoms[2–4]. Acute appendicitis is the most common surgical condition with over 12,000 children undergoing emergency surgery for the condition each year in England[5]. It is vital to prevent an unnecessary operation in children, which is reported in 5% to 20% of cases[6–9], whilst also undertaking prompt treatment and avoiding inappropriate discharge to mitigate the risks of more severe advanced disease including perforation[10,11]. Radiological investigations including computed tomography (CT) imaging, magnetic resonance imaging (MRI) or ultrasound are useful adjunct to decide who needs surgery[7,12]. However, these modalities will often require admission and routine use in all suspected cases would increase the burden on the health-care system and children[13].

Blood tests are frequently used to investigate children with suspected appendicitis to help inform diagnostic decisions; with White cell count (WCC), neutrophils (as count or percentage) and C-reactive protein (CRP) being most widely reported[14,15]. Procalcitonin is also increasingly used to distinguish paediatric sepsis[16]. Much of the evidence for their accuracy in this setting is applied from adult studies[17,18]. All these blood tests can be available within hours and are also increasingly available rapidly as point-of-care (POC) tests thus extending their future application to expediate the clinical decision making process and broaden their use into community care[19].

Clinical scoring tools, which can incorporate clinical signs and blood results, can be used to triage patients at hospital to determine the need for imaging or surgery[20]. Although the clinical prediction scores are recommended their reported accuracy and uptake varies widely, and use is more limited in children[21]. Additionally a core scoring criteria of each is blood tests with the type of test and cut-off varying, reflecting the fact the evidence of optimum diagnostic cut off is scarce[13,22].

Taken together, blood tests are widely performed but exact accuracy for informing the diagnosis of paediatric appendicitis is debated[23]. More insight in the diagnostic accuracy of several cut offs of blood tests would help to choose which POC tests should be prioritised


For peer review only - http://bmjopen.bmj.com/site/about/guidelines.xhtml

for implementation in the emergency department, which should best inform admission or discharge decisions, and which cut-offs are optimum to integrate with future optimisation of clinical scoring tools. To date a large, systematic appraisal of the diagnostic accuracy of these tests in paediatric appendicitis is lacking and so this study aims to undertake a systematic review and meta-analysis of all studies reporting the diagnostic accuracy of common blood tests in the diagnosis of paediatric appendicitis.

For peer review only


For peer review only - http://bmjopen.bmj.com/site/about/guidelines.xhtml

## Methods

This systematic review and meta-analysis was undertaken in line with the Preferred Reporting Items for Systematic Reviews and Meta-Analyses (PRISMA) guidelines[24] and was prospectively registered (PROSPERO: CRD42017080036).

### Public and patient involvement:

The concept of this review involved discussion with patients and families before it was undertaken and discussed experience in paediatric blood tests, identifying it as important to know the exact value (Precision antimicrobial prescribing patient and public involvement group, University of Oxford). The results of this manuscript were also presented in this forum which helped formulate the write up and key discussion points within the final manuscript.

### Search Strategy:

Searching of databases (Cochrane Database of Systematic Reviews, Cochrane Central Register of controlled trials, Database of abstracts of reviews of effects(DARE), Embase, Ovid(Medline) Epub Ahead of print in process and other Non-Indexed Citations, Ovid Medline(R) Daily, Ovid Medline(R) and Science Citation Index and Conference Proceedings Citation Index (SCIENCE)) included key word combinations to identify children with symptoms suggestive of appendicitis having one of the four blood tests (WCC, neutrophil, CRP, procalcitonin) for diagnosis. Searching was performed on 15th March 2022 to include all relevant studies from database inception with no language or date limitations. Full search strategy is provided in **Supplementary Materials**. Hand searching of included studies references was undertaken. Assessment of foreign language studies was completed by a native language speaker.

### Study Selection and Data Extraction:

Included studies reported children (<18 years) presenting with symptoms suggestive of appendicitis that would have testing of WCC, neutrophil, CRP and/or procalcitonin, by laboratory or POC, to distinguish appendicitis from non-surgical cause.


For peer review only - http://bmjopen.bmj.com/site/about/guidelines.xhtml

For studies that were included the optimal reference standard for confirming diagnosis of appendicitis was decided as histology after surgery (ideal standard) or; surgery alone, imaging confirming appendicitis in non-operatively managed patients or surgical diagnosis in studies without histological reporting. In patients without surgery (non-appendicitis) the reference standard was follow-up after initial assessment to rule out appendicitis with an ideal standard of ≥14 days with active follow up. Studies that differed or were unclear about confirming these standards would be highlighted when assessing risk of bias.

Studies were included if they matched these pre-defined criteria and reported sufficient data to construct or derive a two-by-two contingency table for any/multiple blood tests. Studies were excluded if they only reported surgical cases, had <20 participants, reported atypical appendiceal pathology (e.g. carcinoid, appendiceal mass) or if the proportion of participants outside the age range was >15%. Where multiple studies reported data on the same population, only outcomes of the earliest study were used, unless the authors confirmed individual patient results.

Screening of titles and abstracts was performed independently by two reviewers (DFC and GAH). Full text articles of all short-listed studies were then assessed for eligibility.

Four reviewers (DFC, GAH, JMOM, MA) independently extracted data from each included study on a pre-designed data extraction sheet including information on demographics, prevalence, reference standards, measurement of blood tests including cut-offs, and 2x2 tables. Disagreements were resolved by consensus or from input of an independent reviewer (GNH).

In studies which matched inclusion criteria exclusion with reason would occur if: (a) data was not able to be extracted in 2x2 format (derivation from reported sensitivity / specificity or other metrics would be used if possible); (b) a paediatric sub-group was not adequately detailed; (c) exclusion criteria were unclear; (d) the paper was only available in an abstract form. If reason (a) was given for full-paper exclusion an attempt would be made to contact an author on up to three occasions before exclusion. If contacted authors provided individual patient data, this was included.

**Assessment of study methodological quality:**

All included studies were assessed by four reviewers (DFC, GAH, JMOM, MA) independently for risk of bias and applicability using the QUADAS-2 tool[25]. Discrepancies were resolved by


For peer review only - http://bmjopen.bmj.com/site/about/guidelines.xhtml

discussion or review from a third independent reviewer (GNH) to achieve consensus. Full details of QUADAS-2 scoring criteria are detailed in **Supplementary Materials: QUADAS-2**. Overview graph of QUADAS-2 outcomes was created using GraphPad Prism (V 8.0, GraphPad Software Inc).

**Data synthesis and statistical analysis:**

We extracted binary data for all appropriate measurements of diagnostic accuracy for the pre-defined blood tests. Cut-offs were defined by authors in each individual publication. In cases where individual data was provided by authors a 2x2 table was calculated using the most commonly reported cut-offs. Data for age-dependent cut-offs would be included in study overviews and forest plots but not in cut-off specific meta-analysis. Measurement of neutrophils by means of absolute count (ANC) or percentage of white cells was analysed separately.

Test accuracy overviews for each marker were reported as forest plots including 2x2 tables, sensitivity and specificity with 95% confidence intervals using RevMan (Cochrane collaboration, Copenhagen, Denmark). Meta-analysis was performed using a bivariate random effects model and pooled estimates of sensitivity, specificity and likelihood ratios were generated when >4 studies per marker was included using the *metandi* module in STATA version 16[26–28]. When a study provided multiple cut-offs for each test, we calculated the summary receiver operating characteristics (SROC) curve with 95% CI using R *diagmeta* package developed by Steinhauser et al[29] assuming a fixed intercept (diagnostic accuracy) and slope (threshold effect) across studies. In addition, we calculated the pooled sensitivity and specificity for commonly presented cut-offs and we compared the results with the bivariate random effects model of cut-off groups of these tests.

We assessed the heterogeneity by visual inspection of the forest plot and SROC plot. Potential differences in studies with: high risk of bias in patient selection; case-control design; from primary care; with low prevalence (<10%); with high prevalence (≥60%), were evaluated by adding these covariates to the bivariate model and performing sensitivity and sub-group analysis to measure if these factors could explain heterogeneity. To assess for publication bias we used the Deeks' test[30].

**Potential clinical impact:**


For peer review only - http://bmjopen.bmj.com/site/about/guidelines.xhtml

To provide more insight into the clinical consequences of using the results of each test, for each measurement with pooled sensitivity and specificity hypothetical 2×2 tables were constructed in 1000 children presenting with symptoms suggestive of acute appendicitis. The appendicitis prevalence was modelled on the median prevalence in the cohort studies included in the final meta-analysis, rounded to the nearest 10% to help facilitate interpretation. Standardisation of prevalence allowed comparison of the results of each test.

To illustrate the clinical application of tests we interpreted these as follows: children with appendicitis missed are those with appendicitis and a negative index test result (false negatives); the numbers of unnecessary surgeries are the children without appendicitis with a positive index test result (false positives).

For peer review only


For peer review only - http://bmjopen.bmj.com/site/about/guidelines.xhtml

## Results

### Search Strategy and study selection

The search strategy, reference screening and paediatric data provided by contacted authors identified 3,211 studies. 67 studies matched inclusion criteria and were included in final analysis, of which 11 studies had patient data provided after contacting authors to clarify criteria or provide paediatric-only data[31–41]. Remaining papers were excluded with reason (**Figure 1, Supplementary Table 1**).

### Study Characteristics

All included studies were in the hospital or emergency department setting. The most common study designs were cohort study, with either a prospective (n=40, 60%) or retrospective (n=11, 16%) cohort design, or case control (n=6, 9%) (**Table 1, Supplementary Table 2**). One study had a low prevalence (<10%) of appendicitis[42]. Seven cohort studies had a high prevalence (>60%) due to a more select setting such as GP referrals[43,44], hospitalised [39,43,45,46] or ITU based patients[41]. In one study the reason for high prevalence was unclear[47]. The median prevalence for all studies was 44% (IQR 34-58%). No study used POC blood test devices.

The most frequent reference standard in appendicitis patients was histopathology with the remainder using operative reports (**Table 1, Supplementary Table 3**). Of 67 studies, 61 (91%) described inclusion of a population of children with clinical symptoms of appendicitis and eight of these studies specified a blood test and/or ultrasound for inclusion; the remaining 6 studies (9%) were of case-control designs and most commonly identified cases through surgery and controls as those with symptoms of appendicitis which had the condition ruled out (**Supplementary Table 2**).

For peer review only


For peer review only - http://bmjopen.bmj.com/site/about/guidelines.xhtml

| | | Studies (n=67) |
|---|---|---|
| **Study design** | Prospective cohort study | 40 (60%) |
| | Retrospective cohort study | 11 (16%) |
| | Case control study | 6 (9%) |
| | Prospective observational study | 3 (4%) |
| | Retrospective case series | 2 (3%) |
| | Other* | 5 (7%) |
| **Setting** | **Institution**<br>Hospital | 67 (100%) |
| | **Department†**<br>Emergency department<br>Surgical department | 49 (73%)<br>17 (25%) |
| | **Centre**<br>Single centre<br>Multiple centre | 54 (81%)<br>13 (19%) |
| **Study participants** | Total (n) | 34,839 ‡ |
| | Median patients included (range) | 275 (44-3791) |
| | ≤50 participants | 4 (6%) |
| | 51-250 participants | 28 (42%) |
| | 251-500 participants | 14 (21%) |
| | >500 participants | 21 (31%) |
| **Year Published** | Before 1989 | 2 (3%) |
| | 1990-1999 | 5 (7%) |
| | 2000-2009 | 14 (21%) |
| | 2010-2022 | 46 (69%) |
| **AA Prevalence** | <20% | 6 (9%) |
| | 21-35% | 14 (21%) |
| | 36-50% | 24 (36%) |
| | 51-65% | 18 (27%) |
| | >65% | 5 (7%) |
| **Gender demographics(% Male)** | <35% | 1 (1%) |
| | 36-45% | 8 (12%) |
| | 46-55% | 29 (43%) |
| | 56-65% | 20 (30%) |
| | >65% | 2 (3%) |
| | Unclear | 7 (10%) |
| **Reference standard (AA)** | Histology/ Pathology | 63 (94%) |
| | Operative findings | 4 (6%) |
| **Reference Standard (non-AA)** | Active follow up | 19 (28%) |
| | Discharge diagnosis | 16 (24%) |
| | Unspecified follow up | 8 (12%) |
| | Observation | 8 (12%) |
| | Unclear | 8 (12%) |
| | Failure to reattend | 6 (9%) |
| | Case note review | 2 (3%) |
| **Index test(s) measured** | White cell count (WCC) | 59 (88%) |
| | C-reactive protein (CRP) | 38 (57%) |
| | Absolute neutrophil count | 25 (37%) |
| | Neutrophil percentage | 16 (24%) |
| | WCC and CRP | 13 (19%) |
| | WCC, CRP and neutrophil | 6 (9%) |
| | Procalcitonin | 6 (9%) |
| | Neutrophil : lymphocyte ratio | 3 (4%) |
| | WCC and Neutrophil count | 1 (1%) |

For peer review only


For peer review only - http://bmjopen.bmj.com/site/about/guidelines.xhtml

**Table 1**: **Pooled overview of design and standards of included studies**
(*In 5 studies authors reported other study designs than those listed. † In one study (1%) department setting was unclear. ‡ value includes all participants in included studies, in 5 studies a paediatric sub-group was provided meaning total paediatric patients in analysis n=31,143. Full details of all study characteristics including design, setting, population characteristics and demographics, exclusion and inclusion criteria is fully detailed in **Supplementary Table 2.** Full details of study reference standard, index tests and cut offs in **Supplementary Table 3**)

For peer review only


For peer review only - http://bmjopen.bmj.com/site/about/guidelines.xhtml

## Risk of Bias

Applying the QUADAS-2 framework[25] patient selection in 60 studies (90%) were low risk of concern for applicability of results as they clearly included patients with suspected acute appendicitis. Those not scoring low included higher ages (n=4)[36,48-50] or only those undergoing ultrasound scans (8·3%, n=5)[51-55]. However fewer studies scored low for risk of bias (44%, n=30) when considering patient selection; most commonly because studies did not clearly state whether they recruited a random or fully consecutive population (**Figure 2, Supplementary table 4)**.

Risk of bias and applicability of the index test and reference standard was often low risk or unclear - owing to a lack of detail regarding blinding (i.e. clinician or pathologist) or follow-up method. No study scored low for "flow and timing" as a component as this required both groups to receive the same reference standard. It is understandably not ethical to perform surgery in all children, and therefore in this dimension the seven studies that score "unclear" due to lack of detailed outcomes for all participants are likely of greater concern (**Figure 2**).

## Blood Tests

The 67 included studies consisted of 34,839 children (13,342 with appendicitis and 21,497 without) which evaluated the following tests: WCC, CRP, ANC, neutrophils as percentage of leukocytes, procalcitonin, combined WCC and CRP, and combined WCC/CRP/neutrophil percentage. Neutrophil to lymphocyte ratio (NLR) was reported in three studies which was not sufficient to allow a meta-analysis. Reported accuracies ranged from sensitivity 0.18-0.91, and specificity 0.69-0.95 but cut-offs used were dissimilar[56-58]. Similarly, the combination of WCC and ANC (>13,300/µl and >10,800/µl respectively) was reported in one study (sensitivity = 0.76, specificity =0.82)[59]. We found no evidence of publication bias for each test where this was appropriate to assess (**Supplementary Figure 1**).

An overall pooled estimate for each test, at different cut-offs, is shown in **Table 2**. For WCC and CRP the pooled estimates obtained for each cut-off with the Steinhauser method were similar for the cut-off groups using the bivariate model (**Supplementary table 5**). In subgroup analyses, the test characteristics did not differ between studies with a high


For peer review only - http://bmjopen.bmj.com/site/about/guidelines.xhtml

appendicitis prevalence (≥60%) and lower prevalence. In some tests when using bivariate analysis the study design (for WCC 10-15,000 cells/μl and neutrophils) or patient selection (for WCC and CRP) had an effect on the sensitivity or specificity which we quantified (**Supplementary table 5**).

For peer review only


For peer review only - http://bmjopen.bmj.com/site/about/guidelines.xhtml

| Marker | Studies | Threshold | Pooled sensitivity* (95% CI) | Pooled specificity* (95% CI) | Hypothetical cohort of 1000 children with appendicitis prevalence of 40%[†] | | Hypothetical cohort of 1000 children with appendicitis prevalence of 5% | |
|---|---|---|---|---|---|---|---|---|
| | | | | | Missed appendicitis (FN) | Unnecessary treatment (FP) | Missed appendicitis (FN) | Unnecessary treatment (FP) |
| WCC[‡] (Cells/µl) | 53 | 10,000 | 0·85 (0·80 – 0·89) | 0·58 (0·51 – 0·64) | 60 | 252 | 8 | 399 |
| | | 12,000 | 0·73 (0·66 – 0·79) | 0·71 (0·65 – 0·76) | 108 | 174 | 14 | 276 |
| | | 15,000 | 0·53 (0·45 – 0·62) | 0·83 (0·78 – 0·87) | 188 | 102 | 24 | 162 |
| CRP[§] (mg/L) | 38 | 5 | 0·77 (0·69 – 0·84) | 0·54 (0·45 – 0·63) | 92 | 276 | 12 | 437 |
| | | 10 | 0·68 (0·59 – 0·76) | 0·66 (0·58 – 0·73) | 128 | 204 | 16 | 323 |
| | | 30 | 0·50 (0·39 – 0·60) | 0·81 (0·75 – 0·87) | 200 | 114 | 25 | 181 |
| | | 50 | 0·41 (0·30 – 0·53) | 0·87(0·80 – 0·91) | 276 | 78 | 35 | 124 |
| Neutrophils[¶] (% of lymphocytes) | 12 | 75% | 0·76 (0·73-0·80) | 0·63 (0·57-0·69) | 96 | 222 | 12 | 352 |
| ANC(Cells/µl) | 5 | <7,500 | 0·90 (0·85 – 0·94) | 0·54 (0·49 – 0·58) | 40 | 276 | 5 | 437 |
| | 13 | 7,500 | 0·86 (0·82 – 0·89) | 0·60 (0·54 – 0·66) | 56 | 240 | 7 | 380 |
| | 6 | >7,500 | 0·70 (0·65 – 0·75) | 0·78 (0·71 – 0·85) | 120 | 132 | 15 | 209 |
| Procalcitonin (ng/ml) | 6 | 0·1 – 0·5 | 0·30 (0·26 – 0·35) | 0·83 (0·73 – 0·89) | 280 | 132 | 35 | 209 |
| WCC and CRP[‡] | 6 IPD studies | 10,000 and 10 | 0·59 (0·49-0·68) | 0·79 (0·74-0·84) | 164 | 126 | 21 | 200 |
| WCC and CRP | 6 IPD studies | 10,000 and 50 | 0·27 (0·19-0·37) | 0·93(0·91-0·95) | 292 | 46 | 37 | 67 |
| WCC or CRP | 6 IPD studies | 10,000 and 10 | 0.97 (0.93 – 0.99) | 0.25 (0.10 – 0.51) | 12 | 450 | 2 | 713 |
| WCC or CRP | 6 IPD studies | 10,000 and 50 | 0.91 (0.86 – 0.95) | 0.50 (0.40 – 0.60) | 36 | 300 | 5 | 475 |

**Table 2.** Pooled estimates of diagnostic accuracy of makers for diagnosis of appendicitis in children (*All pooled sensitivity and specificity were obtained from the bivariate model, except for WCC and CRP for which we calculated sensitivity and specificity for selected cut offs using the Steinhauser method, comparison with bivariate mode including Likelihood ratios for all tests is demonstrated in **Supplementary Table 5**. [†]Median prevalence of appendicitis in the included 62 cohort studies is 40% [‡]WCC was evaluated in 59 studies whereof 53 were included in the meta-analysis whereof 12 provided multiple cut offs. Six studies could not be used in the pooled analysis, because 5 studies reported WCC provided age-dependent cut offs and 1 study reported pain duration dependent cut offs. [§]CRP was evaluated in 39 studies whereof 11 provided multiple cut offs. [¶]Neutrophils evaluated in 16 studies of which 12 provided cut off at 75%. [‡]WCC and CRP calculated from individual patient data from 6 studies (n=1541 participants) and analysed using the bivariate model)

For peer review only



For peer review only - http://bmjopen.bmj.com/site/about/guidelines.xhtml

*WCC*

WCC was the most frequently reported blood test (59 studies, including 22,779 patients; 9,733 with appendicitis). At a cut-off of 10,000 cell/µl WCC exhibited one of the highest pooled sensitivities of any individual test (0.85, 95% CI 0.80-89, 53 studies) but specificity was low (0.58, 95% CI 0.51-0.64] **Table 2, Figure 3A, Supplementary Figure 2**).

*CRP*

CRP was the second most frequently reported test (38 studies, 9934 patients [4201 appendicitis]. The sensitivity and specificity for lower cut-off values varied considerably between studies on visual inspection (**Supplementary Figure 3**). Specificity of a CRP value of ≥50 mg/L was more homogenous. The highest pooled sensitivity of CRP was at a cut-off of ≤5mg/L (0.77 (95% CI 0.69 – 0.84)), although specificity was low (0.54, 95% CI 0.45 – 0.63**, Table 2**). Use of a cut-off ≥50mg/L had the best specificity of any single test at 0.87 (95% CI 0.80-0.91, **Table 2** and **Figure 3B**) and all studies reported a specificity of >0.8 for this (**Supplementary Figure 3**).

*Neutrophil measurement*

Neutrophil measurement was reported as an absolute count (ANC) in 25 studies, including 12,982 patients (5311 appendicitis). ANC was more frequently reported and demonstrated the highest sensitivity of any test at a low cut-off (≥7,500 cells/µl, 0.90, 95% CI 0.85-0.94) **Table 2, Supplementary Figure 4**). However, none of the studies evaluating ANC reported multiple cut-offs and therefore the SROC curve could not be calculated using the Steinhauser method. Relatively more studies (n=13) reported a cut-off of 7,500 cells/µl which reported a similar sensitivity (0.86 (95% CI 0.82-0.89), **Table 2**) to WCC.

Fewer studies (n=16, 5884 patients; 2275 appendicitis) reported neutrophil count as a percentage (**Supplementary Figure 5, Supplementary Table 5**), the most common cut-off reported was 75% and pooled (n=9) analysis at this level showed the sensitivity to be lower than ANC and WCC (**Table 2**).

*Procalcitonin*


For peer review only - http://bmjopen.bmj.com/site/about/guidelines.xhtml

Procalcitonin accuracy was quantified in 6 studies (860 patients; 469 appendicitis) which led to pooled analysis being performed on varied cut-offs. Sensitivity was homogenous, while specificity varied between the studies (**Supplementary Figure 6**). Pooled procalcitonin had a relatively high pooled specificity (0.83, 95% CI 0.73-0.89) but a low sensitivity (0.30, 95% CI 0.26-0.35) (**Table 2**).

*Combinations of tests*

When considering combinations of tests all but one of the included studies reported as a combination of both tests being positive to define a positive test. The cut-offs did vary between studies and the studies were therefore too heterogeneous to perform a meta-analysis. A combination of CRP and WCC (13 studies, 4,887 patients, 1,629 appendicitis) demonstrated a sensitivity range of 0.34 to 0.97 and specificity range of 0.34 to 1.00 (**Supplementary Figure 7**). The study with a combination of one test being positive showed a sensitivity and specificity of 0.997 and 0.24, respectively.

A combination of CRP, WCC and neutrophils (six studies, 1,784 patients, 625 appendicitis) showed a range of sensitivities (0.43-1.00) and specificity (0.25-0.89) which likely reflected the differing reported cut offs (**Supplementary Figure 8**).

To account for pooling of differing cut-offs in combinations of tests we performed a sub-group analysis of patients from six studies which provided individual patient data (IPD) on request for clarification[31–35,41]. This included the raw results for 1541 patients and showed that a combination of WCC >10,000 cell/µl and CRP>10mg/L has reasonable pooled specificity (0.79, 95% CI 0.74-0.84) which was increased when the threshold for CRP was raised to >50mg/L with WCC (0.93, 95% CI 0.91-0.95), with a resulting low sensitivity (0.27, 95% CI 0.19-0.37, **Table 2**). Using a combination of tests as either WCC or CRP being positive at a low cut off generated the best specificity of any measured investigation (WCC >10,000 cell/µl or CRP>10mg/L; 0.97, 95% CI 0.93-0.99, **Table 2**).

*Clinical impact*

Modelling the effect of each test upon a hypothetical cohort of 1,000 children with a 40% prevalence of appendicitis, which was the median prevalence of all included studies rounded to the nearest decile (median 44%, IQR 33-58%), showed that the best single tests performing best for ruling out appendicitis were ANC <7,500 cells/µl and WCC


For peer review only - http://bmjopen.bmj.com/site/about/guidelines.xhtml

≥10,000cells/µl, as these only miss 40 and 60 of the 400 children with appendicitis respectively, but would capture a large number of non-appendicitis cases for further review of treatment – 276 and 252 out of 600 respectively (**Table 2, Supplementary Table 5**). Therefore, in clinical application these tests would be the best for supporting a decision to discharge, or to not refer a patient for surgical review.

The best single test for ruling in appendicitis was a CRP value ≥50mg/L and so could be the most useful in deciding to rule in the diagnosis of appendicitis. Although in the hypothetical cohort 78 would still undergo unnecessary review or treatment (False positive) of the 202 that would meet this threshold. Also, 276 children with appendicitis would be missed (**Table 2**).

Using IPD identified a combination of WCC >10,000 cells/µl and CRP >50mg/L was more specific, application of this to inform surgical decision would result in the lowest number of unnecessary operations (n=46) but only 150 patients would meet this threshold in the hypothetical cohort, 104 of which would have appendicitis (**Table 2**).

Although no included studies were in the community setting, the analysis was repeated with a lower prevalence (5%), which is reported as the prevalence in primary care[60]. This was used to reflect metrics for use of blood tests in a non-emergency department setting, or if it was used for a triage test to decide other diagnostic modalities such as ultrasound (**Table 2**).

For peer review only



For peer review only - http://bmjopen.bmj.com/site/about/guidelines.xhtml

## Discussion

In a pooled analysis of 31,143 children attending hospital with symptoms of appendicitis the most sensitive blood tests for discriminating appendicitis from non-surgical conditions were WCC or ANC, and the most specific were CRP with or without WCC. However, if these were used as a sole decision-making tool for discharge in a hypothetical cohort of 1000 children with 40% appendicitis prevalence, even the most sensitive test would result in 40 cases of missed appendicitis (ANC).

Conversely, although a blood test alone would unlikely be used as the sole tool for surgical decision making, if a blood test was used to rule in the condition the most specific test would result in 78 unnecessary treatments (CRP). Therefore, all common inflammatory blood tests have limitations in the diagnosis of paediatric appendicitis which we accurately quantify. We highlight which tests should be valued most highly in more complex diagnostic pathways.

*Strengths*

To our knowledge, this is the largest systematic review of the diagnostic accuracy of commonly utilised blood tests to support diagnosis in children with suspected appendicitis. Through use of a broad search strategy we combined information from 67 studies and report pooled measurements for five common blood tests at multiple cut-offs and in combination. Our analysis used multiple models for quantification and also undertook analysis for potential sub-group and publication biases. Previous diagnostic accuracy meta-analysis methods have used diagnostic data for a single cut-off, and often the selected cut-off is the one maximizing both diagnostic accuracy parameters (sensitivity and specificity). In our methods we used commonly reported cut offs to identify those with the highest clinical impact. Thus, by using our approach, we ensure the pooled diagnostic accuracy parameters are not overestimated[29].

The study gained important additional data through contacting authors and used individual patient data where possible, adding to the applicability of results to clinical practice. It also aided us in reporting combination of tests, with cut-offs being variable in literature but allowing inclusion of over 1500 children to demonstrate the power of combined WCC and


For peer review only - http://bmjopen.bmj.com/site/about/guidelines.xhtml

CRP to maximise sensitivity if either are positive, or specificity if both are negative. The included studies had representation from a variety of continents and 69% were undertaken in the last twelve years adding to the applicability of results. In addition, 94% of the studies confirmed the diagnosis of acute appendicitis with histopathology results adding to the reliability of findings.

*Potential limitations*

An important limitation is that no studies were identified from the primary care settings, or reported results of POC devices. Our findings could be readily applicable to the emergency setting for use in triage, similar to other conditions[61]. Although our sensitivity analysis suggested that prevalence did not affect the diagnostic accuracy, these results should be interpreted with care as there was only one study with a very low prevalence (<10%)[42]. We repeated the cohort analysis in a lower prevalence setting (5%) to illustrate likely performance if blood tests were used in the community or as a triage test for imaging investigation, although metrics did reflect information from higher prevalence studies and differences in case-mix or severity in appendicitis could result in a different diagnostic accuracy.

Another possible limitation is that acute appendicitis can represent a spectrum of severity, some reports suggesting histological subtypes of appendicitis can result in differing laboratory results[62]. This differentiation can only be made post-operatively, but our approach was to evaluate test application to all cases of suspected appendicitis so that it best represented the diagnostic decision making when clinicians first encounter cases. A small number of studies (n=5 for WCC, n=1 for ANC, and n=1 for neutrophils) also divided tests thresholds by age. These were too few to pool and were heterogeneous, so these results were reported but not used in meta-analysis.

Additionally, pooled metrics were applied to a hypothetical cohort which aids in the application but would not wholly represent the clinical setting, where full decision making would be more complex and could utilise other diagnostic modalities such as imaging. Also, although these results give one of the largest pooled analysis of blood tests the strategy is not able to identify the added value of these to specific signs and symptoms, or as a triage to additional diagnostics.



For peer review only - http://bmjopen.bmj.com/site/about/guidelines.xhtml

*Comparison with literature*

Smaller systematic reviews including adults and children have reported different metrics for diagnostic accuracy, such as WCC sensitivity being 0.62-0.87[18,63,64], which is likely a result of pooling multiple cut-offs from studies. To mitigate this effect we have pooled by similar cut-off generating SROC curves for each or from IPD where possible. This should increase the reliability and applicability of results, and has highlighted that at a specific cut-off of WCC sensitivity was higher than previously reported (0.84 at 10,000cells/μl).

Other studies report combined clinical scoring tools for diagnosing appendicitis, with blood tests as one component. A recent review reported the best performing of these (the Shera score) at a low cut-off to miss 18 out of 539 cases of paediatric appendicitis if used[20]. The specificity of this scoring tool was lower than any of our measured blood tests however (44.3% 95% CI 41.4-47.2%). These tools have the advantage of incorporating clinical signs and symptoms, and our results complement these by quantifying the exact benefit of tests at different cut-offs.

Blood tests in paediatric appendicitis have also been reviewed as part of a systematic review of other diagnostic modalities, with WCC identified as sensitive at 10,000 cells/μl (0.88% 95% CI 87-90%) and CRP as specific (no pooled analysis)[65]. This did not include retrospective or non-ED studies, being limited to single studies at some cut offs. Our study seems to agree with this but included more studies and IPD where available which allowed reporting of different cut-offs and highlighted the benefit of test combinations.

*Clinical implications*

Applying the meta-analysis results to a hypothetical clinical cohort demonstrated that ANC, WCC and CRP are the tests with greatest clinical value. Although in practice clinicians may not rely on blood tests alone, it is still important to know ANC or WCC at a low cut off would be the best tests to inform a decision to discharge. Using this strategy for triage would result in a relatively low number of missed appendicitis cases (64 and 40 out of the 400 cases in the modelled cohorts respectively). These values could be used to develop triage pathways, or equate risk to parents to self-observe. These results could provide the basis for application of selected blood tests in a prospective trial in the triage setting, or an evaluation of POC tests outside the emergency department[66].


For peer review only - http://bmjopen.bmj.com/site/about/guidelines.xhtml

CRP at a high cut off or combined with WCC was the most specific test but a significant proportion of children would still reach this threshold, and a large number of true cases would be missed (276 and 188 respectively). A recent international cohort study of over 1800 children with right iliac fossa pain, identified a negative appendicectomy rate of 15.9%, increasing to 22.4% in girls aged 11-15 years[20]. Our results suggest using those blood tests to inform surgery would perform worse than this, highlighting blood tests alone should not be used to rule in appendicitis.

Application of these results, especially in the situation of combined blood tests in addition to signs and symptoms, would leave a proportion of children between the two clinical situations of a positive low WCC/ANC, and high CRP. In these children where diagnostic uncertainty remains, further assessment by specialist or use of an adjunct such as ultrasound could be considered. Using specific tests in combination with prediction rules to determine the need for investigations such as ultrasound could also lead to an increased diagnostic accuracy of imaging, being higher when the pre-test probability increases[67]. Our results would not support using procalcitonin or neutrophil percentage as a preferred test for clinical decision making[68].

*Conclusion*

In conclusion, we report the largest study of commonly available blood tests and identify WCC value below 10,000 cells/µl and ANC value below 7,500 cells/µl are the best test in ruling-out suspected childhood appendicitis. These tests could be used in addition to signs and symptoms to discharge or safety-net patients. A CRP value above 50mg/l in addition to signs and symptoms could be used to select patients for further imaging. However, more information about the exact added value of these blood tests incorporated with other diagnostic modalities is needed. Since we did not find any primary care studies or studies evaluating POC tests, these three tests could be prioritised for future evaluation.


For peer review only - http://bmjopen.bmj.com/site/about/guidelines.xhtml

**Figure Legends:**

**Figure 1:** PRISMA flow chart for identification of included studies (*some studies excluded for 2 reasons, see full overview of excluded studies in **Supplementary table 1**)[24]

**Figure 2:** Overview of QUADAS-2 assessment of all included studies (n=67) showing review authors' assessment of risk of bias and study applicability in each domain reported as a percentage of all included studies (proportion coloured by assessment as high [red], unclear [yellow] or low [green], full outline of all individual components of QUADAS-2 is detailed in **Supplementary table 4**).

**Figure 3:** Summary receiver operating characteristic plot of WCC **(A)** and CRP **(B)** for diagnosis of paediatric acute appendicitis (Colour of dot representing study identified in legend, hashed grey box = 95% confidence interval specificity, solid grey box = 95% confidence interval sensitivity. Examples of commonly reported cut-offs shown in **(A)** as blue [10,000/µl], green [12,000/ µl] and red [15,000/ µl] crosses on curve; in **(B)** as yellow [5ml/L], pink [10mg/L], blue [20mg/L], green [30mg/L] and red [50mg/L] crosses on curve)

For peer review only



For peer review only - http://bmjopen.bmj.com/site/about/guidelines.xhtml

**Contributorship statement:** DFC, GNH, AvdB and GAH conceived and designed the study. DFC and GAH assessed initial search results and selected studies for inclusion with discrepancies resolved with GNH and AvdB. DFC, GNH, JMOM and MA performed data extraction. DFC and GAH performed analysis. All authors were involved in formation and revision of final manuscript. The corresponding author attests that all listed authors meet authorship criteria and that no others meeting the criteria have been omitted.

**Competing interests:** No authors declare any competing interests.

**Funding:** This research was funded by the National Institute for Health and Care Research (NIHR) Community Healthcare MedTech and In Vitro Diagnostics Co-operative at Oxford Health NHS Foundation Trust. The views expressed are those of the author(s) and not necessarily those of the NHS, the NIHR or the Department of Health and Social Care. DFC received funding from the National Institute for Health Research as an Academic Clinical Fellow and the Wellcome Trust Doctoral Training Fellowship at the University of Oxford. GAH received funding by Ter Meulen Grant of the Royal Netherlands Academy of Arts and Sciences, and NWO talent programme Veni.

**Acknowledgements**: We thank Nia Roberts and the Bodleian healthcare libraries for assistance in conducting the search strategy. We acknowledge help in the review of foreign language studies provided by Patrick Garfjeld Roberts, University of Oxford. We appreciate those authors that provided patient data upon request to allow inclusion of their original studies

**Ethics approval statement:** Study design and methodology was prospectively registered (PROSPERO-CRD42017080036). Study design used analysis of published data which did not involve participants and so did not require institutional or research committee ethical approvals.

**Data sharing statement:** Data sharing is not applicable as no new datasets were generated in the generation of this study.

For peer review only


For peer review only - http://bmjopen.bmj.com/site/about/guidelines.xhtml

## References


1. Armon, K. *et al.* Determining the common medical presenting problems to an accident and emergency department. *Arch. Dis. Child.* **84**, 390–392 (2001).
2. Baxter, K. J., Nguyen, H. T. M. H., Wulkan, M. L. & Raval, M. V. Association of Health Care Utilization With Rates of Perforated Appendicitis in Children 18 Years or Younger. *JAMA Surg.* **153**, 544–550 (2018).
3. Rothrock, S. G., Skeoch, G., Rush, J. J. & Johnson, N. E. Clinical features of misdiagnosed appendicitis in children. *Ann. Emerg. Med.* **20**, 45–50 (1991).
4. Rentea, R. M. & St. Peter, S. D. Pediatric Appendicitis. *Surg. Clin. North Am.* **97**, 93–112 (2017).
5. Statistics, H. E. Hospital Episode Statistics (HES), Admitted Patient Care, England 2012-2013. *Hospital Episode Statistics (HES), Admitted Patient Care, England 2012-2013 [Report].* content.digital.nhs.uk/catalogue/PUB12566/hosp-epis-stat-admi-proc-2012-13-%0Dtab.xlsx (2012).
6. Wagner, J. M., McKinney, W. P. & Carpenter, J. L. Does This Patient Have Appendicitis? *JAMA* **276**, 1589–1594 (1996).
7. Tseng, J., Cohen, T., Melo, N. & Alban, R. F. Imaging utilization affects negative appendectomy rates in appendicitis: An ACS-NSQIP study. *Am. J. Surg.* **217**, 1094–1098 (2019).
8. Macco, S., Vrouenraets, B. C. & de Castro, S. M. M. Evaluation of scoring systems in predicting acute appendicitis in children. *Surgery* **160**, 1599–1604 (2016).
9. van Rossem, C. C. *et al.* Diagnosing acute appendicitis: surgery or imaging? *Color. Dis.* **18**, 1129–1132 (2016).
10. Lewis, S. R. R., Mahony, P. J. & Simpson, J. Appendicitis. *BMJ* **343**, (2011).
11. Meltzer, J. A. *et al.* Association of Delay in Appendectomy With Perforation in Children With Appendicitis. *Pediatr. Emerg. Care* **35**, 45–49 (2019).
12. Sola, R. *et al.* Standardized reporting of appendicitis-related findings improves reliability of ultrasound in diagnosing appendicitis in children. *J. Pediatr. Surg.* **53**, 984–987 (2018).
13. Di Saverio, S. *et al.* WSES Jerusalem guidelines for diagnosis and treatment of acute appendicitis. *World J. Emerg. Surg.* **11**, 1–25 (2016).
14. Andersson, R. E. B. Meta-analysis of the clinical and laboratory diagnosis of appendicitis. *Br. J. Surg.* **91**, 28–37 (2003).
15. Kwan, K. Y. & Nager, A. L. Diagnosing pediatric appendicitis: usefulness of laboratory markers. *Am. J. Emerg. Med.* **28**, 1009–1015 (2010).
16. Waterfield, T., Maney, J. A., Hanna, M., Fairley, D. & Shields, M. D. Point-of-care testing for procalcitonin in identifying bacterial infections in young infants: A diagnostic accuracy study. *BMC Pediatr.* **18**, 1–6 (2018).
17. Snyder, B. K. & Hayden, S. R. Accuracy of Leukocyte Count in the Diagnosis of Acute Appendicitis. *Ann. Emerg. Med.* **33**, 565–574 (1999).
18. Yu, C. W. *et al.* Systematic review and meta-analysis of the diagnostic accuracy of procalcitonin, C-reactive protein and white blood cell count for suspected acute appendicitis. *Br. J. Surg.* **100**, 322–329 (2013).
19. Ordóñez-Mena, J. M. *et al.* C-reactive protein and neutrophil count laboratory test requests from primary care: What is the demand and would substitution by point-of-care technology be viable? *J. Clin. Pathol.* **72**, 474–481 (2019).




For peer review only - http://bmjopen.bmj.com/site/about/guidelines.xhtml

For peer review only

20. Nepogodiev, D. *et al.* Appendicitis risk prediction models in children presenting with right iliac fossa pain (RIFT study): a prospective, multicentre validation study. *Lancet Child Adolesc. Heal.* **4**, 271–280 (2020).
21. Pogorelić, Z., Rak, S., Mrklić, I. & Jurić, I. Prospective validation of Alvarado score and pediatric appendicitis score for the diagnosis of acute appendicitis in children. *Pediatr. Emerg. Care* **31**, 164–168 (2015).
22. Gorter, R. R. *et al.* Diagnosis and management of acute appendicitis. EAES consensus development conference 2015. *Surg. Endosc. 2016 3011* **30**, 4668–4690 (2016).
23. Humes, D. J. & Simpson, J. Acute appendicitis. *Br. Med. J.* **333**, 530–531 (2006).
24. Moher, D., Liberati, A., Tetzlaff, J. & Altman, D. G. Preferred reporting items for systematic reviews and meta-analyses: the PRISMA statement. *BMJ* **339**, 332–336 (2009).
25. Whiting, P. F. *et al.* Quadas-2: A revised tool for the quality assessment of diagnostic accuracy studies. *Ann. Intern. Med.* **155**, 529–536 (2011).
26. Reitsma, J. B. *et al.* Bivariate analysis of sensitivity and specificity produces informative summary measures in diagnostic reviews. *J. Clin. Epidemiol.* **58**, 982–990 (2005).
27. Diaz, M. Performance measures of the bivariate random effects model for meta-analyses of diagnostic accuracy. *Comput. Stat. Data Anal.* **83**, 82–90 (2015).
28. RM Harbord, P Whiting, J. S. metandi: Stata software for statistically rigorous meta-analysis of diagnostic accuracy studies — University of Bristol. https://research-information.bris.ac.uk/en/publications/metandi-stata-software-for-statistically-rigorous-meta-analysis-o.
29. Steinhauser, S., Schumacher, M. & Rücker, G. Modelling multiple thresholds in meta-analysis of diagnostic test accuracy studies. *BMC Med. Res. Methodol.* **16**, 1–15 (2016).
30. Deeks, J. J., Macaskill, P. & Irwig, L. The performance of tests of publication bias and other sample size effects in systematic reviews of diagnostic test accuracy was assessed. *J. Clin. Epidemiol.* **58**, 882–893 (2005).
31. Andersson, R. E. *et al.* Diagnostic Value of Disease History , Clinical Presentation , and Inflammatory Parameters of Appendicitis. *World J. Surg.* **23**, 133–140 (1999).
32. Andersson, M. & Andersson, R. E. The appendicitis inflammatory response score: A tool for the diagnosis of acute appendicitis that outperforms the Alvarado score. *World J. Surg.* **32**, 1843–1849 (2008).
33. Thompson, G. C. *et al.* The use of routine blood cultures in pediatric appendicitis. *Pediatr. Emerg. Care* **33**, e160–e163 (2017).
34. Andersson, M. *et al.* Can new inflammatory markers improve the diagnosis of acute appendicitis? *World J. Surg.* **38**, 2777–2783 (2014).
35. Andersson, M. *et al.* Randomized clinical trial of Appendicitis Inflammatory Response score-based management of patients with suspected appendicitis. *Br. J. Surg.* **104**, 1451–1461 (2017).
36. González Del Castillo, J. *et al.* Diagnostic accuracy of the APPY1 Test in patients aged 2-20 years with suspected acute appendicitis presenting to emergency departments. *Emerg. Med. J.* **33**, 853–859 (2016).
37. Kaiser, M. *et al.* The diagnostic value of hepcidin to predict the presence and severity of appendicitis in children. *J. Surg. Res.* **222**, 102–107 (2018).
38. Khanafer, I. *et al.* Test characteristics of common appendicitis scores with and

For peer review only


For peer review only - http://bmjopen.bmj.com/site/about/guidelines.xhtml

without laboratory investigations: A prospective observational study. *BMC Pediatr.* **16**, 1–8 (2016).
39. Ozguner, I. F. *et al.* Are neutrophil CD64 expression and interleukin-6 early useful markers for diagnosis of acute appendicitis? *Eur. J. Pediatr. Surg.* **24**, 179–183 (2014).
40. Shommu, N. S. *et al.* The Use of Metabolomics and Inflammatory Mediator Profiling Provides a Novel Approach to Identifying Pediatric Appendicitis in the Emergency Department. *Sci. Rep.* **8**, 1–11 (2018).
41. Thompson, G. C. *et al.* Variation in the Diagnosis and Management of Appendicitis at Canadian Pediatric Hospitals. *Acad. Emerg. Med.* **22**, 811–822 (2015).
42. Dadeh AA, Puitong K. Predictive Factors to Diagnose Appendicitis in Children in the Emergency Department. Open Access Emerg Med 2021 Jul 29;13:363-372.
43. Mohammed, A. A., Daghman, N. A., Aboud, S. M. & Oshibi, H. O. The diagnostic value of C-reactive protein, white blood cell count and neutrophil percentage in childhood appendicitis. *Saudi Med. J.* **25**, 1212–1215 (2004).
44. van den Broek WT, van der Ende ED, Bijnen AB, Breslau PJ, Gouma DJ. Which children could benefit from additional diagnostic tools in case of suspected appendicitis? Journal of pediatric surgery. 2004;39(4):570-4.
45. Calvo Rigual, F. *et al.* [The value of C-reactive protein in the diagnosis of acute appendicitis in children]. *An. Esp. Pediatr.* **48**, 376–380 (1998).
46. Kouame, D. B. *et al.* La procalcitonine peut-elle aider au diagnostic des appendicites de l'enfant ? *Ann. Chir.* **130**, 169–174 (2005).
47. Samuel, M. Pediatric appendicitis score. *J. Pediatr. Surg.* **37**, 877–881 (2002).
48. Altali, K. *et al.* Escala INFURG-SEMES para el diagnóstico de apendicitis aguda en los pacientes de 2 a 20 años atendidos en los servicios de urgencias hospitalarios. *Emergencias (St. Vicenç dels Horts)* **84**, 231–236 (2017).
49. Huckins, D. S. *et al.* Prospective validation of a biomarker panel to identify pediatric ED patients with abdominal pain who are at low risk for acute appendicitis. *Am. J. Emerg. Med.* **34**, 1373–1382 (2016).
50. Huckins, D. S. *et al.* A novel biomarker panel to rule out acute appendicitis in pediatric patients with abdominal pain. *Am. J. Emerg. Med.* **31**, 1368–1375 (2013).
51. Anandalwar, S. P. *et al.* Use of white blood cell count and polymorphonuclear leukocyte differential to improve the predictive value of ultrasound for suspected appendicitis in children. *J. Am. Coll. Surg.* **220**, 1010–1017 (2015).
52. Corkum, K. S., Oyetunji, T. A., Grabowski, J. E., Rigsby, C. K. & Lautz, T. B. Absolute neutrophil count as a diagnostic guide for the use of MRI in the workup of suspected appendicitis in children. *J. Pediatr. Surg.* **54**, 1359–1364 (2019).
53. Esparaz, J. R. *et al.* A simple algorithm to improve quality while reducing resource utilization in evaluation of suspected appendicitis in children. *Am. J. Surg.* **217**, 469–472 (2019).
54. Malia, L. *et al.* Predictors for Acute Appendicitis in Children. *Pediatr. Emerg. Care* (2019).
55. Prada-Arias, M. *et al.* Diagnostic accuracy of fibrinogen to differentiate appendicitis from nonspecific abdominal pain in children. *Am. J. Emerg. Med.* **35**, 66–70 (2017).
56. Tuncer, A. A. *et al.* Can mean platelet volume, Neutrophil-to-Lymphocyte, Lymphocyte-to-Monocyte, Platelet-to-Lymphocyte ratios be favourable predictors for the differential diagnosis of appendicitis? *J. Pak. Med. Assoc.* **69**, 647–654 (2019).
57. Yazici M, Ozkisacik S, Oztan MO, Gursoy H. Neutrophil/lymphocyte ratio in the


For peer review only - http://bmjopen.bmj.com/site/about/guidelines.xhtml

diagnosis of childhood appendicitis. The Turkish journal of pediatrics. 2010;52(4):400-3.
58. Greer D, Bennett P, Wagstaff B, Croaker D. Lymphopaenia in the diagnosis of paediatric appendicitis: a false sense of security? ANZ J Surg 2019 Sep;89(9):1122-1125.
59. Oikonomopoulou, N. *et al.* Assessment of proadrenomedullin as diagnostic or prognostic biomarker of acute appendicitis in children with acute abdominal pain. *Am. J. Emerg. Med.* **37**, 1289–1294 (2019).
60. Blok GCGH, Veenstra LMM, van der Lei J, Berger MY, Holtman GA. Appendicitis in children with acute abdominal pain in primary care, a retrospective cohort study. Fam Pract 2021 Nov 24;38(6):758-765.
61. Verbakel, J. Y. *et al.* Point-of-care C reactive protein to identify serious infection in acutely ill children presenting to hospital: Prospective cohort study. *Arch. Dis. Child.* **103**, 420–426 (2018).
62. Reismann J, Schädlich D, Minderjahn MI, Rothe K, Reismann M. Eosinophilia in pediatric uncomplicated appendicitis is a time stable pattern. Pediatr Surg Int 2019 Mar;35(3):335-340.
63. Acharya, A., Markar, S. R., Ni, M. & Hanna, G. B. Biomarkers of acute appendicitis: systematic review and cost–benefit trade-off analysis. Surg. Endosc. 31, 1022–1031 (2017).
64. Shogilev, D. J., Duus, N., Odom, S. R. & Shapiro, N. I. Diagnosing appendicitis: Evidence-based review of the diagnostic approach in 2014. *West. J. Emerg. Med.* **15**, 859–871 (2014).
65. Benabbas, R., Hanna, M., Shah, J. & Sinert, R. Diagnostic Accuracy of History, Physical Examination, Laboratory Tests, and Point-of-care Ultrasound for Pediatric Acute Appendicitis in the Emergency Department: A Systematic Review and Meta-analysis. *Acad. Emerg. Med.* **24**, 523–551 (2017).
66. Horvath, A. R. *et al.* From biomarkers to medical tests: The changing landscape of test evaluation. *Clin. Chim. Acta* **427**, 49–57 (2014).
67. Lahaye, M. J. *et al.* Mandatory imaging cuts costs and reduces the rate of unnecessary surgeries in the diagnostic work-up of patients suspected of having appendicitis. *Eur. Radiol.* **25**, 1464–1470 (2015).
68. Gongidi, P. & Bellah, R. D. Ultrasound of the pediatric appendix. *Pediatr. Radiol.* **47**, 1091–1100 (2017).

For peer review only


For peer review only - http://bmjopen.bmj.com/site/about/guidelines.xhtml

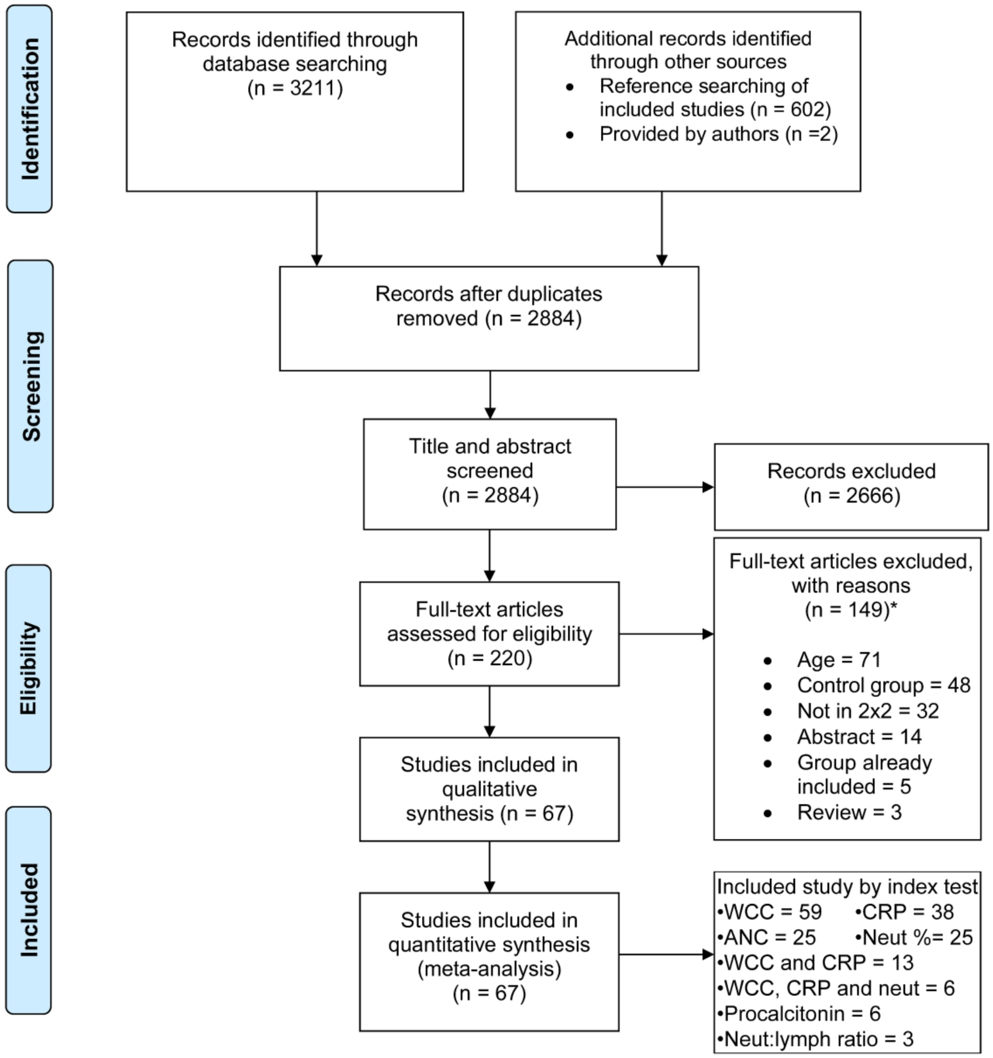


PRISMA flow chart for identification of included studies (*some studies excluded for 2 reasons, see full overview of excluded studies in Supplementary table 1)24

183x200mm (600 x 600 DPI)

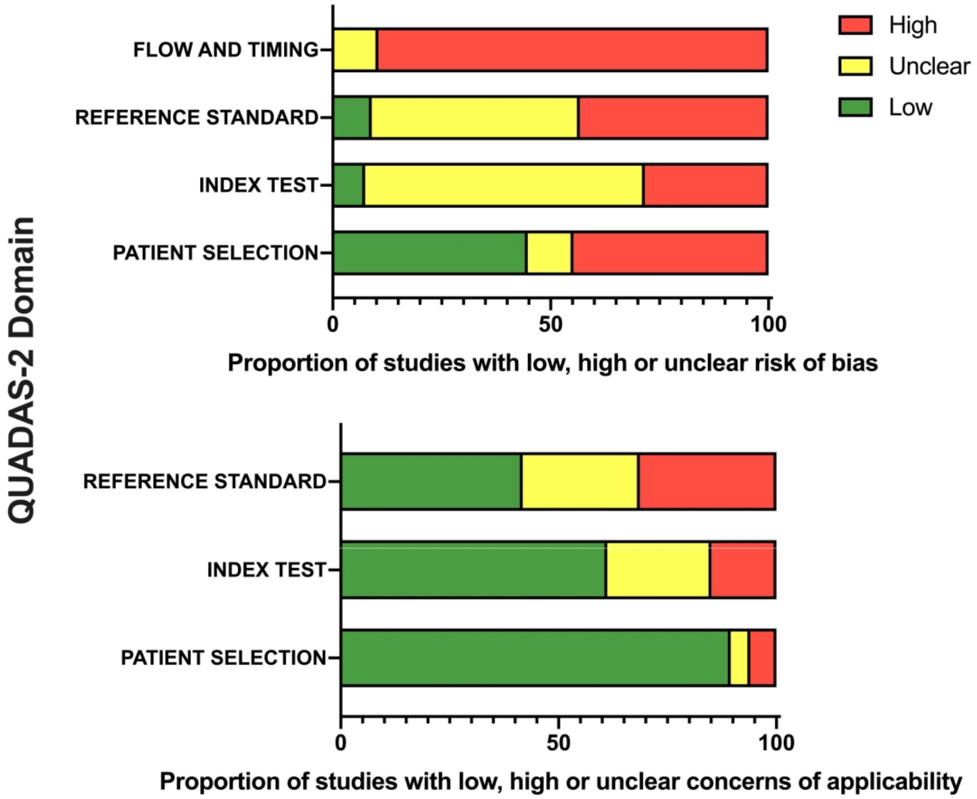


Figure 2: Overview of QUADAS-2 assessment of all included studies (n=67) showing review authors' assessment of risk of bias and study applicability in each domain reported as a percentage of all included studies (proportion coloured by assessment as high [red], unclear [yellow] or low [green], full outline of all individual components of QUADAS-2 is detailed in Supplementary table 4).

184x158mm (300 x 300 DPI)

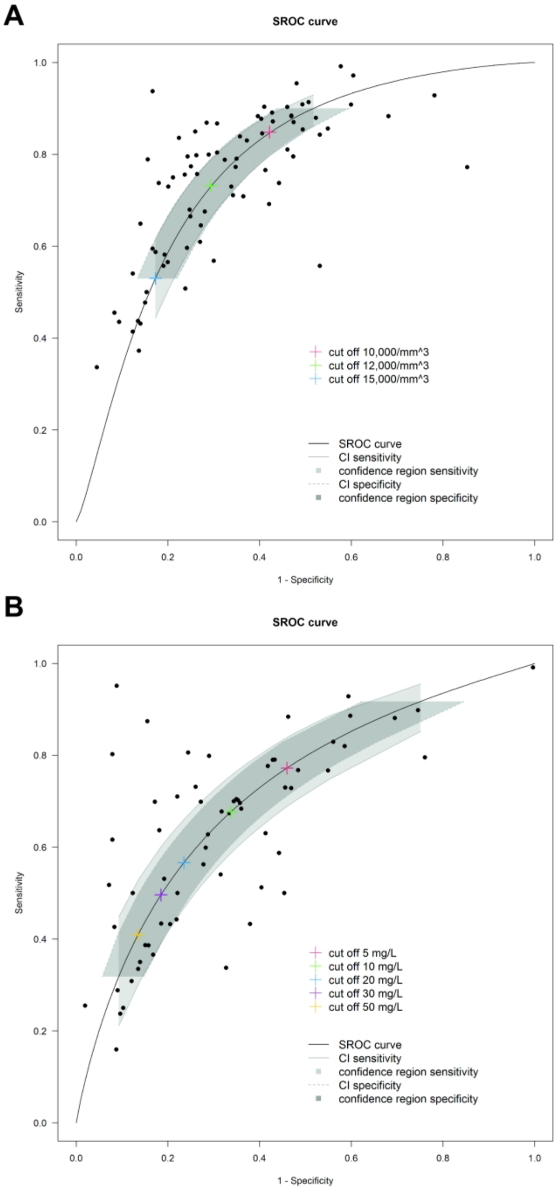


Figure 3: Summary receiver operating characteristic plot of WCC (A) and CRP (B) for diagnosis of paediatric acute appendicitis (Colour of dot representing study identified in legend, hashed grey box = 95% confidence interval specificity, solid grey box = 95% confidence interval sensitivity. Examples of commonly reported cut-offs shown in (A) as blue [10,000/µl], green [12,000/ µl] and red [15,000/ µl] crosses on curve; in (B) as yellow [5ml/L], pink [10mg/L], blue [20mg/L], green [30mg/L] and red [50mg/L] crosses on curve)

148x296mm (600 x 600 DPI)

# Supplementary Materials for "Diagnostic accuracy of blood tests in paediatric appendicitis – a systematic review and meta-analysis"

Overview of Supplementary materials

**Supplementary Tables:** ...2

*Supplementary Table 1* ...2

*Supplementary Table 2:* ...8

*Supplementary Table 3:* ...13

*Supplementary Table 4:* ...18

*Supplementary Table 5:* ...20

**Supplementary Figures and legends:** ...21

*Supplementary Figure 1:* ...21

*Supplementary Figure 2:* ...22

*Supplementary Figure 3* ...23

*Supplementary Figure 4* ...24

*Supplementary Figure 5* ...24

*Supplementary Figure 6* ...24

*Supplementary Figure 7* ...25

*Supplementary Figure 8* ...25

**Search Strategy:** ...26

**QUADAS-2 Scoring criteria** ...28

**Supplementary References** ...30

For peer review only

## Supplementary Tables:

**Supplementary Table 1:** Overview of studies excluded at full paper review stage and reason(s) for exclusion (* = Author was contacted and confirmed exclusion or did not respond. Unsuitable control group also includes those studies with no control group)

| First Author | Year | Exclusion Reason 1 | Exclusion Reason 2 |
|---|---|---|---|
| 1) Abu-Eshy | 1995 | Incompatible age demographics | Unsuitable control group |
| 2)Barker | 1998 | Unsuitable control group | Incompatible age demographics |
| 3)Bolandparvaz | 2004 | Incompatible age demographics | |
| 4)Bower | 1981 | Unsuitable control group | |
| 5)Clavien | 1987 | Incompatible age demographics | Unsuitable control group |
| 6)Cohen* | 2015 | Data not available in 2x2 | |
| 7)Depinet* | 2016 | Data not available in 2x2 | |
| 8)Erdem | 2015 | Incompatible age demographics | |
| 9)Ficek | 1958 | Unsuitable control group | |
| 10) Kharbanda | 2016 | Study population already included | |
| 11) Lofvenberg | 2016 | Unsuitable control group | Data not available in 2x2 |
| 12) Ng | 2002 | Incompatible age demographics | |
| 13) Park | 2010 | Incompatible age demographics | |
| 14) Vegar-Zubovic | 2003 | Unsuitable control group | |
| 15) Wattanasirichaigoon | 1994 | Incompatible age demographics | |
| 16) Anielski | 2010 | Incompatible age demographics | |
| 17) Atema | 2015 | Incompatible age demographics | |
| 18) Kessler | 2004 | Incompatible age demographics | |
| 19) Nasiri | 2012 | Incompatible age demographics | |
| 20) Thuijls | 2011 | Incompatible age demographics | |
| 21) Yardimci | 2016 | Incompatible age demographics | |
| 22) Yorke* | 2016 | Abstract | Incompatible age demographics |



For peer review only - http://bmjopen.bmj.com/site/about/guidelines.xhtml

| | | | |
|---|---|---|---|
| 23) Young | 1986 | Review | |
| 24) Agrawal | 2008 | Incompatible age demographics | Unsuitable control group |
| 25) Albu | 1994 | Incompatible age demographics | Data not available in 2x2 |
| 26) Allister* | 2011 | Incompatible age demographics | Data not available in 2x2 |
| 27) Amland | 1989 | Incompatible age demographics | |
| 28) Atema | 2015 | Incompatible age demographics | Unsuitable control group |
| 29) Bakal* | 2016 | Data not available in 2x2 | |
| 30) Bealer | 2010 | Incompatible age demographics | |
| 31) Beardsley* | 2014 | Abstract | |
| 32) Beardsley* | 2012 | Abstract | |
| 33) Bhatt* | 2009 | Data not available in 2x2 | |
| 34) Birkhahn | 2006 | Incompatible age demographics | |
| 35) Blab* | 2004 | Data not available in 2x2 | |
| 36) Cardall | 2004 | Incompatible age demographics | |
| 37) Chakhunashvili | 2005 | Unsuitable control group | |
| 38) Chandel | 2011 | Unsuitable control group | |
| 39) Colvin* | 2007 | Data not available in 2x2 | |
| 40) D'Souza | 2013 | Incompatible age demographics | |
| 41) Davies | 1991 | Incompatible age demographics | |
| 42) Dueholm | 1989 | Incompatible age demographics | |
| 43) Duzgun | 2007 | Incompatible age demographics | |
| 44) Eriksson | 1994 | Incompatible age demographics | |
| 45) Eriksson | 1995 | Incompatible age demographics | |
| 46) Eriksson | 1995 | Incompatible age demographics | |
| 47) Erkasap | 2000 | Incompatible age demographics | |
| 48) Escriba* | 2011 | Data not available in 2x2 | |
| 49) Gendel | 2011 | Unsuitable control group | Data not available in 2x2 |



For peer review only - http://bmjopen.bmj.com/site/about/guidelines.xhtml

| | | | |
|---|---|---|---|
| 50) Groselj-Grenc | 2007 | Study population already included | |
| 51) Gurau | 2016 | Unsuitable control group | Data not available in 2x2 |
| 51) Hallan | 1997 | Incompatible age demographics | |
| 53) Hotic | 2012 | Unsuitable control group | Data not available in 2x2 |
| 54) Hu* | 2014 | Data not available in 2x2 | |
| 55) Huang* | 2012 | Data not available in 2x2 | |
| 56) Jahn | 1997 | Incompatible age demographics | |
| 57) Jamaluddin | 2013 | Incompatible age demographics | |
| 58) Johansson | 2007 | Incompatible age demographics | |
| 59) John | 1993 | Incompatible age demographics | |
| 60) Khan | 2004 | Incompatible age demographics | |
| 61) Khan | 2009 | Incompatible age demographics | Unsuitable control group |
| 62) Kharbanda* | 2011 | Data not available in 2x2 | |
| 63) Kosloske* | 2004 | Data not available in 2x2 | |
| 64) Kostic* | 2010 | Data not available in 2x2 | |
| 65) Kyriakidis | 2010 | Incompatible age demographics | |
| 66) Lam | 2014 | Incompatible age demographics | |
| 67) Li | 2005 | Unsuitable control group | Data not available in 2x2 |
| 68) Li | 2012 | Unsuitable control group | |
| 69) Lietzen | 2016 | Incompatible age demographics | |
| 70) Lin* | 2007 | Data not available in 2x2 | |
| 71) Mollitt* | 1988 | Data not available in 2x2 | |
| 72) Oosterhuis | 1993 | Incompatible age demographics | |
| 73) Patel | 2014 | Unsuitable control group | |
| 74) Raftery | 1976 | Incompatible age demographics | Unsuitable control group |
| 75) Ring-Mrozik* | 1991 | Unsuitable control group | |
| 76) Sack* | 2006 | Unsuitable control group | |



For peer review only - http://bmjopen.bmj.com/site/about/guidelines.xhtml

| | | | |
|---|---|---|---|
| 77) Sarsu* | 2017 | Unsuitable control group | |
| 78) Schellekens | 2013 | Incompatible age demographics | |
| 79) Socea | 2013 | Incompatible age demographics | |
| 80) Sondenaa | 1992 | Incompatible age demographics | |
| 81) Suhaymi | 2017 | Unsuitable control group | Incompatible age demographics |
| 82) Summa | 2007 | Incompatible age demographics | |
| 83) Tamanna | 2012 | Incompatible age demographics | |
| 84) Tanrikulu | 2014 | Incompatible age demographics | |
| 85) Tepel | 2004 | Incompatible age demographics | |
| 86) Thimsen | 1989 | Incompatible age demographics | |
| 87) Thompson | 1992 | Incompatible age demographics | |
| 88) Turkyilmaz | 2000 | Unsuitable control group | |
| 89) Uyanik | 2012 | Unsuitable control group | |
| 90) van den Broek | 2002 | Study population already included | |
| 91) Wu | 2010 | Unsuitable control group | |
| 92) Wu | 2006 | Incompatible age demographics | |
| 93) Xharra | 2012 | Incompatible age demographics | |
| 94) Zviedre* | 2014 | Abstract | |
| 95) Brown* | 2004 | Abstract | |
| 96) Hodgkinson* | 2011 | Abstract | |
| 97) Hodgkinson* | 2011 | Abstract | |
| 98) Akin | 2018 | Unsuitable control group | |
| 99) Aydin | 2017 | Incompatible age demographics | |
| 100) Favot | 2017 | Review | |
| 101) Kharbanda* | 2018 | Study population already included | Data not available in 2x2 |
| 102) Mikaere | 2018 | Incompatible age demographics | |
| 103) Nazik | 2017 | Unsuitable control group | |


For peer review only - http://bmjopen.bmj.com/site/about/guidelines.xhtml

| | | | |
|---|---|---|---|
| 104) Nia | 2018 | Unsuitable control group | |
| 105) Salih | 2017 | Incompatible age demographics | Unsuitable control group |
| 106) Zouari | 2017 | Unsuitable control group | |
| 107) Daly | 2018 | Unsuitable control group | |
| 108) Elmas | 2017 | Unsuitable control group | |
| 109) Garcia Gamiz* | 2016 | Abstract | |
| 110) Oztan | 2018 | Unsuitable control group | |
| 111) Shommu | 2018 | Unsuitable control group | |
| 112) Yu* | 2018 | Unsuitable control group | |
| 113) Zouari | 2018 | Unsuitable control group | |
| 114) Sola | 2017 | Unsuitable control group | |
| 115) Zahoor* | 2018 | Abstract | |
| 116) van Dieijen-Visser | 1991 | Incompatible age demographics | |
| 117) Snyder | 1999 | Review | |
| 118) Sengupta | 2009 | Incompatible age demographics | Data not available in 2x2 |
| 119) Scott | 2015 | Incompatible age demographics | |
| 120) Randall Bond* | 1990 | Data not available in 2x2 | |
| 121) Msolli | 2018 | Incompatible age demographics | |
| 122) Malik | 1998 | Incompatible age demographics | |
| 123) Macco* | 2016 | Data not available in 2x2 | |
| 124) Lee | 2008 | Abstract | Incompatible age demographics |
| 125) Kohn | 2005 | Incompatible age demographics | |
| 126) Jang | 2008 | Incompatible age demographics | |
| 127) Haghi | 2018 | Incompatible age demographics | |
| 128) Blitman* | 2015 | Data not available in 2x2 | |
| 129) Akturk | 2018 | Incompatible age demographics | |
| 130) Ahmed | 2017 | Unsuitable control group | |



For peer review only - http://bmjopen.bmj.com/site/about/guidelines.xhtml

| | | | |
|---|---|---|---|
| 131) Agilinko | 2019 | Abstract | |
| 132) Benito | 2020 | Data not available in 2x2 | |
| 133) Boettcher | 2022 | Data not available in 2x2 | |
| 134) Dal | 2019 | Incompatible age demographics | |
| 135) Davis | 2021 | Data not available in 2x2 | |
| 136) Davis | 2019 | Abstract | |
| 137) Dooki | 2021 | Unsuitable control group | |
| 138) Duman | 2022 | Unsuitable control group | |
| 139) Duman | 2020 | Unsuitable control group | |
| 140) Güney | 2019 | Data not available in 2x2 | |
| 141) Lima | 2019 | Unsuitable control group | |
| 142) Morandi | 2020 | Unsuitable control group | |
| 143) Musbahi | 2019 | Unsuitable control group | Data not available in 2x2 |
| 144) Najd Sepas | 2019 | Unsuitable control group | Data not available in 2x2 |
| 145) Nawaz | 2021 | Abstract | |
| 146) Ng | 2019 | Abstract | |
| 147) Pehlivanli | 2019 | Incompatible age demographics | Unsuitable control group |
| 148) Saleem | 2021 | Incompatible age demographics | Unsuitable control group |
| 149) Altali Alhames | 2021 | Study population already included | |

For peer review only

**Supplementary Table 2:** Overview of demographics of included studies describing design, setting and population characteristics (* = data not provided or none described. AA= acute appendicitis. (P)ED = (Paediatric) Emergency Department. US = Ultrasound. CT = Computed Tomography. PICU= Paediatric Intensive Care Unit. In studies where authors provided paediatric data sub-population information is shown in square brackets.)

| Study | Study Design; Setting; Country | No. of children in study; with AA (% prevalence) | Age range (years) | Gender (n (%) male) | Inclusion criteria | Exclusion criteria |
|---|---|---|---|---|---|---|
| Akgül, 2021 (1) | Prospective cohort study; PED; Turkey | 320, 190 (59%) | 2 to 18 | 201 (63%) | Acute abdominal pain and Paediatric Appendicitis Score (PAS) above 3 points | Previous appendicectomy. Abdominal trauma. Malignancies. Inflammatory intestinal disease. Familial Mediterranean Fever. |
| Altali, 2017 (2) | Prospective cohort study; Hospital ED; Spain | 331, 116 (35%) | 2 to 20 | 175 (53%) | Abdominal pain <72 hours suspicious of AA as reviewed by doctor | Radiology prior to presentation. Pregnancy. Previous appendicectomy. Cancer. Trauma. Surgery in last 7 days. Steroids, immunosuppressive treatment or chemotherapy. |
| Anandalwar, 2015 (3) | Retrospective cohort study; ED; USA | 845, 393 (47%) | 3 to 18 | * (48%) | Consultation by surgeon for suspected AA. Underwent abdominal US. | History of abdominal surgery. Cognitive delay. Neurologic impairment. Immunodeficiency. Antibiotic use. Missing laboratory data. Abdominal imaging before presentation. |
| Andersson, 1999 (4) | Prospective cohort study; ED, 2 centres; Sweden | 496,[data provided n=296 <18yrs] 79(27%) | 10 to 86 | 133 (45%) | Patients admitted with suspected AA | * |
| Andersson, 2008 (5) | Prospective cohort study; Hospital, 2 centres; Sweden | 545 [data provided n=436 <18yrs, 140 of which not included in Andersson 1999] , 191[25](18%) | All | 58 (41%) | Admitted with suspected AA | Patients with missing blood tests. |
| Andersson, 2014 (6) | Prospective cohort study; Hospital ED, 2 centres; Sweden | 428 [data provided n=259 children], 177[76](30%) | 4 to 84 | 133 (49%) | Abdominal pain <5 days suggestive of AA | Pregnancy. Inability to consent. Multiple missing variables. |
| Andersson, 2017 (7) | Prospective interventional study with a nested randomized trial; ED, 21 centres; Sweden | 3791[data provided n=1046 children], [338](33%) | 5 to 95 | 2052 (54%) | Acute abdominal pain suggestive of AA | Pregnancy. Pain lasting >5 days. |
| Bachur 2016 (8) | Prospective cohort study; ED; USA | 2133, 870 (41%) | 3 to 18 | * (52%) | Acute abdominal pain < 72 hours. Had a WCC count and WCC differential obtained. | Pregnancy. Prior abdominal surgery. Chronic gastrointestinal condition. Severe developmental delay. |
| Bal 2016 (9) | Prospective cohort study; PED; Turkey | 96, 56 (58%) | 4 to 18 | 58 (21%) | Acute abdominal pain < 96 hours. Surgical consultation for suspected AA. Documented follow up. | Pregnancy. Familial Mediterranean fever. Chronic abdominal illness. Sickle cell anaemia. Cystic fibrosis. Previous abdominal surgery. Immunosuppressive therapy. |
| Beltran, 2007 (10) | Prospective Cohort study; Hospital; ED; Chile | 121, 71 (59%) | 5 to 14 | 73 (60%) | Presenting with abdominal pain, nausea, vomiting and fever. | Re-presentation. |
| Benito, 2016 (11) | Prospective cohort study; PED; Spain | 185, 89 (48%) | 2 to 14 | 123 (66%) | Abdominal pain suggestive of AA as evaluated by attending physician. | Symptoms >5 days. Prior appendicectomy. Urinary tract infection. Cancer. Inflammatory disease. Corticoids |
| Calvo, 1998 (12) | Retrospective cohort study; Hospital; Spain | 231, 141 (61%) | 2 to 14 | 149 (65%) | Acute abdominal pain. Inpatient. | Less severe cases of abdominal pain. |
| Cayrol 2016 (13) | Prospective cohort study; PED; Spain | 135, 52 (39%) | 1 to 15 | 69 (51%) | Children with acute abdominal pain suggestive of AA. | Previous appendicectomy. Immunological, haemostatic or thrombotic disorder. Vasculitis. Cardiopathy. Cancer. Pregnancy. |
| Chiang, 2020 (14) | Retrospective cohort study; ED; Singapore | 1391, 718 (52%) | 2-16 | 814 (59%) | Admitted with suspected AA based on clinical history and physical examination. | Pregnancy. Previous appendicectomy. Inflammatory disease. Cancer. |



For peer review only - http://bmjopen.bmj.com/site/about/guidelines.xhtml

| | | | | | | |
|---|---|---|---|---|---|---|
| Corkum, 2018 (15) | Retrospective cohort study; ED; USA | 738, 156 (21%) | 5 to 18 | * | Child with suspected AA -defined by undergoing an abdominal US and had blood test. | * |
| Dadeh, 2021 (16) | Retrospective cohort study; ED; Thailand | 1043, 97 (9%) | 3 to 14 | 565 (54%) | Acute abdominal pain. | Intra-abdominal organ disease. Previous abdominal surgery. Cancer. Incomplete medical records. |
| Doraiswamy 1979 (17) | Case control study; Hospital; UK | 375, 225 (60%) | 5 to 15 | * | Cases: children with AA. Controls: children with normal appendix on histology and children with acute abdominal pain. | Extra-abdominal complications. |
| Esparaz, 2019 (18) | Retrospective cohort study; ED; USA | 327, 56 (17%) | 2 to 17 | * | Abdominal US for suspected AA | None |
| Fleischman, 2013 (19) | Prospective cohort study; PED; USA | 178, 65 (37%) | 3 to 18 | 84 (47%) | Patients with symptoms suggestive of AA, duration <7 days. | Negative appendectomy but other diagnosis indicated by imaging/surgery |
| Goldman, 2008(20) | Prospective cohort study; ED; Canada | 849, 123 (36%) | 1 to 17 | * | Children with chief complaint of abdominal pain for <7 days. | Known diagnosis of AA (US or CT) on arrival to ED. Patients with prior appendicectomy. |
| Gonzalez del Castillo, 2016 (21) | Prospective cohort study; ED, 4 centres; Spain | 321, 111 (35%) | 2 to 20 | 167 (52%) | Abdominal pain <72 hours suggestive of AA | Ulcerative colitis. CT prior to consent. Pregnancy. Appendicectomy. Cancer. Trauma. Surgery within 7 days. Immunosuppressive treatment or chemotherapy. |
| Greer, 2019 (22) | Retrospective cohort study; ED; Australia | 546, 86 (16%) | 2 to 15 | 278 (51%) | Abdominal pain. | Trauma. Other confirmed diagnoses. Patients without blood test. |
| Groselj-Grenc 2007(23) | Nested case-control study; Hospital; Slovenia | 82, 49 (60%) | 3 to 14 | 53 (65%) | Cases: Children who underwent surgery for AA. Controls: Diagnosis of non-specific abdominal pain or sonographic mesenteric lymphadenitis. | Clinically or microbiologically established infections. |
| Hsiao 2005(24) | Case-control; Hospital; Taiwan | 222, 111 (50%) | 0 to 14 | * (ratio 1.52 M:1 F) | Cases: Diagnosis of AA on histology. Controls: Suspected AA in same period. | None |
| Huckins 2013(25) | Prospective cohort study; ED; 12 centres; USA | 503, 144 (29%) | 2 to 20 | 243 (48%) | Right lower quadrant/ generalized abdominal pain with other signs/ symptoms consistent with AA of duration < 72 hours. | Previous appendicectomy. Cancer. Bleeding disorder. Trauma. Invasive abdominal procedures or prior diagnostic imaging during this illness. Participation in other research protocols within 2 weeks. Unable to obtain consent. |
| Huckins 2016(26) | Prospective cohort study; ED; 29 centres; USA | 1887, 477 (25%) | 2 to 20 | 848 (45%) | Right lower quadrant/generalized abdominal pain <72 hours with other signs/symptoms suggesting AA. Informed consent. | Previous appendicectomy. Cancer. Bleeding disorder. Autoimmune disorder. Trauma. Immunosuppressive medications. Invasive abdominal procedure within 2 weeks. Surgery without imaging. Prisoners. Participation in other research protocol within 30 days. |
| Kaiser, 2018(27) | Case control study; Hospital; Austria | 64, 39(61%) | *(mean age 12 yr) | 24 (38%) | Cases: children who underwent appendectomy. Controls: abdominal pain and improved under conservative treatment | * |
| Kashtan, 2020 (28) | Retrospective cohort study; ED; USA | 2277, 974 (43%) | 3 to 18 | 1098 (48%) | Abdominal US and surgical consultation for possible AA. | Missing data. Prior abdominal surgery. Immunodeficiency. |
| Khan 2012 (29) | Prospective cohort study; PED; USA | 50, 22 (44%) | 5 to 17 | 22 (44%) | Right lower quadrant abdominal pain without signs of gastroenteritis. | Chronic haematological, immunological or gastrointestinal disease. Pregnancy. |
| Khanafer, 2016(30) | Prospective cohort study; paediatric ED; Canada | 180, 55(31%) | 5 to 17 | 78 (43%) | Abdominal pain <5 days. AA in differential from ED with bloods done. | Previous appendicectomy. Positive imaging prior to presentation. Pregnancy. Immuno-suppressive disorders. |
| Kharbanda 2005(31) | Prospective cohort study; PED; USA | 601, 211 (37%) | 3 to 18 | 307 (51%) | Surgical consultation for possible AA | Pregnant. Previous abdominal surgery. Chronic medical condition. Radiological study of abdomen within 2 weeks. |
| Kharbanda, 2012 (32) | Prospective cross-sectional study; PED; USA | 176, 58 (33%) | 3 to 18 | 92 (52%) | Acute abdominal pain <96 hours duration evaluated for AA as defined by clinician. | Pregnancy. Prior abdominal surgery, chronic GI illness. Inaccurate history. Imaging / abdominal trauma in <7 days. |
| Kharbanda, 2012 (33) | Prospective cross-sectional study; PED; 10 centres; USA | 2625, 1018 (39%) | 3 to 18 | 1339 (51%) | Abdominal pain <96 hours duration in keeping with AA (as defined by clinician). | Pregnancy. Prior abdominal surgery. Chronic abdominal illness. Sickle cell. Cystic fibrosis. Radiological investigation diagnosing AA before arrival. Trauma within 7 days |


For peer review only - http://bmjopen.bmj.com/site/about/guidelines.xhtml

| | | | | | | |
|---|---|---|---|---|---|---|
| Klein, 2021 (34) | Prospective cohort study; PED; Israel | 134, 53 (40%) | 3 to 17 | 71 (53%) | Admitted with suspected AA | Previous appendectomy. Cancer. Haematological disorders. Active auto-inflammatory disorders. Trauma. Abdominal surgery. Previously known infectious disease such as urinary tract infection or pneumonia. Previously known urological/gynecological disorders such as pelvic inflammatory disease. |
| Ko, 1995 (35) | Prospective cohort study; Hospital; Taiwan | 47, 27 (57%) | 2 to 14 | 29 (62%) | Patients with clinically suspected AA | Atypical clinical signs. Didn't complete protocol. |
| Kouame, 2005 (36) | Prospective diagnostic accuracy study; Hospital; France | 101, 68 (67%) | 2 to 15 | 52 (51%) | Hospitalised children with abdominal pain that was suspected to require surgery. | Antibiotics prior to hospital presentation. |
| Kumar, 2021 (37) | Prospective observational study; tertiary PED; India | 120, 55 (46%) | 5 to 12 | Unknown | Acute right lower abdomen pain. | Duration of symptoms <6 h or >72 h. Haematological disorders. Oncological disorders. Hepatic dysfunction. Infectious, or inflammatory conditions. Anticoagulants, anti-inflammatory medications or antibiotics. |
| Kwan, 2010(38) | Prospective observational study; PED; USA | 209, 115 (55%) | 1 to 18 | 123 (59%) | Abdominal pain suspicious of AA. | Pregnancy. Bleeding disorder. Severe anaemia. Chronic disease. Abdominal surgery in the previous year. |
| Lin, 2009(39) | Retrospective cohort study; ED; Taiwan | 100, 53 (53%) | 2 to 17 | 46(46%) | Children with RLQ pain presenting to ED | Patients who went home against medical advice. |
| Lycopoulou, 2005(40) | Prospective observational with nested case-control study; PED; Greece | 60, 42 (70%) | 5 to 14 | 30 (50%) | A nested control of children operated for AA; Controls: children presenting to PED during same period with symptoms and signs of AA | No consent. Retrocaecal abscess. Normal appendix histology. |
| Malia, 2019(41) | Prospective cohort study; Paediatric hospital ED; USA | 762, 225 (30%) | 0 to 18 | 356 (47%) | Children with suspected AA who underwent US | Patient absconded, previous appendicectomy, appendix not visualised on US, US at other facility. |
| Mandeville, 2011(42) | Prospective observational study; PED; USA | 287, 155 (54%) | 4 to 17 | 151 (53%) | Abdominal pain suggestive of AA | Pregnancy, previous abdominal surgery, chronic medical condition, non-verbal, radiological investigation of abdomen in preceding 2 weeks, no follow up. |
| Miguez, 2016(43) | Prospective cohort study; Paediatric hospital ED; Spain | 136, 44 (32%) | 3 to 16 | 76 (56%) | Admitted to ED with abdominal pain <96 hours AA suspected at initial evaluation. | Appendicectomy /recent surgery. Immune disease. Chronic respiratory or cardiovascular disease. Inflammatory bowel disease. Antibiotics or steroids in the last month. |
| Mohammed, 2004(44) | Prospective cohort study; Hospital paediatric surgical department; Libya | 216, 130 (60%) | 3 to 13 | 139(64%) | Child presenting with clinical suspicion of AA | * |
| Moreno, 2012(45) | Retrospective Cohort study; Hospital; Spain | 184, 85 (46%) | * (Mean age 8.9 yr) | 110 (60%) | Acute abdominal pain suggestive of AA. | * |
| Naqvi, 2019(46) | Observational cohort study; Tertiary Paediatric Hospital; Canada | 185, 79 (43%) | 4 to 17 | 86 (46%) | Children with suspected AA demonstrated by US or referral to paediatric surgical team. Also children with sepsis defined by SIRS criteria or PICU admission (AA sub-group reported) | Previous appendicectomy, resuscitation in ED, discharge to PICU, pregnancy, AP >5 days, immune-suppression, abdominal imaging prior to admission, life expectancy <24 hours. |
| Oikonomopoulou, 2019(47) | Prospective cohort study; PEDs, 6 centres; Spain | 285, 100 (35%) | 0 to 18 | 167 (59%) | Clinical suspicion of AA after physician assessment | Pain >72hr, lack of blood sample, history of surgery (within 3 months)/ immune pathology/ inflammatory bowel disease/ cardio-respiratory disease, recent steroid or antibiotic use |

For peer review only

| | | | | | | |
|---|---|---|---|---|---|---|
| Ovrebo, 1993(48) | Retrospective case series; Hospital surgical department; Norway | 470, 161 (34%) | 0 to 15 | 235 (50%) | All patients presenting with abdominal pain <1 week duration suggestive of AA. | Abdominal trauma. Incarcerated herniae. |
| Ozguner, 2014(49) | Prospective cohort study; Children's hospital; Turkey | 49, 34(69%) | 3 to 17 | 34 (69%) | Children hospitalised with right lower-quadrant pain suspicious of AA between 0400 and 1500. | If sample for CD64 measurement could not be obtained. |
| Peltola, 1986(50) | Prospective cohort study; Children's hospital; Finland | 162, 94 (58%) | 1 to 16 | 77 (48%) | Children presenting with abdominal pain or symptoms suggestive of AA | Symptoms suggestive or respiratory infection. |
| Prada-Arias, 2017(51) | Prospective cohort study; Tertiary hospital PED; Spain | 275, 132 (48%) | 5 to 15 | 153 (56%) | Clinical suspicion of AA with blood tests and US performed | Symptom duration <6hr or >72hr. Haematological / oncological / hepatic / infectious or inflammatory disease present. >12 hours between blood test and surgery |
| Rodríguez Sánchez de la Blanca, 2014(52) | Prospective cohort study, PED, Spain | 105, 41 (39%) | <16 | 59 (56%) | Abdominal pain or clinical suspicion of AA | Antibiotic treatment in the preceding 10 days, known inflammatory pathology |
| Salo, 2016(53) | Case control study; PED; Sweden | 44, 22 (50%) | 3 to 14 | 27 (61%) | Referral to paediatric surgeon with suspicion of AA | Immuno-modulating therapy or clear-cut diagnosis of AA |
| Samuel, 2002(54) | Prospective cohort study; Hospital, 2 centres; UK | 1170, 734 (63%) | 4 to 15 | 752(64%) | Abdominal pain suggestive of AA | Appendicula mass with peri-appendiceal abscess |
| Sanchez, 1998(55) | Prospective cohort study; Paediatric hospital ED; Spain | 195, 94 (48%) | 2 to 14 | 107(55%) | Children with suspected AA | * |
| Shommu, 2018(56) | Prospective cohort study; PED; Canada | 140, 52 (36%) | 5 to 17 | 61 (44%) | Clinically suspected AA or US evaluation for AA with IV placed and did not require intensive care treatment from PED. Informed consent | Previous appendectomy. Pregnancy. Abdominal pain >5 days. Immuno-suppression. Previously enrolled in the study. Imaging study performed elsewhere |
| Thompson, 2015(57) | Retrospective cohort study; ED, 12 centres; Canada | 619, 510 (83%) | 3 to 17 | 352 (53%) | Children admitted with suspected AA | Intensive care unit admission. |
| Thompson, 2016 (58) | Retrospective cohort study; Children's hospital ED; Canada | 1315, 561[data provided n=1138,471 children] (41%) | 2 to17 | 565 (43%) | Children investigated for suspected AA with blood culture sample collected | * |
| Tuncer, 2019(59) | Retrospective cross-sectional study; Paediatric Surgical/Medical department; Turkey | 301; 137 (46%) | *(Mean age 11yr) | 139 (46%) | Children with acute abdominal pain, recorded diagnosis of acute appendicitis, mesenteric lymphadenitis or familial Mediterranean fever. | Heart failure, peripheral vascular / haematological /liver disease, anticoagulation, steroid use, concurrent acute or chronic infection |
| Turkyilmaz, 2006(60) | Prospective cohort study; Hospital; Turkey | 105, 61 (58%) | 3 to 16 | 68 (65%) | Admission with acute right-lower abdominal pain | * |
| Van Den Broek, 2004(61) | Prospective cohort study; ED, 2 centres; Netherlands | 275, 169 (66%) | *(Mean age 9 yr) | 148 (58%) | GP referral with suspicion of AA | * |
| Wang 2007(62) | Prospective and retrospective cohort study; PED; USA | 722, 74 (18%) | 1 to 19 | 334 (46%) | Prospective: ED presentation with suspected AA. Retrospective: AA on surgery, radiology, and laboratory records. | History of trauma |
| Wu, 2012(63) | Prospective cohort study; ED; Taiwan | 594, 306 (52%) | 4 to 18 | 358 (60%) | Children with clinically suspected AA, as determined by emergency physician. | Symptoms >3 day. Patients lost to follow up. |
| Yap, 2015(64) | Prospective cohort study; Paediatric hospital ED; Singapore | 747, 229 (32%) | 4 to 16 | 429(57%) | Admitted to ED with suspected AA. | Previous appendicectomy, pregnancy, chronic medical or malignant conditions. CT in the previous month. |


For peer review only - http://bmjopen.bmj.com/site/about/guidelines.xhtml

| | | | | | | |
|---|---|---|---|---|---|---|
| Yazici, 2010(65) | Retrospective case series; Paediatric surgical department; Turkey | 240, 183 (76%) | 3 to 16 | * | Patients with acute abdominal pain | Urinary tract infection, Henoch-Schonlein pupura or gastroenteritis. |
| Zouari, 2016(66) | Prospective cohort study; Paediatric surgical department; Tunisia | 292, 132 (45%) | 3 to 14 | 170(58%) | Diagnosis of suspected AA by consultant paediatric surgeon on the basis of clinical history and physical exam | Patients who had not undergone US |
| Zuniga, 2012(67) | Prospective cohort study; Hospital ED; Spain | 101, 28 (28%) | 0 to 14 | 55 (54%) | Presenting with clinical suspicion of AA | Pain >7 days. Previous appendicectomy. No blood tests. |

For peer review only


For peer review only - http://bmjopen.bmj.com/site/about/guidelines.xhtml

**Supplementary Table 3:** Overview of included studies Index tests, cut off values and reference standards. (CRP = C-reactive protein. WCC = White Cell Count. PMN = Polymorphonuclear cells. * = non-AA not defined/not clearly defined, †=multiple cut offs or range in paper, ‡=author provided raw data)

| Study | Reference Standard | Index Test(s) measured | Index test cut off value(s) |
|---|---|---|---|
| Akgül, 2021 (1) | Histopathology or 14 days follow up | • WCC<br>• Neutrophil count<br>• CRP<br>• Procalcitonin | 10,000 μL<br>7500 μL<br>5 mg/L<br>0.5 ng/mL |
| Altali, 2017 (2) | Histopathology or discharge diagnosis | • WCC<br>• Neutrophil count<br>• CRP | >10,500/mm$^3$<br>>6,600/mm$^3$<br>>5mg/L |
| Anandalwar, 2015 (3) | Pathology proven appendicitis * | • WCC<br>• Neutrophil % | >9,000/mm$^3$<br>>65% |
| Andersson, 1999 (4) | Operation note and histopathology or failure to re-present | • WCC<br>• Neutrophil count<br>• Neutrophil %<br>• CRP<br>WCC/CRP as separate and combined | ‡ |
| Andersson, 2008 (5) | Histopathology or review of case files in the following 1 month. | • WCC<br>• Neutrophil count<br>• Neutrophil %<br>• CRP<br>WCC/CRP as separate and combined | ‡ |
| Andersson, 2014 (6) | Histopathology or 6 month follow up | • WCC<br>• Neutrophil count<br>• Neutrophil %<br>• CRP<br>WCC/CRP as separate and combined | ‡ |
| Andersson, 2017 (7) | Histopathology or 30 day follow up | • WCC<br>• Neutrophil count<br>• Neutrophil %<br>• CRP<br>WCC/ CRP as separate and combined | ‡ |
| Bachur 2016 (8) | Operative diagnosis, telephone follow-up (1 -2 weeks) or record review (90 days) after visit. | • WCC<br>• Neutrophil count | 5-15,000/mm$^3$† <br>5-15,000/mm$^3$† |
| Bal 2016 (9) | Surgery and pathology report or follow-up by phone interview 7-14 days after visit | • WCC<br>• Neutrophil count<br>• CRP<br>• Procalcitonin | >13,400/mm$^3$<br>>9,400/mm$^3$<br>>0·9mg/L<br>0·37ng/mL |
| Beltran, 2007 (10) | Histopathological report or discharge diagnosis | • WCC<br>• CRP | >12,500/mm$^3$<br>>47mg/L |


For peer review only - http://bmjopen.bmj.com/site/about/guidelines.xhtml
For peer review only

| | | | |
|---|---|---|---|
| Benito, 2016 (11) | Histopathological analysis of surgical specimen, telephone follow up 15 days after consultation, or check of electronic registry if not contactable. | • WCC<br>• Neutrophil count<br>• CRP<br>• Procalcitonin | <10,000/mm$^3$<br><7,500/mm$^3$<br><20 mg/L<br><0·1ng/ml |
| Calvo, 1998 (12) | Histopathological examination or discharge diagnosis | • WCC<br>• CRP<br>Tests reported as separate and as combination | >15,000/mm$^3$<br>>20mg/l |
| Cayrol 2016 (13) | Surgery and histopathology or clinical decision with telephone follow up 5 days later | • WCC<br>• CRP | >10,000/mm$^3$<br>>30mg/L |
| Chiang, 2020 (14) | Histopathology or 24 hours of clinical observation with or without radiologic imaging and with 3 days follow up after discharge. | • WCC<br>• Neutrophil %<br>• CRP | ≥10,000/μL<br>≥75%<br>≥5 mg/L |
| Corkum, 2018 (15) | Histopathology and operative note or 30 day follow up | • Neutrophil count | >8,000/mm$^3$ |
| Dadeh, 2021 (16) | Histopathology * | • WCC<br>• Neutrophil % | >10,000 or >14,000<br>>75%<br>(based on duration of pain less or more than 24 hours) |
| Doraiswamy 1979 (17) | Appendicitis confirmed or refuted at surgery or recovered without surgery | • WCC<br>• Neutrophil % | >15,000/mm$^3$ or >13,000/mm$^3$[†]<br>>50%, >65% or >75%[†]<br>(age dependent) |
| Esparaz, 2019 (18) | Histopathology * | • WCC | >10,000/mm$^3$ |
| Fleischman, 2013 (19) | Surgical pathology, positive CT scan finding or diagnosis and follow up at 2 weeks | • WCC<br>• Neutrophil count<br>• Neutrophil % | >10,000/mm$^3$<br>>6750/mm$^3$<br>>75% |
| Goldman, 2008(20) | Appendicectomy pathology or follow-up 5 to 7 days after ED discharge | • WCC<br>• Neutrophil count | >10,000/mm$^3$<br>>7,500/mm$^3$ |
| Gonzalez del Castillo, 2016 (21) | Histopathology reported by blinded pathologist or telephone follow up 2 weeks after discharge | • WCC<br>• CRP | >12,000/mm$^3$<br>>5mg/L |
| Greer, 2019 (22) | Histopathology or discharge diagnosis | • WCC<br>• Neutrophil count<br>• CRP | >13.5×10$^9$/L<br>>7.5×10$^9$/L<br>≥5 mg/L |
| Groselj-Grenc 2007(23) | Appendicitis diagnosed by surgery or ultrasound, or diagnosis of non-specific abdominal pain/sonographic mesenteric adenitis | • WCC<br>• CRP | >11,600/mm$^3$<br>>9.0mg/L |
| Hsiao 2005(24) | Surgery or pathology report, or appendicitis excluded in final diagnosis | • WCC<br>• Neutrophil % | >10,400/mm$^3$<br>>75% |
| Huckins 2013(25) | Surgical pathology report after appendectomy or discharge diagnosis | • WCC<br>• CRP<br>Tests only reported in combination | >10,000/mm$^3$<br>>8mg/L |
| Huckins 2016(26) | Surgical pathology reports after appendectomy or follow-up telephone calls at 14 days (±3 days) | • WCC<br>• CRP<br>Tests only reported in combination | >10,000/mm$^3$<br>>8mg/L |
| Kaiser, 2018(27) | Histopathology or discharge diagnosis | • WCC<br>• CRP | >10 -15,000/mm$^3$[†]<br>5-50mg/L[†] |



For peer review only - http://bmjopen.bmj.com/site/about/guidelines.xhtml

| | | | |
|---|---|---|---|
| | | Tests as separate and combined | |
| Kashtan, 2020 (28) | Histopathology or 7 days follow up | • WCC | >9000 cells/uL |
| Khan 2012 (29) | Histopathological evidence or telephone follow up 24 hour and 2 weeks after discharge | • Procalcitonin | >0·39ng/ml |
| Khanafer, 2016(30) | Histopathology or follow up 2 weeks after discharge | • WCC | 10-15,000m/$^{3\dagger}$ |
| Kharbanda 2005(31) | Pathology or follow-up telephone call 2 to 4 weeks after the ED visit | • WCC<br>• Neutrophil count | >8850/mm$^3$<br>6·75x10$^3$/ul |
| Kharbanda, 2012 (32) | Surgical histology or telephone follow up 14-21 days after admission/note review if uncontactable. | • WCC | >8850/mm$^3$ |
| Kharbanda, 2012 (33) | Histology or telephone follow up 2 weeks after discharge or note review 90 days after discharge if unable to contact. | • Neutrophil count | >6750/mm$^3$ |
| Klein, 2021 (34) | Histopathology or discharge diagnosis or clinical observation | • WCC<br>• Neutrophil % | > 12,000 or > 10,500<br>≥75%<br>(age dependent) |
| Ko, 1995 (35) | Surgical histology or diagnosis on discharge | • WCC<br>• Neutrophil %<br>• CRP | >10,000/mm$^3$<br>>75%<br>>9mg/L and >50mg/L |
| Kouame, 2005 (36) | Histology * | • Procalcitonin | >0.5µg/L |
| Kumar, 2021 (37) | Histopathology or 2 weeks follow up | • WCC<br>• Neutrophil count<br>• CRP | 14,930/mm$^3$<br>10,485.5 cell/ul<br>10.5 mg/L |
| Kwan, 2010(38) | Operative reports and histology or telephone follow up or note review 2-6 weeks after discharge | • WCC<br>• CRP<br>Tests reported as separate and in combination | >12,000/mm$^3$<br>>30mg/L |
| Lin, 2009(39) | Appendectomy (histologic examination) or telephone follow-up within 1 week of consultation | • WCC<br>• CRP | >15,000/mm$^3$<br>>1 mg/dL |
| Lycopoulou, 2005(40) | Appendicectomy (histological examination) or 1 week follow up | • WCC<br>• CRP | >10,000/mm$^{3\dagger}$<br>>10mg/L |
| Malia, 2019(41) | Histopathology report or failure to re-attend within 1 week | • WCC<br>• CRP | >10,000/mm$^3$<br>>5mg/L |
| Mandeville, 2011(42) | Histopathology or telephone follow up at 2 weeks | • WCC<br>• Neutrophil % | >10,000/mm$^3$<br>>75% |
| Miguez, 2016(43) | Histopathology of appendicectomy specimen or telephone follow up 5-7 days afterwards. | • WCC<br>• Neutrophil count<br>• CRP | >12,300/mm$^3$<br>>9,600/mm$^3$<br>>3 mg/L |
| Mohammed, 2004(44) | Appendicectomy (histopathological results) or clinical observation | • WCC<br>• Neutrophil %<br>• CRP<br>Tests reported as separate and in combination (all three) | >11,000/mm$^3$<br>>75%<br>>8mg/L |
| Moreno, 2012(45) | Surgery, histology, clinical observation or follow up | • WCC<br>• CRP | >12,800/mm$^3$<br>>18mg/L |

For peer review only

| | | | |
|---|---|---|---|
| Naqvi, 2019(46) | Histopathology, insertion of percutaneous drain or failure to re-attend within 2 weeks | • CRP | >7·9mg/L |
| Oikonomopoulou, 2019(47) | Histopathology and surgeons notes, telephone follow up after 5-7 days or checked for readmission if no response | • WCC<br>• Neutrophil count<br>• CRP<br>Tests reported as separate and in paired combinations | >13,300/$mm^3$<br>>10,800/$mm^3$<br>>12·5mg/L |
| Ovrebo, 1993(48) | Operative note (all) and histology (some) or discharge diagnosis | • WCC<br>• CRP<br>Tests reported as separate and in combination | >11,000/$mm^3$<br>>10mg/L |
| Ozguner, 2014(49) | Operative findings, histopathological examination and discharge diagnosis | • WCC<br>• CRP | >12,000/$mm^3$<br>>16mg/L |
| Peltola, 1986(50) | Histology on those operated or diagnosis of non-specific abdominal pain | • WCC<br>• CRP<br>Tests reported as separate and in combination | >14,900/$mm^3$<br>>10mg/L |
| Prada-Arias, 2017(51) | Histology and surgical report or no specific disease identified, no findings on US and no antibiotic treatment | • WCC<br>• Neutrophil count<br>• CRP | >11,000/$mm^3$<br>>7,000/$mm^3$<br>>25·2mg/L |
| Rodríguez Sánchez de la Blanca, 2014(52) | Histopathology* | • WCC<br>• CRP<br>• Procalcitonin | >13,000/$mm^3$<br>>5mg/L<br>>0·5μg/L |
| Salo, 2016(53) | Surgical note and histopathology report or follow up | • WCC<br>• Neutrophil count<br>• Neutrophil %<br>• CRP<br>Combined WCC and neutrophil count reported | >13-16,000/$mm^{3\dagger}$<br>>6·5-7,000/$mm^{3\dagger}$<br>>71%<br>>15mg/L<br>(ranges age dependent) |
| Samuel, 2002(54) | Histology in surgical group* | • WCC<br>• Neutrophil count | >10,00/$mm^3$<br>>7,500/$mm^3$ |
| Sanchez, 1998(55) | Histology and surgical report or discharge diagnosis | • WCC<br>• CRP | >14000/$mm^3$<br>>30mg/L |
| Shommu, 2018(56) | Histopathology* | • WCC<br>• Neutrophil count<br>• CRP | ‡ (Provided 2x2 tables for multiple cut offs of tests but not IPD) |
| Thompson, 2015(57) | Histopathology or period of observation | • WCC<br>• Neutrophil count<br>• CRP | ‡ |
| Thompson, 2016 (58) | Histopathology or discharge diagnosis | • WCC<br>• Neutrophil count<br>• CRP | ‡ |
| Tuncer, 2019(59) | Histopathology or discharge diagnosis | • WCC | >13,050/$mm^3$ |
| Turkyilmaz, 2006(60) | Histopathology and operative assessment or clinical observation | • WCC | >15000/$mm^3$ or 13,000/$mm^{3\dagger}$ (age dependent) |
| Van Den Broek, 2004(61) | Histology* | • WCC | >10,000/$mm^3$ |
| Wang 2007(62) | Histology or review of medical records | • WCC | †(Age dependent cut-off) |


For peer review only - http://bmjopen.bmj.com/site/about/guidelines.xhtml

| | | | |
|---|---|---|---|
| | | • Neutrophil %<br>Tests reported as separate and in combination | >80% |
| Wu, 2012(63) | Surgery, histological examination or follow up interview two weeks after visit | • WCC<br>• CRP | >11000/mm$^3$<br>>25 and 89mg/L |
| Yap, 2015(64) | Histology, operative findings or observation on the ward until >24 hours pain free | • WCC<br>• Neutrophil %<br>• CRP<br>Tests reported as separate and in combination (all three) | > 10,000/mm$^3$<br>>75%<br>>5mg/L |
| Yazici, 2010(65) | Histopathology or observation for 24-48 hours | • WCC | >10,000/mm$^3$ |
| Zouari, 2016(66) | Histology showing acute appendicitis or failure to represent for 2 weeks | • CRP | 6mg/L |
| Zuniga, 2012(67) | Histopathology or failure to re-present for 7 days | • WCC<br>• Neutrophil count | >10,000/mm$^3$<br>>7,500/mm$^3$ |


For peer review only - http://bmjopen.bmj.com/site/about/guidelines.xhtml

**Supplementary Table 4:** Individual assessment scoring of each included study assessed by means of the QUADAS-2 tool for risk of bias.

| Study | RISK OF BIAS | | | | APPLICABILITY CONCERNS | | |
|---|---|---|---|---|---|---|---|
| | PATIENT SELECTION | INDEX TEST | REFERENCE STANDARD | FLOW AND TIMING | PATIENT SELECTION | INDEX TEST | REFERENCE STANDARD |
| Akgül, 2021 | High | High | Low | High | Low | Low | Low |
| Altali, 2017 | Low | Unclear | Low | Unclear | Low | High | Low |
| Anandalwar, 2015 | High | High | Unclear | Unclear | Low | High | Unclear |
| Andersson, 1999 | Unclear | High | High | High | Unclear | Unclear | High |
| Andersson, 2014 | Low | Unclear | Unclear | High | Low | Unclear | Low |
| Andersson, 2017 | Low | Unclear | Unclear | High | Low | Low | Low |
| Andersson,2008 | Low | Unclear | Unclear | High | Low | Unclear | Low |
| Bachur, 2016 | Low | Unclear | Unclear | High | Low | Unclear | Unclear |
| Bal, 2016 | High | Unclear | Unclear | High | Low | Low | Low |
| Beltran, 2007 | Low | Low | High | Unclear | Low | Low | Unclear |
| Benito, 2016 | High | Low | High | High | Low | Low | Low |
| Calvo Rigual, 1998 | Low | Unclear | High | High | Low | Low | Low |
| Cayrol, 2016 | High | Low | High | High | Low | Low | High |
| Chiang, 2020 | Low | Unclear | High | High | Low | Low | High |
| Corkum, 2018 | High | Unclear | Unclear | High | Low | Unclear | Low |
| Dadeh, 2021 | Unclear | Unclear | Unclear | Unclear | Low | Unclear | High |
| Diaz Moreno, 2012 | Low | High | Unclear | Unclear | Low | Unclear | Unclear |
| Doraiswamy, 1979 | High | Unclear | Unclear | High | Low | Low | Unclear |
| Esparaz, 2019 | High | Unclear | High | High | Low | Low | Low |
| Fleischman, 2013 | High | Unclear | Unclear | High | Low | Low | Low |
| Goldman, 2008 | Low | Unclear | High | High | Low | Low | High |
| Gonzalez, 2016 | Low | High | High | High | Low | High | Low |
| Greer, 2019 | High | Unclear | High | High | Low | Low | High |
| Groselj-Grenc, 2007 | Low | High | Unclear | High | Low | Low | High |
| Hsiao, 2005 | High | Unclear | Unclear | High | Low | Low | Unclear |
| Huckins, 2013 | High | Low | High | High | High | Low | High |
| Huckins, 2016 | High | Low | Low | High | High | Low | Low |
| Kaiser, 2018 | High | Unclear | High | High | Low | Unclear | High |
| Kashtan, 2020 | Unclear | Unclear | High | High | Low | Low | Low |
| Khan, 2012 | Low | High | Unclear | Unclear | Low | High | Low |
| Khanafer, 2016 | High | Unclear | Unclear | High | Low | Low | Unclear |
| Kharbanda, 2005 | High | Unclear | Unclear | High | Low | Low | Low |
| Kharbanda, 2012 | Low | High | High | High | Low | Low | Low |
| Kharbanda, 2012 | High | Unclear | Unclear | High | Low | Low | Low |
| Klein, 2021 | High | Unclear | High | High | Low | Low | High |
| Ko, 1995 | Low | Unclear | Unclear | High | Low | Low | Unclear |
| Kouame, 2005 | Low | Unclear | Unclear | Unclear | Low | Low | Unclear |
| Kumar, 2021 | High | Unclear | Low | High | Low | Low | Low |
| Kwan, 2010 | Low | High | Unclear | High | Low | Low | Low |
| Lin, 2009 | Low | Unclear | High | High | Low | High | High |
| Lycopoulou, 2005 | High | Unclear | High | High | Low | Low | High |

For peer review only

| | | | | | | | |
|---|---|---|---|---|---|---|---|
| Malia, 2019 | High | High | High | High | High | Unclear | Low |
| Mandeville, 2011 | High | Unclear | Low | High | Low | Low | Low |
| Miguez, 2015 | Low | High | High | High | Low | High | High |
| Mohammed, 2004 | Low | Unclear | Unclear | High | Low | Low | Unclear |
| Naqvi, 2019 | High | High | Unclear | High | Unclear | High | Low |
| Oikonomopoulou, 2019 | Low | High | High | High | Low | High | Low |
| Ovrebo, 1993 | Unclear | High | High | High | Low | Low | High |
| Ozguner, 2014 | High | High | High | High | Low | High | High |
| Peltola, 1986 | Low | Unclear | Unclear | High | Low | Low | Unclear |
| Prada-Arias, 2017 | Low | Unclear | Unclear | High | Low | Low | Low |
| Rodriguez, 2014 | High | Unclear | Low | High | Low | Low | Low |
| Salo, 2016 | High | Unclear | Unclear | High | Low | Low | Unclear |
| Samuel, 2002 | Low | Unclear | Unclear | High | Low | Low | Unclear |
| Sanchez, 1998 | Low | High | Unclear | High | Unclear | Unclear | Unclear |
| Shommu, 2018 | High | High | High | High | High | Unclear | High |
| Thompson, 2015 | Low | Unclear | High | High | Low | Unclear | High |
| Thompson, 2017 | Unclear | Unclear | High | High | Low | Unclear | High |
| Tuncer, 2019 | High | High | High | High | Low | Unclear | Unclear |
| Turkyilmaz, 2006 | Low | Unclear | Unclear | High | Low | Low | Unclear |
| Van Den Broek, 2004 | Unclear | Unclear | High | High | Low | Low | High |
| Wang, 2007 | Low | Unclear | Unclear | High | Low | Low | Unclear |
| Wu, 2012 | Unclear | High | Unclear | High | Low | High | Low |
| Yap, 2015 | Low | Unclear | Unclear | High | Low | Low | Unclear |
| Yazici, 2010 | High | Unclear | High | High | Low | Unclear | High |
| Zouari, 2016 | Low | Unclear | Unclear | High | Low | Unclear | Low |
| Zuniga, 2012 | High | Unclear | High | High | Low | Low | High |

For peer review only

**Supplementary Table 5:** Pooled estimates of diagnostic accuracy for blood tests used for diagnosis of appendicitis in children calculated using the bivariate model and reporting likelihood ratios(*Median prevalence of appendicitis in the included 54 cohort studies is 40%. Sensitivity analysis was performed for all tests at all cut offs, except procalcitonin as number of studies was too low, and identified significant (p<0.05) effects in: †WCC 10,000-15,000cells/ul for case-control study design (p=0·01, sensitivity 0·70[0·57-0·84] and specificity 0·49[0·31-0·66] n=3 case-control studies vs sensitivity 0·76[0·72-0·80] and specificity 0·72[0·68-0·76] n=27 non case-control studies))

| Marker | Threshold | Studies | Number of patients | Pooled sensitivity (95% CI) | Pooled specificity (95% CI) | Pooled LR+ (95% CI) | Pooled LR- (95% CI) | Hypothetical cohort of 1000 children with appendicitis prevalence of 40%* | |
|---|---|---|---|---|---|---|---|---|---|
| | | | | | | | | Missed appendicitis (FN) | Unnecessary treatment (FP) |
| CRP (mg/L) | ≤5 | 14 | 3754 | 0·82 (0·72 – 0·89) | 0·46 (0·28 – 0·66) | 1·52 (1·14 – 2·03) | 0·39 (0·30 – 0·51) | 112 | 324 |
| | 5-10 | 16 | 3170 | 0·73 (0·67 – 0·80) | 0·67 (0·58 – 0·75) | 2·21 (1·75 – 2·79) | 0·40 (0·33 – 0·50) | 108 | 198 |
| | 10-50 | 19 | 4470 | 0·53 (0·45 – 0·62) | 0·79 (0·74 – 0·83) | 2·53 (1·99 – 3·21) | 0·59 (0·49 – 0·71) | 188 | 126 |
| | ≥50 | 10 | 2654 | 0·35 (0·25 – 0·46) | 0·89 (0·87 – 0·91) | 3·25 (2·63 – 4·01) | 0·73 (0·63 – 0·84) | 260 | 66 |
| WCC (Cells/µl) | ≤10,000 | 28 | 11791 | 0·87 (0·83 – 0·89) | 0·58 (0·53 – 0·63) | 2·08 (1·87 – 2·31) | 0·23 (0·19 – 0·27) | 52 | 252 |
| | 10,000 - 15,000† | 30 | 9967 | 0·76 (0·72 – 0·79) | 0·70 (0·65 – 0·75) | 2·56 (2·19 – 3·00) | 0·35 (0·30 – 0·40) | 96 | 180 |
| | ≥15,000 | 14 | 6406 | 0·50 (0·44 – 0·55) | 0·84 (0·80 – 0·87) | 3·12 (2·74 – 3·55) | 0·60 (0·55 – 0·66) | 200 | 96 |
| Neutrophils (% of lymphocytes) | 65% - 80% | 13 | 3316 | 0·81 (0·74 – 0·86) | 0·64 (0·55 – 0·72) | 2·23 (1·76 – 2·82) | 0·30 (0·22 – 0·41) | 76 | 216 |
| | 75% | 9 | 1642 | 0·78 (0·73-0·82) | 0·61 (0·53-0·69) | 2·02 (1·61-2·53) | 0·36 (0·28-0·46) | 88 | 234 |
| ANC (Cells/µl) | 6600 - 10000 | 22 | 11966 | 0·85 (0·81 – 0·88) | 0·63 (0·58 – 0·68) | 2·29 (2·06 – 2·56) | 0·23 (0·19 – 0·28) | 60 | 222 |
| | <7500 | 5 | 3834 | 0·90 (0·85 – 0·94) | 0·54 (0·49 – 0·58) | 1·95 (1·79 – 2·13) | 0·18 (0·12 – 0·27) | 40 | 276 |
| | 7500 | 11 | 4730 | 0·86 (0·82 – 0·90) | 0·60 (0·54 – 0·65) | 2·14 (1·90 – 2·42) | 0·23 (0·17 – 0·30) | 56 | 240 |
| | >7500 | 5 | 3388 | 0·73 (0·70 – 0·77) | 0·77 (0·70 – 0·82) | 3·13 (2·35 – 4·16) | 0·35 (0·29 – 0·41) | 108 | 138 |
| Procalcitonin (ng/ml) | 0·1 – 0·5 | 5 | 540 | 0·31 (0·25 – 0·37) | 0·82 (0·70 – 0·90) | 1·72 (0·99 – 3·00) | 0·84 (0·74 – 0·96) | 276 | 108 |
| WCC and CRP‡ | | 13 | 4887 | 0·68 (0·52 – 0·81) | 0·78 (0·65 – 0·88) | 3·17 (2·04 – 4·93) | 0·41 (0·27 – 0·60) | 128 | 132 |
| WCC, CRP and Neutrophils | | 6 | 1784 | 0·80 (0·35 – 0·97) | 0·79 (0·60 – 0·90) | 3·78 (2·44 – 5·85) | 0·26 (0·06 – 1·09) | 80 | 126 |



For peer review only - http://bmjopen.bmj.com/site/about/guidelines.xhtml

**Supplementary Figures and legends:**

**Supplementary Figure 1:** Funnel plots outputs for testing individual blood tests for publication bias(68). (Procalcitonin/ WCC and CRP/ WCC,ANC and CRP not shown as study numbers were low [n=4, p=0·2]. Testing was also performed for individual cut-offs of WCC and CRP with all results >0·1 [data not shown]. Y axis = inverse root of effective sample size. X axis = Odds ratio. 95% Confidence intervals for bias: CRP -6.10 – 15.03; WCC -5.26 – 7.15; Neutrophils % -13.72 – 17.74; ANC -4.18 – 11.87)

## WCC

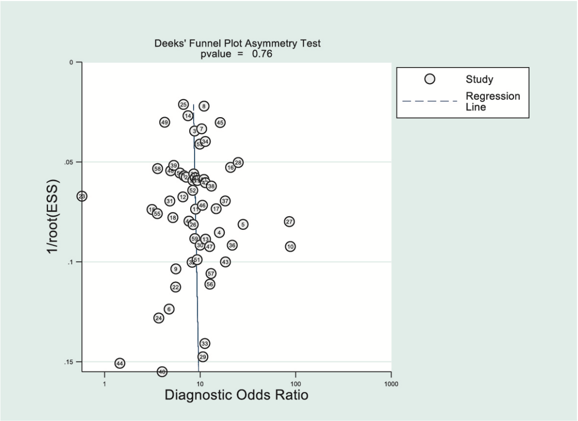


## CRP

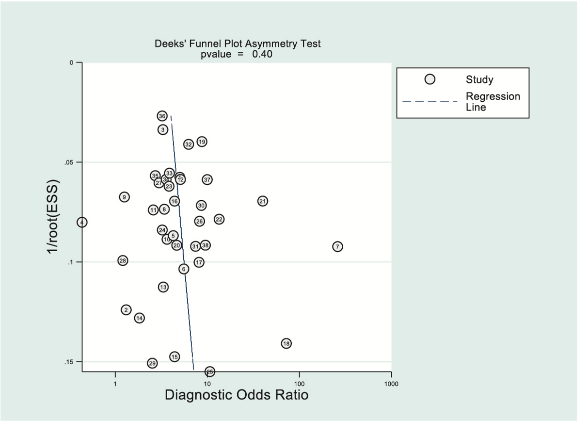


## Neutrophils (%)

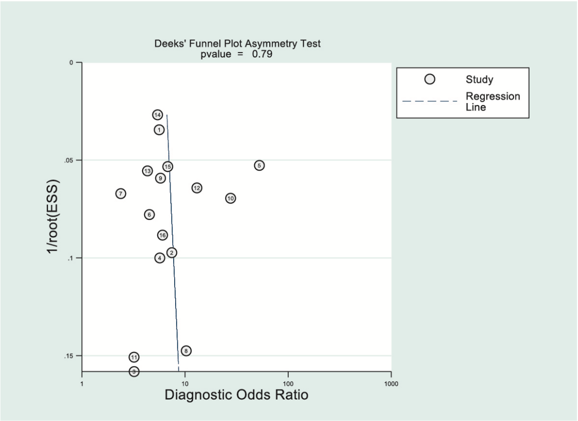


## Absolute neutrophil count

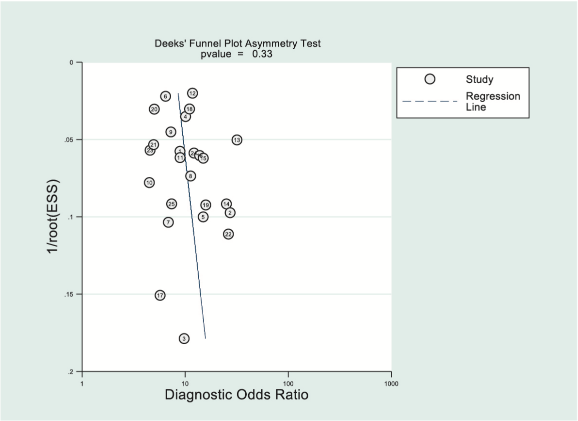




For peer review only - http://bmjopen.bmj.com/site/about/guidelines.xhtml

**Supplementary Figure 2:** Forest plot showing all studies reporting diagnostic accuracy of WCC, split by reported cut off, in paediatric appendicitis. (Sensitivity and specificity for each study represented visually (blue square) with 95% confidence interval (line)).

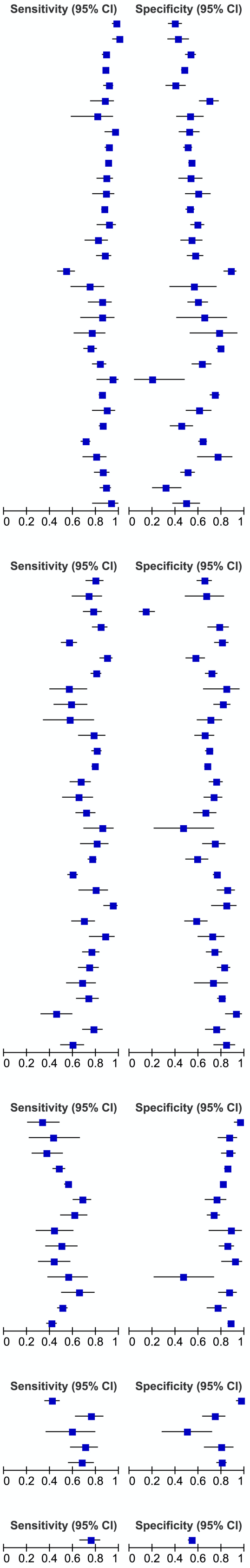

**WCC at or below 10,000 /mm^3**

| Study | TP | FP | FN | TN | Threshold | Sensitivity (95% CI) | Specificity (95% CI) |
|---|---|---|---|---|---|---|---|
| Kharbanda 2005 | 153 | 162 | 4 | 106 | 8.85 | 0.97 [0.94, 0.99] | 0.40 [0.34, 0.46] |
| Kharbanda 2012 | 58 | 67 | 0 | 49 | 8.85 | 1.00 [0.94, 1.00] | 0.42 [0.33, 0.52] |
| Anadalwar 2015 | 348 | 212 | 45 | 240 | 9.0 | 0.89 [0.85, 0.92] | 0.53 [0.48, 0.58] |
| Kashtan 2020 | 857 | 681 | 117 | 622 | 9.0 | 0.88 [0.86, 0.90] | 0.48 [0.45, 0.50] |
| Akgul 2021 | 173 | 78 | 17 | 52 | 10.0 | 0.91 [0.86, 0.95] | 0.40 [0.32, 0.49] |
| Andersson 1999 | 42 | 37 | 6 | 84 | 10.0 | 0.88 [0.75, 0.95] | 0.69 [0.60, 0.77] |
| Andersson 2008 | 17 | 35 | 4 | 39 | 10.0 | 0.81 [0.58, 0.95] | 0.53 [0.41, 0.64] |
| Andersson 2014 | 52 | 61 | 2 | 66 | 10.0 | 0.96 [0.87, 1.00] | 0.52 [0.43, 0.61] |
| Andersson 2017 | 303 | 339 | 30 | 348 | 10.0 | 0.91 [0.87, 0.94] | 0.51 [0.47, 0.54] |
| Bachur 2016 | 786 | 581 | 84 | 682 | 10.0 | 0.90 [0.88, 0.92] | 0.54 [0.51, 0.57] |
| Benito 2016 | 79 | 45 | 10 | 51 | 10.0 | 0.89 [0.80, 0.94] | 0.53 [0.43, 0.63] |
| Cayrol 2016 | 46 | 33 | 6 | 49 | 10.0 | 0.88 [0.77, 0.96] | 0.60 [0.48, 0.70] |
| Chiang 2020 | 625 | 319 | 93 | 354 | 10.0 | 0.87 [0.84, 0.89] | 0.53 [0.49, 0.56] |
| Esparaz 2019 | 51 | 111 | 5 | 160 | 10.0 | 0.91 [0.80, 0.97] | 0.59 [0.53, 0.65] |
| Fleischman 2013 | 53 | 52 | 12 | 61 | 10.0 | 0.82 [0.70, 0.90] | 0.54 [0.44, 0.63] |
| Goldman 2008 | 98 | 84 | 14 | 112 | 10.0 | 0.88 [0.80, 0.93] | 0.57 [0.50, 0.64] |
| Gonzalez Del Castillo 2016 | 93 | 18 | 79 | 131 | 10.0 | 0.54 [0.46, 0.62] | 0.88 [0.82, 0.93] |
| Kaiser 2018 | 29 | 11 | 10 | 14 | 10.0 | 0.74 [0.58, 0.87] | 0.56 [0.35, 0.76] |
| Khanafer 2016 | 46 | 51 | 8 | 75 | 10.0 | 0.85 [0.73, 0.93] | 0.60 [0.50, 0.68] |
| Ko 1995 | 23 | 7 | 4 | 13 | 10.0 | 0.85 [0.66, 0.96] | 0.65 [0.41, 0.85] |
| Lycopoulou 2005 | 32 | 4 | 10 | 14 | 10.0 | 0.76 [0.61, 0.88] | 0.78 [0.52, 0.94] |
| Malia 2019 | 169 | 113 | 56 | 424 | 10.0 | 0.75 [0.69, 0.81] | 0.79 [0.75, 0.82] |
| Mandeville 2011 | 129 | 49 | 26 | 83 | 10.0 | 0.83 [0.76, 0.89] | 0.63 [0.54, 0.71] |
| Ozgunur 2014 | 32 | 12 | 2 | 3 | 10.0 | 0.94 [0.80, 0.99] | 0.20 [0.04, 0.48] |
| Samuel 2002 | 624 | 113 | 110 | 323 | 10.0 | 0.85 [0.82, 0.88] | 0.74 [0.70, 0.78] |
| Shommu 2018 | 41 | 32 | 5 | 49 | 10.0 | 0.89 [0.76, 0.96] | 0.60 [0.49, 0.71] |
| Thompson 2015 | 425 | 56 | 71 | 46 | 10.0 | 0.86 [0.82, 0.89] | 0.45 [0.35, 0.55] |
| Thompson 2017 | 334 | 243 | 137 | 424 | 10.0 | 0.71 [0.67, 0.75] | 0.64 [0.60, 0.67] |
| van den Broek 2004 | 52 | 8 | 13 | 26 | 10.0 | 0.80 [0.68, 0.89] | 0.76 [0.59, 0.89] |
| Yap 2015 | 102 | 126 | 17 | 129 | 10.0 | 0.86 [0.78, 0.91] | 0.51 [0.44, 0.57] |
| Yazici 2010 | 162 | 39 | 21 | 18 | 10.0 | 0.89 [0.83, 0.93] | 0.32 [0.20, 0.45] |
| Zuniga 2012 | 26 | 37 | 2 | 36 | 10.0 | 0.93 [0.76, 0.99] | 0.49 [0.37, 0.61] |

**WCC above 10,000 and below 15,000 /mm^3**

| Study | TP | FP | FN | TN | Threshold | Sensitivity (95% CI) | Specificity (95% CI) |
|---|---|---|---|---|---|---|---|
| Altali 2017 | 92 | 75 | 24 | 140 | 10.5 | 0.79 [0.71, 0.86] | 0.65 [0.58, 0.71] |
| Groselj-Grenc 2007 | 36 | 11 | 13 | 22 | 11.0 | 0.73 [0.59, 0.85] | 0.67 [0.48, 0.82] |
| Hsiao 2005 | 86 | 95 | 25 | 16 | 11.0 | 0.77 [0.69, 0.85] | 0.14 [0.08, 0.22] |
| Mohammed 2004 | 109 | 19 | 21 | 67 | 11.0 | 0.84 [0.76, 0.90] | 0.78 [0.68, 0.86] |
| Ovrebo 1993 | 116 | 34 | 89 | 138 | 11.0 | 0.57 [0.50, 0.63] | 0.80 [0.73, 0.86] |
| Prada Arias 2017 | 118 | 61 | 14 | 82 | 11.0 | 0.89 [0.83, 0.94] | 0.57 [0.49, 0.66] |
| Wu 2012 | 245 | 83 | 61 | 205 | 11.0 | 0.80 [0.75, 0.84] | 0.71 [0.66, 0.76] |
| Kaiser 2018 | 22 | 4 | 17 | 21 | 11.6 | 0.56 [0.40, 0.72] | 0.84 [0.64, 0.95] |
| Andersson 1999 | 28 | 23 | 20 | 98 | 12.0 | 0.58 [0.43, 0.72] | 0.81 [0.73, 0.88] |
| Andersson 2008 | 12 | 22 | 9 | 52 | 12.0 | 0.57 [0.34, 0.78] | 0.70 [0.59, 0.80] |
| Andersson 2014 | 42 | 44 | 12 | 83 | 12.0 | 0.78 [0.64, 0.88] | 0.65 [0.56, 0.74] |
| Andersson 2017 | 268 | 211 | 65 | 476 | 12.0 | 0.80 [0.76, 0.85] | 0.69 [0.66, 0.73] |
| Bachur 2016 | 686 | 409 | 184 | 854 | 12.0 | 0.79 [0.76, 0.82] | 0.68 [0.65, 0.70] |
| Gonzalez Del Castillo 2016 | 74 | 52 | 37 | 158 | 12.0 | 0.67 [0.57, 0.75] | 0.75 [0.69, 0.81] |
| Khanafer 2016 | 35 | 34 | 19 | 92 | 12.0 | 0.65 [0.51, 0.77] | 0.73 [0.64, 0.81] |
| Kwan 2010 | 82 | 32 | 33 | 62 | 12.0 | 0.71 [0.62, 0.79] | 0.66 [0.55, 0.75] |
| Ozgunur 2014 | 29 | 8 | 5 | 7 | 12.0 | 0.85 [0.69, 0.95] | 0.47 [0.21, 0.73] |
| Shommu 2018 | 37 | 21 | 9 | 60 | 12.0 | 0.80 [0.66, 0.91] | 0.74 [0.63, 0.83] |
| Thompson 2015 | 380 | 42 | 116 | 60 | 12.0 | 0.77 [0.73, 0.80] | 0.59 [0.49, 0.68] |
| Thompson 2017 | 281 | 161 | 190 | 506 | 12.0 | 0.60 [0.55, 0.64] | 0.76 [0.72, 0.79] |
| Miguez 2016 | 35 | 14 | 9 | 78 | 12.3 | 0.80 [0.65, 0.90] | 0.85 [0.76, 0.91] |
| Beltran 2007 | 67 | 8 | 4 | 42 | 12.5 | 0.94 [0.86, 0.98] | 0.84 [0.71, 0.93] |
| Diaz Moreno 2012 | 59 | 42 | 26 | 58 | 12.8 | 0.69 [0.58, 0.79] | 0.58 [0.48, 0.68] |
| Rodriguez Sanchez 2013 | 36 | 18 | 5 | 46 | 13.0 | 0.88 [0.74, 0.96] | 0.72 [0.59, 0.82] |
| Tuncer 2019 | 104 | 43 | 33 | 121 | 13.05 | 0.76 [0.68, 0.83] | 0.74 [0.66, 0.80] |
| Oikonomopoulou 2018 | 74 | 33 | 26 | 152 | 13.3 | 0.74 [0.64, 0.82] | 0.82 [0.76, 0.87] |
| Bal 2016 | 38 | 11 | 18 | 29 | 13.4 | 0.68 [0.54, 0.80] | 0.72 [0.56, 0.85] |
| Greer 2019 | 63 | 92 | 23 | 368 | 13.5 | 0.73 [0.63, 0.82] | 0.80 [0.76, 0.84] |
| Kumar 2021 | 25 | 5 | 30 | 60 | 14.0 | 0.45 [0.32, 0.59] | 0.92 [0.83, 0.97] |
| Sanchez-Echaniz 1997 | 73 | 25 | 21 | 76 | 14.0 | 0.78 [0.68, 0.86] | 0.75 [0.66, 0.83] |
| Peltola 1986 | 56 | 11 | 38 | 57 | 14.9 | 0.60 [0.49, 0.70] | 0.84 [0.73, 0.92] |

**WCC at or above 15,000 /mm^3**

| Study | TP | FP | FN | TN | Threshold | Sensitivity (95% CI) | Specificity (95% CI) |
|---|---|---|---|---|---|---|---|
| Andersson 1999 | 16 | 5 | 32 | 116 | 15.0 | 0.33 [0.20, 0.48] | 0.96 [0.91, 0.99] |
| Andersson 2008 | 9 | 10 | 12 | 64 | 15.0 | 0.43 [0.22, 0.66] | 0.86 [0.77, 0.93] |
| Andersson 2014 | 20 | 17 | 34 | 110 | 15.0 | 0.37 [0.24, 0.51] | 0.87 [0.79, 0.92] |
| Andersson 2017 | 159 | 103 | 174 | 584 | 15.0 | 0.48 [0.42, 0.53] | 0.85 [0.82, 0.88] |
| Bachur 2016 | 485 | 240 | 385 | 1023 | 15.0 | 0.56 [0.52, 0.59] | 0.81 [0.79, 0.83] |
| Calvo Rigual 1998 | 96 | 22 | 45 | 68 | 15.0 | 0.68 [0.60, 0.76] | 0.76 [0.65, 0.84] |
| Gonzalez Del Castillo 2016 | 44 | 67 | 28 | 182 | 15.0 | 0.61 [0.49, 0.72] | 0.73 [0.67, 0.78] |
| Kaiser 2018 | 17 | 3 | 22 | 22 | 15.0 | 0.44 [0.28, 0.60] | 0.88 [0.69, 0.97] |
| Khanafer 2016 | 27 | 19 | 27 | 107 | 15.0 | 0.50 [0.36, 0.64] | 0.85 [0.77, 0.91] |
| Lin 2009 | 23 | 4 | 30 | 43 | 15.0 | 0.43 [0.30, 0.58] | 0.91 [0.80, 0.98] |
| Ozgunur 2014 | 19 | 8 | 15 | 7 | 15.0 | 0.56 [0.38, 0.73] | 0.47 [0.21, 0.73] |
| Shommu 2018 | 30 | 11 | 16 | 70 | 15.0 | 0.65 [0.50, 0.79] | 0.86 [0.77, 0.93] |
| Thompson 2015 | 252 | 24 | 244 | 78 | 15.0 | 0.51 [0.46, 0.55] | 0.76 [0.67, 0.84] |
| Thompson 2017 | 195 | 82 | 276 | 585 | 15.0 | 0.41 [0.37, 0.46] | 0.88 [0.85, 0.90] |

**WCC threshold depending on age**

| Study | TP | FP | FN | TN | Threshold | Sensitivity (95% CI) | Specificity (95% CI) |
|---|---|---|---|---|---|---|---|
| Doraiswamy 1979 | 94 | 5 | 131 | 145 | | 0.42 [0.35, 0.49] | 0.97 [0.92, 0.99] |
| Klein 2021 | 40 | 21 | 13 | 60 | | 0.75 [0.62, 0.86] | 0.74 [0.63, 0.83] |
| Salo 2016 | 13 | 11 | 9 | 11 | | 0.59 [0.36, 0.79] | 0.50 [0.28, 0.72] |
| Turkyilmaz 2006 | 43 | 9 | 18 | 35 | | 0.70 [0.57, 0.81] | 0.80 [0.65, 0.90] |
| Wang 2007 | 50 | 67 | 24 | 269 | | 0.68 [0.56, 0.78] | 0.80 [0.75, 0.84] |

**WCC threshold depending on duration pain**

| Study | TP | FP | FN | TN | Threshold | Sensitivity (95% CI) | Specificity (95% CI) |
|---|---|---|---|---|---|---|---|
| Dadeh 2021 | 73 | 434 | 24 | 512 | | 0.75 [0.65, 0.83] | 0.54 [0.51, 0.57] |

**Supplementary Figure 3:** Forest plot showing all studies reporting diagnostic accuracy of CPR, split by reported cut off, in paediatric appendicitis. (Sensitivity and specificity for each study represented visually (blue square) with 95% confidence interval (line)).

**CRP at or below 5 mg/l**

| Study | TP | FP | FN | TN | Threshold | Sensitivity (95% CI) | Specificity (95% CI) |
|---|---|---|---|---|---|---|---|
| Bal 2016 | 50 | 24 | 6 | 16 | 0.9 | 0.89 [0.78, 0.96] | 0.40 [0.25, 0.57] |
| Miguez 2016 | 30 | 29 | 14 | 63 | 3.0 | 0.68 [0.52, 0.81] | 0.68 [0.58, 0.78] |
| Lycopoulou 2005 | 34 | 1 | 8 | 17 | 4.5 | 0.81 [0.66, 0.91] | 0.94 [0.73, 1.00] |
| Akgul 2021 | 146 | 72 | 44 | 59 | 5.0 | 0.77 [0.70, 0.83] | 0.45 [0.36, 0.54] |
| Altali 2017 | 92 | 93 | 24 | 122 | 5.0 | 0.79 [0.71, 0.86] | 0.57 [0.50, 0.63] |
| Andersson 1999 | 29 | 34 | 17 | 85 | 5.0 | 0.63 [0.48, 0.77] | 0.71 [0.62, 0.79] |
| Andersson 2008 | 17 | 55 | 4 | 17 | 5.0 | 0.81 [0.58, 0.95] | 0.24 [0.14, 0.35] |
| Andersson 2014 | 55 | 127 | 0 | 0 | 5.0 | 1.00 [0.94, 1.00] | 0.00 [0.00, 0.03] |
| Andersson 2017 | 285 | 481 | 38 | 211 | 5.0 | 0.88 [0.84, 0.92] | 0.30 [0.27, 0.34] |
| Chiang 2020 | 589 | 394 | 129 | 279 | 5.0 | 0.82 [0.79, 0.85] | 0.41 [0.38, 0.45] |
| Gonzalez Del Castillo 2016 | 88 | 90 | 23 | 120 | 5.0 | 0.79 [0.71, 0.86] | 0.57 [0.50, 0.64] |
| Greer 2019 | 69 | 133 | 17 | 327 | 5.0 | 0.80 [0.70, 0.88] | 0.71 [0.67, 0.75] |
| Kaiser 2018 | 23 | 11 | 16 | 14 | 5.0 | 0.59 [0.42, 0.74] | 0.56 [0.35, 0.76] |
| Malia 2019 | 160 | 118 | 65 | 419 | 5.0 | 0.71 [0.65, 0.77] | 0.78 [0.74, 0.81] |
| Ozgunur 2014 | 32 | 9 | 2 | 6 | 5.0 | 0.94 [0.80, 0.99] | 0.40 [0.16, 0.68] |
| Rodriguez Sanchez 2013 | 21 | 29 | 21 | 35 | 5.0 | 0.50 [0.34, 0.66] | 0.55 [0.42, 0.67] |
| Yap 2015 | 99 | 143 | 20 | 112 | 5.0 | 0.83 [0.75, 0.89] | 0.44 [0.38, 0.50] |

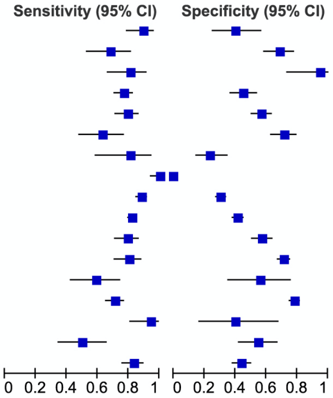


**CRP between 5 and 11 mg/l**

| Study | TP | FP | FN | TN | Threshold | Sensitivity (95% CI) | Specificity (95% CI) |
|---|---|---|---|---|---|---|---|
| Zouari 2016 | 107 | 74 | 32 | 79 | 6.0 | 0.77 [0.69, 0.84] | 0.52 [0.43, 0.60] |
| Naqvi 2019 | 64 | 20 | 15 | 63 | 7.9 | 0.81 [0.71, 0.89] | 0.76 [0.65, 0.85] |
| Mohammed 2004 | 114 | 13 | 16 | 73 | 8.0 | 0.88 [0.81, 0.93] | 0.85 [0.76, 0.92] |
| Groselj-Grenc 2007 | 36 | 15 | 13 | 18 | 9.0 | 0.73 [0.59, 0.85] | 0.55 [0.36, 0.72] |
| Ko 1995 | 19 | 7 | 8 | 13 | 9.0 | 0.70 [0.50, 0.86] | 0.65 [0.41, 0.85] |
| Andersson 1999 | 23 | 26 | 23 | 93 | 10.0 | 0.50 [0.35, 0.65] | 0.78 [0.70, 0.85] |
| Andersson 2008 | 15 | 25 | 6 | 47 | 10.0 | 0.71 [0.48, 0.89] | 0.65 [0.53, 0.76] |
| Andersson 2014 | 43 | 53 | 12 | 74 | 10.0 | 0.78 [0.65, 0.88] | 0.58 [0.49, 0.67] |
| Andersson 2017 | 221 | 249 | 102 | 443 | 10.0 | 0.68 [0.63, 0.73] | 0.64 [0.60, 0.68] |
| Gonzalez Del Castillo 2016 | 75 | 70 | 36 | 140 | 10.0 | 0.68 [0.58, 0.76] | 0.67 [0.60, 0.73] |
| Kaiser 2018 | 20 | 10 | 19 | 15 | 10.0 | 0.51 [0.35, 0.68] | 0.60 [0.39, 0.79] |
| Lin 2009 | 39 | 12 | 14 | 35 | 10.0 | 0.74 [0.60, 0.85] | 0.74 [0.60, 0.86] |
| Lycopoulou 2005 | 26 | 1 | 16 | 17 | 10.0 | 0.62 [0.46, 0.76] | 0.94 [0.73, 1.00] |
| Ovrebo 1993 | 48 | 80 | 5 | 27 | 10.0 | 0.91 [0.79, 0.97] | 0.25 [0.17, 0.35] |
| Ozgunur 2014 | 25 | 7 | 9 | 8 | 10.0 | 0.74 [0.56, 0.87] | 0.53 [0.27, 0.79] |
| Peltola 1986 | 60 | 12 | 34 | 56 | 10.0 | 0.64 [0.53, 0.73] | 0.82 [0.71, 0.91] |
| Kumar 2021 | 49 | 30 | 6 | 35 | 10.5 | 0.89 [0.78, 0.96] | 0.54 [0.41, 0.66] |

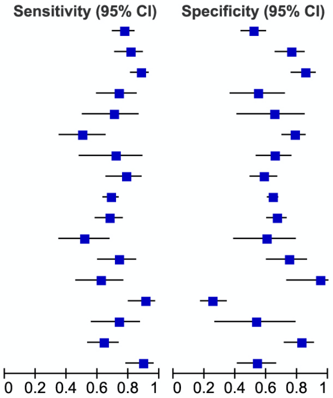


**CRP between 11 and 50 mg/l**

| Study | TP | FP | FN | TN | Threshold | Sensitivity (95% CI) | Specificity (95% CI) |
|---|---|---|---|---|---|---|---|
| Oikonomopoulou 2018 | 60 | 52 | 40 | 133 | 12.5 | 0.60 [0.50, 0.70] | 0.72 [0.65, 0.78] |
| Salo 2016 | 14 | 9 | 8 | 13 | 15.0 | 0.64 [0.41, 0.83] | 0.59 [0.36, 0.79] |
| Ozgunur 2014 | 24 | 5 | 10 | 10 | 16.0 | 0.71 [0.53, 0.85] | 0.67 [0.38, 0.88] |
| Diaz Moreno 2012 | 46 | 31 | 39 | 68 | 18.0 | 0.54 [0.43, 0.65] | 0.69 [0.59, 0.78] |
| Benito 2016 | 31 | 13 | 58 | 83 | 20.0 | 0.35 [0.25, 0.46] | 0.86 [0.78, 0.93] |
| Calvo Rigual 1998 | 61 | 34 | 80 | 56 | 20.0 | 0.43 [0.35, 0.52] | 0.62 [0.51, 0.72] |
| Shommu 2018 | 24 | 9 | 24 | 67 | 20.0 | 0.50 [0.35, 0.65] | 0.88 [0.79, 0.94] |
| Wu 2012 | 214 | 78 | 92 | 210 | 25.0 | 0.70 [0.64, 0.75] | 0.73 [0.67, 0.78] |
| Prada Arias 2017 | 57 | 29 | 75 | 114 | 25.2 | 0.43 [0.35, 0.52] | 0.80 [0.72, 0.86] |
| Andersson 1999 | 14 | 14 | 32 | 105 | 30.0 | 0.30 [0.18, 0.46] | 0.88 [0.81, 0.93] |
| Andersson 2008 | 8 | 11 | 13 | 61 | 30.0 | 0.38 [0.18, 0.62] | 0.85 [0.74, 0.92] |
| Andersson 2014 | 31 | 35 | 24 | 92 | 30.0 | 0.56 [0.42, 0.70] | 0.72 [0.64, 0.80] |
| Andersson 2017 | 140 | 128 | 183 | 564 | 30.0 | 0.43 [0.38, 0.49] | 0.82 [0.78, 0.84] |
| Cayrol 2016 | 20 | 12 | 32 | 70 | 30.0 | 0.38 [0.25, 0.53] | 0.85 [0.76, 0.92] |
| Gonzalez Del Castillo 2016 | 59 | 40 | 52 | 170 | 30.0 | 0.53 [0.43, 0.63] | 0.81 [0.75, 0.86] |
| Kaiser 2018 | 13 | 8 | 26 | 17 | 30.0 | 0.33 [0.19, 0.50] | 0.68 [0.46, 0.85] |
| Kwan 2010 | 81 | 33 | 34 | 61 | 30.0 | 0.70 [0.61, 0.79] | 0.65 [0.54, 0.74] |
| Sanchez-Echaniz 1997 | 40 | 8 | 54 | 93 | 30.0 | 0.43 [0.32, 0.53] | 0.92 [0.85, 0.97] |
| Beltran 2007 | 68 | 4 | 3 | 46 | 47.0 | 0.96 [0.88, 0.99] | 0.92 [0.81, 0.98] |

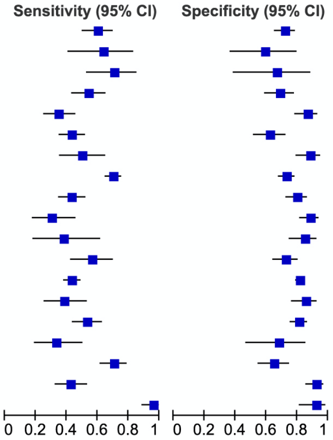


**CRP at or above 50 mg/l**

| Study | TP | FP | FN | TN | Threshold | Sensitivity (95% CI) | Specificity (95% CI) |
|---|---|---|---|---|---|---|---|
| Andersson 1999 | 7 | 10 | 39 | 109 | 50.0 | 0.15 [0.06, 0.29] | 0.92 [0.85, 0.96] |
| Andersson 2008 | 5 | 7 | 16 | 65 | 50.0 | 0.24 [0.08, 0.47] | 0.90 [0.81, 0.96] |
| Andersson 2014 | 20 | 21 | 35 | 106 | 50.0 | 0.36 [0.24, 0.50] | 0.83 [0.76, 0.89] |
| Andersson 2017 | 93 | 62 | 230 | 630 | 50.0 | 0.29 [0.24, 0.34] | 0.91 [0.89, 0.93] |
| Gonzalez Del Castillo 2016 | 37 | 28 | 74 | 182 | 50.0 | 0.33 [0.25, 0.43] | 0.87 [0.81, 0.91] |
| Kaiser 2018 | 9 | 2 | 30 | 23 | 50.0 | 0.23 [0.11, 0.39] | 0.92 [0.74, 0.99] |
| Ko 1995 | 14 | 1 | 13 | 19 | 50.0 | 0.52 [0.32, 0.71] | 0.95 [0.75, 1.00] |
| Ozgunur 2014 | 15 | 3 | 19 | 12 | 50.0 | 0.44 [0.27, 0.62] | 0.80 [0.52, 0.96] |
| Shommu 2018 | 12 | 1 | 36 | 75 | 50.0 | 0.25 [0.14, 0.40] | 0.99 [0.93, 1.00] |
| Wu 2012 | 214 | 49 | 92 | 239 | 89.0 | 0.70 [0.64, 0.75] | 0.83 [0.78, 0.87] |

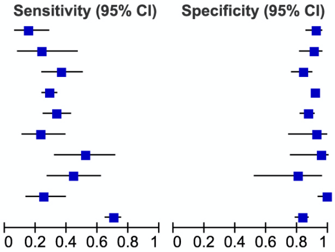




For peer review only - http://bmjopen.bmj.com/site/about/guidelines.xhtml

**Supplementary Figure 4:** Forest plot showing all studies reporting diagnostic accuracy of ANC in paediatric appendicitis. (Sensitivity and specificity for each study represented visually (blue square) with 95% confidence interval (line)).

| Study | TP | FP | FN | TN | Threshold | Sensitivity (95% CI) | Specificity (95% CI) |
|---|---|---|---|---|---|---|---|
| Salo 2016 | 16 | 7 | 6 | 15 | age dependent | 0.73 [0.50, 0.89] | 0.68 [0.45, 0.86] |
| Altali 2017 | 99 | 85 | 17 | 130 | 6.6 | 0.85 [0.78, 0.91] | 0.60 [0.54, 0.67] |
| Kharbanda 2012 | 942 | 824 | 76 | 783 | 6.73 | 0.93 [0.91, 0.94] | 0.49 [0.46, 0.51] |
| Fleischman 2013 | 53 | 56 | 12 | 57 | 6.75 | 0.82 [0.70, 0.90] | 0.50 [0.41, 0.60] |
| Kharbanda 2005 | 152 | 131 | 5 | 137 | 6.75 | 0.97 [0.93, 0.99] | 0.51 [0.45, 0.57] |
| Prada Arias 2017 | 119 | 57 | 13 | 86 | 7.0 | 0.90 [0.84, 0.95] | 0.60 [0.52, 0.68] |
| Akgul 2021 | 163 | 74 | 27 | 56 | 7.5 | 0.86 [0.80, 0.90] | 0.43 [0.34, 0.52] |
| Andersson 1999 | 34 | 38 | 2 | 61 | 7.5 | 0.94 [0.81, 0.99] | 0.62 [0.51, 0.71] |
| Andersson 2008 | 8 | 27 | 1 | 33 | 7.5 | 0.89 [0.52, 1.00] | 0.55 [0.42, 0.68] |
| Andersson 2014 | 35 | 32 | 3 | 41 | 7.5 | 0.92 [0.79, 0.98] | 0.56 [0.44, 0.68] |
| Andersson 2017 | 274 | 282 | 31 | 322 | 7.5 | 0.90 [0.86, 0.93] | 0.53 [0.49, 0.57] |
| Benito 2016 | 78 | 37 | 11 | 59 | 7.5 | 0.88 [0.79, 0.94] | 0.61 [0.51, 0.71] |
| Goldman 2008 | 84 | 67 | 17 | 121 | 7.5 | 0.83 [0.74, 0.90] | 0.64 [0.57, 0.71] |
| Greer 2019 | 69 | 115 | 17 | 345 | 7.5 | 0.80 [0.70, 0.88] | 0.75 [0.71, 0.79] |
| Samuel 2002 | 595 | 122 | 139 | 314 | 7.5 | 0.81 [0.78, 0.84] | 0.72 [0.68, 0.76] |
| Shommu 2018 | 40 | 24 | 6 | 57 | 7.5 | 0.87 [0.74, 0.95] | 0.70 [0.59, 0.80] |
| Thompson 2015 | 351 | 68 | 47 | 45 | 7.5 | 0.88 [0.85, 0.91] | 0.40 [0.31, 0.49] |
| Thompson 2017 | 319 | 206 | 141 | 457 | 7.5 | 0.69 [0.65, 0.74] | 0.69 [0.65, 0.72] |
| Zuniga 2012 | 27 | 37 | 1 | 36 | 7.5 | 0.96 [0.82, 1.00] | 0.49 [0.37, 0.61] |
| Corkum 2018 | 116 | 166 | 40 | 416 | 8.0 | 0.74 [0.67, 0.81] | 0.71 [0.68, 0.75] |
| Bal 2016 | 43 | 13 | 13 | 27 | 9.4 | 0.77 [0.64, 0.87] | 0.68 [0.51, 0.81] |
| Miguez 2016 | 37 | 16 | 7 | 76 | 9.6 | 0.84 [0.70, 0.93] | 0.83 [0.73, 0.90] |
| Bachur 2016 | 630 | 365 | 240 | 898 | 10.0 | 0.72 [0.69, 0.75] | 0.71 [0.69, 0.74] |
| Kumar 2021 | 28 | 8 | 27 | 57 | 10.48 | 0.51 [0.37, 0.65] | 0.88 [0.77, 0.95] |
| Oikonomopoulou 2018 | 71 | 26 | 29 | 159 | 10.8 | 0.71 [0.61, 0.80] | 0.86 [0.80, 0.91] |

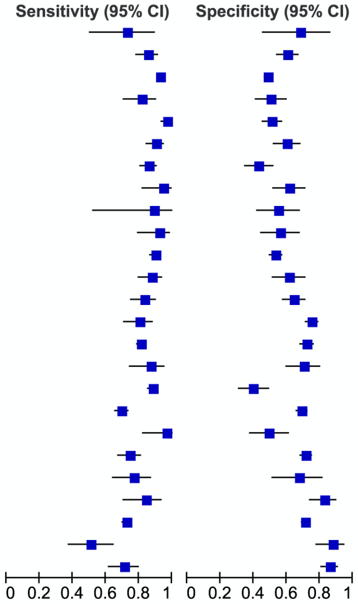


**Supplementary Figure 5:** Forest plot showing all studies reporting diagnostic accuracy of Neutrophil count as a percentage in paediatric appendicitis. (Sensitivity and specificity for each study represented visually (blue square) with 95% confidence interval (line)).

| Study | TP | FP | FN | TN | Threshold | Sensitivity (95% CI) | Specificity (95% CI) |
|---|---|---|---|---|---|---|---|
| Anadalwar 2015 | 340 | 241 | 53 | 211 | 65% | 0.87 [0.83, 0.90] | 0.47 [0.42, 0.51] |
| Salo 2016 | 16 | 10 | 6 | 12 | 71% | 0.73 [0.50, 0.89] | 0.55 [0.32, 0.76] |
| Andersson 1999 | 31 | 45 | 5 | 54 | 75% | 0.86 [0.71, 0.95] | 0.55 [0.44, 0.65] |
| Andersson 2008 | 9 | 29 | 3 | 31 | 75% | 0.75 [0.43, 0.95] | 0.52 [0.38, 0.65] |
| Andersson 2014 | 30 | 29 | 8 | 44 | 75% | 0.79 [0.63, 0.90] | 0.60 [0.48, 0.72] |
| Chiang 2020 | 524 | 224 | 194 | 449 | 75% | 0.73 [0.70, 0.76] | 0.67 [0.63, 0.70] |
| Dadeh 2021 | 74 | 304 | 23 | 642 | 75% | 0.76 [0.67, 0.84] | 0.68 [0.65, 0.71] |
| Fleischman 2013 | 50 | 48 | 15 | 65 | 75% | 0.77 [0.65, 0.86] | 0.58 [0.48, 0.67] |
| Hsiao 2005 | 87 | 67 | 24 | 44 | 75% | 0.78 [0.70, 0.86] | 0.40 [0.30, 0.49] |
| Klein 2021 | 36 | 21 | 17 | 60 | 75% | 0.68 [0.54, 0.80] | 0.74 [0.63, 0.83] |
| Ko 1995 | 22 | 6 | 5 | 14 | 75% | 0.81 [0.62, 0.94] | 0.70 [0.46, 0.88] |
| Mandeville 2011 | 119 | 48 | 36 | 84 | 75% | 0.77 [0.69, 0.83] | 0.64 [0.55, 0.72] |
| Mohammed 2004 | 111 | 15 | 19 | 71 | 75% | 0.85 [0.78, 0.91] | 0.83 [0.73, 0.90] |
| Yap 2015 | 79 | 80 | 40 | 175 | 75% | 0.66 [0.57, 0.75] | 0.69 [0.63, 0.74] |
| Wang 2007 | 44 | 34 | 30 | 302 | 80% | 0.59 [0.47, 0.71] | 0.90 [0.86, 0.93] |
| Doraiswamy 1979 | 216 | 47 | 9 | 103 | age dependent | 0.96 [0.93, 0.98] | 0.69 [0.61, 0.76] |

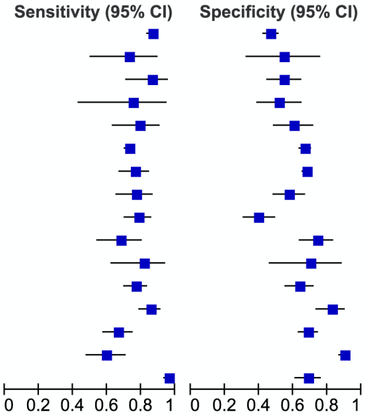


**Supplementary Figure 6:** Forest plot showing all studies reporting diagnostic accuracy of procalcitonin in paediatric appendicitis. (Sensitivity and specificity for each study represented visually (blue square) with 95% confidence interval (line)).

| Study | TP | FP | FN | TN | Threshold | Sensitivity (95% CI) | Specificity (95% CI) |
|---|---|---|---|---|---|---|---|
| Benito 2016 | 34 | 34 | 55 | 62 | 0.1 | 0.38 [0.28, 0.49] | 0.65 [0.54, 0.74] |
| Bal 2016 | 18 | 4 | 38 | 36 | 0.37 | 0.32 [0.20, 0.46] | 0.90 [0.76, 0.97] |
| Khan 2012 | 6 | 2 | 16 | 26 | 0.39 | 0.27 [0.11, 0.50] | 0.93 [0.76, 0.99] |
| Rodriguez Sanchez 2013 | 10 | 18 | 34 | 46 | 0.5 | 0.23 [0.11, 0.38] | 0.72 [0.59, 0.82] |
| Kouame 2005 | 19 | 4 | 49 | 29 | 0.5 | 0.28 [0.18, 0.40] | 0.88 [0.72, 0.97] |
| Akgul 2021 | 55 | 19 | 135 | 111 | 0.5 | 0.29 [0.23, 0.36] | 0.85 [0.78, 0.91] |

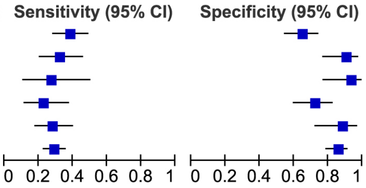




For peer review only - http://bmjopen.bmj.com/site/about/guidelines.xhtml

**Supplementary Figure 7:** Forest plot showing all studies reporting diagnostic accuracy of combination of WCC and/or CRP in paediatric appendicitis. (Sensitivity and specificity for each study represented visually (blue square) with 95% confidence interval (line)).

**CRP and WCC**

| Study | TP | FP | FN | TN | Threshold WCC - CRP | Sensitivity (95% CI) | Specificity (95% CI) |
|---|---|---|---|---|---|---|---|
| Andersson 1999 | 20 | 12 | 26 | 107 | 10.0 - 10 | 0.43 [0.29, 0.59] | 0.90 [0.83, 0.95] |
| Andersson 2008 | 12 | 16 | 9 | 56 | 10.0 - 10 | 0.57 [0.34, 0.78] | 0.78 [0.66, 0.87] |
| Andersson 2014 | 39 | 31 | 14 | 96 | 10.0 - 10 | 0.74 [0.60, 0.85] | 0.76 [0.67, 0.83] |
| Andersson 2017 | 197 | 150 | 122 | 521 | 10.0 - 10 | 0.62 [0.56, 0.67] | 0.78 [0.74, 0.81] |
| Calvo Rigual 1998 | 113 | 45 | 28 | 45 | 15.0 - 20 | 0.80 [0.73, 0.86] | 0.50 [0.39, 0.61] |
| Huckins 2013 | 140 | 238 | 4 | 121 | 10.0 - 8 | 0.97 [0.93, 0.99] | 0.34 [0.29, 0.39] |
| Huckins 2016 | 457 | 775 | 19 | 635 | 10.0 - 8 | 0.96 [0.94, 0.98] | 0.45 [0.42, 0.48] |
| Kaiser 2018 | 16 | 7 | 23 | 18 | 10.0 - 10 | 0.41 [0.26, 0.58] | 0.72 [0.51, 0.88] |
| Kwan 2010 | 48 | 8 | 67 | 86 | 12.0 - 30 | 0.42 [0.33, 0.51] | 0.91 [0.84, 0.96] |
| Ovrebo 1993 | 39 | 0 | 14 | 107 | 11.0- 10 | 0.74 [0.60, 0.85] | 1.00 [0.97, 1.00] |
| Ozgunur 2014 | 24 | 5 | 10 | 10 | 10.0 - 10 | 0.71 [0.53, 0.85] | 0.67 [0.38, 0.88] |
| Peltola 1986 | 42 | 20 | 52 | 48 | 14.9 - 100 | 0.45 [0.34, 0.55] | 0.71 [0.58, 0.81] |
| Sanchez-Echaniz 1997 | 32 | 5 | 62 | 96 | 14.0 - 30 | 0.34 [0.25, 0.45] | 0.95 [0.89, 0.98] |

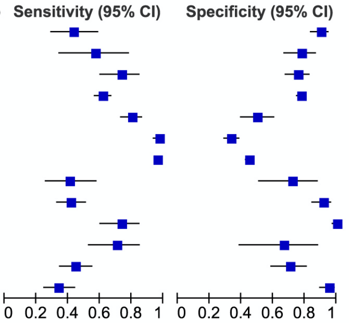


**CRP or WCC**

| Study | TP | FP | FN | TN | Threshold WCC - CRP | Sensitivity (95% CI) | Specificity (95% CI) |
|---|---|---|---|---|---|---|---|
| Chiang 2020 | 716 | 514 | 2 | 159 | 10.0 - 5 | 1.00 [0.99, 1.00] | 0.24 [0.20, 0.27] |

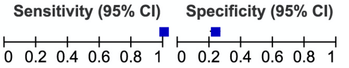


For peer review only

**Supplementary Figure 8:** Forest plot showing all studies reporting diagnostic accuracy of combination of WCC, CRP and NC in paediatric appendicitis. (Sensitivity and specificity for each study represented visually (blue square) with 95% confidence interval (line)).

| Study | TP | FP | FN | TN | Threshold WCC - CRP - NC% | Sensitivity (95% CI) | Specificity (95% CI) |
|---|---|---|---|---|---|---|---|
| Andersson 1999 | 15 | 11 | 20 | 88 | 10.0 - 10 - 75% | 0.43 [0.26, 0.61] | 0.89 [0.81, 0.94] |
| Andersson 2008 | 6 | 11 | 6 | 48 | 10.0 - 10 - 75% | 0.50 [0.21, 0.79] | 0.81 [0.69, 0.90] |
| Andersson 2014 | 21 | 12 | 17 | 61 | 10.0 - 10 - 75% | 0.55 [0.38, 0.71] | 0.84 [0.73, 0.91] |
| Andersson 2017 | 139 | 89 | 152 | 498 | 10.0 - 10 - 75% | 0.48 [0.42, 0.54] | 0.85 [0.82, 0.88] |
| Mohammed 2004 | 112 | 8 | 18 | 78 | 11.0 - 80 - 75% | 0.86 [0.79, 0.92] | 0.91 [0.82, 0.96] |
| Yap 2015 | 119 | 192 | 0 | 63 | 10.0 - 5 - 75% | 1.00 [0.97, 1.00] | 0.25 [0.20, 0.30] |

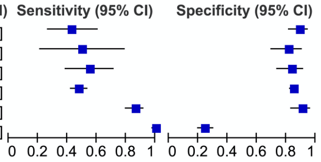



For peer review only - http://bmjopen.bmj.com/site/about/guidelines.xhtml

**Search Strategy:**

Searches were performed on the 15th March 2022 with search terms that had been informed by scoping searches. Databases searched included Cochrane Database of Systematic Reviews, Cochrane Central Register of controlled trials, Database of abstracts of Reviews of Effects, Embase, Ovid(Medline) Epub Ahead of print in process and other Non-Indexed Citations, Ovid Medline(R) Daily, Ovid Medline(R) and Science Citation Index and Conference Proceedings Citation Index (SCIENCE). These databases were searched through the interfaces Cochrane Library(Wiley), OvidSP and Web of Science Core Collection. Specific terms used for these interfaces are detailed below, following searching all results were merged and checked for duplicates before review (**Figure 1**).

**Medline**:

| # | Searches | Results |
|---|---|---|
| 1 | adolescent/ or exp child/ or exp infant/ | 3410061 |
| 2 | (child* or schoolchild* or preschool* or pre-school* or boys or girls or infant? or pediatric* or paediatric* or adolescen* or teen* or youth?).ti,ab. | 1876871 |
| 3 | 1 or 2 | 3876495 |
| 4 | exp Leukocyte Count/ or leukocytes/ or neutrophils/ | 225318 |
| 5 | (leukocyte? or white cell or white blood cell or neutrophil*).ti,ab. | 272998 |
| 6 | (wbcc or wcc).ti,ab. | 655 |
| 7 | C-Reactive Protein/ | 42976 |
| 8 | (c-reactive protein? or crp).ti,ab. | 75114 |
| 9 | Calcitonin/ | 15622 |
| 10 | (procalcitonin or calcitonin or pct).ti,ab. | 32717 |
| 11 | 4 or 5 or 6 or 7 or 8 or 9 or 10 | 490743 |
| 12 | Appendicitis/ | 18470 |
| 13 | Appendectomy/ | 10586 |
| 14 | (appendix or appendicitis or appendectom* or appendicectom*).ti,ab. | 33426 |
| 15 | 12 or 13 or 14 | 38889 |
| 16 | 3 and 11 and 15 | 787 |

**Embase:**

| # | Searches | Results |
|---|---|---|
| 1 | juvenile/ or exp adolescent/ or exp child/ | 3172209 |
| 2 | (child* or schoolchild* or preschool* or pre-school* or boys or girls or infant? or pediatric* or paediatric* or adolescen* or teen* or youth?).ti,ab. | 2298797 |
| 3 | 1 or 2 | 3780916 |
| 4 | exp leukocyte count/ or leukocyte/ or neutrophil/ | 394999 |
| 5 | (leukocyte? or white cell or white blood cell or neutrophil*).ti,ab. | 364068 |
| 6 | (wbcc or wcc).ti,ab. | 1603 |
| 7 | C reactive protein/ | 158251 |
| 8 | (c-reactive protein? or crp).ti,ab. | 129235 |
| 9 | Procalcitonin/ | 10738 |
| 10 | (procalcitonin or calcitonin or pct).ti,ab. | 43836 |


For peer review only - http://bmjopen.bmj.com/site/about/guidelines.xhtml
For peer review only

| | | |
|---|---|---|
| 11 | 4 or 5 or 6 or 7 or 8 or 9 or 10 | 749518 |
| 12 | exp appendicitis/ | 22927 |
| 13 | appendectomy/ | 18892 |
| 14 | (appendix or appendicitis or appendectom* or appendicectom*).ti,ab. | 37059 |
| 15 | 12 or 13 or 14 | 46062 |
| 16 | 3 and 11 and 15 | 1369 |

**Cochrane:**

| # | Searches |
|---|---|
| 1 | MeSH descriptor: [Adolescent] explode all trees |
| 2 | MeSH descriptor: [Child] explode all trees |
| 3 | MeSH descriptor: [Infant] explode all trees |
| 4 | exp leukocyte count/ or leukocyte/ or neutrophil/ |
| 5 | child* or schoolchild* or preschool* or pre-school* or boys or girls or infant? or pediatric* or paediatric* or adolescen* or teen* or youth?:ti,ab,kw (Word variations have been searched) |
| 6 | #1 or #2 or #3 or #4 |
| 7 | MeSH descriptor: [Leukocyte Count] explode all trees |
| 8 | MeSH descriptor: [Leukocytes] this term only |
| 9 | MeSH descriptor: [Neutrophils] explode all trees |
| 10 | wbcc or wcc:ti,ab,kw (Word variations have been searched) |
| 11 | MeSH descriptor: [C-Reactive Protein] explode all trees |
| 12 | c-reactive protein* or crp:ti,ab,kw (Word variations have been searched) |
| 13 | MeSH descriptor: [Calcitonin] explode all trees |
| 14 | procalcitonin or calcitonin or pct:ti,ab,kw |
| 15 | #6 or #7 or #8 or #9 or #10 or #11 or #12 or #13 or #14 |
| 16 | MeSH descriptor: [Appendicitis] explode all trees |
| 17 | MeSH descriptor: [Appendectomy] explode all trees |
| 18 | appendix or appendicitis or appendectom* or appendicectom*:ti,ab,kw (Word variations have been searched) |
| 19 | #16 or #17 or #18 |
| 20 | #5 and #15 and #19 |

**Web of Science:**

| # | Searches | Results |
|---|---|---|
| 1 | TS=(child* or schoolchild* or preschool* or pre-school* or boys or girls or infant? or pediatric* or paediatric* or adolescen* or teen* or youth?) | 1709598 |
| 2 | TS=(leukocyte* or "white cell" or "white blood cell" or neutrophil*) OR TS=("c-reactive protein" OR "c reactive protein" OR crp) OR TS=(procalcitonin OR calcitonin OR pct) | 433876 |
| 3 | TS=(appendix or appendicitis or appendectom* or appendicectom*) | 33245 |
| 4 | #3 AND #2 AND #1 | 397 |


For peer review only - http://bmjopen.bmj.com/site/about/guidelines.xhtml

**QUADAS-2 Scoring criteria**

Below is an overview, with examples of how the QUADAS-2 scoring criteria was used to measure risk of bias and applicability in selected studies(69). In those patients not undergoing surgery the optimal reference standard was follow up after 2 weeks to confirm absence of disease. Studies including only patients who had undergone a radiological investigation were included but this caveat was noted when assessing these studies for risk of bias. Study quality was not an exclusion criteria.

Domain 1: Patient Selection

- Was a consecutive or random sample of patients enrolled? (Yes/Unclear/No)
  - *Scored yes if the words in article state "consecutive", "random", or "all patients were included*
- Was a case-control design avoided? (Yes/Unclear/No)
  - *Scored yes if article stated the study design as not being case-control*
- Did the study avoid inappropriate exclusions? (Yes/Unclear/No)
  - *Scored yes if studies excluded no children with abdominal pain or suspected appendicitis.*
- (Risk) Could the selection of patients have introduced bias? (Low / Unclear/ High)
  - *Scored low if all answers to signalling questions were yes. Score high if any were answered no. Score unclear if any were answered as unclear with remainder scoring low.*
- (Concern) Is there concern that included patients do not match the review question? (Low / Unclear /High)
  - *Scored low if all patients were <18 years, had symptoms in keeping with appendicitis and no previous diagnosis of appendicitis*

Domain 2: Index Test

- Were the index tests interpreted without knowledge of the results of the reference standard? (Yes / Unclear/ No)
  - *Scored yes if text stated blood test results were concealed or blinded to clinician or pathologist.*
- If a threshold was used, was it pre-specified? (Yes / Unclear/ No)
  - *Scored yes if a cut-off for index test was stated before commencing study or analysing results*
- (Risk) Could the conduct or interpretation of the index test have introduced bias? (Low / Unclear/ High)
  - *Scored low if all answers to signalling questions were yes. Score high if any were answered no. Score unclear if any were answered as unclear with remainder scoring low.*
- (Concern) Is there concern that the index test, its conduct, or interpretation differ from the review question? (Low / Unclear / High)
  - *Scored low if the study provides a clear description of the index test and a clear description or definition of a positive test result*

Domain 3: Reference Standard

- Is the reference standard likely to correctly classify the target condition? (Yes / Unclear/ No)
  - *Score yes if the reference standard was histology after surgery or clinical follow up of patient after 2 weeks. Score no if the reference standard was surgery alone without histology.*
- Were the reference standard results interpreted without knowledge of the results of the index test? (Yes / Unclear/ No)
  - *Score yes if study states the assessment of reference standard was done without knowledge or with blinding of the results of the index test*
- (Risk) Could the reference standard, its conduct, or its interpretation have introduced bias? (Low / Unclear/ High)
  - *Scored low if all answers to signalling questions were yes. Score high if any were answered no. Score unclear if any were answered as unclear with remainder scoring low.*
- (Concern) Is there concern that the target condition as defined by the reference standard does not match the review question? (Low / Unclear/ High)
  - *Score Low if all patients were in keeping with the target condition of appendicitis as defined by histology after surgery or refuted by clinical follow-up of greater than or equal to 2 weeks*

Domain 4: Flow and Timing

- Was there an appropriate interval between index test(s) and reference standard? (Yes / Unclear/ No)
  - *Score yes if the time period was 48 hours or less and was stated in text; Score no if the time period was more than 48 hours. Score unclear if insufficient information to determine the interval between index test and reference standard.*

For peer review only


For peer review only - http://bmjopen.bmj.com/site/about/guidelines.xhtml

- Did all patients receive a reference standard? (Yes / Unclear/ No)
    - *Score yes if all patients received either appendicectomy with histology or clinical follow up of greater than or equal to 2 weeks to refute appendicitis*
- Did patients receive the same reference standard? (Yes / Unclear/ No)
    - *Score yes if all patients received the same reference standard.*
- Were all patients included in the analysis? (Yes / Unclear/ No)
    - *Score yes if all patients included at patient selection underwent index test and reference standard.*
- (Risk) Could the patient flow have introduced bias? (Low / Unclear/ High)
    - *Scored low if all answers to signalling questions were yes. Score high if any were answered no. Score unclear if any were answered as unclear with remainder scoring low.*

For peer review only

**Supplementary References**

1. Akgül F, Er A, Ulusoy E, Çağlar A, Çitlenbik H, Keskinoğlu P, et al. Integration of Physical Examination, Old and New Biomarkers, and Ultrasonography by Using Neural Networks for Pediatric Appendicitis. Pediatr Emerg Care 2021 Dec 1;37(12):e1075-e1081.
2. Altali K, Ruiz-Artacho P, Trenchs V, Martinez Ortiz de Zarate M, Navarro C, Fernandez C, et al. [Hospital emergency room diagnosis of acute appendicitis in patients aged 2 to 20 years: the INFURG-SEMES score from the emergency infections study of the Spanish Society of Emergency Medicine]. Emergencias : revista de la Sociedad Espanola de Medicina de Emergencias. 2017;29(4):231-6.
3. Anandalwar SP, Callahan MJ, Bachur RG, Feng C, Sidhwa F, Karki M, et al. Use of White Blood Cell Count and Polymorphonuclear Leukocyte Differential to Improve the Predictive Value of Ultrasound for Suspected Appendicitis in Children. Journal of the American College of Surgeons. 2015;220(6):1010-7.
4. Andersson RE, Hugander AP, Ghazi SH, Ravn H, Offenbartl SK, Nystrom PO, et al. Diagnostic value of disease history, clinical presentation, and inflammatory parameters of appendicitis. World J Surg. 1999;23(2):133-40.
5. Andersson M, Andersson RE. The appendicitis inflammatory response score: a tool for the diagnosis of acute appendicitis that outperforms the Alvarado score. World J Surg. 2008;32(8):1843-9.
6. Andersson M, Rubér M, Ekerfelt C, Hallgren HB, Olaison G, Andersson RE. Can New Inflammatory Markers Improve the Diagnosis of Acute Appendicitis? World Journal of Surgery. 2014;38(11):2777-83.
7. Andersson M, Kolodziej B, Andersson RE. Randomized clinical trial of Appendicitis Inflammatory Response score-based management of patients with suspected appendicitis. The British journal of surgery. 2017;104(11):1451-61.
8. Bachur RG, Dayan PS, Dudley NC, Bajaj L, Stevenson MD, Macias CG, et al. The Influence of Age on the Diagnostic Performance of White Blood Cell Count and Absolute Neutrophil Count in Suspected Pediatric Appendicitis. Academic emergency medicine : official journal of the Society for Academic Emergency Medicine. 2016;23(11):1235-42.
9. Bal A, Anil M, Narturk M, Ozdemir T, Arikan A, Koyluoglu G, et al. Importance of Clinical Decision Making by Experienced Pediatric Surgeons When Children Are Suspected of Having Acute Appendicitis: The Reality in a High-Volume Pediatric Emergency Department. Pediatr Emerg Care. 2017;33(9):e38-e42.
10. Beltrán A M, Almonacid F J, Gutiérrez C J, Cruces B K. Puntuación diagnóstica de apendicitis aguda en niños realizada por pediatras de las Unidades de Emergencia. Revista chilena de pediatría. 2007;78:584-91.
11. Benito J, Acedo Y, Medrano L, Barcena E, Garay RP, Arri EA. Usefulness of new and traditional serum biomarkers in children with suspected appendicitis. The American journal of emergency medicine. 2016;34(5):871-6.
12. Calvo Rigual F, Sendra Esteve S, Mialaret Lahiguera A, Montagud Beltrán E, Llanes Domingo S, Medrano González J. [The value of C-reactive protein in the diagnosis of acute appendicitis in children]. An Esp Pediatr. 1998;48(4):376-80.
13. Cayrol J, Miguez MC, Guerrero G, Tomatis C, Simal I, Maranon R. Diagnostic accuracy and prognostic utility of D Dimer in acute appendicitis in children. European journal of pediatrics. 2016;175(3):313-20.
14. Chiang JJY, Angus MI, Nah SA, Jacobsen AS, Low Y, Choo CSC, et al. Time course response of inflammatory markers in pediatric appendicitis. Pediatr Surg Int 2020 Apr;36(4):493-500.
15. Corkum KS, Oyetunji TA, Grabowski JE, Rigsby CK, Lautz TB. Absolute neutrophil count as a diagnostic guide for the use of MRI in the workup of suspected appendicitis in children. Journal of pediatric surgery. 2019;54(7):1359-64.
16. Dadeh AA, Puitong K. Predictive Factors to Diagnose Appendicitis in Children in the Emergency Department. Open Access Emerg Med 2021 Jul 29;13:363-372.
17. Doraiswamy NV. Leucocyte counts in the diagnosis and prognosis of acute appendicitis in children. The British journal of surgery. 1979;66(11):782-4.
18. Esparaz JR, McGovern GC, Mowrer AR, Nierstedt RT, Biesboer EA, Elger BM, et al. A simple algorithm to improve quality while reducing resource utilization in evaluation of suspected appendicitis in children. American journal of surgery. 2019;217(3):469-72.
19. Fleischman RJ, Devine MK, Yagapen MA, Steichen AJ, Hansen ML, Zigman AF, et al. Evaluation of a novel pediatric appendicitis pathway using high- and low-risk scoring systems. Pediatr Emerg Care. 2013;29(10):1060-5.
20. Goldman RD, Carter S, Stephens D, Antoon R, Mounstephen W, Langer JC. Prospective validation of the pediatric appendicitis score. The Journal of pediatrics. 2008;153(2):278-82.
21. Gonzalez Del Castillo J, Ayuso FJ, Trenchs V, Martinez Ortiz de Zarate M, Navarro C, Altali K, et al. Diagnostic accuracy of the APPY1 Test in patients aged 2-20 years with suspected acute appendicitis presenting to emergency departments. Emergency medicine journal : EMJ. 2016;33(12):853-9.


For peer review only - http://bmjopen.bmj.com/site/about/guidelines.xhtml

22. Greer D, Bennett P, Wagstaff B, Croaker D. Lymphopaenia in the diagnosis of paediatric appendicitis: a false sense of security? ANZ J Surg 2019 Sep;89(9):1122-1125.
23. Groselj-Grenc M, Repse S, Dolenc-Strazar Z, Hojker S, Derganc M. Interleukin-6 and lipopolysaccharide-binding protein in acute appendicitis in children. Scandinavian journal of clinical and laboratory investigation. 2007;67(2):197-206.
24. Hsiao KH, Lin LH, Chen DF. Application of the MANTRELS scoring system in the diagnosis of acute appendicitis in children. Acta paediatrica Taiwanica = Taiwan er ke yi xue hui za zhi. 2005;46(3):128-31.
25. Huckins DS, Simon HK, Copeland K, Spiro DM, Gogain J, Wandell M. A novel biomarker panel to rule out acute appendicitis in pediatric patients with abdominal pain. The American journal of emergency medicine. 2013;31(9):1368-75.
26. Huckins DS, Simon HK, Copeland K, Milling TJ, Jr., Spandorfer PR, Hennes H, et al. Prospective validation of a biomarker panel to identify pediatric ED patients with abdominal pain who are at low risk for acute appendicitis. The American journal of emergency medicine. 2016;34(8):1373-82.
27. Kaiser M, Schroeckenfuchs M, Castellani C, Warncke G, Till H, Singer G. The diagnostic value of hepcidin to predict the presence and severity of appendicitis in children. The Journal of surgical research. 2018;222:102-7.
28. Kashtan M, Graham D, Anandalwar S, Hills-Dunlap J, Rangel S. Influence of symptom duration and WBC profile on the negative predictive value of a nondiagnostic ultrasound in children with suspected appendicitis. J Pediatr Surg 2020 Jun;55(6):1032-1036.
29. Khan AN, Sawan A, Likourezos A, Schnellinger M, Garcia E. The usefulness of procalcitonin in the diagnosis of appendicitis in children: a pilot study. Emergency medicine international. 2012;2012:317504.
30. Khanafer I, Martin DA, Mitra TP, Eccles R, Brindle ME, Nettel-Aguirre A, et al. Test characteristics of common appendicitis scores with and without laboratory investigations: a prospective observational study. BMC Pediatr. 2016;16(1):147.
31. Kharbanda AB, Taylor GA, Fishman SJ, Bachur RG. A clinical decision rule to identify children at low risk for appendicitis. Pediatrics. 2005;116(3):709-16.
32. Kharbanda AB, Rai AJ, Cosme Y, Liu K, Dayan PS. Novel serum and urine markers for pediatric appendicitis. Academic emergency medicine : official journal of the Society for Academic Emergency Medicine. 2012;19(1):56-62.
33. Kharbanda AB, Dudley NC, Bajaj L, Stevenson MD, Macias CG, Mittal MK, et al. Validation and refinement of a prediction rule to identify children at low risk for acute appendicitis. Archives of pediatrics & adolescent medicine. 2012;166(8):738-44.
34. Klein TT, Kohn E, Klin B, Ziv-Baran T, Kozer E, Berkovitch M, et al. sTREM-1 as a diagnostic biomarker for acute appendicitis in children. Asian J Surg 2021 Sep;44(9):1172-1178.
35. Ko YS, Lin LH, Chen DF. Laboratory aid and ultrasonography in the diagnosis of appendicitis in children. Zhonghua Minguo xiao er ke yi xue hui za zhi [Journal] Zhonghua Minguo xiao er ke yi xue hui. 1995;36(6):415-9.
36. Kouame DB, Garrigue MA, Lardy H, Machet MC, Giraudeau B, Robert M. [Is procalcitonin able to help in pediatric appendicitis diagnosis?]. Annales de chirurgie. 2005;130(3):169-74.
37. Kumar MSV, Tiwari MK, Singh J, Malik A. Plasma Fibrinogen: An Independent Predictor of Pediatric Appendicitis. J Indian Assoc Pediatr Surg 2021 Jul-Aug;26(4):240-245.
38. Kwan KY, Nager AL. Diagnosing pediatric appendicitis: usefulness of laboratory markers. The American journal of emergency medicine. 2010;28(9):1009-15.
39. Lin CH, Chen JH, Li TC, Ho YJ, Lin WC. Children presenting at the emergency department with right lower quadrant pain. The Kaohsiung journal of medical sciences. 2009;25(1):1-9.
40. Lycopoulou L, Mamoulakis C, Hantzi E, Demetriadis D, Antypas S, Giannaki M, et al. Serum amyloid A protein levels as a possible aid in the diagnosis of acute appendicitis in children. Clinical chemistry and laboratory medicine. 2005;43(1):49-53.
41. Malia L, Sturm JJ, Smith SR, Brown RT, Campbell B, Chicaiza H. Predictors for Acute Appendicitis in Children. Pediatr Emerg Care. 2019.
42. Mandeville K, Pottker T, Bulloch B, Liu J. Using appendicitis scores in the pediatric ED. The American journal of emergency medicine. 2011;29(9):972-7.
43. Miguez C, Tomatis Souverbielle C, Haro A, Guerrero G, Perez-Egido L, Garcia-Gamiz M, et al. Evaluation of proadrenomedullin as a diagnostic or prognostic biomarker of acute appendicitis in children. The American journal of emergency medicine. 2016;34(12):2298-305.
44. Mohammed AA, Daghman NA, Aboud SM, Oshibi HO. The diagnostic value of C-reactive protein, white blood cell count and neutrophil percentage in childhood appendicitis. Saudi medical journal. 2004;25(9):1212-5.


For peer review only - http://bmjopen.bmj.com/site/about/guidelines.xhtml

45. Diaz Moreno E, Garcia Gomez M, Castejon Casado J, Liceras Liceras E, Martin Cano F, Munoz Miguelsanz MA. [Analysis of the medical decision in abdominal pain suggestive of acute appendicitis]. Cirugia pediatrica : organo oficial de la Sociedad Espanola de Cirugia Pediatrica. 2012;25(1):40-5.
46. Naqvi SA, Thompson GC, Joffe AR, Blackwood J, Martin DA, Brindle M, et al. Cytokines and Chemokines in Pediatric Appendicitis: A Multiplex Analysis of Inflammatory Protein Mediators. Mediators of inflammation. 2019;2019:2359681.
47. Oikonomopoulou N, Miguez-Navarro C, Rivas-Garcia A, Garcia Gamiz M, Lopez-Lopez R, Oliver-Saez P, et al. Assessment of proadrenomedullin as diagnostic or prognostic biomarker of acute appendicitis in children with acute abdominal pain. The American journal of emergency medicine. 2019;37(7):1289-94.
48. Ovrebo KK, Eckerbom RM, Haram S, Rokke O. [Acute abdomen among children and adolescents. A retrospective study of 470 children and adolescents with acute abdominal pain]. Tidsskrift for den Norske laegeforening : tidsskrift for praktisk medicin, ny raekke. 1993;113(26):3244-7.
49. Ozguner I, Kizilgun M, Karaman A, Cavusoglu YH, Erdogan D, Karaman I, et al. Are neutrophil CD64 expression and interleukin-6 early useful markers for diagnosis of acute appendicitis? European journal of pediatric surgery : official journal of Austrian Association of Pediatric Surgery [et al] = Zeitschrift fur Kinderchirurgie. 2014;24(2):179-83.
50. Peltola H, Ahlqvist J, Rapola J, Rasanen J, Louhimo I, Saarinen M, et al. C-reactive protein compared with white blood cell count and erythrocyte sedimentation rate in the diagnosis of acute appendicitis in children. Acta chirurgica Scandinavica. 1986;152:55-8.
51. Prada-Arias M, Vazquez JL, Salgado-Barreira A, Gomez-Veiras J, Montero-Sanchez M, Fernandez-Lorenzo JR. Diagnostic accuracy of fibrinogen to differentiate appendicitis from nonspecific abdominal pain in children. The American journal of emergency medicine. 2017;35(1):66-70.
52. A. Rodríguez Sánchez de la Blanca RMP, J. Lorente Romero, A. Rivas García, P. Vázquez López, C. Míguez Navarro. [Usefulness of procalcitonin in early diagnosis of acute appendicitis]. Acta Pediatr Esp. 2014;72(7):4.
53. Salo M, Roth B, Stenstrom P, Arnbjornsson E, Ohlsson B. Urinary biomarkers in pediatric appendicitis. Pediatric surgery international. 2016;32(8):795-804.
54. Samuel M. Pediatric appendicitis score. Journal of pediatric surgery. 2002;37(6):877-81.
55. Sanchez Echaniz J, Luis Garcia M, Vazquez Ronco MA, Mintegui Raso S, Benito Fernandez J, Lopez Alvarez-Buhilla P. [Diagnostic value of reactive C protein in suspected acute appendicitis in children]. An Esp Pediatr. 1998;48(5):470-4.
56. Shommu NS, Jenne CN, Blackwood J, Martin DA, Joffe AR, Eccles R, et al. The Use of Metabolomics and Inflammatory Mediator Profiling Provides a Novel Approach to Identifying Pediatric Appendicitis in the Emergency Department. Scientific reports. 2018;8(1):4083.
57. Thompson GC, Schuh S, Gravel J, Reid S, Fitzpatrick E, Turner T, et al. Variation in the Diagnosis and Management of Appendicitis at Canadian Pediatric Hospitals. Academic emergency medicine : official journal of the Society for Academic Emergency Medicine. 2015;22(7):811-22.
58. Thompson GC, Morrison E, Ross M, Liu H, Vanderkooi OG, Eccles R. The Use of Routine Blood Cultures in Pediatric Appendicitis. Pediatr Emerg Care. 2017;33(12):e160-e3.
59. Tuncer AA, Cavus S, Balcioglu A, Silay S, Demiralp I, Calkan E, et al. Can mean platelet volume, Neutrophil-to-Lymphocyte, Lymphocyte-to-Monocyte, Platelet-to-Lymphocyte ratios be favourable predictors for the differential diagnosis of appendicitis? JPMA The Journal of the Pakistan Medical Association. 2019;69(5):647-54.
60. Turkyilmaz Z, Sonmez K, Karabulut R, Elbeg S, Moralioglu S, Demirtola A, et al. Sequential cytokine levels in the diagnosis of appendicitis. Scandinavian journal of clinical and laboratory investigation. 2006;66(8):723-31.
61. van den Broek WT, van der Ende ED, Bijnen AB, Breslau PJ, Gouma DJ. Which children could benefit from additional diagnostic tools in case of suspected appendicitis? Journal of pediatric surgery. 2004;39(4):570-4.
62. Wang LT, Prentiss KA, Simon JZ, Doody DP, Ryan DP. The use of white blood cell count and left shift in the diagnosis of appendicitis in children. Pediatr Emerg Care. 2007;23(2):69-76.
63. Wu HP, Chen CY, Kuo IT, Wu YK, Fu YC. Diagnostic values of a single serum biomarker at different time points compared with Alvarado score and imaging examinations in pediatric appendicitis. The Journal of surgical research. 2012;174(2):272-7.
64. Yap TL, Chen Y, Low WW, Ong CC, Nah SA, Jacobsen AS, et al. A new 2-step risk-stratification clinical score for suspected appendicitis in children. Journal of pediatric surgery. 2015;50(12):2051-5.
65. Yazici M, Ozkisacik S, Oztan MO, Gursoy H. Neutrophil/lymphocyte ratio in the diagnosis of childhood appendicitis. The Turkish journal of pediatrics. 2010;52(4):400-3.

For peer review only

66. Zouari M, Jallouli M, Louati H, Kchaou R, Chtourou R, Kotti A, et al. Predictive value of C-reactive protein, ultrasound and Alvarado score in acute appendicitis: a prospective pediatric cohort. The American journal of emergency medicine. 2016;34(2):189-92.
67. Zuniga RV, Arribas JL, Montes SP, Fernandez MN, Abad CG, Martin LG, et al. Application of Pediatric Appendicitis Score on the emergency department of a secondary level hospital. Pediatr Emerg Care. 2012;28(6):489-92.
68. Deeks JJ, Macaskill P, Irwig L. The performance of tests of publication bias and other sample size effects in systematic reviews of diagnostic test accuracy was assessed. Journal of Clinical Epidemiology. 2005;58(9):882-93.
69. Whiting PF, Rutjes AWS, Westwood ME, Mallett S, Deeks JJ, Reitsma JB, et al. QUADAS-2: A Revised Tool for the Quality Assessment of Diagnostic Accuracy Studies. Annals of Internal Medicine. 2011;155(8):529-36.

For peer review only



For peer review only - http://bmjopen.bmj.com/site/about/guidelines.xhtml

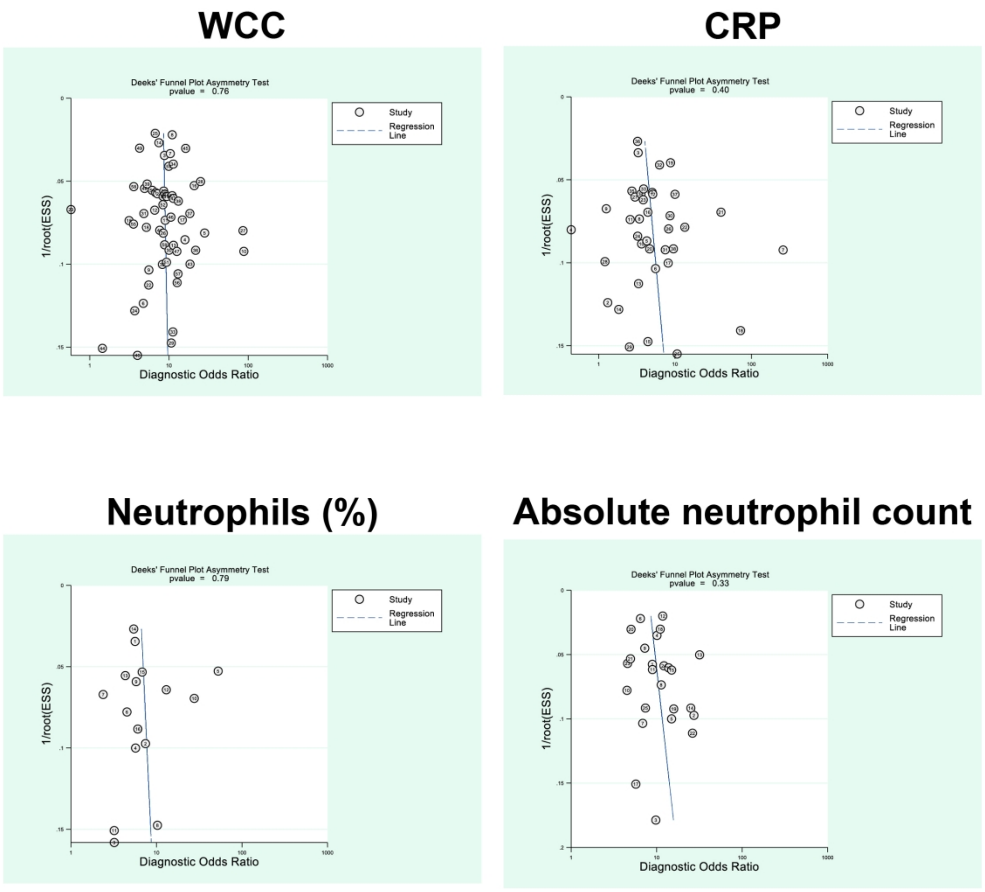


210x188mm (300 x 300 DPI)

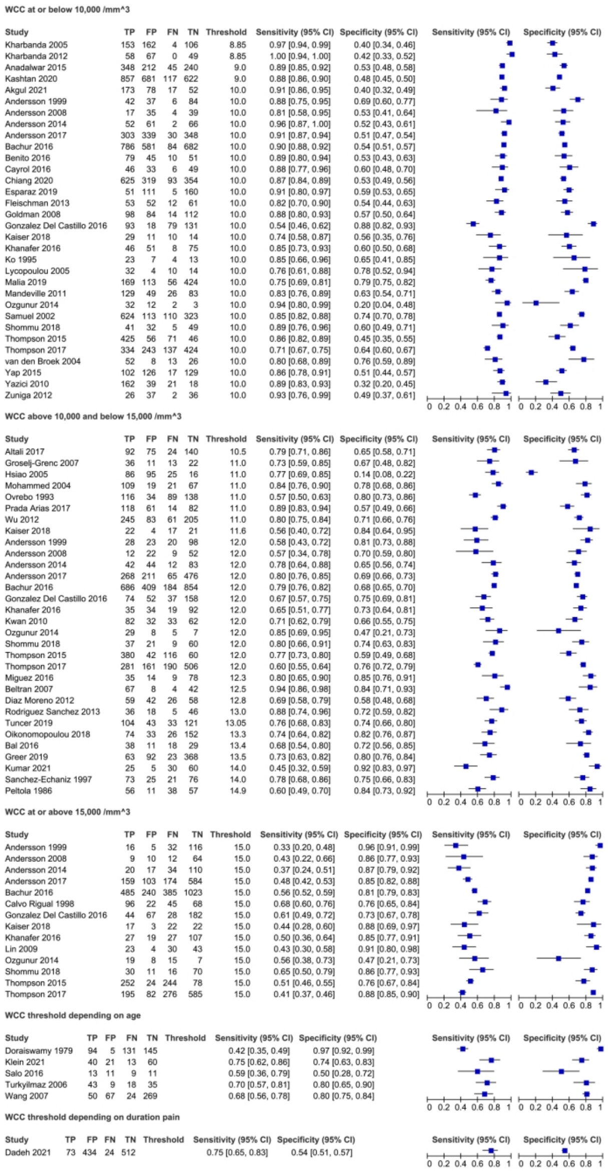


135x264mm (300 x 300 DPI)

**CRP at or below 5 mg/l**

| Study | TP | FP | FN | TN | Threshold | Sensitivity (95% CI) | Specificity (95% CI) |
|---|---|---|---|---|---|---|---|
| Bal 2016 | 50 | 24 | 6 | 16 | 0.9 | 0.89 [0.78, 0.96] | 0.40 [0.25, 0.57] |
| Miguez 2016 | 30 | 29 | 14 | 63 | 3.0 | 0.68 [0.52, 0.81] | 0.68 [0.58, 0.78] |
| Lycopoulou 2005 | 34 | 1 | 8 | 17 | 4.5 | 0.81 [0.66, 0.91] | 0.94 [0.73, 1.00] |
| Akgul 2021 | 146 | 72 | 44 | 59 | 5.0 | 0.77 [0.70, 0.83] | 0.45 [0.36, 0.54] |
| Altali 2017 | 92 | 93 | 24 | 122 | 5.0 | 0.79 [0.71, 0.86] | 0.57 [0.50, 0.63] |
| Andersson 1999 | 29 | 34 | 17 | 85 | 5.0 | 0.63 [0.48, 0.77] | 0.71 [0.62, 0.79] |
| Andersson 2008 | 17 | 55 | 4 | 17 | 5.0 | 0.81 [0.58, 0.95] | 0.24 [0.14, 0.35] |
| Andersson 2014 | 55 | 127 | 0 | 0 | 5.0 | 1.00 [0.94, 1.00] | 0.00 [0.00, 0.03] |
| Andersson 2017 | 285 | 481 | 38 | 211 | 5.0 | 0.88 [0.84, 0.92] | 0.30 [0.27, 0.34] |
| Chiang 2020 | 589 | 394 | 129 | 279 | 5.0 | 0.82 [0.79, 0.85] | 0.41 [0.38, 0.45] |
| Gonzalez Del Castillo 2016 | 88 | 90 | 23 | 120 | 5.0 | 0.79 [0.71, 0.86] | 0.57 [0.50, 0.64] |
| Greer 2019 | 69 | 133 | 17 | 327 | 5.0 | 0.80 [0.70, 0.88] | 0.71 [0.67, 0.75] |
| Kaiser 2018 | 23 | 11 | 16 | 14 | 5.0 | 0.59 [0.42, 0.74] | 0.56 [0.35, 0.76] |
| Malia 2019 | 160 | 118 | 65 | 419 | 5.0 | 0.71 [0.65, 0.77] | 0.78 [0.74, 0.81] |
| Ozgunur 2014 | 32 | 9 | 2 | 6 | 5.0 | 0.94 [0.80, 0.99] | 0.40 [0.16, 0.68] |
| Rodriguez Sanchez 2013 | 21 | 29 | 21 | 35 | 5.0 | 0.50 [0.34, 0.66] | 0.55 [0.42, 0.67] |
| Yap 2015 | 99 | 143 | 20 | 112 | 5.0 | 0.83 [0.75, 0.89] | 0.44 [0.38, 0.50] |

**CRP between 5 and 11 mg/l**

| Study | TP | FP | FN | TN | Threshold | Sensitivity (95% CI) | Specificity (95% CI) |
|---|---|---|---|---|---|---|---|
| Zouari 2016 | 107 | 74 | 32 | 79 | 6.0 | 0.77 [0.69, 0.84] | 0.52 [0.43, 0.60] |
| Naqvi 2019 | 64 | 20 | 15 | 63 | 7.9 | 0.81 [0.71, 0.89] | 0.76 [0.65, 0.85] |
| Mohammed 2004 | 114 | 13 | 16 | 73 | 8.0 | 0.88 [0.81, 0.93] | 0.85 [0.76, 0.92] |
| Groselj-Grenc 2007 | 36 | 15 | 13 | 18 | 9.0 | 0.73 [0.59, 0.85] | 0.55 [0.36, 0.72] |
| Ko 1995 | 19 | 7 | 8 | 13 | 9.0 | 0.70 [0.50, 0.86] | 0.65 [0.41, 0.85] |
| Andersson 1999 | 23 | 26 | 23 | 93 | 10.0 | 0.50 [0.35, 0.65] | 0.78 [0.70, 0.85] |
| Andersson 2008 | 15 | 25 | 6 | 47 | 10.0 | 0.71 [0.48, 0.89] | 0.65 [0.53, 0.76] |
| Andersson 2014 | 43 | 53 | 12 | 74 | 10.0 | 0.78 [0.65, 0.88] | 0.58 [0.49, 0.67] |
| Andersson 2017 | 221 | 249 | 102 | 443 | 10.0 | 0.68 [0.63, 0.73] | 0.64 [0.60, 0.68] |
| Gonzalez Del Castillo 2016 | 75 | 70 | 36 | 140 | 10.0 | 0.68 [0.58, 0.76] | 0.67 [0.60, 0.73] |
| Kaiser 2018 | 20 | 10 | 19 | 15 | 10.0 | 0.51 [0.35, 0.68] | 0.60 [0.39, 0.79] |
| Lin 2009 | 39 | 12 | 14 | 35 | 10.0 | 0.74 [0.60, 0.85] | 0.74 [0.60, 0.86] |
| Lycopoulou 2005 | 26 | 1 | 16 | 17 | 10.0 | 0.62 [0.46, 0.76] | 0.94 [0.73, 1.00] |
| Ovrebo 1993 | 48 | 80 | 5 | 27 | 10.0 | 0.91 [0.79, 0.97] | 0.25 [0.17, 0.35] |
| Ozgunur 2014 | 25 | 7 | 9 | 8 | 10.0 | 0.74 [0.56, 0.87] | 0.53 [0.27, 0.79] |
| Peltola 1986 | 60 | 12 | 34 | 56 | 10.0 | 0.64 [0.53, 0.73] | 0.82 [0.71, 0.91] |
| Kumar 2021 | 49 | 30 | 6 | 35 | 10.5 | 0.89 [0.78, 0.96] | 0.54 [0.41, 0.66] |

**CRP between 11 and 50 mg/l**

| Study | TP | FP | FN | TN | Threshold | Sensitivity (95% CI) | Specificity (95% CI) |
|---|---|---|---|---|---|---|---|
| Oikonomopoulou 2018 | 60 | 52 | 40 | 133 | 12.5 | 0.60 [0.50, 0.70] | 0.72 [0.65, 0.78] |
| Salo 2016 | 14 | 9 | 8 | 13 | 15.0 | 0.64 [0.41, 0.83] | 0.59 [0.36, 0.79] |
| Ozgunur 2014 | 24 | 5 | 10 | 10 | 16.0 | 0.71 [0.53, 0.85] | 0.67 [0.38, 0.88] |
| Diaz Moreno 2012 | 46 | 31 | 39 | 68 | 18.0 | 0.54 [0.43, 0.65] | 0.69 [0.59, 0.78] |
| Benito 2016 | 31 | 13 | 58 | 83 | 20.0 | 0.35 [0.25, 0.46] | 0.86 [0.78, 0.93] |
| Calvo Rigual 1998 | 61 | 34 | 80 | 56 | 20.0 | 0.43 [0.35, 0.52] | 0.62 [0.51, 0.72] |
| Shommu 2018 | 24 | 9 | 24 | 67 | 20.0 | 0.50 [0.35, 0.65] | 0.88 [0.79, 0.94] |
| Wu 2012 | 214 | 78 | 92 | 210 | 25.0 | 0.70 [0.64, 0.75] | 0.73 [0.67, 0.78] |
| Prada Arias 2017 | 57 | 29 | 75 | 114 | 25.2 | 0.43 [0.35, 0.52] | 0.80 [0.72, 0.86] |
| Andersson 1999 | 14 | 14 | 32 | 105 | 30.0 | 0.30 [0.18, 0.46] | 0.88 [0.81, 0.93] |
| Andersson 2008 | 8 | 11 | 13 | 61 | 30.0 | 0.38 [0.18, 0.62] | 0.85 [0.74, 0.92] |
| Andersson 2014 | 31 | 35 | 24 | 92 | 30.0 | 0.56 [0.42, 0.70] | 0.72 [0.64, 0.80] |
| Andersson 2017 | 140 | 128 | 183 | 564 | 30.0 | 0.43 [0.38, 0.49] | 0.82 [0.78, 0.84] |
| Cayrol 2016 | 20 | 12 | 32 | 70 | 30.0 | 0.38 [0.25, 0.53] | 0.85 [0.76, 0.92] |
| Gonzalez Del Castillo 2016 | 59 | 40 | 52 | 170 | 30.0 | 0.53 [0.43, 0.63] | 0.81 [0.75, 0.86] |
| Kaiser 2018 | 13 | 8 | 26 | 17 | 30.0 | 0.33 [0.19, 0.50] | 0.68 [0.46, 0.85] |
| Kwan 2010 | 81 | 33 | 34 | 61 | 30.0 | 0.70 [0.61, 0.79] | 0.65 [0.54, 0.74] |
| Sanchez-Echaniz 1997 | 40 | 8 | 54 | 93 | 30.0 | 0.43 [0.32, 0.53] | 0.92 [0.85, 0.97] |
| Beltran 2007 | 68 | 4 | 3 | 46 | 47.0 | 0.96 [0.88, 0.99] | 0.92 [0.81, 0.98] |

**CRP at or above 50 mg/l**

| Study | TP | FP | FN | TN | Threshold | Sensitivity (95% CI) | Specificity (95% CI) |
|---|---|---|---|---|---|---|---|
| Andersson 1999 | 7 | 10 | 39 | 109 | 50.0 | 0.15 [0.06, 0.29] | 0.92 [0.85, 0.96] |
| Andersson 2008 | 5 | 7 | 16 | 65 | 50.0 | 0.24 [0.08, 0.47] | 0.90 [0.81, 0.96] |
| Andersson 2014 | 20 | 21 | 35 | 106 | 50.0 | 0.36 [0.24, 0.50] | 0.83 [0.76, 0.89] |
| Andersson 2017 | 93 | 62 | 230 | 630 | 50.0 | 0.29 [0.24, 0.34] | 0.91 [0.89, 0.93] |
| Gonzalez Del Castillo 2016 | 37 | 28 | 74 | 182 | 50.0 | 0.33 [0.25, 0.43] | 0.87 [0.81, 0.91] |
| Kaiser 2018 | 9 | 2 | 30 | 23 | 50.0 | 0.23 [0.11, 0.39] | 0.92 [0.74, 0.99] |
| Ko 1995 | 14 | 1 | 13 | 19 | 50.0 | 0.52 [0.32, 0.71] | 0.95 [0.75, 1.00] |
| Ozgunur 2014 | 15 | 3 | 19 | 12 | 50.0 | 0.44 [0.27, 0.62] | 0.80 [0.52, 0.96] |
| Shommu 2018 | 12 | 1 | 36 | 75 | 50.0 | 0.25 [0.14, 0.40] | 0.99 [0.93, 1.00] |
| Wu 2012 | 214 | 49 | 92 | 239 | 89.0 | 0.70 [0.64, 0.75] | 0.83 [0.78, 0.87] |

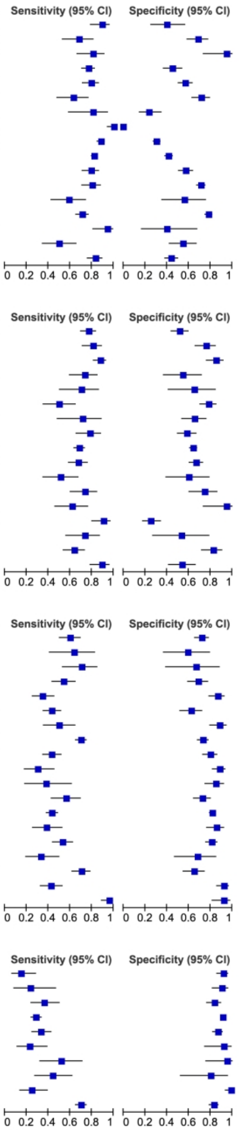


173x265mm (300 x 300 DPI)

| Study | TP | FP | FN | TN | Threshold | Sensitivity (95% CI) | Specificity (95% CI) |
|---|---|---|---|---|---|---|---|
| Salo 2016 | 16 | 7 | 6 | 15 | age dependent | 0.73 [0.50, 0.89] | 0.68 [0.45, 0.86] |
| Altali 2017 | 99 | 85 | 17 | 130 | 6.6 | 0.85 [0.78, 0.91] | 0.60 [0.54, 0.67] |
| Kharbanda 2012 | 942 | 824 | 76 | 783 | 6.73 | 0.93 [0.91, 0.94] | 0.49 [0.46, 0.51] |
| Fleischman 2013 | 53 | 56 | 12 | 57 | 6.75 | 0.82 [0.70, 0.90] | 0.50 [0.41, 0.60] |
| Kharbanda 2005 | 152 | 131 | 5 | 137 | 6.75 | 0.97 [0.93, 0.99] | 0.51 [0.45, 0.57] |
| Prada Arias 2017 | 119 | 57 | 13 | 86 | 7.0 | 0.90 [0.84, 0.95] | 0.60 [0.52, 0.68] |
| Akgul 2021 | 163 | 74 | 27 | 56 | 7.5 | 0.86 [0.80, 0.90] | 0.43 [0.34, 0.52] |
| Andersson 1999 | 34 | 38 | 2 | 61 | 7.5 | 0.94 [0.81, 0.99] | 0.62 [0.51, 0.71] |
| Andersson 2008 | 8 | 27 | 1 | 33 | 7.5 | 0.89 [0.52, 1.00] | 0.55 [0.42, 0.68] |
| Andersson 2014 | 35 | 32 | 3 | 41 | 7.5 | 0.92 [0.79, 0.98] | 0.56 [0.44, 0.68] |
| Andersson 2017 | 274 | 282 | 31 | 322 | 7.5 | 0.90 [0.86, 0.93] | 0.53 [0.49, 0.57] |
| Benito 2016 | 78 | 37 | 11 | 59 | 7.5 | 0.88 [0.79, 0.94] | 0.61 [0.51, 0.71] |
| Goldman 2008 | 84 | 67 | 17 | 121 | 7.5 | 0.83 [0.74, 0.90] | 0.64 [0.57, 0.71] |
| Greer 2019 | 69 | 115 | 17 | 345 | 7.5 | 0.80 [0.70, 0.88] | 0.75 [0.71, 0.79] |
| Samuel 2002 | 595 | 122 | 139 | 314 | 7.5 | 0.81 [0.78, 0.84] | 0.72 [0.68, 0.76] |
| Shommu 2018 | 40 | 24 | 6 | 57 | 7.5 | 0.87 [0.74, 0.95] | 0.70 [0.59, 0.80] |
| Thompson 2015 | 351 | 68 | 47 | 45 | 7.5 | 0.88 [0.85, 0.91] | 0.40 [0.31, 0.49] |
| Thompson 2017 | 319 | 206 | 141 | 457 | 7.5 | 0.69 [0.65, 0.74] | 0.69 [0.65, 0.72] |
| Zuniga 2012 | 27 | 37 | 1 | 36 | 7.5 | 0.96 [0.82, 1.00] | 0.49 [0.37, 0.61] |
| Corkum 2018 | 116 | 166 | 40 | 416 | 8.0 | 0.74 [0.67, 0.81] | 0.71 [0.68, 0.75] |
| Bal 2016 | 43 | 13 | 13 | 27 | 9.4 | 0.77 [0.64, 0.87] | 0.68 [0.51, 0.81] |
| Miguez 2016 | 37 | 16 | 7 | 76 | 9.6 | 0.84 [0.70, 0.93] | 0.83 [0.73, 0.90] |
| Bachur 2016 | 630 | 365 | 240 | 898 | 10.0 | 0.72 [0.69, 0.75] | 0.71 [0.69, 0.74] |
| Kumar 2021 | 28 | 8 | 27 | 57 | 10.48 | 0.51 [0.37, 0.65] | 0.88 [0.77, 0.95] |
| Oikonomopoulou 2018 | 71 | 26 | 29 | 159 | 10.8 | 0.71 [0.61, 0.80] | 0.86 [0.80, 0.91] |

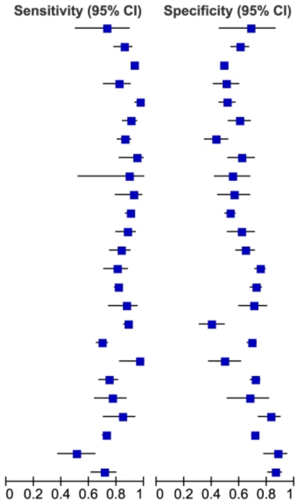


195x102mm (300 x 300 DPI)

| Study | TP | FP | FN | TN | Threshold | Sensitivity (95% CI) | Specificity (95% CI) |
|---|---|---|---|---|---|---|---|
| Anadalwar 2015 | 340 | 241 | 53 | 211 | 65% | 0.87 [0.83, 0.90] | 0.47 [0.42, 0.51] |
| Salo 2016 | 16 | 10 | 6 | 12 | 71% | 0.73 [0.50, 0.89] | 0.55 [0.32, 0.76] |
| Andersson 1999 | 31 | 45 | 5 | 54 | 75% | 0.86 [0.71, 0.95] | 0.55 [0.44, 0.65] |
| Andersson 2008 | 9 | 29 | 3 | 31 | 75% | 0.75 [0.43, 0.95] | 0.52 [0.38, 0.65] |
| Andersson 2014 | 30 | 29 | 8 | 44 | 75% | 0.79 [0.63, 0.90] | 0.60 [0.48, 0.72] |
| Chiang 2020 | 524 | 224 | 194 | 449 | 75% | 0.73 [0.70, 0.76] | 0.67 [0.63, 0.70] |
| Dadeh 2021 | 74 | 304 | 23 | 642 | 75% | 0.76 [0.67, 0.84] | 0.68 [0.65, 0.71] |
| Fleischman 2013 | 50 | 48 | 15 | 65 | 75% | 0.77 [0.65, 0.86] | 0.58 [0.48, 0.67] |
| Hsiao 2005 | 87 | 67 | 24 | 44 | 75% | 0.78 [0.70, 0.86] | 0.40 [0.30, 0.49] |
| Klein 2021 | 36 | 21 | 17 | 60 | 75% | 0.68 [0.54, 0.80] | 0.74 [0.63, 0.83] |
| Ko 1995 | 22 | 6 | 5 | 14 | 75% | 0.81 [0.62, 0.94] | 0.70 [0.46, 0.88] |
| Mandeville 2011 | 119 | 48 | 36 | 84 | 75% | 0.77 [0.69, 0.83] | 0.64 [0.55, 0.72] |
| Mohammed 2004 | 111 | 15 | 19 | 71 | 75% | 0.85 [0.78, 0.91] | 0.83 [0.73, 0.90] |
| Yap 2015 | 79 | 80 | 40 | 175 | 75% | 0.66 [0.57, 0.75] | 0.69 [0.63, 0.74] |
| Wang 2007 | 44 | 34 | 30 | 302 | 80% | 0.59 [0.47, 0.71] | 0.90 [0.86, 0.93] |
| Doraiswamy 1979 | 216 | 47 | 9 | 103 | age dependent | 0.96 [0.93, 0.98] | 0.69 [0.61, 0.76] |

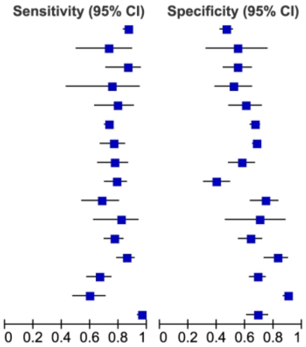


195x71mm (300 x 300 DPI)

| Study | TP | FP | FN | TN | Threshold | Sensitivity (95% CI) | Specificity (95% CI) |
|---|---|---|---|---|---|---|---|
| Benito 2016 | 34 | 34 | 55 | 62 | 0.1 | 0.38 [0.28, 0.49] | 0.65 [0.54, 0.74] |
| Bal 2016 | 18 | 4 | 38 | 36 | 0.37 | 0.32 [0.20, 0.46] | 0.90 [0.76, 0.97] |
| Khan 2012 | 6 | 2 | 16 | 26 | 0.39 | 0.27 [0.11, 0.50] | 0.93 [0.76, 0.99] |
| Rodriguez Sanchez 2013 | 10 | 18 | 34 | 46 | 0.5 | 0.23 [0.11, 0.38] | 0.72 [0.59, 0.82] |
| Kouame 2005 | 19 | 4 | 49 | 29 | 0.5 | 0.28 [0.18, 0.40] | 0.88 [0.72, 0.97] |
| Akgul 2021 | 55 | 19 | 135 | 111 | 0.5 | 0.29 [0.23, 0.36] | 0.85 [0.78, 0.91] |

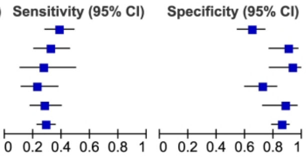


195x34mm (300 x 300 DPI)

**CRP and WCC**

| Study | TP | FP | FN | TN | Threshold WCC - CRP | Sensitivity (95% CI) | Specificity (95% CI) |
|---|---|---|---|---|---|---|---|
| Andersson 1999 | 20 | 12 | 26 | 107 | 10.0 - 10 | 0.43 [0.29, 0.59] | 0.90 [0.83, 0.95] |
| Andersson 2008 | 12 | 16 | 9 | 56 | 10.0 - 10 | 0.57 [0.34, 0.78] | 0.78 [0.66, 0.87] |
| Andersson 2014 | 39 | 31 | 14 | 96 | 10.0 - 10 | 0.74 [0.60, 0.85] | 0.76 [0.67, 0.83] |
| Andersson 2017 | 197 | 150 | 122 | 521 | 10.0 - 10 | 0.62 [0.56, 0.67] | 0.78 [0.74, 0.81] |
| Calvo Rigual 1998 | 113 | 45 | 28 | 45 | 15.0 - 20 | 0.80 [0.73, 0.86] | 0.50 [0.39, 0.61] |
| Huckins 2013 | 140 | 238 | 4 | 121 | 10.0 - 8 | 0.97 [0.93, 0.99] | 0.34 [0.29, 0.39] |
| Huckins 2016 | 457 | 775 | 19 | 635 | 10.0 - 8 | 0.96 [0.94, 0.98] | 0.45 [0.42, 0.48] |
| Kaiser 2018 | 16 | 7 | 23 | 18 | 10.0 - 10 | 0.41 [0.26, 0.58] | 0.72 [0.51, 0.88] |
| Kwan 2010 | 48 | 8 | 67 | 86 | 12.0 - 30 | 0.42 [0.33, 0.51] | 0.91 [0.84, 0.96] |
| Ovrebo 1993 | 39 | 0 | 14 | 107 | 11.0- 10 | 0.74 [0.60, 0.85] | 1.00 [0.97, 1.00] |
| Ozgunur 2014 | 24 | 5 | 10 | 10 | 10.0 - 10 | 0.71 [0.53, 0.85] | 0.67 [0.38, 0.88] |
| Peltola 1986 | 42 | 20 | 52 | 48 | 14.9 - 100 | 0.45 [0.34, 0.55] | 0.71 [0.58, 0.81] |
| Sanchez-Echaniz 1997 | 32 | 5 | 62 | 96 | 14.0 - 30 | 0.34 [0.25, 0.45] | 0.95 [0.89, 0.98] |

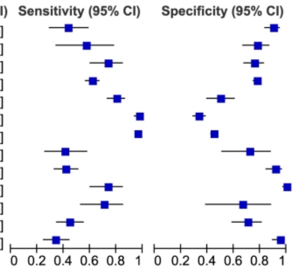


**CRP or WCC**

| Study | TP | FP | FN | TN | Threshold WCC - CRP | Sensitivity (95% CI) | Specificity (95% CI) |
|---|---|---|---|---|---|---|---|
| Chiang 2020 | 716 | 514 | 2 | 159 | 10.0 - 5 | 1.00 [0.99, 1.00] | 0.24 [0.20, 0.27] |

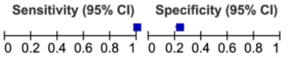


195x87mm (300 x 300 DPI)

| Study | TP | FP | FN | TN | Threshold WCC - CRP - NC% | Sensitivity (95% CI) | Specificity (95% CI) |
|---|---|---|---|---|---|---|---|
| Andersson 1999 | 15 | 11 | 20 | 88 | 10.0 - 10 - 75% | 0.43 [0.26, 0.61] | 0.89 [0.81, 0.94] |
| Andersson 2008 | 6 | 11 | 6 | 48 | 10.0 - 10 - 75% | 0.50 [0.21, 0.79] | 0.81 [0.69, 0.90] |
| Andersson 2014 | 21 | 12 | 17 | 61 | 10.0 - 10 - 75% | 0.55 [0.38, 0.71] | 0.84 [0.73, 0.91] |
| Andersson 2017 | 139 | 89 | 152 | 498 | 10.0 - 10 - 75% | 0.48 [0.42, 0.54] | 0.85 [0.82, 0.88] |
| Mohammed 2004 | 112 | 8 | 18 | 78 | 11.0 - 80 - 75% | 0.86 [0.79, 0.92] | 0.91 [0.82, 0.96] |
| Yap 2015 | 119 | 192 | 0 | 63 | 10.0 - 5 - 75% | 1.00 [0.97, 1.00] | 0.25 [0.20, 0.30] |

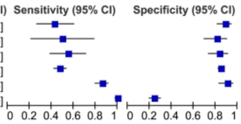


209x279mm (300 x 300 DPI)

# PRISMA-DTA Checklist

| Section/topic | # | PRISMA-DTA Checklist Item | Reported on page # |
|---|---|---|---|
| **TITLE / ABSTRACT** | | | |
| Title | 1 | Identify the report as a systematic review (+/- meta-analysis) of diagnostic test accuracy (DTA) studies. | 1 |
| Abstract | 2 | Abstract: See PRISMA-DTA for abstracts. | 2 |
| **INTRODUCTION** | | | |
| Rationale | 3 | Describe the rationale for the review in the context of what is already known. | 4 |
| Clinical role of index test | D1 | State the scientific and clinical background, including the intended use and clinical role of the index test, and if applicable, the rationale for minimally acceptable test accuracy (or minimum difference in accuracy for comparative design). | 4 and 5 |
| Objectives | 4 | Provide an explicit statement of question(s) being addressed in terms of participants, index test(s), and target condition(s). | 5 and 6 |
| **METHODS** | | | |
| Protocol and registration | 5 | Indicate if a review protocol exists, if and where it can be accessed (e.g., Web address), and, if available, provide registration information including registration number. | 6 |
| Eligibility criteria | 6 | Specify study characteristics (participants, setting, index test(s), reference standard(s), target condition(s), and study design) and report characteristics (e.g., years considered, language, publication status) used as criteria for eligibility, giving rationale. | 6 and 10 |
| Information sources | 7 | Describe all information sources (e.g., databases with dates of coverage, contact with study authors to identify additional studies) in the search and date last searched. | 6 and 7 and supplementary materials |
| Search | 8 | Present full search strategies for all electronic databases and other sources searched, including any limits used, such that they could be repeated. | 6 and supplementary materials |

For peer review only

# PRISMA-DTA Checklist

| | | | |
|---|---|---|---|
| Study selection | 9 | State the process for selecting studies (i.e., screening, eligibility, included in systematic review, and, if applicable, included in the meta-analysis). | 7 |
| Data collection process | 10 | Describe method of data extraction from reports (e.g., piloted forms, independently, in duplicate) and any processes for obtaining and confirming data from investigators. | 7 |
| Definitions for data extraction | 11 | Provide definitions used in data extraction and classifications of target condition(s), index test(s), reference standard(s) and other characteristics (e.g. study design, clinical setting). | 6 and 10 |
| Risk of bias and applicability | 12 | Describe methods used for assessing risk of bias in individual studies and concerns regarding the applicability to the review question. | 7 |
| Diagnostic accuracy measures | 13 | State the principal diagnostic accuracy measure(s) reported (e.g. sensitivity, specificity) and state the unit of assessment (e.g. per-patient, per-lesion). | 8 |
| Synthesis of results | 14 | Describe methods of handling data, combining results of studies and describing variability between studies. This could include, but is not limited to: a) handling of multiple definitions of target condition. b) handling of multiple thresholds of test positivity, c) handling multiple index test readers, d) handling of indeterminate test results, e) grouping and comparing tests, f) handling of different reference standards | 7 and 8 |



| Section/topic | # | PRISMA-DTA Checklist Item | Reported on page # |
|---|---|---|---|
| Meta-analysis | D2 | Report the statistical methods used for meta-analyses, if performed. | 8 and 9 |
| Additional analyses | 16 | Describe methods of additional analyses (e.g., sensitivity or subgroup analyses, meta-regression), if done, indicating which were pre-specified. | 8 |
| **RESULTS** | | | |
| Study selection | 17 | Provide numbers of studies screened, assessed for eligibility, included in the review (and included in meta-analysis, if applicable) with reasons for exclusions at each stage, ideally with a flow diagram. | Figure 1, 10 and supplementary materials |

For peer review only - http://bmjopen.bmj.com/site/about/guidelines.xhtml

# PRISMA-DTA Checklist

| | | | |
|---|---|---|---|
| Study characteristics | 18 | For each included study provide citations and present key characteristics including: a) participant characteristics (presentation, prior testing), b) clinical setting, c) study design, d) target condition definition, e) index test, f) reference standard, g) sample size, h) funding sources | 10 |
| Risk of bias and applicability | 19 | Present evaluation of risk of bias and concerns regarding applicability for each study. | 10 |
| Results of individual studies | 20 | For each analysis in each study (e.g. unique combination of index test, reference standard, and positivity threshold) report 2x2 data (TP, FP, FN, TN) with estimates of diagnostic accuracy and confidence intervals, ideally with a forest or receiver operator characteristic (ROC) plot. | 11,12 and 13 |
| Synthesis of results | 21 | Describe test accuracy, including variability; if meta-analysis was done, include results and confidence intervals. | 13 and 14 |
| Additional analysis | 23 | Give results of additional analyses, if done (e.g., sensitivity or subgroup analyses, meta-regression; analysis of index test: failure rates, proportion of inconclusive results, adverse events). | 13 |
| **DISCUSSION** | | | |
| Summary of evidence | 24 | Summarize the main findings including the strength of evidence. | 15 |
| Limitations | 25 | Discuss limitations from included studies (e.g. risk of bias and concerns regarding applicability) and from the review process (e.g. incomplete retrieval of identified research). | 16 and 17 |
| Conclusions | 26 | Provide a general interpretation of the results in the context of other evidence. Discuss implications for future research and clinical practice (e.g. the intended use and clinical role of the index test). | 18 |
| **FUNDING** | | | |
| Funding | 27 | For the systematic review, describe the sources of funding and other support and the role of the funders. | 21 |

*Adapted From:* McInnes MDF, Moher D, Thombs BD, McGrath TA, Bossuyt PM, The PRISMA-DTA Group (2018). Preferred Reporting Items for a Systematic Review and Meta-analysis of Diagnostic Test Accuracy Studies: The PRISMA-DTA Statement. JAMA. 2018 Jan 23;319(4):388-396. doi: 10.1001/jama.2017.19163.

For more information, visit: www.prisma-statement.org.



For peer review only - http://bmjopen.bmj.com/site/about/guidelines.xhtml

# PRISMA 2020 Checklist

| Section and Topic | Item # | Checklist item | Location where item is reported |
|---|---|---|---|
| **TITLE** | | | |
| Title | 1 | Identify the report as a systematic review. | 1 |
| **ABSTRACT** | | | |
| Abstract | 2 | See the PRISMA 2020 for Abstracts checklist. | 2 |
| **INTRODUCTION** | | | |
| Rationale | 3 | Describe the rationale for the review in the context of existing knowledge. | 4 |
| Objectives | 4 | Provide an explicit statement of the objective(s) or question(s) the review addresses. | 5 |
| **METHODS** | | | |
| Eligibility criteria | 5 | Specify the inclusion and exclusion criteria for the review and how studies were grouped for the syntheses. | 6 |
| Information sources | 6 | Specify all databases, registers, websites, organisations, reference lists and other sources searched or consulted to identify studies. Specify the date when each source was last searched or consulted. | 6 |
| Search strategy | 7 | Present the full search strategies for all databases, registers and websites, including any filters and limits used. | 6, Supplementary materials pages 21 and 22 |
| Selection process | 8 | Specify the methods used to decide whether a study met the inclusion criteria of the review, including how many reviewers screened each record and each report retrieved, whether they worked independently, and if applicable, details of automation tools used in the process. | 7 and 8 |
| Data collection process | 9 | Specify the methods used to collect data from reports, including how many reviewers collected data from each report, whether they worked independently, any processes for obtaining or confirming data from study investigators, and if applicable, details of automation tools used in the process. | 7 and 8 |
| Data items | 10a | List and define all outcomes for which data were sought. Specify whether all results that were compatible with each outcome domain in each study were sought (e.g. for all measures, time points, analyses), and if not, the methods used to decide which results to collect. | 8 |
| | 10b | List and define all other variables for which data were sought (e.g. participant and intervention characteristics, funding sources). Describe any assumptions made about any missing or unclear information. | 7 |
| Study risk of bias assessment | 11 | Specify the methods used to assess risk of bias in the included studies, including details of the tool(s) used, how many reviewers assessed each study and whether they worked independently, and if applicable, details of automation tools used in the process. | 7 and 8 |
| Effect measures | 12 | Specify for each outcome the effect measure(s) (e.g. risk ratio, mean difference) used in the synthesis or presentation of results. | 8 |
| Synthesis methods | 13a | Describe the processes used to decide which studies were eligible for each synthesis (e.g. tabulating the study intervention characteristics and comparing against the planned groups for each synthesis (item #5)). | 7 |
| | 13b | Describe any methods required to prepare the data for presentation or synthesis, such as handling of missing summary statistics, or data conversions. | 7 and 8 |
| | 13c | Describe any methods used to tabulate or visually display results of individual studies and syntheses. | 8 and 9 |
| | 13d | Describe any methods used to synthesize results and provide a rationale for the choice(s). If meta-analysis was performed, describe the model(s), method(s) to identify the presence and extent of statistical heterogeneity, and software package(s) used. | 8 and 9 |
| | 13e | Describe any methods used to explore possible causes of heterogeneity among study results (e.g. subgroup analysis, meta-regression). | 8 |

For peer review only

# PRISMA 2020 Checklist

| Section and Topic | Item # | Checklist item | Location where item is reported |
|---|---|---|---|
| | 13f | Describe any sensitivity analyses conducted to assess robustness of the synthesized results. | 8 and 9 |
| Reporting bias assessment | 14 | Describe any methods used to assess risk of bias due to missing results in a synthesis (arising from reporting biases). | 7 and 8 |
| Certainty assessment | 15 | Describe any methods used to assess certainty (or confidence) in the body of evidence for an outcome. | 8 and 9 |
| **RESULTS** | | | |
| Study selection | 16a | Describe the results of the search and selection process, from the number of records identified in the search to the number of studies included in the review, ideally using a flow diagram. | 10, Figure 1 and Supplementary Table 1 |
| | 16b | Cite studies that might appear to meet the inclusion criteria, but which were excluded, and explain why they were excluded. | Supplementary Table 1 |
| Study characteristics | 17 | Cite each included study and present its characteristics. | Supplementary Table 2 |
| Risk of bias in studies | 18 | Present assessments of risk of bias for each included study. | 10 and Figure 2 |
| Results of individual studies | 19 | For all outcomes, present, for each study: (a) summary statistics for each group (where appropriate) and (b) an effect estimate and its precision (e.g. confidence/credible interval), ideally using structured tables or plots. | 11, 12, 13 and Supplementary Table 5 |
| Results of syntheses | 20a | For each synthesis, briefly summarise the characteristics and risk of bias among contributing studies. | Supplementary Table 4 |
| | 20b | Present results of all statistical syntheses conducted. If meta-analysis was done, present for each the summary estimate and its precision (e.g. confidence/credible interval) and measures of statistical heterogeneity. If comparing groups, describe the direction of the effect. | 11,12,13 and 14 |
| | 20c | Present results of all investigations of possible causes of heterogeneity among study results. | 10 and 11 |
| | 20d | Present results of all sensitivity analyses conducted to assess the robustness of the synthesized results. | 11,12,13 and 14 |
| Reporting biases | 21 | Present assessments of risk of bias due to missing results (arising from reporting biases) for each synthesis assessed. | 10, Figure 2 and Supplementary table 4 |
| Certainty of evidence | 22 | Present assessments of certainty (or confidence) in the body of evidence for each outcome assessed. | 11, 12, 13 and 14 |
| **DISCUSSION** | | | |
| Discussion | 23a | Provide a general interpretation of the results in the context of other evidence. | 17 |
| | 23b | Discuss any limitations of the evidence included in the review. | 16 |
| | 23c | Discuss any limitations of the review processes used. | 16 and 17 |
| | 23d | Discuss implications of the results for practice, policy, and future research. | 17 |
| **OTHER INFORMATION** | | | |

For peer review only
For peer review only - http://bmjopen.bmj.com/site/about/guidelines.xhtml

# PRISMA 2020 Checklist

| Section and Topic | Item # | Checklist item | Location where item is reported |
|---|---|---|---|
| Registration and protocol | 24a | Provide registration information for the review, including register name and registration number, or state that the review was not registered. | 21 |
| | 24b | Indicate where the review protocol can be accessed, or state that a protocol was not prepared. | 6 |
| | 24c | Describe and explain any amendments to information provided at registration or in the protocol. | 6 |
| Support | 25 | Describe sources of financial or non-financial support for the review, and the role of the funders or sponsors in the review. | 21 |
| Competing interests | 26 | Declare any competing interests of review authors. | 21 |
| Availability of data, code and other materials | 27 | Report which of the following are publicly available and where they can be found: template data collection forms; data extracted from included studies; data used for all analyses; analytic code; any other materials used in the review. | Available in supplementary materials |

*From:* Page MJ, McKenzie JE, Bossuyt PM, Boutron I, Hoffmann TC, Mulrow CD, et al. The PRISMA 2020 statement: an updated guideline for reporting systematic reviews. BMJ 2021;372:n71. doi: 10.1136/bmj.n71

For more information, visit: http://www.prisma-statement.org/

For peer review only

BMJ Open

# Diagnostic accuracy of blood tests of inflammation in paediatric appendicitis – a systematic review and meta-analysis

| | |
|---|---|
| Journal: | *BMJ Open* |
| Manuscript ID | bmjopen-2021-056854.R2 |
| Article Type: | Original research |
| Date Submitted by the Author: | 05-Sep-2022 |
| Complete List of Authors: | Fawkner-Corbett, David; Weatherall Institute of Molecular Medicine, ; University of Oxford Nuffield Department of Surgical Sciences, Hayward, Gail; University of Oxford, Nuffield Department of Primary Care Health Sciences Alkhmees, Mohammed; University of Groningen, Department of General Practice and Elderly Care Medicine Van Den Bruel, Ann; KU Leuven Ordóñez-Mena, JM; University of Oxford, Nuffield Department of Primary Care Health Sciences Holtman, G.; University Medical Centre Groningen, Department of General Practice and Elderly Care Medicine |
| <b>Primary Subject Heading</b>: | Surgery |
| Secondary Subject Heading: | Surgery, Paediatrics, Evidence based practice |
| Keywords: | PAEDIATRIC SURGERY, PAEDIATRICS, ACCIDENT & EMERGENCY MEDICINE |
| | |

SCHOLARONE™
Manuscripts

*I, the Submitting Author has the right to grant and does grant on behalf of all authors of the Work (as defined in the below author licence), an exclusive licence and/or a non-exclusive licence for contributions from authors who are: i) UK Crown employees; ii) where BMJ has agreed a CC-BY licence shall apply, and/or iii) in accordance with the terms applicable for US Federal Government officers or employees acting as part of their official duties; on a worldwide, perpetual, irrevocable, royalty-free basis to BMJ Publishing Group Ltd ("BMJ") its licensees and where the relevant Journal is co-owned by BMJ to the co-owners of the Journal, to publish the Work in this journal and any other BMJ products and to exploit all rights, as set out in our licence.*

*The Submitting Author accepts and understands that any supply made under these terms is made by BMJ to the Submitting Author unless you are acting as an employee on behalf of your employer or a postgraduate student of an affiliated institution which is paying any applicable article publishing charge ("APC") for Open Access articles. Where the Submitting Author wishes to make the Work available on an Open Access basis (and intends to pay the relevant APC), the terms of reuse of such Open Access shall be governed by a Creative Commons licence – details of these licences and which Creative Commons licence will apply to this Work are set out in our licence referred to above.*

*Other than as permitted in any relevant BMJ Author's Self Archiving Policies, I confirm this Work has not been accepted for publication elsewhere, is not being considered for publication elsewhere and does not duplicate material already published. I confirm all authors consent to publication of this Work and authorise the granting of this licence.*

For peer review only

**Title:**

Diagnostic accuracy of blood tests of inflammation in paediatric appendicitis – a systematic review and meta-analysis

**Authors names:**

David Fawkner-Corbett (0000-0002-5273-3682), Gail N Hayward (0000-0003-0852-627X), Mohammed Alkhmees (0000-0002-8608-6938), Ann Van den Bruel (000-0001-9012-2009), Jose M Ordóñez-Mena (0000-0002-8965-103X), Gea A Holtman (0000-0001-6579-767X)

**Address and positions for each author:**

NIHR Community Healthcare MedTech and IVD Co-operative, Nuffield Department of Primary Care Health Sciences, University of Oxford, Oxford OX2 6GG, United Kingdom; Academic Paediatric Surgery Unit, Nuffield Department of Surgical Sciences, University of Oxford, OX3 9DU, Oxford, United Kingdom; MRC Weatherall Institute of Molecular Medicine, Radcliffe Department of Medicine, University of Oxford, OX3 9DS, Oxford United Kingdom; David Fawkner-Corbett, Paediatric surgical trainee

NIHR Community Healthcare MedTech and IVD Co-operative, Nuffield Department of Primary Care Health Sciences, University of Oxford, Oxford OX2 6GG, United Kingdom; Gail N Hayward, Associate professor

Department of General Practice and Elderly Care Medicine, University of Groningen, University Medical Center Groningen, PO Box 196, 9700 AD, Groningen, Netherlands, Mohammed Alkhmees, medical student

NIHR Community Healthcare MedTech and IVD Co-operative, Nuffield Department of Primary Care Health Sciences, University of Oxford, Oxford OX2 6GG, United Kingdom; NIHR Oxford Biomedical Research Centre, Oxford University Hospitals NHS Foundation Trust, Oxford, OX3 9DU, Oxford, United Kingdom, Jose M Ordóñez-Mena, medical statistician

EPI-Centre, Academic Centre for Primary Care, Department of Public Health and Primary Care, KU Leuven, Bus 7001, 3000 Leuven, Belgium; Ann Van den Bruel, Associate professor

NIHR Community Healthcare MedTech and IVD Co-operative, Nuffield Department of Primary Care Health Sciences, University of Oxford, Oxford OX2 6GG, United Kingdom; Department of General Practice and Elderly Care Medicine, University of Groningen, University Medical Center Groningen, PO Box 196, 9700 AD, Groningen, Netherlands, Gea A Holtman, assistant professor

Correspondence to: David Fawkner-Corbett (David.fawkner-corbett@imm.ox.ac.uk)

NIHR Community Healthcare MedTech and IVD Co-operative, Nuffield Department of Primary Care Health Sciences, University of Oxford, Oxford OX2 6GG, United Kingdom

**Word count:** 4539

**Figures:** Three main figures, eight supplementary

**Tables:** Two main tables, five supplementary



For peer review only - http://bmjopen.bmj.com/site/about/guidelines.xhtml

## Abstract

**Objective:** Possible childhood appendicitis is a common emergency presentation. The exact value of blood tests is debated. This study sought to determine the diagnostic accuracy of four blood tests (White cell count[WCC], neutrophil[count or percentage], C-reactive protein[CRP] and/or procalcitonin) for childhood appendicitis.

**Design:** Systematic review and diagnostic meta-analysis.

**Data sources:** MEDLINE, EMBASE, Central, Web of Science searched from inception-March 2022 with reference searching and authors contacted for missing/unclear data.

**Eligibility criteria for study selection:** Studies reported the diagnostic accuracy of the four blood tests compared to the reference standard(histology or follow-up). Risk of bias was assessed(QUADAS-2), pooled sensitivity and specificity were generated for each test and commonly presented cut-offs. To provide insight into clinical impact, we present strategies using a hypothetical cohort.

**Results:** 67 studies were included (34,839 children, 13,342 with appendicitis), all in the hospital setting. The most sensitive tests were WCC(≥10,000 cells/µl, 53 studies sensitivity 0·85[95% CI 0·80-0·89]) and absolute neutrophil count [ANC](≥7,500 cells/µl, five studies sensitivity 0·90[95% CI 0·85-0·94]). Combination of WCC or CRP increased sensitivity further(≥10,000 cells/µl or ≥10mg/l, individual patient data(IPD) of 6 studies, 0.97[95% CI 0.93-0.99]).
Applying results to a hypothetical cohort(1000 children with appendicitis symptoms, of whom 400 have appendicitis) 60 and 40 children would be wrongly discharged based solely on WCC and ANC respectively; 12 with combination of WCC or CRP.
The most specific tests were CRP alone(≥50mg/l, 38 studies, specificity 0·87[95% CI 0·80-0·91]) or combined with WCC(≥10,000 cells/µl and ≥50mg/l, IPD of six studies, 0·93[95% CI 0·91-0·95]).

**Conclusions:** The best performing single blood tests for ruling-out paediatric appendicitis are WCC or ANC; with accuracy improved combining WCC and CRP. These tests could be used at the point of care in combination with clinical prediction rules. We provide insight into the best cut-offs for clinical application.

**Systematic review registration:** PROSPERO-CRD42017080036



For peer review only - http://bmjopen.bmj.com/site/about/guidelines.xhtml

**Strengths and limitations**

- This systematic review and meta-analysis looked at the accuracy of the four most commonly used blood tests for diagnosing appendicitis against a reference standard of histology/surgical diagnosis or follow-up.
- An extensive literature search for all studies reporting children presenting with symptoms of appendicitis reporting metrics for white cell count, neutrophil (count or percentage), C-Reactive protein and/or procalcitonin were included.
- Authors were contacted when data was unclear, generating a pool of individual patient data to allow use of new methods and information about the cut offs that perform best for clinical application for all tests.
- The literature is limited for application of results in primary care or as point of care tests, as no studies reported diagnostic accuracy in these settings.
- Results were applied to a hypothetical cohort to aid clinical interpretation.

For peer review only


For peer review only - http://bmjopen.bmj.com/site/about/guidelines.xhtml

## Introduction

Abdominal pain is a common reason for a child to attend the emergency department (ED)[1]. Differentiation between self-limiting and surgical conditions, such as acute appendicitis, can be especially difficult in children where less than 50% of patients present with classically described symptoms[2–4]. Acute appendicitis is the most common surgical condition with over 12,000 children undergoing emergency surgery for the condition each year in England[5]. It is vital to prevent an unnecessary operation in children, which is reported in 5% to 20% of cases[6–9], whilst also undertaking prompt treatment and avoiding inappropriate discharge to mitigate the risks of more severe advanced disease including perforation[10,11]. Radiological investigations including computed tomography (CT) imaging, magnetic resonance imaging (MRI) or ultrasound are useful adjunct to decide who needs surgery[7,12]. However, these modalities will often require admission and routine use in all suspected cases would increase the burden on the health-care system and children[13].

Blood tests are frequently used to investigate children with suspected appendicitis to help inform diagnostic decisions; with White cell count (WCC), neutrophils (as count or percentage) and C-reactive protein (CRP) being most widely reported[14,15]. Procalcitonin is also increasingly used to distinguish paediatric sepsis[16]. Much of the evidence for their accuracy in this setting is applied from adult studies[17,18]. All these blood tests can be available within hours and are also increasingly available rapidly as point-of-care (POC) tests thus extending their future application to expediate the clinical decision making process and broaden their use into community care[19].

Clinical scoring tools, which can incorporate clinical signs and blood results, can be used to triage patients at hospital to determine the need for imaging or surgery[20]. Although the clinical prediction scores are recommended their reported accuracy and uptake varies widely, and use is more limited in children[21]. Additionally a core scoring criteria of each is blood tests with the type of test and cut-off varying, reflecting the fact the evidence of optimum diagnostic cut off is scarce[13,22].

Taken together, blood tests are widely performed but exact accuracy for informing the diagnosis of paediatric appendicitis is debated[23]. More insight in the diagnostic accuracy of several cut offs of blood tests would help to choose which POC tests should be prioritised


For peer review only - http://bmjopen.bmj.com/site/about/guidelines.xhtml

for implementation in the emergency department, which should best inform admission or discharge decisions, and which cut-offs are optimum to integrate with future optimisation of clinical scoring tools. To date a large, systematic appraisal of the diagnostic accuracy of these tests in paediatric appendicitis is lacking and so this study aims to undertake a systematic review and meta-analysis of all studies reporting the diagnostic accuracy of common blood tests in the diagnosis of paediatric appendicitis.

For peer review only


For peer review only - http://bmjopen.bmj.com/site/about/guidelines.xhtml

## Methods

This systematic review and meta-analysis was undertaken in line with the Preferred Reporting Items for Systematic Reviews and Meta-Analyses (PRISMA) guidelines[24] and was prospectively registered (PROSPERO: CRD42017080036).

**Public and patient involvement:**

The concept of this review involved discussion with patients and families before it was undertaken and discussed experience in paediatric blood tests, identifying it as important to know the exact value (Precision antimicrobial prescribing patient and public involvement group, University of Oxford). The results of this manuscript were also presented in this forum which helped formulate the write up and key discussion points within the final manuscript.

**Search Strategy:**

Searching of databases (Cochrane Database of Systematic Reviews, Cochrane Central Register of controlled trials, Database of abstracts of reviews of effects(DARE), Embase, Ovid(Medline) Epub Ahead of print in process and other Non-Indexed Citations, Ovid Medline(R) Daily, Ovid Medline(R) and Science Citation Index and Conference Proceedings Citation Index (SCIENCE)) included key word combinations to identify children with symptoms suggestive of appendicitis having one of the four blood tests (WCC, neutrophil, CRP, procalcitonin) for diagnosis. Searching was performed on 15$^{th}$ March 2022 to include all relevant studies from database inception with no language or date limitations. Full search strategy is provided in **Supplementary Materials**. Hand searching of included studies references was undertaken. Assessment of foreign language studies was completed by a native language speaker.

**Study Selection and Data Extraction:**

Included studies reported children (<18 years) presenting with symptoms suggestive of appendicitis that would have testing of WCC, neutrophil, CRP and/or procalcitonin, by laboratory or POC, to distinguish appendicitis from non-surgical cause.


For peer review only - http://bmjopen.bmj.com/site/about/guidelines.xhtml

For studies that were included the optimal reference standard for confirming diagnosis of appendicitis was decided as histology after surgery (ideal standard) or; surgery alone, imaging confirming appendicitis in non-operatively managed patients or surgical diagnosis in studies without histological reporting. In patients without surgery (non-appendicitis) the reference standard was follow-up after initial assessment to rule out appendicitis with an ideal standard of ≥14 days with active follow up. Studies that differed or were unclear about confirming these standards would be highlighted when assessing risk of bias.

Studies were included if they matched these pre-defined criteria and reported sufficient data to construct or derive a two-by-two contingency table for any/multiple blood tests. Studies were excluded if they only reported surgical cases, had <20 participants, reported atypical appendiceal pathology (e.g. carcinoid, appendiceal mass) or if the proportion of participants outside the age range was >15%. Where multiple studies reported data on the same population, only outcomes of the earliest study were used, unless the authors confirmed individual patient results.

Screening of titles and abstracts was performed independently by two reviewers (DFC and GAH). Full text articles of all short-listed studies were then assessed for eligibility.

Four reviewers (DFC, GAH, JMOM, MA) independently extracted data from each included study on a pre-designed data extraction sheet including information on demographics, prevalence, reference standards, measurement of blood tests including cut-offs, and 2x2 tables. Disagreements were resolved by consensus or from input of an independent reviewer (GNH).

In studies which matched inclusion criteria exclusion with reason would occur if: (a) data was not able to be extracted in 2x2 format (derivation from reported sensitivity / specificity or other metrics would be used if possible); (b) a paediatric sub-group was not adequately detailed; (c) exclusion criteria were unclear; (d) the paper was only available in an abstract form. If reason (a) was given for full-paper exclusion an attempt would be made to contact an author on up to three occasions before exclusion. If contacted authors provided individual patient data, this was included.

**Assessment of study methodological quality:**

All included studies were assessed by four reviewers (DFC, GAH, JMOM, MA) independently for risk of bias and applicability using the QUADAS-2 tool[25]. Discrepancies were resolved by



For peer review only - http://bmjopen.bmj.com/site/about/guidelines.xhtml

discussion or review from a third independent reviewer (GNH) to achieve consensus. Full details of QUADAS-2 scoring criteria are detailed in **Supplementary Materials: QUADAS-2**. Overview graph of QUADAS-2 outcomes was created using GraphPad Prism (V 8.0, GraphPad Software Inc).

**Data synthesis and statistical analysis:**

We extracted binary data for all appropriate measurements of diagnostic accuracy for the pre-defined blood tests. Cut-offs were defined by authors in each individual publication. In cases where individual data was provided by authors a 2x2 table was calculated using the most commonly reported cut-offs. Data for age-dependent cut-offs would be included in study overviews and forest plots but not in cut-off specific meta-analysis. Measurement of neutrophils by means of absolute count (ANC) or percentage of white cells was analysed separately.

Test accuracy overviews for each marker were reported as forest plots including 2x2 tables, sensitivity and specificity with 95% confidence intervals using RevMan (Cochrane collaboration, Copenhagen, Denmark). Meta-analysis was performed using a bivariate random effects model and pooled estimates of sensitivity, specificity and likelihood ratios were generated when >4 studies per marker was included using the *metandi* module in STATA version 16[26–28]. When a study provided multiple cut-offs for each test, we calculated the summary receiver operating characteristics (SROC) curve with 95% CI using R *diagmeta* package developed by Steinhauser et al[29] assuming a fixed intercept (diagnostic accuracy) and slope (threshold effect) across studies. In addition, we calculated the pooled sensitivity and specificity for commonly presented cut-offs and we compared the results with the bivariate random effects model of cut-off groups of these tests.

We assessed the heterogeneity by visual inspection of the forest plot and SROC plot. Potential differences in studies with: high risk of bias in patient selection; case-control design; from primary care; with low prevalence (<10%); with high prevalence (≥60%), were evaluated by adding these covariates to the bivariate model and performing sensitivity and sub-group analysis to measure if these factors could explain heterogeneity. To assess for publication bias we used the Deeks' test[30].

**Potential clinical impact:**


For peer review only - http://bmjopen.bmj.com/site/about/guidelines.xhtml

To provide more insight into the clinical consequences of using the results of each test, for each measurement with pooled sensitivity and specificity hypothetical 2×2 tables were constructed in 1000 children presenting with symptoms suggestive of acute appendicitis. The appendicitis prevalence was modelled on the median prevalence in the cohort studies included in the final meta-analysis, rounded to the nearest 10% to help facilitate interpretation. Standardisation of prevalence allowed comparison of the results of each test.

To illustrate the clinical application of tests we interpreted these as follows: children with appendicitis missed are those with appendicitis and a negative index test result (false negatives); the numbers of unnecessary surgeries are the children without appendicitis with a positive index test result (false positives).

For peer review only


For peer review only - http://bmjopen.bmj.com/site/about/guidelines.xhtml

## Results

### Search Strategy and study selection

The search strategy, reference screening and paediatric data provided by contacted authors identified 3,211 studies. 67 studies matched inclusion criteria and were included in final analysis, of which 11 studies had patient data provided after contacting authors to clarify criteria or provide paediatric-only data[31–41]. Remaining papers were excluded with reason (**Figure 1, Supplementary Table 1**).

### Study Characteristics

All included studies were in the hospital or emergency department setting. The most common study designs were cohort study, with either a prospective (n=40, 60%) or retrospective (n=11, 16%) cohort design, or case control (n=6, 9%) (**Table 1, Supplementary Table 2**). One study had a low prevalence (<10%) of appendicitis[42]. Seven cohort studies had a high prevalence (>60%) due to a more select setting such as GP referrals[43,44], hospitalised [39,43,45,46] or ITU based patients[41]. In one study the reason for high prevalence was unclear[47]. The median prevalence for all studies was 44% (IQR 34-58%). No study used POC blood test devices.

The most frequent reference standard in appendicitis patients was histopathology with the remainder using operative reports (**Table 1, Supplementary Table 3**). Of 67 studies, 61 (91%) described inclusion of a population of children with clinical symptoms of appendicitis and eight of these studies specified a blood test and/or ultrasound for inclusion; the remaining 6 studies (9%) were of case-control designs and most commonly identified cases through surgery and controls as those with symptoms of appendicitis which had the condition ruled out (**Supplementary Table 2**).

For peer review only


For peer review only - http://bmjopen.bmj.com/site/about/guidelines.xhtml

| | | Studies (n=67) |
|---|---|---|
| **Study design** | Prospective cohort study | 40 (60%) |
| | Retrospective cohort study | 11 (16%) |
| | Case control study | 6 (9%) |
| | Prospective observational study | 3 (4%) |
| | Retrospective case series | 2 (3%) |
| | Other* | 5 (7%) |
| **Setting** | **Institution**<br>Hospital | 67 (100%) |
| | **Department**†<br>Emergency department<br>Surgical department | 49 (73%)<br>17 (25%) |
| | **Centre**<br>Single centre<br>Multiple centre | 54 (81%)<br>13 (19%) |
| **Study participants** | Total (n) | 34,839 ‡ |
| | Median patients included (range) | 275 (44-3791) |
| | ≤50 participants | 4 (6%) |
| | 51-250 participants | 28 (42%) |
| | 251-500 participants | 14 (21%) |
| | >500 participants | 21 (31%) |
| **Year Published** | Before 1989 | 2 (3%) |
| | 1990-1999 | 5 (7%) |
| | 2000-2009 | 14 (21%) |
| | 2010-2022 | 46 (69%) |
| **AA Prevalence** | <20% | 6 (9%) |
| | 21-35% | 14 (21%) |
| | 36-50% | 24 (36%) |
| | 51-65% | 18 (27%) |
| | >65% | 5 (7%) |
| **Gender demographics(% Male)** | <35% | 1 (1%) |
| | 36-45% | 8 (12%) |
| | 46-55% | 29 (43%) |
| | 56-65% | 20 (30%) |
| | >65% | 2 (3%) |
| | Unclear | 7 (10%) |
| **Reference standard (AA)** | Histology/ Pathology | 63 (94%) |
| | Operative findings | 4 (6%) |
| **Reference Standard (non-AA)** | Active follow up | 19 (28%) |
| | Discharge diagnosis | 16 (24%) |
| | Unspecified follow up | 8 (12%) |
| | Observation | 8 (12%) |
| | Unclear | 8 (12%) |
| | Failure to reattend | 6 (9%) |
| | Case note review | 2 (3%) |
| **Index test(s) measured** | White cell count (WCC) | 59 (88%) |
| | C-reactive protein (CRP) | 38 (57%) |
| | Absolute neutrophil count | 25 (37%) |
| | Neutrophil percentage | 16 (24%) |
| | WCC and CRP | 13 (19%) |
| | WCC, CRP and neutrophil | 6 (9%) |
| | Procalcitonin | 6 (9%) |
| | Neutrophil : lymphocyte ratio | 3 (4%) |
| | WCC and Neutrophil count | 1 (1%) |

For peer review only


For peer review only - http://bmjopen.bmj.com/site/about/guidelines.xhtml

**Table 1**: **Pooled overview of design and standards of included studies**

(*In 5 studies authors reported other study designs than those listed. † In one study (1%) department setting was unclear. ‡ value includes all participants in included studies, in 5 studies a paediatric sub-group was provided meaning total paediatric patients in analysis n=31,143. Full details of all study characteristics including design, setting, population characteristics and demographics, exclusion and inclusion criteria is fully detailed in **Supplementary Table 2.** Full details of study reference standard, index tests and cut offs in **Supplementary Table 3**)

For peer review only


For peer review only - http://bmjopen.bmj.com/site/about/guidelines.xhtml

## Risk of Bias

Applying the QUADAS-2 framework[25] patient selection in 60 studies (90%) were low risk of concern for applicability of results as they clearly included patients with suspected acute appendicitis. Those not scoring low included higher ages (n=4)[36,48-50] or only those undergoing ultrasound scans (8·3%, n=5)[51-55]. However fewer studies scored low for risk of bias (44%, n=30) when considering patient selection; most commonly because studies did not clearly state whether they recruited a random or fully consecutive population (**Figure 2, Supplementary table 4)**.

Risk of bias and applicability of the index test and reference standard was often low risk or unclear - owing to a lack of detail regarding blinding (i.e. clinician or pathologist) or follow-up method. No study scored low for "flow and timing" as a component as this required both groups to receive the same reference standard. It is understandably not ethical to perform surgery in all children, and therefore in this dimension the seven studies that score "unclear" due to lack of detailed outcomes for all participants are likely of greater concern (**Figure 2**).

## Blood Tests

The 67 included studies consisted of 34,839 children (13,342 with appendicitis and 21,497 without) which evaluated the following tests: WCC, CRP, ANC, neutrophils as percentage of leukocytes, procalcitonin, combined WCC and CRP, and combined WCC/CRP/neutrophil percentage. Neutrophil to lymphocyte ratio (NLR) was reported in three studies which was not sufficient to allow a meta-analysis. Reported accuracies ranged from sensitivity 0.18-0.91, and specificity 0.69-0.95 but cut-offs used were dissimilar[56-58]. Similarly, the combination of WCC and ANC (>13,300/µl and >10,800/µl respectively) was reported in one study (sensitivity = 0.76, specificity =0.82)[59]. We found no evidence of publication bias for each test where this was appropriate to assess (**Supplementary Figure 1**).

An overall pooled estimate for each test, at different cut-offs, is shown in **Table 2**. For WCC and CRP the pooled estimates obtained for each cut-off with the Steinhauser method were similar for the cut-off groups using the bivariate model (**Supplementary table 5**). In subgroup analyses, the test characteristics did not differ between studies with a high

For peer review only


For peer review only - http://bmjopen.bmj.com/site/about/guidelines.xhtml

appendicitis prevalence (≥60%) and lower prevalence. In some tests when using bivariate analysis the study design (for WCC 10-15,000 cells/μl and neutrophils) or patient selection (for WCC and CRP) had an effect on the sensitivity or specificity which we quantified (**Supplementary table 5**).

For peer review only


For peer review only - http://bmjopen.bmj.com/site/about/guidelines.xhtml

| Marker | Studies | Threshold | Pooled sensitivity* (95% CI) | Pooled specificity* (95% CI) | Hypothetical cohort of 1000 children with appendicitis prevalence of 40%[†] | | Hypothetical cohort of 1000 children with appendicitis prevalence of 5% | |
|---|---|---|---|---|---|---|---|---|
| | | | | | Missed appendicitis (FN) | Unnecessary treatment (FP) | Missed appendicitis (FN) | Unnecessary treatment (FP) |
| WCC[‡] (Cells/µl) | 53 | 10,000 | 0·85 (0·80 – 0·89) | 0·58 (0·51 – 0·64) | 60 | 252 | 8 | 399 |
| | | 12,000 | 0·73 (0·66 – 0·79) | 0·71 (0·65 – 0·76) | 108 | 174 | 14 | 276 |
| | | 15,000 | 0·53 (0·45 – 0·62) | 0·83 (0·78 – 0·87) | 188 | 102 | 24 | 162 |
| CRP[§] (mg/L) | 38 | 5 | 0·77 (0·69 – 0·84) | 0·54 (0·45 – 0·63) | 92 | 276 | 12 | 437 |
| | | 10 | 0·68 (0·59 – 0·76) | 0·66 (0·58 – 0·73) | 128 | 204 | 16 | 323 |
| | | 30 | 0·50 (0·39 – 0·60) | 0·81 (0·75 – 0·87) | 200 | 114 | 25 | 181 |
| | | 50 | 0·41 (0·30 – 0·53) | 0·87(0·80 – 0·91) | 276 | 78 | 35 | 124 |
| Neutrophils[¶] (% of lymphocytes) | 12 | 75% | 0·76 (0·73-0·80) | 0·63 (0·57-0·69) | 96 | 222 | 12 | 352 |
| ANC(Cells/µl) | 5 | <7,500 | 0·90 (0·85 – 0·94) | 0·54 (0·49 – 0·58) | 40 | 276 | 5 | 437 |
| | 13 | 7,500 | 0·86 (0·82 – 0·89) | 0·60 (0·54 – 0·66) | 56 | 240 | 7 | 380 |
| | 6 | >7,500 | 0·70 (0·65 – 0·75) | 0·78 (0·71 – 0·85) | 120 | 132 | 15 | 209 |
| Procalcitonin (ng/ml) | 6 | 0·1 – 0·5 | 0·30 (0·26 – 0·35) | 0·83 (0·73 – 0·89) | 280 | 132 | 35 | 209 |
| WCC and CRP[‡] | 6 IPD studies | 10,000 and 10 | 0·59 (0·49-0·68) | 0·79 (0·74-0·84) | 164 | 126 | 21 | 200 |
| WCC and CRP | 6 IPD studies | 10,000 and 50 | 0·27 (0·19-0·37) | 0·93(0·91-0·95) | 292 | 46 | 37 | 67 |
| WCC or CRP | 6 IPD studies | 10,000 and 10 | 0.97 (0.93 – 0.99) | 0.40 (0.30 – 0.51) | 12 | 360 | 2 | 570 |
| WCC or CRP | 6 IPD studies | 10,000 and 50 | 0.91 (0.86 – 0.95) | 0.50 (0.40 – 0.60) | 36 | 300 | 5 | 475 |

**Table 2.** Pooled estimates of diagnostic accuracy of makers for diagnosis of appendicitis in children (*All pooled sensitivity and specificity were obtained from the bivariate model, except for WCC and CRP for which we calculated sensitivity and specificity for selected cut offs using the Steinhauser method, comparison with bivariate mode including Likelihood ratios for all tests is demonstrated in **Supplementary Table 5**. [†]Median prevalence of appendicitis in the included 62 cohort studies is 40% [‡]WCC was evaluated in 59 studies whereof 53 were included in the meta-analysis whereof 12 provided multiple cut offs. Six studies could not be used in the pooled analysis, because 5 studies reported WCC provided age-dependent cut offs and 1 study reported pain duration dependent cut offs. [§]CRP was evaluated in 39 studies whereof 11 provided multiple cut offs. [¶]Neutrophils evaluated in 16 studies of which 12 provided cut off at 75%. [‡]WCC and CRP calculated from individual patient data from 6 studies (n=1541 participants) and analysed using the bivariate model)

For peer review only



For peer review only - http://bmjopen.bmj.com/site/about/guidelines.xhtml

*WCC*

WCC was the most frequently reported blood test (59 studies, including 22,779 patients; 9,733 with appendicitis). At a cut-off of 10,000 cell/µl WCC exhibited one of the highest pooled sensitivities of any individual test (0.85, 95% CI 0.80-89, 53 studies) but specificity was low (0.58, 95% CI 0.51-0.64] **Table 2, Figure 3A, Supplementary Figure 2**).

*CRP*

CRP was the second most frequently reported test (38 studies, 9934 patients [4201 appendicitis]. The sensitivity and specificity for lower cut-off values varied considerably between studies on visual inspection (**Supplementary Figure 3**). Specificity of a CRP value of ≥50 mg/L was more homogenous. The highest pooled sensitivity of CRP was at a cut-off of ≤5mg/L (0.77 (95% CI 0.69 – 0.84)), although specificity was low (0.54, 95% CI 0.45 – 0.63**, Table 2**). Use of a cut-off ≥50mg/L had the best specificity of any single test at 0.87 (95% CI 0.80-0.91, **Table 2** and **Figure 3B**) and all studies reported a specificity of >0.8 for this (**Supplementary Figure 3**).

*Neutrophil measurement*

Neutrophil measurement was reported as an absolute count (ANC) in 25 studies, including 12,982 patients (5311 appendicitis). ANC was more frequently reported and demonstrated the highest sensitivity of any test at a low cut-off (≥7,500 cells/µl, 0.90, 95% CI 0.85-0.94) **Table 2, Supplementary Figure 4**). However, none of the studies evaluating ANC reported multiple cut-offs and therefore the SROC curve could not be calculated using the Steinhauser method. Relatively more studies (n=13) reported a cut-off of 7,500 cells/µl which reported a similar sensitivity (0.86 (95% CI 0.82-0.89), **Table 2**) to WCC.

Fewer studies (n=16, 5884 patients; 2275 appendicitis) reported neutrophil count as a percentage (**Supplementary Figure 5, Supplementary Table 5**), the most common cut-off reported was 75% and pooled (n=9) analysis at this level showed the sensitivity to be lower than ANC and WCC (**Table 2**).

*Procalcitonin*


For peer review only - http://bmjopen.bmj.com/site/about/guidelines.xhtml

Procalcitonin accuracy was quantified in 6 studies (860 patients; 469 appendicitis) which led to pooled analysis being performed on varied cut-offs. Sensitivity was homogenous, while specificity varied between the studies (**Supplementary Figure 6**). Pooled procalcitonin had a relatively high pooled specificity (0.83, 95% CI 0.73-0.89) but a low sensitivity (0.30, 95% CI 0.26-0.35) (**Table 2**).

*Combinations of tests*

When considering combinations of tests all but one of the included studies reported as a combination of both tests being positive to define a positive test. The cut-offs did vary between studies and the studies were therefore too heterogeneous to perform a meta-analysis. A combination of CRP and WCC (11 studies, 2,497 patients, 1,008 appendicitis) demonstrated a sensitivity range of 0.34 to 0.80 and specificity range of 0.50 to 0.95 (**Supplementary Figure 7**). A combination of one test being positive (10 studies, 5,490 patients, 1,912 appendicitis) showed a sensitivity and specificity range of 0.85 to 1.00 and 0.07 to 58, respectively.

A combination of CRP, WCC and neutrophils (five studies, 1,410 patients, 506 appendicitis) showed a range of sensitivities (0.43-0.86) but specificity was generally high (0.81-0.91) and one study evaluated the *or* combination with a sensitivity of 1.00 (0.97-1.00) and specificity of 0.25 (95% CI 0.20-0.30) (**Supplementary Figure 8**).

To account for pooling of differing cut-offs in combinations of tests we performed a sub-group analysis of patients from six studies which provided individual patient data (IPD) on request for clarification[31–35,41]. This included the raw results for 1541 patients and showed that a combination of WCC >10,000 cell/μl and CRP>10mg/L has reasonable pooled specificity (0.79, 95% CI 0.74-0.84) which was increased when the threshold for CRP was raised to >50mg/L with WCC (0.93, 95% CI 0.91-0.95), with a resulting low sensitivity (0.27, 95% CI 0.19-0.37, **Table 2**). Using a combination of tests as either WCC or CRP being positive at a low cut off generated the best sensitivity of any measured investigation (WCC >10,000 cell/μl or CRP>10mg/L; 0.97, 95% CI 0.93-0.99, **Table 2**).

*Clinical impact*

Modelling the effect of each test upon a hypothetical cohort of 1,000 children with a 40% prevalence of appendicitis, which was the median prevalence of all included studies



For peer review only - http://bmjopen.bmj.com/site/about/guidelines.xhtml

rounded to the nearest decile (median 44%, IQR 33-58%), showed that the best single tests performing best for ruling out appendicitis were ANC <7,500 cells/µl and WCC ≥10,000cells/µl, as these only miss 40 and 60 of the 400 children with appendicitis respectively, but would capture a large number of non-appendicitis cases for further review of treatment – 276 and 252 out of 600 respectively (**Table 2, Supplementary Table 5**). Therefore, in clinical application these single tests would be the best for supporting a decision to discharge, or to not refer a patient for surgical review.

The best single test for ruling in appendicitis was a CRP value ≥50mg/L and so could be the most useful in deciding to rule in the diagnosis of appendicitis. Although in the hypothetical cohort 78 would still undergo unnecessary review or treatment (False positive) of the 202 that would meet this threshold. Also, 276 children with appendicitis would be missed (**Table 2**).

Using IPD identified a combination of WCC or CRP (WCC >10,000 cell/µl or CRP>10mg/L) to lead to the least cases of missed appendicitis (n=12). Similarly, a combination of WCC >10,000 cells/µl and CRP >50mg/L was more specific, application of this to inform surgical decision would result in the lowest number of unnecessary treatment (n=46) but only 150 patients would meet this threshold in the hypothetical cohort, 104 of which would have appendicitis (**Table 2**).

Although no included studies were in the community setting, the analysis was repeated with a lower prevalence (5%), which is reported as the prevalence in primary care[60]. This was used to reflect metrics for use of blood tests in a non-emergency department setting, or if it was used for a triage test to decide other diagnostic modalities such as ultrasound (**Table 2**).

For peer review only


For peer review only - http://bmjopen.bmj.com/site/about/guidelines.xhtml

## Discussion

In a pooled analysis of 31,143 children attending hospital with symptoms of appendicitis the most sensitive single blood tests for discriminating appendicitis from non-surgical conditions were WCC or ANC, and combining WCC with CRP increases this further. The most specific were CRP with or without WCC. However, if these were used as a sole decision-making tool for discharge in a hypothetical cohort of 1000 children with 40% appendicitis prevalence, even the most sensitive single test would result in 40 cases of missed appendicitis (ANC). Conversely, although a blood test alone would unlikely be used as the sole tool for surgical decision making, if a blood test was used to rule in the condition the most specific test would result in 78 unnecessary treatments (CRP). Therefore, all common inflammatory blood tests have limitations in the diagnosis of paediatric appendicitis which we accurately quantify. We highlight which tests should be valued most highly in more complex diagnostic pathways.

*Strengths*

To our knowledge, this is the largest systematic review of the diagnostic accuracy of commonly utilised blood tests to support diagnosis in children with suspected appendicitis. Through use of a broad search strategy we combined information from 67 studies and report pooled measurements for five common blood tests at multiple cut-offs and in combination. Our analysis used multiple models for quantification and also undertook analysis for potential sub-group and publication biases. Previous diagnostic accuracy meta-analysis methods have used diagnostic data for a single cut-off, and often the selected cut-off is the one maximizing both diagnostic accuracy parameters (sensitivity and specificity). In our methods we used commonly reported cut offs to identify those with the highest clinical impact. Thus, by using our approach, we ensure the pooled diagnostic accuracy parameters are not overestimated[29].

The study gained important additional data through contacting authors and used individual patient data where possible, adding to the applicability of results to clinical practice. It also aided us in reporting combination of tests, with cut-offs being variable in literature but allowing inclusion of over 1500 children to demonstrate the power of combined WCC and


For peer review only - http://bmjopen.bmj.com/site/about/guidelines.xhtml

CRP to maximise sensitivity if either are positive, or specificity if both are negative. The included studies had representation from a variety of continents and 69% were undertaken in the last twelve years adding to the applicability of results. In addition, 94% of the studies confirmed the diagnosis of acute appendicitis with histopathology results adding to the reliability of findings.

*Potential limitations*

An important limitation is that no studies were identified from the primary care settings, or reported results of POC devices. Our findings could be readily applicable to the emergency setting for use in triage, similar to other conditions[61]. Although our sensitivity analysis suggested that prevalence did not affect the diagnostic accuracy, these results should be interpreted with care as there was only one study with a very low prevalence (<10%)[42]. We repeated the cohort analysis in a lower prevalence setting (5%) to illustrate likely performance if blood tests were used in the community or as a triage test for imaging investigation, although metrics did reflect information from higher prevalence studies and differences in case-mix or severity in appendicitis could result in a different diagnostic accuracy.

Another possible limitation is that acute appendicitis can represent a spectrum of severity, some reports suggesting histological subtypes of appendicitis can result in differing laboratory results[62]. This differentiation can only be made post-operatively, but our approach was to evaluate test application to all cases of suspected appendicitis so that it best represented the diagnostic decision making when clinicians first encounter cases. A small number of studies (n=5 for WCC, n=1 for ANC, and n=1 for neutrophils) also divided tests thresholds by age. These were too few to pool and were heterogeneous, so these results were reported but not used in meta-analysis.

Additionally, pooled metrics were applied to a hypothetical cohort which aids in the application but would not wholly represent the clinical setting, where full decision making would be more complex and could utilise other diagnostic modalities such as imaging. Also, although these results give one of the largest pooled analysis of blood tests the strategy is not able to identify the added value of these to specific signs and symptoms, or as a triage to additional diagnostics.


For peer review only - http://bmjopen.bmj.com/site/about/guidelines.xhtml

*Comparison with literature*

Smaller systematic reviews including adults and children have reported different metrics for diagnostic accuracy, such as WCC sensitivity being 0.62-0.87[18,63,64], which is likely a result of pooling multiple cut-offs from studies. To mitigate this effect we have pooled by similar cut-off generating SROC curves for each or from IPD where possible. This should increase the reliability and applicability of results, and has highlighted that at a specific cut-off of WCC sensitivity was higher than previously reported (0.84 at 10,000cells/μl).

Other studies report combined clinical scoring tools for diagnosing appendicitis, with blood tests as one component. A recent review reported the best performing of these (the Shera score) at a low cut-off to miss 18 out of 539 cases of paediatric appendicitis if used[20]. The specificity of this scoring tool was lower than any of our measured blood tests however (44.3% 95% CI 41.4-47.2%). These tools have the advantage of incorporating clinical signs and symptoms, and our results complement these by quantifying the exact benefit of tests at different cut-offs.

Blood tests in paediatric appendicitis have also been reviewed as part of a systematic review of other diagnostic modalities, with WCC identified as sensitive at 10,000 cells/μl (0.88% 95% CI 87-90%) and CRP as specific (no pooled analysis)[65]. This did not include retrospective or non-ED studies, being limited to single studies at some cut offs. Our study seems to agree with this but included more studies and IPD where available which allowed reporting of different cut-offs and highlighted the benefit of test combinations.

*Clinical implications*

Applying the meta-analysis results to a hypothetical clinical cohort demonstrated that ANC, WCC and CRP are the tests with greatest clinical value. Although in practice clinicians may not rely on blood tests alone, it is still important to know ANC or WCC (alone or combined with CRP) at a low cut off would be the best tests to inform a decision to discharge. Using this strategy for triage would result in a relatively low number of missed appendicitis cases (60 and 40 out of the 400 cases in the modelled cohorts respectively). Combining WCC and CRP increases this further (12/400 missed). These values could be used to develop triage pathways, or equate risk to parents to self-observe. These results could provide the basis for application of selected blood tests in a prospective trial in the triage setting, or an evaluation of POC tests outside the emergency department[66].


For peer review only - http://bmjopen.bmj.com/site/about/guidelines.xhtml
For peer review only

CRP at a high cut off or combined with WCC was the most specific test but a significant proportion of children would still reach this threshold, and a large number of true cases would be missed (276 and 188 respectively). A recent international cohort study of over 1800 children with right iliac fossa pain, identified a negative appendicectomy rate of 15.9%, increasing to 22.4% in girls aged 11-15 years[20]. Our results suggest using those blood tests to inform surgery would perform worse than this, highlighting blood tests alone should not be used to rule in appendicitis.

Application of these results, especially in the situation of combined blood tests in addition to signs and symptoms, would leave a proportion of children between the two clinical situations of a positive low WCC/ANC, and high CRP. In these children where diagnostic uncertainty remains, further assessment by specialist or use of an adjunct such as ultrasound could be considered. Using specific tests in combination with prediction rules to determine the need for investigations such as ultrasound could also lead to an increased diagnostic accuracy of imaging, being higher when the pre-test probability increases[67].

Our results would not support using procalcitonin or neutrophil percentage as a preferred test for clinical decision making[68].

*Conclusion*

In conclusion, we report the largest study of commonly available blood tests and identify WCC value below 10,000 cells/µl and ANC value below 7,500 cells/µl are the best single tests in ruling-out suspected childhood appendicitis. Combining WCC and CRP (10,000 cells/µl or 10mg/l) increased this further. These tests could be used in addition to signs and symptoms to discharge or safety-net patients. A CRP value above 50mg/l in addition to signs and symptoms could be used to select patients for further imaging. However, more information about the exact added value of these blood tests incorporated with other diagnostic modalities is needed. Since we did not find any primary care studies or studies evaluating POC tests, these three tests could be prioritised for future evaluation.

For peer review only


For peer review only - http://bmjopen.bmj.com/site/about/guidelines.xhtml

**Figure Legends:**

**Figure 1:** PRISMA flow chart for identification of included studies (*some studies excluded for 2 reasons, see full overview of excluded studies in **Supplementary table 1**)[24]

**Figure 2:** Overview of QUADAS-2 assessment of all included studies (n=67) showing review authors' assessment of risk of bias and study applicability in each domain reported as a percentage of all included studies (proportion coloured by assessment as high [red], unclear [yellow] or low [green], full outline of all individual components of QUADAS-2 is detailed in **Supplementary table 4**).

**Figure 3:** Summary receiver operating characteristic plot of WCC **(A)** and CRP **(B)** for diagnosis of paediatric acute appendicitis (Colour of dot representing study identified in legend, hashed grey box = 95% confidence interval specificity, solid grey box = 95% confidence interval sensitivity. Examples of commonly reported cut-offs shown in **(A)** as blue [10,000/µl], green [12,000/ µl] and red [15,000/ µl] crosses on curve; in **(B)** as yellow [5ml/L], pink [10mg/L], blue [20mg/L], green [30mg/L] and red [50mg/L] crosses on curve)

For peer review only


For peer review only - http://bmjopen.bmj.com/site/about/guidelines.xhtml

**Contributorship statement:** DFC, GNH, AvdB and GAH conceived and designed the study. DFC and GAH assessed initial search results and selected studies for inclusion with discrepancies resolved with GNH and AvdB. DFC, GNH, JMOM and MA performed data extraction. DFC and GAH performed analysis. All authors were involved in formation and revision of final manuscript. The corresponding author attests that all listed authors meet authorship criteria and that no others meeting the criteria have been omitted.

**Competing interests:** No authors declare any competing interests.

**Funding:** This research was funded by the National Institute for Health and Care Research (NIHR) Community Healthcare MedTech and In Vitro Diagnostics Co-operative at Oxford Health NHS Foundation Trust. The views expressed are those of the author(s) and not necessarily those of the NHS, the NIHR or the Department of Health and Social Care. DFC received funding from the National Institute for Health Research as an Academic Clinical Fellow and the Wellcome Trust Doctoral Training Fellowship at the University of Oxford. GAH received funding by Ter Meulen Grant of the Royal Netherlands Academy of Arts and Sciences, and NWO talent programme Veni.

**Acknowledgements**: We thank Nia Roberts and the Bodleian healthcare libraries for assistance in conducting the search strategy. We acknowledge help in the review of foreign language studies provided by Patrick Garfjeld Roberts, University of Oxford. We appreciate those authors that provided patient data upon request to allow inclusion of their original studies

**Ethics approval statement:** Study design and methodology was prospectively registered (PROSPERO-CRD42017080036). Study design used analysis of published data which did not involve participants and so did not require institutional or research committee ethical approvals.

**Data sharing statement:** Data sharing is not applicable as no new datasets were generated in the generation of this study.

For peer review only


For peer review only - http://bmjopen.bmj.com/site/about/guidelines.xhtml

## References


1. Armon, K. *et al.* Determining the common medical presenting problems to an accident and emergency department. *Arch. Dis. Child.* **84**, 390–392 (2001).
2. Baxter, K. J., Nguyen, H. T. M. H., Wulkan, M. L. & Raval, M. V. Association of Health Care Utilization With Rates of Perforated Appendicitis in Children 18 Years or Younger. *JAMA Surg.* **153**, 544–550 (2018).
3. Rothrock, S. G., Skeoch, G., Rush, J. J. & Johnson, N. E. Clinical features of misdiagnosed appendicitis in children. *Ann. Emerg. Med.* **20**, 45–50 (1991).
4. Rentea, R. M. & St. Peter, S. D. Pediatric Appendicitis. *Surg. Clin. North Am.* **97**, 93–112 (2017).
5. Statistics, H. E. Hospital Episode Statistics (HES), Admitted Patient Care, England 2012-2013. *Hospital Episode Statistics (HES), Admitted Patient Care, England 2012-2013 [Report].* content.digital.nhs.uk/catalogue/PUB12566/hosp-epis-stat-admi-proc-2012-13-%0Dtab.xlsx (2012).
6. Wagner, J. M., McKinney, W. P. & Carpenter, J. L. Does This Patient Have Appendicitis? *JAMA* **276**, 1589–1594 (1996).
7. Tseng, J., Cohen, T., Melo, N. & Alban, R. F. Imaging utilization affects negative appendectomy rates in appendicitis: An ACS-NSQIP study. *Am. J. Surg.* **217**, 1094–1098 (2019).
8. Macco, S., Vrouenraets, B. C. & de Castro, S. M. M. Evaluation of scoring systems in predicting acute appendicitis in children. *Surgery* **160**, 1599–1604 (2016).
9. van Rossem, C. C. *et al.* Diagnosing acute appendicitis: surgery or imaging? *Color. Dis.* **18**, 1129–1132 (2016).
10. Lewis, S. R. R., Mahony, P. J. & Simpson, J. Appendicitis. *BMJ* **343**, (2011).
11. Meltzer, J. A. *et al.* Association of Delay in Appendectomy With Perforation in Children With Appendicitis. *Pediatr. Emerg. Care* **35**, 45–49 (2019).
12. Sola, R. *et al.* Standardized reporting of appendicitis-related findings improves reliability of ultrasound in diagnosing appendicitis in children. *J. Pediatr. Surg.* **53**, 984–987 (2018).
13. Di Saverio, S. *et al.* WSES Jerusalem guidelines for diagnosis and treatment of acute appendicitis. *World J. Emerg. Surg.* **11**, 1–25 (2016).
14. Andersson, R. E. B. Meta-analysis of the clinical and laboratory diagnosis of appendicitis. *Br. J. Surg.* **91**, 28–37 (2003).
15. Kwan, K. Y. & Nager, A. L. Diagnosing pediatric appendicitis: usefulness of laboratory markers. *Am. J. Emerg. Med.* **28**, 1009–1015 (2010).
16. Waterfield, T., Maney, J. A., Hanna, M., Fairley, D. & Shields, M. D. Point-of-care testing for procalcitonin in identifying bacterial infections in young infants: A diagnostic accuracy study. *BMC Pediatr.* **18**, 1–6 (2018).
17. Snyder, B. K. & Hayden, S. R. Accuracy of Leukocyte Count in the Diagnosis of Acute Appendicitis. *Ann. Emerg. Med.* **33**, 565–574 (1999).
18. Yu, C. W. *et al.* Systematic review and meta-analysis of the diagnostic accuracy of procalcitonin, C-reactive protein and white blood cell count for suspected acute appendicitis. *Br. J. Surg.* **100**, 322–329 (2013).
19. Ordóñez-Mena, J. M. *et al.* C-reactive protein and neutrophil count laboratory test requests from primary care: What is the demand and would substitution by point-of-care technology be viable? *J. Clin. Pathol.* **72**, 474–481 (2019).



For peer review only - http://bmjopen.bmj.com/site/about/guidelines.xhtml

20. Nepogodiev, D. *et al.* Appendicitis risk prediction models in children presenting with right iliac fossa pain (RIFT study): a prospective, multicentre validation study. *Lancet Child Adolesc. Heal.* **4**, 271–280 (2020).
21. Pogorelić, Z., Rak, S., Mrklić, I. & Jurić, I. Prospective validation of Alvarado score and pediatric appendicitis score for the diagnosis of acute appendicitis in children. *Pediatr. Emerg. Care* **31**, 164–168 (2015).
22. Gorter, R. R. *et al.* Diagnosis and management of acute appendicitis. EAES consensus development conference 2015. *Surg. Endosc. 2016 3011* **30**, 4668–4690 (2016).
23. Humes, D. J. & Simpson, J. Acute appendicitis. *Br. Med. J.* **333**, 530–531 (2006).
24. Moher, D., Liberati, A., Tetzlaff, J. & Altman, D. G. Preferred reporting items for systematic reviews and meta-analyses: the PRISMA statement. *BMJ* **339**, 332–336 (2009).
25. Whiting, P. F. *et al.* Quadas-2: A revised tool for the quality assessment of diagnostic accuracy studies. *Ann. Intern. Med.* **155**, 529–536 (2011).
26. Reitsma, J. B. *et al.* Bivariate analysis of sensitivity and specificity produces informative summary measures in diagnostic reviews. *J. Clin. Epidemiol.* **58**, 982–990 (2005).
27. Diaz, M. Performance measures of the bivariate random effects model for meta-analyses of diagnostic accuracy. *Comput. Stat. Data Anal.* **83**, 82–90 (2015).
28. RM Harbord, P Whiting, J. S. metandi: Stata software for statistically rigorous meta-analysis of diagnostic accuracy studies — University of Bristol. https://research-information.bris.ac.uk/en/publications/metandi-stata-software-for-statistically-rigorous-meta-analysis-o.
29. Steinhauser, S., Schumacher, M. & Rücker, G. Modelling multiple thresholds in meta-analysis of diagnostic test accuracy studies. *BMC Med. Res. Methodol.* **16**, 1–15 (2016).
30. Deeks, J. J., Macaskill, P. & Irwig, L. The performance of tests of publication bias and other sample size effects in systematic reviews of diagnostic test accuracy was assessed. *J. Clin. Epidemiol.* **58**, 882–893 (2005).
31. Andersson, R. E. *et al.* Diagnostic Value of Disease History , Clinical Presentation , and Inflammatory Parameters of Appendicitis. *World J. Surg.* **23**, 133–140 (1999).
32. Andersson, M. & Andersson, R. E. The appendicitis inflammatory response score: A tool for the diagnosis of acute appendicitis that outperforms the Alvarado score. *World J. Surg.* **32**, 1843–1849 (2008).
33. Thompson, G. C. *et al.* The use of routine blood cultures in pediatric appendicitis. *Pediatr. Emerg. Care* **33**, e160–e163 (2017).
34. Andersson, M. *et al.* Can new inflammatory markers improve the diagnosis of acute appendicitis? *World J. Surg.* **38**, 2777–2783 (2014).
35. Andersson, M. *et al.* Randomized clinical trial of Appendicitis Inflammatory Response score-based management of patients with suspected appendicitis. *Br. J. Surg.* **104**, 1451–1461 (2017).
36. González Del Castillo, J. *et al.* Diagnostic accuracy of the APPY1 Test in patients aged 2-20 years with suspected acute appendicitis presenting to emergency departments. *Emerg. Med. J.* **33**, 853–859 (2016).
37. Kaiser, M. *et al.* The diagnostic value of hepcidin to predict the presence and severity of appendicitis in children. *J. Surg. Res.* **222**, 102–107 (2018).
38. Khanafer, I. *et al.* Test characteristics of common appendicitis scores with and



For peer review only - http://bmjopen.bmj.com/site/about/guidelines.xhtml

without laboratory investigations: A prospective observational study. *BMC Pediatr.* **16**, 1–8 (2016).
39. Ozguner, I. F. *et al.* Are neutrophil CD64 expression and interleukin-6 early useful markers for diagnosis of acute appendicitis? *Eur. J. Pediatr. Surg.* **24**, 179–183 (2014).
40. Shommu, N. S. *et al.* The Use of Metabolomics and Inflammatory Mediator Profiling Provides a Novel Approach to Identifying Pediatric Appendicitis in the Emergency Department. *Sci. Rep.* **8**, 1–11 (2018).
41. Thompson, G. C. *et al.* Variation in the Diagnosis and Management of Appendicitis at Canadian Pediatric Hospitals. *Acad. Emerg. Med.* **22**, 811–822 (2015).
42. Dadeh AA, Puitong K. Predictive Factors to Diagnose Appendicitis in Children in the Emergency Department. Open Access Emerg Med 2021 Jul 29;13:363-372.
43. Mohammed, A. A., Daghman, N. A., Aboud, S. M. & Oshibi, H. O. The diagnostic value of C-reactive protein, white blood cell count and neutrophil percentage in childhood appendicitis. *Saudi Med. J.* **25**, 1212–1215 (2004).
44. van den Broek WT, van der Ende ED, Bijnen AB, Breslau PJ, Gouma DJ. Which children could benefit from additional diagnostic tools in case of suspected appendicitis? Journal of pediatric surgery. 2004;39(4):570-4.
45. Calvo Rigual, F. *et al.* [The value of C-reactive protein in the diagnosis of acute appendicitis in children]. *An. Esp. Pediatr.* **48**, 376–380 (1998).
46. Kouame, D. B. *et al.* La procalcitonine peut-elle aider au diagnostic des appendicites de l'enfant ? *Ann. Chir.* **130**, 169–174 (2005).
47. Samuel, M. Pediatric appendicitis score. *J. Pediatr. Surg.* **37**, 877–881 (2002).
48. Altali, K. *et al.* Escala INFURG-SEMES para el diagnóstico de apendicitis aguda en los pacientes de 2 a 20 años atendidos en los servicios de urgencias hospitalarios. *Emergencias (St. Vicenç dels Horts)* **84**, 231–236 (2017).
49. Huckins, D. S. *et al.* Prospective validation of a biomarker panel to identify pediatric ED patients with abdominal pain who are at low risk for acute appendicitis. *Am. J. Emerg. Med.* **34**, 1373–1382 (2016).
50. Huckins, D. S. *et al.* A novel biomarker panel to rule out acute appendicitis in pediatric patients with abdominal pain. *Am. J. Emerg. Med.* **31**, 1368–1375 (2013).
51. Anandalwar, S. P. *et al.* Use of white blood cell count and polymorphonuclear leukocyte differential to improve the predictive value of ultrasound for suspected appendicitis in children. *J. Am. Coll. Surg.* **220**, 1010–1017 (2015).
52. Corkum, K. S., Oyetunji, T. A., Grabowski, J. E., Rigsby, C. K. & Lautz, T. B. Absolute neutrophil count as a diagnostic guide for the use of MRI in the workup of suspected appendicitis in children. *J. Pediatr. Surg.* **54**, 1359–1364 (2019).
53. Esparaz, J. R. *et al.* A simple algorithm to improve quality while reducing resource utilization in evaluation of suspected appendicitis in children. *Am. J. Surg.* **217**, 469–472 (2019).
54. Malia, L. *et al.* Predictors for Acute Appendicitis in Children. *Pediatr. Emerg. Care* (2019).
55. Prada-Arias, M. *et al.* Diagnostic accuracy of fibrinogen to differentiate appendicitis from nonspecific abdominal pain in children. *Am. J. Emerg. Med.* **35**, 66–70 (2017).
56. Tuncer, A. A. *et al.* Can mean platelet volume, Neutrophil-to-Lymphocyte, Lymphocyte-to-Monocyte, Platelet-to-Lymphocyte ratios be favourable predictors for the differential diagnosis of appendicitis? *J. Pak. Med. Assoc.* **69**, 647–654 (2019).
57. Yazici M, Ozkisacik S, Oztan MO, Gursoy H. Neutrophil/lymphocyte ratio in the



For peer review only - http://bmjopen.bmj.com/site/about/guidelines.xhtml

diagnosis of childhood appendicitis. The Turkish journal of pediatrics. 2010;52(4):400-3.
58. Greer D, Bennett P, Wagstaff B, Croaker D. Lymphopaenia in the diagnosis of paediatric appendicitis: a false sense of security? ANZ J Surg 2019 Sep;89(9):1122-1125.
59. Oikonomopoulou, N. *et al.* Assessment of proadrenomedullin as diagnostic or prognostic biomarker of acute appendicitis in children with acute abdominal pain. *Am. J. Emerg. Med.* **37**, 1289–1294 (2019).
60. Blok GCGH, Veenstra LMM, van der Lei J, Berger MY, Holtman GA. Appendicitis in children with acute abdominal pain in primary care, a retrospective cohort study. Fam Pract 2021 Nov 24;38(6):758-765.
61. Verbakel, J. Y. *et al.* Point-of-care C reactive protein to identify serious infection in acutely ill children presenting to hospital: Prospective cohort study. *Arch. Dis. Child.* **103**, 420–426 (2018).
62. Reismann J, Schädlich D, Minderjahn MI, Rothe K, Reismann M. Eosinophilia in pediatric uncomplicated appendicitis is a time stable pattern. Pediatr Surg Int 2019 Mar;35(3):335-340.
63. Acharya, A., Markar, S. R., Ni, M. & Hanna, G. B. Biomarkers of acute appendicitis: systematic review and cost–benefit trade-off analysis. Surg. Endosc. 31, 1022–1031 (2017).
64. Shogilev, D. J., Duus, N., Odom, S. R. & Shapiro, N. I. Diagnosing appendicitis: Evidence-based review of the diagnostic approach in 2014. *West. J. Emerg. Med.* **15**, 859–871 (2014).
65. Benabbas, R., Hanna, M., Shah, J. & Sinert, R. Diagnostic Accuracy of History, Physical Examination, Laboratory Tests, and Point-of-care Ultrasound for Pediatric Acute Appendicitis in the Emergency Department: A Systematic Review and Meta-analysis. *Acad. Emerg. Med.* **24**, 523–551 (2017).
66. Horvath, A. R. *et al.* From biomarkers to medical tests: The changing landscape of test evaluation. *Clin. Chim. Acta* **427**, 49–57 (2014).
67. Lahaye, M. J. *et al.* Mandatory imaging cuts costs and reduces the rate of unnecessary surgeries in the diagnostic work-up of patients suspected of having appendicitis. *Eur. Radiol.* **25**, 1464–1470 (2015).
68. Gongidi, P. & Bellah, R. D. Ultrasound of the pediatric appendix. *Pediatr. Radiol.* **47**, 1091–1100 (2017).


For peer review only - http://bmjopen.bmj.com/site/about/guidelines.xhtml

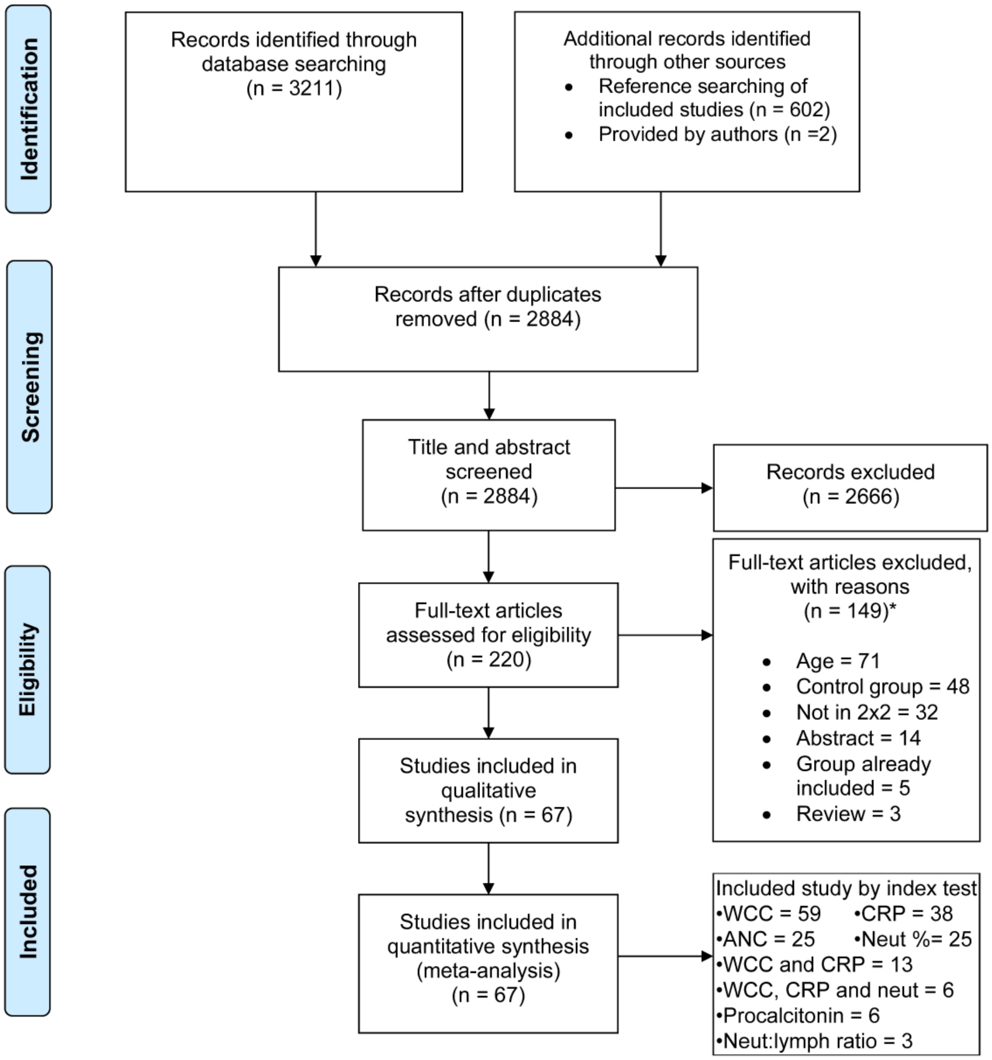


PRISMA flow chart for identification of included studies (*some studies excluded for 2 reasons, see full overview of excluded studies in Supplementary table 1)24

183x200mm (750 x 750 DPI)

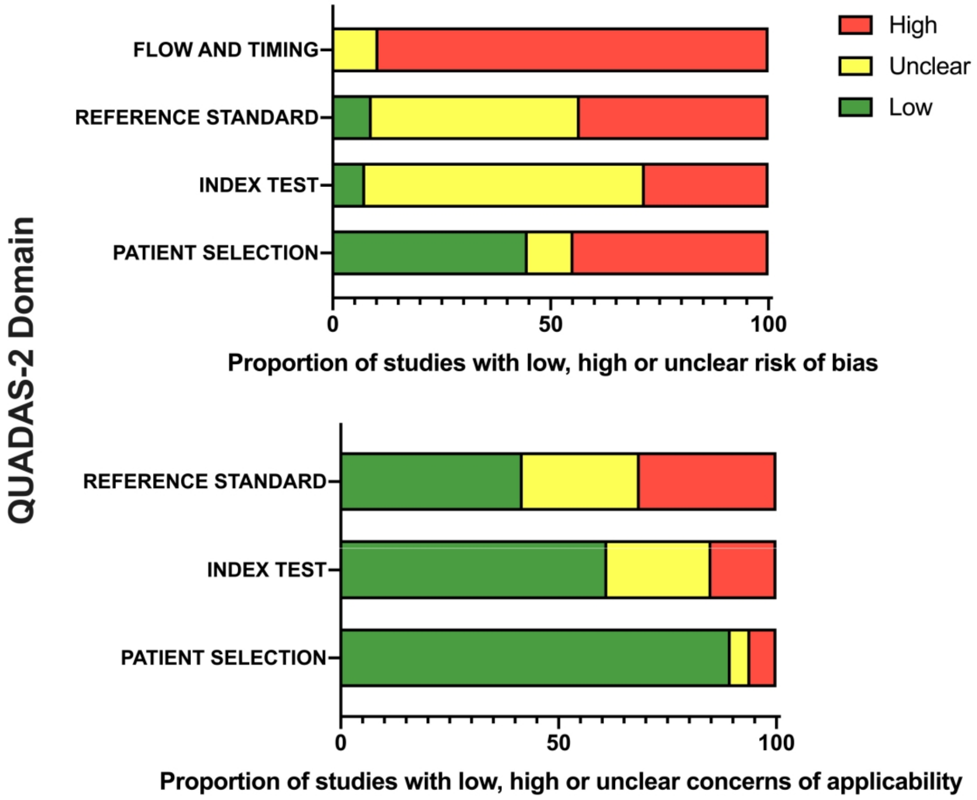


Figure 2: Overview of QUADAS-2 assessment of all included studies (n=67) showing review authors' assessment of risk of bias and study applicability in each domain reported as a percentage of all included studies (proportion coloured by assessment as high [red], unclear [yellow] or low [green], full outline of all individual components of QUADAS-2 is detailed in Supplementary table 4).

184x158mm (300 x 300 DPI)

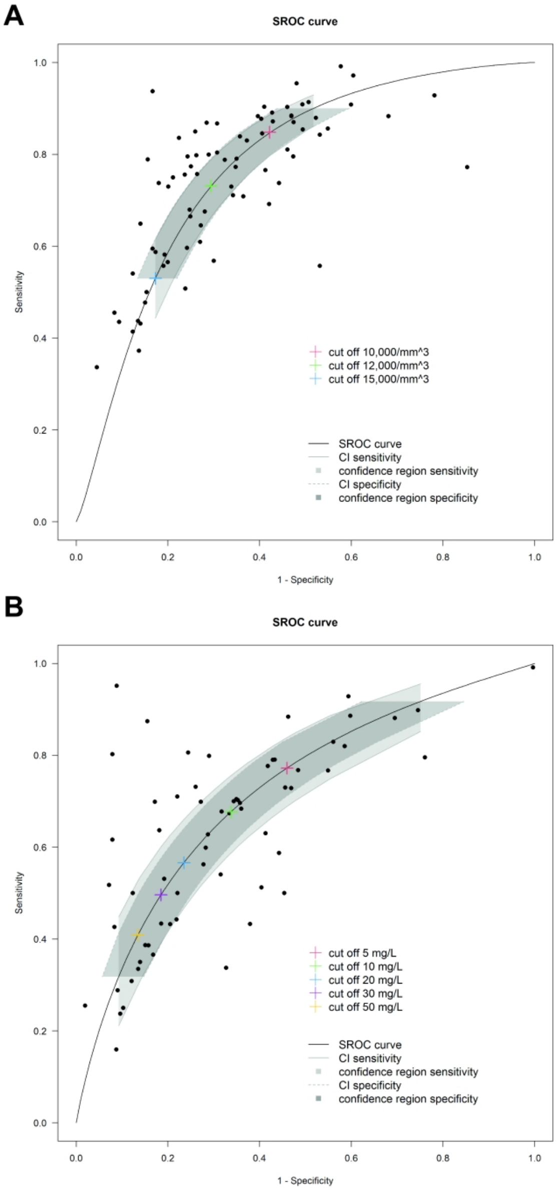


Figure 3: Summary receiver operating characteristic plot of WCC (A) and CRP (B) for diagnosis of paediatric acute appendicitis (Colour of dot representing study identified in legend, hashed grey box = 95% confidence interval specificity, solid grey box = 95% confidence interval sensitivity. Examples of commonly reported cut-offs shown in (A) as blue [10,000/µl], green [12,000/ µl] and red [15,000/ µl] crosses on curve; in (B) as yellow [5ml/L], pink [10mg/L], blue [20mg/L], green [30mg/L] and red [50mg/L] crosses on curve)

148x296mm (750 x 750 DPI)

# Supplementary Materials for "Diagnostic accuracy of blood tests in paediatric appendicitis – a systematic review and meta-analysis"

Overview of Supplementary materials

**Supplementary Tables:** ........ **2**

*Supplementary Table 1* ........ *2*

*Supplementary Table 2:* ........ *8*

*Supplementary Table 3:* ........ *13*

*Supplementary Table 4:* ........ *18*

*Supplementary Table 5:* ........ *20*

**Supplementary Figures and legends:** ........ **21**

*Supplementary Figure 1:* ........ *21*

*Supplementary Figure 2:* ........ *22*

*Supplementary Figure 3* ........ *23*

*Supplementary Figure 4* ........ *24*

*Supplementary Figure 5* ........ *24*

*Supplementary Figure 6* ........ *24*

*Supplementary Figure 7* ........ *25*

*Supplementary Figure 8* ........ *25*

**Search Strategy:** ........ **26**

**QUADAS-2 Scoring criteria** ........ **28**

**Supplementary References** ........ **30**

For peer review only

## Supplementary Tables:

**Supplementary Table 1:** Overview of studies excluded at full paper review stage and reason(s) for exclusion (* = Author was contacted and confirmed exclusion or did not respond. Unsuitable control group also includes those studies with no control group)

| First Author | Year | Exclusion Reason 1 | Exclusion Reason 2 |
|---|---|---|---|
| 1) Abu-Eshy | 1995 | Incompatible age demographics | Unsuitable control group |
| 2)Barker | 1998 | Unsuitable control group | Incompatible age demographics |
| 3)Bolandparvaz | 2004 | Incompatible age demographics | |
| 4)Bower | 1981 | Unsuitable control group | |
| 5)Clavien | 1987 | Incompatible age demographics | Unsuitable control group |
| 6)Cohen* | 2015 | Data not available in 2x2 | |
| 7)Depinet* | 2016 | Data not available in 2x2 | |
| 8)Erdem | 2015 | Incompatible age demographics | |
| 9)Ficek | 1958 | Unsuitable control group | |
| 10) Kharbanda | 2016 | Study population already included | |
| 11) Lofvenberg | 2016 | Unsuitable control group | Data not available in 2x2 |
| 12) Ng | 2002 | Incompatible age demographics | |
| 13) Park | 2010 | Incompatible age demographics | |
| 14) Vegar-Zubovic | 2003 | Unsuitable control group | |
| 15) Wattanasirichaigoon | 1994 | Incompatible age demographics | |
| 16) Anielski | 2010 | Incompatible age demographics | |
| 17) Atema | 2015 | Incompatible age demographics | |
| 18) Kessler | 2004 | Incompatible age demographics | |
| 19) Nasiri | 2012 | Incompatible age demographics | |
| 20) Thuijls | 2011 | Incompatible age demographics | |
| 21) Yardimci | 2016 | Incompatible age demographics | |
| 22) Yorke* | 2016 | Abstract | Incompatible age demographics |



For peer review only - http://bmjopen.bmj.com/site/about/guidelines.xhtml

For peer review only

| | | | |
|---|---|---|---|
| 23) Young | 1986 | Review | |
| 24) Agrawal | 2008 | Incompatible age demographics | Unsuitable control group |
| 25) Albu | 1994 | Incompatible age demographics | Data not available in 2x2 |
| 26) Allister* | 2011 | Incompatible age demographics | Data not available in 2x2 |
| 27) Amland | 1989 | Incompatible age demographics | |
| 28) Atema | 2015 | Incompatible age demographics | Unsuitable control group |
| 29) Bakal* | 2016 | Data not available in 2x2 | |
| 30) Bealer | 2010 | Incompatible age demographics | |
| 31) Beardsley* | 2014 | Abstract | |
| 32) Beardsley* | 2012 | Abstract | |
| 33) Bhatt* | 2009 | Data not available in 2x2 | |
| 34) Birkhahn | 2006 | Incompatible age demographics | |
| 35) Blab* | 2004 | Data not available in 2x2 | |
| 36) Cardall | 2004 | Incompatible age demographics | |
| 37) Chakhunashvili | 2005 | Unsuitable control group | |
| 38) Chandel | 2011 | Unsuitable control group | |
| 39) Colvin* | 2007 | Data not available in 2x2 | |
| 40) D'Souza | 2013 | Incompatible age demographics | |
| 41) Davies | 1991 | Incompatible age demographics | |
| 42) Dueholm | 1989 | Incompatible age demographics | |
| 43) Duzgun | 2007 | Incompatible age demographics | |
| 44) Eriksson | 1994 | Incompatible age demographics | |
| 45) Eriksson | 1995 | Incompatible age demographics | |
| 46) Eriksson | 1995 | Incompatible age demographics | |
| 47) Erkasap | 2000 | Incompatible age demographics | |
| 48) Escriba* | 2011 | Data not available in 2x2 | |
| 49) Gendel | 2011 | Unsuitable control group | Data not available in 2x2 |



For peer review only - http://bmjopen.bmj.com/site/about/guidelines.xhtml

| | | | |
|---|---|---|---|
| 50) Groselj-Grenc | 2007 | Study population already included | |
| 51) Gurau | 2016 | Unsuitable control group | Data not available in 2x2 |
| 51) Hallan | 1997 | Incompatible age demographics | |
| 53) Hotic | 2012 | Unsuitable control group | Data not available in 2x2 |
| 54) Hu* | 2014 | Data not available in 2x2 | |
| 55) Huang* | 2012 | Data not available in 2x2 | |
| 56) Jahn | 1997 | Incompatible age demographics | |
| 57) Jamaluddin | 2013 | Incompatible age demographics | |
| 58) Johansson | 2007 | Incompatible age demographics | |
| 59) John | 1993 | Incompatible age demographics | |
| 60) Khan | 2004 | Incompatible age demographics | |
| 61) Khan | 2009 | Incompatible age demographics | Unsuitable control group |
| 62) Kharbanda* | 2011 | Data not available in 2x2 | |
| 63) Kosloske* | 2004 | Data not available in 2x2 | |
| 64) Kostic* | 2010 | Data not available in 2x2 | |
| 65) Kyriakidis | 2010 | Incompatible age demographics | |
| 66) Lam | 2014 | Incompatible age demographics | |
| 67) Li | 2005 | Unsuitable control group | Data not available in 2x2 |
| 68) Li | 2012 | Unsuitable control group | |
| 69) Lietzen | 2016 | Incompatible age demographics | |
| 70) Lin* | 2007 | Data not available in 2x2 | |
| 71) Mollitt* | 1988 | Data not available in 2x2 | |
| 72) Oosterhuis | 1993 | Incompatible age demographics | |
| 73) Patel | 2014 | Unsuitable control group | |
| 74) Raftery | 1976 | Incompatible age demographics | Unsuitable control group |
| 75) Ring-Mrozik* | 1991 | Unsuitable control group | |
| 76) Sack* | 2006 | Unsuitable control group | |



For peer review only - http://bmjopen.bmj.com/site/about/guidelines.xhtml

| | | | |
|---|---|---|---|
| 77) Sarsu* | 2017 | Unsuitable control group | |
| 78) Schellekens | 2013 | Incompatible age demographics | |
| 79) Socea | 2013 | Incompatible age demographics | |
| 80) Sondenaa | 1992 | Incompatible age demographics | |
| 81) Suhaymi | 2017 | Unsuitable control group | Incompatible age demographics |
| 82) Summa | 2007 | Incompatible age demographics | |
| 83) Tamanna | 2012 | Incompatible age demographics | |
| 84) Tanrikulu | 2014 | Incompatible age demographics | |
| 85) Tepel | 2004 | Incompatible age demographics | |
| 86) Thimsen | 1989 | Incompatible age demographics | |
| 87) Thompson | 1992 | Incompatible age demographics | |
| 88) Turkyilmaz | 2000 | Unsuitable control group | |
| 89) Uyanik | 2012 | Unsuitable control group | |
| 90) van den Broek | 2002 | Study population already included | |
| 91) Wu | 2010 | Unsuitable control group | |
| 92) Wu | 2006 | Incompatible age demographics | |
| 93) Xharra | 2012 | Incompatible age demographics | |
| 94) Zviedre* | 2014 | Abstract | |
| 95) Brown* | 2004 | Abstract | |
| 96) Hodgkinson* | 2011 | Abstract | |
| 97) Hodgkinson* | 2011 | Abstract | |
| 98) Akin | 2018 | Unsuitable control group | |
| 99) Aydin | 2017 | Incompatible age demographics | |
| 100) Favot | 2017 | Review | |
| 101) Kharbanda* | 2018 | Study population already included | Data not available in 2x2 |
| 102) Mikaere | 2018 | Incompatible age demographics | |
| 103) Nazik | 2017 | Unsuitable control group | |



For peer review only - http://bmjopen.bmj.com/site/about/guidelines.xhtml

| | | | |
|---|---|---|---|
| 104) Nia | 2018 | Unsuitable control group | |
| 105) Salih | 2017 | Incompatible age demographics | Unsuitable control group |
| 106) Zouari | 2017 | Unsuitable control group | |
| 107) Daly | 2018 | Unsuitable control group | |
| 108) Elmas | 2017 | Unsuitable control group | |
| 109) Garcia Gamiz* | 2016 | Abstract | |
| 110) Oztan | 2018 | Unsuitable control group | |
| 111) Shommu | 2018 | Unsuitable control group | |
| 112) Yu* | 2018 | Unsuitable control group | |
| 113) Zouari | 2018 | Unsuitable control group | |
| 114) Sola | 2017 | Unsuitable control group | |
| 115) Zahoor* | 2018 | Abstract | |
| 116) van Dieijen-Visser | 1991 | Incompatible age demographics | |
| 117) Snyder | 1999 | Review | |
| 118) Sengupta | 2009 | Incompatible age demographics | Data not available in 2x2 |
| 119) Scott | 2015 | Incompatible age demographics | |
| 120) Randall Bond* | 1990 | Data not available in 2x2 | |
| 121) Msolli | 2018 | Incompatible age demographics | |
| 122) Malik | 1998 | Incompatible age demographics | |
| 123) Macco* | 2016 | Data not available in 2x2 | |
| 124) Lee | 2008 | Abstract | Incompatible age demographics |
| 125) Kohn | 2005 | Incompatible age demographics | |
| 126) Jang | 2008 | Incompatible age demographics | |
| 127) Haghi | 2018 | Incompatible age demographics | |
| 128) Blitman* | 2015 | Data not available in 2x2 | |
| 129) Akturk | 2018 | Incompatible age demographics | |
| 130) Ahmed | 2017 | Unsuitable control group | |

For peer review only



For peer review only - http://bmjopen.bmj.com/site/about/guidelines.xhtml

| | | | |
|---|---|---|---|
| 131) Agilinko | 2019 | Abstract | |
| 132) Benito | 2020 | Data not available in 2x2 | |
| 133) Boettcher | 2022 | Data not available in 2x2 | |
| 134) Dal | 2019 | Incompatible age demographics | |
| 135) Davis | 2021 | Data not available in 2x2 | |
| 136) Davis | 2019 | Abstract | |
| 137) Dooki | 2021 | Unsuitable control group | |
| 138) Duman | 2022 | Unsuitable control group | |
| 139) Duman | 2020 | Unsuitable control group | |
| 140) Güney | 2019 | Data not available in 2x2 | |
| 141) Lima | 2019 | Unsuitable control group | |
| 142) Morandi | 2020 | Unsuitable control group | |
| 143) Musbahi | 2019 | Unsuitable control group | Data not available in 2x2 |
| 144) Najd Sepas | 2019 | Unsuitable control group | Data not available in 2x2 |
| 145) Nawaz | 2021 | Abstract | |
| 146) Ng | 2019 | Abstract | |
| 147) Pehlivanli | 2019 | Incompatible age demographics | Unsuitable control group |
| 148) Saleem | 2021 | Incompatible age demographics | Unsuitable control group |
| 149) Altali Alhames | 2021 | Study population already included | |

For peer review only

**Supplementary Table 2:** Overview of demographics of included studies describing design, setting and population characteristics (* = data not provided or none described. AA= acute appendicitis. (P)ED = (Paediatric) Emergency Department. US = Ultrasound. CT = Computed Tomography. PICU= Paediatric Intensive Care Unit. In studies where authors provided paediatric data sub-population information is shown in square brackets.)

| Study | Study Design; Setting; Country | No. of children in study; with AA (% prevalence) | Age range (years) | Gender (n (%) male) | Inclusion criteria | Exclusion criteria |
|---|---|---|---|---|---|---|
| Akgül, 2021 (1) | Prospective cohort study; PED; Turkey | 320, 190 (59%) | 2 to 18 | 201 (63%) | Acute abdominal pain and Paediatric Appendicitis Score (PAS) above 3 points | Previous appendicectomy. Abdominal trauma. Malignancies. Inflammatory intestinal disease. Familial Mediterranean Fever. |
| Altali, 2017 (2) | Prospective cohort study; Hospital ED; Spain | 331, 116 (35%) | 2 to 20 | 175 (53%) | Abdominal pain <72 hours suspicious of AA as reviewed by doctor | Radiology prior to presentation. Pregnancy. Previous appendicectomy. Cancer. Trauma. Surgery in last 7 days. Steroids, immunosuppressive treatment or chemotherapy. |
| Anandalwar, 2015 (3) | Retrospective cohort study; ED; USA | 845, 393 (47%) | 3 to 18 | * (48%) | Consultation by surgeon for suspected AA. Underwent abdominal US. | History of abdominal surgery. Cognitive delay. Neurologic impairment. Immunodeficiency. Antibiotic use. Missing laboratory data. Abdominal imaging before presentation. |
| Andersson, 1999 (4) | Prospective cohort study; ED, 2 centres; Sweden | 496,[data provided n=296 <18yrs] 79(27%) | 10 to 86 | 133 (45%) | Patients admitted with suspected AA | * |
| Andersson, 2008 (5) | Prospective cohort study; Hospital, 2 centres; Sweden | 545 [data provided n=436 <18yrs, 140 of which not included in Andersson 1999] , 191[25](18%) | All | 58 (41%) | Admitted with suspected AA | Patients with missing blood tests. |
| Andersson, 2014 (6) | Prospective cohort study; Hospital ED, 2 centres; Sweden | 428 [data provided n=259 children], 177[76](30%) | 4 to 84 | 133 (49%) | Abdominal pain <5 days suggestive of AA | Pregnancy. Inability to consent. Multiple missing variables. |
| Andersson, 2017 (7) | Prospective interventional study with a nested randomized trial; ED, 21 centres; Sweden | 3791[data provided n=1046 children], [338](33%) | 5 to 95 | 2052 (54%) | Acute abdominal pain suggestive of AA | Pregnancy. Pain lasting >5 days. |
| Bachur 2016 (8) | Prospective cohort study; ED; USA | 2133, 870 (41%) | 3 to 18 | * (52%) | Acute abdominal pain < 72 hours. Had a WCC count and WCC differential obtained. | Pregnancy. Prior abdominal surgery. Chronic gastrointestinal condition. Severe developmental delay. |
| Bal 2016 (9) | Prospective cohort study; PED; Turkey | 96, 56 (58%) | 4 to 18 | 58 (21%) | Acute abdominal pain < 96 hours. Surgical consultation for suspected AA. Documented follow up. | Pregnancy. Familial Mediterranean fever. Chronic abdominal illness. Sickle cell anaemia. Cystic fibrosis. Previous abdominal surgery. Immunosuppressive therapy. |
| Beltran, 2007 (10) | Prospective Cohort study; Hospital; ED; Chile | 121, 71 (59%) | 5 to 14 | 73 (60%) | Presenting with abdominal pain, nausea, vomiting and fever. | Re-presentation. |
| Benito, 2016 (11) | Prospective cohort study; PED; Spain | 185, 89 (48%) | 2 to 14 | 123 (66%) | Abdominal pain suggestive of AA as evaluated by attending physician. | Symptoms >5 days. Prior appendicectomy. Urinary tract infection. Cancer. Inflammatory disease. Corticoids |
| Calvo, 1998 (12) | Retrospective cohort study; Hospital; Spain | 231, 141 (61%) | 2 to 14 | 149 (65%) | Acute abdominal pain. Inpatient. | Less severe cases of abdominal pain. |
| Cayrol 2016 (13) | Prospective cohort study; PED; Spain | 135, 52 (39%) | 1 to 15 | 69 (51%) | Children with acute abdominal pain suggestive of AA. | Previous appendicectomy. Immunological, haemostatic or thrombotic disorder. Vasculitis. Cardiopathy. Cancer. Pregnancy. |
| Chiang, 2020 (14) | Retrospective cohort study; ED; Singapore | 1391, 718 (52%) | 2-16 | 814 (59%) | Admitted with suspected AA based on clinical history and physical examination. | Pregnancy. Previous appendicectomy. Inflammatory disease. Cancer. |


For peer review only - http://bmjopen.bmj.com/site/about/guidelines.xhtml

| | | | | | | |
|---|---|---|---|---|---|---|
| Corkum, 2018 (15) | Retrospective cohort study; ED; USA | 738, 156 (21%) | 5 to 18 | * | Child with suspected AA -defined by undergoing an abdominal US and had blood test. | * |
| Dadeh, 2021 (16) | Retrospective cohort study; ED; Thailand | 1043, 97 (9%) | 3 to 14 | 565 (54%) | Acute abdominal pain. | Intra-abdominal organ disease. Previous abdominal surgery. Cancer. Incomplete medical records. |
| Doraiswamy 1979 (17) | Case control study; Hospital; UK | 375, 225 (60%) | 5 to 15 | * | Cases: children with AA. Controls: children with normal appendix on histology and children with acute abdominal pain. | Extra-abdominal complications. |
| Esparaz, 2019 (18) | Retrospective cohort study; ED; USA | 327, 56 (17%) | 2 to 17 | * | Abdominal US for suspected AA | None |
| Fleischman, 2013 (19) | Prospective cohort study; PED; USA | 178, 65 (37%) | 3 to 18 | 84 (47%) | Patients with symptoms suggestive of AA, duration <7 days. | Negative appendectomy but other diagnosis indicated by imaging/surgery |
| Goldman, 2008(20) | Prospective cohort study; ED; Canada | 849, 123 (36%) | 1 to 17 | * | Children with chief complaint of abdominal pain for <7 days. | Known diagnosis of AA (US or CT) on arrival to ED. Patients with prior appendicectomy. |
| Gonzalez del Castillo, 2016 (21) | Prospective cohort study; ED, 4 centres; Spain | 321, 111 (35%) | 2 to 20 | 167 (52%) | Abdominal pain <72 hours suggestive of AA | Ulcerative colitis. CT prior to consent. Pregnancy. Appendicectomy. Cancer. Trauma. Surgery within 7 days. Immunosuppressive treatment or chemotherapy. |
| Greer, 2019 (22) | Retrospective cohort study; ED; Australia | 546, 86 (16%) | 2 to 15 | 278 (51%) | Abdominal pain. | Trauma. Other confirmed diagnoses. Patients without blood test. |
| Groselj-Grenc 2007(23) | Nested case-control study; Hospital; Slovenia | 82, 49 (60%) | 3 to 14 | 53 (65%) | Cases: Children who underwent surgery for AA. Controls: Diagnosis of non-specific abdominal pain or sonographic mesenteric lymphadenitis. | Clinically or microbiologically established infections. |
| Hsiao 2005(24) | Case-control; Hospital; Taiwan | 222, 111 (50%) | 0 to 14 | * (ratio 1.52 M:1 F) | Cases: Diagnosis of AA on histology. Controls: Suspected AA in same period. | None |
| Huckins 2013(25) | Prospective cohort study; ED; 12 centres; USA | 503, 144 (29%) | 2 to 20 | 243 (48%) | Right lower quadrant/ generalized abdominal pain with other signs/ symptoms consistent with AA of duration < 72 hours. | Previous appendicectomy. Cancer. Bleeding disorder. Trauma. Invasive abdominal procedures or prior diagnostic imaging during this illness. Participation in other research protocols within 2 weeks. Unable to obtain consent. |
| Huckins 2016(26) | Prospective cohort study; ED; 29 centres; USA | 1887, 477 (25%) | 2 to 20 | 848 (45%) | Right lower quadrant/generalized abdominal pain <72 hours with other signs/symptoms suggesting AA. Informed consent. | Previous appendicectomy. Cancer. Bleeding disorder. Autoimmune disorder. Trauma. Immunosuppressive medications. Invasive abdominal procedure within 2 weeks. Surgery without imaging. Prisoners. Participation in other research protocol within 30 days. |
| Kaiser, 2018(27) | Case control study; Hospital; Austria | 64, 39(61%) | *(mean age 12 yr) | 24 (38%) | Cases: children who underwent appendectomy. Controls: abdominal pain and improved under conservative treatment | * |
| Kashtan, 2020 (28) | Retrospective cohort study; ED; USA | 2277, 974 (43%) | 3 to 18 | 1098 (48%) | Abdominal US and surgical consultation for possible AA. | Missing data. Prior abdominal surgery. Immunodeficiency. |
| Khan 2012 (29) | Prospective cohort study; PED; USA | 50, 22 (44%) | 5 to 17 | 22 (44%) | Right lower quadrant abdominal pain without signs of gastroenteritis. | Chronic haematological, immunological or gastrointestinal disease. Pregnancy. |
| Khanafer, 2016(30) | Prospective cohort study; paediatric ED; Canada | 180, 55(31%) | 5 to 17 | 78 (43%) | Abdominal pain <5 days. AA in differential from ED with bloods done. | Previous appendicectomy. Positive imaging prior to presentation. Pregnancy. Immuno-suppressive disorders. |
| Kharbanda 2005(31) | Prospective cohort study; PED; USA | 601, 211 (37%) | 3 to 18 | 307 (51%) | Surgical consultation for possible AA | Pregnant. Previous abdominal surgery. Chronic medical condition. Radiological study of abdomen within 2 weeks. |
| Kharbanda, 2012 (32) | Prospective cross-sectional study; PED; USA | 176, 58 (33%) | 3 to 18 | 92 (52%) | Acute abdominal pain <96 hours duration evaluated for AA as defined by clinician. | Pregnancy. Prior abdominal surgery, chronic GI illness. Inaccurate history. Imaging / abdominal trauma in <7 days. |
| Kharbanda, 2012 (33) | Prospective cross-sectional study; PED; 10 centres; USA | 2625, 1018 (39%) | 3 to 18 | 1339 (51%) | Abdominal pain <96 hours duration in keeping with AA (as defined by clinician). | Pregnancy. Prior abdominal surgery. Chronic abdominal illness. Sickle cell. Cystic fibrosis. Radiological investigation diagnosing AA before arrival. Trauma within 7 days |

For peer review only



For peer review only - http://bmjopen.bmj.com/site/about/guidelines.xhtml

| | | | | | | |
|---|---|---|---|---|---|---|
| Klein, 2021 (34) | Prospective cohort study; PED; Israel | 134, 53 (40%) | 3 to 17 | 71 (53%) | Admitted with suspected AA | Previous appendectomy. Cancer. Haematological disorders. Active auto-inflammatory disorders. Trauma. Abdominal surgery. Previously known infectious disease such as urinary tract infection or pneumonia. Previously known urological/gynecological disorders such as pelvic inflammatory disease. |
| Ko, 1995 (35) | Prospective cohort study; Hospital; Taiwan | 47, 27 (57%) | 2 to 14 | 29 (62%) | Patients with clinically suspected AA | Atypical clinical signs. Didn't complete protocol. |
| Kouame, 2005 (36) | Prospective diagnostic accuracy study; Hospital; France | 101, 68 (67%) | 2 to 15 | 52 (51%) | Hospitalised children with abdominal pain that was suspected to require surgery. | Antibiotics prior to hospital presentation. |
| Kumar, 2021 (37) | Prospective observational study; tertiary PED; India | 120, 55 (46%) | 5 to 12 | Unknown | Acute right lower abdomen pain. | Duration of symptoms <6 h or >72 h. Haematological disorders. Oncological disorders. Hepatic dysfunction. Infectious, or inflammatory conditions. Anticoagulants, anti-inflammatory medications or antibiotics. |
| Kwan, 2010(38) | Prospective observational study; PED; USA | 209, 115 (55%) | 1 to 18 | 123 (59%) | Abdominal pain suspicious of AA. | Pregnancy. Bleeding disorder. Severe anaemia. Chronic disease. Abdominal surgery in the previous year. |
| Lin, 2009(39) | Retrospective cohort study; ED; Taiwan | 100, 53 (53%) | 2 to 17 | 46(46%) | Children with RLQ pain presenting to ED | Patients who went home against medical advice. |
| Lycopoulou, 2005(40) | Prospective observational with nested case-control study; PED; Greece | 60, 42 (70%) | 5 to 14 | 30 (50%) | A nested control of children operated for AA; Controls: children presenting to PED during same period with symptoms and signs of AA | No consent. Retrocaecal abscess. Normal appendix histology. |
| Malia, 2019(41) | Prospective cohort study; Paediatric hospital ED; USA | 762, 225 (30%) | 0 to 18 | 356 (47%) | Children with suspected AA who underwent US | Patient absconded, previous appendicectomy, appendix not visualised on US, US at other facility. |
| Mandeville, 2011(42) | Prospective observational study; PED; USA | 287, 155 (54%) | 4 to 17 | 151 (53%) | Abdominal pain suggestive of AA | Pregnancy, previous abdominal surgery, chronic medical condition, non-verbal, radiological investigation of abdomen in preceding 2 weeks, no follow up. |
| Miguez, 2016(43) | Prospective cohort study; Paediatric hospital ED; Spain | 136, 44 (32%) | 3 to 16 | 76 (56%) | Admitted to ED with abdominal pain <96 hours AA suspected at initial evaluation. | Appendicectomy /recent surgery. Immune disease. Chronic respiratory or cardiovascular disease. Inflammatory bowel disease. Antibiotics or steroids in the last month. |
| Mohammed, 2004(44) | Prospective cohort study; Hospital paediatric surgical department; Libya | 216, 130 (60%) | 3 to 13 | 139(64%) | Child presenting with clinical suspicion of AA | * |
| Moreno, 2012(45) | Retrospective Cohort study; Hospital; Spain | 184, 85 (46%) | * (Mean age 8.9 yr) | 110 (60%) | Acute abdominal pain suggestive of AA. | * |
| Naqvi, 2019(46) | Observational cohort study; Tertiary Paediatric Hospital; Canada | 185, 79 (43%) | 4 to 17 | 86 (46%) | Children with suspected AA demonstrated by US or referral to paediatric surgical team. Also children with sepsis defined by SIRS criteria or PICU admission (AA sub-group reported) | Previous appendicectomy, resuscitation in ED, discharge to PICU, pregnancy, AP >5 days, immune-suppression, abdominal imaging prior to admission, life expectancy <24 hours. |
| Oikonomopoulou, 2019(47) | Prospective cohort study; PEDs, 6 centres; Spain | 285, 100 (35%) | 0 to 18 | 167 (59%) | Clinical suspicion of AA after physician assessment | Pain >72hr, lack of blood sample, history of surgery (within 3 months)/ immune pathology/ inflammatory bowel disease/ cardio-respiratory disease, recent steroid or antibiotic use |



For peer review only - http://bmjopen.bmj.com/site/about/guidelines.xhtml

| | | | | | | |
|---|---|---|---|---|---|---|
| Ovrebo, 1993(48) | Retrospective case series; Hospital surgical department; Norway | 470, 161 (34%) | 0 to 15 | 235 (50%) | All patients presenting with abdominal pain <1 week duration suggestive of AA. | Abdominal trauma. Incarcerated herniae. |
| Ozguner, 2014(49) | Prospective cohort study; Children's hospital; Turkey | 49, 34(69%) | 3 to 17 | 34 (69%) | Children hospitalised with right lower-quadrant pain suspicious of AA between 0400 and 1500. | If sample for CD64 measurement could not be obtained. |
| Peltola, 1986(50) | Prospective cohort study; Children's hospital; Finland | 162, 94 (58%) | 1 to 16 | 77 (48%) | Children presenting with abdominal pain or symptoms suggestive of AA | Symptoms suggestive or respiratory infection. |
| Prada-Arias, 2017(51) | Prospective cohort study; Tertiary hospital PED; Spain | 275, 132 (48%) | 5 to 15 | 153 (56%) | Clinical suspicion of AA with blood tests and US performed | Symptom duration <6hr or >72hr. Haematological / oncological / hepatic / infectious or inflammatory disease present. >12 hours between blood test and surgery |
| Rodríguez Sánchez de la Blanca, 2014(52) | Prospective cohort study, PED, Spain | 105, 41 (39%) | <16 | 59 (56%) | Abdominal pain or clinical suspicion of AA | Antibiotic treatment in the preceding 10 days, known inflammatory pathology |
| Salo, 2016(53) | Case control study; PED; Sweden | 44, 22 (50%) | 3 to 14 | 27 (61%) | Referral to paediatric surgeon with suspicion of AA | Immuno-modulating therapy or clear-cut diagnosis of AA |
| Samuel, 2002(54) | Prospective cohort study; Hospital, 2 centres; UK | 1170, 734 (63%) | 4 to 15 | 752(64%) | Abdominal pain suggestive of AA | Appendicula mass with peri-appendiceal abscess |
| Sanchez, 1998(55) | Prospective cohort study; Paediatric hospital ED; Spain | 195, 94 (48%) | 2 to 14 | 107(55%) | Children with suspected AA | * |
| Shommu, 2018(56) | Prospective cohort study; PED; Canada | 140, 52 (36%) | 5 to 17 | 61 (44%) | Clinically suspected AA or US evaluation for AA with IV placed and did not require intensive care treatment from PED. Informed consent | Previous appendectomy. Pregnancy. Abdominal pain >5 days. Immuno-suppression. Previously enrolled in the study. Imaging study performed elsewhere |
| Thompson, 2015(57) | Retrospective cohort study; ED, 12 centres; Canada | 619, 510 (83%) | 3 to 17 | 352 (53%) | Children admitted with suspected AA | Intensive care unit admission. |
| Thompson, 2016 (58) | Retrospective cohort study; Children's hospital ED; Canada | 1315, 561[data provided n=1138,471 children] (41%) | 2 to17 | 565 (43%) | Children investigated for suspected AA with blood culture sample collected | * |
| Tuncer, 2019(59) | Retrospective cross-sectional study; Paediatric Surgical/Medical department; Turkey | 301; 137 (46%) | *(Mean age 11yr) | 139 (46%) | Children with acute abdominal pain, recorded diagnosis of acute appendicitis, mesenteric lymphadenitis or familial Mediterranean fever. | Heart failure, peripheral vascular / haematological /liver disease, anticoagulation, steroid use, concurrent acute or chronic infection |
| Turkyilmaz, 2006(60) | Prospective cohort study; Hospital; Turkey | 105, 61 (58%) | 3 to 16 | 68 (65%) | Admission with acute right-lower abdominal pain | * |
| Van Den Broek, 2004(61) | Prospective cohort study; ED, 2 centres; Netherlands | 275, 169 (66%) | *(Mean age 9 yr) | 148 (58%) | GP referral with suspicion of AA | * |
| Wang 2007(62) | Prospective and retrospective cohort study; PED; USA | 722, 74 (18%) | 1 to 19 | 334 (46%) | Prospective: ED presentation with suspected AA. Retrospective: AA on surgery, radiology, and laboratory records. | History of trauma |
| Wu, 2012(63) | Prospective cohort study; ED; Taiwan | 594, 306 (52%) | 4 to 18 | 358 (60%) | Children with clinically suspected AA, as determined by emergency physician. | Symptoms >3 day. Patients lost to follow up. |
| Yap, 2015(64) | Prospective cohort study; Paediatric hospital ED; Singapore | 747, 229 (32%) | 4 to 16 | 429(57%) | Admitted to ED with suspected AA. | Previous appendicectomy, pregnancy, chronic medical or malignant conditions. CT in the previous month. |

For peer review only

| | | | | | | |
|---|---|---|---|---|---|---|
| Yazici, 2010(65) | Retrospective case series; Paediatric surgical department; Turkey | 240, 183 (76%) | 3 to 16 | * | Patients with acute abdominal pain | Urinary tract infection, Henoch-Schonlein pupura or gastroenteritis. |
| Zouari, 2016(66) | Prospective cohort study; Paediatric surgical department; Tunisia | 292, 132 (45%) | 3 to 14 | 170(58%) | Diagnosis of suspected AA by consultant paediatric surgeon on the basis of clinical history and physical exam | Patients who had not undergone US |
| Zuniga, 2012(67) | Prospective cohort study; Hospital ED; Spain | 101, 28 (28%) | 0 to 14 | 55 (54%) | Presenting with clinical suspicion of AA | Pain >7 days. Previous appendicectomy. No blood tests. |

For peer review only


For peer review only - http://bmjopen.bmj.com/site/about/guidelines.xhtml

**Supplementary Table 3:** Overview of included studies Index tests, cut off values and reference standards. (CRP = C-reactive protein. WCC = White Cell Count. PMN = Polymorphonuclear cells. * = non-AA not defined/not clearly defined, †=multiple cut offs or range in paper, ‡=author provided raw data)

| Study | Reference Standard | Index Test(s) measured | Index test cut off value(s) |
|---|---|---|---|
| Akgül, 2021 (1) | Histopathology or 14 days follow up | • WCC<br>• Neutrophil count<br>• CRP<br>• Procalcitonin | 10,000 µL<br>7500 µL<br>5 mg/L<br>0.5 ng/mL |
| Altali, 2017 (2) | Histopathology or discharge diagnosis | • WCC<br>• Neutrophil count<br>• CRP | >10,500/mm³<br>>6,600/mm³<br>>5mg/L |
| Anandalwar, 2015 (3) | Pathology proven appendicitis * | • WCC<br>• Neutrophil % | >9,000/mm³<br>>65% |
| Andersson, 1999 (4) | Operation note and histopathology or failure to re-present | • WCC<br>• Neutrophil count<br>• Neutrophil %<br>• CRP<br>WCC/CRP as separate and combined | ‡ |
| Andersson, 2008 (5) | Histopathology or review of case files in the following 1 month. | • WCC<br>• Neutrophil count<br>• Neutrophil %<br>• CRP<br>WCC/CRP as separate and combined | ‡ |
| Andersson, 2014 (6) | Histopathology or 6 month follow up | • WCC<br>• Neutrophil count<br>• Neutrophil %<br>• CRP<br>WCC/CRP as separate and combined | ‡ |
| Andersson, 2017 (7) | Histopathology or 30 day follow up | • WCC<br>• Neutrophil count<br>• Neutrophil %<br>• CRP<br>WCC/ CRP as separate and combined | ‡ |
| Bachur 2016 (8) | Operative diagnosis, telephone follow-up (1 -2 weeks) or record review (90 days) after visit. | • WCC<br>• Neutrophil count | 5-15,000/mm³†<br>5-15,000/mm³† |
| Bal 2016 (9) | Surgery and pathology report or follow-up by phone interview 7-14 days after visit | • WCC<br>• Neutrophil count<br>• CRP<br>• Procalcitonin | >13,400/mm³<br>>9,400/mm³<br>>0·9mg/L<br>0·37ng/mL |
| Beltran, 2007 (10) | Histopathological report or discharge diagnosis | • WCC<br>• CRP | >12,500/mm³<br>>47mg/L |


For peer review only - http://bmjopen.bmj.com/site/about/guidelines.xhtml

| | | | |
|---|---|---|---|
| Benito, 2016 (11) | Histopathological analysis of surgical specimen, telephone follow up 15 days after consultation, or check of electronic registry if not contactable. | • WCC<br>• Neutrophil count<br>• CRP<br>• Procalcitonin | <10,000/mm³<br><7,500/mm³<br><20 mg/L<br><0·1ng/ml |
| Calvo, 1998 (12) | Histopathological examination or discharge diagnosis | • WCC<br>• CRP<br>Tests reported as separate and as combination | >15,000/mm³<br>>20mg/l |
| Cayrol 2016 (13) | Surgery and histopathology or clinical decision with telephone follow up 5 days later | • WCC<br>• CRP | >10,000/mm³<br>>30mg/L |
| Chiang, 2020 (14) | Histopathology or 24 hours of clinical observation with or without radiologic imaging and with 3 days follow up after discharge. | • WCC<br>• Neutrophil %<br>• CRP | ≥10,000/µL<br>≥75%<br>≥5 mg/L |
| Corkum, 2018 (15) | Histopathology and operative note or 30 day follow up | • Neutrophil count | >8,000/mm³ |
| Dadeh, 2021 (16) | Histopathology * | • WCC<br>• Neutrophil % | >10,000 or >14,000<br>>75%<br>(based on duration of pain less or more than 24 hours) |
| Doraiswamy 1979 (17) | Appendicitis confirmed or refuted at surgery or recovered without surgery | • WCC<br>• Neutrophil % | >15,000/mm³ or >13,000/mm³†<br>>50%, >65% or >75%†<br>(age dependent) |
| Esparaz, 2019 (18) | Histopathology * | • WCC | >10,000/mm³ |
| Fleischman, 2013 (19) | Surgical pathology, positive CT scan finding or diagnosis and follow up at 2 weeks | • WCC<br>• Neutrophil count<br>• Neutrophil % | >10,000/mm³<br>>6750/mm³<br>>75% |
| Goldman, 2008(20) | Appendicectomy pathology or follow-up 5 to 7 days after ED discharge | • WCC<br>• Neutrophil count | >10,000/mm³<br>>7,500/mm³ |
| Gonzalez del Castillo, 2016 (21) | Histopathology reported by blinded pathologist or telephone follow up 2 weeks after discharge | • WCC<br>• CRP | >12,000/mm³<br>>5mg/L |
| Greer, 2019 (22) | Histopathology or discharge diagnosis | • WCC<br>• Neutrophil count<br>• CRP | >13.5×10⁹/L<br>>7.5×10⁹/L<br>≥5 mg/L |
| Groselj-Grenc 2007(23) | Appendicitis diagnosed by surgery or ultrasound, or diagnosis of non-specific abdominal pain/sonographic mesenteric adenitis | • WCC<br>• CRP | >11,600/mm³<br>>9.0mg/L |
| Hsiao 2005(24) | Surgery or pathology report, or appendicitis excluded in final diagnosis | • WCC<br>• Neutrophil % | >10,400/mm³<br>>75% |
| Huckins 2013(25) | Surgical pathology report after appendectomy or discharge diagnosis | • WCC<br>• CRP<br>Tests only reported in combination | >10,000/mm³<br>>8mg/L |
| Huckins 2016(26) | Surgical pathology reports after appendectomy or follow-up telephone calls at 14 days (±3 days) | • WCC<br>• CRP<br>Tests only reported in combination | >10,000/mm³<br>>8mg/L |
| Kaiser, 2018(27) | Histopathology or discharge diagnosis | • WCC<br>• CRP | >10 -15,000/mm³†<br>5-50mg/L† |


For peer review only - http://bmjopen.bmj.com/site/about/guidelines.xhtml

| | | | |
|---|---|---|---|
| | | Tests as separate and combined | |
| Kashtan, 2020 (28) | Histopathology or 7 days follow up | • WCC | >9000 cells/uL |
| Khan 2012 (29) | Histopathological evidence or telephone follow up 24 hour and 2 weeks after discharge | • Procalcitonin | >0·39ng/ml |
| Khanafer, 2016(30) | Histopathology or follow up 2 weeks after discharge | • WCC | 10-15,000m/$^{3\dagger}$ |
| Kharbanda 2005(31) | Pathology or follow-up telephone call 2 to 4 weeks after the ED visit | • WCC<br>• Neutrophil count | $>8850/mm^3$<br>$6{\cdot}75x10^3/ul$ |
| Kharbanda, 2012 (32) | Surgical histology or telephone follow up 14-21 days after admission/note review if uncontactable. | • WCC | $>8850/mm^3$ |
| Kharbanda, 2012 (33) | Histology or telephone follow up 2 weeks after discharge or note review 90 days after discharge if unable to contact. | • Neutrophil count | $>6750/mm^3$ |
| Klein, 2021 (34) | Histopathology or discharge diagnosis or clinical observation | • WCC<br>• Neutrophil % | > 12,000 or > 10,500<br>≥75%<br>(age dependent) |
| Ko, 1995 (35) | Surgical histology or diagnosis on discharge | • WCC<br>• Neutrophil %<br>• CRP | $>10,000/mm^3$<br>>75%<br>>9mg/L and >50mg/L |
| Kouame, 2005 (36) | Histology * | • Procalcitonin | >0.5µg/L |
| Kumar, 2021 (37) | Histopathology or 2 weeks follow up | • WCC<br>• Neutrophil count<br>• CRP | $14,930/mm^3$<br>10,485.5 cell/ul<br>10.5 mg/L |
| Kwan, 2010(38) | Operative reports and histology or telephone follow up or note review 2-6 weeks after discharge | • WCC<br>• CRP<br>Tests reported as separate and in combination | $>12,000/mm^3$<br>>30mg/L |
| Lin, 2009(39) | Appendectomy (histologic examination) or telephone follow-up within 1 week of consultation | • WCC<br>• CRP | $>15,000/mm^3$<br>>1 mg/dL |
| Lycopoulou, 2005(40) | Appendicectomy (histological examination) or 1 week follow up | • WCC<br>• CRP | $>10,000/mm^{3\dagger}$<br>>10mg/L |
| Malia, 2019(41) | Histopathology report or failure to re-attend within 1 week | • WCC<br>• CRP | $>10,000/mm^3$<br>>5mg/L |
| Mandeville, 2011(42) | Histopathology or telephone follow up at 2 weeks | • WCC<br>• Neutrophil % | $>10,000/mm^3$<br>>75% |
| Miguez, 2016(43) | Histopathology of appendicectomy specimen or telephone follow up 5-7 days afterwards. | • WCC<br>• Neutrophil count<br>• CRP | $>12,300/mm^3$<br>$>9,600/mm^3$<br>>3 mg/L |
| Mohammed, 2004(44) | Appendicectomy (histopathological results) or clinical observation | • WCC<br>• Neutrophil %<br>• CRP<br>Tests reported as separate and in combination (all three) | $>11,000/mm^3$<br>>75%<br>>8mg/L |
| Moreno, 2012(45) | Surgery, histology, clinical observation or follow up | • WCC<br>• CRP | $>12,800/mm^3$<br>>18mg/L |

For peer review only

| | | | |
|---|---|---|---|
| Naqvi, 2019(46) | Histopathology, insertion of percutaneous drain or failure to re-attend within 2 weeks | • CRP | >7·9mg/L |
| Oikonomopoulou, 2019(47) | Histopathology and surgeons notes, telephone follow up after 5-7 days or checked for readmission if no response | • WCC<br>• Neutrophil count<br>• CRP<br>Tests reported as separate and in paired combinations | >13,300/$mm^3$<br>>10,800/$mm^3$<br>>12·5mg/L |
| Ovrebo, 1993(48) | Operative note (all) and histology (some) or discharge diagnosis | • WCC<br>• CRP<br>Tests reported as separate and in combination | >11,000/$mm^3$<br>>10mg/L |
| Ozguner, 2014(49) | Operative findings, histopathological examination and discharge diagnosis | • WCC<br>• CRP | >12,000/$mm^3$<br>>16mg/L |
| Peltola, 1986(50) | Histology on those operated or diagnosis of non-specific abdominal pain | • WCC<br>• CRP<br>Tests reported as separate and in combination | >14,900/$mm^3$<br>>10mg/L |
| Prada-Arias, 2017(51) | Histology and surgical report or no specific disease identified, no findings on US and no antibiotic treatment | • WCC<br>• Neutrophil count<br>• CRP | >11,000/$mm^3$<br>>7,000/$mm^3$<br>>25·2mg/L |
| Rodríguez Sánchez de la Blanca, 2014(52) | Histopathology* | • WCC<br>• CRP<br>• Procalcitonin | >13,000/$mm^3$<br>>5mg/L<br>>0·5µg/L |
| Salo, 2016(53) | Surgical note and histopathology report or follow up | • WCC<br>• Neutrophil count<br>• Neutrophil %<br>• CRP<br>Combined WCC and neutrophil count reported | >13-16,000/$mm^{3\dagger}$<br>>6·5-7,000/$mm^{3\dagger}$<br>>71%<br>>15mg/L<br>(ranges age dependent) |
| Samuel, 2002(54) | Histology in surgical group* | • WCC<br>• Neutrophil count | >10,00/$mm^3$<br>>7,500/$mm^3$ |
| Sanchez, 1998(55) | Histology and surgical report or discharge diagnosis | • WCC<br>• CRP | >14000/$mm^3$<br>>30mg/L |
| Shommu, 2018(56) | Histopathology* | • WCC<br>• Neutrophil count<br>• CRP | ‡ (Provided 2x2 tables for multiple cut offs of tests but not IPD) |
| Thompson, 2015(57) | Histopathology or period of observation | • WCC<br>• Neutrophil count<br>• CRP | ‡ |
| Thompson, 2016 (58) | Histopathology or discharge diagnosis | • WCC<br>• Neutrophil count<br>• CRP | ‡ |
| Tuncer, 2019(59) | Histopathology or discharge diagnosis | • WCC | >13,050/$mm^3$ |
| Turkyilmaz, 2006(60) | Histopathology and operative assessment or clinical observation | • WCC | >15000/$mm^3$ or 13,000/$mm^{3\dagger}$ (age dependent) |
| Van Den Broek, 2004(61) | Histology* | • WCC | >10,000/$mm^3$ |
| Wang 2007(62) | Histology or review of medical records | • WCC | † (Age dependent cut-off) |



For peer review only - http://bmjopen.bmj.com/site/about/guidelines.xhtml

| | | | |
|---|---|---|---|
| | | • Neutrophil %<br>Tests reported as separate and in combination | >80% |
| Wu, 2012(63) | Surgery, histological examination or follow up interview two weeks after visit | • WCC<br>• CRP | >11000/mm$^3$<br>>25 and 89mg/L |
| Yap, 2015(64) | Histology, operative findings or observation on the ward until >24 hours pain free | • WCC<br>• Neutrophil %<br>• CRP<br>Tests reported as separate and in combination (all three) | > 10,000/mm$^3$<br>>75%<br>>5mg/L |
| Yazici, 2010(65) | Histopathology or observation for 24-48 hours | • WCC | >10,000/mm$^3$ |
| Zouari, 2016(66) | Histology showing acute appendicitis or failure to represent for 2 weeks | • CRP | 6mg/L |
| Zuniga, 2012(67) | Histopathology or failure to re-present for 7 days | • WCC<br>• Neutrophil count | >10,000/mm$^3$<br>>7,500/mm$^3$ |

For peer review only



For peer review only - http://bmjopen.bmj.com/site/about/guidelines.xhtml

**Supplementary Table 4:** Individual assessment scoring of each included study assessed by means of the QUADAS-2 tool for risk of bias.

| Study | RISK OF BIAS | | | | APPLICABILITY CONCERNS | | |
|---|---|---|---|---|---|---|---|
| | PATIENT SELECTION | INDEX TEST | REFERENCE STANDARD | FLOW AND TIMING | PATIENT SELECTION | INDEX TEST | REFERENCE STANDARD |
| Akgül, 2021 | High | High | Low | High | Low | Low | Low |
| Altali, 2017 | Low | Unclear | Low | Unclear | Low | High | Low |
| Anandalwar, 2015 | High | High | Unclear | Unclear | Low | High | Unclear |
| Andersson, 1999 | Unclear | High | High | High | Unclear | Unclear | High |
| Andersson, 2014 | Low | Unclear | Unclear | High | Low | Unclear | Low |
| Andersson, 2017 | Low | Unclear | Unclear | High | Low | Low | Low |
| Andersson,2008 | Low | Unclear | Unclear | High | Low | Unclear | Low |
| Bachur, 2016 | Low | Unclear | Unclear | High | Low | Unclear | Unclear |
| Bal, 2016 | High | Unclear | Unclear | High | Low | Low | Low |
| Beltran, 2007 | Low | Low | High | Unclear | Low | Low | Unclear |
| Benito, 2016 | High | Low | High | High | Low | Low | Low |
| Calvo Rigual, 1998 | Low | Unclear | High | High | Low | Low | Low |
| Cayrol, 2016 | High | Low | High | High | Low | Low | High |
| Chiang, 2020 | Low | Unclear | High | High | Low | Low | High |
| Corkum, 2018 | High | Unclear | Unclear | High | Low | Unclear | Low |
| Dadeh, 2021 | Unclear | Unclear | Unclear | Unclear | Low | Unclear | High |
| Diaz Moreno, 2012 | Low | High | Unclear | Unclear | Low | Unclear | Unclear |
| Doraiswamy, 1979 | High | Unclear | Unclear | High | Low | Low | Unclear |
| Esparaz, 2019 | High | Unclear | High | High | Low | Low | Low |
| Fleischman, 2013 | High | Unclear | Unclear | High | Low | Low | Low |
| Goldman, 2008 | Low | Unclear | High | High | Low | Low | High |
| Gonzalez, 2016 | Low | High | High | High | Low | High | Low |
| Greer, 2019 | High | Unclear | High | High | Low | Low | High |
| Groselj-Grenc, 2007 | Low | High | Unclear | High | Low | Low | High |
| Hsiao, 2005 | High | Unclear | Unclear | High | Low | Low | Unclear |
| Huckins, 2013 | High | Low | High | High | High | Low | High |
| Huckins, 2016 | High | Low | Low | High | High | Low | Low |
| Kaiser, 2018 | High | Unclear | High | High | Low | Unclear | High |
| Kashtan, 2020 | Unclear | Unclear | High | High | Low | Low | Low |
| Khan, 2012 | Low | High | Unclear | Unclear | Low | High | Low |
| Khanafer, 2016 | High | Unclear | Unclear | High | Low | Low | Unclear |
| Kharbanda, 2005 | High | Unclear | Unclear | High | Low | Low | Low |
| Kharbanda, 2012 | Low | High | High | High | Low | Low | Low |
| Kharbanda, 2012 | High | Unclear | Unclear | High | Low | Low | Low |
| Klein, 2021 | High | Unclear | High | High | Low | Low | High |
| Ko, 1995 | Low | Unclear | Unclear | High | Low | Low | Unclear |
| Kouame, 2005 | Low | Unclear | Unclear | Unclear | Low | Low | Unclear |
| Kumar, 2021 | High | Unclear | Low | High | Low | Low | Low |
| Kwan, 2010 | Low | High | Unclear | High | Low | Low | Low |
| Lin, 2009 | Low | Unclear | High | High | Low | High | High |
| Lycopoulou, 2005 | High | Unclear | High | High | Low | Low | High |

For peer review only

| | | | | | | | |
|---|---|---|---|---|---|---|---|
| Malia, 2019 | High | High | High | High | High | Unclear | Low |
| Mandeville, 2011 | High | Unclear | Low | High | Low | Low | Low |
| Miguez, 2015 | Low | High | High | High | Low | High | High |
| Mohammed, 2004 | Low | Unclear | Unclear | High | Low | Low | Unclear |
| Naqvi, 2019 | High | High | Unclear | High | Unclear | High | Low |
| Oikonomopoulou, 2019 | Low | High | High | High | Low | High | Low |
| Ovrebo, 1993 | Unclear | High | High | High | Low | Low | High |
| Ozguner, 2014 | High | High | High | High | Low | High | High |
| Peltola, 1986 | Low | Unclear | Unclear | High | Low | Low | Unclear |
| Prada-Arias, 2017 | Low | Unclear | Unclear | High | Low | Low | Low |
| Rodriguez, 2014 | High | Unclear | Low | High | Low | Low | Low |
| Salo, 2016 | High | Unclear | Unclear | High | Low | Low | Unclear |
| Samuel, 2002 | Low | Unclear | Unclear | High | Low | Low | Unclear |
| Sanchez, 1998 | Low | High | Unclear | High | Unclear | Unclear | Unclear |
| Shommu, 2018 | High | High | High | High | High | Unclear | High |
| Thompson, 2015 | Low | Unclear | High | High | Low | Unclear | High |
| Thompson, 2017 | Unclear | Unclear | High | High | Low | Unclear | High |
| Tuncer, 2019 | High | High | High | High | Low | Unclear | Unclear |
| Turkyilmaz, 2006 | Low | Unclear | Unclear | High | Low | Low | Unclear |
| Van Den Broek, 2004 | Unclear | Unclear | High | High | Low | Low | High |
| Wang, 2007 | Low | Unclear | Unclear | High | Low | Low | Unclear |
| Wu, 2012 | Unclear | High | Unclear | High | Low | High | Low |
| Yap, 2015 | Low | Unclear | Unclear | High | Low | Low | Unclear |
| Yazici, 2010 | High | Unclear | High | High | Low | Unclear | High |
| Zouari, 2016 | Low | Unclear | Unclear | High | Low | Unclear | Low |
| Zuniga, 2012 | High | Unclear | High | High | Low | Low | High |

For peer review only


For peer review only - http://bmjopen.bmj.com/site/about/guidelines.xhtml

**Supplementary Table 5:** Pooled estimates of diagnostic accuracy for blood tests used for diagnosis of appendicitis in children calculated using the bivariate model and reporting likelihood ratios(*Median prevalence of appendicitis in the included 54 cohort studies is 40%. Sensitivity analysis was performed for all tests at all cut offs, except procalcitonin as number of studies was too low, and identified significant (p<0.05) effects in: †WCC 10,000-15,000cells/ul for case-control study design (p=0·01, sensitivity 0·70[0·57-0·84] and specificity 0·49[0·31-0·66] n=3 case-control studies vs sensitivity 0·76[0·72-0·80] and specificity 0·72[0·68-0·76] n=27 non case-control studies))

| Marker | Threshold | Studies | Number of patients | Pooled sensitivity (95% CI) | Pooled specificity (95% CI) | Pooled LR+ (95% CI) | Pooled LR- (95% CI) | Hypothetical cohort of 1000 children with appendicitis prevalence of 40%* | |
|---|---|---|---|---|---|---|---|---|---|
| | | | | | | | | Missed appendicitis (FN) | Unnecessary treatment (FP) |
| CRP (mg/L) | ≤5 | 14 | 3754 | 0·82 (0·72 – 0·89) | 0·46 (0·28 – 0·66) | 1·52 (1·14 – 2·03) | 0·39 (0·30 – 0·51) | 112 | 324 |
| | 5-10 | 16 | 3170 | 0·73 (0·67 – 0·80) | 0·67 (0·58 – 0·75) | 2·21 (1·75 – 2·79) | 0·40 (0·33 – 0·50) | 108 | 198 |
| | 10-50 | 19 | 4470 | 0·53 (0·45 – 0·62) | 0·79 (0·74 – 0·83) | 2·53 (1·99 – 3·21) | 0·59 (0·49 – 0·71) | 188 | 126 |
| | ≥50 | 10 | 2654 | 0·35 (0·25 – 0·46) | 0·89 (0·87 – 0·91) | 3·25 (2·63 – 4·01) | 0·73 (0·63 – 0·84) | 260 | 66 |
| WCC (Cells/µl) | ≤10,000 | 28 | 11791 | 0·87 (0·83 – 0·89) | 0·58 (0·53 – 0·63) | 2·08 (1·87 – 2·31) | 0·23 (0·19 – 0·27) | 52 | 252 |
| | 10,000 - 15,000† | 30 | 9967 | 0·76 (0·72 – 0·79) | 0·70 (0·65 – 0·75) | 2·56 (2·19 – 3·00) | 0·35 (0·30 – 0·40) | 96 | 180 |
| | ≥15,000 | 14 | 6406 | 0·50 (0·44 – 0·55) | 0·84 (0·80 – 0·87) | 3·12 (2·74 – 3·55) | 0·60 (0·55 – 0·66) | 200 | 96 |
| Neutrophils (% of lymphocytes) | 65% - 80% | 13 | 3316 | 0·81 (0·74 – 0·86) | 0·64 (0·55 – 0·72) | 2·23 (1·76 – 2·82) | 0·30 (0·22 – 0·41) | 76 | 216 |
| | 75% | 9 | 1642 | 0·78 (0·73-0·82) | 0·61 (0·53-0·69) | 2·02 (1·61-2·53) | 0·36 (0·28-0·46) | 88 | 234 |
| ANC (Cells/µl) | 6600 - 10000 | 22 | 11966 | 0·85 (0·81 – 0·88) | 0·63 (0·58 – 0·68) | 2·29 (2·06 – 2·56) | 0·23 (0·19 – 0·28) | 60 | 222 |
| | <7500 | 5 | 3834 | 0·90 (0·85 – 0·94) | 0·54 (0·49 – 0·58) | 1·95 (1·79 – 2·13) | 0·18 (0·12 – 0·27) | 40 | 276 |
| | 7500 | 11 | 4730 | 0·86 (0·82 – 0·90) | 0·60 (0·54 – 0·65) | 2·14 (1·90 – 2·42) | 0·23 (0·17 – 0·30) | 56 | 240 |
| | >7500 | 5 | 3388 | 0·73 (0·70 – 0·77) | 0·77 (0·70 – 0·82) | 3·13 (2·35 – 4·16) | 0·35 (0·29 – 0·41) | 108 | 138 |
| Procalcitonin (ng/ml) | 0·1 – 0·5 | 5 | 540 | 0·31 (0·25 – 0·37) | 0·82 (0·70 – 0·90) | 1·72 (0·99 – 3·00) | 0·84 (0·74 – 0·96) | 276 | 108 |



For peer review only - http://bmjopen.bmj.com/site/about/guidelines.xhtml

**Supplementary Figures and legends:**

**Supplementary Figure 1:** Funnel plots outputs for testing individual blood tests for publication bias(68). (Procalcitonin/ WCC and CRP/ WCC,ANC and CRP not shown as study numbers were low [n=4, p=0·2]. Testing was also performed for individual cut-offs of WCC and CRP with all results >0·1 [data not shown]. Y axis = inverse root of effective sample size. X axis = Odds ratio. 95% Confidence intervals for bias: CRP -6.10 – 15.03; WCC -5.26 – 7.15; Neutrophils % -13.72 – 17.74; ANC -4.18 – 11.87)

**WCC**

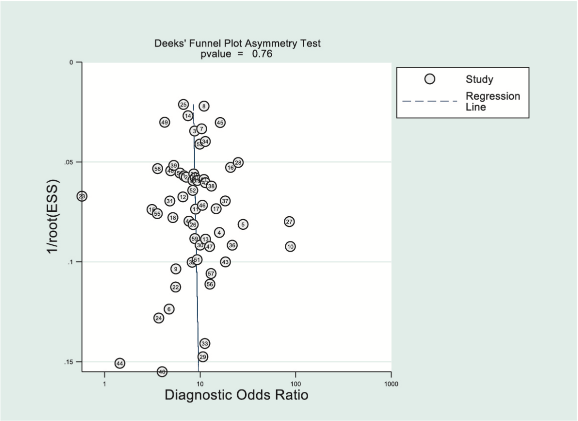


**CRP**

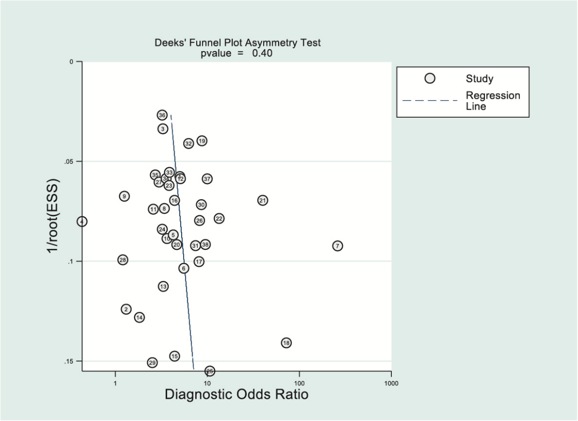


**Neutrophils (%)**

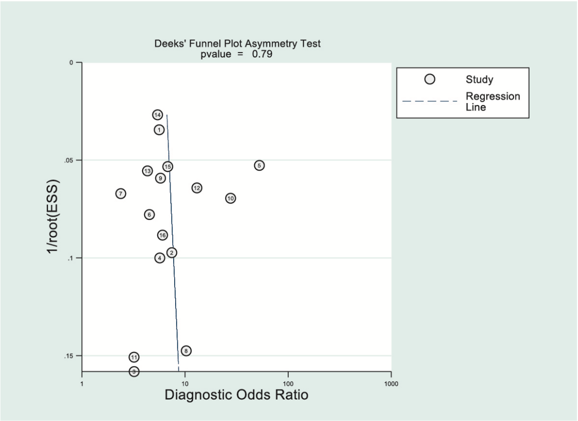


**Absolute neutrophil count**

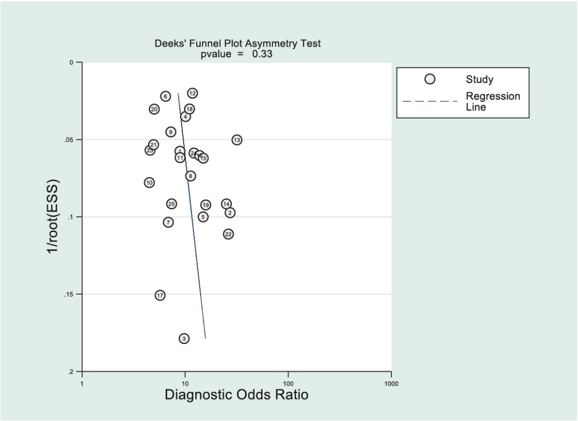

**Supplementary Figure 2:** Forest plot showing all studies reporting diagnostic accuracy of WCC, split by reported cut off, in paediatric appendicitis. (Sensitivity and specificity for each study represented visually (blue square) with 95% confidence interval (line)).

**WCC at or below 10,000 /mm^3**

| Study | TP | FP | FN | TN | Threshold | Sensitivity (95% CI) | Specificity (95% CI) |
|---|---|---|---|---|---|---|---|
| Kharbanda 2005 | 153 | 162 | 4 | 106 | 8.85 | 0.97 [0.94, 0.99] | 0.40 [0.34, 0.46] |
| Kharbanda 2012 | 58 | 67 | 0 | 49 | 8.85 | 1.00 [0.94, 1.00] | 0.42 [0.33, 0.52] |
| Anadalwar 2015 | 348 | 212 | 45 | 240 | 9.0 | 0.89 [0.85, 0.92] | 0.53 [0.48, 0.58] |
| Kashtan 2020 | 857 | 681 | 117 | 622 | 9.0 | 0.88 [0.86, 0.90] | 0.48 [0.45, 0.50] |
| Akgul 2021 | 173 | 78 | 17 | 52 | 10.0 | 0.91 [0.86, 0.95] | 0.40 [0.32, 0.49] |
| Andersson 1999 | 42 | 37 | 6 | 84 | 10.0 | 0.88 [0.75, 0.95] | 0.69 [0.60, 0.77] |
| Andersson 2008 | 17 | 35 | 4 | 39 | 10.0 | 0.81 [0.58, 0.95] | 0.53 [0.41, 0.64] |
| Andersson 2014 | 52 | 61 | 2 | 66 | 10.0 | 0.96 [0.87, 1.00] | 0.52 [0.43, 0.61] |
| Andersson 2017 | 303 | 339 | 30 | 348 | 10.0 | 0.91 [0.87, 0.94] | 0.51 [0.47, 0.54] |
| Bachur 2016 | 786 | 581 | 84 | 682 | 10.0 | 0.90 [0.88, 0.92] | 0.54 [0.51, 0.57] |
| Benito 2016 | 79 | 45 | 10 | 51 | 10.0 | 0.89 [0.80, 0.94] | 0.53 [0.43, 0.63] |
| Cayrol 2016 | 46 | 33 | 6 | 49 | 10.0 | 0.88 [0.77, 0.96] | 0.60 [0.48, 0.70] |
| Chiang 2020 | 625 | 319 | 93 | 354 | 10.0 | 0.87 [0.84, 0.89] | 0.53 [0.49, 0.56] |
| Esparaz 2019 | 51 | 111 | 5 | 160 | 10.0 | 0.91 [0.80, 0.97] | 0.59 [0.53, 0.65] |
| Fleischman 2013 | 53 | 52 | 12 | 61 | 10.0 | 0.82 [0.70, 0.90] | 0.54 [0.44, 0.63] |
| Goldman 2008 | 98 | 84 | 14 | 112 | 10.0 | 0.88 [0.80, 0.93] | 0.57 [0.50, 0.64] |
| Gonzalez Del Castillo 2016 | 93 | 18 | 79 | 131 | 10.0 | 0.54 [0.46, 0.62] | 0.88 [0.82, 0.93] |
| Kaiser 2018 | 29 | 11 | 10 | 14 | 10.0 | 0.74 [0.58, 0.87] | 0.56 [0.35, 0.76] |
| Khanafer 2016 | 46 | 51 | 8 | 75 | 10.0 | 0.85 [0.73, 0.93] | 0.60 [0.50, 0.68] |
| Ko 1995 | 23 | 7 | 4 | 13 | 10.0 | 0.85 [0.66, 0.96] | 0.65 [0.41, 0.85] |
| Lycopoulou 2005 | 32 | 4 | 10 | 14 | 10.0 | 0.76 [0.61, 0.88] | 0.78 [0.52, 0.94] |
| Malia 2019 | 169 | 113 | 56 | 424 | 10.0 | 0.75 [0.69, 0.81] | 0.79 [0.75, 0.82] |
| Mandeville 2011 | 129 | 49 | 26 | 83 | 10.0 | 0.83 [0.76, 0.89] | 0.63 [0.54, 0.71] |
| Ozgunur 2014 | 32 | 12 | 2 | 3 | 10.0 | 0.94 [0.80, 0.99] | 0.20 [0.04, 0.48] |
| Samuel 2002 | 624 | 113 | 110 | 323 | 10.0 | 0.85 [0.82, 0.88] | 0.74 [0.70, 0.78] |
| Shommu 2018 | 41 | 32 | 5 | 49 | 10.0 | 0.89 [0.76, 0.96] | 0.60 [0.49, 0.71] |
| Thompson 2015 | 425 | 56 | 71 | 46 | 10.0 | 0.86 [0.82, 0.89] | 0.45 [0.35, 0.55] |
| Thompson 2017 | 334 | 243 | 137 | 424 | 10.0 | 0.71 [0.67, 0.75] | 0.64 [0.60, 0.67] |
| van den Broek 2004 | 52 | 8 | 13 | 26 | 10.0 | 0.80 [0.68, 0.89] | 0.76 [0.59, 0.89] |
| Yap 2015 | 102 | 126 | 17 | 129 | 10.0 | 0.86 [0.78, 0.91] | 0.51 [0.44, 0.57] |
| Yazici 2010 | 162 | 39 | 21 | 18 | 10.0 | 0.89 [0.83, 0.93] | 0.32 [0.20, 0.45] |
| Zuniga 2012 | 26 | 37 | 2 | 36 | 10.0 | 0.93 [0.76, 0.99] | 0.49 [0.37, 0.61] |

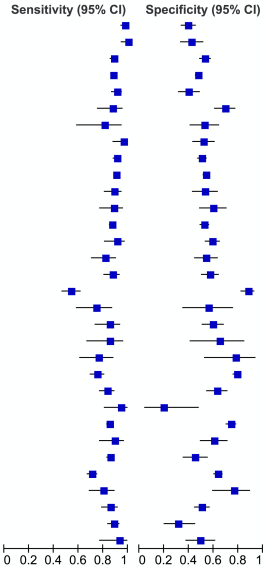


**WCC above 10,000 and below 15,000 /mm^3**

| Study | TP | FP | FN | TN | Threshold | Sensitivity (95% CI) | Specificity (95% CI) |
|---|---|---|---|---|---|---|---|
| Altali 2017 | 92 | 75 | 24 | 140 | 10.5 | 0.79 [0.71, 0.86] | 0.65 [0.58, 0.71] |
| Groselj-Grenc 2007 | 36 | 11 | 13 | 22 | 11.0 | 0.73 [0.59, 0.85] | 0.67 [0.48, 0.82] |
| Hsiao 2005 | 86 | 95 | 25 | 16 | 11.0 | 0.77 [0.69, 0.85] | 0.14 [0.08, 0.22] |
| Mohammed 2004 | 109 | 19 | 21 | 67 | 11.0 | 0.84 [0.76, 0.90] | 0.78 [0.68, 0.86] |
| Ovrebo 1993 | 116 | 34 | 89 | 138 | 11.0 | 0.57 [0.50, 0.63] | 0.80 [0.73, 0.86] |
| Prada Arias 2017 | 118 | 61 | 14 | 82 | 11.0 | 0.89 [0.83, 0.94] | 0.57 [0.49, 0.66] |
| Wu 2012 | 245 | 83 | 61 | 205 | 11.0 | 0.80 [0.75, 0.84] | 0.71 [0.66, 0.76] |
| Kaiser 2018 | 22 | 4 | 17 | 21 | 11.6 | 0.56 [0.40, 0.72] | 0.84 [0.64, 0.95] |
| Andersson 1999 | 28 | 23 | 20 | 98 | 12.0 | 0.58 [0.43, 0.72] | 0.81 [0.73, 0.88] |
| Andersson 2008 | 12 | 22 | 9 | 52 | 12.0 | 0.57 [0.34, 0.78] | 0.70 [0.59, 0.80] |
| Andersson 2014 | 42 | 44 | 12 | 83 | 12.0 | 0.78 [0.64, 0.88] | 0.65 [0.56, 0.74] |
| Andersson 2017 | 268 | 211 | 65 | 476 | 12.0 | 0.80 [0.76, 0.85] | 0.69 [0.66, 0.73] |
| Bachur 2016 | 686 | 409 | 184 | 854 | 12.0 | 0.79 [0.76, 0.82] | 0.68 [0.65, 0.70] |
| Gonzalez Del Castillo 2016 | 74 | 52 | 37 | 158 | 12.0 | 0.67 [0.57, 0.75] | 0.75 [0.69, 0.81] |
| Khanafer 2016 | 35 | 34 | 19 | 92 | 12.0 | 0.65 [0.51, 0.77] | 0.73 [0.64, 0.81] |
| Kwan 2010 | 82 | 32 | 33 | 62 | 12.0 | 0.71 [0.62, 0.79] | 0.66 [0.55, 0.75] |
| Ozgunur 2014 | 29 | 8 | 5 | 7 | 12.0 | 0.85 [0.69, 0.95] | 0.47 [0.21, 0.73] |
| Shommu 2018 | 37 | 21 | 9 | 60 | 12.0 | 0.80 [0.66, 0.91] | 0.74 [0.63, 0.83] |
| Thompson 2015 | 380 | 42 | 116 | 60 | 12.0 | 0.77 [0.73, 0.80] | 0.59 [0.49, 0.68] |
| Thompson 2017 | 281 | 161 | 190 | 506 | 12.0 | 0.60 [0.55, 0.64] | 0.76 [0.72, 0.79] |
| Miguez 2016 | 35 | 14 | 9 | 78 | 12.3 | 0.80 [0.65, 0.90] | 0.85 [0.76, 0.91] |
| Beltran 2007 | 67 | 8 | 4 | 42 | 12.5 | 0.94 [0.86, 0.98] | 0.84 [0.71, 0.93] |
| Diaz Moreno 2012 | 59 | 42 | 26 | 58 | 12.8 | 0.69 [0.58, 0.79] | 0.58 [0.48, 0.68] |
| Rodriguez Sanchez 2013 | 36 | 18 | 5 | 46 | 13.0 | 0.88 [0.74, 0.96] | 0.72 [0.59, 0.82] |
| Tuncer 2019 | 104 | 43 | 33 | 121 | 13.05 | 0.76 [0.68, 0.83] | 0.74 [0.66, 0.80] |
| Oikonomopoulou 2018 | 74 | 33 | 26 | 152 | 13.3 | 0.74 [0.64, 0.82] | 0.82 [0.76, 0.87] |
| Bal 2016 | 38 | 11 | 18 | 29 | 13.4 | 0.68 [0.54, 0.80] | 0.72 [0.56, 0.85] |
| Greer 2019 | 63 | 92 | 23 | 368 | 13.5 | 0.73 [0.63, 0.82] | 0.80 [0.76, 0.84] |
| Kumar 2021 | 25 | 5 | 30 | 60 | 14.0 | 0.45 [0.32, 0.59] | 0.92 [0.83, 0.97] |
| Sanchez-Echaniz 1997 | 73 | 25 | 21 | 76 | 14.0 | 0.78 [0.68, 0.86] | 0.75 [0.66, 0.83] |
| Peltola 1986 | 56 | 11 | 38 | 57 | 14.9 | 0.60 [0.49, 0.70] | 0.84 [0.73, 0.92] |

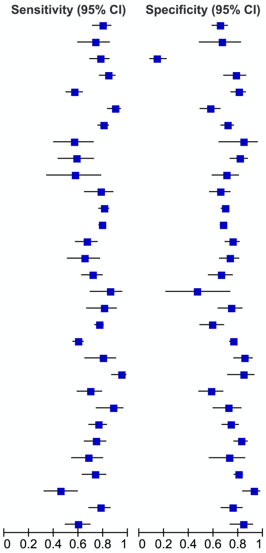


**WCC at or above 15,000 /mm^3**

| Study | TP | FP | FN | TN | Threshold | Sensitivity (95% CI) | Specificity (95% CI) |
|---|---|---|---|---|---|---|---|
| Andersson 1999 | 16 | 5 | 32 | 116 | 15.0 | 0.33 [0.20, 0.48] | 0.96 [0.91, 0.99] |
| Andersson 2008 | 9 | 10 | 12 | 64 | 15.0 | 0.43 [0.22, 0.66] | 0.86 [0.77, 0.93] |
| Andersson 2014 | 20 | 17 | 34 | 110 | 15.0 | 0.37 [0.24, 0.51] | 0.87 [0.79, 0.92] |
| Andersson 2017 | 159 | 103 | 174 | 584 | 15.0 | 0.48 [0.42, 0.53] | 0.85 [0.82, 0.88] |
| Bachur 2016 | 485 | 240 | 385 | 1023 | 15.0 | 0.56 [0.52, 0.59] | 0.81 [0.79, 0.83] |
| Calvo Rigual 1998 | 96 | 22 | 45 | 68 | 15.0 | 0.68 [0.60, 0.76] | 0.76 [0.65, 0.84] |
| Gonzalez Del Castillo 2016 | 44 | 67 | 28 | 182 | 15.0 | 0.61 [0.49, 0.72] | 0.73 [0.67, 0.78] |
| Kaiser 2018 | 17 | 3 | 22 | 22 | 15.0 | 0.44 [0.28, 0.60] | 0.88 [0.69, 0.97] |
| Khanafer 2016 | 27 | 19 | 27 | 107 | 15.0 | 0.50 [0.36, 0.64] | 0.85 [0.77, 0.91] |
| Lin 2009 | 23 | 4 | 30 | 43 | 15.0 | 0.43 [0.30, 0.58] | 0.91 [0.80, 0.98] |
| Ozgunur 2014 | 19 | 8 | 15 | 7 | 15.0 | 0.56 [0.38, 0.73] | 0.47 [0.21, 0.73] |
| Shommu 2018 | 30 | 11 | 16 | 70 | 15.0 | 0.65 [0.50, 0.79] | 0.86 [0.77, 0.93] |
| Thompson 2015 | 252 | 24 | 244 | 78 | 15.0 | 0.51 [0.46, 0.55] | 0.76 [0.67, 0.84] |
| Thompson 2017 | 195 | 82 | 276 | 585 | 15.0 | 0.41 [0.37, 0.46] | 0.88 [0.85, 0.90] |

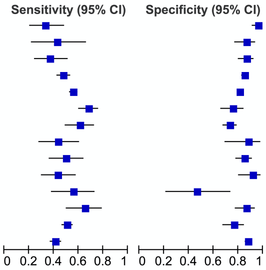


**WCC threshold depending on age**

| Study | TP | FP | FN | TN | Threshold | Sensitivity (95% CI) | Specificity (95% CI) |
|---|---|---|---|---|---|---|---|
| Doraiswamy 1979 | 94 | 5 | 131 | 145 | | 0.42 [0.35, 0.49] | 0.97 [0.92, 0.99] |
| Klein 2021 | 40 | 21 | 13 | 60 | | 0.75 [0.62, 0.86] | 0.74 [0.63, 0.83] |
| Salo 2016 | 13 | 11 | 9 | 11 | | 0.59 [0.36, 0.79] | 0.50 [0.28, 0.72] |
| Turkyilmaz 2006 | 43 | 9 | 18 | 35 | | 0.70 [0.57, 0.81] | 0.80 [0.65, 0.90] |
| Wang 2007 | 50 | 67 | 24 | 269 | | 0.68 [0.56, 0.78] | 0.80 [0.75, 0.84] |

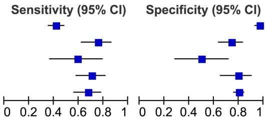


**WCC threshold depending on duration pain**

| Study | TP | FP | FN | TN | Threshold | Sensitivity (95% CI) | Specificity (95% CI) |
|---|---|---|---|---|---|---|---|
| Dadeh 2021 | 73 | 434 | 24 | 512 | | 0.75 [0.65, 0.83] | 0.54 [0.51, 0.57] |

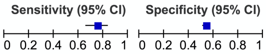




For peer review only - http://bmjopen.bmj.com/site/about/guidelines.xhtml

**Supplementary Figure 3:** Forest plot showing all studies reporting diagnostic accuracy of CPR, split by reported cut off, in paediatric appendicitis. (Sensitivity and specificity for each study represented visually (blue square) with 95% confidence interval (line)).

**CRP at or below 5 mg/l**

| Study | TP | FP | FN | TN | Threshold | Sensitivity (95% CI) | Specificity (95% CI) |
|---|---|---|---|---|---|---|---|
| Bal 2016 | 50 | 24 | 6 | 16 | 0.9 | 0.89 [0.78, 0.96] | 0.40 [0.25, 0.57] |
| Miguez 2016 | 30 | 29 | 14 | 63 | 3.0 | 0.68 [0.52, 0.81] | 0.68 [0.58, 0.78] |
| Lycopoulou 2005 | 34 | 1 | 8 | 17 | 4.5 | 0.81 [0.66, 0.91] | 0.94 [0.73, 1.00] |
| Akgul 2021 | 146 | 72 | 44 | 59 | 5.0 | 0.77 [0.70, 0.83] | 0.45 [0.36, 0.54] |
| Altali 2017 | 92 | 93 | 24 | 122 | 5.0 | 0.79 [0.71, 0.86] | 0.57 [0.50, 0.63] |
| Andersson 1999 | 29 | 34 | 17 | 85 | 5.0 | 0.63 [0.48, 0.77] | 0.71 [0.62, 0.79] |
| Andersson 2008 | 17 | 55 | 4 | 17 | 5.0 | 0.81 [0.58, 0.95] | 0.24 [0.14, 0.35] |
| Andersson 2014 | 55 | 127 | 0 | 0 | 5.0 | 1.00 [0.94, 1.00] | 0.00 [0.00, 0.03] |
| Andersson 2017 | 285 | 481 | 38 | 211 | 5.0 | 0.88 [0.84, 0.92] | 0.30 [0.27, 0.34] |
| Chiang 2020 | 589 | 394 | 129 | 279 | 5.0 | 0.82 [0.79, 0.85] | 0.41 [0.38, 0.45] |
| Gonzalez Del Castillo 2016 | 88 | 90 | 23 | 120 | 5.0 | 0.79 [0.71, 0.86] | 0.57 [0.50, 0.64] |
| Greer 2019 | 69 | 133 | 17 | 327 | 5.0 | 0.80 [0.70, 0.88] | 0.71 [0.67, 0.75] |
| Kaiser 2018 | 23 | 11 | 16 | 14 | 5.0 | 0.59 [0.42, 0.74] | 0.56 [0.35, 0.76] |
| Malia 2019 | 160 | 118 | 65 | 419 | 5.0 | 0.71 [0.65, 0.77] | 0.78 [0.74, 0.81] |
| Ozgunur 2014 | 32 | 9 | 2 | 6 | 5.0 | 0.94 [0.80, 0.99] | 0.40 [0.16, 0.68] |
| Rodriguez Sanchez 2013 | 21 | 29 | 21 | 35 | 5.0 | 0.50 [0.34, 0.66] | 0.55 [0.42, 0.67] |
| Yap 2015 | 99 | 143 | 20 | 112 | 5.0 | 0.83 [0.75, 0.89] | 0.44 [0.38, 0.50] |

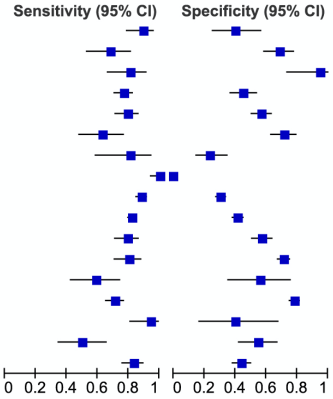


**CRP between 5 and 11 mg/l**

| Study | TP | FP | FN | TN | Threshold | Sensitivity (95% CI) | Specificity (95% CI) |
|---|---|---|---|---|---|---|---|
| Zouari 2016 | 107 | 74 | 32 | 79 | 6.0 | 0.77 [0.69, 0.84] | 0.52 [0.43, 0.60] |
| Naqvi 2019 | 64 | 20 | 15 | 63 | 7.9 | 0.81 [0.71, 0.89] | 0.76 [0.65, 0.85] |
| Mohammed 2004 | 114 | 13 | 16 | 73 | 8.0 | 0.88 [0.81, 0.93] | 0.85 [0.76, 0.92] |
| Groselj-Grenc 2007 | 36 | 15 | 13 | 18 | 9.0 | 0.73 [0.59, 0.85] | 0.55 [0.36, 0.72] |
| Ko 1995 | 19 | 7 | 8 | 13 | 9.0 | 0.70 [0.50, 0.86] | 0.65 [0.41, 0.85] |
| Andersson 1999 | 23 | 26 | 23 | 93 | 10.0 | 0.50 [0.35, 0.65] | 0.78 [0.70, 0.85] |
| Andersson 2008 | 15 | 25 | 6 | 47 | 10.0 | 0.71 [0.48, 0.89] | 0.65 [0.53, 0.76] |
| Andersson 2014 | 43 | 53 | 12 | 74 | 10.0 | 0.78 [0.65, 0.88] | 0.58 [0.49, 0.67] |
| Andersson 2017 | 221 | 249 | 102 | 443 | 10.0 | 0.68 [0.63, 0.73] | 0.64 [0.60, 0.68] |
| Gonzalez Del Castillo 2016 | 75 | 70 | 36 | 140 | 10.0 | 0.68 [0.58, 0.76] | 0.67 [0.60, 0.73] |
| Kaiser 2018 | 20 | 10 | 19 | 15 | 10.0 | 0.51 [0.35, 0.68] | 0.60 [0.39, 0.79] |
| Lin 2009 | 39 | 12 | 14 | 35 | 10.0 | 0.74 [0.60, 0.85] | 0.74 [0.60, 0.86] |
| Lycopoulou 2005 | 26 | 1 | 16 | 17 | 10.0 | 0.62 [0.46, 0.76] | 0.94 [0.73, 1.00] |
| Ovrebo 1993 | 48 | 80 | 5 | 27 | 10.0 | 0.91 [0.79, 0.97] | 0.25 [0.17, 0.35] |
| Ozgunur 2014 | 25 | 7 | 9 | 8 | 10.0 | 0.74 [0.56, 0.87] | 0.53 [0.27, 0.79] |
| Peltola 1986 | 60 | 12 | 34 | 56 | 10.0 | 0.64 [0.53, 0.73] | 0.82 [0.71, 0.91] |
| Kumar 2021 | 49 | 30 | 6 | 35 | 10.5 | 0.89 [0.78, 0.96] | 0.54 [0.41, 0.66] |

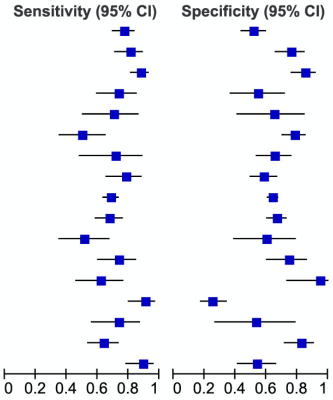


**CRP between 11 and 50 mg/l**

| Study | TP | FP | FN | TN | Threshold | Sensitivity (95% CI) | Specificity (95% CI) |
|---|---|---|---|---|---|---|---|
| Oikonomopoulou 2018 | 60 | 52 | 40 | 133 | 12.5 | 0.60 [0.50, 0.70] | 0.72 [0.65, 0.78] |
| Salo 2016 | 14 | 9 | 8 | 13 | 15.0 | 0.64 [0.41, 0.83] | 0.59 [0.36, 0.79] |
| Ozgunur 2014 | 24 | 5 | 10 | 10 | 16.0 | 0.71 [0.53, 0.85] | 0.67 [0.38, 0.88] |
| Diaz Moreno 2012 | 46 | 31 | 39 | 68 | 18.0 | 0.54 [0.43, 0.65] | 0.69 [0.59, 0.78] |
| Benito 2016 | 31 | 13 | 58 | 83 | 20.0 | 0.35 [0.25, 0.46] | 0.86 [0.78, 0.93] |
| Calvo Rigual 1998 | 61 | 34 | 80 | 56 | 20.0 | 0.43 [0.35, 0.52] | 0.62 [0.51, 0.72] |
| Shommu 2018 | 24 | 9 | 24 | 67 | 20.0 | 0.50 [0.35, 0.65] | 0.88 [0.79, 0.94] |
| Wu 2012 | 214 | 78 | 92 | 210 | 25.0 | 0.70 [0.64, 0.75] | 0.73 [0.67, 0.78] |
| Prada Arias 2017 | 57 | 29 | 75 | 114 | 25.2 | 0.43 [0.35, 0.52] | 0.80 [0.72, 0.86] |
| Andersson 1999 | 14 | 14 | 32 | 105 | 30.0 | 0.30 [0.18, 0.46] | 0.88 [0.81, 0.93] |
| Andersson 2008 | 8 | 11 | 13 | 61 | 30.0 | 0.38 [0.18, 0.62] | 0.85 [0.74, 0.92] |
| Andersson 2014 | 31 | 35 | 24 | 92 | 30.0 | 0.56 [0.42, 0.70] | 0.72 [0.64, 0.80] |
| Andersson 2017 | 140 | 128 | 183 | 564 | 30.0 | 0.43 [0.38, 0.49] | 0.82 [0.78, 0.84] |
| Cayrol 2016 | 20 | 12 | 32 | 70 | 30.0 | 0.38 [0.25, 0.53] | 0.85 [0.76, 0.92] |
| Gonzalez Del Castillo 2016 | 59 | 40 | 52 | 170 | 30.0 | 0.53 [0.43, 0.63] | 0.81 [0.75, 0.86] |
| Kaiser 2018 | 13 | 8 | 26 | 17 | 30.0 | 0.33 [0.19, 0.50] | 0.68 [0.46, 0.85] |
| Kwan 2010 | 81 | 33 | 34 | 61 | 30.0 | 0.70 [0.61, 0.79] | 0.65 [0.54, 0.74] |
| Sanchez-Echaniz 1997 | 40 | 8 | 54 | 93 | 30.0 | 0.43 [0.32, 0.53] | 0.92 [0.85, 0.97] |
| Beltran 2007 | 68 | 4 | 3 | 46 | 47.0 | 0.96 [0.88, 0.99] | 0.92 [0.81, 0.98] |

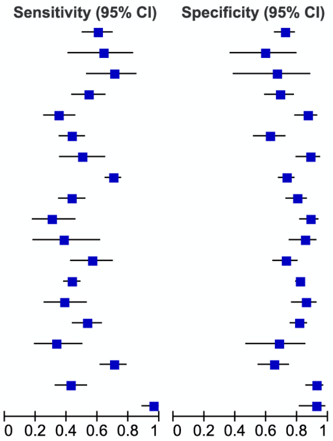


**CRP at or above 50 mg/l**

| Study | TP | FP | FN | TN | Threshold | Sensitivity (95% CI) | Specificity (95% CI) |
|---|---|---|---|---|---|---|---|
| Andersson 1999 | 7 | 10 | 39 | 109 | 50.0 | 0.15 [0.06, 0.29] | 0.92 [0.85, 0.96] |
| Andersson 2008 | 5 | 7 | 16 | 65 | 50.0 | 0.24 [0.08, 0.47] | 0.90 [0.81, 0.96] |
| Andersson 2014 | 20 | 21 | 35 | 106 | 50.0 | 0.36 [0.24, 0.50] | 0.83 [0.76, 0.89] |
| Andersson 2017 | 93 | 62 | 230 | 630 | 50.0 | 0.29 [0.24, 0.34] | 0.91 [0.89, 0.93] |
| Gonzalez Del Castillo 2016 | 37 | 28 | 74 | 182 | 50.0 | 0.33 [0.25, 0.43] | 0.87 [0.81, 0.91] |
| Kaiser 2018 | 9 | 2 | 30 | 23 | 50.0 | 0.23 [0.11, 0.39] | 0.92 [0.74, 0.99] |
| Ko 1995 | 14 | 1 | 13 | 19 | 50.0 | 0.52 [0.32, 0.71] | 0.95 [0.75, 1.00] |
| Ozgunur 2014 | 15 | 3 | 19 | 12 | 50.0 | 0.44 [0.27, 0.62] | 0.80 [0.52, 0.96] |
| Shommu 2018 | 12 | 1 | 36 | 75 | 50.0 | 0.25 [0.14, 0.40] | 0.99 [0.93, 1.00] |
| Wu 2012 | 214 | 49 | 92 | 239 | 89.0 | 0.70 [0.64, 0.75] | 0.83 [0.78, 0.87] |

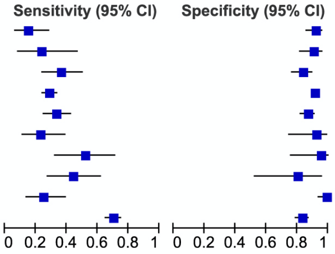



For peer review only - http://bmjopen.bmj.com/site/about/guidelines.xhtml

**Supplementary Figure 4:** Forest plot showing all studies reporting diagnostic accuracy of ANC in paediatric appendicitis. (Sensitivity and specificity for each study represented visually (blue square) with 95% confidence interval (line)).

| Study | TP | FP | FN | TN | Threshold | Sensitivity (95% CI) | Specificity (95% CI) |
|---|---|---|---|---|---|---|---|
| Salo 2016 | 16 | 7 | 6 | 15 | age dependent | 0.73 [0.50, 0.89] | 0.68 [0.45, 0.86] |
| Altali 2017 | 99 | 85 | 17 | 130 | 6.6 | 0.85 [0.78, 0.91] | 0.60 [0.54, 0.67] |
| Kharbanda 2012 | 942 | 824 | 76 | 783 | 6.73 | 0.93 [0.91, 0.94] | 0.49 [0.46, 0.51] |
| Fleischman 2013 | 53 | 56 | 12 | 57 | 6.75 | 0.82 [0.70, 0.90] | 0.50 [0.41, 0.60] |
| Kharbanda 2005 | 152 | 131 | 5 | 137 | 6.75 | 0.97 [0.93, 0.99] | 0.51 [0.45, 0.57] |
| Prada Arias 2017 | 119 | 57 | 13 | 86 | 7.0 | 0.90 [0.84, 0.95] | 0.60 [0.52, 0.68] |
| Akgul 2021 | 163 | 74 | 27 | 56 | 7.5 | 0.86 [0.80, 0.90] | 0.43 [0.34, 0.52] |
| Andersson 1999 | 34 | 38 | 2 | 61 | 7.5 | 0.94 [0.81, 0.99] | 0.62 [0.51, 0.71] |
| Andersson 2008 | 8 | 27 | 1 | 33 | 7.5 | 0.89 [0.52, 1.00] | 0.55 [0.42, 0.68] |
| Andersson 2014 | 35 | 32 | 3 | 41 | 7.5 | 0.92 [0.79, 0.98] | 0.56 [0.44, 0.68] |
| Andersson 2017 | 274 | 282 | 31 | 322 | 7.5 | 0.90 [0.86, 0.93] | 0.53 [0.49, 0.57] |
| Benito 2016 | 78 | 37 | 11 | 59 | 7.5 | 0.88 [0.79, 0.94] | 0.61 [0.51, 0.71] |
| Goldman 2008 | 84 | 67 | 17 | 121 | 7.5 | 0.83 [0.74, 0.90] | 0.64 [0.57, 0.71] |
| Greer 2019 | 69 | 115 | 17 | 345 | 7.5 | 0.80 [0.70, 0.88] | 0.75 [0.71, 0.79] |
| Samuel 2002 | 595 | 122 | 139 | 314 | 7.5 | 0.81 [0.78, 0.84] | 0.72 [0.68, 0.76] |
| Shommu 2018 | 40 | 24 | 6 | 57 | 7.5 | 0.87 [0.74, 0.95] | 0.70 [0.59, 0.80] |
| Thompson 2015 | 351 | 68 | 47 | 45 | 7.5 | 0.88 [0.85, 0.91] | 0.40 [0.31, 0.49] |
| Thompson 2017 | 319 | 206 | 141 | 457 | 7.5 | 0.69 [0.65, 0.74] | 0.69 [0.65, 0.72] |
| Zuniga 2012 | 27 | 37 | 1 | 36 | 7.5 | 0.96 [0.82, 1.00] | 0.49 [0.37, 0.61] |
| Corkum 2018 | 116 | 166 | 40 | 416 | 8.0 | 0.74 [0.67, 0.81] | 0.71 [0.68, 0.75] |
| Bal 2016 | 43 | 13 | 13 | 27 | 9.4 | 0.77 [0.64, 0.87] | 0.68 [0.51, 0.81] |
| Miguez 2016 | 37 | 16 | 7 | 76 | 9.6 | 0.84 [0.70, 0.93] | 0.83 [0.73, 0.90] |
| Bachur 2016 | 630 | 365 | 240 | 898 | 10.0 | 0.72 [0.69, 0.75] | 0.71 [0.69, 0.74] |
| Kumar 2021 | 28 | 8 | 27 | 57 | 10.48 | 0.51 [0.37, 0.65] | 0.88 [0.77, 0.95] |
| Oikonomopoulou 2018 | 71 | 26 | 29 | 159 | 10.8 | 0.71 [0.61, 0.80] | 0.86 [0.80, 0.91] |

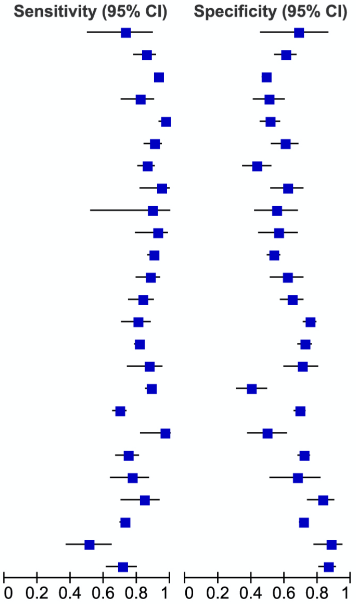


**Supplementary Figure 5:** Forest plot showing all studies reporting diagnostic accuracy of Neutrophil count as a percentage in paediatric appendicitis. (Sensitivity and specificity for each study represented visually (blue square) with 95% confidence interval (line)).

| Study | TP | FP | FN | TN | Threshold | Sensitivity (95% CI) | Specificity (95% CI) |
|---|---|---|---|---|---|---|---|
| Anadalwar 2015 | 340 | 241 | 53 | 211 | 65% | 0.87 [0.83, 0.90] | 0.47 [0.42, 0.51] |
| Salo 2016 | 16 | 10 | 6 | 12 | 71% | 0.73 [0.50, 0.89] | 0.55 [0.32, 0.76] |
| Andersson 1999 | 31 | 45 | 5 | 54 | 75% | 0.86 [0.71, 0.95] | 0.55 [0.44, 0.65] |
| Andersson 2008 | 9 | 29 | 3 | 31 | 75% | 0.75 [0.43, 0.95] | 0.52 [0.38, 0.65] |
| Andersson 2014 | 30 | 29 | 8 | 44 | 75% | 0.79 [0.63, 0.90] | 0.60 [0.48, 0.72] |
| Chiang 2020 | 524 | 224 | 194 | 449 | 75% | 0.73 [0.70, 0.76] | 0.67 [0.63, 0.70] |
| Dadeh 2021 | 74 | 304 | 23 | 642 | 75% | 0.76 [0.67, 0.84] | 0.68 [0.65, 0.71] |
| Fleischman 2013 | 50 | 48 | 15 | 65 | 75% | 0.77 [0.65, 0.86] | 0.58 [0.48, 0.67] |
| Hsiao 2005 | 87 | 67 | 24 | 44 | 75% | 0.78 [0.70, 0.86] | 0.40 [0.30, 0.49] |
| Klein 2021 | 36 | 21 | 17 | 60 | 75% | 0.68 [0.54, 0.80] | 0.74 [0.63, 0.83] |
| Ko 1995 | 22 | 6 | 5 | 14 | 75% | 0.81 [0.62, 0.94] | 0.70 [0.46, 0.88] |
| Mandeville 2011 | 119 | 48 | 36 | 84 | 75% | 0.77 [0.69, 0.83] | 0.64 [0.55, 0.72] |
| Mohammed 2004 | 111 | 15 | 19 | 71 | 75% | 0.85 [0.78, 0.91] | 0.83 [0.73, 0.90] |
| Yap 2015 | 79 | 80 | 40 | 175 | 75% | 0.66 [0.57, 0.75] | 0.69 [0.63, 0.74] |
| Wang 2007 | 44 | 34 | 30 | 302 | 80% | 0.59 [0.47, 0.71] | 0.90 [0.86, 0.93] |
| Doraiswamy 1979 | 216 | 47 | 9 | 103 | age dependent | 0.96 [0.93, 0.98] | 0.69 [0.61, 0.76] |

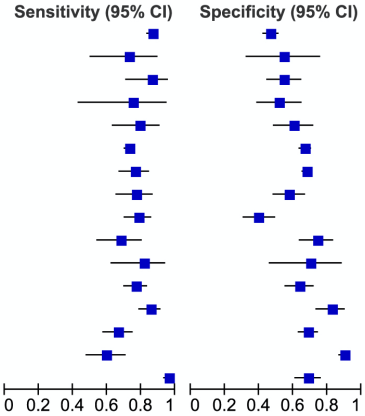


**Supplementary Figure 6:** Forest plot showing all studies reporting diagnostic accuracy of procalcitonin in paediatric appendicitis. (Sensitivity and specificity for each study represented visually (blue square) with 95% confidence interval (line)).

| Study | TP | FP | FN | TN | Threshold | Sensitivity (95% CI) | Specificity (95% CI) |
|---|---|---|---|---|---|---|---|
| Benito 2016 | 34 | 34 | 55 | 62 | 0.1 | 0.38 [0.28, 0.49] | 0.65 [0.54, 0.74] |
| Bal 2016 | 18 | 4 | 38 | 36 | 0.37 | 0.32 [0.20, 0.46] | 0.90 [0.76, 0.97] |
| Khan 2012 | 6 | 2 | 16 | 26 | 0.39 | 0.27 [0.11, 0.50] | 0.93 [0.76, 0.99] |
| Rodriguez Sanchez 2013 | 10 | 18 | 34 | 46 | 0.5 | 0.23 [0.11, 0.38] | 0.72 [0.59, 0.82] |
| Kouame 2005 | 19 | 4 | 49 | 29 | 0.5 | 0.28 [0.18, 0.40] | 0.88 [0.72, 0.97] |
| Akgul 2021 | 55 | 19 | 135 | 111 | 0.5 | 0.29 [0.23, 0.36] | 0.85 [0.78, 0.91] |

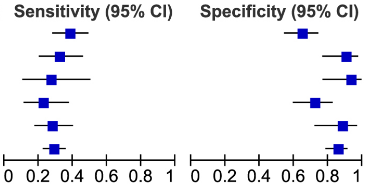




For peer review only - http://bmjopen.bmj.com/site/about/guidelines.xhtml

**Supplementary Figure 7:** Forest plot showing all studies reporting diagnostic accuracy of combination of WCC and/or CRP in paediatric appendicitis. (Sensitivity and specificity for each study represented visually (blue square) with 95% confidence interval (line)).

**CRP and WCC**

| Study | TP | FP | FN | TN | Threshold WCC - CRP | Sensitivity (95% CI) | Specificity (95% CI) |
|---|---|---|---|---|---|---|---|
| Andersson 1999 | 20 | 12 | 26 | 107 | 10.0 - 10 | 0.43 [0.29, 0.59] | 0.90 [0.83, 0.95] |
| Andersson 2008 | 12 | 16 | 9 | 56 | 10.0 - 10 | 0.57 [0.34, 0.78] | 0.78 [0.66, 0.87] |
| Andersson 2014 | 39 | 31 | 14 | 96 | 10.0 - 10 | 0.74 [0.60, 0.85] | 0.76 [0.67, 0.83] |
| Andersson 2017 | 197 | 150 | 122 | 521 | 10.0 - 10 | 0.62 [0.56, 0.67] | 0.78 [0.74, 0.81] |
| Calvo Rigual 1998 | 113 | 45 | 28 | 45 | 15.0 - 20 | 0.80 [0.73, 0.86] | 0.50 [0.39, 0.61] |
| Kaiser 2018 | 16 | 7 | 23 | 18 | 10.0 - 10 | 0.41 [0.26, 0.58] | 0.72 [0.51, 0.88] |
| Kwan 2010 | 48 | 8 | 67 | 86 | 12.0 - 30 | 0.42 [0.33, 0.51] | 0.91 [0.84, 0.96] |
| Ovrebo 1993 | 38 | 30 | 14 | 77 | 11.0- 10 | 0.73 [0.59, 0.84] | 0.72 [0.62, 0.80] |
| Ozgunur 2014 | 24 | 5 | 10 | 10 | 10.0 - 10 | 0.71 [0.53, 0.85] | 0.67 [0.38, 0.88] |
| Peltola 1986 | 42 | 20 | 52 | 48 | 14.9 - 100 | 0.45 [0.34, 0.55] | 0.71 [0.58, 0.81] |
| Sanchez-Echaniz 1997 | 32 | 5 | 62 | 96 | 14.0 - 30 | 0.34 [0.25, 0.45] | 0.95 [0.89, 0.98] |

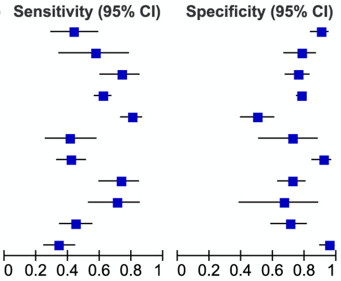


**CRP or WCC**

| Study | TP | FP | FN | TN | Threshold WCC - CRP | Sensitivity (95% CI) | Specificity (95% CI) |
|---|---|---|---|---|---|---|---|
| Andersson 1999 | 44 | 50 | 2 | 69 | 10.0 - 10 | 0.96 [0.85, 0.99] | 0.58 [0.49, 0.67] |
| Andersson 2008 | 20 | 43 | 1 | 29 | 10.0 - 10 | 0.95 [0.76, 1.00] | 0.40 [0.29, 0.53] |
| Andersson 2014 | 53 | 83 | 0 | 44 | 10.0 - 10 | 1.00 [0.93, 1.00] | 0.35 [0.26, 0.44] |
| Andersson 2017 | 313 | 425 | 6 | 246 | 10.0 - 10 | 0.98 [0.96, 0.99] | 0.37 [0.33, 0.40] |
| Chiang 2020 | 716 | 514 | 2 | 159 | 10.0 - 5 | 1.00 [0.99, 1.00] | 0.24 [0.20, 0.27] |
| Huckins 2013 | 140 | 238 | 4 | 121 | 10.0 - 8 | 0.97 [0.93, 0.99] | 0.34 [0.29, 0.39] |
| Huckins 2016 | 457 | 775 | 19 | 635 | 10.0 - 8 | 0.96 [0.94, 0.98] | 0.45 [0.42, 0.48] |
| Kaiser 2018 | 33 | 14 | 6 | 11 | 10.0 - 10 | 0.85 [0.69, 0.94] | 0.44 [0.24, 0.65] |
| Ovrebo 1993 | 62 | 86 | 0 | 21 | 11.0- 10 | 1.00 [0.94, 1.00] | 0.20 [0.13, 0.28] |
| Ozgunur 2014 | 33 | 14 | 1 | 1 | 10.0 - 10 | 0.97 [0.85, 1.00] | 0.07 [0.00, 0.32] |

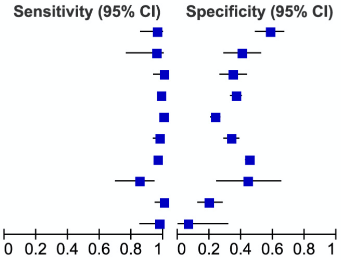


**Supplementary Figure 8:** Forest plot showing all studies reporting diagnostic accuracy of combination of WCC, CRP and/or NC in paediatric appendicitis. (Sensitivity and specificity for each study represented visually (blue square) with 95% confidence interval (line)).

**WCC, CRP, and Neutrophils**

| Study | TP | FP | FN | TN | Threshold WCC - CRP - NC% | Sensitivity (95% CI) | Specificity (95% CI) |
|---|---|---|---|---|---|---|---|
| Andersson 1999 | 15 | 11 | 20 | 88 | 10.0 - 10 - 75% | 0.43 [0.26, 0.61] | 0.89 [0.81, 0.94] |
| Andersson 2008 | 6 | 11 | 6 | 48 | 10.0 - 10 - 75% | 0.50 [0.21, 0.79] | 0.81 [0.69, 0.90] |
| Andersson 2014 | 21 | 12 | 17 | 61 | 10.0 - 10 - 75% | 0.55 [0.38, 0.71] | 0.84 [0.73, 0.91] |
| Andersson 2017 | 139 | 89 | 152 | 498 | 10.0 - 10 - 75% | 0.48 [0.42, 0.54] | 0.85 [0.82, 0.88] |
| Mohammed 2004 | 112 | 8 | 18 | 78 | 11.0 - 80 - 75% | 0.86 [0.79, 0.92] | 0.91 [0.82, 0.96] |

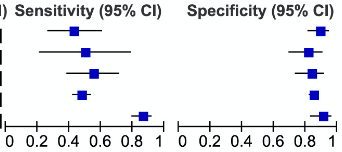


**WCC, CRP, or Neutrophils**

| Study | TP | FP | FN | TN | Threshold WCC - CRP - NC% | Sensitivity (95% CI) | Specificity (95% CI) |
|---|---|---|---|---|---|---|---|
| Yap 2015 | 119 | 192 | 0 | 63 | 10.0 - 5 - 75% | 1.00 [0.97, 1.00] | 0.25 [0.20, 0.30] |

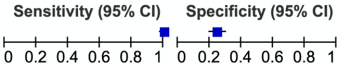



For peer review only - http://bmjopen.bmj.com/site/about/guidelines.xhtml

**Search Strategy:**

Searches were performed on the 15th March 2022 with search terms that had been informed by scoping searches. Databases searched included Cochrane Database of Systematic Reviews, Cochrane Central Register of controlled trials, Database of abstracts of Reviews of Effects, Embase, Ovid(Medline) Epub Ahead of print in process and other Non-Indexed Citations, Ovid Medline(R) Daily, Ovid Medline(R) and Science Citation Index and Conference Proceedings Citation Index (SCIENCE). These databases were searched through the interfaces Cochrane Library(Wiley), OvidSP and Web of Science Core Collection. Specific terms used for these interfaces are detailed below, following searching all results were merged and checked for duplicates before review (**Figure 1**).

**Medline**:

| # | Searches | Results |
|---|---|---|
| 1 | adolescent/ or exp child/ or exp infant/ | 3410061 |
| 2 | (child* or schoolchild* or preschool* or pre-school* or boys or girls or infant? or pediatric* or paediatric* or adolescen* or teen* or youth?).ti,ab. | 1876871 |
| 3 | 1 or 2 | 3876495 |
| 4 | exp Leukocyte Count/ or leukocytes/ or neutrophils/ | 225318 |
| 5 | (leukocyte? or white cell or white blood cell or neutrophil*).ti,ab. | 272998 |
| 6 | (wbcc or wcc).ti,ab. | 655 |
| 7 | C-Reactive Protein/ | 42976 |
| 8 | (c-reactive protein? or crp).ti,ab. | 75114 |
| 9 | Calcitonin/ | 15622 |
| 10 | (procalcitonin or calcitonin or pct).ti,ab. | 32717 |
| 11 | 4 or 5 or 6 or 7 or 8 or 9 or 10 | 490743 |
| 12 | Appendicitis/ | 18470 |
| 13 | Appendectomy/ | 10586 |
| 14 | (appendix or appendicitis or appendectom* or appendicectom*).ti,ab. | 33426 |
| 15 | 12 or 13 or 14 | 38889 |
| 16 | 3 and 11 and 15 | 787 |

**Embase:**

| # | Searches | Results |
|---|---|---|
| 1 | juvenile/ or exp adolescent/ or exp child/ | 3172209 |
| 2 | (child* or schoolchild* or preschool* or pre-school* or boys or girls or infant? or pediatric* or paediatric* or adolescen* or teen* or youth?).ti,ab. | 2298797 |
| 3 | 1 or 2 | 3780916 |
| 4 | exp leukocyte count/ or leukocyte/ or neutrophil/ | 394999 |
| 5 | (leukocyte? or white cell or white blood cell or neutrophil*).ti,ab. | 364068 |
| 6 | (wbcc or wcc).ti,ab. | 1603 |
| 7 | C reactive protein/ | 158251 |
| 8 | (c-reactive protein? or crp).ti,ab. | 129235 |
| 9 | Procalcitonin/ | 10738 |
| 10 | (procalcitonin or calcitonin or pct).ti,ab. | 43836 |

For peer review only

| | | |
|---|---|---|
| 11 | 4 or 5 or 6 or 7 or 8 or 9 or 10 | 749518 |
| 12 | exp appendicitis/ | 22927 |
| 13 | appendectomy/ | 18892 |
| 14 | (appendix or appendicitis or appendectom* or appendicectom*).ti,ab. | 37059 |
| 15 | 12 or 13 or 14 | 46062 |
| 16 | 3 and 11 and 15 | 1369 |

**Cochrane:**

| # | Searches |
|---|---|
| 1 | MeSH descriptor: [Adolescent] explode all trees |
| 2 | MeSH descriptor: [Child] explode all trees |
| 3 | MeSH descriptor: [Infant] explode all trees |
| 4 | exp leukocyte count/ or leukocyte/ or neutrophil/ |
| 5 | child* or schoolchild* or preschool* or pre-school* or boys or girls or infant? or pediatric* or paediatric* or adolescen* or teen* or youth?:ti,ab,kw (Word variations have been searched) |
| 6 | #1 or #2 or #3 or #4 |
| 7 | MeSH descriptor: [Leukocyte Count] explode all trees |
| 8 | MeSH descriptor: [Leukocytes] this term only |
| 9 | MeSH descriptor: [Neutrophils] explode all trees |
| 10 | wbcc or wcc:ti,ab,kw (Word variations have been searched) |
| 11 | MeSH descriptor: [C-Reactive Protein] explode all trees |
| 12 | c-reactive protein* or crp:ti,ab,kw (Word variations have been searched) |
| 13 | MeSH descriptor: [Calcitonin] explode all trees |
| 14 | procalcitonin or calcitonin or pct:ti,ab,kw |
| 15 | #6 or #7 or #8 or #9 or #10 or #11 or #12 or #13 or #14 |
| 16 | MeSH descriptor: [Appendicitis] explode all trees |
| 17 | MeSH descriptor: [Appendectomy] explode all trees |
| 18 | appendix or appendicitis or appendectom* or appendicectom*:ti,ab,kw (Word variations have been searched) |
| 19 | #16 or #17 or #18 |
| 20 | #5 and #15 and #19 |

**Web of Science:**

| # | Searches | Results |
|---|---|---|
| 1 | TS=(child* or schoolchild* or preschool* or pre-school* or boys or girls or infant? or pediatric* or paediatric* or adolescen* or teen* or youth?) | 1709598 |
| 2 | TS=(leukocyte* or "white cell" or "white blood cell" or neutrophil*) OR TS=("c-reactive protein" OR "c reactive protein" OR crp) OR TS=(procalcitonin OR calcitonin OR pct) | 433876 |
| 3 | TS=(appendix or appendicitis or appendectom* or appendicectom*) | 33245 |
| 4 | #3 AND #2 AND #1 | 397 |


For peer review only - http://bmjopen.bmj.com/site/about/guidelines.xhtml

**QUADAS-2 Scoring criteria**

Below is an overview, with examples of how the QUADAS-2 scoring criteria was used to measure risk of bias and applicability in selected studies(69). In those patients not undergoing surgery the optimal reference standard was follow up after 2 weeks to confirm absence of disease. Studies including only patients who had undergone a radiological investigation were included but this caveat was noted when assessing these studies for risk of bias. Study quality was not an exclusion criteria.

Domain 1: Patient Selection

- Was a consecutive or random sample of patients enrolled? (Yes/Unclear/No)
  - *Scored yes if the words in article state "consecutive", "random", or "all patients were included*
- Was a case-control design avoided? (Yes/Unclear/No)
  - *Scored yes if article stated the study design as not being case-control*
- Did the study avoid inappropriate exclusions? (Yes/Unclear/No)
  - *Scored yes if studies excluded no children with abdominal pain or suspected appendicitis.*
- (Risk) Could the selection of patients have introduced bias? (Low / Unclear/ High)
  - *Scored low if all answers to signalling questions were yes. Score high if any were answered no. Score unclear if any were answered as unclear with remainder scoring low.*
- (Concern) Is there concern that included patients do not match the review question? (Low / Unclear /High)
  - *Scored low if all patients were <18 years, had symptoms in keeping with appendicitis and no previous diagnosis of appendicitis*

Domain 2: Index Test

- Were the index tests interpreted without knowledge of the results of the reference standard? (Yes / Unclear/ No)
  - *Scored yes if text stated blood test results were concealed or blinded to clinician or pathologist.*
- If a threshold was used, was it pre-specified? (Yes / Unclear/ No)
  - *Scored yes if a cut-off for index test was stated before commencing study or analysing results*
- (Risk) Could the conduct or interpretation of the index test have introduced bias? (Low / Unclear/ High)
  - *Scored low if all answers to signalling questions were yes. Score high if any were answered no. Score unclear if any were answered as unclear with remainder scoring low.*
- (Concern) Is there concern that the index test, its conduct, or interpretation differ from the review question? (Low / Unclear / High)
  - *Scored low if the study provides a clear description of the index test and a clear description or definition of a positive test result*

Domain 3: Reference Standard

- Is the reference standard likely to correctly classify the target condition? (Yes / Unclear/ No)
  - *Score yes if the reference standard was histology after surgery or clinical follow up of patient after 2 weeks. Score no if the reference standard was surgery alone without histology.*
- Were the reference standard results interpreted without knowledge of the results of the index test? (Yes / Unclear/ No)
  - *Score yes if study states the assessment of reference standard was done without knowledge or with blinding of the results of the index test*
- (Risk) Could the reference standard, its conduct, or its interpretation have introduced bias? (Low / Unclear/ High)
  - *Scored low if all answers to signalling questions were yes. Score high if any were answered no. Score unclear if any were answered as unclear with remainder scoring low.*
- (Concern) Is there concern that the target condition as defined by the reference standard does not match the review question? (Low / Unclear/ High)
  - *Score Low if all patients were in keeping with the target condition of appendicitis as defined by histology after surgery or refuted by clinical follow-up of greater than or equal to 2 weeks*

Domain 4: Flow and Timing

- Was there an appropriate interval between index test(s) and reference standard? (Yes / Unclear/ No)
  - *Score yes if the time period was 48 hours or less and was stated in text; Score no if the time period was more than 48 hours. Score unclear if insufficient information to determine the interval between index test and reference standard.*

For peer review only

- Did all patients receive a reference standard? (Yes / Unclear/ No)
    - *Score yes if all patients received either appendicectomy with histology or clinical follow up of greater than or equal to 2 weeks to refute appendicitis*
- Did patients receive the same reference standard? (Yes / Unclear/ No)
    - *Score yes if all patients received the same reference standard.*
- Were all patients included in the analysis? (Yes / Unclear/ No)
    - *Score yes if all patients included at patient selection underwent index test and reference standard.*
- (Risk) Could the patient flow have introduced bias? (Low / Unclear/ High)
    - *Scored low if all answers to signalling questions were yes. Score high if any were answered no. Score unclear if any were answered as unclear with remainder scoring low.*

For peer review only


For peer review only - http://bmjopen.bmj.com/site/about/guidelines.xhtml

**Supplementary References**


1. Akgül F, Er A, Ulusoy E, Çağlar A, Çitlenbik H, Keskinoğlu P, et al. Integration of Physical Examination, Old and New Biomarkers, and Ultrasonography by Using Neural Networks for Pediatric Appendicitis. Pediatr Emerg Care 2021 Dec 1;37(12):e1075-e1081.
2. Altali K, Ruiz-Artacho P, Trenchs V, Martinez Ortiz de Zarate M, Navarro C, Fernandez C, et al. [Hospital emergency room diagnosis of acute appendicitis in patients aged 2 to 20 years: the INFURG-SEMES score from the emergency infections study of the Spanish Society of Emergency Medicine]. Emergencias : revista de la Sociedad Espanola de Medicina de Emergencias. 2017;29(4):231-6.
3. Anandalwar SP, Callahan MJ, Bachur RG, Feng C, Sidhwa F, Karki M, et al. Use of White Blood Cell Count and Polymorphonuclear Leukocyte Differential to Improve the Predictive Value of Ultrasound for Suspected Appendicitis in Children. Journal of the American College of Surgeons. 2015;220(6):1010-7.
4. Andersson RE, Hugander AP, Ghazi SH, Ravn H, Offenbartl SK, Nystrom PO, et al. Diagnostic value of disease history, clinical presentation, and inflammatory parameters of appendicitis. World J Surg. 1999;23(2):133-40.
5. Andersson M, Andersson RE. The appendicitis inflammatory response score: a tool for the diagnosis of acute appendicitis that outperforms the Alvarado score. World J Surg. 2008;32(8):1843-9.
6. Andersson M, Rubér M, Ekerfelt C, Hallgren HB, Olaison G, Andersson RE. Can New Inflammatory Markers Improve the Diagnosis of Acute Appendicitis? World Journal of Surgery. 2014;38(11):2777-83.
7. Andersson M, Kolodziej B, Andersson RE. Randomized clinical trial of Appendicitis Inflammatory Response score-based management of patients with suspected appendicitis. The British journal of surgery. 2017;104(11):1451-61.
8. Bachur RG, Dayan PS, Dudley NC, Bajaj L, Stevenson MD, Macias CG, et al. The Influence of Age on the Diagnostic Performance of White Blood Cell Count and Absolute Neutrophil Count in Suspected Pediatric Appendicitis. Academic emergency medicine : official journal of the Society for Academic Emergency Medicine. 2016;23(11):1235-42.
9. Bal A, Anil M, Narturk M, Ozdemir T, Arikan A, Koyluoglu G, et al. Importance of Clinical Decision Making by Experienced Pediatric Surgeons When Children Are Suspected of Having Acute Appendicitis: The Reality in a High-Volume Pediatric Emergency Department. Pediatr Emerg Care. 2017;33(9):e38-e42.
10. Beltrán A M, Almonacid F J, Gutiérrez C J, Cruces B K. Puntuación diagnóstica de apendicitis aguda en niños realizada por pediatras de las Unidades de Emergencia. Revista chilena de pediatría. 2007;78:584-91.
11. Benito J, Acedo Y, Medrano L, Barcena E, Garay RP, Arri EA. Usefulness of new and traditional serum biomarkers in children with suspected appendicitis. The American journal of emergency medicine. 2016;34(5):871-6.
12. Calvo Rigual F, Sendra Esteve S, Mialaret Lahiguera A, Montagud Beltrán E, Llanes Domingo S, Medrano González J. [The value of C-reactive protein in the diagnosis of acute appendicitis in children]. An Esp Pediatr. 1998;48(4):376-80.
13. Cayrol J, Miguez MC, Guerrero G, Tomatis C, Simal I, Maranon R. Diagnostic accuracy and prognostic utility of D Dimer in acute appendicitis in children. European journal of pediatrics. 2016;175(3):313-20.
14. Chiang JJY, Angus MI, Nah SA, Jacobsen AS, Low Y, Choo CSC, et al. Time course response of inflammatory markers in pediatric appendicitis. Pediatr Surg Int 2020 Apr;36(4):493-500.
15. Corkum KS, Oyetunji TA, Grabowski JE, Rigsby CK, Lautz TB. Absolute neutrophil count as a diagnostic guide for the use of MRI in the workup of suspected appendicitis in children. Journal of pediatric surgery. 2019;54(7):1359-64.
16. Dadeh AA, Puitong K. Predictive Factors to Diagnose Appendicitis in Children in the Emergency Department. Open Access Emerg Med 2021 Jul 29;13:363-372.
17. Doraiswamy NV. Leucocyte counts in the diagnosis and prognosis of acute appendicitis in children. The British journal of surgery. 1979;66(11):782-4.
18. Esparaz JR, McGovern GC, Mowrer AR, Nierstedt RT, Biesboer EA, Elger BM, et al. A simple algorithm to improve quality while reducing resource utilization in evaluation of suspected appendicitis in children. American journal of surgery. 2019;217(3):469-72.
19. Fleischman RJ, Devine MK, Yagapen MA, Steichen AJ, Hansen ML, Zigman AF, et al. Evaluation of a novel pediatric appendicitis pathway using high- and low-risk scoring systems. Pediatr Emerg Care. 2013;29(10):1060-5.
20. Goldman RD, Carter S, Stephens D, Antoon R, Mounstephen W, Langer JC. Prospective validation of the pediatric appendicitis score. The Journal of pediatrics. 2008;153(2):278-82.
21. Gonzalez Del Castillo J, Ayuso FJ, Trenchs V, Martinez Ortiz de Zarate M, Navarro C, Altali K, et al. Diagnostic accuracy of the APPY1 Test in patients aged 2-20 years with suspected acute appendicitis presenting to emergency departments. Emergency medicine journal : EMJ. 2016;33(12):853-9.


For peer review only

22. Greer D, Bennett P, Wagstaff B, Croaker D. Lymphopaenia in the diagnosis of paediatric appendicitis: a false sense of security? ANZ J Surg 2019 Sep;89(9):1122-1125.
23. Groselj-Grenc M, Repse S, Dolenc-Strazar Z, Hojker S, Derganc M. Interleukin-6 and lipopolysaccharide-binding protein in acute appendicitis in children. Scandinavian journal of clinical and laboratory investigation. 2007;67(2):197-206.
24. Hsiao KH, Lin LH, Chen DF. Application of the MANTRELS scoring system in the diagnosis of acute appendicitis in children. Acta paediatrica Taiwanica = Taiwan er ke yi xue hui za zhi. 2005;46(3):128-31.
25. Huckins DS, Simon HK, Copeland K, Spiro DM, Gogain J, Wandell M. A novel biomarker panel to rule out acute appendicitis in pediatric patients with abdominal pain. The American journal of emergency medicine. 2013;31(9):1368-75.
26. Huckins DS, Simon HK, Copeland K, Milling TJ, Jr., Spandorfer PR, Hennes H, et al. Prospective validation of a biomarker panel to identify pediatric ED patients with abdominal pain who are at low risk for acute appendicitis. The American journal of emergency medicine. 2016;34(8):1373-82.
27. Kaiser M, Schroeckenfuchs M, Castellani C, Warncke G, Till H, Singer G. The diagnostic value of hepcidin to predict the presence and severity of appendicitis in children. The Journal of surgical research. 2018;222:102-7.
28. Kashtan M, Graham D, Anandalwar S, Hills-Dunlap J, Rangel S. Influence of symptom duration and WBC profile on the negative predictive value of a nondiagnostic ultrasound in children with suspected appendicitis. J Pediatr Surg 2020 Jun;55(6):1032-1036.
29. Khan AN, Sawan A, Likourezos A, Schnellinger M, Garcia E. The usefulness of procalcitonin in the diagnosis of appendicitis in children: a pilot study. Emergency medicine international. 2012;2012:317504.
30. Khanafer I, Martin DA, Mitra TP, Eccles R, Brindle ME, Nettel-Aguirre A, et al. Test characteristics of common appendicitis scores with and without laboratory investigations: a prospective observational study. BMC Pediatr. 2016;16(1):147.
31. Kharbanda AB, Taylor GA, Fishman SJ, Bachur RG. A clinical decision rule to identify children at low risk for appendicitis. Pediatrics. 2005;116(3):709-16.
32. Kharbanda AB, Rai AJ, Cosme Y, Liu K, Dayan PS. Novel serum and urine markers for pediatric appendicitis. Academic emergency medicine : official journal of the Society for Academic Emergency Medicine. 2012;19(1):56-62.
33. Kharbanda AB, Dudley NC, Bajaj L, Stevenson MD, Macias CG, Mittal MK, et al. Validation and refinement of a prediction rule to identify children at low risk for acute appendicitis. Archives of pediatrics & adolescent medicine. 2012;166(8):738-44.
34. Klein TT, Kohn E, Klin B, Ziv-Baran T, Kozer E, Berkovitch M, et al. sTREM-1 as a diagnostic biomarker for acute appendicitis in children. Asian J Surg 2021 Sep;44(9):1172-1178.
35. Ko YS, Lin LH, Chen DF. Laboratory aid and ultrasonography in the diagnosis of appendicitis in children. Zhonghua Minguo xiao er ke yi xue hui za zhi [Journal] Zhonghua Minguo xiao er ke yi xue hui. 1995;36(6):415-9.
36. Kouame DB, Garrigue MA, Lardy H, Machet MC, Giraudeau B, Robert M. [Is procalcitonin able to help in pediatric appendicitis diagnosis?]. Annales de chirurgie. 2005;130(3):169-74.
37. Kumar MSV, Tiwari MK, Singh J, Malik A. Plasma Fibrinogen: An Independent Predictor of Pediatric Appendicitis. J Indian Assoc Pediatr Surg 2021 Jul-Aug;26(4):240-245.
38. Kwan KY, Nager AL. Diagnosing pediatric appendicitis: usefulness of laboratory markers. The American journal of emergency medicine. 2010;28(9):1009-15.
39. Lin CH, Chen JH, Li TC, Ho YJ, Lin WC. Children presenting at the emergency department with right lower quadrant pain. The Kaohsiung journal of medical sciences. 2009;25(1):1-9.
40. Lycopoulou L, Mamoulakis C, Hantzi E, Demetriadis D, Antypas S, Giannaki M, et al. Serum amyloid A protein levels as a possible aid in the diagnosis of acute appendicitis in children. Clinical chemistry and laboratory medicine. 2005;43(1):49-53.
41. Malia L, Sturm JJ, Smith SR, Brown RT, Campbell B, Chicaiza H. Predictors for Acute Appendicitis in Children. Pediatr Emerg Care. 2019.
42. Mandeville K, Pottker T, Bulloch B, Liu J. Using appendicitis scores in the pediatric ED. The American journal of emergency medicine. 2011;29(9):972-7.
43. Miguez C, Tomatis Souverbielle C, Haro A, Guerrero G, Perez-Egido L, Garcia-Gamiz M, et al. Evaluation of proadrenomedullin as a diagnostic or prognostic biomarker of acute appendicitis in children. The American journal of emergency medicine. 2016;34(12):2298-305.
44. Mohammed AA, Daghman NA, Aboud SM, Oshibi HO. The diagnostic value of C-reactive protein, white blood cell count and neutrophil percentage in childhood appendicitis. Saudi medical journal. 2004;25(9):1212-5.


For peer review only - http://bmjopen.bmj.com/site/about/guidelines.xhtml

45. Diaz Moreno E, Garcia Gomez M, Castejon Casado J, Liceras Liceras E, Martin Cano F, Munoz Miguelsanz MA. [Analysis of the medical decision in abdominal pain suggestive of acute appendicitis]. Cirugia pediatrica : organo oficial de la Sociedad Espanola de Cirugia Pediatrica. 2012;25(1):40-5.
46. Naqvi SA, Thompson GC, Joffe AR, Blackwood J, Martin DA, Brindle M, et al. Cytokines and Chemokines in Pediatric Appendicitis: A Multiplex Analysis of Inflammatory Protein Mediators. Mediators of inflammation. 2019;2019:2359681.
47. Oikonomopoulou N, Miguez-Navarro C, Rivas-Garcia A, Garcia Gamiz M, Lopez-Lopez R, Oliver-Saez P, et al. Assessment of proadrenomedullin as diagnostic or prognostic biomarker of acute appendicitis in children with acute abdominal pain. The American journal of emergency medicine. 2019;37(7):1289-94.
48. Ovrebo KK, Eckerbom RM, Haram S, Rokke O. [Acute abdomen among children and adolescents. A retrospective study of 470 children and adolescents with acute abdominal pain]. Tidsskrift for den Norske laegeforening : tidsskrift for praktisk medicin, ny raekke. 1993;113(26):3244-7.
49. Ozguner I, Kizilgun M, Karaman A, Cavusoglu YH, Erdogan D, Karaman I, et al. Are neutrophil CD64 expression and interleukin-6 early useful markers for diagnosis of acute appendicitis? European journal of pediatric surgery : official journal of Austrian Association of Pediatric Surgery [et al] = Zeitschrift fur Kinderchirurgie. 2014;24(2):179-83.
50. Peltola H, Ahlqvist J, Rapola J, Rasanen J, Louhimo I, Saarinen M, et al. C-reactive protein compared with white blood cell count and erythrocyte sedimentation rate in the diagnosis of acute appendicitis in children. Acta chirurgica Scandinavica. 1986;152:55-8.
51. Prada-Arias M, Vazquez JL, Salgado-Barreira A, Gomez-Veiras J, Montero-Sanchez M, Fernandez-Lorenzo JR. Diagnostic accuracy of fibrinogen to differentiate appendicitis from nonspecific abdominal pain in children. The American journal of emergency medicine. 2017;35(1):66-70.
52. A. Rodríguez Sánchez de la Blanca RMP, J. Lorente Romero, A. Rivas García, P. Vázquez López, C. Míguez Navarro. [Usefulness of procalcitonin in early diagnosis of acute appendicitis]. Acta Pediatr Esp. 2014;72(7):4.
53. Salo M, Roth B, Stenstrom P, Arnbjornsson E, Ohlsson B. Urinary biomarkers in pediatric appendicitis. Pediatric surgery international. 2016;32(8):795-804.
54. Samuel M. Pediatric appendicitis score. Journal of pediatric surgery. 2002;37(6):877-81.
55. Sanchez Echaniz J, Luis Garcia M, Vazquez Ronco MA, Mintegui Raso S, Benito Fernandez J, Lopez Alvarez-Buhilla P. [Diagnostic value of reactive C protein in suspected acute appendicitis in children]. An Esp Pediatr. 1998;48(5):470-4.
56. Shommu NS, Jenne CN, Blackwood J, Martin DA, Joffe AR, Eccles R, et al. The Use of Metabolomics and Inflammatory Mediator Profiling Provides a Novel Approach to Identifying Pediatric Appendicitis in the Emergency Department. Scientific reports. 2018;8(1):4083.
57. Thompson GC, Schuh S, Gravel J, Reid S, Fitzpatrick E, Turner T, et al. Variation in the Diagnosis and Management of Appendicitis at Canadian Pediatric Hospitals. Academic emergency medicine : official journal of the Society for Academic Emergency Medicine. 2015;22(7):811-22.
58. Thompson GC, Morrison E, Ross M, Liu H, Vanderkooi OG, Eccles R. The Use of Routine Blood Cultures in Pediatric Appendicitis. Pediatr Emerg Care. 2017;33(12):e160-e3.
59. Tuncer AA, Cavus S, Balcioglu A, Silay S, Demiralp I, Calkan E, et al. Can mean platelet volume, Neutrophil-to-Lymphocyte, Lymphocyte-to-Monocyte, Platelet-to-Lymphocyte ratios be favourable predictors for the differential diagnosis of appendicitis? JPMA The Journal of the Pakistan Medical Association. 2019;69(5):647-54.
60. Turkyilmaz Z, Sonmez K, Karabulut R, Elbeg S, Moralioglu S, Demirtola A, et al. Sequential cytokine levels in the diagnosis of appendicitis. Scandinavian journal of clinical and laboratory investigation. 2006;66(8):723-31.
61. van den Broek WT, van der Ende ED, Bijnen AB, Breslau PJ, Gouma DJ. Which children could benefit from additional diagnostic tools in case of suspected appendicitis? Journal of pediatric surgery. 2004;39(4):570-4.
62. Wang LT, Prentiss KA, Simon JZ, Doody DP, Ryan DP. The use of white blood cell count and left shift in the diagnosis of appendicitis in children. Pediatr Emerg Care. 2007;23(2):69-76.
63. Wu HP, Chen CY, Kuo IT, Wu YK, Fu YC. Diagnostic values of a single serum biomarker at different time points compared with Alvarado score and imaging examinations in pediatric appendicitis. The Journal of surgical research. 2012;174(2):272-7.
64. Yap TL, Chen Y, Low WW, Ong CC, Nah SA, Jacobsen AS, et al. A new 2-step risk-stratification clinical score for suspected appendicitis in children. Journal of pediatric surgery. 2015;50(12):2051-5.
65. Yazici M, Ozkisacik S, Oztan MO, Gursoy H. Neutrophil/lymphocyte ratio in the diagnosis of childhood appendicitis. The Turkish journal of pediatrics. 2010;52(4):400-3.

For peer review only

66. Zouari M, Jallouli M, Louati H, Kchaou R, Chtourou R, Kotti A, et al. Predictive value of C-reactive protein, ultrasound and Alvarado score in acute appendicitis: a prospective pediatric cohort. The American journal of emergency medicine. 2016;34(2):189-92.
67. Zuniga RV, Arribas JL, Montes SP, Fernandez MN, Abad CG, Martin LG, et al. Application of Pediatric Appendicitis Score on the emergency department of a secondary level hospital. Pediatr Emerg Care. 2012;28(6):489-92.
68. Deeks JJ, Macaskill P, Irwig L. The performance of tests of publication bias and other sample size effects in systematic reviews of diagnostic test accuracy was assessed. Journal of Clinical Epidemiology. 2005;58(9):882-93.
69. Whiting PF, Rutjes AWS, Westwood ME, Mallett S, Deeks JJ, Reitsma JB, et al. QUADAS-2: A Revised Tool for the Quality Assessment of Diagnostic Accuracy Studies. Annals of Internal Medicine. 2011;155(8):529-36.

For peer review only


For peer review only - http://bmjopen.bmj.com/site/about/guidelines.xhtml

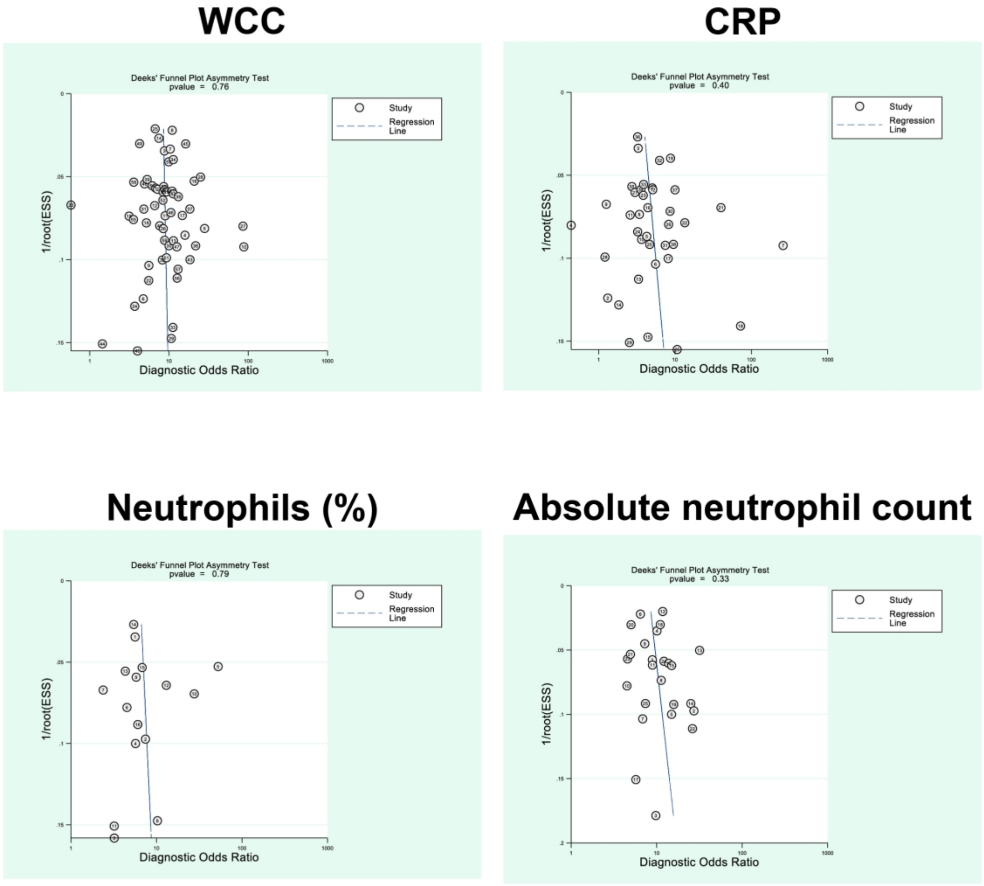


210x188mm (300 x 300 DPI)

**WCC at or below 10,000 /mm^3**

| Study | TP | FP | FN | TN | Threshold | Sensitivity (95% CI) | Specificity (95% CI) |
|---|---|---|---|---|---|---|---|
| Kharbanda 2005 | 153 | 162 | 4 | 106 | 8.85 | 0.97 [0.94, 0.99] | 0.40 [0.34, 0.46] |
| Kharbanda 2012 | 58 | 67 | 0 | 49 | 8.85 | 1.00 [0.94, 1.00] | 0.42 [0.33, 0.52] |
| Anadalwar 2015 | 348 | 212 | 45 | 240 | 9.0 | 0.89 [0.85, 0.92] | 0.53 [0.48, 0.58] |
| Kashtan 2020 | 857 | 681 | 117 | 622 | 9.0 | 0.88 [0.86, 0.90] | 0.48 [0.45, 0.50] |
| Akgul 2021 | 173 | 78 | 17 | 52 | 10.0 | 0.91 [0.86, 0.95] | 0.40 [0.32, 0.49] |
| Andersson 1999 | 42 | 37 | 6 | 84 | 10.0 | 0.88 [0.75, 0.95] | 0.69 [0.60, 0.77] |
| Andersson 2008 | 17 | 35 | 4 | 39 | 10.0 | 0.81 [0.58, 0.95] | 0.53 [0.41, 0.64] |
| Andersson 2014 | 52 | 61 | 2 | 66 | 10.0 | 0.96 [0.87, 1.00] | 0.52 [0.43, 0.61] |
| Andersson 2017 | 303 | 339 | 30 | 348 | 10.0 | 0.91 [0.87, 0.94] | 0.51 [0.47, 0.54] |
| Bachur 2016 | 786 | 581 | 84 | 682 | 10.0 | 0.90 [0.88, 0.92] | 0.54 [0.51, 0.57] |
| Benito 2016 | 79 | 45 | 10 | 51 | 10.0 | 0.89 [0.80, 0.94] | 0.53 [0.43, 0.63] |
| Cayrol 2016 | 46 | 33 | 6 | 49 | 10.0 | 0.88 [0.77, 0.96] | 0.60 [0.48, 0.70] |
| Chiang 2020 | 625 | 319 | 93 | 354 | 10.0 | 0.87 [0.84, 0.89] | 0.53 [0.49, 0.56] |
| Esparaz 2019 | 51 | 111 | 5 | 160 | 10.0 | 0.91 [0.80, 0.97] | 0.59 [0.53, 0.65] |
| Fleischman 2013 | 53 | 52 | 12 | 61 | 10.0 | 0.82 [0.70, 0.90] | 0.54 [0.44, 0.63] |
| Goldman 2008 | 98 | 84 | 14 | 112 | 10.0 | 0.88 [0.80, 0.93] | 0.57 [0.50, 0.64] |
| Gonzalez Del Castillo 2016 | 93 | 18 | 79 | 131 | 10.0 | 0.54 [0.46, 0.62] | 0.88 [0.82, 0.93] |
| Kaiser 2018 | 29 | 11 | 10 | 14 | 10.0 | 0.74 [0.58, 0.87] | 0.56 [0.35, 0.76] |
| Khanafer 2016 | 46 | 51 | 8 | 75 | 10.0 | 0.85 [0.73, 0.93] | 0.60 [0.50, 0.68] |
| Ko 1995 | 23 | 7 | 4 | 13 | 10.0 | 0.85 [0.66, 0.96] | 0.65 [0.41, 0.85] |
| Lycopoulou 2005 | 32 | 4 | 10 | 14 | 10.0 | 0.76 [0.61, 0.88] | 0.78 [0.52, 0.94] |
| Malia 2019 | 169 | 113 | 56 | 424 | 10.0 | 0.75 [0.69, 0.81] | 0.79 [0.75, 0.82] |
| Mandeville 2011 | 129 | 49 | 26 | 83 | 10.0 | 0.83 [0.76, 0.89] | 0.63 [0.54, 0.71] |
| Ozgunur 2014 | 32 | 12 | 2 | 3 | 10.0 | 0.94 [0.80, 0.99] | 0.20 [0.04, 0.48] |
| Samuel 2002 | 624 | 113 | 110 | 323 | 10.0 | 0.85 [0.82, 0.88] | 0.74 [0.70, 0.78] |
| Shommu 2018 | 41 | 32 | 5 | 49 | 10.0 | 0.89 [0.76, 0.96] | 0.60 [0.49, 0.71] |
| Thompson 2015 | 425 | 56 | 71 | 46 | 10.0 | 0.86 [0.82, 0.89] | 0.45 [0.35, 0.55] |
| Thompson 2017 | 334 | 243 | 137 | 424 | 10.0 | 0.71 [0.67, 0.75] | 0.64 [0.60, 0.67] |
| van den Broek 2004 | 52 | 8 | 13 | 26 | 10.0 | 0.80 [0.68, 0.89] | 0.76 [0.59, 0.89] |
| Yap 2015 | 102 | 126 | 17 | 129 | 10.0 | 0.86 [0.78, 0.91] | 0.51 [0.44, 0.57] |
| Yazici 2010 | 162 | 39 | 21 | 18 | 10.0 | 0.89 [0.83, 0.93] | 0.32 [0.20, 0.45] |
| Zuniga 2012 | 26 | 37 | 2 | 36 | 10.0 | 0.93 [0.76, 0.99] | 0.49 [0.37, 0.61] |

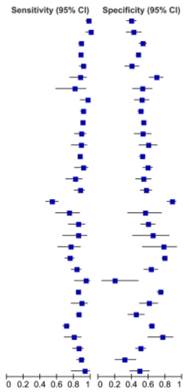


**WCC above 10,000 and below 15,000 /mm^3**

| Study | TP | FP | FN | TN | Threshold | Sensitivity (95% CI) | Specificity (95% CI) |
|---|---|---|---|---|---|---|---|
| Altali 2017 | 92 | 75 | 24 | 140 | 10.5 | 0.79 [0.71, 0.86] | 0.65 [0.58, 0.71] |
| Groselj-Grenc 2007 | 36 | 11 | 13 | 22 | 11.0 | 0.73 [0.59, 0.85] | 0.67 [0.48, 0.82] |
| Hsiao 2005 | 86 | 95 | 25 | 16 | 11.0 | 0.77 [0.69, 0.85] | 0.14 [0.08, 0.22] |
| Mohammed 2004 | 109 | 19 | 21 | 67 | 11.0 | 0.84 [0.76, 0.90] | 0.78 [0.68, 0.86] |
| Ovrebo 1993 | 116 | 34 | 89 | 138 | 11.0 | 0.57 [0.50, 0.63] | 0.80 [0.73, 0.86] |
| Prada Arias 2017 | 118 | 61 | 14 | 82 | 11.0 | 0.89 [0.83, 0.94] | 0.57 [0.49, 0.66] |
| Wu 2012 | 245 | 83 | 61 | 205 | 11.0 | 0.80 [0.75, 0.84] | 0.71 [0.66, 0.76] |
| Kaiser 2018 | 22 | 4 | 17 | 21 | 11.6 | 0.56 [0.40, 0.72] | 0.84 [0.64, 0.95] |
| Andersson 1999 | 28 | 23 | 20 | 98 | 12.0 | 0.58 [0.43, 0.72] | 0.81 [0.73, 0.88] |
| Andersson 2008 | 12 | 22 | 9 | 52 | 12.0 | 0.57 [0.34, 0.78] | 0.70 [0.59, 0.80] |
| Andersson 2014 | 42 | 44 | 12 | 83 | 12.0 | 0.78 [0.64, 0.88] | 0.65 [0.56, 0.74] |
| Andersson 2017 | 268 | 211 | 65 | 476 | 12.0 | 0.80 [0.76, 0.85] | 0.69 [0.66, 0.73] |
| Bachur 2016 | 686 | 409 | 184 | 854 | 12.0 | 0.79 [0.76, 0.82] | 0.68 [0.65, 0.70] |
| Gonzalez Del Castillo 2016 | 74 | 52 | 37 | 158 | 12.0 | 0.67 [0.57, 0.75] | 0.75 [0.69, 0.81] |
| Khanafer 2016 | 35 | 34 | 19 | 92 | 12.0 | 0.65 [0.51, 0.77] | 0.73 [0.64, 0.81] |
| Kwan 2010 | 82 | 32 | 33 | 62 | 12.0 | 0.71 [0.62, 0.79] | 0.66 [0.55, 0.75] |
| Ozgunur 2014 | 29 | 8 | 5 | 7 | 12.0 | 0.85 [0.69, 0.95] | 0.47 [0.21, 0.73] |
| Shommu 2018 | 37 | 21 | 9 | 60 | 12.0 | 0.80 [0.66, 0.91] | 0.74 [0.63, 0.83] |
| Thompson 2015 | 380 | 42 | 116 | 60 | 12.0 | 0.77 [0.73, 0.80] | 0.59 [0.49, 0.68] |
| Thompson 2017 | 281 | 161 | 190 | 506 | 12.0 | 0.60 [0.55, 0.64] | 0.76 [0.72, 0.79] |
| Miguez 2016 | 35 | 14 | 9 | 78 | 12.3 | 0.80 [0.65, 0.90] | 0.85 [0.76, 0.91] |
| Beltran 2007 | 67 | 8 | 4 | 42 | 12.5 | 0.94 [0.86, 0.98] | 0.84 [0.71, 0.93] |
| Diaz Moreno 2012 | 59 | 42 | 26 | 58 | 12.8 | 0.69 [0.58, 0.79] | 0.58 [0.48, 0.68] |
| Rodriguez Sanchez 2013 | 36 | 18 | 5 | 46 | 13.0 | 0.88 [0.74, 0.96] | 0.72 [0.59, 0.82] |
| Tuncer 2019 | 104 | 43 | 33 | 121 | 13.05 | 0.76 [0.68, 0.83] | 0.74 [0.66, 0.80] |
| Oikonomopoulou 2018 | 74 | 33 | 26 | 152 | 13.3 | 0.74 [0.64, 0.82] | 0.82 [0.76, 0.87] |
| Bal 2016 | 38 | 11 | 18 | 29 | 13.4 | 0.68 [0.54, 0.80] | 0.72 [0.56, 0.85] |
| Greer 2019 | 63 | 92 | 23 | 368 | 13.5 | 0.73 [0.63, 0.82] | 0.80 [0.76, 0.84] |
| Kumar 2021 | 25 | 5 | 30 | 60 | 14.0 | 0.45 [0.32, 0.59] | 0.92 [0.83, 0.97] |
| Sanchez-Echaniz 1997 | 73 | 25 | 21 | 76 | 14.0 | 0.78 [0.68, 0.86] | 0.75 [0.66, 0.83] |
| Peltola 1986 | 56 | 11 | 38 | 57 | 14.9 | 0.60 [0.49, 0.70] | 0.84 [0.73, 0.92] |

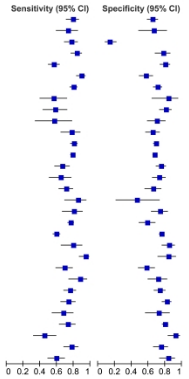


**WCC at or above 15,000 /mm^3**

| Study | TP | FP | FN | TN | Threshold | Sensitivity (95% CI) | Specificity (95% CI) |
|---|---|---|---|---|---|---|---|
| Andersson 1999 | 16 | 5 | 32 | 116 | 15.0 | 0.33 [0.20, 0.48] | 0.96 [0.91, 0.99] |
| Andersson 2008 | 9 | 10 | 12 | 64 | 15.0 | 0.43 [0.22, 0.66] | 0.86 [0.77, 0.93] |
| Andersson 2014 | 20 | 17 | 34 | 110 | 15.0 | 0.37 [0.24, 0.51] | 0.87 [0.79, 0.92] |
| Andersson 2017 | 159 | 103 | 174 | 584 | 15.0 | 0.48 [0.42, 0.53] | 0.85 [0.82, 0.88] |
| Bachur 2016 | 485 | 240 | 385 | 1023 | 15.0 | 0.56 [0.52, 0.59] | 0.81 [0.79, 0.83] |
| Calvo Rigual 1998 | 96 | 22 | 45 | 68 | 15.0 | 0.68 [0.60, 0.76] | 0.76 [0.65, 0.84] |
| Gonzalez Del Castillo 2016 | 44 | 67 | 28 | 182 | 15.0 | 0.61 [0.49, 0.72] | 0.73 [0.67, 0.78] |
| Kaiser 2018 | 17 | 3 | 22 | 22 | 15.0 | 0.44 [0.28, 0.60] | 0.88 [0.69, 0.97] |
| Khanafer 2016 | 27 | 19 | 27 | 107 | 15.0 | 0.50 [0.36, 0.64] | 0.85 [0.77, 0.91] |
| Lin 2009 | 23 | 4 | 30 | 43 | 15.0 | 0.43 [0.30, 0.58] | 0.91 [0.80, 0.98] |
| Ozgunur 2014 | 19 | 8 | 15 | 7 | 15.0 | 0.56 [0.38, 0.73] | 0.47 [0.21, 0.73] |
| Shommu 2018 | 30 | 11 | 16 | 70 | 15.0 | 0.65 [0.50, 0.79] | 0.86 [0.77, 0.93] |
| Thompson 2015 | 252 | 24 | 244 | 78 | 15.0 | 0.51 [0.46, 0.55] | 0.76 [0.67, 0.84] |
| Thompson 2017 | 195 | 82 | 276 | 585 | 15.0 | 0.41 [0.37, 0.46] | 0.88 [0.85, 0.90] |

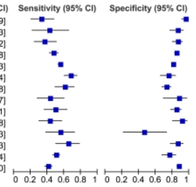


**WCC threshold depending on age**

| Study | TP | FP | FN | TN | Threshold | Sensitivity (95% CI) | Specificity (95% CI) |
|---|---|---|---|---|---|---|---|
| Doraiswamy 1979 | 94 | 5 | 131 | 145 | | 0.42 [0.35, 0.49] | 0.97 [0.92, 0.99] |
| Klein 2021 | 40 | 21 | 13 | 60 | | 0.75 [0.62, 0.86] | 0.74 [0.63, 0.83] |
| Salo 2016 | 13 | 11 | 9 | 11 | | 0.59 [0.36, 0.79] | 0.50 [0.28, 0.72] |
| Turkyilmaz 2006 | 43 | 9 | 18 | 35 | | 0.70 [0.57, 0.81] | 0.80 [0.65, 0.90] |
| Wang 2007 | 50 | 67 | 24 | 269 | | 0.68 [0.56, 0.78] | 0.80 [0.75, 0.84] |

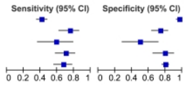


**WCC threshold depending on duration pain**

| Study | TP | FP | FN | TN | Threshold | Sensitivity (95% CI) | Specificity (95% CI) |
|---|---|---|---|---|---|---|---|
| Dadeh 2021 | 73 | 434 | 24 | 512 | | 0.75 [0.65, 0.83] | 0.54 [0.51, 0.57] |

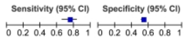


135x264mm (750 x 750 DPI)

**CRP at or below 5 mg/l**

| Study | TP | FP | FN | TN | Threshold | Sensitivity (95% CI) | Specificity (95% CI) |
|---|---|---|---|---|---|---|---|
| Bal 2016 | 50 | 24 | 6 | 16 | 0.9 | 0.89 [0.78, 0.96] | 0.40 [0.25, 0.57] |
| Miguez 2016 | 30 | 29 | 14 | 63 | 3.0 | 0.68 [0.52, 0.81] | 0.68 [0.58, 0.78] |
| Lycopoulou 2005 | 34 | 1 | 8 | 17 | 4.5 | 0.81 [0.66, 0.91] | 0.94 [0.73, 1.00] |
| Akgul 2021 | 146 | 72 | 44 | 59 | 5.0 | 0.77 [0.70, 0.83] | 0.45 [0.36, 0.54] |
| Altali 2017 | 92 | 93 | 24 | 122 | 5.0 | 0.79 [0.71, 0.86] | 0.57 [0.50, 0.63] |
| Andersson 1999 | 29 | 34 | 17 | 85 | 5.0 | 0.63 [0.48, 0.77] | 0.71 [0.62, 0.79] |
| Andersson 2008 | 17 | 55 | 4 | 17 | 5.0 | 0.81 [0.58, 0.95] | 0.24 [0.14, 0.35] |
| Andersson 2014 | 55 | 127 | 0 | 0 | 5.0 | 1.00 [0.94, 1.00] | 0.00 [0.00, 0.03] |
| Andersson 2017 | 285 | 481 | 38 | 211 | 5.0 | 0.88 [0.84, 0.92] | 0.30 [0.27, 0.34] |
| Chiang 2020 | 589 | 394 | 129 | 279 | 5.0 | 0.82 [0.79, 0.85] | 0.41 [0.38, 0.45] |
| Gonzalez Del Castillo 2016 | 88 | 90 | 23 | 120 | 5.0 | 0.79 [0.71, 0.86] | 0.57 [0.50, 0.64] |
| Greer 2019 | 69 | 133 | 17 | 327 | 5.0 | 0.80 [0.70, 0.88] | 0.71 [0.67, 0.75] |
| Kaiser 2018 | 23 | 11 | 16 | 14 | 5.0 | 0.59 [0.42, 0.74] | 0.56 [0.35, 0.76] |
| Malia 2019 | 160 | 118 | 65 | 419 | 5.0 | 0.71 [0.65, 0.77] | 0.78 [0.74, 0.81] |
| Ozgunur 2014 | 32 | 9 | 2 | 6 | 5.0 | 0.94 [0.80, 0.99] | 0.40 [0.16, 0.68] |
| Rodriguez Sanchez 2013 | 21 | 29 | 21 | 35 | 5.0 | 0.50 [0.34, 0.66] | 0.55 [0.42, 0.67] |
| Yap 2015 | 99 | 143 | 20 | 112 | 5.0 | 0.83 [0.75, 0.89] | 0.44 [0.38, 0.50] |

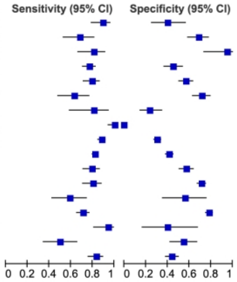


**CRP between 5 and 11 mg/l**

| Study | TP | FP | FN | TN | Threshold | Sensitivity (95% CI) | Specificity (95% CI) |
|---|---|---|---|---|---|---|---|
| Zouari 2016 | 107 | 74 | 32 | 79 | 6.0 | 0.77 [0.69, 0.84] | 0.52 [0.43, 0.60] |
| Naqvi 2019 | 64 | 20 | 15 | 63 | 7.9 | 0.81 [0.71, 0.89] | 0.76 [0.65, 0.85] |
| Mohammed 2004 | 114 | 13 | 16 | 73 | 8.0 | 0.88 [0.81, 0.93] | 0.85 [0.76, 0.92] |
| Groselj-Grenc 2007 | 36 | 15 | 13 | 18 | 9.0 | 0.73 [0.59, 0.85] | 0.55 [0.36, 0.72] |
| Ko 1995 | 19 | 7 | 8 | 13 | 9.0 | 0.70 [0.50, 0.86] | 0.65 [0.41, 0.85] |
| Andersson 1999 | 23 | 26 | 23 | 93 | 10.0 | 0.50 [0.35, 0.65] | 0.78 [0.70, 0.85] |
| Andersson 2008 | 15 | 25 | 6 | 47 | 10.0 | 0.71 [0.48, 0.89] | 0.65 [0.53, 0.76] |
| Andersson 2014 | 43 | 53 | 12 | 74 | 10.0 | 0.78 [0.65, 0.88] | 0.58 [0.49, 0.67] |
| Andersson 2017 | 221 | 249 | 102 | 443 | 10.0 | 0.68 [0.63, 0.73] | 0.64 [0.60, 0.68] |
| Gonzalez Del Castillo 2016 | 75 | 70 | 36 | 140 | 10.0 | 0.68 [0.58, 0.76] | 0.67 [0.60, 0.73] |
| Kaiser 2018 | 20 | 10 | 19 | 15 | 10.0 | 0.51 [0.35, 0.68] | 0.60 [0.39, 0.79] |
| Lin 2009 | 39 | 12 | 14 | 35 | 10.0 | 0.74 [0.60, 0.85] | 0.74 [0.60, 0.86] |
| Lycopoulou 2005 | 26 | 1 | 16 | 17 | 10.0 | 0.62 [0.46, 0.76] | 0.94 [0.73, 1.00] |
| Ovrebo 1993 | 48 | 80 | 5 | 27 | 10.0 | 0.91 [0.79, 0.97] | 0.25 [0.17, 0.35] |
| Ozgunur 2014 | 25 | 7 | 9 | 8 | 10.0 | 0.74 [0.56, 0.87] | 0.53 [0.27, 0.79] |
| Peltola 1986 | 60 | 12 | 34 | 56 | 10.0 | 0.64 [0.53, 0.73] | 0.82 [0.71, 0.91] |
| Kumar 2021 | 49 | 30 | 6 | 35 | 10.5 | 0.89 [0.78, 0.96] | 0.54 [0.41, 0.66] |

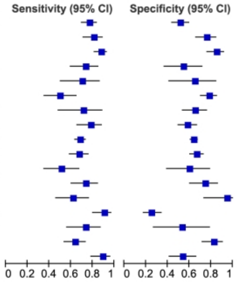


**CRP between 11 and 50 mg/l**

| Study | TP | FP | FN | TN | Threshold | Sensitivity (95% CI) | Specificity (95% CI) |
|---|---|---|---|---|---|---|---|
| Oikonomopoulou 2018 | 60 | 52 | 40 | 133 | 12.5 | 0.60 [0.50, 0.70] | 0.72 [0.65, 0.78] |
| Salo 2016 | 14 | 9 | 8 | 13 | 15.0 | 0.64 [0.41, 0.83] | 0.59 [0.36, 0.79] |
| Ozgunur 2014 | 24 | 5 | 10 | 10 | 16.0 | 0.71 [0.53, 0.85] | 0.67 [0.38, 0.88] |
| Diaz Moreno 2012 | 46 | 31 | 39 | 68 | 18.0 | 0.54 [0.43, 0.65] | 0.69 [0.59, 0.78] |
| Benito 2016 | 31 | 13 | 58 | 83 | 20.0 | 0.35 [0.25, 0.46] | 0.86 [0.78, 0.93] |
| Calvo Rigual 1998 | 61 | 34 | 80 | 56 | 20.0 | 0.43 [0.35, 0.52] | 0.62 [0.51, 0.72] |
| Shommu 2018 | 24 | 9 | 24 | 67 | 20.0 | 0.50 [0.35, 0.65] | 0.88 [0.79, 0.94] |
| Wu 2012 | 214 | 78 | 92 | 210 | 25.0 | 0.70 [0.64, 0.75] | 0.73 [0.67, 0.78] |
| Prada Arias 2017 | 57 | 29 | 75 | 114 | 25.2 | 0.43 [0.35, 0.52] | 0.80 [0.72, 0.86] |
| Andersson 1999 | 14 | 14 | 32 | 105 | 30.0 | 0.30 [0.18, 0.46] | 0.88 [0.81, 0.93] |
| Andersson 2008 | 8 | 11 | 13 | 61 | 30.0 | 0.38 [0.18, 0.62] | 0.85 [0.74, 0.92] |
| Andersson 2014 | 31 | 35 | 24 | 92 | 30.0 | 0.56 [0.42, 0.70] | 0.72 [0.64, 0.80] |
| Andersson 2017 | 140 | 128 | 183 | 564 | 30.0 | 0.43 [0.38, 0.49] | 0.82 [0.78, 0.84] |
| Cayrol 2016 | 20 | 12 | 32 | 70 | 30.0 | 0.38 [0.25, 0.53] | 0.85 [0.76, 0.92] |
| Gonzalez Del Castillo 2016 | 59 | 40 | 52 | 170 | 30.0 | 0.53 [0.43, 0.63] | 0.81 [0.75, 0.86] |
| Kaiser 2018 | 13 | 8 | 26 | 17 | 30.0 | 0.33 [0.19, 0.50] | 0.68 [0.46, 0.85] |
| Kwan 2010 | 81 | 33 | 34 | 61 | 30.0 | 0.70 [0.61, 0.79] | 0.65 [0.54, 0.74] |
| Sanchez-Echaniz 1997 | 40 | 8 | 54 | 93 | 30.0 | 0.43 [0.32, 0.53] | 0.92 [0.85, 0.97] |
| Beltran 2007 | 68 | 4 | 3 | 46 | 47.0 | 0.96 [0.88, 0.99] | 0.92 [0.81, 0.98] |

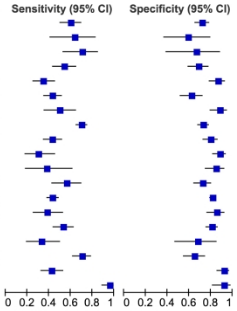


**CRP at or above 50 mg/l**

| Study | TP | FP | FN | TN | Threshold | Sensitivity (95% CI) | Specificity (95% CI) |
|---|---|---|---|---|---|---|---|
| Andersson 1999 | 7 | 10 | 39 | 109 | 50.0 | 0.15 [0.06, 0.29] | 0.92 [0.85, 0.96] |
| Andersson 2008 | 5 | 7 | 16 | 65 | 50.0 | 0.24 [0.08, 0.47] | 0.90 [0.81, 0.96] |
| Andersson 2014 | 20 | 21 | 35 | 106 | 50.0 | 0.36 [0.24, 0.50] | 0.83 [0.76, 0.89] |
| Andersson 2017 | 93 | 62 | 230 | 630 | 50.0 | 0.29 [0.24, 0.34] | 0.91 [0.89, 0.93] |
| Gonzalez Del Castillo 2016 | 37 | 28 | 74 | 182 | 50.0 | 0.33 [0.25, 0.43] | 0.87 [0.81, 0.91] |
| Kaiser 2018 | 9 | 2 | 30 | 23 | 50.0 | 0.23 [0.11, 0.39] | 0.92 [0.74, 0.99] |
| Ko 1995 | 14 | 1 | 13 | 19 | 50.0 | 0.52 [0.32, 0.71] | 0.95 [0.75, 1.00] |
| Ozgunur 2014 | 15 | 3 | 19 | 12 | 50.0 | 0.44 [0.27, 0.62] | 0.80 [0.52, 0.96] |
| Shommu 2018 | 12 | 1 | 36 | 75 | 50.0 | 0.25 [0.14, 0.40] | 0.99 [0.93, 1.00] |
| Wu 2012 | 214 | 49 | 92 | 239 | 89.0 | 0.70 [0.64, 0.75] | 0.83 [0.78, 0.87] |

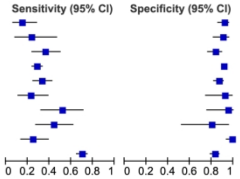


173x265mm (300 x 300 DPI)

| Study | TP | FP | FN | TN | Threshold | Sensitivity (95% CI) | Specificity (95% CI) |
|---|---|---|---|---|---|---|---|
| Salo 2016 | 16 | 7 | 6 | 15 | age dependent | 0.73 [0.50, 0.89] | 0.68 [0.45, 0.86] |
| Altali 2017 | 99 | 85 | 17 | 130 | 6.6 | 0.85 [0.78, 0.91] | 0.60 [0.54, 0.67] |
| Kharbanda 2012 | 942 | 824 | 76 | 783 | 6.73 | 0.93 [0.91, 0.94] | 0.49 [0.46, 0.51] |
| Fleischman 2013 | 53 | 56 | 12 | 57 | 6.75 | 0.82 [0.70, 0.90] | 0.50 [0.41, 0.60] |
| Kharbanda 2005 | 152 | 131 | 5 | 137 | 6.75 | 0.97 [0.93, 0.99] | 0.51 [0.45, 0.57] |
| Prada Arias 2017 | 119 | 57 | 13 | 86 | 7.0 | 0.90 [0.84, 0.95] | 0.60 [0.52, 0.68] |
| Akgul 2021 | 163 | 74 | 27 | 56 | 7.5 | 0.86 [0.80, 0.90] | 0.43 [0.34, 0.52] |
| Andersson 1999 | 34 | 38 | 2 | 61 | 7.5 | 0.94 [0.81, 0.99] | 0.62 [0.51, 0.71] |
| Andersson 2008 | 8 | 27 | 1 | 33 | 7.5 | 0.89 [0.52, 1.00] | 0.55 [0.42, 0.68] |
| Andersson 2014 | 35 | 32 | 3 | 41 | 7.5 | 0.92 [0.79, 0.98] | 0.56 [0.44, 0.68] |
| Andersson 2017 | 274 | 282 | 31 | 322 | 7.5 | 0.90 [0.86, 0.93] | 0.53 [0.49, 0.57] |
| Benito 2016 | 78 | 37 | 11 | 59 | 7.5 | 0.88 [0.79, 0.94] | 0.61 [0.51, 0.71] |
| Goldman 2008 | 84 | 67 | 17 | 121 | 7.5 | 0.83 [0.74, 0.90] | 0.64 [0.57, 0.71] |
| Greer 2019 | 69 | 115 | 17 | 345 | 7.5 | 0.80 [0.70, 0.88] | 0.75 [0.71, 0.79] |
| Samuel 2002 | 595 | 122 | 139 | 314 | 7.5 | 0.81 [0.78, 0.84] | 0.72 [0.68, 0.76] |
| Shommu 2018 | 40 | 24 | 6 | 57 | 7.5 | 0.87 [0.74, 0.95] | 0.70 [0.59, 0.80] |
| Thompson 2015 | 351 | 68 | 47 | 45 | 7.5 | 0.88 [0.85, 0.91] | 0.40 [0.31, 0.49] |
| Thompson 2017 | 319 | 206 | 141 | 457 | 7.5 | 0.69 [0.65, 0.74] | 0.69 [0.65, 0.72] |
| Zuniga 2012 | 27 | 37 | 1 | 36 | 7.5 | 0.96 [0.82, 1.00] | 0.49 [0.37, 0.61] |
| Corkum 2018 | 116 | 166 | 40 | 416 | 8.0 | 0.74 [0.67, 0.81] | 0.71 [0.68, 0.75] |
| Bal 2016 | 43 | 13 | 13 | 27 | 9.4 | 0.77 [0.64, 0.87] | 0.68 [0.51, 0.81] |
| Miguez 2016 | 37 | 16 | 7 | 76 | 9.6 | 0.84 [0.70, 0.93] | 0.83 [0.73, 0.90] |
| Bachur 2016 | 630 | 365 | 240 | 898 | 10.0 | 0.72 [0.69, 0.75] | 0.71 [0.69, 0.74] |
| Kumar 2021 | 28 | 8 | 27 | 57 | 10.48 | 0.51 [0.37, 0.65] | 0.88 [0.77, 0.95] |
| Oikonomopoulou 2018 | 71 | 26 | 29 | 159 | 10.8 | 0.71 [0.61, 0.80] | 0.86 [0.80, 0.91] |

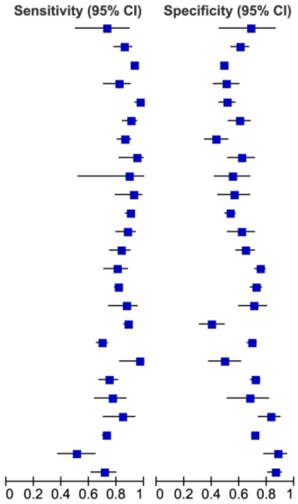


195x102mm (300 x 300 DPI)

| Study | TP | FP | FN | TN | Threshold | Sensitivity (95% CI) | Specificity (95% CI) |
|---|---|---|---|---|---|---|---|
| Anadalwar 2015 | 340 | 241 | 53 | 211 | 65% | 0.87 [0.83, 0.90] | 0.47 [0.42, 0.51] |
| Salo 2016 | 16 | 10 | 6 | 12 | 71% | 0.73 [0.50, 0.89] | 0.55 [0.32, 0.76] |
| Andersson 1999 | 31 | 45 | 5 | 54 | 75% | 0.86 [0.71, 0.95] | 0.55 [0.44, 0.65] |
| Andersson 2008 | 9 | 29 | 3 | 31 | 75% | 0.75 [0.43, 0.95] | 0.52 [0.38, 0.65] |
| Andersson 2014 | 30 | 29 | 8 | 44 | 75% | 0.79 [0.63, 0.90] | 0.60 [0.48, 0.72] |
| Chiang 2020 | 524 | 224 | 194 | 449 | 75% | 0.73 [0.70, 0.76] | 0.67 [0.63, 0.70] |
| Dadeh 2021 | 74 | 304 | 23 | 642 | 75% | 0.76 [0.67, 0.84] | 0.68 [0.65, 0.71] |
| Fleischman 2013 | 50 | 48 | 15 | 65 | 75% | 0.77 [0.65, 0.86] | 0.58 [0.48, 0.67] |
| Hsiao 2005 | 87 | 67 | 24 | 44 | 75% | 0.78 [0.70, 0.86] | 0.40 [0.30, 0.49] |
| Klein 2021 | 36 | 21 | 17 | 60 | 75% | 0.68 [0.54, 0.80] | 0.74 [0.63, 0.83] |
| Ko 1995 | 22 | 6 | 5 | 14 | 75% | 0.81 [0.62, 0.94] | 0.70 [0.46, 0.88] |
| Mandeville 2011 | 119 | 48 | 36 | 84 | 75% | 0.77 [0.69, 0.83] | 0.64 [0.55, 0.72] |
| Mohammed 2004 | 111 | 15 | 19 | 71 | 75% | 0.85 [0.78, 0.91] | 0.83 [0.73, 0.90] |
| Yap 2015 | 79 | 80 | 40 | 175 | 75% | 0.66 [0.57, 0.75] | 0.69 [0.63, 0.74] |
| Wang 2007 | 44 | 34 | 30 | 302 | 80% | 0.59 [0.47, 0.71] | 0.90 [0.86, 0.93] |
| Doraiswamy 1979 | 216 | 47 | 9 | 103 | age dependent | 0.96 [0.93, 0.98] | 0.69 [0.61, 0.76] |

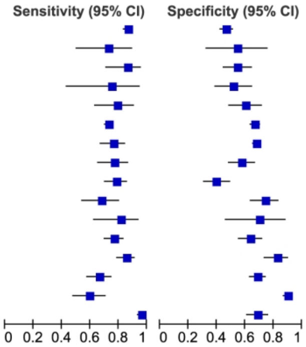


195x71mm (300 x 300 DPI)

| Study | TP | FP | FN | TN | Threshold | Sensitivity (95% CI) | Specificity (95% CI) |
|---|---|---|---|---|---|---|---|
| Benito 2016 | 34 | 34 | 55 | 62 | 0.1 | 0.38 [0.28, 0.49] | 0.65 [0.54, 0.74] |
| Bal 2016 | 18 | 4 | 38 | 36 | 0.37 | 0.32 [0.20, 0.46] | 0.90 [0.76, 0.97] |
| Khan 2012 | 6 | 2 | 16 | 26 | 0.39 | 0.27 [0.11, 0.50] | 0.93 [0.76, 0.99] |
| Rodriguez Sanchez 2013 | 10 | 18 | 34 | 46 | 0.5 | 0.23 [0.11, 0.38] | 0.72 [0.59, 0.82] |
| Kouame 2005 | 19 | 4 | 49 | 29 | 0.5 | 0.28 [0.18, 0.40] | 0.88 [0.72, 0.97] |
| Akgul 2021 | 55 | 19 | 135 | 111 | 0.5 | 0.29 [0.23, 0.36] | 0.85 [0.78, 0.91] |

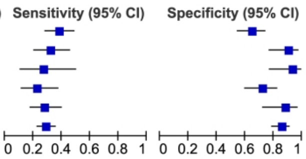


195x34mm (300 x 300 DPI)

**CRP and WCC**

| Study | TP | FP | FN | TN | Threshold WCC - CRP | Sensitivity (95% CI) | Specificity (95% CI) |
|---|---|---|---|---|---|---|---|
| Andersson 1999 | 20 | 12 | 26 | 107 | 10.0 - 10 | 0.43 [0.29, 0.59] | 0.90 [0.83, 0.95] |
| Andersson 2008 | 12 | 16 | 9 | 56 | 10.0 - 10 | 0.57 [0.34, 0.78] | 0.78 [0.66, 0.87] |
| Andersson 2014 | 39 | 31 | 14 | 96 | 10.0 - 10 | 0.74 [0.60, 0.85] | 0.76 [0.67, 0.83] |
| Andersson 2017 | 197 | 150 | 122 | 521 | 10.0 - 10 | 0.62 [0.56, 0.67] | 0.78 [0.74, 0.81] |
| Calvo Rigual 1998 | 113 | 45 | 28 | 45 | 15.0 - 20 | 0.80 [0.73, 0.86] | 0.50 [0.39, 0.61] |
| Kaiser 2018 | 16 | 7 | 23 | 18 | 10.0 - 10 | 0.41 [0.26, 0.58] | 0.72 [0.51, 0.88] |
| Kwan 2010 | 48 | 8 | 67 | 86 | 12.0 - 30 | 0.42 [0.33, 0.51] | 0.91 [0.84, 0.96] |
| Ovrebo 1993 | 38 | 30 | 14 | 77 | 11.0- 10 | 0.73 [0.59, 0.84] | 0.72 [0.62, 0.80] |
| Ozgunur 2014 | 24 | 5 | 10 | 10 | 10.0 - 10 | 0.71 [0.53, 0.85] | 0.67 [0.38, 0.88] |
| Peltola 1986 | 42 | 20 | 52 | 48 | 14.9 - 100 | 0.45 [0.34, 0.55] | 0.71 [0.58, 0.81] |
| Sanchez-Echaniz 1997 | 32 | 5 | 62 | 96 | 14.0 - 30 | 0.34 [0.25, 0.45] | 0.95 [0.89, 0.98] |

**CRP or WCC**

| Study | TP | FP | FN | TN | Threshold WCC - CRP | Sensitivity (95% CI) | Specificity (95% CI) |
|---|---|---|---|---|---|---|---|
| Andersson 1999 | 44 | 50 | 2 | 69 | 10.0 - 10 | 0.96 [0.85, 0.99] | 0.58 [0.49, 0.67] |
| Andersson 2008 | 20 | 43 | 1 | 29 | 10.0 - 10 | 0.95 [0.76, 1.00] | 0.40 [0.29, 0.53] |
| Andersson 2014 | 53 | 83 | 0 | 44 | 10.0 - 10 | 1.00 [0.93, 1.00] | 0.35 [0.26, 0.44] |
| Andersson 2017 | 313 | 425 | 6 | 246 | 10.0 - 10 | 0.98 [0.96, 0.99] | 0.37 [0.33, 0.40] |
| Chiang 2020 | 716 | 514 | 2 | 159 | 10.0 - 5 | 1.00 [0.99, 1.00] | 0.24 [0.20, 0.27] |
| Huckins 2013 | 140 | 238 | 4 | 121 | 10.0 - 8 | 0.97 [0.93, 0.99] | 0.34 [0.29, 0.39] |
| Huckins 2016 | 457 | 775 | 19 | 635 | 10.0 - 8 | 0.96 [0.94, 0.98] | 0.45 [0.42, 0.48] |
| Kaiser 2018 | 33 | 14 | 6 | 11 | 10.0 - 10 | 0.85 [0.69, 0.94] | 0.44 [0.24, 0.65] |
| Ovrebo 1993 | 62 | 86 | 0 | 21 | 11.0- 10 | 1.00 [0.94, 1.00] | 0.20 [0.13, 0.28] |
| Ozgunur 2014 | 33 | 14 | 1 | 1 | 10.0 - 10 | 0.97 [0.85, 1.00] | 0.07 [0.00, 0.32] |

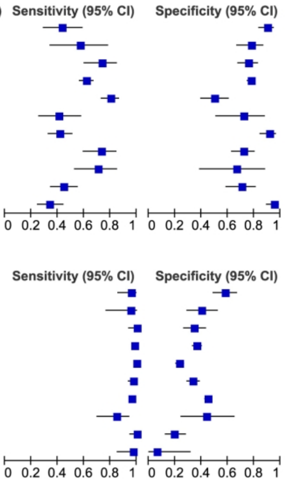


195x111mm (300 x 300 DPI)

**WCC, CRP, and Neutrophils**

| Study | TP | FP | FN | TN | Threshold WCC - CRP - NC% | Sensitivity (95% CI) | Specificity (95% CI) |
|---|---|---|---|---|---|---|---|
| Andersson 1999 | 15 | 11 | 20 | 88 | 10.0 - 10 - 75% | 0.43 [0.26, 0.61] | 0.89 [0.81, 0.94] |
| Andersson 2008 | 6 | 11 | 6 | 48 | 10.0 - 10 - 75% | 0.50 [0.21, 0.79] | 0.81 [0.69, 0.90] |
| Andersson 2014 | 21 | 12 | 17 | 61 | 10.0 - 10 - 75% | 0.55 [0.38, 0.71] | 0.84 [0.73, 0.91] |
| Andersson 2017 | 139 | 89 | 152 | 498 | 10.0 - 10 - 75% | 0.48 [0.42, 0.54] | 0.85 [0.82, 0.88] |
| Mohammed 2004 | 112 | 8 | 18 | 78 | 11.0 - 80 - 75% | 0.86 [0.79, 0.92] | 0.91 [0.82, 0.96] |

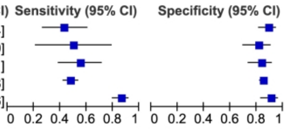


**WCC, CRP, or Neutrophils**

| Study | TP | FP | FN | TN | Threshold WCC - CRP - NC% | Sensitivity (95% CI) | Specificity (95% CI) |
|---|---|---|---|---|---|---|---|
| Yap 2015 | 119 | 192 | 0 | 63 | 10.0 - 5 - 75% | 1.00 [0.97, 1.00] | 0.25 [0.20, 0.30] |

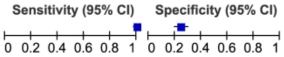


195x59mm (300 x 300 DPI)

# PRISMA-DTA Checklist

| Section/topic | # | PRISMA-DTA Checklist Item | Reported on page # |
|---|---|---|---|
| **TITLE / ABSTRACT** | | | |
| Title | 1 | Identify the report as a systematic review (+/- meta-analysis) of diagnostic test accuracy (DTA) studies. | 1 |
| Abstract | 2 | Abstract: See PRISMA-DTA for abstracts. | 2 |
| **INTRODUCTION** | | | |
| Rationale | 3 | Describe the rationale for the review in the context of what is already known. | 4 |
| Clinical role of index test | D1 | State the scientific and clinical background, including the intended use and clinical role of the index test, and if applicable, the rationale for minimally acceptable test accuracy (or minimum difference in accuracy for comparative design). | 4 and 5 |
| Objectives | 4 | Provide an explicit statement of question(s) being addressed in terms of participants, index test(s), and target condition(s). | 5 and 6 |
| **METHODS** | | | |
| Protocol and registration | 5 | Indicate if a review protocol exists, if and where it can be accessed (e.g., Web address), and, if available, provide registration information including registration number. | 6 |
| Eligibility criteria | 6 | Specify study characteristics (participants, setting, index test(s), reference standard(s), target condition(s), and study design) and report characteristics (e.g., years considered, language, publication status) used as criteria for eligibility, giving rationale. | 6 and 10 |
| Information sources | 7 | Describe all information sources (e.g., databases with dates of coverage, contact with study authors to identify additional studies) in the search and date last searched. | 6 and 7 and supplementary materials |
| Search | 8 | Present full search strategies for all electronic databases and other sources searched, including any limits used, such that they could be repeated. | 6 and supplementary materials |

For peer review only
For peer review only - http://bmjopen.bmj.com/site/about/guidelines.xhtml

# PRISMA-DTA Checklist

| Section/topic | # | PRISMA-DTA Checklist Item | Reported on page # |
|---|---|---|---|
| Study selection | 9 | State the process for selecting studies (i.e., screening, eligibility, included in systematic review, and, if applicable, included in the meta-analysis). | 7 |
| Data collection process | 10 | Describe method of data extraction from reports (e.g., piloted forms, independently, in duplicate) and any processes for obtaining and confirming data from investigators. | 7 |
| Definitions for data extraction | 11 | Provide definitions used in data extraction and classifications of target condition(s), index test(s), reference standard(s) and other characteristics (e.g. study design, clinical setting). | 6 and 10 |
| Risk of bias and applicability | 12 | Describe methods used for assessing risk of bias in individual studies and concerns regarding the applicability to the review question. | 7 |
| Diagnostic accuracy measures | 13 | State the principal diagnostic accuracy measure(s) reported (e.g. sensitivity, specificity) and state the unit of assessment (e.g. per-patient, per-lesion). | 8 |
| Synthesis of results | 14 | Describe methods of handling data, combining results of studies and describing variability between studies. This could include, but is not limited to: a) handling of multiple definitions of target condition. b) handling of multiple thresholds of test positivity, c) handling multiple index test readers, d) handling of indeterminate test results, e) grouping and comparing tests, f) handling of different reference standards | 7 and 8 |



| Section/topic | # | PRISMA-DTA Checklist Item | Reported on page # |
|---|---|---|---|
| Meta-analysis | D2 | Report the statistical methods used for meta-analyses, if performed. | 8 and 9 |
| Additional analyses | 16 | Describe methods of additional analyses (e.g., sensitivity or subgroup analyses, meta-regression), if done, indicating which were pre-specified. | 8 |
| **RESULTS** | | | |
| Study selection | 17 | Provide numbers of studies screened, assessed for eligibility, included in the review (and included in meta-analysis, if applicable) with reasons for exclusions at each stage, ideally with a flow diagram. | Figure 1, 10 and supplementary materials |

For peer review only - http://bmjopen.bmj.com/site/about/guidelines.xhtml

# PRISMA-DTA Checklist

| | | | |
|---|---|---|---|
| Study characteristics | 18 | For each included study provide citations and present key characteristics including: a) participant characteristics (presentation, prior testing), b) clinical setting, c) study design, d) target condition definition, e) index test, f) reference standard, g) sample size, h) funding sources | 10 |
| Risk of bias and applicability | 19 | Present evaluation of risk of bias and concerns regarding applicability for each study. | 10 |
| Results of individual studies | 20 | For each analysis in each study (e.g. unique combination of index test, reference standard, and positivity threshold) report 2x2 data (TP, FP, FN, TN) with estimates of diagnostic accuracy and confidence intervals, ideally with a forest or receiver operator characteristic (ROC) plot. | 11,12 and 13 |
| Synthesis of results | 21 | Describe test accuracy, including variability; if meta-analysis was done, include results and confidence intervals. | 13 and 14 |
| Additional analysis | 23 | Give results of additional analyses, if done (e.g., sensitivity or subgroup analyses, meta-regression; analysis of index test: failure rates, proportion of inconclusive results, adverse events). | 13 |
| **DISCUSSION** | | | |
| Summary of evidence | 24 | Summarize the main findings including the strength of evidence. | 15 |
| Limitations | 25 | Discuss limitations from included studies (e.g. risk of bias and concerns regarding applicability) and from the review process (e.g. incomplete retrieval of identified research). | 16 and 17 |
| Conclusions | 26 | Provide a general interpretation of the results in the context of other evidence. Discuss implications for future research and clinical practice (e.g. the intended use and clinical role of the index test). | 18 |
| **FUNDING** | | | |
| Funding | 27 | For the systematic review, describe the sources of funding and other support and the role of the funders. | 21 |

For Peer Review Only

*Adapted From:* McInnes MDF, Moher D, Thombs BD, McGrath TA, Bossuyt PM, The PRISMA-DTA Group (2018). Preferred Reporting Items for a Systematic Review and Meta-analysis of Diagnostic Test Accuracy Studies: The PRISMA-DTA Statement. JAMA. 2018 Jan 23;319(4):388-396. doi: 10.1001/jama.2017.19163.

For more information, visit: www.prisma-statement.org.



For peer review only - http://bmjopen.bmj.com/site/about/guidelines.xhtml

# PRISMA 2020 Checklist

| Section and Topic | Item # | Checklist item | Location where item is reported |
|---|---|---|---|
| **TITLE** | | | |
| Title | 1 | Identify the report as a systematic review. | 1 |
| **ABSTRACT** | | | |
| Abstract | 2 | See the PRISMA 2020 for Abstracts checklist. | 2 |
| **INTRODUCTION** | | | |
| Rationale | 3 | Describe the rationale for the review in the context of existing knowledge. | 4 |
| Objectives | 4 | Provide an explicit statement of the objective(s) or question(s) the review addresses. | 5 |
| **METHODS** | | | |
| Eligibility criteria | 5 | Specify the inclusion and exclusion criteria for the review and how studies were grouped for the syntheses. | 6 |
| Information sources | 6 | Specify all databases, registers, websites, organisations, reference lists and other sources searched or consulted to identify studies. Specify the date when each source was last searched or consulted. | 6 |
| Search strategy | 7 | Present the full search strategies for all databases, registers and websites, including any filters and limits used. | 6, Supplementary materials pages 21 and 22 |
| Selection process | 8 | Specify the methods used to decide whether a study met the inclusion criteria of the review, including how many reviewers screened each record and each report retrieved, whether they worked independently, and if applicable, details of automation tools used in the process. | 7 and 8 |
| Data collection process | 9 | Specify the methods used to collect data from reports, including how many reviewers collected data from each report, whether they worked independently, any processes for obtaining or confirming data from study investigators, and if applicable, details of automation tools used in the process. | 7 and 8 |
| Data items | 10a | List and define all outcomes for which data were sought. Specify whether all results that were compatible with each outcome domain in each study were sought (e.g. for all measures, time points, analyses), and if not, the methods used to decide which results to collect. | 8 |
| | 10b | List and define all other variables for which data were sought (e.g. participant and intervention characteristics, funding sources). Describe any assumptions made about any missing or unclear information. | 7 |
| Study risk of bias assessment | 11 | Specify the methods used to assess risk of bias in the included studies, including details of the tool(s) used, how many reviewers assessed each study and whether they worked independently, and if applicable, details of automation tools used in the process. | 7 and 8 |
| Effect measures | 12 | Specify for each outcome the effect measure(s) (e.g. risk ratio, mean difference) used in the synthesis or presentation of results. | 8 |
| Synthesis methods | 13a | Describe the processes used to decide which studies were eligible for each synthesis (e.g. tabulating the study intervention characteristics and comparing against the planned groups for each synthesis (item #5)). | 7 |
| | 13b | Describe any methods required to prepare the data for presentation or synthesis, such as handling of missing summary statistics, or data conversions. | 7 and 8 |
| | 13c | Describe any methods used to tabulate or visually display results of individual studies and syntheses. | 8 and 9 |
| | 13d | Describe any methods used to synthesize results and provide a rationale for the choice(s). If meta-analysis was performed, describe the model(s), method(s) to identify the presence and extent of statistical heterogeneity, and software package(s) used. | 8 and 9 |
| | 13e | Describe any methods used to explore possible causes of heterogeneity among study results (e.g. subgroup analysis, meta-regression). | 8 |

For peer review only - http://bmjopen.bmj.com/site/about/guidelines.xhtml

# PRISMA 2020 Checklist

| Section and Topic | Item # | Checklist item | Location where item is reported |
|---|---|---|---|
| | 13f | Describe any sensitivity analyses conducted to assess robustness of the synthesized results. | 8 and 9 |
| Reporting bias assessment | 14 | Describe any methods used to assess risk of bias due to missing results in a synthesis (arising from reporting biases). | 7 and 8 |
| Certainty assessment | 15 | Describe any methods used to assess certainty (or confidence) in the body of evidence for an outcome. | 8 and 9 |
| **RESULTS** | | | |
| Study selection | 16a | Describe the results of the search and selection process, from the number of records identified in the search to the number of studies included in the review, ideally using a flow diagram. | 10, Figure 1 and Supplementary Table 1 |
| | 16b | Cite studies that might appear to meet the inclusion criteria, but which were excluded, and explain why they were excluded. | Supplementary Table 1 |
| Study characteristics | 17 | Cite each included study and present its characteristics. | Supplementary Table 2 |
| Risk of bias in studies | 18 | Present assessments of risk of bias for each included study. | 10 and Figure 2 |
| Results of individual studies | 19 | For all outcomes, present, for each study: (a) summary statistics for each group (where appropriate) and (b) an effect estimate and its precision (e.g. confidence/credible interval), ideally using structured tables or plots. | 11, 12, 13 and Supplementary Table 5 |
| Results of syntheses | 20a | For each synthesis, briefly summarise the characteristics and risk of bias among contributing studies. | Supplementary Table 4 |
| | 20b | Present results of all statistical syntheses conducted. If meta-analysis was done, present for each the summary estimate and its precision (e.g. confidence/credible interval) and measures of statistical heterogeneity. If comparing groups, describe the direction of the effect. | 11,12,13 and 14 |
| | 20c | Present results of all investigations of possible causes of heterogeneity among study results. | 10 and 11 |
| | 20d | Present results of all sensitivity analyses conducted to assess the robustness of the synthesized results. | 11,12,13 and 14 |
| Reporting biases | 21 | Present assessments of risk of bias due to missing results (arising from reporting biases) for each synthesis assessed. | 10, Figure 2 and Supplementary table 4 |
| Certainty of evidence | 22 | Present assessments of certainty (or confidence) in the body of evidence for each outcome assessed. | 11, 12, 13 and 14 |
| **DISCUSSION** | | | |
| Discussion | 23a | Provide a general interpretation of the results in the context of other evidence. | 17 |
| | 23b | Discuss any limitations of the evidence included in the review. | 16 |
| | 23c | Discuss any limitations of the review processes used. | 16 and 17 |
| | 23d | Discuss implications of the results for practice, policy, and future research. | 17 |
| **OTHER INFORMATION** | | | |

For peer review only

# PRISMA 2020 Checklist

| Section and Topic | Item # | Checklist item | Location where item is reported |
|---|---|---|---|
| Registration and protocol | 24a | Provide registration information for the review, including register name and registration number, or state that the review was not registered. | 21 |
| | 24b | Indicate where the review protocol can be accessed, or state that a protocol was not prepared. | 6 |
| | 24c | Describe and explain any amendments to information provided at registration or in the protocol. | 6 |
| Support | 25 | Describe sources of financial or non-financial support for the review, and the role of the funders or sponsors in the review. | 21 |
| Competing interests | 26 | Declare any competing interests of review authors. | 21 |
| Availability of data, code and other materials | 27 | Report which of the following are publicly available and where they can be found: template data collection forms; data extracted from included studies; data used for all analyses; analytic code; any other materials used in the review. | Available in supplementary materials |

*From:* Page MJ, McKenzie JE, Bossuyt PM, Boutron I, Hoffmann TC, Mulrow CD, et al. The PRISMA 2020 statement: an updated guideline for reporting systematic reviews. BMJ 2021;372:n71. doi: 10.1136/bmj.n71

For more information, visit: http://www.prisma-statement.org/

For peer review only
